# Identification of Three Autophagy-Related Long Non-Coding RNAs as a Novel Head and Neck Squamous Cell Carcinoma Prognostic Signature

**DOI:** 10.3389/fonc.2020.603864

**Published:** 2021-01-26

**Authors:** Ya Guo, Peng Tao Yang, Zhong Wei Wang, Kun Xu, Wei Hua Kou, Heng Luo

**Affiliations:** Department of Radiation Oncology, Second Affiliated Hospital, Xi’an Jiaotong University, Xi’an, China

**Keywords:** head and neck squamous cell carcinoma, autophagy, long non-coding RNA, prognosis, The Cancer Genome Atlas

## Abstract

Head and neck squamous cell carcinoma (HNSCC) has a poor prognosis. Considerable evidence indicates that autophagy and non-coding RNA play essential roles in the biological processes involved in cancers, but associations between autophagy-related long non-coding RNAs (lncRNAs) and HNSCC remain unclear. In the present study, HNSCC RNA sequences and autophagy-related gene data were extracted from The Cancer Genome Atlas database and the Human Autophagy Database. A total of 1,153 autophagy-related lncRNAs were selected *via* calculating Pearson’s correlation coefficient. Three prognosis-related autophagy lncRNAs were identified *via* univariate Cox regression, least absolute shrinkage and selection operator analysis, and multivariate Cox regression analysis. We also constructed a prognostic model based on these autophagy-related lncRNAs and evaluated its ability to accurately and independently predict the prognosis of HNSCC patients. The area under the curve (AUC) was 0.864 (3-year) and 0.836 (5-year), and our model can independently predict the prognosis of HNSCC. The prognostic value of the three autophagy lncRNAs was confirmed *via* analysis of samples from five databases. To further identify the functions of the three lncRNAs, a co-expression network was constructed and pathway analysis was performed. In that analysis the lncRNAs were correlated with 189 related genes and 20 autophagy-related genes, and these lncRNAs mainly involved homologous recombination, the Fanconi anemia pathway, the autophagy-related pathway, and immune-related pathways. In addition, we validated the expression levels of three lncRNAs and autophagy markers (ATG12, BECN1, and MAP1LC3B) based on TIMER, Oncomine, and HPA database analysis. Our results indicated that TTTY15 was increased in HPV positive and HPV negative HNSCC patients, and three autophagy markers were up-regulated in all HNSCCC patients. Lastly, association between three lncRNAs and autophagy markers was performed, and our results showed that TTTY15 and MIF-AS1 were associated with autophagy markers. Collectively, these results suggested that three autophagy-related lncRNAs have prognostic value in HNSCC patients.

## Introduction

Head and neck squamous cell carcinoma (HNSCC) is the sixth most common cancer in the world and accounts for more than 90% of head and neck cancers (1). Advances in surgical treatment combined with radiotherapy, chemotherapy, or biotherapy have resulted in improved HNSCC prognoses, but advanced-stage HNSCC is still associated with an unfavorable prognosis and a high mortality rate (2, 3). Prognoses are mainly based on the TNM classification system, but that system currently does not incorporate enough information from which to derive accurate prognosis (4). Therefore, the identification of effective biomarkers for early diagnosis, assessment, and prognosis of HNSCC is essential.

Autophagy is a self-degradative process that maintains cellular homeostasis. It plays complex roles at different stages of tumorigenesis, and it may promote oncogenesis in some contexts but participate in tumor suppression in others (5). Clarifying the mechanisms involved in autophagy may contribute to the development of new cancer treatment strategies. Several studies suggest that inhibiting autophagy in patients with advanced cancers may enhance the treatment of malignancy, and that the promotion of autophagy in HNSCC can protect cells (6). Therefore, further attention to elucidate the roles and clarify the mechanisms of autophagy in HNSCC is needed.

Long non-coding RNAs (lncRNAs) are defined as non-coding RNAs that are ≥ 200 nucleotides in length, and they are reportedly associated with HNSCC prognosis (5, 7). Previous studies suggest that lncRNAs have an important role in the regulation of autophagy *via* the mediation of autophagy-related gene (ATG) expression (8, 9). Increasing evidence indicates that autophagy and lncRNAs play a key role in tumorigenesis and cancer cell survival, but whether lncRNAs modulate autophagy in HNSCC remains unknown, and the molecular mechanisms involved in autophagy-related lncRNA in HNSCC have not been elucidated (10).

The aim of the present study was to identify autophagy-related lncRNAs, and evaluate their prognostic value with respect to HNSCC. HNSCC RNA sequencing and ATG data from The Cancer Genome Atlas (TCGA) database and the Human Autophagy Database were extracted and analyzed to identify autophagy-related lncRNAs (11, 12). Univariate Cox regression analysis and least absolute shrinkage and selection operator analysis were performed to identify key autophagy related lncRNAs, which were then used to construct a prognostic model using multivariate Cox regression analysis. Receiver operating characteristic (ROC) curves and Kaplan-Meier survival curves were plotted, and concordance indexes were calculated to evaluate the prognostic accuracy of the models. The prognostic value of three lncRNAs was then investigated *via* analyses incorporating five databases. To putatively predict the function of these three lncRNAs, a co-expression network was constructed and pathway analysis was performed. Moreover, the mRNA and protein expression of three lncRNAs and autophagy markers ATG12, BECN1, and MAP1LC3B were measured through analysis in Tumor Immune Estimation Resource (TIMER), Oncomine, and Human Protein Atlas (HPA) database. Lastly, we analyzed the correlation between three lncRNAs and autophagy makers.

## Materials and Methods

### Data Collection and Preparation

Raw RNA sequencing data and corresponding clinical data from HNSCC patients were obtained from the TCGA database (https://portal.gdc.cancer.gov/) (11). These HNSCC samples were mainly derived from patients with laryngeal cancer, nasopharyngeal cancer, tonsil cancer, and lip cancer. As well as 155 HNSCC samples, the gene expression profiles of 12 normal control samples were included in the study. Data post-processing and clinical information extraction were performed using a programming language (strawberry-perl-5.32.0.1-64bit.msi, http://www.perl.org) (13). Protein-coding genes and lncRNAs were annotated and classified using Ensembl human genome browser GRCh38.p13 (http://asia.ensembl.org/index.html) (14). Prior to assessing the prognostic value of the autophagy-related lncRNAs, survival time and survival status data were merged with autophagy-related lncRNA expression data. The study excluded repeated samples and survival times of patients more than 30 days. Because the study data were obtained from the TCGA database, no ethics committee approval or patient consent was required.

### Identification of Autophagy-Related lncRNAs

Autophagy-related genes (ATGs) were downloaded from the Human Autophagy Database (http://www.autophagy.lu/) (12). Pearson’s correlational coefficients were calculated to assess associations between lncRNA expression and ATGs. lncRNAs with correlational coefficients > 0.3 and *p* values < 0.001 were deemed to be putatively autophagy-related lncRNAs.

### Autophagy-Related lncRNA Prognostic Model Construction

Univariate Cox regression was used to assess associations between autophagy-related lncRNA expression and overall survival using the “survival” software package in R (cran.r-project.org/web/packages/survival/index.html). A significant difference was defined as *p* < 0.05. LASSO regression was performed based on the results of univariate Cox regression analysis using the survival software and the “glmnet” software package (https://cran.rproject.org/web/packages/glmnet/index.html). After the identification of prognosis-related lncRNAs *via* LASSO regression analysis, multivariate Cox regression analysis with stepwise selection to identify optimal prognostic autophagy related lncRNAs according to Akaike information criterion (AIC) value (15). We then calculated a risk score for each patient (16). HNSCC patients were divided into high-risk (*n* = 77) and low-risk (*n* = 77) groups based on the median risk score. To investigate relationships between prognosis and expression levels of the autophagy-related lncRNAs identified, Kaplan-Meier survival curves were then plotted for three key lncRNAs. Based on average expression levels of candidate lncRNAs, HNSCC patients were divided into high and low expression groups.

### Evaluation of Accuracy of Our Model

Kaplan-Meier survival curves were plotted to compare the difference in overall survival between the low-risk and high-risk groups using the survival package (17). Using the survival and time ROC (cran.r-project.org/web/packages/timeROC/index.html) software packages, 3-year and 5-year ROC curves were generated, and areas under the curves (AUCs) and concordance indexes were calculated to investigate the predictive capacity of the prognostic model (18). AUC values range from 0.5 to 1.0. AUC: 0.5 means without any predictive power; AUC: 0.5–0.7 represents low accuracy; AUC: 0.7–0.9 means medium accuracy; AUC > 0.9 represents high accuracy. Concordance index values range from 0.5 to 1.0. Univariate and multivariate Cox regression analyses were used to determine whether risk score was independent of other clinical characteristics (age, sex, grade, stage) for the prediction of HNSCC prognosis. HR>1 and p-value<0.05 represent poor prognostic factors. HR<1 and p-value<0.05 represent favorable prognostic factors.

### Validation of the Prognostic Value of Three Autophagy-Related lncRNAs

The Gene Expression Profiling Interactive Analysis database (GEPIA; http://gepia.cancerpku.cn/detail.php) is a web server tool based on TCGA and GTEx data that can be used to conduct patient survival analysis, differential expression analysis, and correlational analysis, among other things (19). The Head and Neck Cancer database (http://hncdb.canceromics.org/) is a comprehensive source that includes head and neck cancer-related genes and an expression analysis platform. Lnc2Cancer (http://www.bio-bigdata.net/lnc2cancer/) is a database that provides comprehensive experimentally supported associations between lncRNA and human cancer (20). The Online Consensus Survival for Head and Neck Squamous Cell Carcinoma database (OShnscc, http://bioinfo.henu.edu.cn/HNSC/HNSCList.jsp) is an online prognostic biomarker analysis tool for head and neck squamous cell carcinoma (21). UALCAN (http://ualcan.path.uab.edu/) is a user-friendly web resource for analyzing cancer OMICS data that allows the user to analyze associations between gene expression and patient survival (22). In the present study the GEPIA, Head and Neck Cancer database, Lnc2cancer, OShnscc, and UALCAN resources were used to investigate the prognostic value of the lncRNAs identified. *P* < 0.05 was considered statistically significant.

### Co-expression and Function Enrichment Analysis

To investigate the function of three autophagy-related lncRNAs and the potential mechanisms involved in the effects of these lncRNAs on HNSCC prognosis, associations between the expression levels of the lncRNAs and protein coding genes (PCGs) were assessed using R software. Pearson’s correlational coefficient > 0.4 and *p* < 0.001 were considered to indicate significant correlations. A visual co-expression network reflecting the three autophagy-related lncRNAs and PCG co-expression was generated *via* Cytoscape software (version 3.7.2) (18). To assess associations between the three lncRNAs, PCGs, and risk status the “ggalluvial” component of the R package was used to visualize interaction modules between them (23). Lastly, gene ontology and Kyoto Encyclopedia of Genes and Genomes (KEGG) enrichment analyses were performed on three lncRNA-related PCGs using Metascape and the R package (24). *P* < 0.05 was considered significant in gene ontology and KEGG pathway analyses. Enriched gene ontology terms and KEGG pathways were defined as the potential functional terms of the three lncRNAs.

### Associations Between Three lncRNAs and Autophagy-Related Pathway

Associations between three lncRNAs and ATGs were investigated by calculating Pearson’s correlational coefficients. Sankey plots were then constructed to visualize association networks between the three lncRNAs, ATGs, and risk status. Gene ontology and KEGG pathway analyses of the three lncRNA-related ATGs were conducted using Metascape (http://metascape.org/gp/index.html) (24). *P* < 0.05 was considered statistically significant.

### Confirmation of the Expression of Three lncRNAs and Autophagy Makers and Evaluation of Their Correlation

The mRNA expression level of three lncRNAs and autophagy markers ATG12, BECN1, and MAP1LC3B were examined through analysis DiffExp module of TIMER database (https://cistrome.shinyapps.io/timer/) and Oncomine database (http://www.oncomine.org) (25, 26). The protein expression level of these makers were compared between HNSCC and normal tissues using HPA database (https://www.proteinatlas.org/) (27). Association analyses between three lncRNAs and autophagy related genes was also performed by GEPIA database and correlation module of TIMER database (19, 25).

### Statistical Analysis

Data were processed using the PERL language (strawberry-perl-5.32.0.1-64bit.msi, http://www.perl.org). All statistical analyses were conducted using R software (version 3.6.3, https://www.r-project.org/). *P* < 0.05 was considered statistically significant.

## Results

### Identification of Autophagy-Related lncRNAs

The analysis procedure of the current study is shown in **Supplementary Material 1**. A total of 14,142 lncRNAs were identified by analyzing RNA sequence data from HNSCC patient tissue samples in the TCGA database, and 232 ATGs were extracted from the Human Autophagy Database (**Supplementary Material 2**). From the initial dataset, 1,153 autophagy-related lncRNAs were selected by calculating Pearson’s correlational coefficients between the lncRNAs and the ATGs (**Supplementary Material 3**).

### Establishment of a Three-Autophagy-Related lncRNA Prognostic Signature

**Figure 1:**
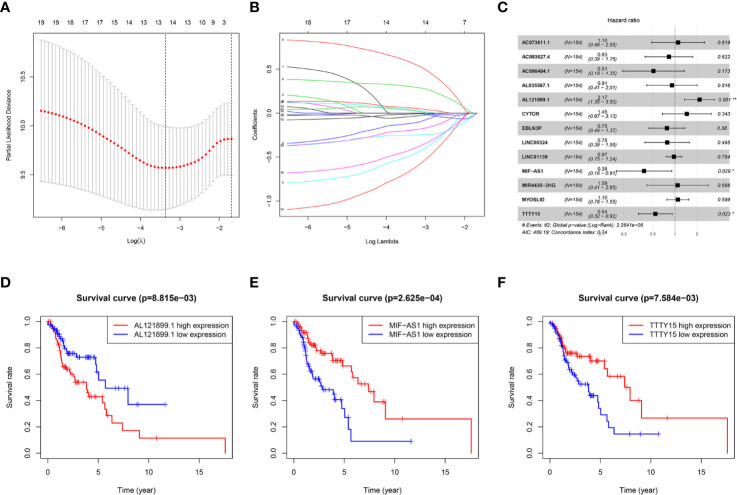
Identification of a three-autophagy-related lncRNA prognostic signature. **(A)** Partial likelihood deviance was plotted versus log(λ). The vertical dotted line indicates the lambda value with the minimum error and the largest lambda value, where the deviance is within one standard error of the minimum. **(B)** LASSO coefficient profiles of genes associated with overall survival of head and neck squamous cell carcinoma. **(C)** Forest plot depicting associations between lncRNAs and risk value determined *via* multivariate Cox regression analysis. *p < 0.05, **p < 0.01. **(D–F)** Kaplan–Meier survival curves derived from three autophagy-related lncRNAs and HNSCC patients. These lncRNAs were considered to constitute a prognostic signature for HNSCC. AL121899.1 was identified as a negative prognostic factor, and TTTY15 and MIF-AS1 were identified as positive prognostic factors. **(D)** AL121899, **(E)** MIF-AS, **(F)** TTTY15.

In univariate Cox regression analysis of 1,153 autophagy-related lncRNAs, 19 autophagy-related lncRNAs were significantly associated with HNSCC prognosis (*p* < 0.05, **Table 1**). LASSO regression analysis was conducted to assess the correlations between these 19 lncRNAs and survival. That analysis indicated that 13 lncRNAs had prognostic value (**Figures 1A, B**). In multivariate Cox regression analysis three autophagy-related lncRNAs were significantly correlated with HNSCC prognosis; AL121899.1, TTTY15, and MIF-AS1 (**Figure 1C**). AL121899.1 was considered a risk factor with a hazard ratio (HR) > 1, and TTTY15 and MIF-AS1 were considered protective factors with hazard ratios < 1. In Kaplan-Meier survival curve analysis of the three autophagy-related lncRNAs AL121899.1 overexpression was correlated with a poor prognosis, and high expressions of TTTY15 and MIF-AS were associated with a good prognosis (**Figures 1D–F**).

**Table 1:** Prognosis-related autophagy lncRNAs by univariate Cox regression analysis.

lncRNA	KM	B	SE	HR	Low	Upper	p-value
AC073611.1	0.0032	0.5092	0.1812	1.6639	1.1664	2.3736	0.005
MIR4435-2HG	0.0008	0.3496	0.1049	1.4185	1.1548	1.7423	0.0009
AL121899.1	0.0088	0.3302	0.0776	1.3912	1.195	1.6196	0
CYTOR	0.0082	0.1949	0.052	1.2151	1.0974	1.3455	0.0002
MYOSLID	0.0082	0.0626	0.0192	1.0646	1.0253	1.1055	0.0011
LINC01139	0.0071	-0.1196	0.045	0.8872	0.8123	0.9691	0.0079
EBLN3P	0.0026	-0.1721	0.048	0.8419	0.7663	0.925	0.0003
MIR9-3HG	0.0041	-0.1789	0.062	0.8362	0.7405	0.9442	0.0039
TTTY15	0.0076	-0.4966	0.1909	0.6086	0.4187	0.8848	0.0093
LINC00324	0.0004	-0.4996	0.1848	0.6068	0.4224	0.8717	0.0069
AL035587.1	0.0001	-0.535	0.2034	0.5857	0.3931	0.8725	0.0085
AC108010.1	0.0088	-0.5403	0.2008	0.5826	0.393	0.8635	0.0071
AC091057.1	0.0009	-0.7382	0.261	0.478	0.2866	0.7972	0.0047
DTX2P1-UPK3BP1-PMS2P11	0.0002	-0.7479	0.2691	0.4733	0.2794	0.8021	0.0054
AC008763.1	0.0008	-0.8182	0.3163	0.4412	0.2374	0.8201	0.0097
MIF-AS1	0.0003	-0.8733	0.3209	0.4176	0.2226	0.7832	0.0065
ZNF436-AS1	0.0033	-0.879	0.324	0.4152	0.22	0.7836	0.0067
AC093627.4	0.0081	-0.8912	0.3316	0.4101	0.2141	0.7856	0.0072
AC098484.1	0.0048	-0.9006	0.2977	0.4063	0.2267	0.7282	0.0025

HR, Hazard ratio; lncRNA, long noncoding RNA.

### Evaluation of the Prognostic Model

**Figure 2:**
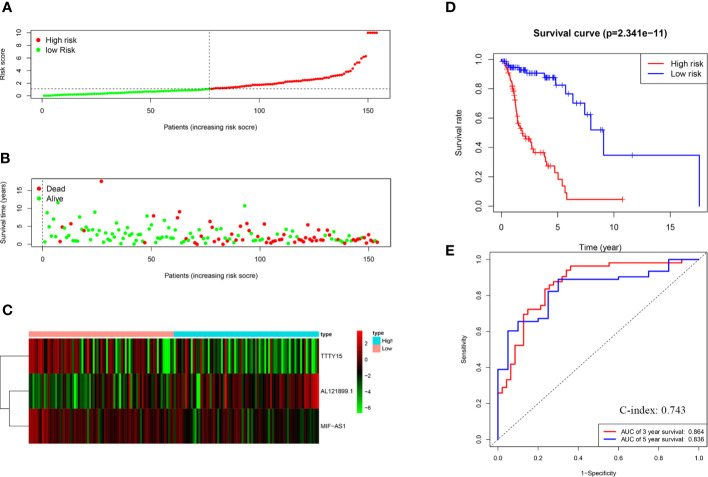
Construction and evaluation of the risk score model. **(A)** Survival rate was significantly associated with risk score. **(B)** Scatterplot of patient survival. **(C)** Expression heatmap of three autophagy-related lncRNAs. **(D)** Kaplan-Meier survival curves of the high-risk group and the low-risk group. **(E)** ROC curve based on the risk score model. AUC: 0.5 means without any predictive power; AUC: 0.5–0.7 represents low accuracy; AUC: 0.7–0.9 means medium accuracy; AUC > 0.9 represents high accuracy.

**Figure 3:**
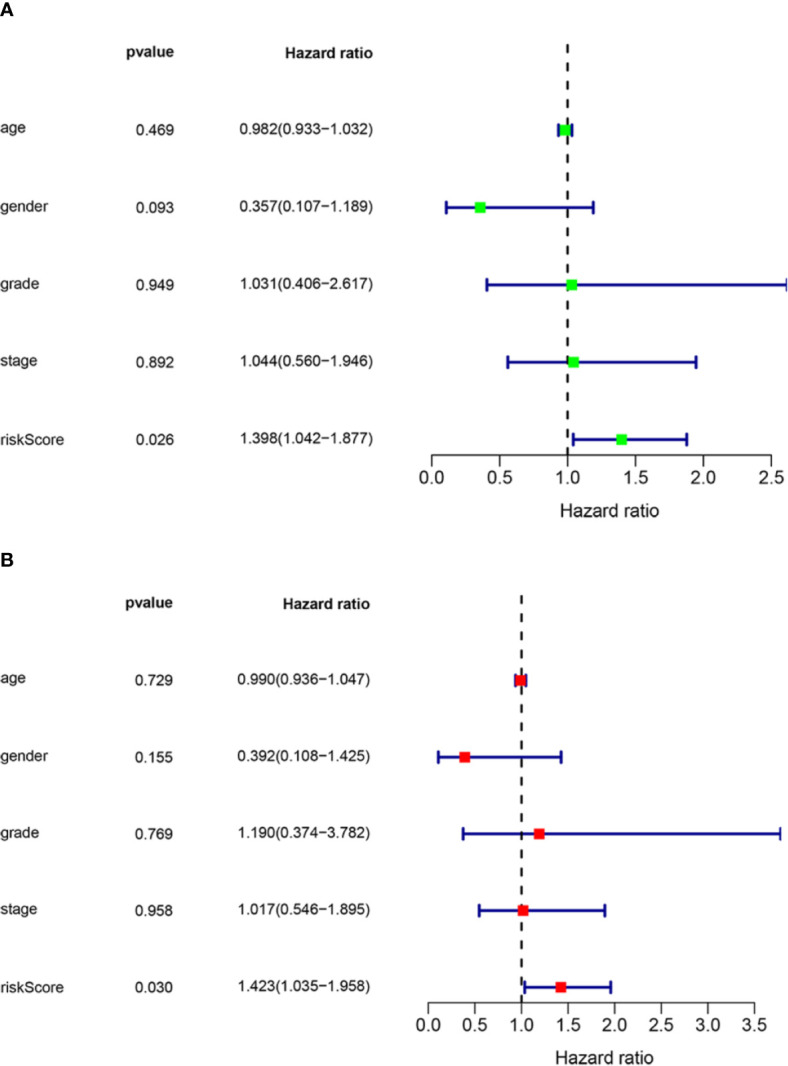
Assessment of the independence of the HNSCC prognostic model. **(A)** Univariate analysis and **(B)** multivariate Cox regression analysis confirmed the independence of the prognostic model. HNSCC, head and neck squamous cell carcinoma. HR>1 and p-value<0.05 represent poor prognostic factors. HR<1 and p-value <0.05 represent favorable prognostic factors.

Risk scores were calculated for each HNSCC patient, and the patients were divided into high-risk (*n* = 77) and low-risk (*n* = 77) groups based on the median risk score. HNSCC patients were ranked based on risk scores, and survival rate was significantly associated with risk score (**Figure 2A**). The survival time of patients with higher risk scores was lower than that of patients with low risk scores (**Figure 2B**). Heat mapping indicated that three autophagy-related lncRNAs were significantly differentially expressed in the high-risk group and the low-risk group (**Figure 2C**). AL121899.1 expression was higher in high-risk patients than in low-risk patients, and TTTY15 and MIF-AS expression were lower in high-risk patients than in low-risk patients. In Kaplan-Meier survival analysis patients with low risk scores survived significantly longer than those with high risk scores (**Figure 2D**). To assess the predictive value of the prognostic model, 3-year and 5-year ROC curves were plotted and the concordance index was calculated. The AUC values were 0.864 (3-year ROC) and 0.836 (5-year ROC), and the concordance index was 0.743 (**Figure 2E**). This result suggested that our model had a medium accuracy to evaluate the prognosis of head and neck squamous cell carcinoma patients. Univariate and multivariate Cox regression analyses demonstrated that risk score was an independent predictor of a poor prognosis in HNSCC patients (**Figures 3A, B**).

### Three Autophagy-Related lncRNAs Have Prognostic Value in HNSCC

**Figure 4:**
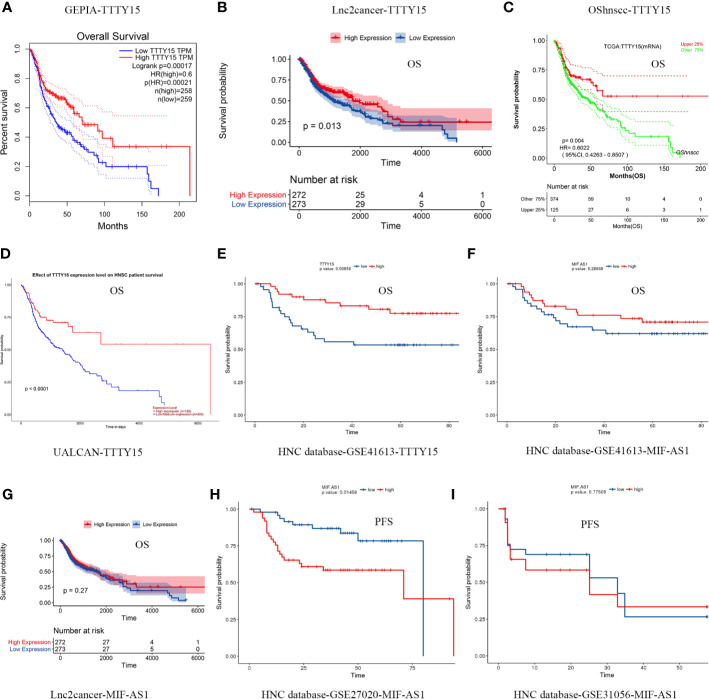
Confirmation of the prognostic value of the three autophagy-related lncRNAs. **(A–E)** Five databases were used to examine the prognostic value of TTTY15. **(A)** GEPIA database, **(B)** Lnc2cancer 3.0 online database, **(C)** OShnscc database, **(D)** UALCAN database, **(E)** HNC database. **(F–I)** Analyses of associations between MIF-AS1 and overall survival and progression-free survival based on different databases. **(F)** Association between MIF-AS1 and overall survival based on the GSE41613 database. **(G)** Association between MIF-AS1 and overall survival based on the lnc2cancer 3.0 online database. **(H)** Association between MIF-AS1 and progression-free survival based on the GSE27020 database. **(I)** Association between MIF-AS1 and progression-free survival based on the GSE31056 database. PFS, progression-free survival; OS, overall survival.

Multiple databases were used to confirm the prognostic value of the aforementioned three autophagy-related lncRNAs. TTTY15 overexpression had a protective effect on overall survival in different databases, including the GEPIA, Lnc2cancer3.0, Head and Neck Cancer database, UALCAN, and OShnscc (**Figures 4A–E**, **Table 2**). These observations are fully consistent with the results of the current study. In GSE27020 analysis, high MIF-AS1 expression was associated with progression-free survival in HNSCC patients, but there were no significant correlations between MIF-AS1 overexpression and progression-free survival or overall survival in analyses of the GES31056, GSE41613, and Lnc2cancer3.0 databases (**Figures 4F–I**, **Table 2**). AL121899.1 was not found in the five aforementioned databases.

**Table 2:** Confirmation of prognostic value of three autophagy related IncRNAs.

IncRNA	Database	Datasets	Endpoint	p	HR	Prognostic
MIF-AS1	HNC database	GSE41613	OS	0.28668		
MIF-AS1	HNC database	GSE27020	PFS	0.01456		Poor
MIF-AS1	HNC database	GSE31056	PFS	0.77508		
MIF-AS1	Lnc2cancer3.0		OS	0.27		
TTTY15	GEPIA	TCGA and GTEx	OS	0.00021	0.6	Good
TTTY15	HNC database	GSE41613	OS	0.00858		Good
TTTY15	HNC database	GSE27020	PFS	0.0802		
TTTY15	HNC database	GSE31056	PFS	0.99911		
TTTY15	Lnc2cancer3.0		OS	0.013		Good
TTTY15	OShnscc	TCGA	OS	0.0004	0.6022	Good
TTTY15	UALCAN	TCGA	OS	<0.0001		Good

Five databases were used to validate the correlation between the three autophagy related lncRNAs and prognosis in HNSCC. HR, Hazara Ratio, PFS, Progression Free Survival; OS, Overall Survival.

### Potential Functions of the Three Autophagy-Related lncRNAs

**Figure 5:**
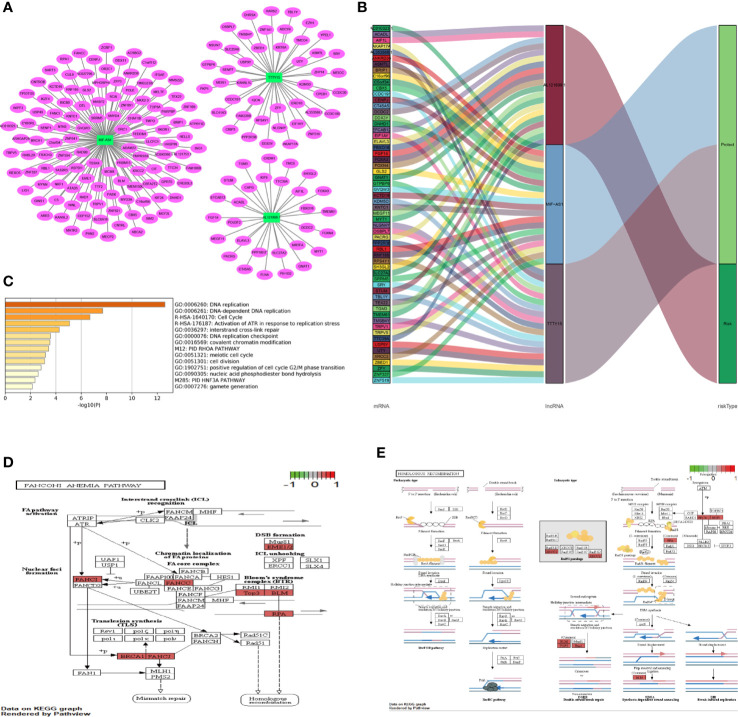
Co-expression network and function enrichment analysis. **(A)** Construction of a co-expression network between three prognosis-related autophagy lncRNAs and PCGs. The green rectangles represent three prognosis-related lncRNAs, and the red ovals represent PCGs. **(B)** Sankey plots were generated to visualize association networks between the three lncRNAs, PCGs, and risk status. Due to the size of the image, the module on the left only shows the first 20 PCGs of each lncRNA. **(C)** Gene enrichment analysis of 189 PCGs. **(D, E)** KEGG pathway analysis of 189 PCGs. The red highlighted part represents the PCGs in the current study. **(D)** Eight PCGs involved in the Fanconi anemia pathway. **(E)** Seven PCGs associated with homologous recombination. PCG, protein coding gene; lncRNA, long non-coding RNA.

A co-expression network including the three autophagy-related lncRNAs and PCGs was constructed to predict the function of the three lncRNAs and investigate the potential mechanisms that they were involved in that affected HNSCC prognosis. The three lncRNAs interacted with 189 PCGs (**Figure 5A**). Associations between the three lncRNAs, the PCGs, and different risk types were then evaluated. TTTY15 and MIF-AS1 and their related PCGs were putative protective factors. AL121899.1 and its related PCGs were putative risk factors (**Figure 5B**). To predict the functions of the three lncRNAs, 189 PCGs were assessed *via* gene enrichment and KEGG pathway analysis. The enriched gene ontology terms were mainly involved in DNA replication, the cell cycle, DNA-dependent DNA replication, activation of ATRs in response to replication stress, cell division, the PID HNF3A pathway, and so on (**Figure 5C**). In KEGG pathway analysis 189 PCGs were mainly involved in homologous recombination and the Fanconi anemia pathway (**Figures 5D, E**). These identified gene ontology terms or KEGG pathways were considered indicative of the potential functions of the three lncRNAs.

### The lncRNAs Were Associated With the Autophagy-Related Pathway

**Figure 6:**
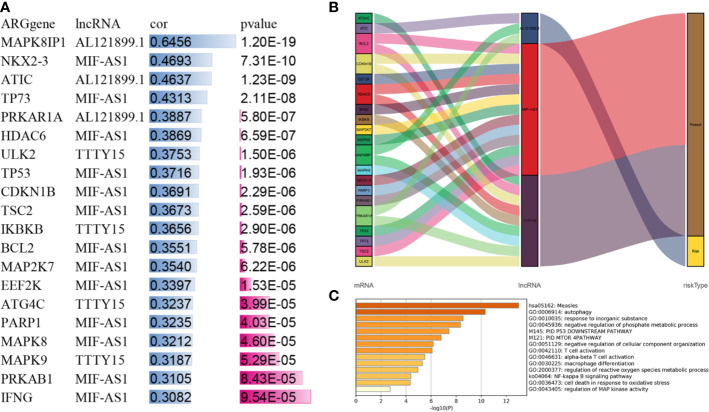
Associations between the three lncRNAs and the autophagy-related pathway. **(A)** Three lncRNAs interacted with 20 ATGs. **(B)** Sankey plot depicting degrees of connection between 20 ATGs and 3 autophagy-related lncRNAs (risk/protective). **(C)** Metascape analysis of the gene ontology terms and pathways of three lncRNA-related ATGs. ATG, autophagy-related gene.

Three lncRNAs were significantly associated with 20 autophagy-related genes (**Figure 6A**). Sankey plots indicated that AL121899.1 and related ATGs were associated with an unfavorable prognosis, whereas TTTY15 and MIF-AS1 and their related ATGs were associated with a favorable prognosis (**Figure 6B**). Gene ontology and KEGG pathway analysis indicated that the ATGs were mainly involved in measles, autophagy, the P53 pathway, the PID MTOR pathway, the nuclear factor kappa B signaling pathway, regulation of mitogen-activated protein kinase activity, and the immune related pathway (**Figure 6C**).

### Three lncRNAs Are Correlated With Autophagy Markers

**Figure 7:**
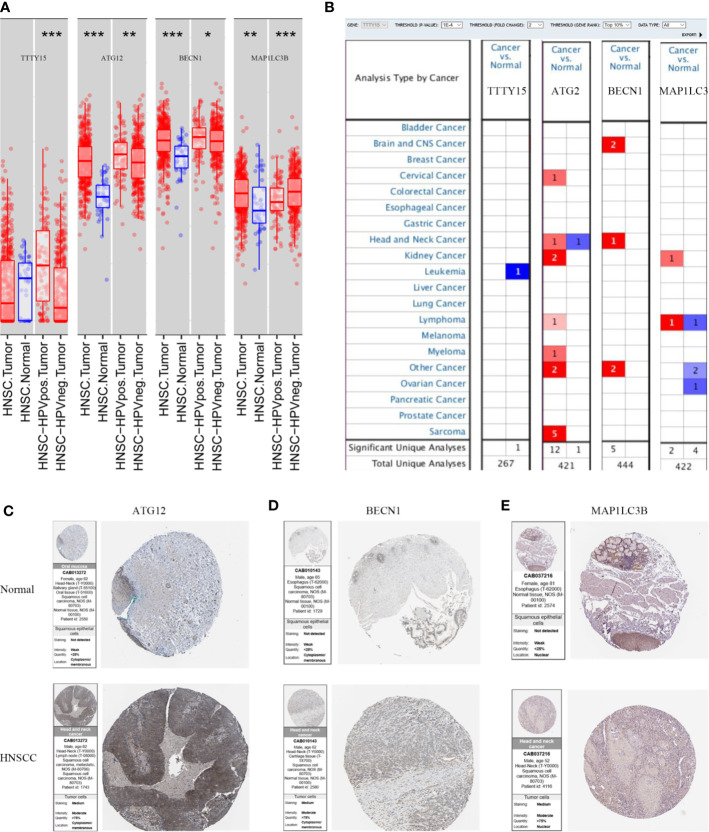
The expression of three lncRNAs and autophagy related genes at mRNA and protein level. **(A)**TIMER analyze the expression of three lncRNAs and ATGs at the RNA level. **(B)** Different expression of three lncRNAs and ATGs in HNSCC and normal sample from Oncomine database. Red indicates up-regulated, blue represents down-regulated. **(C–E)** The protein expression levels of ATG12, BECN1, and MAP1LC3B for HNSCC tissues and normal tissues from HPA database. **(C)** ATG12, **(D)** BECN1, **(E)** MAP1LC3B. Autophagy Related Genes=ATGs. BECN1=Beclin1. Statistical significance was defined by a p value:*** means 0 ≤p-value < 0.001, ** represents 0.001≤p-value < 0.01, * represents 0.01≤p-value < 0.05,. means 0.05 ≤p-value <0.1.

**Figure 8:**
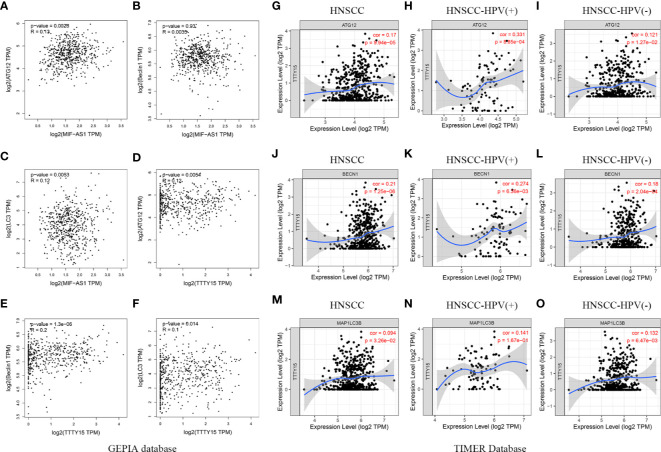
Correlation between three lncRNAs and autophagy makers. **(A–C)** The association between MIF-AS1 and three autophagy makers was evaluated using GEPIA database. **(A)** ATG12, **(B)** Beclin1, **(C)** LC3. **(D–F)** Association between TTTY15 and three autophagy makers was assessed by GEPIA analysis. **(D)** ATG12, **(E)** Beclin1, **(F)** LC3. **(G–I)** The correlation between TTTY15 and ATG12 in different HNSCC patients was analyzed using TIMER database. **(G)** HNSCC patients, **(H)** HPV (+) HNSCC patients, **(I)** HPV (-) HNSCC patients. **(J–L)** The association between TTTY15 and BECN1 was detected in different HNSCC patients. **(J)** HNSCC patients, **(K)** HPV (+) HNSCC patients, **(L)** HPV (-) HNSCC patients. **(M–O)** Evaluation the correlation between TTTY15 and MAP1LC3B based on TIMER database. **(M)** HNSCC patients, **(N)** HPV (+) HNSCC patients, **(O)** HPV (-) HNSCC patients.

We verified the expression levels of the three lncRNAs and autophagy markers ATG12, BECN1, and MAP1LC3B through analysis TIMER, Oncomine, and HPA database. As shown in **Figure 7A**, TTTY15 was highly expressed in HPV (+) and HPV (-) patients, while TTTY15 expression had no difference in HNSCC patients compared with normal tissues. ATG12, BECN1, and MAP1LC3B were increased in HNSCC tissue, HPV (+), and HPV (-) patients compared with normal tissues. ATG12 and BECN1 were upregulated in HNSCC tissue compared with normal tissues based on Oncomine database. There was no significant difference discovered in HNSCC patients with an expression level of TTTY15 and MAP1LC3B (**Figure 7B**). The protein level of three autophagy makers were highly expressed in HNSCC tissues (**Figures 7C–E**). The results of GEPIA analysis showed that MIF-AS1 was positively correlated with ATG12 and LC3, and TTTY15 was positively related to three autophagy markers (ATG12, Bclin1, LC3) (**Figures 8A–F**). TTTY15 was positively correlated with ATG12 and BECN1 in different HNSCC patients, including HNSCC patients, HPV (+) HNSCC patients, and HPV (-) HNSCC patients (**Figures 8G–L**). The positive association between TTTY15 and MAP1LC3B was detected in HNSCC samples and HPV (-) HNSCC samples based on correlation module of TIMER analysis (**Figures 8M–O**). MIF-AS1 was not found in TIMER database.

## Discussion

More than 500,000 new cases of head and neck cancer are diagnosed every year. HNSCC is the most common pathological type of head and neck cancer, and it is typically highly aggressive and has a poor prognosis (28). TNM staging is not sensitive enough to accurately predict HNSCC prognosis, thus a new method for formulating HNSCC prognosis is required (29). Due to the increased amount of gene expression profile data that has become available in recent years in conjunction with developments in bioinformatics, increased attention has been given to investigating associations between the alteration of RNA, non-coding RNA, and HNSCC prognosis (30, 31). Autophagy plays complex roles in HNSCC. Further investigation to elucidate the roles and clarify the mechanisms of autophagy in HNSCC is imperative (32–34). The analyses conducted in the current study suggest that autophagy-related lncRNAs may provide novel insights into the prediction of HNSCC prognoses. Little is known about the prognostic value of autophagy-related lncRNAs in HNSCC.

AL121899.1 has previously been identified as a tumor suppressor gene in esophageal squamous cell carcinoma *via* differential co-expression analysis (35). MIF-AS1 is reportedly significantly upregulated in ovarian cancer, and that upregulation can promote the proliferative, migratory, and invasive capacities of ovarian cancer cells. Higher levels of MIF-AS1 predicted poorer prognoses in ovarian cancer patients (36). In breast cancer and gastric cancer, lncRNA MIF-AS1 evidently promotes cell proliferation (37, 38). In a previous study, TTTY15 expression was downregulated in patients with non-small cell lung cancer, and low expression of TTTY15 was associated with tumor stage and poorer prognosis (39). TTTY15 was also reportedly upregulated in most prostate cancer patients and could promote prostate cancer progression by sponging let-7 (40). However, there are no reports on associations between HNSCC prognosis and AL121899.1, MIF-AS1, or TTTY15. In the present study MIF-AS1 and TTTY15 were identified as favorable prognostic biomarkers, and AL121899.1 was identified as an unfavorable prognostic biomarker. These findings were consistent with our understanding that the three lncRNAs were associated with the prognosis of cancer. Moreover, previous study suggested that TTTY15 was associated with HPV infection, and which was upregulated in HPV-positive patients (41). Previous studies reported that Beclin 1 (BECN1) and LC3 are unique autophagy-related protein (42). ATG12, ATG5, and MAP1LC3/LC3 have been reported to mediate the formation of the autophagosome (43). The prognosis for HPV positive patients is significantly longer than those with HPV negative carcinomas in cases of similar treatment (44). We discovered that TTTY15 had upregulation both in HPV (+) and HPV (-) patients, and which was positively correlated with autophagy makers in different HPV status. Positive correlation between MIF-AS1 and autophagy makers (ATG12 and LC3) was examined in HNSCC patients. Taken together, three autophagy-related lncRNAs may regulate the prognosis of HNSCC patients with different HPV status, which provides new insights for evaluating the prognosis of HNSCC.

In previous studies, moderate autophagy reportedly promoted homologous recombination (45, 46). Autophagy can regulate proteins in the homologous recombination repair pathway. DNA damage occurs, the activation of ATG promotes autophagy and activates the downstream STAT3-BRCA1 pathway, allowing BRCA1 protein to participate in chain scission during homologous recombination repair  (46). Inhibition of the classic PI3K/Akt/mTOR autophagy pathway can significantly reduce the level of autophagy in prostate cancer cells, and suppress homologous recombination repair (47). Fanconi anemia pathway genes play an important role in tumor suppression and repair of damage to nuclear DNA. Defects in selective autophagy have profound effects on mutations in Fanconi anemia pathway genes (48). Fanconi anemia genes have also been implicated in breast, ovarian, and pancreatic cancer (49). The three Fanconi anemia genes FANCC, FANCF, and FANCL were identified as candidate autophagy factors *via* whole genome screening (50). It has been reported that autophagy can be regulated by different pathways, such as those involving AKT, MTOR, mitogen-activated protein kinase, AMP-activated protein kinase, PIK3C3, and the proteins encoded by ATG genes (51). Nuclear factor kappa B contributes to repression of tumor necrosis factor alpha-induced autophagy (52). In a previous study a prognostic model was constructed based on autophagy-related lncRNA, and bladder urothelial carcinoma (BLCA) patients were divided into high-risk and low-risk groups. The PPAR signaling pathway, mitogen-activated protein kinase signaling pathway, P53 signaling pathway, and mTOR signaling pathway were associated with high risk in BLCA patients, whereas immune-related pathways were significantly enriched in a low-risk group (14). Co-expression network and pathway analysis in the current study indicated that functionally the three lncRNAs identified were mainly involved in homologous recombination, the Fanconi anemia pathway, the autophagy-related pathway, and the immune-related pathway. These results are consistent with previous studies indicating that autophagy is regulated by the aforementioned pathways, which may play critical roles in regulating HNSCC prognosis.

## Conclusion

Three autophagy-related lncRNAs have prognostic value with respect to HNSCC, and their related pathways may be involved in regulating HNSCC prognosis. Further investigations and experiments such as quantitative real-time PCR, western blotting, and clinical data analyses are required to validate the results of the present study. We will perform further experiments to investigate the molecular mechanisms involved in the effects of autophagy-related lncRNAs on HNSCC prognosis, and their effects on clinical transformation in HNSCC patients.

## Data Availability Statement

Publicly available datasets were analyzed in this study. RNA-seq and corresponding clinical data were available from the TCGA database (https://portal.gdc.cancer.gov/).

## Author Contributions

YG conceived and designed the study and wrote the manuscript. PY participated in data analysis. ZW reviewed the manuscript. KX performed language editing. WK and HL prepared the figures and tables. All authors contributed to the article and approved the submitted version.

## Funding

This work was supported by the Shaanxi Provincial Natural Science Foundation (2017JQ8057).

## Conflict of Interest

The authors declare that the research was conducted in the absence of any commercial or financial relationships that could be construed as a potential conflict of interest.
