# Supplementary material for: Identification of Three Autophagy-Related Long Non-Coding RNAs as a Novel Head and Neck Squamous Cell Carcinoma Prognostic Signature
Source: Front Oncol. 2021 Jan 26;10:603864. doi: 10.3389/fonc.2020.603864 (PMC7871905; doi:10.3389/fonc.2020.603864)

Supplementary material 1

Analysis of flowchart illustrates the exploration procedure for the HNSCC prognosis related autophagy lncRNAs

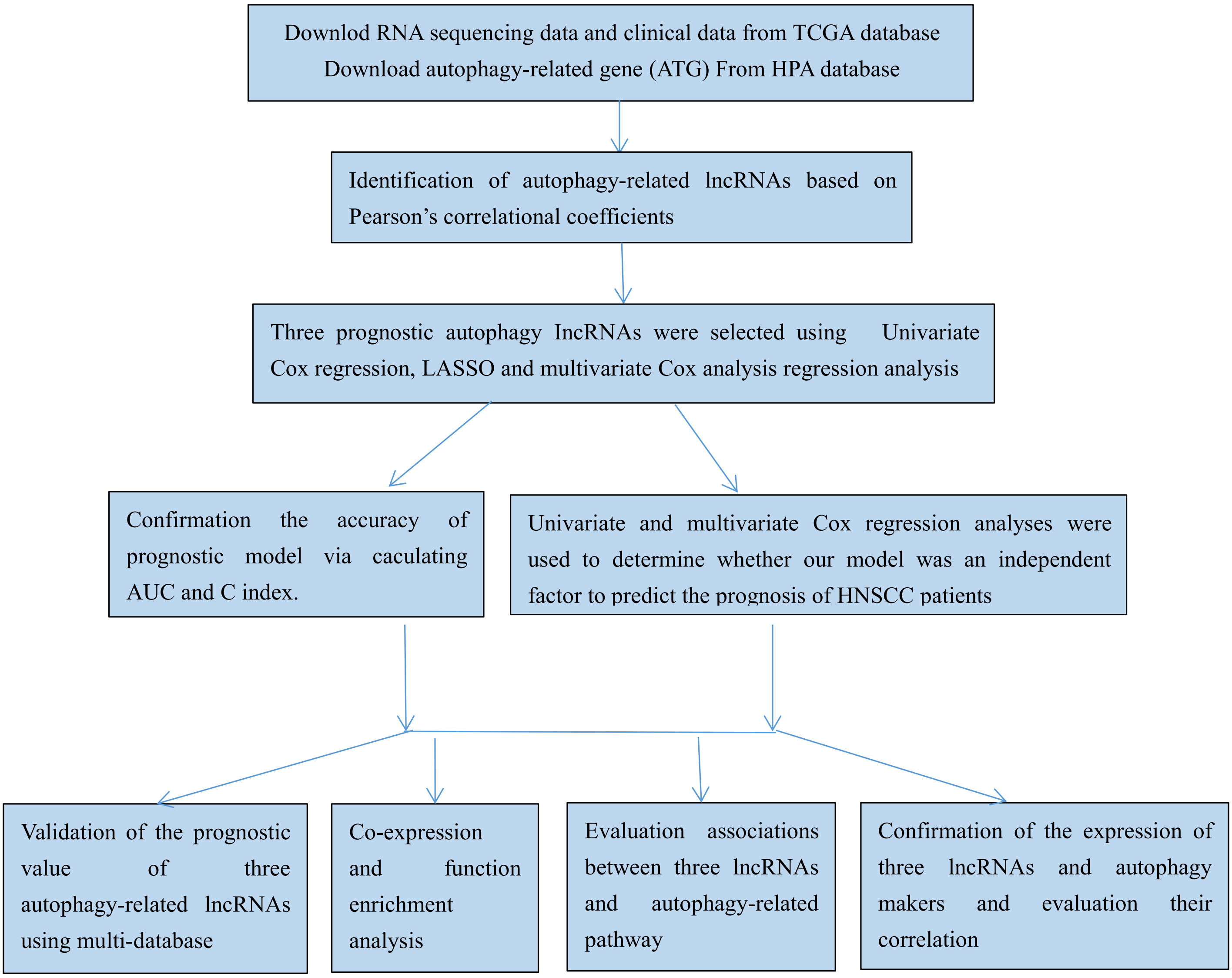

Supplement: Supplementary file 1 [file DataSheet_1.pdf]
