# Supplementary material for: Identification of Three Autophagy-Related Long Non-Coding RNAs as a Novel Head and Neck Squamous Cell Carcinoma Prognostic Signature
Source: Front Oncol. 2021 Jan 26;10:603864. doi: 10.3389/fonc.2020.603864 (PMC7871905; doi:10.3389/fonc.2020.603864)
Supplement: Supplementary file 2 [file DataSheet_2.docx]

**Supplementary material 2**

**232 autophagy-related genes were obtained from HADb database**

| **Gene Id** | **Symbol** | **Name** |
| --- | --- | --- |
| [55626](http://www.autophagy.lu/genes/55626) | AMBRA1 | autophagy/beclin-1 regulator 1 |
| [8542](http://www.autophagy.lu/genes/8542) | APOL1 | apolipoprotein L, 1 |
| [405](http://www.autophagy.lu/genes/405) | ARNT | aryl hydrocarbon receptor nuclear translocator |
| [410](http://www.autophagy.lu/genes/410) | ARSA | arylsulfatase A |
| [411](http://www.autophagy.lu/genes/411) | ARSB | arylsulfatase B |
| [468](http://www.autophagy.lu/genes/468) | ATF4 | activating transcription factor 4 (tax-responsive enhancer element B67) |
| [22926](http://www.autophagy.lu/genes/22926) | ATF6 | activating transcription factor 6 |
| [83734](http://www.autophagy.lu/genes/83734) | ATG10 | ATG10 autophagy related 10 homolog (S. cerevisiae) |
| [9140](http://www.autophagy.lu/genes/9140) | ATG12 | ATG12 autophagy related 12 homolog (S. cerevisiae) |
| [55054](http://www.autophagy.lu/genes/55054) | ATG16L1 | ATG16 autophagy related 16-like 1 (S. cerevisiae) |
| [89849](http://www.autophagy.lu/genes/89849) | ATG16L2 | ATG16 autophagy related 16-like 2 (S. cerevisiae) |
| [23130](http://www.autophagy.lu/genes/23130) | ATG2A | ATG2 autophagy related 2 homolog A (S. cerevisiae) |
| [55102](http://www.autophagy.lu/genes/55102) | ATG2B | ATG2 autophagy related 2 homolog B (S. cerevisiae) |
| [64422](http://www.autophagy.lu/genes/64422) | ATG3 | ATG3 autophagy related 3 homolog (S. cerevisiae) |
| [115201](http://www.autophagy.lu/genes/115201) | ATG4A | ATG4 autophagy related 4 homolog A (S. cerevisiae) |
| [23192](http://www.autophagy.lu/genes/23192) | ATG4B | ATG4 autophagy related 4 homolog B (S. cerevisiae) |
| [84938](http://www.autophagy.lu/genes/84938) | ATG4C | ATG4 autophagy related 4 homolog C (S. cerevisiae) |
| [84971](http://www.autophagy.lu/genes/84971) | ATG4D | ATG4 autophagy related 4 homolog D (S. cerevisiae) |
| [9474](http://www.autophagy.lu/genes/9474) | ATG5 | ATG5 autophagy related 5 homolog (S. cerevisiae) |
| [10533](http://www.autophagy.lu/genes/10533) | ATG7 | ATG7 autophagy related 7 homolog (S. cerevisiae) |
| [79065](http://www.autophagy.lu/genes/79065) | ATG9A | ATG9 autophagy related 9 homolog A (S. cerevisiae) |
| [285973](http://www.autophagy.lu/genes/285973) | ATG9B | ATG9 autophagy related 9 homolog B (S. cerevisiae) |
| [471](http://www.autophagy.lu/genes/471) | ATIC | 5-aminoimidazole-4-carboxamide ribonucleotide formyltransferase/IMP cyclohydrolase |
| [573](http://www.autophagy.lu/genes/573) | BAG1 | BCL2-associated athanogene |
| [9531](http://www.autophagy.lu/genes/9531) | BAG3 | BCL2-associated athanogene 3 |
| [578](http://www.autophagy.lu/genes/578) | BAK1 | BCL2-antagonist/killer 1 |
| [581](http://www.autophagy.lu/genes/581) | BAX | BCL2-associated X protein |
| [596](http://www.autophagy.lu/genes/596) | BCL2 | B-cell CLL/lymphoma 2 |
| [598](http://www.autophagy.lu/genes/598) | BCL2L1 | BCL2-like 1 |
| [8678](http://www.autophagy.lu/genes/8678) | BECN1 | beclin 1, autophagy related |
| [637](http://www.autophagy.lu/genes/637) | BID | BH3 interacting domain death agonist |
| [332](http://www.autophagy.lu/genes/332) | BIRC5 | baculoviral IAP repeat-containing 5 |
| [57448](http://www.autophagy.lu/genes/57448) | BIRC6 | baculoviral IAP repeat-containing 6 |
| [662](http://www.autophagy.lu/genes/662) | BNIP1 | BCL2/adenovirus E1B 19kDa interacting protein 1 |
| [664](http://www.autophagy.lu/genes/664) | BNIP3 | BCL2/adenovirus E1B 19kDa interacting protein 3 |
| [665](http://www.autophagy.lu/genes/665) | BNIP3L | BCL2/adenovirus E1B 19kDa interacting protein 3-like |
| [60673](http://www.autophagy.lu/genes/60673) | C12orf44 | chromosome 12 open reading frame 44 |
| [23591](http://www.autophagy.lu/genes/23591) | C17orf88 | chromosome 17 open reading frame 88 |
| [10241](http://www.autophagy.lu/genes/10241) | CALCOCO2 | calcium binding and coiled-coil domain 2 |
| [10645](http://www.autophagy.lu/genes/10645) | CAMKK2 | calcium/calmodulin-dependent protein kinase kinase 2, beta |
| [821](http://www.autophagy.lu/genes/821) | CANX | calnexin |
| [823](http://www.autophagy.lu/genes/823) | CAPN1 | calpain 1, (mu/I) large subunit |
| [11132](http://www.autophagy.lu/genes/11132) | CAPN10 | calpain 10 |
| [824](http://www.autophagy.lu/genes/824) | CAPN2 | calpain 2, (m/II) large subunit |
| [826](http://www.autophagy.lu/genes/826) | CAPNS1 | calpain, small subunit 1 |
| [834](http://www.autophagy.lu/genes/834) | CASP1 | caspase 1, apoptosis-related cysteine peptidase (interleukin 1, beta, convertase) |
| [836](http://www.autophagy.lu/genes/836) | CASP3 | caspase 3, apoptosis-related cysteine peptidase |
| [837](http://www.autophagy.lu/genes/837) | CASP4 | caspase 4, apoptosis-related cysteine peptidase |
| [841](http://www.autophagy.lu/genes/841) | CASP8 | caspase 8, apoptosis-related cysteine peptidase |
| [6347](http://www.autophagy.lu/genes/6347) | CCL2 | chemokine (C-C motif) ligand 2 |
| [729230](http://www.autophagy.lu/genes/729230) | CCR2 | chemokine (C-C motif) receptor 2 |
| [4179](http://www.autophagy.lu/genes/4179) | CD46 | CD46 molecule, complement regulatory protein |
| [1026](http://www.autophagy.lu/genes/1026) | CDKN1A | cyclin-dependent kinase inhibitor 1A (p21, Cip1) |
| [1027](http://www.autophagy.lu/genes/1027) | CDKN1B | cyclin-dependent kinase inhibitor 1B (p27, Kip1) |
| [1029](http://www.autophagy.lu/genes/1029) | CDKN2A | cyclin-dependent kinase inhibitor 2A (melanoma, p16, inhibits CDK4) |
| [8837](http://www.autophagy.lu/genes/8837) | CFLAR | CASP8 and FADD-like apoptosis regulator |
| [25978](http://www.autophagy.lu/genes/25978) | CHMP2B | chromatin modifying protein 2B |
| [128866](http://www.autophagy.lu/genes/128866) | CHMP4B | chromatin modifying protein 4B |
| [1201](http://www.autophagy.lu/genes/1201) | CLN3 | ceroid-lipofuscinosis, neuronal 3 |
| [1508](http://www.autophagy.lu/genes/1508) | CTSB | cathepsin B |
| [1509](http://www.autophagy.lu/genes/1509) | CTSD | cathepsin D |
| [1514](http://www.autophagy.lu/genes/1514) | CTSL1 | cathepsin L1 |
| [6376](http://www.autophagy.lu/genes/6376) | CX3CL1 | chemokine (C-X3-C motif) ligand 1 |
| [7852](http://www.autophagy.lu/genes/7852) | CXCR4 | chemokine (C-X-C motif) receptor 4 |
| [1612](http://www.autophagy.lu/genes/1612) | DAPK1 | death-associated protein kinase 1 |
| [23604](http://www.autophagy.lu/genes/23604) | DAPK2 | death-associated protein kinase 2 |
| [1649](http://www.autophagy.lu/genes/1649) | DDIT3 | DNA-damage-inducible transcript 3 |
| [9077](http://www.autophagy.lu/genes/9077) | DIRAS3 | DIRAS family, GTP-binding RAS-like 3 |
| [10395](http://www.autophagy.lu/genes/10395) | DLC1 | deleted in liver cancer 1 |
| [3337](http://www.autophagy.lu/genes/3337) | DNAJB1 | DnaJ (Hsp40) homolog, subfamily B, member 1 |
| [4189](http://www.autophagy.lu/genes/4189) | DNAJB9 | DnaJ (Hsp40) homolog, subfamily B, member 9 |
| [55332](http://www.autophagy.lu/genes/55332) | DRAM1 | DNA-damage regulated autophagy modulator 1 |
| [9695](http://www.autophagy.lu/genes/9695) | EDEM1 | ER degradation enhancer, mannosidase alpha-like 1 |
| [1938](http://www.autophagy.lu/genes/1938) | EEF2 | eukaryotic translation elongation factor 2 |
| [29904](http://www.autophagy.lu/genes/29904) | EEF2K | eukaryotic elongation factor-2 kinase |
| [1956](http://www.autophagy.lu/genes/1956) | EGFR | epidermal growth factor receptor (erythroblastic leukemia viral (v-erb-b) oncogene homolog, avian) |
| [5610](http://www.autophagy.lu/genes/5610) | EIF2AK2 | eukaryotic translation initiation factor 2-alpha kinase 2 |
| [9451](http://www.autophagy.lu/genes/9451) | EIF2AK3 | eukaryotic translation initiation factor 2-alpha kinase 3 |
| [1965](http://www.autophagy.lu/genes/1965) | EIF2S1 | eukaryotic translation initiation factor 2, subunit 1 alpha, 35kDa |
| [1978](http://www.autophagy.lu/genes/1978) | EIF4EBP1 | eukaryotic translation initiation factor 4E binding protein 1 |
| [1981](http://www.autophagy.lu/genes/1981) | EIF4G1 | eukaryotic translation initiation factor 4 gamma, 1 |
| [2064](http://www.autophagy.lu/genes/2064) | ERBB2 | v-erb-b2 erythroblastic leukemia viral oncogene homolog 2, neuro/glioblastoma derived oncogene homolog (avian) |
| [2081](http://www.autophagy.lu/genes/2081) | ERN1 | endoplasmic reticulum to nucleus signaling 1 |
| [30001](http://www.autophagy.lu/genes/30001) | ERO1L | ERO1-like (S. cerevisiae) |
| [8772](http://www.autophagy.lu/genes/8772) | FADD | Fas (TNFRSF6)-associated via death domain |
| [55578](http://www.autophagy.lu/genes/55578) | FAM48A | family with sequence similarity 48, member A |
| [355](http://www.autophagy.lu/genes/355) | FAS | Fas (TNF receptor superfamily, member 6) |
| [2280](http://www.autophagy.lu/genes/2280) | FKBP1A | FK506 binding protein 1A, 12kDa |
| [2281](http://www.autophagy.lu/genes/2281) | FKBP1B | FK506 binding protein 1B, 12.6 kDa |
| [2353](http://www.autophagy.lu/genes/2353) | FOS | FBJ murine osteosarcoma viral oncogene homolog |
| [2308](http://www.autophagy.lu/genes/2308) | FOXO1 | forkhead box O1 |
| [2309](http://www.autophagy.lu/genes/2309) | FOXO3 | forkhead box O3 |
| [2548](http://www.autophagy.lu/genes/2548) | GAA | glucosidase, alpha; acid |
| [2548](http://www.autophagy.lu/genes/2548) | GAA | glucosidase, alpha; acid |
| [11337](http://www.autophagy.lu/genes/11337) | GABARAP | GABA(A) receptor-associated protein |
| [11337](http://www.autophagy.lu/genes/11337) | GABARAP | GABA(A) receptor-associated protein |
| [23710](http://www.autophagy.lu/genes/23710) | GABARAPL1 | GABA(A) receptor-associated protein like 1 |
| [23710](http://www.autophagy.lu/genes/23710) | GABARAPL1 | GABA(A) receptor-associated protein like 1 |
| [11345](http://www.autophagy.lu/genes/11345) | GABARAPL2 | GABA(A) receptor-associated protein-like 2 |
| [11345](http://www.autophagy.lu/genes/11345) | GABARAPL2 | GABA(A) receptor-associated protein-like 2 |
| [2597](http://www.autophagy.lu/genes/2597) | GAPDH | glyceraldehyde-3-phosphate dehydrogenase |
| [2597](http://www.autophagy.lu/genes/2597) | GAPDH | glyceraldehyde-3-phosphate dehydrogenase |
| [2773](http://www.autophagy.lu/genes/2773) | GNAI3 | guanine nucleotide binding protein (G protein), alpha inhibiting activity polypeptide 3 |
| [2773](http://www.autophagy.lu/genes/2773) | GNAI3 | guanine nucleotide binding protein (G protein), alpha inhibiting activity polypeptide 3 |
| [10399](http://www.autophagy.lu/genes/10399) | GNB2L1 | guanine nucleotide binding protein (G protein), beta polypeptide 2-like 1 |
| [10399](http://www.autophagy.lu/genes/10399) | GNB2L1 | guanine nucleotide binding protein (G protein), beta polypeptide 2-like 1 |
| [57120](http://www.autophagy.lu/genes/57120) | GOPC | golgi-associated PDZ and coiled-coil motif containing |
| [57120](http://www.autophagy.lu/genes/57120) | GOPC | golgi-associated PDZ and coiled-coil motif containing |
| [2894](http://www.autophagy.lu/genes/2894) | GRID1 | glutamate receptor, ionotropic, delta 1 |
| [2894](http://www.autophagy.lu/genes/2894) | GRID1 | glutamate receptor, ionotropic, delta 1 |
| [2895](http://www.autophagy.lu/genes/2895) | GRID2 | glutamate receptor, ionotropic, delta 2 |
| [2895](http://www.autophagy.lu/genes/2895) | GRID2 | glutamate receptor, ionotropic, delta 2 |
| [3065](http://www.autophagy.lu/genes/3065) | HDAC1 | histone deacetylase 1 |
| [10013](http://www.autophagy.lu/genes/10013) | HDAC6 | histone deacetylase 6 |
| [9146](http://www.autophagy.lu/genes/9146) | HGS | hepatocyte growth factor-regulated tyrosine kinase substrate |
| [3091](http://www.autophagy.lu/genes/3091) | HIF1A | hypoxia inducible factor 1, alpha subunit (basic helix-loop-helix transcription factor) |
| [3326](http://www.autophagy.lu/genes/3326) | HSP90AB1 | heat shock protein 90kDa alpha (cytosolic), class B member 1 |
| [3309](http://www.autophagy.lu/genes/3309) | HSPA5 | heat shock 70kDa protein 5 (glucose-regulated protein, 78kDa) |
| [3312](http://www.autophagy.lu/genes/3312) | HSPA8 | heat shock 70kDa protein 8 |
| [26353](http://www.autophagy.lu/genes/26353) | HSPB8 | heat shock 22kDa protein 8 |
| [3458](http://www.autophagy.lu/genes/3458) | IFNG | interferon, gamma |
| [3551](http://www.autophagy.lu/genes/3551) | IKBKB | inhibitor of kappa light polypeptide gene enhancer in B-cells, kinase beta |
| [9641](http://www.autophagy.lu/genes/9641) | IKBKE | inhibitor of kappa light polypeptide gene enhancer in B-cells, kinase epsilon |
| [11009](http://www.autophagy.lu/genes/11009) | IL24 | interleukin 24 |
| [345611](http://www.autophagy.lu/genes/345611) | IRGM | immunity-related GTPase family, M |
| [3675](http://www.autophagy.lu/genes/3675) | ITGA3 | integrin, alpha 3 (antigen CD49C, alpha 3 subunit of VLA-3 receptor) |
| [3655](http://www.autophagy.lu/genes/3655) | ITGA6 | integrin, alpha 6 |
| [3688](http://www.autophagy.lu/genes/3688) | ITGB1 | integrin, beta 1 (fibronectin receptor, beta polypeptide, antigen CD29 includes MDF2, MSK12) |
| [3691](http://www.autophagy.lu/genes/3691) | ITGB4 | integrin, beta 4 |
| [3708](http://www.autophagy.lu/genes/3708) | ITPR1 | inositol 1,4,5-triphosphate receptor, type 1 |
| [9711](http://www.autophagy.lu/genes/9711) | KIAA0226 | KIAA0226 |
| [9776](http://www.autophagy.lu/genes/9776) | KIAA0652 | KIAA0652 |
| [22863](http://www.autophagy.lu/genes/22863) | KIAA0831 | KIAA0831 |
| [3799](http://www.autophagy.lu/genes/3799) | KIF5B | kinesin family member 5B |
| [54800](http://www.autophagy.lu/genes/54800) | KLHL24 | kelch-like 24 (Drosophila) |
| [3916](http://www.autophagy.lu/genes/3916) | LAMP1 | lysosomal-associated membrane protein 1 |
| [3920](http://www.autophagy.lu/genes/3920) | LAMP2 | lysosomal-associated membrane protein 2 |
| [84557](http://www.autophagy.lu/genes/84557) | MAP1LC3A | microtubule-associated protein 1 light chain 3 alpha |
| [81631](http://www.autophagy.lu/genes/81631) | MAP1LC3B | microtubule-associated protein 1 light chain 3 beta |
| [440738](http://www.autophagy.lu/genes/440738) | MAP1LC3C | microtubule-associated protein 1 light chain 3 gamma |
| [5609](http://www.autophagy.lu/genes/5609) | MAP2K7 | mitogen-activated protein kinase kinase 7 |
| [5594](http://www.autophagy.lu/genes/5594) | MAPK1 | mitogen-activated protein kinase 1 |
| [5595](http://www.autophagy.lu/genes/5595) | MAPK3 | mitogen-activated protein kinase 3 |
| [5599](http://www.autophagy.lu/genes/5599) | MAPK8 | mitogen-activated protein kinase 8 |
| [9479](http://www.autophagy.lu/genes/9479) | MAPK8IP1 | mitogen-activated protein kinase 8 interacting protein 1 |
| [5601](http://www.autophagy.lu/genes/5601) | MAPK9 | mitogen-activated protein kinase 9 |
| [51360](http://www.autophagy.lu/genes/51360) | MBTPS2 | membrane-bound transcription factor peptidase, site 2 |
| [64223](http://www.autophagy.lu/genes/64223) | MLST8 | MTOR associated protein, LST8 homolog (S. cerevisiae) |
| [64419](http://www.autophagy.lu/genes/64419) | MTMR14 | myotubularin related protein 14 |
| [2475](http://www.autophagy.lu/genes/2475) | MTOR | mechanistic target of rapamycin (serine/threonine kinase) |
| [4609](http://www.autophagy.lu/genes/4609) | MYC | v-myc myelocytomatosis viral oncogene homolog (avian) |
| [92345](http://www.autophagy.lu/genes/92345) | NAF1 | nuclear assembly factor 1 homolog (S. cerevisiae) |
| [10135](http://www.autophagy.lu/genes/10135) | NAMPT | nicotinamide phosphoribosyltransferase |
| [4077](http://www.autophagy.lu/genes/4077) | NBR1 | neighbor of BRCA1 gene 1 |
| [10787](http://www.autophagy.lu/genes/10787) | NCKAP1 | NCK-associated protein 1 |
| [4780](http://www.autophagy.lu/genes/4780) | NFE2L2 | nuclear factor (erythroid-derived 2)-like 2 |
| [4790](http://www.autophagy.lu/genes/4790) | NFKB1 | nuclear factor of kappa light polypeptide gene enhancer in B-cells 1 |
| [159296](http://www.autophagy.lu/genes/159296) | NKX2-3 | NK2 transcription factor related, locus 3 (Drosophila) |
| [58484](http://www.autophagy.lu/genes/58484) | NLRC4 | NLR family, CARD domain containing 4 |
| [4864](http://www.autophagy.lu/genes/4864) | NPC1 | Niemann-Pick disease, type C1 |
| [3084](http://www.autophagy.lu/genes/3084) | NRG1 | neuregulin 1 |
| [9542](http://www.autophagy.lu/genes/9542) | NRG2 | neuregulin 2 |
| [10718](http://www.autophagy.lu/genes/10718) | NRG3 | neuregulin 3 |
| [5034](http://www.autophagy.lu/genes/5034) | P4HB | prolyl 4-hydroxylase, beta polypeptide |
| [5071](http://www.autophagy.lu/genes/5071) | PARK2 | Parkinson disease (autosomal recessive, juvenile) 2, parkin |
| [142](http://www.autophagy.lu/genes/142) | PARP1 | poly (ADP-ribose) polymerase 1 |
| [8682](http://www.autophagy.lu/genes/8682) | PEA15 | phosphoprotein enriched in astrocytes 15 |
| [27043](http://www.autophagy.lu/genes/27043) | PELP1 | proline, glutamate and leucine rich protein 1 |
| [5195](http://www.autophagy.lu/genes/5195) | PEX14 | peroxisomal biogenesis factor 14 |
| [8504](http://www.autophagy.lu/genes/8504) | PEX3 | peroxisomal biogenesis factor 3 |
| [5289](http://www.autophagy.lu/genes/5289) | PIK3C3 | phosphoinositide-3-kinase, class 3 |
| [30849](http://www.autophagy.lu/genes/30849) | PIK3R4 | phosphoinositide-3-kinase, regulatory subunit 4 |
| [65018](http://www.autophagy.lu/genes/65018) | PINK1 | PTEN induced putative kinase 1 |
| [23645](http://www.autophagy.lu/genes/23645) | PPP1R15A | protein phosphatase 1, regulatory (inhibitor) subunit 15A |
| [5564](http://www.autophagy.lu/genes/5564) | PRKAB1 | protein kinase, AMP-activated, beta 1 non-catalytic subunit |
| [5573](http://www.autophagy.lu/genes/5573) | PRKAR1A | protein kinase, cAMP-dependent, regulatory, type I, alpha (tissue specific extinguisher 1) |
| [5580](http://www.autophagy.lu/genes/5580) | PRKCD | protein kinase C, delta |
| [5588](http://www.autophagy.lu/genes/5588) | PRKCQ | protein kinase C, theta |
| [5728](http://www.autophagy.lu/genes/5728) | PTEN | phosphatase and tensin homolog |
| [5753](http://www.autophagy.lu/genes/5753) | PTK6 | PTK6 protein tyrosine kinase 6 |
| [8766](http://www.autophagy.lu/genes/8766) | RAB11A | RAB11A, member RAS oncogene family |
| [5861](http://www.autophagy.lu/genes/5861) | RAB1A | RAB1A, member RAS oncogene family |
| [53917](http://www.autophagy.lu/genes/53917) | RAB24 | RAB24, member RAS oncogene family |
| [83452](http://www.autophagy.lu/genes/83452) | RAB33B | RAB33B, member RAS oncogene family |
| [5868](http://www.autophagy.lu/genes/5868) | RAB5A | RAB5A, member RAS oncogene family |
| [7879](http://www.autophagy.lu/genes/7879) | RAB7A | RAB7A, member RAS oncogene family |
| [5879](http://www.autophagy.lu/genes/5879) | RAC1 | ras-related C3 botulinum toxin substrate 1 (rho family, small GTP binding protein Rac1) |
| [5894](http://www.autophagy.lu/genes/5894) | RAF1 | v-raf-1 murine leukemia viral oncogene homolog 1 |
| [5925](http://www.autophagy.lu/genes/5925) | RB1 | retinoblastoma 1 |
| [9821](http://www.autophagy.lu/genes/9821) | RB1CC1 | RB1-inducible coiled-coil 1 |
| [5970](http://www.autophagy.lu/genes/5970) | RELA | v-rel reticuloendotheliosis viral oncogene homolog A (avian) |
| [10287](http://www.autophagy.lu/genes/10287) | RGS19 | regulator of G-protein signaling 19 |
| [6009](http://www.autophagy.lu/genes/6009) | RHEB | Ras homolog enriched in brain |
| [6198](http://www.autophagy.lu/genes/6198) | RPS6KB1 | ribosomal protein S6 kinase, 70kDa, polypeptide 1 |
| [57521](http://www.autophagy.lu/genes/57521) | RPTOR | regulatory associated protein of MTOR, complex 1 |
| [56681](http://www.autophagy.lu/genes/56681) | SAR1A | SAR1 homolog A (S. cerevisiae) |
| [5265](http://www.autophagy.lu/genes/5265) | SERPINA1 | serpin peptidase inhibitor, clade A (alpha-1 antiproteinase, antitrypsin), member 1 |
| [83667](http://www.autophagy.lu/genes/83667) | SESN2 | sestrin 2 |
| [51100](http://www.autophagy.lu/genes/51100) | SH3GLB1 | SH3-domain GRB2-like endophilin B1 |
| [23411](http://www.autophagy.lu/genes/23411) | SIRT1 | sirtuin (silent mating type information regulation 2 homolog) 1 (S. cerevisiae) |
| [22933](http://www.autophagy.lu/genes/22933) | SIRT2 | sirtuin (silent mating type information regulation 2 homolog) 2 (S. cerevisiae) |
| [8877](http://www.autophagy.lu/genes/8877) | SPHK1 | sphingosine kinase 1 |
| [83985](http://www.autophagy.lu/genes/83985) | SPNS1 | spinster homolog 1 (Drosophila) |
| [8878](http://www.autophagy.lu/genes/8878) | SQSTM1 | sequestosome 1 |
| [6767](http://www.autophagy.lu/genes/6767) | ST13 | suppression of tumorigenicity 13 (colon carcinoma) (Hsp70 interacting protein) |
| [6794](http://www.autophagy.lu/genes/6794) | STK11 | serine/threonine kinase 11 |
| [29110](http://www.autophagy.lu/genes/29110) | TBK1 | TANK-binding kinase 1 |
| [10548](http://www.autophagy.lu/genes/10548) | TM9SF1 | transmembrane 9 superfamily member 1 |
| [81671](http://www.autophagy.lu/genes/81671) | TMEM49 | transmembrane protein 49 |
| [157753](http://www.autophagy.lu/genes/157753) | TMEM74 | transmembrane protein 74 |
| [8743](http://www.autophagy.lu/genes/8743) | TNFSF10 | tumor necrosis factor (ligand) superfamily, member 10 |
| [7157](http://www.autophagy.lu/genes/7157) | TP53 | tumor protein p53 |
| [58476](http://www.autophagy.lu/genes/58476) | TP53INP2 | tumor protein p53 inducible nuclear protein 2 |
| [8626](http://www.autophagy.lu/genes/8626) | TP63 | tumor protein p63 |
| [7161](http://www.autophagy.lu/genes/7161) | TP73 | tumor protein p73 |
| [7248](http://www.autophagy.lu/genes/7248) | TSC1 | tuberous sclerosis 1 |
| [7249](http://www.autophagy.lu/genes/7249) | TSC2 | tuberous sclerosis 2 |
| [286319](http://www.autophagy.lu/genes/286319) | TUSC1 | tumor suppressor candidate 1 |
| [8408](http://www.autophagy.lu/genes/8408) | ULK1 | unc-51-like kinase 1 (C. elegans) |
| [9706](http://www.autophagy.lu/genes/9706) | ULK2 | unc-51-like kinase 2 (C. elegans) |
| [25989](http://www.autophagy.lu/genes/25989) | ULK3 | unc-51-like kinase 3 (C. elegans) |
| [9100](http://www.autophagy.lu/genes/9100) | USP10 | ubiquitin specific peptidase 10 |
| [7405](http://www.autophagy.lu/genes/7405) | UVRAG | UV radiation resistance associated gene |
| [9341](http://www.autophagy.lu/genes/9341) | VAMP3 | vesicle-associated membrane protein 3 (cellubrevin) |
| [6845](http://www.autophagy.lu/genes/6845) | VAMP7 | vesicle-associated membrane protein 7 |
| [7422](http://www.autophagy.lu/genes/7422) | VEGFA | vascular endothelial growth factor A |
| [23001](http://www.autophagy.lu/genes/23001) | WDFY3 | WD repeat and FYVE domain containing 3 |
| [11152](http://www.autophagy.lu/genes/11152) | WDR45 | WD repeat domain 45 |
| [56270](http://www.autophagy.lu/genes/56270) | WDR45L | WDR45-like |
| [55062](http://www.autophagy.lu/genes/55062) | WIPI1 | WD repeat domain, phosphoinositide interacting 1 |
| [26100](http://www.autophagy.lu/genes/26100) | WIPI2 | WD repeat domain, phosphoinositide interacting 2 |
| [53349](http://www.autophagy.lu/genes/53349) | ZFYVE1 | zinc finger, FYVE domain containing 1 |
