# Supplementary material for: Identification of Three Autophagy-Related Long Non-Coding RNAs as a Novel Head and Neck Squamous Cell Carcinoma Prognostic Signature
Source: Front Oncol. 2021 Jan 26;10:603864. doi: 10.3389/fonc.2020.603864 (PMC7871905; doi:10.3389/fonc.2020.603864)
Supplement: Supplementary file 3 [file DataSheet_3.doc]

**Supplementary material 3**

**1153 autophagy related IncRNAs by performing Pearson correlation coefficient**

ARGgene, autophagy related gene. IncRNA, Long non-coding RNA. Cor, coefficent.

| ARGgene | lncRNA | cor | pvalue |
| --- | --- | --- | --- |
| BIRC5 | AC108047.1 | 0.306085972 | 0.000107382 |
| PIK3C3 | AC108047.1 | 0.336607785 | 1.85E-05 |
| CASP1 | AC005392.2 | 0.413867675 | 8.66E-08 |
| CASP4 | AC005392.2 | 0.341965616 | 1.33E-05 |
| RAB11A | AC005392.2 | 0.337027238 | 1.80E-05 |
| STK11 | AC005392.2 | 0.324120189 | 3.88E-05 |
| ATG16L2 | AL360181.2 | 0.315673724 | 6.31E-05 |
| ATG2A | AL360181.2 | 0.316704157 | 5.95E-05 |
| CXCR4 | AL360181.2 | 0.325743717 | 3.53E-05 |
| EDEM1 | AL360181.2 | 0.347547911 | 9.39E-06 |
| EEF2K | AL360181.2 | 0.333001691 | 2.30E-05 |
| ERN1 | AL360181.2 | 0.314506353 | 6.73E-05 |
| IKBKB | AL360181.2 | 0.349350428 | 8.37E-06 |
| MAPK8 | AL360181.2 | 0.35849616 | 4.64E-06 |
| MTOR | AL360181.2 | 0.359967597 | 4.22E-06 |
| SIRT1 | AL360181.2 | 0.355358025 | 5.70E-06 |
| SPNS1 | AL360181.2 | 0.306812715 | 0.0001032 |
| TP73 | AL360181.2 | 0.305209405 | 0.000112637 |
| TSC1 | AL360181.2 | 0.330119253 | 2.73E-05 |
| TSC2 | AL360181.2 | 0.330755101 | 2.63E-05 |
| VEGFA | AL360181.2 | 0.349120729 | 8.50E-06 |
| GABARAPL1 | AL391427.1 | 0.38985791 | 5.32E-07 |
| NFE2L2 | AL391427.1 | 0.348548185 | 8.81E-06 |
| RHEB | AL391427.1 | 0.356899606 | 5.15E-06 |
| TP63 | AL391427.1 | 0.361668688 | 3.77E-06 |
| WIPI1 | AL391427.1 | 0.353792906 | 6.30E-06 |
| WIPI1 | AC114956.1 | 0.333124902 | 2.28E-05 |
| ATG10 | AC092718.4 | 0.303600526 | 0.000122911 |
| ATG3 | AC092718.4 | 0.370089948 | 2.14E-06 |
| ATG4B | AC092718.4 | 0.419902351 | 5.36E-08 |
| BAX | AC092718.4 | 0.430676778 | 2.22E-08 |
| BIRC5 | AC092718.4 | 0.3745 | 1.58E-06 |
| BNIP1 | AC092718.4 | 0.307708883 | 9.83E-05 |
| CAPNS1 | AC092718.4 | 0.347671688 | 9.31E-06 |
| CDKN2A | AC092718.4 | 0.442541864 | 8.14E-09 |
| CX3CL1 | AC092718.4 | 0.417753593 | 6.36E-08 |
| DLC1 | AC092718.4 | -0.314993381 | 6.55E-05 |
| ERN1 | AC092718.4 | -0.303456241 | 0.000123874 |
| MTMR14 | AC092718.4 | 0.332022259 | 2.44E-05 |
| PEX14 | AC092718.4 | 0.387541947 | 6.30E-07 |
| PINK1 | AC092718.4 | -0.318891836 | 5.25E-05 |
| EIF2AK3 | OR2A1-AS1 | 0.388638028 | 5.82E-07 |
| FOXO3 | OR2A1-AS1 | 0.465001348 | 1.09E-09 |
| NFE2L2 | OR2A1-AS1 | 0.449189375 | 4.56E-09 |
| RHEB | OR2A1-AS1 | 0.380984627 | 1.01E-06 |
| TP63 | OR2A1-AS1 | 0.362154679 | 3.65E-06 |
| GABARAPL1 | TM4SF1-AS1 | 0.305190768 | 0.000112751 |
| NFE2L2 | TM4SF1-AS1 | 0.318260251 | 5.44E-05 |
| TP63 | TM4SF1-AS1 | 0.356927723 | 5.14E-06 |
| AMBRA1 | AC025048.4 | -0.335486303 | 1.98E-05 |
| ARSB | AC025048.4 | -0.305308597 | 0.00011203 |
| ATG9B | AC025048.4 | 0.461193745 | 1.55E-09 |
| EEF2 | AC025048.4 | -0.305354008 | 0.000111754 |
| EIF2AK2 | AC025048.4 | -0.309121838 | 9.09E-05 |
| FOXO1 | AC025048.4 | -0.328071341 | 3.08E-05 |
| LAMP2 | AC025048.4 | -0.325886718 | 3.50E-05 |
| RAB24 | AC025048.4 | 0.332214449 | 2.41E-05 |
| TM9SF1 | AC025048.4 | -0.30006919 | 0.000148606 |
| USP10 | AC025048.4 | -0.309024864 | 9.14E-05 |
| MAP1LC3B | AL583808.1 | 0.34451835 | 1.14E-05 |
| CDKN1A | MIR22HG | 0.334673225 | 2.08E-05 |
| RAB1A | MIR22HG | 0.311200131 | 8.10E-05 |
| ARNT | AL132712.2 | 0.300489856 | 0.000145302 |
| ATG2B | AL132712.2 | 0.339146716 | 1.58E-05 |
| ATG16L2 | AL050341.2 | 0.307672331 | 9.85E-05 |
| ATG4C | AL050341.2 | 0.329528434 | 2.83E-05 |
| CAPN10 | AL050341.2 | 0.349917133 | 8.08E-06 |
| CD46 | AL050341.2 | 0.327673105 | 3.16E-05 |
| GABARAPL2 | AL050341.2 | 0.316254923 | 6.10E-05 |
| HDAC1 | AL050341.2 | 0.34326414 | 1.23E-05 |
| HDAC6 | AL050341.2 | 0.438377926 | 1.16E-08 |
| MAP2K7 | AL050341.2 | 0.300906358 | 0.000142098 |
| ULK3 | AL050341.2 | 0.323460337 | 4.04E-05 |
| WDR45 | AL050341.2 | 0.332043616 | 2.43E-05 |
| AMBRA1 | AC108449.2 | 0.437764165 | 1.23E-08 |
| ARNT | AC108449.2 | 0.603552994 | 9.41E-17 |
| ARSB | AC108449.2 | 0.547535811 | 1.67E-13 |
| ATF4 | AC108449.2 | -0.306236562 | 0.000106503 |
| ATF6 | AC108449.2 | 0.384567504 | 7.80E-07 |
| ATG2A | AC108449.2 | 0.32404439 | 3.90E-05 |
| ATG2B | AC108449.2 | 0.402269285 | 2.12E-07 |
| BCL2 | AC108449.2 | 0.508163389 | 1.48E-11 |
| BIRC6 | AC108449.2 | 0.657190418 | 1.59E-20 |
| BNIP3L | AC108449.2 | 0.36206821 | 3.67E-06 |
| CAMKK2 | AC108449.2 | 0.385589155 | 7.25E-07 |
| CANX | AC108449.2 | 0.34034768 | 1.47E-05 |
| CCR2 | AC108449.2 | 0.41604772 | 7.29E-08 |
| CD46 | AC108449.2 | 0.377866033 | 1.25E-06 |
| CDKN1B | AC108449.2 | 0.403510505 | 1.93E-07 |
| DAPK1 | AC108449.2 | 0.334899313 | 2.05E-05 |
| DLC1 | AC108449.2 | 0.309398995 | 8.95E-05 |
| DRAM1 | AC108449.2 | 0.394812748 | 3.70E-07 |
| EDEM1 | AC108449.2 | 0.415700128 | 7.49E-08 |
| EEF2 | AC108449.2 | 0.326637522 | 3.35E-05 |
| EEF2K | AC108449.2 | 0.559172409 | 3.97E-14 |
| EIF2AK3 | AC108449.2 | 0.492644815 | 7.45E-11 |
| EIF4EBP1 | AC108449.2 | -0.303660461 | 0.000122513 |
| ERN1 | AC108449.2 | 0.325245574 | 3.64E-05 |
| FAS | AC108449.2 | 0.37635879 | 1.39E-06 |
| FOXO1 | AC108449.2 | 0.354651281 | 5.96E-06 |
| FOXO3 | AC108449.2 | 0.464797866 | 1.11E-09 |
| HDAC6 | AC108449.2 | 0.322986927 | 4.15E-05 |
| IKBKE | AC108449.2 | 0.333263523 | 2.26E-05 |
| ITPR1 | AC108449.2 | 0.437594179 | 1.24E-08 |
| KIF5B | AC108449.2 | 0.323172052 | 4.10E-05 |
| KLHL24 | AC108449.2 | 0.420769181 | 5.00E-08 |
| MAPK1 | AC108449.2 | 0.384342026 | 7.92E-07 |
| MAPK8 | AC108449.2 | 0.395822158 | 3.44E-07 |
| MBTPS2 | AC108449.2 | 0.407298299 | 1.44E-07 |
| MTOR | AC108449.2 | 0.53501634 | 7.42E-13 |
| NAF1 | AC108449.2 | 0.406199701 | 1.57E-07 |
| NBR1 | AC108449.2 | 0.506974613 | 1.68E-11 |
| NFKB1 | AC108449.2 | 0.354173068 | 6.15E-06 |
| NKX2-3 | AC108449.2 | 0.347066358 | 9.68E-06 |
| PARP1 | AC108449.2 | 0.487409863 | 1.26E-10 |
| PIK3C3 | AC108449.2 | 0.311623986 | 7.91E-05 |
| PIK3R4 | AC108449.2 | 0.548167828 | 1.55E-13 |
| PRKAB1 | AC108449.2 | 0.320958823 | 4.66E-05 |
| RAB33B | AC108449.2 | 0.423999179 | 3.85E-08 |
| RAF1 | AC108449.2 | 0.331100938 | 2.57E-05 |
| RB1CC1 | AC108449.2 | 0.47254392 | 5.37E-10 |
| SIRT1 | AC108449.2 | 0.496568596 | 4.99E-11 |
| SPNS1 | AC108449.2 | 0.349833968 | 8.12E-06 |
| TP53 | AC108449.2 | 0.389346956 | 5.53E-07 |
| TP73 | AC108449.2 | 0.385285737 | 7.41E-07 |
| TSC1 | AC108449.2 | 0.480956545 | 2.38E-10 |
| TSC2 | AC108449.2 | 0.380422286 | 1.05E-06 |
| ULK2 | AC108449.2 | 0.36509268 | 3.00E-06 |
| UVRAG | AC108449.2 | 0.395533237 | 3.51E-07 |
| WDFY3 | AC108449.2 | 0.393718376 | 4.01E-07 |
| ATG16L2 | LINC01176 | 0.405731347 | 1.63E-07 |
| BAX | LINC01176 | 0.313457821 | 7.14E-05 |
| BIRC6 | LINC01176 | -0.398169073 | 2.89E-07 |
| CAPN10 | LINC01176 | 0.314945544 | 6.57E-05 |
| DLC1 | LINC01176 | -0.329265895 | 2.87E-05 |
| EIF4G1 | LINC01176 | -0.302917049 | 0.000127535 |
| KIF5B | LINC01176 | -0.351787518 | 7.17E-06 |
| MAPK1 | LINC01176 | -0.374985829 | 1.53E-06 |
| MBTPS2 | LINC01176 | -0.328620193 | 2.98E-05 |
| NBR1 | LINC01176 | -0.326430772 | 3.39E-05 |
| NCKAP1 | LINC01176 | -0.302066478 | 0.000133517 |
| RAB24 | LINC01176 | 0.444336613 | 6.97E-09 |
| RPTOR | LINC01176 | -0.325332555 | 3.62E-05 |
| USP10 | LINC01176 | -0.305823874 | 0.000108929 |
| WDFY3 | LINC01176 | -0.338336737 | 1.66E-05 |
| ATIC | AC005076.1 | 0.321329778 | 4.57E-05 |
| BAX | AC005076.1 | 0.324195682 | 3.87E-05 |
| BIRC5 | AC005076.1 | 0.425551494 | 3.39E-08 |
| GABARAPL2 | AC005076.1 | 0.358807947 | 4.55E-06 |
| MAPK8IP1 | AC005076.1 | 0.374611031 | 1.57E-06 |
| WDR45 | AC005076.1 | 0.316565394 | 5.99E-05 |
| MAPK8 | LINC00511 | 0.314839181 | 6.61E-05 |
| PRKAB1 | LINC00511 | 0.419758284 | 5.42E-08 |
| ATG10 | AC005696.1 | 0.321003578 | 4.65E-05 |
| ATG3 | AC005696.1 | 0.406069063 | 1.59E-07 |
| ATG4B | AC005696.1 | 0.350675409 | 7.70E-06 |
| ATG4C | AC005696.1 | 0.303357473 | 0.000124537 |
| ATG4D | AC005696.1 | 0.555698003 | 6.13E-14 |
| BNIP1 | AC005696.1 | 0.320214907 | 4.87E-05 |
| CAPN10 | AC005696.1 | 0.385282489 | 7.41E-07 |
| CDKN1B | AC005696.1 | 0.391210763 | 4.82E-07 |
| CDKN2A | AC005696.1 | 0.359193395 | 4.44E-06 |
| EEF2K | AC005696.1 | 0.308416733 | 9.45E-05 |
| HDAC6 | AC005696.1 | 0.405506566 | 1.66E-07 |
| MAP2K7 | AC005696.1 | 0.563844092 | 2.19E-14 |
| MAPK8 | AC005696.1 | 0.344755146 | 1.12E-05 |
| MAPK9 | AC005696.1 | 0.326315838 | 3.42E-05 |
| NKX2-3 | AC005696.1 | 0.395529054 | 3.51E-07 |
| PELP1 | AC005696.1 | 0.364619629 | 3.10E-06 |
| PEX14 | AC005696.1 | 0.300183721 | 0.0001477 |
| PINK1 | AC005696.1 | -0.308194062 | 9.57E-05 |
| PRKAB1 | AC005696.1 | 0.373108427 | 1.74E-06 |
| RAF1 | AC005696.1 | 0.432231993 | 1.95E-08 |
| STK11 | AC005696.1 | 0.466087594 | 9.85E-10 |
| TP53 | AC005696.1 | 0.406772242 | 1.50E-07 |
| TSC2 | AC005696.1 | 0.380507406 | 1.04E-06 |
| ULK3 | AC005696.1 | 0.402230729 | 2.13E-07 |
| WDR45 | AC005696.1 | 0.317661311 | 5.63E-05 |
| CDKN1B | PCAT7 | 0.316019875 | 6.18E-05 |
| HDAC6 | PCAT7 | 0.31716357 | 5.79E-05 |
| MAP2K7 | PCAT7 | 0.323019027 | 4.14E-05 |
| SPNS1 | PCAT7 | 0.351215405 | 7.44E-06 |
| ARNT | AC254633.1 | 0.32846295 | 3.01E-05 |
| ATG4C | AC254633.1 | 0.312296042 | 7.62E-05 |
| ATG7 | AC254633.1 | 0.455381371 | 2.63E-09 |
| BCL2 | AC254633.1 | 0.423803543 | 3.91E-08 |
| CD46 | AC254633.1 | 0.406482074 | 1.54E-07 |
| CDKN1B | AC254633.1 | 0.361739156 | 3.75E-06 |
| EDEM1 | AC254633.1 | 0.347043304 | 9.69E-06 |
| EEF2K | AC254633.1 | 0.394854082 | 3.69E-07 |
| FAS | AC254633.1 | 0.30873816 | 9.28E-05 |
| HDAC6 | AC254633.1 | 0.349659696 | 8.21E-06 |
| IKBKE | AC254633.1 | 0.408544374 | 1.31E-07 |
| MAPK8 | AC254633.1 | 0.441004205 | 9.29E-09 |
| MTOR | AC254633.1 | 0.302103357 | 0.000133252 |
| NBR1 | AC254633.1 | 0.321861993 | 4.43E-05 |
| NKX2-3 | AC254633.1 | 0.414783936 | 8.05E-08 |
| PARP1 | AC254633.1 | 0.331854933 | 2.46E-05 |
| PIK3R4 | AC254633.1 | 0.406851174 | 1.49E-07 |
| PRKAB1 | AC254633.1 | 0.441013763 | 9.29E-09 |
| RAB33B | AC254633.1 | 0.331719388 | 2.48E-05 |
| RAB5A | AC254633.1 | 0.319942442 | 4.95E-05 |
| RAF1 | AC254633.1 | 0.43526466 | 1.51E-08 |
| TP53 | AC254633.1 | 0.418048258 | 6.22E-08 |
| TP73 | AC254633.1 | 0.31230906 | 7.62E-05 |
| TSC1 | AC254633.1 | 0.321378002 | 4.55E-05 |
| TSC2 | AC254633.1 | 0.406567126 | 1.53E-07 |
| BCL2 | AC009159.3 | 0.301199176 | 0.000139885 |
| MAPK8 | AC009159.3 | 0.343112154 | 1.24E-05 |
| NKX2-3 | AC009159.3 | 0.325489709 | 3.59E-05 |
| ARNT | SNHG3 | -0.300510754 | 0.000145139 |
| ATF4 | SNHG3 | 0.313140312 | 7.27E-05 |
| ATG16L2 | SNHG3 | 0.499862342 | 3.55E-11 |
| ATG4B | SNHG3 | 0.355825716 | 5.53E-06 |
| ATIC | SNHG3 | 0.337488517 | 1.75E-05 |
| BAX | SNHG3 | 0.341617267 | 1.36E-05 |
| BIRC5 | SNHG3 | 0.384712318 | 7.72E-07 |
| BNIP1 | SNHG3 | 0.357912641 | 4.82E-06 |
| CAPN10 | SNHG3 | 0.499711767 | 3.61E-11 |
| EIF4G1 | SNHG3 | -0.303902582 | 0.000120918 |
| FOXO1 | SNHG3 | -0.339820657 | 1.52E-05 |
| FOXO3 | SNHG3 | -0.301879129 | 0.000134869 |
| GABARAPL2 | SNHG3 | 0.352966706 | 6.65E-06 |
| LAMP2 | SNHG3 | -0.305885609 | 0.000108563 |
| MAPK1 | SNHG3 | -0.340630399 | 1.45E-05 |
| MAPK8IP1 | SNHG3 | 0.300290652 | 0.000146858 |
| MLST8 | SNHG3 | 0.317237939 | 5.77E-05 |
| PEX14 | SNHG3 | 0.31742446 | 5.71E-05 |
| RAB24 | SNHG3 | 0.319039659 | 5.21E-05 |
| STK11 | SNHG3 | 0.309258878 | 9.02E-05 |
| ULK3 | SNHG3 | 0.305706835 | 0.000109626 |
| WDR45 | SNHG3 | 0.370148987 | 2.13E-06 |
| ZFYVE1 | SNHG3 | -0.32503484 | 3.68E-05 |
| TP63 | MIR2117HG | 0.304541549 | 0.0001168 |
| ATG10 | AC090425.1 | 0.411196219 | 1.07E-07 |
| ATG3 | AC090425.1 | 0.339040724 | 1.59E-05 |
| ATG4B | AC090425.1 | 0.517567371 | 5.35E-12 |
| ATG4D | AC090425.1 | 0.420676033 | 5.04E-08 |
| BAX | AC090425.1 | 0.519446119 | 4.35E-12 |
| BID | AC090425.1 | 0.354204996 | 6.14E-06 |
| BNIP1 | AC090425.1 | 0.362278835 | 3.62E-06 |
| CAPN10 | AC090425.1 | 0.424550869 | 3.68E-08 |
| CAPNS1 | AC090425.1 | 0.398194186 | 2.88E-07 |
| CDKN2A | AC090425.1 | 0.479455536 | 2.76E-10 |
| CLN3 | AC090425.1 | 0.317092057 | 5.82E-05 |
| CX3CL1 | AC090425.1 | 0.319466794 | 5.08E-05 |
| CXCR4 | AC090425.1 | 0.409506289 | 1.22E-07 |
| DAPK2 | AC090425.1 | 0.380009139 | 1.08E-06 |
| FKBP1A | AC090425.1 | 0.335958554 | 1.92E-05 |
| GABARAP | AC090425.1 | 0.393260416 | 4.15E-07 |
| HDAC6 | AC090425.1 | 0.308963539 | 9.17E-05 |
| HIF1A | AC090425.1 | -0.308564511 | 9.37E-05 |
| ITGA6 | AC090425.1 | -0.341460634 | 1.37E-05 |
| MAP2K7 | AC090425.1 | 0.406705562 | 1.51E-07 |
| MTMR14 | AC090425.1 | 0.425862345 | 3.31E-08 |
| PELP1 | AC090425.1 | 0.45244728 | 3.41E-09 |
| PEX14 | AC090425.1 | 0.353206116 | 6.55E-06 |
| PINK1 | AC090425.1 | -0.376156116 | 1.41E-06 |
| RAB24 | AC090425.1 | 0.359034198 | 4.48E-06 |
| STK11 | AC090425.1 | 0.45813209 | 2.05E-09 |
| TP53 | AC090425.1 | 0.315184574 | 6.48E-05 |
| TSC2 | AC090425.1 | 0.336422332 | 1.87E-05 |
| WDFY3 | AC090425.1 | -0.30954923 | 8.88E-05 |
| WDR45 | AC090425.1 | 0.40757294 | 1.41E-07 |
| ARNT | AC015849.3 | 0.395027175 | 3.64E-07 |
| ARSB | AC015849.3 | 0.326612389 | 3.36E-05 |
| BCL2 | AC015849.3 | 0.35104745 | 7.52E-06 |
| BECN1 | AC015849.3 | 0.315261999 | 6.45E-05 |
| BIRC6 | AC015849.3 | 0.359027601 | 4.48E-06 |
| CAMKK2 | AC015849.3 | 0.324918312 | 3.71E-05 |
| CD46 | AC015849.3 | 0.381734396 | 9.54E-07 |
| CDKN1B | AC015849.3 | 0.393714546 | 4.01E-07 |
| CXCR4 | AC015849.3 | 0.377751231 | 1.26E-06 |
| EDEM1 | AC015849.3 | 0.307587788 | 9.89E-05 |
| EEF2K | AC015849.3 | 0.48377169 | 1.81E-10 |
| EIF2AK3 | AC015849.3 | 0.35801273 | 4.79E-06 |
| HDAC6 | AC015849.3 | 0.499047703 | 3.86E-11 |
| IKBKB | AC015849.3 | 0.386948362 | 6.57E-07 |
| KLHL24 | AC015849.3 | 0.346548781 | 1.00E-05 |
| MAP2K7 | AC015849.3 | 0.403670092 | 1.91E-07 |
| MAPK8 | AC015849.3 | 0.38329986 | 8.53E-07 |
| MAPK9 | AC015849.3 | 0.3187449 | 5.30E-05 |
| MTOR | AC015849.3 | 0.392493388 | 4.39E-07 |
| NBR1 | AC015849.3 | 0.371732772 | 1.91E-06 |
| NKX2-3 | AC015849.3 | 0.361575314 | 3.79E-06 |
| PARP1 | AC015849.3 | 0.405185356 | 1.70E-07 |
| PIK3R4 | AC015849.3 | 0.372673812 | 1.79E-06 |
| PRKAB1 | AC015849.3 | 0.308449176 | 9.43E-05 |
| RAF1 | AC015849.3 | 0.383275466 | 8.55E-07 |
| RB1CC1 | AC015849.3 | 0.323494644 | 4.03E-05 |
| SIRT1 | AC015849.3 | 0.402006228 | 2.16E-07 |
| SPNS1 | AC015849.3 | 0.378785494 | 1.17E-06 |
| TP53 | AC015849.3 | 0.359758296 | 4.27E-06 |
| TP73 | AC015849.3 | 0.402383526 | 2.10E-07 |
| TSC1 | AC015849.3 | 0.467618884 | 8.54E-10 |
| TSC2 | AC015849.3 | 0.426369412 | 3.17E-08 |
| ULK1 | AC015849.3 | 0.344357186 | 1.15E-05 |
| ULK2 | AC015849.3 | 0.389337827 | 5.53E-07 |
| UVRAG | AC015849.3 | 0.353648252 | 6.36E-06 |
| BNIP3 | AC011632.1 | 0.314056552 | 6.91E-05 |
| CXCR4 | AL512329.2 | 0.355607259 | 5.61E-06 |
| DAPK2 | AL512329.2 | 0.327755238 | 3.14E-05 |
| TP53 | AL512329.2 | 0.323507818 | 4.03E-05 |
| RAB1A | AC087612.1 | 0.306881725 | 0.000102811 |
| PINK1 | GPRC5D-AS1 | -0.32856266 | 2.99E-05 |
| ARSB | BAALC-AS1 | 0.300785703 | 0.000143019 |
| MBTPS2 | BAALC-AS1 | 0.399299916 | 2.65E-07 |
| ST13 | BAALC-AS1 | 0.343384985 | 1.22E-05 |
| ULK2 | BAALC-AS1 | 0.325616459 | 3.56E-05 |
| WIPI1 | BAALC-AS1 | 0.308064263 | 9.64E-05 |
| ATG16L2 | AC004687.1 | 0.402598897 | 2.07E-07 |
| ATG4B | AC004687.1 | 0.470879793 | 6.28E-10 |
| BAG1 | AC004687.1 | 0.312935851 | 7.35E-05 |
| BAX | AC004687.1 | 0.631599704 | 1.24E-18 |
| BIRC6 | AC004687.1 | -0.30665966 | 0.000104068 |
| BNIP1 | AC004687.1 | 0.320539581 | 4.78E-05 |
| CAPN10 | AC004687.1 | 0.564998428 | 1.89E-14 |
| CAPNS1 | AC004687.1 | 0.330037821 | 2.74E-05 |
| CCR2 | AC004687.1 | 0.304001056 | 0.000120275 |
| CDKN2A | AC004687.1 | 0.349571837 | 8.26E-06 |
| CXCR4 | AC004687.1 | 0.531591286 | 1.10E-12 |
| EIF4G1 | AC004687.1 | -0.345504187 | 1.07E-05 |
| GABARAP | AC004687.1 | 0.317591002 | 5.66E-05 |
| HIF1A | AC004687.1 | -0.322166431 | 4.35E-05 |
| HSP90AB1 | AC004687.1 | -0.305314351 | 0.000111995 |
| IFNG | AC004687.1 | 0.356165582 | 5.41E-06 |
| ITGA6 | AC004687.1 | -0.301731571 | 0.000135943 |
| ITGB1 | AC004687.1 | -0.306925977 | 0.000102562 |
| KIF5B | AC004687.1 | -0.315366685 | 6.42E-05 |
| MAPK1 | AC004687.1 | -0.34987805 | 8.10E-06 |
| MBTPS2 | AC004687.1 | -0.329406096 | 2.85E-05 |
| MTMR14 | AC004687.1 | 0.515625184 | 6.62E-12 |
| NCKAP1 | AC004687.1 | -0.434198995 | 1.66E-08 |
| PEX14 | AC004687.1 | 0.327241732 | 3.24E-05 |
| RAB24 | AC004687.1 | 0.511665937 | 1.02E-11 |
| RGS19 | AC004687.1 | 0.322255698 | 4.33E-05 |
| STK11 | AC004687.1 | 0.384052354 | 8.09E-07 |
| TP63 | AC004687.1 | -0.327364161 | 3.21E-05 |
| ULK3 | AC004687.1 | 0.361415593 | 3.83E-06 |
| USP10 | AC004687.1 | -0.31511425 | 6.51E-05 |
| WDFY3 | AC004687.1 | -0.357386652 | 4.99E-06 |
| WDR45 | AC004687.1 | 0.406039639 | 1.59E-07 |
| ARNT | AC008074.2 | 0.324548629 | 3.79E-05 |
| BID | AC008074.2 | 0.311235938 | 8.09E-05 |
| CASP3 | AC008074.2 | 0.348724147 | 8.71E-06 |
| EEF2K | AC008074.2 | 0.309522133 | 8.89E-05 |
| EIF2AK3 | AC008074.2 | 0.367666739 | 2.52E-06 |
| IKBKE | AC008074.2 | 0.343179681 | 1.23E-05 |
| ITGA3 | AC008074.2 | -0.328968545 | 2.92E-05 |
| NFE2L2 | AC008074.2 | 0.306556587 | 0.000104656 |
| RPS6KB1 | AC008074.2 | 0.354318783 | 6.09E-06 |
| CCR2 | AC004847.1 | 0.312689864 | 7.46E-05 |
| ITGB4 | AC004847.1 | 0.344988113 | 1.10E-05 |
| ITPR1 | AC004847.1 | 0.374027096 | 1.63E-06 |
| ATIC | GS1-124K5.4 | 0.449537456 | 4.42E-09 |
| BAK1 | GS1-124K5.4 | -0.306783496 | 0.000103365 |
| CAPN2 | GS1-124K5.4 | -0.321938429 | 4.41E-05 |
| GNAI3 | GS1-124K5.4 | -0.35005628 | 8.01E-06 |
| ITGA3 | GS1-124K5.4 | -0.372586326 | 1.80E-06 |
| ITGB4 | GS1-124K5.4 | -0.313361663 | 7.18E-05 |
| MAPK8IP1 | GS1-124K5.4 | 0.54112111 | 3.62E-13 |
| NFKB1 | GS1-124K5.4 | -0.309383903 | 8.96E-05 |
| PELP1 | GS1-124K5.4 | 0.351375757 | 7.36E-06 |
| PRKAR1A | GS1-124K5.4 | 0.300902599 | 0.000142126 |
| RHEB | GS1-124K5.4 | 0.304236223 | 0.000118751 |
| ST13 | GS1-124K5.4 | 0.343481776 | 1.21E-05 |
| VAMP3 | GS1-124K5.4 | -0.343457929 | 1.21E-05 |
| WIPI1 | GS1-124K5.4 | 0.429798519 | 2.39E-08 |
| ATG7 | SNHG26 | -0.310314365 | 8.51E-05 |
| BNIP3 | SNHG26 | 0.329001448 | 2.92E-05 |
| GAPDH | SNHG26 | 0.331673488 | 2.49E-05 |
| MTMR14 | SNHG26 | -0.352303283 | 6.94E-06 |
| CXCR4 | LINC01569 | 0.308708972 | 9.30E-05 |
| IKBKE | LINC01569 | 0.331716596 | 2.48E-05 |
| MAPK8 | LINC01569 | 0.301443147 | 0.000138065 |
| MLST8 | LINC01569 | 0.300129596 | 0.000148128 |
| NKX2-3 | LINC01569 | 0.370996787 | 2.01E-06 |
| PARP1 | LINC01569 | 0.341823714 | 1.34E-05 |
| PRKAB1 | LINC01569 | 0.327431589 | 3.20E-05 |
| TP73 | LINC01569 | 0.377964143 | 1.24E-06 |
| TSC2 | LINC01569 | 0.388289441 | 5.97E-07 |
| ARNT | MHENCR | -0.329794344 | 2.78E-05 |
| ATG16L2 | MHENCR | 0.408657483 | 1.30E-07 |
| ATG4B | MHENCR | 0.413928394 | 8.61E-08 |
| ATIC | MHENCR | 0.430236181 | 2.31E-08 |
| BAX | MHENCR | 0.427738434 | 2.84E-08 |
| BIRC5 | MHENCR | 0.489351121 | 1.04E-10 |
| BIRC6 | MHENCR | -0.33816572 | 1.68E-05 |
| BNIP1 | MHENCR | 0.384252909 | 7.97E-07 |
| CAPN10 | MHENCR | 0.441271162 | 9.08E-09 |
| CAPN2 | MHENCR | -0.310443096 | 8.45E-05 |
| CLN3 | MHENCR | 0.4071385 | 1.46E-07 |
| EIF4G1 | MHENCR | -0.341722077 | 1.35E-05 |
| FKBP1A | MHENCR | 0.420837654 | 4.97E-08 |
| GABARAPL2 | MHENCR | 0.42989506 | 2.37E-08 |
| ITGB1 | MHENCR | -0.300835707 | 0.000142637 |
| KIF5B | MHENCR | -0.364932787 | 3.03E-06 |
| LAMP2 | MHENCR | -0.329873394 | 2.77E-05 |
| MAPK1 | MHENCR | -0.443081185 | 7.77E-09 |
| MAPK8IP1 | MHENCR | 0.509021862 | 1.35E-11 |
| MLST8 | MHENCR | 0.392988602 | 4.24E-07 |
| MTMR14 | MHENCR | 0.301732473 | 0.000135936 |
| NCKAP1 | MHENCR | -0.353331795 | 6.49E-06 |
| PEX14 | MHENCR | 0.348388977 | 8.90E-06 |
| RAB24 | MHENCR | 0.359283374 | 4.41E-06 |
| RB1 | MHENCR | -0.300883812 | 0.000142269 |
| STK11 | MHENCR | 0.319587746 | 5.05E-05 |
| TP63 | MHENCR | -0.325809725 | 3.52E-05 |
| WDFY3 | MHENCR | -0.332441853 | 2.38E-05 |
| WDR45 | MHENCR | 0.466854326 | 9.17E-10 |
| ZFYVE1 | MHENCR | -0.306914366 | 0.000102628 |
| ATG16L2 | AC005332.4 | 0.328170263 | 3.06E-05 |
| ATG4C | AC005332.4 | 0.344454288 | 1.14E-05 |
| ATG4D | AC005332.4 | 0.32457916 | 3.78E-05 |
| BAK1 | AC005332.4 | -0.382028612 | 9.34E-07 |
| BCL2 | AC005332.4 | 0.310969395 | 8.21E-05 |
| CAPN10 | AC005332.4 | 0.334224747 | 2.14E-05 |
| CASP3 | AC005332.4 | 0.33473366 | 2.07E-05 |
| CD46 | AC005332.4 | 0.316058825 | 6.17E-05 |
| CDKN1B | AC005332.4 | 0.371075047 | 2.00E-06 |
| EIF2AK3 | AC005332.4 | 0.418146112 | 6.17E-08 |
| ERN1 | AC005332.4 | 0.35426132 | 6.12E-06 |
| GABARAP | AC005332.4 | 0.315059676 | 6.53E-05 |
| HDAC6 | AC005332.4 | 0.529843179 | 1.35E-12 |
| ITGA3 | AC005332.4 | -0.460670575 | 1.62E-09 |
| ITGA6 | AC005332.4 | -0.31719817 | 5.78E-05 |
| ITGB4 | AC005332.4 | -0.312060058 | 7.72E-05 |
| KLHL24 | AC005332.4 | 0.316386691 | 6.06E-05 |
| MAP2K7 | AC005332.4 | 0.342458931 | 1.29E-05 |
| MAPK8IP1 | AC005332.4 | 0.352357586 | 6.91E-06 |
| NFE2L2 | AC005332.4 | 0.346483739 | 1.00E-05 |
| PELP1 | AC005332.4 | 0.32908021 | 2.90E-05 |
| PINK1 | AC005332.4 | -0.349416502 | 8.34E-06 |
| PRKAR1A | AC005332.4 | 0.38758718 | 6.28E-07 |
| RAF1 | AC005332.4 | 0.308421325 | 9.45E-05 |
| RB1CC1 | AC005332.4 | 0.328948347 | 2.93E-05 |
| RHEB | AC005332.4 | 0.338454739 | 1.65E-05 |
| RPS6KB1 | AC005332.4 | 0.336563779 | 1.85E-05 |
| ULK3 | AC005332.4 | 0.385904992 | 7.08E-07 |
| VAMP3 | AC005332.4 | -0.306763559 | 0.000103478 |
| WIPI1 | AC005332.4 | 0.430729252 | 2.21E-08 |
| ATF4 | AC018904.1 | 0.33611819 | 1.90E-05 |
| CAPN10 | AC018904.1 | 0.449923957 | 4.27E-09 |
| CASP3 | AC018904.1 | 0.301427306 | 0.000138183 |
| DAPK2 | AC018904.1 | 0.312988146 | 7.33E-05 |
| DIRAS3 | AC018904.1 | 0.355064253 | 5.81E-06 |
| EIF4G1 | AC018904.1 | -0.301007729 | 0.000141328 |
| GABARAP | AC018904.1 | 0.376423732 | 1.38E-06 |
| PELP1 | AC018904.1 | 0.330127936 | 2.73E-05 |
| ULK3 | AC018904.1 | 0.338894901 | 1.61E-05 |
| ATG4B | AC062017.1 | 0.413845306 | 8.67E-08 |
| BAX | AC062017.1 | 0.387289887 | 6.41E-07 |
| CANX | AC062017.1 | -0.32720406 | 3.24E-05 |
| CAPN10 | AC062017.1 | 0.417564476 | 6.46E-08 |
| CLN3 | AC062017.1 | 0.308747694 | 9.28E-05 |
| FKBP1A | AC062017.1 | 0.325310968 | 3.62E-05 |
| GABARAPL2 | AC062017.1 | 0.446797943 | 5.62E-09 |
| GOPC | AC062017.1 | -0.33765862 | 1.73E-05 |
| KIF5B | AC062017.1 | -0.309235371 | 9.03E-05 |
| MAPK1 | AC062017.1 | -0.385970957 | 7.05E-07 |
| MLST8 | AC062017.1 | 0.342874395 | 1.26E-05 |
| MTOR | AC062017.1 | -0.301555343 | 0.000137236 |
| NFKB1 | AC062017.1 | -0.309263702 | 9.02E-05 |
| RAB24 | AC062017.1 | 0.335284827 | 2.00E-05 |
| RB1 | AC062017.1 | -0.308222642 | 9.55E-05 |
| WDR45 | AC062017.1 | 0.367506046 | 2.55E-06 |
| ATG4B | AC019131.2 | 0.383686387 | 8.30E-07 |
| ATG4D | AC019131.2 | 0.442277515 | 8.33E-09 |
| BCL2 | AC019131.2 | 0.404493609 | 1.79E-07 |
| BID | AC019131.2 | 0.354183518 | 6.15E-06 |
| CAPN10 | AC019131.2 | 0.374010328 | 1.64E-06 |
| CASP3 | AC019131.2 | 0.317746088 | 5.61E-05 |
| CDKN1B | AC019131.2 | 0.345992645 | 1.04E-05 |
| CDKN2A | AC019131.2 | 0.30363463 | 0.000122685 |
| CHMP4B | AC019131.2 | 0.314684534 | 6.67E-05 |
| DRAM1 | AC019131.2 | 0.38949382 | 5.47E-07 |
| HDAC6 | AC019131.2 | 0.379330796 | 1.13E-06 |
| IKBKE | AC019131.2 | 0.330119919 | 2.73E-05 |
| MAP1LC3A | AC019131.2 | 0.428128208 | 2.75E-08 |
| MAP2K7 | AC019131.2 | 0.402114324 | 2.14E-07 |
| MAPK8 | AC019131.2 | 0.421287937 | 4.79E-08 |
| NKX2-3 | AC019131.2 | 0.500588172 | 3.29E-11 |
| PRKAB1 | AC019131.2 | 0.52793153 | 1.68E-12 |
| STK11 | AC019131.2 | 0.397308283 | 3.08E-07 |
| TP73 | AC019131.2 | 0.417135738 | 6.68E-08 |
| TSC2 | AC019131.2 | 0.419699522 | 5.45E-08 |
| ARSB | NRAV | -0.323197263 | 4.10E-05 |
| ATG7 | NRAV | 0.347903009 | 9.18E-06 |
| CAPNS1 | NRAV | 0.32976409 | 2.79E-05 |
| DLC1 | NRAV | -0.325648099 | 3.55E-05 |
| EIF2AK2 | NRAV | -0.312978608 | 7.34E-05 |
| FOXO1 | NRAV | -0.363953219 | 3.24E-06 |
| GABARAPL1 | NRAV | -0.343570919 | 1.20E-05 |
| PEA15 | NRAV | -0.384595303 | 7.78E-07 |
| PTK6 | NRAV | 0.381045726 | 1.00E-06 |
| RAB11A | NRAV | 0.31678289 | 5.92E-05 |
| UVRAG | NRAV | -0.315693697 | 6.30E-05 |
| ARNT | EP300-AS1 | 0.325874948 | 3.51E-05 |
| ATG10 | EP300-AS1 | 0.359633849 | 4.31E-06 |
| ATG2A | EP300-AS1 | 0.392117571 | 4.51E-07 |
| ATG7 | EP300-AS1 | 0.407962576 | 1.37E-07 |
| BCL2 | EP300-AS1 | 0.345817402 | 1.05E-05 |
| BNIP3 | EP300-AS1 | -0.360054121 | 4.19E-06 |
| CDKN1B | EP300-AS1 | 0.3566942 | 5.22E-06 |
| CFLAR | EP300-AS1 | 0.312219741 | 7.65E-05 |
| EEF2K | EP300-AS1 | 0.466058357 | 9.88E-10 |
| EIF2AK3 | EP300-AS1 | 0.367305044 | 2.59E-06 |
| FAS | EP300-AS1 | 0.303101532 | 0.000126272 |
| HDAC1 | EP300-AS1 | 0.4147222 | 8.09E-08 |
| HDAC6 | EP300-AS1 | 0.404944653 | 1.73E-07 |
| IKBKB | EP300-AS1 | 0.301285254 | 0.00013924 |
| IKBKE | EP300-AS1 | 0.549790919 | 1.27E-13 |
| MAPK8 | EP300-AS1 | 0.45312765 | 3.21E-09 |
| MTMR14 | EP300-AS1 | 0.3034461 | 0.000123942 |
| MTOR | EP300-AS1 | 0.327389772 | 3.21E-05 |
| NKX2-3 | EP300-AS1 | 0.449854657 | 4.30E-09 |
| PARP1 | EP300-AS1 | 0.375084043 | 1.52E-06 |
| PIK3R4 | EP300-AS1 | 0.465209266 | 1.07E-09 |
| PRKAB1 | EP300-AS1 | 0.379425487 | 1.12E-06 |
| RAB33B | EP300-AS1 | 0.312542742 | 7.52E-05 |
| RAF1 | EP300-AS1 | 0.462909883 | 1.32E-09 |
| SIRT1 | EP300-AS1 | 0.316304164 | 6.08E-05 |
| TP53 | EP300-AS1 | 0.379455996 | 1.12E-06 |
| TP73 | EP300-AS1 | 0.460285967 | 1.68E-09 |
| TSC2 | EP300-AS1 | 0.418612481 | 5.94E-08 |
| GABARAPL1 | AC005722.3 | 0.407316545 | 1.44E-07 |
| NAMPT | AC005722.3 | 0.498304311 | 4.17E-11 |
| NCKAP1 | AC005722.3 | 0.304138297 | 0.000119383 |
| NFE2L2 | AC005722.3 | 0.359286065 | 4.41E-06 |
| SQSTM1 | AC005722.3 | 0.460151253 | 1.70E-09 |
| TP63 | AC005722.3 | 0.388787284 | 5.75E-07 |
| ATIC | PDCD4-AS1 | 0.324668602 | 3.76E-05 |
| BAK1 | PDCD4-AS1 | -0.328364756 | 3.03E-05 |
| DAPK1 | PDCD4-AS1 | 0.360507574 | 4.07E-06 |
| EEF2 | PDCD4-AS1 | 0.344180371 | 1.16E-05 |
| ITGA3 | PDCD4-AS1 | -0.403436043 | 1.94E-07 |
| NFE2L2 | PDCD4-AS1 | 0.306312764 | 0.00010606 |
| PARP1 | PDCD4-AS1 | 0.32770958 | 3.15E-05 |
| ULK3 | PDCD4-AS1 | 0.320526134 | 4.78E-05 |
| UVRAG | PDCD4-AS1 | 0.391436049 | 4.75E-07 |
| ATG4C | OLMALINC | 0.306965202 | 0.000102342 |
| ATIC | OLMALINC | 0.411310719 | 1.06E-07 |
| BAK1 | OLMALINC | -0.395535701 | 3.51E-07 |
| CASP4 | OLMALINC | -0.323930174 | 3.93E-05 |
| CD46 | OLMALINC | 0.374332825 | 1.60E-06 |
| CDKN1B | OLMALINC | 0.405175701 | 1.70E-07 |
| DIRAS3 | OLMALINC | 0.359828162 | 4.25E-06 |
| EEF2 | OLMALINC | 0.322154014 | 4.35E-05 |
| HDAC6 | OLMALINC | 0.443698001 | 7.37E-09 |
| ITGA3 | OLMALINC | -0.37190574 | 1.89E-06 |
| MAPK8IP1 | OLMALINC | 0.501059675 | 3.13E-11 |
| PARP1 | OLMALINC | 0.378072791 | 1.23E-06 |
| PELP1 | OLMALINC | 0.340974161 | 1.41E-05 |
| PIK3C3 | OLMALINC | 0.35078519 | 7.64E-06 |
| PINK1 | OLMALINC | -0.304937489 | 0.000114315 |
| PRKAR1A | OLMALINC | 0.400409057 | 2.44E-07 |
| PTK6 | OLMALINC | -0.334346551 | 2.12E-05 |
| SIRT1 | OLMALINC | 0.314942307 | 6.57E-05 |
| ST13 | OLMALINC | 0.370868656 | 2.03E-06 |
| ULK2 | OLMALINC | 0.322995791 | 4.15E-05 |
| UVRAG | OLMALINC | 0.303759194 | 0.00012186 |
| VAMP3 | OLMALINC | -0.306183337 | 0.000106813 |
| VAMP7 | OLMALINC | 0.331537063 | 2.51E-05 |
| ATG4D | PAXIP1-AS1 | 0.338060341 | 1.69E-05 |
| BAK1 | PAXIP1-AS1 | -0.413681911 | 8.78E-08 |
| GABARAPL1 | PAXIP1-AS1 | 0.491216006 | 8.61E-11 |
| ITGA3 | PAXIP1-AS1 | -0.513597953 | 8.25E-12 |
| ITGB4 | PAXIP1-AS1 | -0.30853034 | 9.39E-05 |
| NFE2L2 | PAXIP1-AS1 | 0.432559981 | 1.90E-08 |
| RHEB | PAXIP1-AS1 | 0.571305423 | 8.31E-15 |
| ULK3 | PAXIP1-AS1 | 0.326579547 | 3.36E-05 |
| WIPI1 | PAXIP1-AS1 | 0.368855084 | 2.33E-06 |
| ARSB | TRAM2-AS1 | 0.345894051 | 1.04E-05 |
| ATF6 | TRAM2-AS1 | 0.31370948 | 7.04E-05 |
| BIRC6 | TRAM2-AS1 | 0.401888178 | 2.18E-07 |
| CASP4 | TRAM2-AS1 | -0.301414372 | 0.000138279 |
| EIF2AK3 | TRAM2-AS1 | 0.305923489 | 0.000108338 |
| GAA | TRAM2-AS1 | 0.369863498 | 2.17E-06 |
| HDAC6 | TRAM2-AS1 | 0.386553362 | 6.76E-07 |
| KLHL24 | TRAM2-AS1 | 0.390235351 | 5.18E-07 |
| MBTPS2 | TRAM2-AS1 | 0.393933949 | 3.95E-07 |
| NBR1 | TRAM2-AS1 | 0.361469436 | 3.82E-06 |
| PARP1 | TRAM2-AS1 | 0.358961162 | 4.50E-06 |
| PIK3R4 | TRAM2-AS1 | 0.333482721 | 2.23E-05 |
| PRKAB1 | TRAM2-AS1 | 0.31723437 | 5.77E-05 |
| RB1CC1 | TRAM2-AS1 | 0.313223097 | 7.24E-05 |
| ST13 | TRAM2-AS1 | 0.443324076 | 7.61E-09 |
| ULK2 | TRAM2-AS1 | 0.31054311 | 8.40E-05 |
| UVRAG | TRAM2-AS1 | 0.327640969 | 3.16E-05 |
| VAMP7 | TRAM2-AS1 | 0.320462946 | 4.80E-05 |
| AMBRA1 | AC108010.1 | 0.36340514 | 3.36E-06 |
| ARNT | AC108010.1 | 0.481852151 | 2.18E-10 |
| ATF6 | AC108010.1 | 0.327689253 | 3.15E-05 |
| ATG2A | AC108010.1 | 0.309226933 | 9.04E-05 |
| ATG2B | AC108010.1 | 0.351392052 | 7.35E-06 |
| BECN1 | AC108010.1 | 0.414217252 | 8.42E-08 |
| BIRC6 | AC108010.1 | 0.603814933 | 9.05E-17 |
| BNIP1 | AC108010.1 | -0.344418644 | 1.14E-05 |
| CAMKK2 | AC108010.1 | 0.437640873 | 1.24E-08 |
| CD46 | AC108010.1 | 0.360957305 | 3.95E-06 |
| EDEM1 | AC108010.1 | 0.331602588 | 2.50E-05 |
| EEF2K | AC108010.1 | 0.410098814 | 1.16E-07 |
| EIF2AK3 | AC108010.1 | 0.463819667 | 1.22E-09 |
| EIF4G1 | AC108010.1 | 0.383862827 | 8.20E-07 |
| ERN1 | AC108010.1 | 0.384882176 | 7.62E-07 |
| FOXO3 | AC108010.1 | 0.387900751 | 6.14E-07 |
| GABARAPL2 | AC108010.1 | -0.340368221 | 1.47E-05 |
| IKBKB | AC108010.1 | 0.380607815 | 1.03E-06 |
| KIF5B | AC108010.1 | 0.338421076 | 1.65E-05 |
| KLHL24 | AC108010.1 | 0.364978162 | 3.02E-06 |
| MAPK1 | AC108010.1 | 0.421233324 | 4.82E-08 |
| MBTPS2 | AC108010.1 | 0.317366608 | 5.73E-05 |
| MTOR | AC108010.1 | 0.432774836 | 1.87E-08 |
| NAF1 | AC108010.1 | 0.32723828 | 3.24E-05 |
| NBR1 | AC108010.1 | 0.449195413 | 4.56E-09 |
| NCKAP1 | AC108010.1 | 0.404342792 | 1.81E-07 |
| NFE2L2 | AC108010.1 | 0.362865812 | 3.48E-06 |
| NFKB1 | AC108010.1 | 0.312778224 | 7.42E-05 |
| PARP1 | AC108010.1 | 0.367414975 | 2.57E-06 |
| PIK3R4 | AC108010.1 | 0.516849226 | 5.79E-12 |
| RB1CC1 | AC108010.1 | 0.493726128 | 6.67E-11 |
| RPS6KB1 | AC108010.1 | 0.307972497 | 9.68E-05 |
| SIRT1 | AC108010.1 | 0.357129004 | 5.08E-06 |
| SPNS1 | AC108010.1 | 0.455691981 | 2.55E-09 |
| TM9SF1 | AC108010.1 | 0.326983684 | 3.29E-05 |
| TP63 | AC108010.1 | 0.345410579 | 1.07E-05 |
| TSC1 | AC108010.1 | 0.447651399 | 5.22E-09 |
| UVRAG | AC108010.1 | 0.354547437 | 6.00E-06 |
| WDFY3 | AC108010.1 | 0.459255721 | 1.85E-09 |
| CHMP2B | AC093904.3 | 0.401825294 | 2.19E-07 |
| RPS6KB1 | AC093904.3 | 0.39919314 | 2.67E-07 |
| BCL2 | AC124283.2 | 0.309973699 | 8.67E-05 |
| BECN1 | AC124283.2 | 0.314785262 | 6.63E-05 |
| CD46 | AC124283.2 | 0.317029343 | 5.84E-05 |
| FAS | AC124283.2 | 0.312637468 | 7.48E-05 |
| HDAC6 | AC124283.2 | 0.418923808 | 5.80E-08 |
| IKBKB | AC124283.2 | 0.348073687 | 9.08E-06 |
| MAPK8 | AC124283.2 | 0.441665288 | 8.78E-09 |
| NKX2-3 | AC124283.2 | 0.445175252 | 6.48E-09 |
| PARP1 | AC124283.2 | 0.382883215 | 8.79E-07 |
| PIK3R4 | AC124283.2 | 0.39652973 | 3.26E-07 |
| PRKAB1 | AC124283.2 | 0.347759823 | 9.26E-06 |
| RAB33B | AC124283.2 | 0.322383747 | 4.30E-05 |
| RAF1 | AC124283.2 | 0.324254313 | 3.85E-05 |
| RPS6KB1 | AC124283.2 | 0.406032647 | 1.59E-07 |
| TP73 | AC124283.2 | 0.357326204 | 5.01E-06 |
| TSC2 | AC124283.2 | 0.336566766 | 1.85E-05 |
| ARSB | LINC00640 | -0.357903905 | 4.83E-06 |
| ATG2A | LINC00640 | -0.320092936 | 4.90E-05 |
| BID | LINC00640 | 0.322628451 | 4.24E-05 |
| CANX | LINC00640 | -0.339357567 | 1.56E-05 |
| EEF2 | LINC00640 | -0.33954706 | 1.54E-05 |
| FOXO1 | LINC00640 | -0.304739581 | 0.000115551 |
| MAPK9 | LINC00640 | -0.312323555 | 7.61E-05 |
| ATG3 | TBILA | 0.448874304 | 4.69E-09 |
| PIK3R4 | TBILA | 0.305449753 | 0.000111173 |
| RAB7A | TBILA | 0.346485198 | 1.00E-05 |
| ATF4 | SREBF2-AS1 | 0.338263124 | 1.67E-05 |
| ATG10 | SREBF2-AS1 | 0.438680578 | 1.13E-08 |
| ATG16L2 | SREBF2-AS1 | 0.321061921 | 4.64E-05 |
| ATG3 | SREBF2-AS1 | 0.322910018 | 4.17E-05 |
| ATG4B | SREBF2-AS1 | 0.40775469 | 1.39E-07 |
| ATG4C | SREBF2-AS1 | 0.421579626 | 4.68E-08 |
| ATG4D | SREBF2-AS1 | 0.401769074 | 2.20E-07 |
| BAX | SREBF2-AS1 | 0.354540601 | 6.01E-06 |
| CAPN10 | SREBF2-AS1 | 0.466760261 | 9.25E-10 |
| CASP3 | SREBF2-AS1 | 0.344076523 | 1.17E-05 |
| CDKN1B | SREBF2-AS1 | 0.418076944 | 6.20E-08 |
| CDKN2A | SREBF2-AS1 | 0.444694053 | 6.76E-09 |
| CX3CL1 | SREBF2-AS1 | 0.368837818 | 2.33E-06 |
| CXCR4 | SREBF2-AS1 | 0.453414341 | 3.13E-09 |
| DAPK2 | SREBF2-AS1 | 0.384059241 | 8.08E-07 |
| EEF2K | SREBF2-AS1 | 0.31508818 | 6.52E-05 |
| GABARAP | SREBF2-AS1 | 0.305642805 | 0.000110009 |
| HDAC6 | SREBF2-AS1 | 0.573983995 | 5.84E-15 |
| IKBKB | SREBF2-AS1 | 0.312297376 | 7.62E-05 |
| IKBKE | SREBF2-AS1 | 0.396657189 | 3.23E-07 |
| ITGA3 | SREBF2-AS1 | -0.341575769 | 1.36E-05 |
| ITGA6 | SREBF2-AS1 | -0.343523624 | 1.21E-05 |
| MAP2K7 | SREBF2-AS1 | 0.446087318 | 5.98E-09 |
| MTMR14 | SREBF2-AS1 | 0.373603993 | 1.68E-06 |
| NKX2-3 | SREBF2-AS1 | 0.356005199 | 5.46E-06 |
| PARP1 | SREBF2-AS1 | 0.318876936 | 5.26E-05 |
| PINK1 | SREBF2-AS1 | -0.337178741 | 1.79E-05 |
| PRKAB1 | SREBF2-AS1 | 0.408314516 | 1.33E-07 |
| PTK6 | SREBF2-AS1 | -0.330345223 | 2.69E-05 |
| RAB24 | SREBF2-AS1 | 0.336385869 | 1.87E-05 |
| RAF1 | SREBF2-AS1 | 0.402783864 | 2.04E-07 |
| RHEB | SREBF2-AS1 | 0.323108114 | 4.12E-05 |
| STK11 | SREBF2-AS1 | 0.403897545 | 1.87E-07 |
| TP53 | SREBF2-AS1 | 0.400378654 | 2.44E-07 |
| TP73 | SREBF2-AS1 | 0.488722826 | 1.11E-10 |
| TSC2 | SREBF2-AS1 | 0.487770431 | 1.22E-10 |
| ULK3 | SREBF2-AS1 | 0.406046018 | 1.59E-07 |
| WDR45 | SREBF2-AS1 | 0.45434457 | 2.88E-09 |
| DAPK1 | ESRG | 0.426772998 | 3.07E-08 |
| DNAJB9 | ESRG | 0.438925051 | 1.11E-08 |
| UVRAG | ESRG | 0.328579479 | 2.99E-05 |
| CTSB | AC025857.2 | 0.658564974 | 1.24E-20 |
| EIF2AK3 | AF165147.1 | 0.318497885 | 5.37E-05 |
| FOXO3 | AF165147.1 | 0.301595034 | 0.000136944 |
| GABARAPL1 | AF165147.1 | 0.410859284 | 1.10E-07 |
| NFE2L2 | AF165147.1 | 0.559898356 | 3.62E-14 |
| RHEB | AF165147.1 | 0.327867187 | 3.12E-05 |
| TP63 | AF165147.1 | 0.519608261 | 4.27E-12 |
| UVRAG | AF165147.1 | 0.348254096 | 8.98E-06 |
| VAMP3 | AF165147.1 | -0.311702833 | 7.88E-05 |
| WIPI1 | AF165147.1 | 0.350993162 | 7.54E-06 |
| ARSB | AL121944.1 | -0.309681413 | 8.81E-05 |
| ATG9B | AL121944.1 | 0.321677008 | 4.48E-05 |
| FKBP1B | AL121944.1 | 0.304662319 | 0.000116037 |
| FOXO1 | AL121944.1 | -0.393218366 | 4.16E-07 |
| HSP90AB1 | AL121944.1 | 0.341113418 | 1.40E-05 |
| HSPA5 | AL121944.1 | 0.368419599 | 2.40E-06 |
| HSPA8 | AL121944.1 | 0.328044784 | 3.09E-05 |
| MYC | AL121944.1 | 0.3049045 | 0.00011452 |
| ITGA3 | WAC-AS1 | -0.333509716 | 2.23E-05 |
| KIF5B | WAC-AS1 | 0.365900989 | 2.84E-06 |
| RHEB | WAC-AS1 | 0.317987096 | 5.53E-05 |
| ULK3 | WAC-AS1 | 0.350932562 | 7.57E-06 |
| ARNT | HCG11 | 0.347231314 | 9.58E-06 |
| BIRC6 | HCG11 | 0.351539916 | 7.28E-06 |
| CAMKK2 | HCG11 | 0.321279357 | 4.58E-05 |
| EEF2K | HCG11 | 0.319864031 | 4.97E-05 |
| HDAC6 | HCG11 | 0.34474166 | 1.12E-05 |
| MAPK8 | HCG11 | 0.314869556 | 6.60E-05 |
| NAF1 | HCG11 | 0.391535799 | 4.71E-07 |
| PARP1 | HCG11 | 0.378547598 | 1.19E-06 |
| PRKAB1 | HCG11 | 0.328031537 | 3.09E-05 |
| RAB33B | HCG11 | 0.359670856 | 4.30E-06 |
| SIRT1 | HCG11 | 0.372299867 | 1.84E-06 |
| ATG10 | AC006449.6 | 0.334041695 | 2.16E-05 |
| ATG16L2 | AC006449.6 | 0.440776249 | 9.48E-09 |
| ATG4B | AC006449.6 | 0.440911017 | 9.37E-09 |
| ATG4C | AC006449.6 | 0.366247365 | 2.78E-06 |
| ATG4D | AC006449.6 | 0.382315822 | 9.15E-07 |
| ATG7 | AC006449.6 | 0.308097004 | 9.62E-05 |
| ATG9B | AC006449.6 | 0.412271291 | 9.81E-08 |
| ATIC | AC006449.6 | 0.327021381 | 3.28E-05 |
| BAX | AC006449.6 | 0.358641266 | 4.60E-06 |
| BID | AC006449.6 | 0.407729554 | 1.40E-07 |
| BIRC5 | AC006449.6 | 0.308677247 | 9.32E-05 |
| CAPN10 | AC006449.6 | 0.57830065 | 3.28E-15 |
| CDKN1B | AC006449.6 | 0.335966001 | 1.92E-05 |
| CDKN2A | AC006449.6 | 0.324319266 | 3.84E-05 |
| CLN3 | AC006449.6 | 0.396533891 | 3.26E-07 |
| GABARAPL2 | AC006449.6 | 0.373239106 | 1.73E-06 |
| IKBKE | AC006449.6 | 0.303105146 | 0.000126247 |
| ITGA3 | AC006449.6 | -0.416444517 | 7.06E-08 |
| ITGA6 | AC006449.6 | -0.375566866 | 1.47E-06 |
| ITGB1 | AC006449.6 | -0.318465047 | 5.38E-05 |
| MAP1LC3A | AC006449.6 | 0.419334907 | 5.61E-08 |
| MAP2K7 | AC006449.6 | 0.324448161 | 3.81E-05 |
| MAPK8 | AC006449.6 | 0.329647759 | 2.81E-05 |
| MLST8 | AC006449.6 | 0.374777361 | 1.55E-06 |
| MTMR14 | AC006449.6 | 0.521583726 | 3.43E-12 |
| PINK1 | AC006449.6 | -0.360772018 | 4.00E-06 |
| PRKAB1 | AC006449.6 | 0.357606222 | 4.92E-06 |
| RAB24 | AC006449.6 | 0.32117097 | 4.61E-05 |
| RAF1 | AC006449.6 | 0.438462275 | 1.16E-08 |
| STK11 | AC006449.6 | 0.407545829 | 1.42E-07 |
| ULK3 | AC006449.6 | 0.466616364 | 9.38E-10 |
| WDR45 | AC006449.6 | 0.33615986 | 1.90E-05 |
| ATG2B | UNC5B-AS1 | -0.32706884 | 3.27E-05 |
| ATG4D | UNC5B-AS1 | 0.366497215 | 2.73E-06 |
| CDKN2A | UNC5B-AS1 | 0.392417303 | 4.42E-07 |
| ATG3 | PARD3-AS1 | 0.386183826 | 6.94E-07 |
| FOXO3 | PARD3-AS1 | 0.313292692 | 7.21E-05 |
| NFE2L2 | PARD3-AS1 | 0.342863373 | 1.26E-05 |
| PINK1 | PARD3-AS1 | -0.352198066 | 6.98E-06 |
| ATG16L2 | AC004148.2 | 0.426920594 | 3.03E-08 |
| ATG4B | AC004148.2 | 0.427589935 | 2.87E-08 |
| CAPN10 | AC004148.2 | 0.4439824 | 7.19E-09 |
| CXCR4 | AC004148.2 | 0.397853877 | 2.95E-07 |
| DAPK2 | AC004148.2 | 0.516870512 | 5.77E-12 |
| GABARAP | AC004148.2 | 0.364965022 | 3.03E-06 |
| HDAC6 | AC004148.2 | 0.399123002 | 2.69E-07 |
| IKBKB | AC004148.2 | 0.35351047 | 6.42E-06 |
| MAP2K7 | AC004148.2 | 0.316151291 | 6.14E-05 |
| PELP1 | AC004148.2 | 0.554084566 | 7.50E-14 |
| PINK1 | AC004148.2 | -0.333800717 | 2.19E-05 |
| STK11 | AC004148.2 | 0.303649776 | 0.000122584 |
| TSC2 | AC004148.2 | 0.329853937 | 2.77E-05 |
| ULK3 | AC004148.2 | 0.398975301 | 2.72E-07 |
| WDR45 | AC004148.2 | 0.381450202 | 9.73E-07 |
| ATG10 | AC022007.1 | 0.337534705 | 1.75E-05 |
| ATG3 | AC022007.1 | 0.394223753 | 3.87E-07 |
| ATG4A | AC022007.1 | 0.400671507 | 2.39E-07 |
| ATG4B | AC022007.1 | 0.357282849 | 5.03E-06 |
| ATG4C | AC022007.1 | 0.383295847 | 8.54E-07 |
| ATG7 | AC022007.1 | 0.326012688 | 3.48E-05 |
| ATIC | AC022007.1 | 0.418415971 | 6.04E-08 |
| BAG3 | AC022007.1 | -0.342296163 | 1.30E-05 |
| BAX | AC022007.1 | 0.520770061 | 3.75E-12 |
| BIRC5 | AC022007.1 | 0.630793361 | 1.41E-18 |
| BNIP1 | AC022007.1 | 0.472029841 | 5.63E-10 |
| CAPN10 | AC022007.1 | 0.416965927 | 6.78E-08 |
| CDKN2A | AC022007.1 | 0.374158521 | 1.62E-06 |
| CXCR4 | AC022007.1 | 0.420450076 | 5.13E-08 |
| FKBP1A | AC022007.1 | 0.362374735 | 3.60E-06 |
| HDAC6 | AC022007.1 | 0.47777735 | 3.25E-10 |
| ITGA6 | AC022007.1 | -0.326351324 | 3.41E-05 |
| ITGB4 | AC022007.1 | -0.320460091 | 4.80E-05 |
| MAP2K7 | AC022007.1 | 0.324604939 | 3.78E-05 |
| MAPK1 | AC022007.1 | -0.320698681 | 4.74E-05 |
| MAPK8IP1 | AC022007.1 | 0.52572185 | 2.16E-12 |
| MTMR14 | AC022007.1 | 0.554265598 | 7.33E-14 |
| NCKAP1 | AC022007.1 | -0.315708395 | 6.29E-05 |
| PEX14 | AC022007.1 | 0.336973863 | 1.81E-05 |
| PRKAR1A | AC022007.1 | 0.308397373 | 9.46E-05 |
| RAB24 | AC022007.1 | 0.364234299 | 3.18E-06 |
| RAF1 | AC022007.1 | 0.464794316 | 1.11E-09 |
| STK11 | AC022007.1 | 0.449568533 | 4.41E-09 |
| TP53 | AC022007.1 | 0.305789008 | 0.000109136 |
| ULK3 | AC022007.1 | 0.32706285 | 3.27E-05 |
| WDR45 | AC022007.1 | 0.485509276 | 1.52E-10 |
| BIRC6 | SNHG4 | 0.31098625 | 8.20E-05 |
| CAMKK2 | SNHG4 | 0.398076914 | 2.90E-07 |
| CANX | SNHG4 | 0.301550635 | 0.000137271 |
| CAPN10 | SNHG4 | 0.300698672 | 0.000143687 |
| CDKN1A | SNHG4 | -0.32754646 | 3.18E-05 |
| CDKN1B | SNHG4 | 0.315608918 | 6.33E-05 |
| EEF2 | SNHG4 | 0.390209417 | 5.19E-07 |
| HGS | SNHG4 | 0.322273931 | 4.32E-05 |
| IKBKB | SNHG4 | 0.329238466 | 2.88E-05 |
| MAP2K7 | SNHG4 | 0.307200442 | 0.000101032 |
| MAPK8 | SNHG4 | 0.397594131 | 3.01E-07 |
| MAPK9 | SNHG4 | 0.373145926 | 1.74E-06 |
| PARP1 | SNHG4 | 0.35794904 | 4.81E-06 |
| PIK3C3 | SNHG4 | 0.32713791 | 3.26E-05 |
| SIRT1 | SNHG4 | 0.35444792 | 6.04E-06 |
| SPNS1 | SNHG4 | 0.331739366 | 2.48E-05 |
| SQSTM1 | SNHG4 | 0.368103449 | 2.45E-06 |
| ULK3 | SNHG4 | 0.358555415 | 4.63E-06 |
| UVRAG | SNHG4 | 0.445845571 | 6.11E-09 |
| ARNT | AP001505.1 | -0.387090345 | 6.50E-07 |
| ATF4 | AP001505.1 | 0.338543063 | 1.64E-05 |
| ATG2B | AP001505.1 | -0.379004043 | 1.16E-06 |
| ATG4B | AP001505.1 | 0.526839814 | 1.90E-12 |
| BAG1 | AP001505.1 | 0.330960372 | 2.60E-05 |
| BAX | AP001505.1 | 0.492078503 | 7.89E-11 |
| BIRC5 | AP001505.1 | 0.455990894 | 2.49E-09 |
| BIRC6 | AP001505.1 | -0.351920051 | 7.11E-06 |
| BNIP1 | AP001505.1 | 0.481545287 | 2.25E-10 |
| CAPN10 | AP001505.1 | 0.361821572 | 3.73E-06 |
| CLN3 | AP001505.1 | 0.302958509 | 0.00012725 |
| DAPK2 | AP001505.1 | 0.361114532 | 3.91E-06 |
| DLC1 | AP001505.1 | -0.314788631 | 6.63E-05 |
| ERN1 | AP001505.1 | -0.300910697 | 0.000142065 |
| FOXO1 | AP001505.1 | -0.338712942 | 1.63E-05 |
| GOPC | AP001505.1 | -0.308024149 | 9.66E-05 |
| MAPK1 | AP001505.1 | -0.386712223 | 6.68E-07 |
| MLST8 | AP001505.1 | 0.362782585 | 3.50E-06 |
| NCKAP1 | AP001505.1 | -0.366900619 | 2.66E-06 |
| PELP1 | AP001505.1 | 0.443428509 | 7.54E-09 |
| PINK1 | AP001505.1 | -0.30202141 | 0.000133841 |
| STK11 | AP001505.1 | 0.381149162 | 9.94E-07 |
| USP10 | AP001505.1 | -0.306654363 | 0.000104098 |
| WDFY3 | AP001505.1 | -0.309528983 | 8.89E-05 |
| WDR45 | AP001505.1 | 0.330289626 | 2.70E-05 |
| ZFYVE1 | AP001505.1 | -0.319990361 | 4.93E-05 |
| ARNT | HOXC13-AS | -0.315032582 | 6.54E-05 |
| BIRC6 | HOXC13-AS | -0.387730105 | 6.21E-07 |
| CASP3 | HOXC13-AS | -0.368106486 | 2.45E-06 |
| CDKN1B | HOXC13-AS | -0.330233932 | 2.71E-05 |
| EEF2K | HOXC13-AS | -0.305970072 | 0.000108063 |
| EIF2AK3 | HOXC13-AS | -0.439417323 | 1.06E-08 |
| HDAC6 | HOXC13-AS | -0.338649036 | 1.63E-05 |
| ITPR1 | HOXC13-AS | -0.30063413 | 0.000144184 |
| MAP2K7 | HOXC13-AS | -0.351164201 | 7.46E-06 |
| MAPK8 | HOXC13-AS | -0.313553934 | 7.10E-05 |
| NAF1 | HOXC13-AS | -0.30443694 | 0.000117465 |
| NBR1 | HOXC13-AS | -0.342557148 | 1.28E-05 |
| NFE2L2 | HOXC13-AS | -0.324293545 | 3.85E-05 |
| PARP1 | HOXC13-AS | -0.397964864 | 2.93E-07 |
| PINK1 | HOXC13-AS | 0.385925602 | 7.07E-07 |
| PRKAB1 | HOXC13-AS | -0.372190051 | 1.85E-06 |
| RAF1 | HOXC13-AS | -0.318870855 | 5.26E-05 |
| RPS6KB1 | HOXC13-AS | -0.380598061 | 1.03E-06 |
| ATIC | DLEU2 | 0.338156102 | 1.68E-05 |
| BIRC5 | DLEU2 | 0.381621176 | 9.61E-07 |
| CHMP4B | DLEU2 | 0.430220136 | 2.31E-08 |
| MAPK8IP1 | DLEU2 | 0.308239081 | 9.54E-05 |
| MAPK9 | DLEU2 | 0.36191065 | 3.71E-06 |
| PTK6 | DLEU2 | -0.301190227 | 0.000139952 |
| SERPINA1 | AP001783.1 | 0.358367924 | 4.68E-06 |
| ATG9B | SMILR | 0.380143354 | 1.07E-06 |
| CAPN2 | SMILR | 0.345472019 | 1.07E-05 |
| PTK6 | SMILR | 0.331088368 | 2.58E-05 |
| RAB11A | SMILR | 0.336708657 | 1.84E-05 |
| RELA | SMILR | 0.320649031 | 4.75E-05 |
| TP53INP2 | SMILR | 0.363224495 | 3.40E-06 |
| ATG10 | AL604028.1 | 0.366564462 | 2.72E-06 |
| ATG4B | AL604028.1 | 0.392081831 | 4.53E-07 |
| ATG4C | AL604028.1 | 0.361002699 | 3.94E-06 |
| BAX | AL604028.1 | 0.363024757 | 3.44E-06 |
| BID | AL604028.1 | 0.336460931 | 1.86E-05 |
| CAPN10 | AL604028.1 | 0.503227654 | 2.50E-11 |
| CLN3 | AL604028.1 | 0.303532608 | 0.000123364 |
| GABARAP | AL604028.1 | 0.342896595 | 1.26E-05 |
| HDAC1 | AL604028.1 | 0.37984503 | 1.09E-06 |
| HDAC6 | AL604028.1 | 0.37049831 | 2.08E-06 |
| ITGA6 | AL604028.1 | -0.308581872 | 9.36E-05 |
| MTMR14 | AL604028.1 | 0.316888757 | 5.89E-05 |
| NRG1 | AL604028.1 | -0.320833624 | 4.70E-05 |
| PEX14 | AL604028.1 | 0.349457895 | 8.32E-06 |
| RAB24 | AL604028.1 | 0.318168626 | 5.47E-05 |
| TP63 | AL604028.1 | -0.302758869 | 0.000128629 |
| ULK3 | AL604028.1 | 0.399876556 | 2.54E-07 |
| WDR45 | AL604028.1 | 0.345465065 | 1.07E-05 |
| ATG4D | PTCSC2 | 0.415641858 | 7.53E-08 |
| ATG9B | PTCSC2 | 0.432475257 | 1.91E-08 |
| BAK1 | PTCSC2 | -0.350233368 | 7.92E-06 |
| BID | PTCSC2 | 0.429185563 | 2.52E-08 |
| EIF2AK3 | PTCSC2 | 0.384339509 | 7.92E-07 |
| GABARAPL1 | PTCSC2 | 0.338133849 | 1.68E-05 |
| ITGA3 | PTCSC2 | -0.369251446 | 2.27E-06 |
| NFE2L2 | PTCSC2 | 0.467564988 | 8.58E-10 |
| PINK1 | PTCSC2 | -0.30019447 | 0.000147615 |
| PRKAB1 | PTCSC2 | 0.31310442 | 7.29E-05 |
| TP53INP2 | PTCSC2 | 0.475542182 | 4.03E-10 |
| TP63 | PTCSC2 | 0.304582359 | 0.000116542 |
| BID | KCNMB2-AS1 | 0.312610943 | 7.49E-05 |
| EIF2AK3 | KCNMB2-AS1 | 0.315170517 | 6.49E-05 |
| GABARAPL1 | KCNMB2-AS1 | 0.359470695 | 4.36E-06 |
| ITGA3 | KCNMB2-AS1 | -0.305560278 | 0.000110505 |
| KLHL24 | KCNMB2-AS1 | 0.481013644 | 2.37E-10 |
| NFE2L2 | KCNMB2-AS1 | 0.374045952 | 1.63E-06 |
| ARNT | LINC02057 | 0.304872485 | 0.00011472 |
| ATG3 | LINC02057 | 0.479315848 | 2.80E-10 |
| CD46 | LINC02057 | 0.329966134 | 2.75E-05 |
| EEF2K | LINC02057 | 0.379753506 | 1.10E-06 |
| EIF2AK3 | LINC02057 | 0.439655985 | 1.04E-08 |
| FOXO3 | LINC02057 | 0.422128768 | 4.48E-08 |
| HDAC6 | LINC02057 | 0.371980525 | 1.88E-06 |
| IKBKE | LINC02057 | 0.310529072 | 8.41E-05 |
| KLHL24 | LINC02057 | 0.345743704 | 1.05E-05 |
| MAP2K7 | LINC02057 | 0.302088801 | 0.000133357 |
| NFE2L2 | LINC02057 | 0.398376925 | 2.84E-07 |
| PIK3R4 | LINC02057 | 0.5555433 | 6.25E-14 |
| PINK1 | LINC02057 | -0.312910753 | 7.36E-05 |
| PRKAB1 | LINC02057 | 0.426746549 | 3.08E-08 |
| RAB33B | LINC02057 | 0.325511289 | 3.58E-05 |
| RAF1 | LINC02057 | 0.346341708 | 1.01E-05 |
| RB1CC1 | LINC02057 | 0.341333464 | 1.38E-05 |
| TP63 | LINC02057 | 0.35807749 | 4.77E-06 |
| TP73 | LINC02057 | 0.309580627 | 8.86E-05 |
| TSC1 | LINC02057 | 0.462180173 | 1.41E-09 |
| ATF6 | AC097468.3 | -0.312173646 | 7.67E-05 |
| ATG10 | AC097468.3 | 0.324347468 | 3.83E-05 |
| ATG2B | AC097468.3 | -0.315273961 | 6.45E-05 |
| ATG4B | AC097468.3 | 0.613578623 | 2.10E-17 |
| ATG4D | AC097468.3 | 0.321846621 | 4.43E-05 |
| ATIC | AC097468.3 | 0.302722215 | 0.000128883 |
| BAX | AC097468.3 | 0.455074196 | 2.70E-09 |
| BIRC6 | AC097468.3 | -0.368820494 | 2.33E-06 |
| BNIP1 | AC097468.3 | 0.558651152 | 4.24E-14 |
| CAPN10 | AC097468.3 | 0.532650536 | 9.76E-13 |
| CAPNS1 | AC097468.3 | 0.415985623 | 7.32E-08 |
| CDKN2A | AC097468.3 | 0.471019428 | 6.20E-10 |
| CHMP4B | AC097468.3 | 0.335126536 | 2.02E-05 |
| CLN3 | AC097468.3 | 0.329960307 | 2.76E-05 |
| DAPK2 | AC097468.3 | 0.324141768 | 3.88E-05 |
| EIF4G1 | AC097468.3 | -0.399827801 | 2.55E-07 |
| FKBP1A | AC097468.3 | 0.435278734 | 1.51E-08 |
| GABARAP | AC097468.3 | 0.35799022 | 4.80E-06 |
| HIF1A | AC097468.3 | -0.366285264 | 2.77E-06 |
| KIF5B | AC097468.3 | -0.368913672 | 2.32E-06 |
| MAP1LC3A | AC097468.3 | 0.499241512 | 3.79E-11 |
| MAPK1 | AC097468.3 | -0.327228684 | 3.24E-05 |
| MBTPS2 | AC097468.3 | -0.324703813 | 3.75E-05 |
| MLST8 | AC097468.3 | 0.378242424 | 1.22E-06 |
| MTMR14 | AC097468.3 | 0.36428735 | 3.17E-06 |
| NCKAP1 | AC097468.3 | -0.444830931 | 6.68E-09 |
| NKX2-3 | AC097468.3 | 0.364367205 | 3.15E-06 |
| NPC1 | AC097468.3 | -0.336219705 | 1.89E-05 |
| PEX14 | AC097468.3 | 0.463012288 | 1.31E-09 |
| RAB24 | AC097468.3 | 0.372392196 | 1.83E-06 |
| STK11 | AC097468.3 | 0.461362445 | 1.52E-09 |
| TP63 | AC097468.3 | -0.421890122 | 4.57E-08 |
| TP73 | AC097468.3 | 0.34963482 | 8.22E-06 |
| USP10 | AC097468.3 | -0.415530703 | 7.59E-08 |
| WDFY3 | AC097468.3 | -0.374465087 | 1.59E-06 |
| WDR45 | AC097468.3 | 0.426596766 | 3.11E-08 |
| ATF4 | AL023284.4 | 0.471006348 | 6.21E-10 |
| ATG2B | AL023284.4 | -0.35702634 | 5.11E-06 |
| ATG4B | AL023284.4 | 0.449829496 | 4.31E-09 |
| BAX | AL023284.4 | 0.314746601 | 6.64E-05 |
| BIRC6 | AL023284.4 | -0.357009015 | 5.12E-06 |
| BNIP1 | AL023284.4 | 0.404677992 | 1.76E-07 |
| CANX | AL023284.4 | -0.3477914 | 9.24E-06 |
| CAPN10 | AL023284.4 | 0.418566796 | 5.96E-08 |
| DAPK2 | AL023284.4 | 0.416402369 | 7.09E-08 |
| DDIT3 | AL023284.4 | 0.343693579 | 1.20E-05 |
| EIF4G1 | AL023284.4 | -0.445404194 | 6.35E-09 |
| GABARAP | AL023284.4 | 0.349938484 | 8.07E-06 |
| MTOR | AL023284.4 | -0.302025631 | 0.000133811 |
| PELP1 | AL023284.4 | 0.360771973 | 4.00E-06 |
| PIK3R4 | AL023284.4 | -0.300934324 | 0.000141885 |
| USP10 | AL023284.4 | -0.376073345 | 1.42E-06 |
| FOXO1 | AC019080.4 | 0.307271536 | 0.000100639 |
| GABARAPL1 | AC019080.4 | 0.358028701 | 4.79E-06 |
| NCKAP1 | AC019080.4 | 0.327271754 | 3.23E-05 |
| NFE2L2 | AC019080.4 | 0.428309096 | 2.71E-08 |
| PIK3C3 | AC019080.4 | 0.308498107 | 9.41E-05 |
| RHEB | AC019080.4 | 0.314014029 | 6.92E-05 |
| TP63 | AC019080.4 | 0.377118019 | 1.32E-06 |
| ATG9B | SBF2-AS1 | 0.312397642 | 7.58E-05 |
| EIF2AK3 | SBF2-AS1 | 0.345551714 | 1.06E-05 |
| NFE2L2 | SBF2-AS1 | 0.35071718 | 7.68E-06 |
| VAMP3 | SBF2-AS1 | -0.308357 | 9.48E-05 |
| WIPI1 | SBF2-AS1 | 0.303598688 | 0.000122923 |
| ATF4 | MAPKAPK5-AS1 | 0.510149635 | 1.20E-11 |
| ATG16L2 | MAPKAPK5-AS1 | 0.367728276 | 2.51E-06 |
| ATG2B | MAPKAPK5-AS1 | -0.330336132 | 2.69E-05 |
| ATG4B | MAPKAPK5-AS1 | 0.5323515 | 1.01E-12 |
| ATG4D | MAPKAPK5-AS1 | 0.353278699 | 6.52E-06 |
| ATIC | MAPKAPK5-AS1 | 0.315953715 | 6.21E-05 |
| BAX | MAPKAPK5-AS1 | 0.458524457 | 1.98E-09 |
| BID | MAPKAPK5-AS1 | 0.333714324 | 2.20E-05 |
| BIRC5 | MAPKAPK5-AS1 | 0.432888109 | 1.85E-08 |
| BNIP1 | MAPKAPK5-AS1 | 0.571442958 | 8.16E-15 |
| CAPN10 | MAPKAPK5-AS1 | 0.547193029 | 1.75E-13 |
| CAPN2 | MAPKAPK5-AS1 | -0.394845966 | 3.69E-07 |
| CLN3 | MAPKAPK5-AS1 | 0.342670464 | 1.27E-05 |
| DAPK2 | MAPKAPK5-AS1 | 0.303455927 | 0.000123876 |
| DDIT3 | MAPKAPK5-AS1 | 0.424615844 | 3.66E-08 |
| EIF4G1 | MAPKAPK5-AS1 | -0.351922069 | 7.11E-06 |
| FKBP1A | MAPKAPK5-AS1 | 0.393887399 | 3.96E-07 |
| GABARAP | MAPKAPK5-AS1 | 0.305421484 | 0.000111344 |
| GABARAPL2 | MAPKAPK5-AS1 | 0.340251801 | 1.48E-05 |
| ITGA3 | MAPKAPK5-AS1 | -0.319768788 | 4.99E-05 |
| MAP1LC3A | MAPKAPK5-AS1 | 0.443637172 | 7.40E-09 |
| MAPK1 | MAPKAPK5-AS1 | -0.379058355 | 1.15E-06 |
| MLST8 | MAPKAPK5-AS1 | 0.346716249 | 9.89E-06 |
| PELP1 | MAPKAPK5-AS1 | 0.451900174 | 3.58E-09 |
| PINK1 | MAPKAPK5-AS1 | -0.35582842 | 5.53E-06 |
| STK11 | MAPKAPK5-AS1 | 0.300411911 | 0.000145909 |
| WDFY3 | MAPKAPK5-AS1 | -0.37917626 | 1.14E-06 |
| WDR45 | MAPKAPK5-AS1 | 0.524877511 | 2.37E-12 |
| ZFYVE1 | MAPKAPK5-AS1 | -0.333000871 | 2.30E-05 |
| ARNT | ANKRD10-IT1 | 0.345290221 | 1.08E-05 |
| ARSB | ANKRD10-IT1 | 0.379667136 | 1.10E-06 |
| BCL2 | ANKRD10-IT1 | 0.317867143 | 5.57E-05 |
| CD46 | ANKRD10-IT1 | 0.359950298 | 4.22E-06 |
| CDKN1A | ANKRD10-IT1 | -0.318642013 | 5.33E-05 |
| CDKN1B | ANKRD10-IT1 | 0.425898509 | 3.30E-08 |
| CX3CL1 | ANKRD10-IT1 | 0.326657379 | 3.35E-05 |
| CXCR4 | ANKRD10-IT1 | 0.527995289 | 1.67E-12 |
| DRAM1 | ANKRD10-IT1 | 0.336680941 | 1.84E-05 |
| EEF2K | ANKRD10-IT1 | 0.40916452 | 1.25E-07 |
| FAS | ANKRD10-IT1 | 0.394892062 | 3.68E-07 |
| GOPC | ANKRD10-IT1 | 0.344424839 | 1.14E-05 |
| IKBKB | ANKRD10-IT1 | 0.312737066 | 7.44E-05 |
| KLHL24 | ANKRD10-IT1 | 0.331166476 | 2.56E-05 |
| MAPK8 | ANKRD10-IT1 | 0.389261048 | 5.56E-07 |
| MAPK9 | ANKRD10-IT1 | 0.342202611 | 1.31E-05 |
| NBR1 | ANKRD10-IT1 | 0.369694022 | 2.20E-06 |
| NKX2-3 | ANKRD10-IT1 | 0.344654857 | 1.13E-05 |
| PARP1 | ANKRD10-IT1 | 0.418430824 | 6.03E-08 |
| PTK6 | ANKRD10-IT1 | -0.341702158 | 1.35E-05 |
| RAB33B | ANKRD10-IT1 | 0.347086438 | 9.66E-06 |
| SIRT1 | ANKRD10-IT1 | 0.528897085 | 1.50E-12 |
| SQSTM1 | ANKRD10-IT1 | 0.328029319 | 3.09E-05 |
| TP53 | ANKRD10-IT1 | 0.326119903 | 3.46E-05 |
| TP73 | ANKRD10-IT1 | 0.37456107 | 1.58E-06 |
| TSC2 | ANKRD10-IT1 | 0.429496898 | 2.45E-08 |
| ULK2 | ANKRD10-IT1 | 0.373202687 | 1.73E-06 |
| UVRAG | ANKRD10-IT1 | 0.454603719 | 2.82E-09 |
| ATG4C | AC012306.2 | -0.326914168 | 3.30E-05 |
| FAS | AC012306.2 | -0.354071102 | 6.19E-06 |
| ARSB | AC009779.2 | 0.318064372 | 5.51E-05 |
| ATG4C | AC009779.2 | 0.322755345 | 4.21E-05 |
| BCL2 | AC009779.2 | 0.319919861 | 4.95E-05 |
| CAPN10 | AC009779.2 | 0.318683591 | 5.31E-05 |
| CD46 | AC009779.2 | 0.309393019 | 8.95E-05 |
| CDKN1A | AC009779.2 | -0.316307331 | 6.08E-05 |
| CDKN1B | AC009779.2 | 0.421741855 | 4.62E-08 |
| CX3CL1 | AC009779.2 | 0.390215638 | 5.19E-07 |
| CXCR4 | AC009779.2 | 0.547764786 | 1.63E-13 |
| FAS | AC009779.2 | 0.347789369 | 9.25E-06 |
| HDAC6 | AC009779.2 | 0.370677091 | 2.06E-06 |
| ITGA6 | AC009779.2 | -0.301862067 | 0.000134993 |
| KLHL24 | AC009779.2 | 0.340276652 | 1.48E-05 |
| NBR1 | AC009779.2 | 0.36072038 | 4.01E-06 |
| PARP1 | AC009779.2 | 0.404801256 | 1.75E-07 |
| PINK1 | AC009779.2 | -0.323006993 | 4.14E-05 |
| PTK6 | AC009779.2 | -0.354019748 | 6.21E-06 |
| SIRT1 | AC009779.2 | 0.307304496 | 0.000100457 |
| SQSTM1 | AC009779.2 | 0.333846428 | 2.18E-05 |
| TP53 | AC009779.2 | 0.384102262 | 8.06E-07 |
| TSC2 | AC009779.2 | 0.353720319 | 6.33E-06 |
| ULK2 | AC009779.2 | 0.302917449 | 0.000127533 |
| UVRAG | AC009779.2 | 0.384103563 | 8.06E-07 |
| WDR45 | AC009779.2 | 0.335200605 | 2.01E-05 |
| CAMKK2 | BOLA3-AS1 | 0.300745402 | 0.000143328 |
| CTSD | BOLA3-AS1 | -0.382219323 | 9.21E-07 |
| DIRAS3 | BOLA3-AS1 | 0.387821171 | 6.17E-07 |
| FKBP1B | BOLA3-AS1 | 0.545708718 | 2.09E-13 |
| FOXO1 | BOLA3-AS1 | -0.328735944 | 2.96E-05 |
| NAF1 | BOLA3-AS1 | 0.330707095 | 2.64E-05 |
| ATF4 | AC024060.1 | 0.387459362 | 6.33E-07 |
| ATG10 | AC024060.1 | 0.408129947 | 1.35E-07 |
| ATG12 | AC024060.1 | 0.322022465 | 4.39E-05 |
| ATG16L2 | AC024060.1 | 0.453368979 | 3.14E-09 |
| ATG3 | AC024060.1 | 0.385007758 | 7.55E-07 |
| ATG4B | AC024060.1 | 0.482642979 | 2.02E-10 |
| ATG4C | AC024060.1 | 0.445881582 | 6.09E-09 |
| ATG4D | AC024060.1 | 0.323915357 | 3.93E-05 |
| ATG7 | AC024060.1 | 0.354866427 | 5.88E-06 |
| ATIC | AC024060.1 | 0.388271222 | 5.97E-07 |
| BAG3 | AC024060.1 | -0.389186691 | 5.59E-07 |
| BAX | AC024060.1 | 0.543634149 | 2.68E-13 |
| BIRC5 | AC024060.1 | 0.464412903 | 1.15E-09 |
| BNIP1 | AC024060.1 | 0.478959624 | 2.90E-10 |
| CAPN10 | AC024060.1 | 0.640055508 | 3.07E-19 |
| CDKN1B | AC024060.1 | 0.308561083 | 9.38E-05 |
| CDKN2A | AC024060.1 | 0.38306658 | 8.68E-07 |
| CLN3 | AC024060.1 | 0.329645625 | 2.81E-05 |
| CXCR4 | AC024060.1 | 0.510419943 | 1.16E-11 |
| DAPK2 | AC024060.1 | 0.347187213 | 9.60E-06 |
| EIF4G1 | AC024060.1 | -0.357713526 | 4.89E-06 |
| FKBP1A | AC024060.1 | 0.425382295 | 3.44E-08 |
| GABARAP | AC024060.1 | 0.412119125 | 9.93E-08 |
| HDAC6 | AC024060.1 | 0.410650571 | 1.11E-07 |
| HIF1A | AC024060.1 | -0.370265045 | 2.12E-06 |
| IKBKB | AC024060.1 | 0.351837789 | 7.15E-06 |
| ITGA6 | AC024060.1 | -0.346942518 | 9.75E-06 |
| ITGB1 | AC024060.1 | -0.303853989 | 0.000121237 |
| LAMP2 | AC024060.1 | -0.313954097 | 6.95E-05 |
| MAP1LC3A | AC024060.1 | 0.343915379 | 1.18E-05 |
| MAP2K7 | AC024060.1 | 0.370330959 | 2.11E-06 |
| MAPK1 | AC024060.1 | -0.357294419 | 5.02E-06 |
| MAPK8IP1 | AC024060.1 | 0.446243415 | 5.90E-09 |
| MLST8 | AC024060.1 | 0.331013584 | 2.59E-05 |
| MTMR14 | AC024060.1 | 0.590832176 | 5.85E-16 |
| NCKAP1 | AC024060.1 | -0.394253533 | 3.86E-07 |
| PELP1 | AC024060.1 | 0.336591823 | 1.85E-05 |
| PEX14 | AC024060.1 | 0.379109091 | 1.15E-06 |
| PINK1 | AC024060.1 | -0.319571564 | 5.05E-05 |
| RAB24 | AC024060.1 | 0.456362384 | 2.40E-09 |
| RAF1 | AC024060.1 | 0.45686466 | 2.30E-09 |
| STK11 | AC024060.1 | 0.4519567 | 3.57E-09 |
| TP53 | AC024060.1 | 0.307485996 | 9.95E-05 |
| TP63 | AC024060.1 | -0.324238241 | 3.86E-05 |
| TSC2 | AC024060.1 | 0.425351086 | 3.45E-08 |
| ULK3 | AC024060.1 | 0.438854666 | 1.12E-08 |
| USP10 | AC024060.1 | -0.3184933 | 5.37E-05 |
| WDFY3 | AC024060.1 | -0.35363317 | 6.37E-06 |
| WDR45 | AC024060.1 | 0.527840767 | 1.69E-12 |
| ZFYVE1 | AC024060.1 | -0.31474737 | 6.64E-05 |
| ATG4D | LINC02454 | -0.363650868 | 3.30E-06 |
| BCL2 | LINC02454 | -0.313311778 | 7.20E-05 |
| CAPN10 | LINC02454 | -0.30162939 | 0.000136691 |
| CDKN1B | LINC02454 | -0.376238938 | 1.40E-06 |
| CDKN2A | LINC02454 | -0.332672366 | 2.34E-05 |
| CLN3 | LINC02454 | -0.383464493 | 8.43E-07 |
| EIF2S1 | LINC02454 | 0.452035867 | 3.54E-09 |
| GNAI3 | LINC02454 | 0.318970219 | 5.23E-05 |
| HDAC6 | LINC02454 | -0.413487127 | 8.92E-08 |
| IKBKB | LINC02454 | -0.312850769 | 7.39E-05 |
| IKBKE | LINC02454 | -0.371453683 | 1.95E-06 |
| IL24 | LINC02454 | 0.331825658 | 2.47E-05 |
| ITGA3 | LINC02454 | 0.523226904 | 2.85E-12 |
| ITGA6 | LINC02454 | 0.484704214 | 1.65E-10 |
| ITGB1 | LINC02454 | 0.449237085 | 4.54E-09 |
| ITGB4 | LINC02454 | 0.356226627 | 5.38E-06 |
| KLHL24 | LINC02454 | -0.328451273 | 3.01E-05 |
| MAP2K7 | LINC02454 | -0.322544783 | 4.26E-05 |
| NFE2L2 | LINC02454 | -0.300415443 | 0.000145881 |
| NRG1 | LINC02454 | 0.343591845 | 1.20E-05 |
| PINK1 | LINC02454 | 0.342845102 | 1.26E-05 |
| PRKAB1 | LINC02454 | -0.311171145 | 8.11E-05 |
| RAF1 | LINC02454 | -0.308607589 | 9.35E-05 |
| SAR1A | LINC02454 | 0.329498889 | 2.83E-05 |
| TP53 | LINC02454 | -0.310831786 | 8.27E-05 |
| ULK3 | LINC02454 | -0.384319042 | 7.94E-07 |
| WDR45 | LINC02454 | -0.337723512 | 1.73E-05 |
| ATG4D | AC087392.1 | 0.428702084 | 2.62E-08 |
| ATG7 | AC087392.1 | 0.453644691 | 3.07E-09 |
| BNIP1 | AC087392.1 | 0.452511829 | 3.39E-09 |
| CAPNS1 | AC087392.1 | 0.43424724 | 1.65E-08 |
| CDKN2A | AC087392.1 | 0.325771259 | 3.53E-05 |
| FKBP1A | AC087392.1 | 0.373819741 | 1.66E-06 |
| IKBKE | AC087392.1 | 0.392961052 | 4.24E-07 |
| MAP1LC3A | AC087392.1 | 0.401686618 | 2.21E-07 |
| MAPK8 | AC087392.1 | 0.375476356 | 1.48E-06 |
| MTMR14 | AC087392.1 | 0.332577124 | 2.36E-05 |
| NKX2-3 | AC087392.1 | 0.494189581 | 6.37E-11 |
| PRKAB1 | AC087392.1 | 0.447165937 | 5.44E-09 |
| RAC1 | AC087392.1 | 0.467257208 | 8.83E-10 |
| TSC2 | AC087392.1 | 0.427372356 | 2.92E-08 |
| WDR45 | AC087392.1 | 0.372446988 | 1.82E-06 |
| AMBRA1 | AC068768.1 | 0.35540152 | 5.68E-06 |
| ARNT | AC068768.1 | 0.352249851 | 6.96E-06 |
| ARSB | AC068768.1 | 0.403661943 | 1.91E-07 |
| ATG2B | AC068768.1 | 0.427068706 | 3.00E-08 |
| BECN1 | AC068768.1 | 0.349576772 | 8.26E-06 |
| BIRC6 | AC068768.1 | 0.55163213 | 1.01E-13 |
| CAMKK2 | AC068768.1 | 0.559334412 | 3.89E-14 |
| DAPK1 | AC068768.1 | 0.428812134 | 2.60E-08 |
| EDEM1 | AC068768.1 | 0.375651969 | 1.46E-06 |
| EIF2AK3 | AC068768.1 | 0.387794381 | 6.18E-07 |
| FOXO3 | AC068768.1 | 0.403500985 | 1.93E-07 |
| ITPR1 | AC068768.1 | 0.310638998 | 8.36E-05 |
| KIF5B | AC068768.1 | 0.31620785 | 6.12E-05 |
| KLHL24 | AC068768.1 | 0.329120043 | 2.90E-05 |
| MAPK1 | AC068768.1 | 0.440659652 | 9.57E-09 |
| MBTPS2 | AC068768.1 | 0.370001472 | 2.15E-06 |
| MTOR | AC068768.1 | 0.373692671 | 1.67E-06 |
| NAF1 | AC068768.1 | 0.36373046 | 3.29E-06 |
| NBR1 | AC068768.1 | 0.457574708 | 2.15E-09 |
| NCKAP1 | AC068768.1 | 0.393293166 | 4.14E-07 |
| PARP1 | AC068768.1 | 0.333185873 | 2.27E-05 |
| PIK3R4 | AC068768.1 | 0.343825845 | 1.19E-05 |
| PRKAB1 | AC068768.1 | 0.385717455 | 7.18E-07 |
| RAB33B | AC068768.1 | 0.318799029 | 5.28E-05 |
| RB1CC1 | AC068768.1 | 0.454144809 | 2.93E-09 |
| SIRT1 | AC068768.1 | 0.371976203 | 1.88E-06 |
| UVRAG | AC068768.1 | 0.456983309 | 2.27E-09 |
| WDFY3 | AC068768.1 | 0.413198235 | 9.12E-08 |
| ATG16L2 | RAB30-AS1 | 0.325891417 | 3.50E-05 |
| DIRAS3 | RAB30-AS1 | 0.32608034 | 3.46E-05 |
| FOXO1 | RAB30-AS1 | -0.309354157 | 8.97E-05 |
| MAPK1 | RAB30-AS1 | -0.301373527 | 0.000138582 |
| MLST8 | RAB30-AS1 | 0.302678907 | 0.000129184 |
| SH3GLB1 | RAB30-AS1 | -0.306534103 | 0.000104785 |
| WDR45 | RAB30-AS1 | 0.350118511 | 7.98E-06 |
| ATG10 | PTOV1-AS1 | 0.397834807 | 2.96E-07 |
| ATG12 | PTOV1-AS1 | 0.399953023 | 2.52E-07 |
| ATG3 | PTOV1-AS1 | 0.371161311 | 1.99E-06 |
| ATG4A | PTOV1-AS1 | 0.397265909 | 3.09E-07 |
| ATG4B | PTOV1-AS1 | 0.446090316 | 5.98E-09 |
| ATG4C | PTOV1-AS1 | 0.506485966 | 1.77E-11 |
| ATG7 | PTOV1-AS1 | 0.404766641 | 1.75E-07 |
| BAX | PTOV1-AS1 | 0.463383833 | 1.27E-09 |
| BCL2 | PTOV1-AS1 | 0.383339825 | 8.51E-07 |
| BIRC5 | PTOV1-AS1 | 0.382674234 | 8.92E-07 |
| BNIP1 | PTOV1-AS1 | 0.431551573 | 2.07E-08 |
| CALCOCO2 | PTOV1-AS1 | 0.337716064 | 1.73E-05 |
| CAPN10 | PTOV1-AS1 | 0.423480807 | 4.02E-08 |
| CAPNS1 | PTOV1-AS1 | 0.358235962 | 4.72E-06 |
| CDKN1B | PTOV1-AS1 | 0.343333223 | 1.22E-05 |
| CDKN2A | PTOV1-AS1 | 0.450701761 | 3.99E-09 |
| CXCR4 | PTOV1-AS1 | 0.376226866 | 1.40E-06 |
| EIF4G1 | PTOV1-AS1 | -0.322982896 | 4.15E-05 |
| FAS | PTOV1-AS1 | 0.41748474 | 6.50E-08 |
| HDAC6 | PTOV1-AS1 | 0.460375953 | 1.67E-09 |
| HIF1A | PTOV1-AS1 | -0.338001595 | 1.70E-05 |
| IKBKE | PTOV1-AS1 | 0.353425362 | 6.45E-06 |
| ITGA6 | PTOV1-AS1 | -0.32102638 | 4.65E-05 |
| MAP1LC3A | PTOV1-AS1 | 0.354097129 | 6.18E-06 |
| MAPK8 | PTOV1-AS1 | 0.395233142 | 3.59E-07 |
| MAPK9 | PTOV1-AS1 | 0.35646108 | 5.30E-06 |
| MTMR14 | PTOV1-AS1 | 0.4257328 | 3.34E-08 |
| NCKAP1 | PTOV1-AS1 | -0.376170883 | 1.41E-06 |
| NKX2-3 | PTOV1-AS1 | 0.455265391 | 2.65E-09 |
| PEX14 | PTOV1-AS1 | 0.555502076 | 6.28E-14 |
| PRKAB1 | PTOV1-AS1 | 0.368686928 | 2.35E-06 |
| RAB24 | PTOV1-AS1 | 0.335180447 | 2.02E-05 |
| RAF1 | PTOV1-AS1 | 0.391489283 | 4.73E-07 |
| STK11 | PTOV1-AS1 | 0.48846383 | 1.14E-10 |
| TP53 | PTOV1-AS1 | 0.315054045 | 6.53E-05 |
| TP63 | PTOV1-AS1 | -0.310741487 | 8.31E-05 |
| TP73 | PTOV1-AS1 | 0.474190412 | 4.59E-10 |
| TSC2 | PTOV1-AS1 | 0.437260125 | 1.28E-08 |
| ULK3 | PTOV1-AS1 | 0.316957461 | 5.86E-05 |
| USP10 | PTOV1-AS1 | -0.349719038 | 8.18E-06 |
| WDR45 | PTOV1-AS1 | 0.522924116 | 2.95E-12 |
| ATG10 | CKMT2-AS1 | 0.540583286 | 3.86E-13 |
| ATG12 | CKMT2-AS1 | 0.498930906 | 3.91E-11 |
| ATG4A | CKMT2-AS1 | 0.374029947 | 1.63E-06 |
| ATG4C | CKMT2-AS1 | 0.48325356 | 1.90E-10 |
| ATIC | CKMT2-AS1 | 0.306991647 | 0.000102194 |
| BCL2 | CKMT2-AS1 | 0.425169663 | 3.50E-08 |
| CAPN10 | CKMT2-AS1 | 0.37346583 | 1.70E-06 |
| CDKN1B | CKMT2-AS1 | 0.441969273 | 8.55E-09 |
| CDKN2A | CKMT2-AS1 | 0.317967491 | 5.54E-05 |
| CXCR4 | CKMT2-AS1 | 0.368276433 | 2.42E-06 |
| FAS | CKMT2-AS1 | 0.310592757 | 8.38E-05 |
| HDAC6 | CKMT2-AS1 | 0.527752585 | 1.71E-12 |
| MAP2K7 | CKMT2-AS1 | 0.447366394 | 5.35E-09 |
| MAPK8 | CKMT2-AS1 | 0.38856312 | 5.85E-07 |
| MAPK9 | CKMT2-AS1 | 0.317102336 | 5.82E-05 |
| MTMR14 | CKMT2-AS1 | 0.367275764 | 2.59E-06 |
| NKX2-3 | CKMT2-AS1 | 0.303882777 | 0.000121048 |
| PEX14 | CKMT2-AS1 | 0.334176263 | 2.14E-05 |
| PRKAB1 | CKMT2-AS1 | 0.307913559 | 9.72E-05 |
| PTK6 | CKMT2-AS1 | -0.312388455 | 7.58E-05 |
| RAF1 | CKMT2-AS1 | 0.425623294 | 3.37E-08 |
| SIRT1 | CKMT2-AS1 | 0.310928194 | 8.23E-05 |
| STK11 | CKMT2-AS1 | 0.317277005 | 5.76E-05 |
| TP53 | CKMT2-AS1 | 0.376066098 | 1.42E-06 |
| TSC2 | CKMT2-AS1 | 0.356035479 | 5.45E-06 |
| ULK2 | CKMT2-AS1 | 0.300163061 | 0.000147863 |
| ULK3 | CKMT2-AS1 | 0.350550016 | 7.76E-06 |
| WDR45 | CKMT2-AS1 | 0.311698296 | 7.88E-05 |
| BECN1 | ATP1B3-AS1 | 0.315895003 | 6.23E-05 |
| EGFR | ATP1B3-AS1 | 0.319265972 | 5.14E-05 |
| EIF2AK3 | ATP1B3-AS1 | 0.312311604 | 7.62E-05 |
| KLHL24 | ATP1B3-AS1 | 0.314788026 | 6.63E-05 |
| PIK3R4 | ATP1B3-AS1 | 0.359397341 | 4.38E-06 |
| TSC1 | ATP1B3-AS1 | 0.315332177 | 6.43E-05 |
| ATG4B | AC103591.3 | 0.352019866 | 7.06E-06 |
| BAX | AC103591.3 | 0.33988484 | 1.51E-05 |
| BID | AC103591.3 | 0.301247696 | 0.000139521 |
| BIRC6 | AC103591.3 | -0.344140443 | 1.16E-05 |
| BNIP1 | AC103591.3 | 0.361768298 | 3.74E-06 |
| CAPN10 | AC103591.3 | 0.372910608 | 1.76E-06 |
| CDKN2A | AC103591.3 | 0.328714801 | 2.97E-05 |
| CHMP4B | AC103591.3 | 0.548845301 | 1.43E-13 |
| GABARAPL2 | AC103591.3 | 0.341234013 | 1.39E-05 |
| MAP1LC3A | AC103591.3 | 0.474095464 | 4.63E-10 |
| MAPK1 | AC103591.3 | -0.320660581 | 4.75E-05 |
| MTMR14 | AC103591.3 | 0.307099429 | 0.000101593 |
| PEX14 | AC103591.3 | 0.35265599 | 6.78E-06 |
| RAB24 | AC103591.3 | 0.374406564 | 1.59E-06 |
| STK11 | AC103591.3 | 0.404986388 | 1.72E-07 |
| WDR45 | AC103591.3 | 0.355901881 | 5.50E-06 |
| CFLAR | AL691432.2 | 0.413392388 | 8.99E-08 |
| SIRT2 | AL691432.2 | 0.398085294 | 2.90E-07 |
| VAMP3 | AL691432.2 | 0.49663738 | 4.95E-11 |
| ATG3 | LINC01133 | 0.307990115 | 9.67E-05 |
| ATG4D | LINC01133 | 0.377448365 | 1.29E-06 |
| DAPK2 | LINC01133 | 0.360590737 | 4.05E-06 |
| EIF2AK3 | LINC01133 | 0.30964139 | 8.83E-05 |
| ITGA3 | LINC01133 | -0.352280731 | 6.95E-06 |
| NFE2L2 | LINC01133 | 0.418725473 | 5.89E-08 |
| PELP1 | LINC01133 | 0.405254893 | 1.69E-07 |
| PTEN | LINC01133 | -0.32679408 | 3.32E-05 |
| BAK1 | AP002748.3 | -0.369647483 | 2.21E-06 |
| CDKN1A | AP002748.3 | -0.310099694 | 8.61E-05 |
| GABARAPL1 | AP002748.3 | 0.419603504 | 5.49E-08 |
| ITGA3 | AP002748.3 | -0.316163382 | 6.13E-05 |
| PRKAB1 | AP002748.3 | 0.324422993 | 3.82E-05 |
| RELA | AP002748.3 | 0.411144067 | 1.07E-07 |
| ST13 | AP002748.3 | 0.304740678 | 0.000115544 |
| ATG12 | AL139287.1 | 0.306423919 | 0.000105418 |
| ATG16L2 | AL139287.1 | 0.537473452 | 5.57E-13 |
| ATG4B | AL139287.1 | 0.444779115 | 6.71E-09 |
| CAPN10 | AL139287.1 | 0.434691313 | 1.59E-08 |
| CFLAR | AL139287.1 | 0.328681293 | 2.97E-05 |
| HDAC1 | AL139287.1 | 0.310764428 | 8.30E-05 |
| IKBKB | AL139287.1 | 0.32349986 | 4.03E-05 |
| PEX14 | AL139287.1 | 0.50280945 | 2.61E-11 |
| RAB24 | AL139287.1 | 0.429683886 | 2.41E-08 |
| STK11 | AL139287.1 | 0.387872698 | 6.15E-07 |
| ULK3 | AL139287.1 | 0.365590648 | 2.90E-06 |
| WDR45 | AL139287.1 | 0.324968107 | 3.70E-05 |
| ATG2B | BBOX1-AS1 | -0.309427705 | 8.94E-05 |
| ATG4D | BBOX1-AS1 | 0.334969722 | 2.04E-05 |
| BID | BBOX1-AS1 | 0.358642777 | 4.60E-06 |
| BNIP1 | BBOX1-AS1 | 0.322805125 | 4.19E-05 |
| CDKN2A | BBOX1-AS1 | 0.31404411 | 6.91E-05 |
| CTSD | BBOX1-AS1 | 0.327472253 | 3.19E-05 |
| IKBKE | BBOX1-AS1 | 0.337199765 | 1.78E-05 |
| MAP1LC3A | BBOX1-AS1 | 0.368775417 | 2.34E-06 |
| NAF1 | BBOX1-AS1 | -0.302505102 | 0.0001304 |
| RAC1 | BBOX1-AS1 | 0.462539933 | 1.37E-09 |
| WDR45 | BBOX1-AS1 | 0.321471523 | 4.53E-05 |
| ITGA3 | AC010280.1 | -0.302386114 | 0.000131239 |
| KLHL24 | AC010280.1 | 0.359478624 | 4.35E-06 |
| NAMPT | AC010280.1 | 0.350709762 | 7.68E-06 |
| NFE2L2 | AC010280.1 | 0.460705663 | 1.62E-09 |
| RHEB | AC010280.1 | 0.369035375 | 2.30E-06 |
| TP63 | AC010280.1 | 0.360834519 | 3.98E-06 |
| ATG16L2 | AL162274.2 | 0.33199177 | 2.44E-05 |
| ATG4B | AL162274.2 | 0.407449563 | 1.43E-07 |
| BAX | AL162274.2 | 0.340273184 | 1.48E-05 |
| BNIP1 | AL162274.2 | 0.36430328 | 3.16E-06 |
| CAPN10 | AL162274.2 | 0.465591809 | 1.03E-09 |
| CXCR4 | AL162274.2 | 0.306450148 | 0.000105267 |
| KIF5B | AL162274.2 | -0.338574761 | 1.64E-05 |
| MAPK1 | AL162274.2 | -0.319711223 | 5.01E-05 |
| PEX14 | AL162274.2 | 0.315464185 | 6.38E-05 |
| RAB24 | AL162274.2 | 0.383004248 | 8.71E-07 |
| STK11 | AL162274.2 | 0.383773613 | 8.25E-07 |
| VEGFA | AL162274.2 | 0.302765943 | 0.000128579 |
| WDR45 | AL162274.2 | 0.418411493 | 6.04E-08 |
| AMBRA1 | AC097713.1 | 0.328222401 | 3.05E-05 |
| ARSB | AC097713.1 | 0.302433283 | 0.000130906 |
| BCL2 | AC097713.1 | 0.345781813 | 1.05E-05 |
| CXCR4 | AC097713.1 | 0.469045146 | 7.47E-10 |
| DAPK1 | AC097713.1 | 0.347229542 | 9.58E-06 |
| DNAJB1 | AC097713.1 | 0.301498203 | 0.000137658 |
| EEF2 | AC097713.1 | 0.373605439 | 1.68E-06 |
| HDAC6 | AC097713.1 | 0.374096046 | 1.63E-06 |
| ITPR1 | AC097713.1 | 0.319207898 | 5.16E-05 |
| KLHL24 | AC097713.1 | 0.413993992 | 8.57E-08 |
| PARP1 | AC097713.1 | 0.354873786 | 5.88E-06 |
| TP53 | AC097713.1 | 0.343561936 | 1.21E-05 |
| TP73 | AC097713.1 | 0.361704798 | 3.76E-06 |
| TSC2 | AC097713.1 | 0.321755411 | 4.46E-05 |
| ULK2 | AC097713.1 | 0.321212637 | 4.60E-05 |
| GABARAPL1 | AC011287.1 | 0.412767736 | 9.44E-08 |
| GNAI3 | AC011287.1 | -0.329836836 | 2.78E-05 |
| LAMP2 | AC011287.1 | 0.333068751 | 2.29E-05 |
| MBTPS2 | AC011287.1 | 0.315059402 | 6.53E-05 |
| NAMPT | AC011287.1 | 0.397357223 | 3.07E-07 |
| NBR1 | AC011287.1 | 0.357374078 | 5.00E-06 |
| NCKAP1 | AC011287.1 | 0.421164322 | 4.84E-08 |
| ULK1 | AC011287.1 | 0.30747501 | 9.95E-05 |
| WIPI1 | AC011287.1 | 0.325355705 | 3.61E-05 |
| AMBRA1 | AC007406.5 | 0.311474313 | 7.98E-05 |
| ARNT | AC007406.5 | 0.374335911 | 1.60E-06 |
| ARSB | AC007406.5 | 0.442068653 | 8.48E-09 |
| ATF6 | AC007406.5 | 0.338824737 | 1.61E-05 |
| ATG4C | AC007406.5 | 0.317273557 | 5.76E-05 |
| ATG4D | AC007406.5 | 0.301478312 | 0.000137805 |
| BAK1 | AC007406.5 | -0.36162917 | 3.78E-06 |
| BCL2 | AC007406.5 | 0.49849823 | 4.09E-11 |
| BIRC6 | AC007406.5 | 0.439901207 | 1.02E-08 |
| BNIP3 | AC007406.5 | -0.310355744 | 8.49E-05 |
| CAMKK2 | AC007406.5 | 0.341990283 | 1.33E-05 |
| CASP3 | AC007406.5 | 0.375920522 | 1.43E-06 |
| CASP4 | AC007406.5 | -0.358079637 | 4.77E-06 |
| CD46 | AC007406.5 | 0.422808774 | 4.24E-08 |
| CDKN1A | AC007406.5 | -0.350606668 | 7.73E-06 |
| CDKN1B | AC007406.5 | 0.602606276 | 1.08E-16 |
| CXCR4 | AC007406.5 | 0.423117798 | 4.14E-08 |
| DRAM1 | AC007406.5 | 0.389976958 | 5.28E-07 |
| EEF2 | AC007406.5 | 0.383837332 | 8.21E-07 |
| EEF2K | AC007406.5 | 0.378597857 | 1.19E-06 |
| EIF2AK3 | AC007406.5 | 0.381339129 | 9.81E-07 |
| FOXO3 | AC007406.5 | 0.305605137 | 0.000110235 |
| GABARAPL1 | AC007406.5 | 0.422317169 | 4.41E-08 |
| HDAC6 | AC007406.5 | 0.423339951 | 4.06E-08 |
| IKBKB | AC007406.5 | 0.463355588 | 1.27E-09 |
| IKBKE | AC007406.5 | 0.397687589 | 2.99E-07 |
| ITGA3 | AC007406.5 | -0.316674151 | 5.96E-05 |
| ITPR1 | AC007406.5 | 0.36810733 | 2.45E-06 |
| KLHL24 | AC007406.5 | 0.44414247 | 7.09E-09 |
| MAP2K7 | AC007406.5 | 0.505908785 | 1.88E-11 |
| MAPK8 | AC007406.5 | 0.454901071 | 2.74E-09 |
| MAPK9 | AC007406.5 | 0.367379287 | 2.57E-06 |
| NBR1 | AC007406.5 | 0.484209314 | 1.73E-10 |
| NKX2-3 | AC007406.5 | 0.415613927 | 7.54E-08 |
| PARP1 | AC007406.5 | 0.465653869 | 1.03E-09 |
| PIK3C3 | AC007406.5 | 0.404072882 | 1.85E-07 |
| PIK3R4 | AC007406.5 | 0.31236184 | 7.59E-05 |
| PRKAB1 | AC007406.5 | 0.346562518 | 9.99E-06 |
| PTK6 | AC007406.5 | -0.429911208 | 2.37E-08 |
| RAF1 | AC007406.5 | 0.34671651 | 9.89E-06 |
| RB1CC1 | AC007406.5 | 0.327033936 | 3.28E-05 |
| RHEB | AC007406.5 | 0.31201069 | 7.74E-05 |
| SIRT1 | AC007406.5 | 0.34321853 | 1.23E-05 |
| TP53 | AC007406.5 | 0.471049191 | 6.18E-10 |
| TSC1 | AC007406.5 | 0.350971528 | 7.55E-06 |
| TSC2 | AC007406.5 | 0.437859128 | 1.22E-08 |
| ULK2 | AC007406.5 | 0.34651456 | 1.00E-05 |
| UVRAG | AC007406.5 | 0.474103757 | 4.62E-10 |
| ATF4 | CRNDE | 0.375272877 | 1.50E-06 |
| ATG3 | CRNDE | 0.350952123 | 7.56E-06 |
| ATG4A | CRNDE | 0.301434686 | 0.000138128 |
| ATG4B | CRNDE | 0.35891381 | 4.52E-06 |
| ATIC | CRNDE | 0.302241614 | 0.000132264 |
| BAX | CRNDE | 0.460808531 | 1.60E-09 |
| BIRC5 | CRNDE | 0.391179724 | 4.84E-07 |
| BNIP1 | CRNDE | 0.32414882 | 3.88E-05 |
| CAPN10 | CRNDE | 0.436828785 | 1.33E-08 |
| CHMP4B | CRNDE | 0.325148819 | 3.66E-05 |
| CLN3 | CRNDE | 0.302317748 | 0.000131723 |
| FKBP1A | CRNDE | 0.304840451 | 0.000114919 |
| GABARAPL2 | CRNDE | 0.390327224 | 5.15E-07 |
| HDAC6 | CRNDE | 0.36675411 | 2.68E-06 |
| ITGA3 | CRNDE | -0.316383847 | 6.06E-05 |
| ITGA6 | CRNDE | -0.316424282 | 6.04E-05 |
| ITGB4 | CRNDE | -0.377046934 | 1.33E-06 |
| MAPK1 | CRNDE | -0.352238227 | 6.97E-06 |
| MAPK8IP1 | CRNDE | 0.398721972 | 2.77E-07 |
| RPTOR | CRNDE | -0.324401014 | 3.82E-05 |
| ULK3 | CRNDE | 0.367021714 | 2.64E-06 |
| WDR45 | CRNDE | 0.450656445 | 4.00E-09 |
| ATG12 | AL022328.1 | 0.30567799 | 0.000109799 |
| ATG16L2 | AL022328.1 | 0.307786373 | 9.78E-05 |
| ATG4B | AL022328.1 | 0.407528488 | 1.42E-07 |
| ATG4C | AL022328.1 | 0.318015268 | 5.52E-05 |
| ATG4D | AL022328.1 | 0.330119704 | 2.73E-05 |
| CAPN10 | AL022328.1 | 0.382435393 | 9.07E-07 |
| CASP3 | AL022328.1 | 0.317903483 | 5.56E-05 |
| CD46 | AL022328.1 | 0.310930127 | 8.22E-05 |
| CDKN1B | AL022328.1 | 0.395398047 | 3.55E-07 |
| CDKN2A | AL022328.1 | 0.306145426 | 0.000107034 |
| CXCR4 | AL022328.1 | 0.458076014 | 2.06E-09 |
| DAPK2 | AL022328.1 | 0.339804492 | 1.52E-05 |
| EEF2K | AL022328.1 | 0.312772626 | 7.42E-05 |
| HDAC6 | AL022328.1 | 0.486136757 | 1.43E-10 |
| IKBKB | AL022328.1 | 0.395274061 | 3.58E-07 |
| IKBKE | AL022328.1 | 0.300339492 | 0.000146475 |
| MAP2K7 | AL022328.1 | 0.43228426 | 1.95E-08 |
| MAPK8 | AL022328.1 | 0.303278733 | 0.000125069 |
| NKX2-3 | AL022328.1 | 0.31287991 | 7.38E-05 |
| PIK3C3 | AL022328.1 | 0.342229174 | 1.31E-05 |
| PINK1 | AL022328.1 | -0.419922163 | 5.35E-08 |
| PRKAB1 | AL022328.1 | 0.375851833 | 1.44E-06 |
| PTK6 | AL022328.1 | -0.313558808 | 7.10E-05 |
| RAF1 | AL022328.1 | 0.320882679 | 4.69E-05 |
| STK11 | AL022328.1 | 0.384082994 | 8.07E-07 |
| TP53 | AL022328.1 | 0.307083171 | 0.000101683 |
| TP73 | AL022328.1 | 0.396863909 | 3.18E-07 |
| TSC1 | AL022328.1 | 0.428402365 | 2.68E-08 |
| TSC2 | AL022328.1 | 0.405171452 | 1.70E-07 |
| ULK3 | AL022328.1 | 0.404052219 | 1.85E-07 |
| WDR45 | AL022328.1 | 0.388404596 | 5.92E-07 |
| ATG10 | LINC01006 | 0.452866504 | 3.29E-09 |
| ATG3 | LINC01006 | 0.401717891 | 2.21E-07 |
| ATG4B | LINC01006 | 0.301171819 | 0.00014009 |
| ATG4C | LINC01006 | 0.426349016 | 3.18E-08 |
| ATG4D | LINC01006 | 0.340423848 | 1.46E-05 |
| ATG7 | LINC01006 | 0.363375534 | 3.36E-06 |
| BAG3 | LINC01006 | -0.313588088 | 7.09E-05 |
| BAX | LINC01006 | 0.487420021 | 1.26E-10 |
| BCL2 | LINC01006 | 0.381783378 | 9.50E-07 |
| BIRC5 | LINC01006 | 0.369076598 | 2.29E-06 |
| BNIP1 | LINC01006 | 0.441452016 | 8.94E-09 |
| CAPN10 | LINC01006 | 0.382137425 | 9.27E-07 |
| CAPNS1 | LINC01006 | 0.301303359 | 0.000139105 |
| CASP4 | LINC01006 | -0.342343717 | 1.30E-05 |
| CDKN1B | LINC01006 | 0.394770646 | 3.71E-07 |
| CDKN2A | LINC01006 | 0.409627808 | 1.21E-07 |
| CLN3 | LINC01006 | 0.31565702 | 6.31E-05 |
| CXCR4 | LINC01006 | 0.395916225 | 3.41E-07 |
| GABARAP | LINC01006 | 0.351410685 | 7.34E-06 |
| HDAC6 | LINC01006 | 0.499347125 | 3.75E-11 |
| HIF1A | LINC01006 | -0.304644031 | 0.000116152 |
| IKBKE | LINC01006 | 0.391764567 | 4.63E-07 |
| ITGA6 | LINC01006 | -0.36258791 | 3.55E-06 |
| MAP2K7 | LINC01006 | 0.376039506 | 1.42E-06 |
| MAPK8 | LINC01006 | 0.312029818 | 7.74E-05 |
| MTMR14 | LINC01006 | 0.4815878 | 2.24E-10 |
| NKX2-3 | LINC01006 | 0.397861281 | 2.95E-07 |
| NRG1 | LINC01006 | -0.31726581 | 5.76E-05 |
| PARP1 | LINC01006 | 0.307768159 | 9.79E-05 |
| PRKAB1 | LINC01006 | 0.326022748 | 3.48E-05 |
| PTK6 | LINC01006 | -0.347168471 | 9.61E-06 |
| RAB1A | LINC01006 | -0.3387426 | 1.62E-05 |
| RAF1 | LINC01006 | 0.397360163 | 3.06E-07 |
| STK11 | LINC01006 | 0.330453527 | 2.68E-05 |
| TP53 | LINC01006 | 0.491446238 | 8.41E-11 |
| TP73 | LINC01006 | 0.373950096 | 1.64E-06 |
| TSC2 | LINC01006 | 0.356594139 | 5.26E-06 |
| WDR45 | LINC01006 | 0.408781724 | 1.29E-07 |
| CDKN1B | AC021054.1 | 0.36956161 | 2.22E-06 |
| GABARAPL1 | AC021054.1 | 0.389981743 | 5.28E-07 |
| ATG16L2 | AC110285.2 | 0.304652854 | 0.000116097 |
| ATIC | AC110285.2 | 0.428634662 | 2.63E-08 |
| CAPN10 | AC110285.2 | 0.360209453 | 4.15E-06 |
| DAPK2 | AC110285.2 | 0.4110064 | 1.08E-07 |
| DIRAS3 | AC110285.2 | 0.351508593 | 7.30E-06 |
| GABARAP | AC110285.2 | 0.359193852 | 4.44E-06 |
| MAPK8IP1 | AC110285.2 | 0.469991093 | 6.83E-10 |
| PELP1 | AC110285.2 | 0.526996168 | 1.87E-12 |
| BAK1 | BX293535.1 | -0.341103681 | 1.40E-05 |
| BID | BX293535.1 | 0.325247333 | 3.64E-05 |
| CAPN10 | BX293535.1 | 0.330956582 | 2.60E-05 |
| CLN3 | BX293535.1 | 0.322587519 | 4.25E-05 |
| ITGA3 | BX293535.1 | -0.313582949 | 7.09E-05 |
| MLST8 | BX293535.1 | 0.331825879 | 2.47E-05 |
| NFE2L2 | BX293535.1 | 0.329835946 | 2.78E-05 |
| PINK1 | BX293535.1 | -0.310458162 | 8.44E-05 |
| RHEB | BX293535.1 | 0.341122024 | 1.40E-05 |
| ARSB | AC009948.1 | 0.301408331 | 0.000138324 |
| ATG12 | AC009948.1 | 0.324604044 | 3.78E-05 |
| CASP8 | AC009948.1 | 0.341482096 | 1.37E-05 |
| EIF2AK2 | AC009948.1 | 0.470587219 | 6.46E-10 |
| HDAC6 | AC009948.1 | 0.41269518 | 9.49E-08 |
| KLHL24 | AC009948.1 | 0.36181117 | 3.73E-06 |
| MAP2K7 | AC009948.1 | 0.322108217 | 4.37E-05 |
| PARP1 | AC009948.1 | 0.3093243 | 8.99E-05 |
| SIRT1 | AC009948.1 | 0.377893342 | 1.25E-06 |
| ST13 | AC009948.1 | 0.348305875 | 8.95E-06 |
| ATF4 | AC109347.2 | 0.306545351 | 0.000104721 |
| ATG10 | AC109347.2 | 0.412860133 | 9.37E-08 |
| ATG12 | AC109347.2 | 0.331128032 | 2.57E-05 |
| ATG4B | AC109347.2 | 0.410358252 | 1.14E-07 |
| ATG4C | AC109347.2 | 0.441153304 | 9.17E-09 |
| ATG4D | AC109347.2 | 0.378344325 | 1.21E-06 |
| ATG7 | AC109347.2 | 0.350621393 | 7.72E-06 |
| ATIC | AC109347.2 | 0.342118024 | 1.32E-05 |
| BAX | AC109347.2 | 0.397174435 | 3.11E-07 |
| BIRC5 | AC109347.2 | 0.491595544 | 8.28E-11 |
| BNIP1 | AC109347.2 | 0.455293111 | 2.65E-09 |
| CAPN10 | AC109347.2 | 0.431279067 | 2.12E-08 |
| CAPNS1 | AC109347.2 | 0.334969345 | 2.04E-05 |
| CASP3 | AC109347.2 | 0.304016869 | 0.000120172 |
| CDKN1B | AC109347.2 | 0.35726857 | 5.03E-06 |
| CDKN2A | AC109347.2 | 0.467268999 | 8.82E-10 |
| CHMP4B | AC109347.2 | 0.329244583 | 2.88E-05 |
| DAPK2 | AC109347.2 | 0.338666154 | 1.63E-05 |
| EIF4G1 | AC109347.2 | -0.345733426 | 1.05E-05 |
| FKBP1A | AC109347.2 | 0.333838615 | 2.19E-05 |
| GABARAP | AC109347.2 | 0.444755361 | 6.72E-09 |
| HDAC6 | AC109347.2 | 0.458701352 | 1.94E-09 |
| HIF1A | AC109347.2 | -0.333772763 | 2.19E-05 |
| IKBKE | AC109347.2 | 0.338946543 | 1.60E-05 |
| MAP1LC3A | AC109347.2 | 0.404200187 | 1.83E-07 |
| MAPK8 | AC109347.2 | 0.336300429 | 1.88E-05 |
| MAPK8IP1 | AC109347.2 | 0.336918463 | 1.81E-05 |
| MTMR14 | AC109347.2 | 0.499394593 | 3.73E-11 |
| NCKAP1 | AC109347.2 | -0.354438701 | 6.05E-06 |
| NKX2-3 | AC109347.2 | 0.353818195 | 6.29E-06 |
| PELP1 | AC109347.2 | 0.301717151 | 0.000136048 |
| PEX14 | AC109347.2 | 0.40867559 | 1.30E-07 |
| PINK1 | AC109347.2 | -0.346979667 | 9.73E-06 |
| PRKAB1 | AC109347.2 | 0.343211646 | 1.23E-05 |
| RAB24 | AC109347.2 | 0.33338595 | 2.25E-05 |
| RAF1 | AC109347.2 | 0.355286732 | 5.72E-06 |
| STK11 | AC109347.2 | 0.442149164 | 8.42E-09 |
| TP73 | AC109347.2 | 0.342066486 | 1.32E-05 |
| TSC2 | AC109347.2 | 0.349656421 | 8.21E-06 |
| ULK3 | AC109347.2 | 0.361215349 | 3.88E-06 |
| WDR45 | AC109347.2 | 0.461780369 | 1.47E-09 |
| BIRC6 | AC080013.4 | 0.381186357 | 9.91E-07 |
| CD46 | AC080013.4 | 0.304538697 | 0.000116818 |
| EIF2AK3 | AC080013.4 | 0.423943914 | 3.87E-08 |
| ERN1 | AC080013.4 | 0.301207549 | 0.000139822 |
| HDAC6 | AC080013.4 | 0.391658145 | 4.67E-07 |
| MAPK8IP1 | AC080013.4 | 0.336723027 | 1.84E-05 |
| MBTPS2 | AC080013.4 | 0.314632648 | 6.69E-05 |
| NBR1 | AC080013.4 | 0.32024922 | 4.86E-05 |
| PARP1 | AC080013.4 | 0.320450474 | 4.80E-05 |
| PIK3R4 | AC080013.4 | 0.336311325 | 1.88E-05 |
| PRKAR1A | AC080013.4 | 0.54226694 | 3.16E-13 |
| RPS6KB1 | AC080013.4 | 0.316527759 | 6.01E-05 |
| SIRT1 | AC080013.4 | 0.340969822 | 1.42E-05 |
| CAPN1 | CERS3-AS1 | 0.338536021 | 1.64E-05 |
| RAB11A | CERS3-AS1 | 0.326091119 | 3.46E-05 |
| TP53INP2 | CERS3-AS1 | 0.328061275 | 3.08E-05 |
| BIRC6 | MID1IP1-AS1 | 0.406522783 | 1.53E-07 |
| CAMKK2 | MID1IP1-AS1 | 0.31084224 | 8.26E-05 |
| DAPK1 | MID1IP1-AS1 | 0.326287597 | 3.42E-05 |
| EIF2AK3 | MID1IP1-AS1 | 0.375574287 | 1.47E-06 |
| EIF4G1 | MID1IP1-AS1 | 0.414343634 | 8.34E-08 |
| FOXO3 | MID1IP1-AS1 | 0.32471915 | 3.75E-05 |
| GABARAPL1 | MID1IP1-AS1 | 0.33313454 | 2.28E-05 |
| KIF5B | MID1IP1-AS1 | 0.413792406 | 8.71E-08 |
| KLHL24 | MID1IP1-AS1 | 0.360787673 | 3.99E-06 |
| MAPK1 | MID1IP1-AS1 | 0.307098977 | 0.000101595 |
| MBTPS2 | MID1IP1-AS1 | 0.356500353 | 5.29E-06 |
| NBR1 | MID1IP1-AS1 | 0.343175151 | 1.23E-05 |
| NCKAP1 | MID1IP1-AS1 | 0.347616994 | 9.35E-06 |
| NFE2L2 | MID1IP1-AS1 | 0.348285884 | 8.96E-06 |
| NPC1 | MID1IP1-AS1 | 0.312653885 | 7.47E-05 |
| PEX14 | MID1IP1-AS1 | -0.30182466 | 0.000135265 |
| PIK3R4 | MID1IP1-AS1 | 0.390460131 | 5.10E-07 |
| RB1CC1 | MID1IP1-AS1 | 0.411840603 | 1.01E-07 |
| TP63 | MID1IP1-AS1 | 0.340221996 | 1.48E-05 |
| USP10 | MID1IP1-AS1 | 0.315144796 | 6.50E-05 |
| UVRAG | MID1IP1-AS1 | 0.301454602 | 0.00013798 |
| WDFY3 | MID1IP1-AS1 | 0.314079818 | 6.90E-05 |
| WIPI1 | MID1IP1-AS1 | 0.333666729 | 2.21E-05 |
| ATIC | AC007405.3 | 0.368335262 | 2.41E-06 |
| DIRAS3 | AC007405.3 | 0.627855631 | 2.26E-18 |
| EEF2 | AC007405.3 | 0.35222767 | 6.97E-06 |
| FKBP1B | AC007405.3 | 0.368946115 | 2.31E-06 |
| MAPK8IP1 | AC007405.3 | 0.36241785 | 3.59E-06 |
| ST13 | AC007405.3 | 0.403390108 | 1.95E-07 |
| BAG3 | AC084033.3 | -0.406487701 | 1.54E-07 |
| BAK1 | AC084033.3 | -0.308771099 | 9.27E-05 |
| BAX | AC084033.3 | 0.525210059 | 2.28E-12 |
| BIRC5 | AC084033.3 | 0.363935343 | 3.24E-06 |
| BNIP1 | AC084033.3 | 0.489932245 | 9.80E-11 |
| CAPN1 | AC084033.3 | -0.325428287 | 3.60E-05 |
| CAPN2 | AC084033.3 | -0.370511967 | 2.08E-06 |
| CDKN1A | AC084033.3 | -0.300586146 | 0.000144555 |
| CXCR4 | AC084033.3 | 0.554694787 | 6.95E-14 |
| FKBP1A | AC084033.3 | 0.395544349 | 3.51E-07 |
| GAA | AC084033.3 | 0.333369371 | 2.25E-05 |
| GNAI3 | AC084033.3 | -0.313178752 | 7.25E-05 |
| HDAC6 | AC084033.3 | 0.352402505 | 6.89E-06 |
| ITGA6 | AC084033.3 | -0.364057551 | 3.21E-06 |
| PTK6 | AC084033.3 | -0.403354431 | 1.95E-07 |
| TP73 | AC084033.3 | 0.334016637 | 2.16E-05 |
| TSC2 | AC084033.3 | 0.309804193 | 8.75E-05 |
| WDR45 | AC084033.3 | 0.434214997 | 1.65E-08 |
| AMBRA1 | C1RL-AS1 | 0.382017491 | 9.35E-07 |
| BIRC6 | C1RL-AS1 | 0.370418122 | 2.09E-06 |
| CAMKK2 | C1RL-AS1 | 0.334203882 | 2.14E-05 |
| CDKN1B | C1RL-AS1 | 0.311341712 | 8.04E-05 |
| CXCR4 | C1RL-AS1 | 0.322334155 | 4.31E-05 |
| IKBKB | C1RL-AS1 | 0.427822878 | 2.82E-08 |
| MTOR | C1RL-AS1 | 0.328601353 | 2.99E-05 |
| PIK3R4 | C1RL-AS1 | 0.331174619 | 2.56E-05 |
| SIRT1 | C1RL-AS1 | 0.324559054 | 3.79E-05 |
| SPNS1 | C1RL-AS1 | 0.373379728 | 1.71E-06 |
| TSC1 | C1RL-AS1 | 0.307434942 | 9.97E-05 |
| TSC2 | C1RL-AS1 | 0.324695917 | 3.76E-05 |
| UVRAG | C1RL-AS1 | 0.357408955 | 4.99E-06 |
| FKBP1A | AP003390.1 | 0.315171758 | 6.49E-05 |
| ATG16L2 | AP006284.1 | 0.490224322 | 9.51E-11 |
| BAG1 | AP006284.1 | 0.303051415 | 0.000126614 |
| CAPN10 | AP006284.1 | 0.435701736 | 1.46E-08 |
| CHMP4B | AP006284.1 | 0.424215804 | 3.78E-08 |
| DIRAS3 | AP006284.1 | 0.31830534 | 5.43E-05 |
| FKBP1B | AP006284.1 | 0.332382428 | 2.39E-05 |
| GABARAPL2 | AP006284.1 | 0.330853856 | 2.61E-05 |
| MAP1LC3A | AP006284.1 | 0.420695935 | 5.03E-08 |
| STK11 | AP006284.1 | 0.340271 | 1.48E-05 |
| ULK3 | AP006284.1 | 0.46669833 | 9.31E-10 |
| ATF4 | AL022328.2 | 0.327996739 | 3.10E-05 |
| ATG16L2 | AL022328.2 | 0.471385592 | 5.99E-10 |
| ATG3 | AL022328.2 | 0.312687796 | 7.46E-05 |
| ATG4B | AL022328.2 | 0.462288196 | 1.40E-09 |
| CAPN10 | AL022328.2 | 0.526000297 | 2.09E-12 |
| CDKN1B | AL022328.2 | 0.332829879 | 2.32E-05 |
| CXCR4 | AL022328.2 | 0.398157988 | 2.89E-07 |
| DAPK2 | AL022328.2 | 0.326522413 | 3.38E-05 |
| HDAC6 | AL022328.2 | 0.405734956 | 1.63E-07 |
| IKBKB | AL022328.2 | 0.430346169 | 2.29E-08 |
| MAP2K7 | AL022328.2 | 0.41247287 | 9.66E-08 |
| PINK1 | AL022328.2 | -0.317927132 | 5.55E-05 |
| RAB24 | AL022328.2 | 0.35590428 | 5.50E-06 |
| RAF1 | AL022328.2 | 0.317649894 | 5.64E-05 |
| STK11 | AL022328.2 | 0.443157137 | 7.72E-09 |
| TSC1 | AL022328.2 | 0.381843871 | 9.46E-07 |
| TSC2 | AL022328.2 | 0.373864505 | 1.65E-06 |
| ULK3 | AL022328.2 | 0.478674553 | 2.98E-10 |
| WDR45 | AL022328.2 | 0.431065857 | 2.15E-08 |
| ATF4 | AC012146.1 | 0.450608536 | 4.02E-09 |
| ATG16L2 | AC012146.1 | 0.305682238 | 0.000109773 |
| ATG2B | AC012146.1 | -0.307597225 | 9.89E-05 |
| ATG4B | AC012146.1 | 0.465060741 | 1.08E-09 |
| ATIC | AC012146.1 | 0.469098703 | 7.43E-10 |
| BAG1 | AC012146.1 | 0.301126928 | 0.000140428 |
| BAG3 | AC012146.1 | -0.426928305 | 3.03E-08 |
| BAX | AC012146.1 | 0.493165062 | 7.06E-11 |
| BIRC5 | AC012146.1 | 0.487698174 | 1.23E-10 |
| BIRC6 | AC012146.1 | -0.319635222 | 5.03E-05 |
| BNIP1 | AC012146.1 | 0.468419935 | 7.92E-10 |
| CAPN1 | AC012146.1 | -0.315159539 | 6.49E-05 |
| CAPN10 | AC012146.1 | 0.499451108 | 3.71E-11 |
| CAPN2 | AC012146.1 | -0.368144058 | 2.44E-06 |
| CLN3 | AC012146.1 | 0.333626212 | 2.21E-05 |
| DAPK2 | AC012146.1 | 0.376450037 | 1.38E-06 |
| EIF4G1 | AC012146.1 | -0.407568675 | 1.41E-07 |
| FKBP1A | AC012146.1 | 0.428940704 | 2.57E-08 |
| GABARAP | AC012146.1 | 0.449472144 | 4.45E-09 |
| ITGB4 | AC012146.1 | -0.301826786 | 0.000135249 |
| LAMP2 | AC012146.1 | -0.313153485 | 7.27E-05 |
| MAP1LC3A | AC012146.1 | 0.363416065 | 3.36E-06 |
| MAPK1 | AC012146.1 | -0.365406607 | 2.94E-06 |
| MAPK8IP1 | AC012146.1 | 0.458695186 | 1.95E-09 |
| MLST8 | AC012146.1 | 0.357434673 | 4.98E-06 |
| NCKAP1 | AC012146.1 | -0.345706014 | 1.05E-05 |
| PELP1 | AC012146.1 | 0.503708242 | 2.38E-11 |
| TP63 | AC012146.1 | -0.300900648 | 0.000142141 |
| USP10 | AC012146.1 | -0.402266034 | 2.12E-07 |
| WDFY3 | AC012146.1 | -0.338028155 | 1.70E-05 |
| WDR45 | AC012146.1 | 0.478576645 | 3.01E-10 |
| ZFYVE1 | AC012146.1 | -0.319012799 | 5.22E-05 |
| ARSB | FAM83A-AS1 | -0.388287501 | 5.97E-07 |
| ATG4C | FAM83A-AS1 | -0.339900384 | 1.51E-05 |
| BCL2 | FAM83A-AS1 | -0.313677744 | 7.05E-05 |
| BNIP3 | FAM83A-AS1 | 0.370300643 | 2.11E-06 |
| CASP3 | FAM83A-AS1 | -0.309379092 | 8.96E-05 |
| CASP4 | FAM83A-AS1 | 0.371236858 | 1.98E-06 |
| CDKN1A | FAM83A-AS1 | 0.329375873 | 2.85E-05 |
| CDKN1B | FAM83A-AS1 | -0.392015927 | 4.55E-07 |
| DRAM1 | FAM83A-AS1 | -0.307197361 | 0.000101049 |
| EEF2 | FAM83A-AS1 | -0.371435111 | 1.95E-06 |
| EEF2K | FAM83A-AS1 | -0.341281082 | 1.39E-05 |
| EIF2AK3 | FAM83A-AS1 | -0.347604142 | 9.35E-06 |
| HDAC6 | FAM83A-AS1 | -0.373136473 | 1.74E-06 |
| HIF1A | FAM83A-AS1 | 0.303714641 | 0.000122155 |
| KLHL24 | FAM83A-AS1 | -0.35454419 | 6.00E-06 |
| MAP2K7 | FAM83A-AS1 | -0.350785748 | 7.64E-06 |
| NBR1 | FAM83A-AS1 | -0.365135666 | 2.99E-06 |
| PARP1 | FAM83A-AS1 | -0.498378497 | 4.14E-11 |
| PIK3R4 | FAM83A-AS1 | -0.320140431 | 4.89E-05 |
| PRKAB1 | FAM83A-AS1 | -0.361405392 | 3.83E-06 |
| PTK6 | FAM83A-AS1 | 0.498652433 | 4.02E-11 |
| RHEB | FAM83A-AS1 | -0.356075609 | 5.44E-06 |
| SQSTM1 | FAM83A-AS1 | -0.308378321 | 9.47E-05 |
| TP53 | FAM83A-AS1 | -0.378665296 | 1.18E-06 |
| UVRAG | FAM83A-AS1 | -0.308566262 | 9.37E-05 |
| VAMP7 | FAM83A-AS1 | -0.322047285 | 4.38E-05 |
| ATG2B | AC012313.1 | 0.374641192 | 1.57E-06 |
| BECN1 | AC012313.1 | 0.383891343 | 8.18E-07 |
| HGS | AC012313.1 | 0.318756494 | 5.29E-05 |
| RPTOR | AC012313.1 | 0.418944425 | 5.79E-08 |
| SIRT2 | AC012313.1 | 0.321887379 | 4.42E-05 |
| SPNS1 | AC012313.1 | 0.307399636 | 9.99E-05 |
| ATF4 | AC016773.1 | 0.389312347 | 5.54E-07 |
| ATG10 | AC016773.1 | 0.306165959 | 0.000106914 |
| ATG16L2 | AC016773.1 | 0.45242322 | 3.42E-09 |
| ATG3 | AC016773.1 | 0.366727074 | 2.69E-06 |
| ATG4B | AC016773.1 | 0.575393505 | 4.84E-15 |
| ATG4C | AC016773.1 | 0.328375034 | 3.03E-05 |
| ATG4D | AC016773.1 | 0.326468423 | 3.39E-05 |
| BAG1 | AC016773.1 | 0.331370489 | 2.53E-05 |
| BAG3 | AC016773.1 | -0.307445248 | 9.97E-05 |
| BAX | AC016773.1 | 0.574452341 | 5.48E-15 |
| BIRC5 | AC016773.1 | 0.486369193 | 1.40E-10 |
| BNIP1 | AC016773.1 | 0.506609042 | 1.75E-11 |
| CANX | AC016773.1 | -0.307215585 | 0.000100948 |
| CAPN10 | AC016773.1 | 0.610840244 | 3.18E-17 |
| CAPN2 | AC016773.1 | -0.323876969 | 3.94E-05 |
| CDKN2A | AC016773.1 | 0.470199557 | 6.70E-10 |
| CHMP4B | AC016773.1 | 0.330188923 | 2.72E-05 |
| CLN3 | AC016773.1 | 0.374932214 | 1.54E-06 |
| CXCR4 | AC016773.1 | 0.477702836 | 3.27E-10 |
| EIF4G1 | AC016773.1 | -0.364777659 | 3.06E-06 |
| FKBP1A | AC016773.1 | 0.404865238 | 1.74E-07 |
| GABARAP | AC016773.1 | 0.39537845 | 3.55E-07 |
| HDAC6 | AC016773.1 | 0.439817992 | 1.03E-08 |
| HIF1A | AC016773.1 | -0.329052659 | 2.91E-05 |
| IKBKB | AC016773.1 | 0.373107995 | 1.74E-06 |
| IKBKE | AC016773.1 | 0.31260593 | 7.49E-05 |
| ITGA6 | AC016773.1 | -0.327988433 | 3.10E-05 |
| ITGB1 | AC016773.1 | -0.338250793 | 1.67E-05 |
| KIF5B | AC016773.1 | -0.377956818 | 1.24E-06 |
| MAP1LC3A | AC016773.1 | 0.382491782 | 9.04E-07 |
| MAP2K7 | AC016773.1 | 0.378771561 | 1.17E-06 |
| MAPK1 | AC016773.1 | -0.37004415 | 2.15E-06 |
| MLST8 | AC016773.1 | 0.311068876 | 8.16E-05 |
| MTMR14 | AC016773.1 | 0.482770704 | 2.00E-10 |
| NCKAP1 | AC016773.1 | -0.373791881 | 1.66E-06 |
| NKX2-3 | AC016773.1 | 0.301072764 | 0.000140836 |
| PELP1 | AC016773.1 | 0.310478914 | 8.43E-05 |
| PEX14 | AC016773.1 | 0.384037946 | 8.10E-07 |
| PINK1 | AC016773.1 | -0.326736834 | 3.33E-05 |
| PRKAB1 | AC016773.1 | 0.340270597 | 1.48E-05 |
| RAB24 | AC016773.1 | 0.494718195 | 6.03E-11 |
| STK11 | AC016773.1 | 0.477743413 | 3.26E-10 |
| TSC2 | AC016773.1 | 0.350646424 | 7.71E-06 |
| ULK3 | AC016773.1 | 0.372331671 | 1.84E-06 |
| USP10 | AC016773.1 | -0.356454373 | 5.31E-06 |
| WDFY3 | AC016773.1 | -0.346736368 | 9.88E-06 |
| WDR45 | AC016773.1 | 0.673433553 | 7.98E-22 |
| ATG3 | ZBTB11-AS1 | 0.619417962 | 8.57E-18 |
| ATG4A | ZBTB11-AS1 | 0.341161729 | 1.40E-05 |
| BECN1 | ZBTB11-AS1 | 0.353217604 | 6.54E-06 |
| PIK3R4 | ZBTB11-AS1 | 0.493570025 | 6.78E-11 |
| RAB7A | ZBTB11-AS1 | 0.51746518 | 5.41E-12 |
| RAF1 | ZBTB11-AS1 | 0.331288509 | 2.55E-05 |
| ULK3 | ZBTB11-AS1 | 0.355581667 | 5.61E-06 |
| CAPN10 | LINC00342 | 0.300333047 | 0.000146526 |
| CAPN2 | LINC00342 | 0.326663204 | 3.35E-05 |
| CASP3 | LINC00342 | 0.346117716 | 1.03E-05 |
| CD46 | LINC00342 | 0.357626797 | 4.91E-06 |
| FOS | LINC00342 | 0.305997015 | 0.000107905 |
| HDAC1 | LINC00342 | 0.308784206 | 9.26E-05 |
| HDAC6 | LINC00342 | 0.306152989 | 0.00010699 |
| IKBKB | LINC00342 | 0.308009385 | 9.66E-05 |
| SH3GLB1 | LINC00342 | 0.330651436 | 2.64E-05 |
| ULK3 | LINC00342 | 0.308065117 | 9.63E-05 |
| BAK1 | BX470102.1 | -0.30670208 | 0.000103827 |
| BID | BX470102.1 | 0.431406047 | 2.09E-08 |
| CAPN10 | BX470102.1 | 0.331447988 | 2.52E-05 |
| FKBP1B | BX470102.1 | 0.314124547 | 6.88E-05 |
| PTEN | BX470102.1 | -0.305620821 | 0.000110141 |
| ATIC | AL121899.1 | 0.463668738 | 1.23E-09 |
| MAPK8IP1 | AL121899.1 | 0.64561169 | 1.20E-19 |
| PRKAR1A | AL121899.1 | 0.388675858 | 5.80E-07 |
| AMBRA1 | AL031058.1 | -0.304697302 | 0.000115817 |
| ARNT | AL031058.1 | -0.341264025 | 1.39E-05 |
| ARSB | AL031058.1 | -0.37750294 | 1.28E-06 |
| BIRC6 | AL031058.1 | -0.367776161 | 2.50E-06 |
| CD46 | AL031058.1 | -0.366827271 | 2.67E-06 |
| CDKN1A | AL031058.1 | 0.318539945 | 5.36E-05 |
| CX3CL1 | AL031058.1 | -0.304667648 | 0.000116003 |
| EIF2AK3 | AL031058.1 | -0.349392492 | 8.35E-06 |
| FOXO1 | AL031058.1 | -0.347871899 | 9.20E-06 |
| FOXO3 | AL031058.1 | -0.320739531 | 4.72E-05 |
| ITPR1 | AL031058.1 | -0.33635792 | 1.88E-05 |
| KLHL24 | AL031058.1 | -0.332706588 | 2.34E-05 |
| MBTPS2 | AL031058.1 | -0.319310569 | 5.13E-05 |
| NBR1 | AL031058.1 | -0.40729022 | 1.44E-07 |
| PIK3R4 | AL031058.1 | -0.308738832 | 9.28E-05 |
| RB1CC1 | AL031058.1 | -0.310015123 | 8.65E-05 |
| SH3GLB1 | AL031058.1 | -0.30948427 | 8.91E-05 |
| TBK1 | AL031058.1 | -0.308564805 | 9.37E-05 |
| ATF6 | AL033397.2 | 0.372349638 | 1.83E-06 |
| GABARAPL1 | AL033397.2 | 0.38954583 | 5.45E-07 |
| GNAI3 | AL033397.2 | -0.348187285 | 9.02E-06 |
| SQSTM1 | AL033397.2 | 0.381379329 | 9.78E-07 |
| TP63 | AL033397.2 | 0.321669413 | 4.48E-05 |
| NFKB1 | AC107959.3 | 0.302841267 | 0.000128058 |
| BAX | AC109322.1 | 0.510421838 | 1.16E-11 |
| BIRC5 | AC109322.1 | 0.343169968 | 1.23E-05 |
| EIF4EBP1 | AC109322.1 | 0.368493487 | 2.39E-06 |
| FKBP1A | AC109322.1 | 0.324391263 | 3.82E-05 |
| WDR45 | AC109322.1 | 0.319147987 | 5.18E-05 |
| ARSB | WNT5A-AS1 | 0.334367794 | 2.12E-05 |
| BIRC6 | WNT5A-AS1 | 0.45464698 | 2.80E-09 |
| EIF2AK3 | WNT5A-AS1 | 0.34728023 | 9.55E-06 |
| EIF4G1 | WNT5A-AS1 | 0.366338624 | 2.76E-06 |
| FOXO1 | WNT5A-AS1 | 0.401474056 | 2.25E-07 |
| FOXO3 | WNT5A-AS1 | 0.315602344 | 6.33E-05 |
| GABARAPL1 | WNT5A-AS1 | 0.362298722 | 3.61E-06 |
| KLHL24 | WNT5A-AS1 | 0.416831264 | 6.85E-08 |
| MBTPS2 | WNT5A-AS1 | 0.357817528 | 4.85E-06 |
| NAMPT | WNT5A-AS1 | 0.633917093 | 8.49E-19 |
| NBR1 | WNT5A-AS1 | 0.345010985 | 1.10E-05 |
| NCKAP1 | WNT5A-AS1 | 0.342868651 | 1.26E-05 |
| NFE2L2 | WNT5A-AS1 | 0.346194151 | 1.02E-05 |
| PEX14 | WNT5A-AS1 | -0.378424093 | 1.20E-06 |
| SQSTM1 | WNT5A-AS1 | 0.32527341 | 3.63E-05 |
| TP63 | WNT5A-AS1 | 0.510118031 | 1.20E-11 |
| ATG16L2 | ZNF213-AS1 | 0.41193442 | 1.01E-07 |
| ATG4B | ZNF213-AS1 | 0.354474972 | 6.03E-06 |
| CAPN10 | ZNF213-AS1 | 0.469589913 | 7.10E-10 |
| CDKN1B | ZNF213-AS1 | 0.319957184 | 4.94E-05 |
| CLN3 | ZNF213-AS1 | 0.316815305 | 5.91E-05 |
| CXCR4 | ZNF213-AS1 | 0.335281639 | 2.00E-05 |
| DAPK2 | ZNF213-AS1 | 0.353498612 | 6.42E-06 |
| HDAC6 | ZNF213-AS1 | 0.333958659 | 2.17E-05 |
| IKBKB | ZNF213-AS1 | 0.313692975 | 7.05E-05 |
| MAP2K7 | ZNF213-AS1 | 0.411065582 | 1.08E-07 |
| MLST8 | ZNF213-AS1 | 0.460574194 | 1.64E-09 |
| STK11 | ZNF213-AS1 | 0.317764635 | 5.60E-05 |
| TSC1 | ZNF213-AS1 | 0.300607716 | 0.000144388 |
| ULK3 | ZNF213-AS1 | 0.475113915 | 4.20E-10 |
| ARNT | AC092611.2 | 0.5088371 | 1.38E-11 |
| ATF4 | AC092611.2 | -0.437748031 | 1.23E-08 |
| ATG2A | AC092611.2 | 0.336587383 | 1.85E-05 |
| ATG2B | AC092611.2 | 0.538734691 | 4.80E-13 |
| ATG4B | AC092611.2 | -0.336374388 | 1.87E-05 |
| BAX | AC092611.2 | -0.449657646 | 4.37E-09 |
| BIRC6 | AC092611.2 | 0.523109782 | 2.89E-12 |
| BNIP1 | AC092611.2 | -0.437408618 | 1.26E-08 |
| CAMKK2 | AC092611.2 | 0.379841618 | 1.09E-06 |
| CAPN10 | AC092611.2 | -0.337022366 | 1.80E-05 |
| CAPN2 | AC092611.2 | 0.362281139 | 3.62E-06 |
| EDEM1 | AC092611.2 | 0.40218777 | 2.13E-07 |
| EEF2K | AC092611.2 | 0.353722421 | 6.33E-06 |
| EIF2AK3 | AC092611.2 | 0.346258517 | 1.02E-05 |
| ERN1 | AC092611.2 | 0.31082876 | 8.27E-05 |
| FOXO3 | AC092611.2 | 0.429525818 | 2.45E-08 |
| GOPC | AC092611.2 | 0.347411971 | 9.47E-06 |
| KIF5B | AC092611.2 | 0.382207751 | 9.22E-07 |
| MAPK1 | AC092611.2 | 0.511668348 | 1.02E-11 |
| MBTPS2 | AC092611.2 | 0.30973018 | 8.79E-05 |
| MTOR | AC092611.2 | 0.484944818 | 1.61E-10 |
| NAF1 | AC092611.2 | 0.530670651 | 1.23E-12 |
| NBR1 | AC092611.2 | 0.4144263 | 8.28E-08 |
| NCKAP1 | AC092611.2 | 0.400771475 | 2.37E-07 |
| NFKB1 | AC092611.2 | 0.499220993 | 3.79E-11 |
| PIK3R4 | AC092611.2 | 0.340428565 | 1.46E-05 |
| RAB24 | AC092611.2 | -0.335670843 | 1.96E-05 |
| RAB33B | AC092611.2 | 0.427322714 | 2.93E-08 |
| RAB5A | AC092611.2 | 0.318012395 | 5.52E-05 |
| RB1 | AC092611.2 | 0.35116363 | 7.46E-06 |
| RB1CC1 | AC092611.2 | 0.442180971 | 8.40E-09 |
| RELA | AC092611.2 | 0.393440643 | 4.10E-07 |
| RPS6KB1 | AC092611.2 | 0.338250855 | 1.67E-05 |
| RPTOR | AC092611.2 | 0.340477749 | 1.46E-05 |
| SH3GLB1 | AC092611.2 | 0.343831129 | 1.19E-05 |
| TP63 | AC092611.2 | 0.313619263 | 7.08E-05 |
| TSC1 | AC092611.2 | 0.352196017 | 6.98E-06 |
| WDFY3 | AC092611.2 | 0.607197346 | 5.49E-17 |
| GABARAPL1 | AC115522.1 | -0.332752886 | 2.33E-05 |
| MAPK3 | AC115522.1 | 0.433566777 | 1.75E-08 |
| RAB24 | AC115522.1 | 0.365675176 | 2.88E-06 |
| ST13 | AC115522.1 | -0.308685986 | 9.31E-05 |
| VAMP3 | AC115522.1 | 0.379209958 | 1.14E-06 |
| ATIC | LINC01063 | 0.337306698 | 1.77E-05 |
| BIRC5 | LINC01063 | 0.422086171 | 4.50E-08 |
| CAPN2 | LINC01063 | -0.355876711 | 5.51E-06 |
| GNAI3 | LINC01063 | -0.302816615 | 0.000128228 |
| KLHL24 | LINC01063 | 0.360918409 | 3.96E-06 |
| MAPK8IP1 | LINC01063 | 0.369721681 | 2.19E-06 |
| MBTPS2 | LINC01063 | 0.301037549 | 0.000141102 |
| NFKB1 | LINC01063 | -0.322698176 | 4.22E-05 |
| ST13 | LINC01063 | 0.312733205 | 7.44E-05 |
| VAMP7 | LINC01063 | 0.372973904 | 1.76E-06 |
| ATG3 | AC104411.1 | 0.37432007 | 1.60E-06 |
| BECN1 | AC104411.1 | 0.344467169 | 1.14E-05 |
| CAPN2 | AC104411.1 | 0.322168697 | 4.35E-05 |
| EIF2AK3 | AC104411.1 | 0.484529046 | 1.68E-10 |
| KLHL24 | AC104411.1 | 0.318589416 | 5.34E-05 |
| NFE2L2 | AC104411.1 | 0.372071467 | 1.87E-06 |
| PIK3R4 | AC104411.1 | 0.460444632 | 1.66E-09 |
| RAB5A | AC104411.1 | 0.360122271 | 4.17E-06 |
| RAF1 | AC104411.1 | 0.379586731 | 1.11E-06 |
| TP53INP2 | AC104411.1 | 0.304405188 | 0.000117668 |
| ATG12 | VPS9D1-AS1 | -0.335616607 | 1.96E-05 |
| ATG4C | VPS9D1-AS1 | -0.308887147 | 9.21E-05 |
| BNIP1 | VPS9D1-AS1 | -0.334998953 | 2.04E-05 |
| CAMKK2 | VPS9D1-AS1 | 0.334079706 | 2.15E-05 |
| EIF4G1 | VPS9D1-AS1 | 0.384795859 | 7.67E-07 |
| GABARAP | VPS9D1-AS1 | -0.336457608 | 1.87E-05 |
| HSP90AB1 | VPS9D1-AS1 | 0.322399075 | 4.29E-05 |
| HSPA8 | VPS9D1-AS1 | 0.34603163 | 1.03E-05 |
| MAP1LC3B | VPS9D1-AS1 | 0.310105679 | 8.61E-05 |
| MYC | VPS9D1-AS1 | 0.415960453 | 7.34E-08 |
| NRG1 | VPS9D1-AS1 | 0.374658939 | 1.56E-06 |
| RAB24 | VPS9D1-AS1 | -0.303698083 | 0.000122264 |
| TP73 | VPS9D1-AS1 | -0.336161736 | 1.90E-05 |
| USP10 | VPS9D1-AS1 | 0.476325783 | 3.74E-10 |
| ATIC | LINC02482 | 0.522402199 | 3.13E-12 |
| BIRC5 | LINC02482 | 0.366616235 | 2.71E-06 |
| DIRAS3 | LINC02482 | 0.519704532 | 4.23E-12 |
| FKBP1B | LINC02482 | 0.313409139 | 7.16E-05 |
| GABARAPL2 | LINC02482 | 0.445752621 | 6.16E-09 |
| MAPK8IP1 | LINC02482 | 0.715035363 | 1.46E-25 |
| PRKAR1A | LINC02482 | 0.420670245 | 5.04E-08 |
| PRKCQ | LINC02482 | 0.363531167 | 3.33E-06 |
| BIRC6 | AC022150.4 | 0.355672201 | 5.58E-06 |
| EDEM1 | AC022150.4 | 0.352431073 | 6.88E-06 |
| EEF2K | AC022150.4 | 0.323244284 | 4.09E-05 |
| NAF1 | AC022150.4 | 0.336183623 | 1.90E-05 |
| NPC1 | AC022150.4 | 0.319287232 | 5.13E-05 |
| PIK3R4 | AC022150.4 | 0.423832451 | 3.90E-08 |
| TSC1 | AC022150.4 | 0.429158356 | 2.52E-08 |
| ARNT | AC002401.4 | -0.350547379 | 7.76E-06 |
| BAK1 | AC002401.4 | 0.38092092 | 1.01E-06 |
| CASP1 | AC002401.4 | 0.579626715 | 2.74E-15 |
| CASP4 | AC002401.4 | 0.492401713 | 7.63E-11 |
| EEF2K | AC002401.4 | -0.302379247 | 0.000131288 |
| EIF2AK3 | AC002401.4 | -0.329677138 | 2.80E-05 |
| ITGA3 | AC002401.4 | 0.437537978 | 1.25E-08 |
| KLHL24 | AC002401.4 | -0.308650703 | 9.33E-05 |
| PARP1 | AC002401.4 | -0.322586126 | 4.25E-05 |
| PTK6 | AC002401.4 | 0.320522922 | 4.78E-05 |
| SIRT1 | AC002401.4 | -0.31641615 | 6.05E-05 |
| ATG10 | FLJ37453 | 0.325129479 | 3.66E-05 |
| ATG4C | FLJ37453 | 0.361877625 | 3.72E-06 |
| BAK1 | FLJ37453 | -0.309475826 | 8.91E-05 |
| BAX | FLJ37453 | 0.321763915 | 4.45E-05 |
| CAPN10 | FLJ37453 | 0.3187535 | 5.29E-05 |
| CDKN1B | FLJ37453 | 0.333195443 | 2.27E-05 |
| CLN3 | FLJ37453 | 0.300329374 | 0.000146554 |
| HDAC1 | FLJ37453 | 0.408287082 | 1.34E-07 |
| HDAC6 | FLJ37453 | 0.434524577 | 1.61E-08 |
| ITGA3 | FLJ37453 | -0.325814258 | 3.52E-05 |
| MAPK8IP1 | FLJ37453 | 0.313450573 | 7.15E-05 |
| MTMR14 | FLJ37453 | 0.36562752 | 2.89E-06 |
| PEX14 | FLJ37453 | 0.349488919 | 8.30E-06 |
| PRKAB1 | FLJ37453 | 0.350122276 | 7.97E-06 |
| RAF1 | FLJ37453 | 0.325021709 | 3.69E-05 |
| ULK3 | FLJ37453 | 0.30835067 | 9.48E-05 |
| NPC1 | AC091729.3 | 0.312426554 | 7.57E-05 |
| WIPI2 | AC091729.3 | 0.460053789 | 1.72E-09 |
| ATG16L2 | Z82243.1 | 0.302869342 | 0.000127864 |
| CAPN10 | Z82243.1 | 0.32448081 | 3.80E-05 |
| NFE2L2 | Z82243.1 | 0.319697841 | 5.02E-05 |
| ATG4A | CARD8-AS1 | 0.306890963 | 0.000102759 |
| ATG4C | CARD8-AS1 | 0.398915518 | 2.73E-07 |
| BCL2 | CARD8-AS1 | 0.313379843 | 7.17E-05 |
| CALCOCO2 | CARD8-AS1 | 0.341084351 | 1.41E-05 |
| CASP8 | CARD8-AS1 | 0.350203383 | 7.93E-06 |
| CCR2 | CARD8-AS1 | 0.459643387 | 1.78E-09 |
| CFLAR | CARD8-AS1 | 0.488272168 | 1.16E-10 |
| CXCR4 | CARD8-AS1 | 0.401885113 | 2.18E-07 |
| FAS | CARD8-AS1 | 0.332363617 | 2.39E-05 |
| GAPDH | CARD8-AS1 | -0.438224952 | 1.18E-08 |
| HDAC6 | CARD8-AS1 | 0.304317332 | 0.00011823 |
| IFNG | CARD8-AS1 | 0.375017003 | 1.53E-06 |
| ITPR1 | CARD8-AS1 | 0.362841442 | 3.49E-06 |
| MTMR14 | CARD8-AS1 | 0.366908868 | 2.66E-06 |
| RAF1 | CARD8-AS1 | 0.384020506 | 8.11E-07 |
| RGS19 | CARD8-AS1 | 0.371127957 | 1.99E-06 |
| SH3GLB1 | CARD8-AS1 | 0.336449021 | 1.87E-05 |
| SIRT2 | CARD8-AS1 | 0.3387243 | 1.62E-05 |
| TNFSF10 | CARD8-AS1 | 0.347327242 | 9.52E-06 |
| ATG16L2 | MALAT1 | 0.342432889 | 1.29E-05 |
| HDAC6 | MALAT1 | 0.345394677 | 1.07E-05 |
| IKBKB | MALAT1 | 0.366632284 | 2.71E-06 |
| ATG3 | LINC00885 | 0.340373212 | 1.47E-05 |
| ATG4D | LINC00885 | 0.432390654 | 1.93E-08 |
| BNIP3L | LINC00885 | 0.312360358 | 7.59E-05 |
| CASP4 | LINC00885 | -0.321769075 | 4.45E-05 |
| CDKN1B | LINC00885 | 0.344211423 | 1.16E-05 |
| CX3CL1 | LINC00885 | 0.314657801 | 6.68E-05 |
| EIF2AK3 | LINC00885 | 0.381560468 | 9.65E-07 |
| EIF2S1 | LINC00885 | -0.31215851 | 7.68E-05 |
| GABARAPL1 | LINC00885 | 0.397551939 | 3.02E-07 |
| HDAC6 | LINC00885 | 0.301870324 | 0.000134933 |
| IKBKE | LINC00885 | 0.326817433 | 3.32E-05 |
| ITGA3 | LINC00885 | -0.34685735 | 9.80E-06 |
| ITGA6 | LINC00885 | -0.309233007 | 9.03E-05 |
| KLHL24 | LINC00885 | 0.340264498 | 1.48E-05 |
| MAP2K7 | LINC00885 | 0.383285463 | 8.54E-07 |
| NBR1 | LINC00885 | 0.353758712 | 6.32E-06 |
| NFE2L2 | LINC00885 | 0.377673006 | 1.27E-06 |
| NKX2-3 | LINC00885 | 0.306427803 | 0.000105396 |
| PIK3R4 | LINC00885 | 0.382538148 | 9.01E-07 |
| PINK1 | LINC00885 | -0.307947457 | 9.70E-05 |
| PRKAB1 | LINC00885 | 0.37930998 | 1.13E-06 |
| RAF1 | LINC00885 | 0.342216257 | 1.31E-05 |
| TP53 | LINC00885 | 0.334652829 | 2.08E-05 |
| TSC2 | LINC00885 | 0.328385602 | 3.03E-05 |
| ULK1 | LINC00885 | 0.415951189 | 7.34E-08 |
| ARSB | AC115618.2 | 0.360866975 | 3.97E-06 |
| ATG4C | AC115618.2 | 0.303866638 | 0.000121154 |
| BCL2 | AC115618.2 | 0.371075623 | 2.00E-06 |
| CAMKK2 | AC115618.2 | 0.309482113 | 8.91E-05 |
| CX3CL1 | AC115618.2 | 0.371224802 | 1.98E-06 |
| CXCR4 | AC115618.2 | 0.363925848 | 3.24E-06 |
| DRAM1 | AC115618.2 | 0.38153308 | 9.67E-07 |
| EEF2K | AC115618.2 | 0.435460399 | 1.49E-08 |
| HDAC6 | AC115618.2 | 0.503776191 | 2.36E-11 |
| IKBKB | AC115618.2 | 0.341840483 | 1.34E-05 |
| KLHL24 | AC115618.2 | 0.304343355 | 0.000118063 |
| MAP2K7 | AC115618.2 | 0.332654076 | 2.35E-05 |
| MAPK8 | AC115618.2 | 0.329188887 | 2.88E-05 |
| MBTPS2 | AC115618.2 | 0.317761329 | 5.60E-05 |
| NKX2-3 | AC115618.2 | 0.34578377 | 1.05E-05 |
| PRKAB1 | AC115618.2 | 0.332447368 | 2.38E-05 |
| SIRT1 | AC115618.2 | 0.305680526 | 0.000109783 |
| TP53 | AC115618.2 | 0.400456016 | 2.43E-07 |
| TP73 | AC115618.2 | 0.434636998 | 1.60E-08 |
| TSC2 | AC115618.2 | 0.454380143 | 2.87E-09 |
| ULK2 | AC115618.2 | 0.30156671 | 0.000137152 |
| PPP1R15A | GAPLINC | 0.502993242 | 2.56E-11 |
| ATG4B | AL117332.1 | 0.326308756 | 3.42E-05 |
| ATIC | AL117332.1 | 0.312792388 | 7.41E-05 |
| BAG3 | AL117332.1 | -0.334161849 | 2.14E-05 |
| BAX | AL117332.1 | 0.477361142 | 3.38E-10 |
| BIRC5 | AL117332.1 | 0.496010937 | 5.28E-11 |
| BNIP1 | AL117332.1 | 0.333618347 | 2.21E-05 |
| CAPN10 | AL117332.1 | 0.346248043 | 1.02E-05 |
| CAPN2 | AL117332.1 | -0.307580084 | 9.90E-05 |
| CXCR4 | AL117332.1 | 0.329759832 | 2.79E-05 |
| FKBP1A | AL117332.1 | 0.329923606 | 2.76E-05 |
| HDAC6 | AL117332.1 | 0.448161376 | 4.99E-09 |
| ITGA3 | AL117332.1 | -0.321446164 | 4.54E-05 |
| ITGA6 | AL117332.1 | -0.308807469 | 9.25E-05 |
| MAPK8IP1 | AL117332.1 | 0.380284486 | 1.06E-06 |
| MLST8 | AL117332.1 | 0.314294393 | 6.81E-05 |
| RB1 | AL117332.1 | -0.302338067 | 0.000131579 |
| WDR45 | AL117332.1 | 0.45089431 | 3.92E-09 |
| ATG16L2 | AP005482.1 | 0.31010215 | 8.61E-05 |
| ARNT | FAM160A1-DT | 0.463045864 | 1.31E-09 |
| ATF4 | FAM160A1-DT | -0.396150816 | 3.35E-07 |
| ATF6 | FAM160A1-DT | 0.326816306 | 3.32E-05 |
| ATG2B | FAM160A1-DT | 0.543437543 | 2.74E-13 |
| BIRC6 | FAM160A1-DT | 0.400512149 | 2.42E-07 |
| CD46 | FAM160A1-DT | 0.313863326 | 6.98E-05 |
| DNAJB9 | FAM160A1-DT | 0.368406013 | 2.40E-06 |
| EEF2K | FAM160A1-DT | 0.301659343 | 0.000136472 |
| GOPC | FAM160A1-DT | 0.358476031 | 4.65E-06 |
| MAPK1 | FAM160A1-DT | 0.363573211 | 3.32E-06 |
| MTOR | FAM160A1-DT | 0.439986877 | 1.01E-08 |
| NAF1 | FAM160A1-DT | 0.40815307 | 1.35E-07 |
| NBR1 | FAM160A1-DT | 0.384219967 | 7.99E-07 |
| NCKAP1 | FAM160A1-DT | 0.32674875 | 3.33E-05 |
| RAB33B | FAM160A1-DT | 0.462980332 | 1.31E-09 |
| RB1CC1 | FAM160A1-DT | 0.34088218 | 1.42E-05 |
| RELA | FAM160A1-DT | 0.311357813 | 8.03E-05 |
| SIRT1 | FAM160A1-DT | 0.382305973 | 9.16E-07 |
| TP53INP2 | FAM160A1-DT | 0.325084615 | 3.67E-05 |
| TP63 | FAM160A1-DT | 0.343604568 | 1.20E-05 |
| USP10 | FAM160A1-DT | 0.312214814 | 7.66E-05 |
| WDFY3 | FAM160A1-DT | 0.444862347 | 6.66E-09 |
| CASP8 | AL359771.1 | 0.335411468 | 1.99E-05 |
| CFLAR | AL359771.1 | 0.307529316 | 9.92E-05 |
| HIF1A | AL359771.1 | 0.3255056 | 3.58E-05 |
| SIRT2 | AL359771.1 | 0.42050619 | 5.11E-08 |
| VAMP3 | AL359771.1 | 0.423529768 | 4.00E-08 |
| ATF4 | AL392172.1 | 0.335148985 | 2.02E-05 |
| ATG10 | AL392172.1 | 0.321733892 | 4.46E-05 |
| ATG4B | AL392172.1 | 0.420151143 | 5.25E-08 |
| ATG4D | AL392172.1 | 0.441550751 | 8.87E-09 |
| BAG1 | AL392172.1 | 0.348268762 | 8.97E-06 |
| BAX | AL392172.1 | 0.616069257 | 1.44E-17 |
| BIRC5 | AL392172.1 | 0.383497812 | 8.41E-07 |
| BNIP1 | AL392172.1 | 0.453378267 | 3.14E-09 |
| CAPN10 | AL392172.1 | 0.523887807 | 2.65E-12 |
| CAPNS1 | AL392172.1 | 0.324805022 | 3.73E-05 |
| CASP3 | AL392172.1 | 0.327343762 | 3.22E-05 |
| CDKN2A | AL392172.1 | 0.356447466 | 5.31E-06 |
| CLN3 | AL392172.1 | 0.375390533 | 1.49E-06 |
| CXCR4 | AL392172.1 | 0.374174995 | 1.62E-06 |
| FKBP1A | AL392172.1 | 0.445430815 | 6.34E-09 |
| GABARAP | AL392172.1 | 0.479357875 | 2.79E-10 |
| HDAC6 | AL392172.1 | 0.359415674 | 4.37E-06 |
| HIF1A | AL392172.1 | -0.312448362 | 7.56E-05 |
| IKBKE | AL392172.1 | 0.337666817 | 1.73E-05 |
| ITGA3 | AL392172.1 | -0.401203467 | 2.30E-07 |
| ITGA6 | AL392172.1 | -0.313070551 | 7.30E-05 |
| ITGB4 | AL392172.1 | -0.354090173 | 6.18E-06 |
| MAP2K7 | AL392172.1 | 0.331000947 | 2.59E-05 |
| MLST8 | AL392172.1 | 0.305489217 | 0.000110934 |
| MTMR14 | AL392172.1 | 0.377197129 | 1.31E-06 |
| PELP1 | AL392172.1 | 0.416203639 | 7.20E-08 |
| PINK1 | AL392172.1 | -0.421070185 | 4.88E-08 |
| PRKAB1 | AL392172.1 | 0.319942537 | 4.95E-05 |
| PTK6 | AL392172.1 | -0.344238273 | 1.16E-05 |
| RAB24 | AL392172.1 | 0.311777378 | 7.85E-05 |
| WDFY3 | AL392172.1 | -0.328203825 | 3.06E-05 |
| WDR45 | AL392172.1 | 0.644763573 | 1.39E-19 |
| ATG4D | NKILA | -0.332515651 | 2.37E-05 |
| FADD | NKILA | 0.321843865 | 4.43E-05 |
| GOPC | NKILA | 0.394574801 | 3.77E-07 |
| IL24 | NKILA | 0.408180287 | 1.35E-07 |
| ITGA3 | NKILA | 0.360066381 | 4.19E-06 |
| ITGB4 | NKILA | 0.320120969 | 4.89E-05 |
| ULK3 | NKILA | -0.303878045 | 0.000121079 |
| ITGA3 | AC110995.1 | 0.357895403 | 4.83E-06 |
| PINK1 | AC110995.1 | 0.315799601 | 6.26E-05 |
| ATG4D | DGCR11 | -0.318520407 | 5.36E-05 |
| NRG1 | DGCR11 | 0.345643341 | 1.06E-05 |
| CASP1 | AC138207.5 | 0.324498007 | 3.80E-05 |
| CTSD | AC138207.5 | 0.329542541 | 2.83E-05 |
| GOPC | AC138207.5 | -0.301227846 | 0.00013967 |
| NCKAP1 | AC138207.5 | -0.381760201 | 9.52E-07 |
| RAB24 | AC138207.5 | 0.441835777 | 8.65E-09 |
| RB1CC1 | AC138207.5 | -0.31488765 | 6.59E-05 |
| AMBRA1 | AC018521.6 | 0.324482609 | 3.80E-05 |
| MAPK1 | AC018521.6 | 0.311582083 | 7.93E-05 |
| PIK3R4 | AC018521.6 | 0.340773247 | 1.43E-05 |
| RAF1 | AC018521.6 | 0.304428228 | 0.000117521 |
| TSC1 | AC018521.6 | 0.456540051 | 2.36E-09 |
| NFE2L2 | LINC01010 | 0.381701152 | 9.56E-07 |
| PIK3C3 | LINC01010 | 0.350658984 | 7.71E-06 |
| RAB1A | LINC01010 | 0.321279354 | 4.58E-05 |
| TP63 | LINC01010 | 0.391322407 | 4.78E-07 |
| USP10 | LINC01010 | 0.300527478 | 0.00014501 |
| UVRAG | LINC01010 | 0.324594444 | 3.78E-05 |
| ATF4 | AC084824.5 | 0.385852797 | 7.11E-07 |
| ATG16L2 | AC084824.5 | 0.391337455 | 4.78E-07 |
| ATG4B | AC084824.5 | 0.541236806 | 3.57E-13 |
| BAG3 | AC084824.5 | -0.366519333 | 2.73E-06 |
| BAX | AC084824.5 | 0.453419254 | 3.13E-09 |
| BNIP1 | AC084824.5 | 0.352304959 | 6.94E-06 |
| CAPN10 | AC084824.5 | 0.559304891 | 3.90E-14 |
| CDKN1B | AC084824.5 | 0.381958946 | 9.39E-07 |
| CDKN2A | AC084824.5 | 0.310972444 | 8.20E-05 |
| CX3CL1 | AC084824.5 | 0.316834448 | 5.90E-05 |
| CXCR4 | AC084824.5 | 0.658650808 | 1.22E-20 |
| DAPK2 | AC084824.5 | 0.511341717 | 1.05E-11 |
| EIF4G1 | AC084824.5 | -0.416259084 | 7.17E-08 |
| FKBP1A | AC084824.5 | 0.3246042 | 3.78E-05 |
| GABARAP | AC084824.5 | 0.330573412 | 2.66E-05 |
| HDAC6 | AC084824.5 | 0.338567433 | 1.64E-05 |
| HIF1A | AC084824.5 | -0.332615917 | 2.35E-05 |
| IKBKB | AC084824.5 | 0.342527175 | 1.29E-05 |
| KIF5B | AC084824.5 | -0.330388939 | 2.69E-05 |
| MAP1LC3B | AC084824.5 | -0.317052835 | 5.83E-05 |
| MAP2K7 | AC084824.5 | 0.383566102 | 8.37E-07 |
| MTMR14 | AC084824.5 | 0.340089155 | 1.49E-05 |
| NCKAP1 | AC084824.5 | -0.34360815 | 1.20E-05 |
| NKX2-3 | AC084824.5 | 0.319719278 | 5.01E-05 |
| PELP1 | AC084824.5 | 0.336619225 | 1.85E-05 |
| PEX14 | AC084824.5 | 0.308027297 | 9.65E-05 |
| RAB24 | AC084824.5 | 0.330707521 | 2.64E-05 |
| STK11 | AC084824.5 | 0.347781299 | 9.25E-06 |
| TP53 | AC084824.5 | 0.312396227 | 7.58E-05 |
| TP73 | AC084824.5 | 0.313082872 | 7.29E-05 |
| TSC2 | AC084824.5 | 0.490686509 | 9.08E-11 |
| ULK3 | AC084824.5 | 0.317120212 | 5.81E-05 |
| USP10 | AC084824.5 | -0.378274115 | 1.22E-06 |
| WDFY3 | AC084824.5 | -0.340808895 | 1.43E-05 |
| WDR45 | AC084824.5 | 0.44477555 | 6.71E-09 |
| ATG3 | STARD7-AS1 | 0.302457234 | 0.000130737 |
| ATG4B | STARD7-AS1 | 0.343358417 | 1.22E-05 |
| ATG4D | STARD7-AS1 | 0.312092883 | 7.71E-05 |
| ATG9B | STARD7-AS1 | 0.348630596 | 8.77E-06 |
| BID | STARD7-AS1 | 0.363818338 | 3.27E-06 |
| CAPN10 | STARD7-AS1 | 0.318492126 | 5.37E-05 |
| EIF2AK3 | STARD7-AS1 | 0.315501167 | 6.37E-05 |
| IKBKB | STARD7-AS1 | 0.318434759 | 5.39E-05 |
| MAP2K7 | STARD7-AS1 | 0.396534914 | 3.26E-07 |
| PELP1 | STARD7-AS1 | 0.305201053 | 0.000112688 |
| PINK1 | STARD7-AS1 | -0.341434264 | 1.38E-05 |
| PRKAB1 | STARD7-AS1 | 0.346300971 | 1.02E-05 |
| RAF1 | STARD7-AS1 | 0.323783174 | 3.96E-05 |
| STK11 | STARD7-AS1 | 0.321173919 | 4.61E-05 |
| TSC1 | STARD7-AS1 | 0.324843623 | 3.72E-05 |
| TSC2 | STARD7-AS1 | 0.332449231 | 2.38E-05 |
| ULK3 | STARD7-AS1 | 0.301916392 | 0.000134599 |
| ATG10 | AC015813.1 | 0.309482299 | 8.91E-05 |
| ATG3 | AC015813.1 | 0.432445587 | 1.92E-08 |
| ATG4B | AC015813.1 | 0.335775424 | 1.94E-05 |
| ATG4C | AC015813.1 | 0.410069575 | 1.16E-07 |
| ATG4D | AC015813.1 | 0.311204896 | 8.10E-05 |
| CAPN10 | AC015813.1 | 0.384620845 | 7.77E-07 |
| CD46 | AC015813.1 | 0.350784272 | 7.64E-06 |
| CXCR4 | AC015813.1 | 0.300447537 | 0.000145631 |
| DAPK2 | AC015813.1 | 0.353817546 | 6.29E-06 |
| EEF2K | AC015813.1 | 0.30148891 | 0.000137727 |
| HDAC1 | AC015813.1 | 0.305401806 | 0.000111463 |
| HDAC6 | AC015813.1 | 0.485430286 | 1.54E-10 |
| IKBKB | AC015813.1 | 0.370268573 | 2.11E-06 |
| MAP2K7 | AC015813.1 | 0.532950019 | 9.43E-13 |
| PEX14 | AC015813.1 | 0.343243315 | 1.23E-05 |
| RAF1 | AC015813.1 | 0.394170521 | 3.88E-07 |
| STK11 | AC015813.1 | 0.46512842 | 1.08E-09 |
| TP73 | AC015813.1 | 0.321080658 | 4.63E-05 |
| TSC1 | AC015813.1 | 0.39642615 | 3.29E-07 |
| TSC2 | AC015813.1 | 0.331084941 | 2.58E-05 |
| ULK3 | AC015813.1 | 0.37658183 | 1.37E-06 |
| ARSB | AC004816.1 | -0.382820999 | 8.83E-07 |
| ATG9B | AC004816.1 | 0.698066798 | 5.85E-24 |
| FOXO1 | AC004816.1 | -0.425730505 | 3.34E-08 |
| GAA | AC004816.1 | -0.314459461 | 6.75E-05 |
| KLHL24 | AC004816.1 | -0.300431576 | 0.000145756 |
| MAPK9 | AC004816.1 | -0.352578094 | 6.82E-06 |
| MYC | AC004816.1 | 0.304897824 | 0.000114562 |
| PARP1 | AC004816.1 | -0.306235135 | 0.000106511 |
| PTK6 | AC004816.1 | 0.487761314 | 1.22E-10 |
| RAB11A | AC004816.1 | 0.492963909 | 7.21E-11 |
| RAB1A | AC004816.1 | 0.306920738 | 0.000102592 |
| TP53INP2 | AC004816.1 | 0.438317258 | 1.17E-08 |
| ULK2 | AC004816.1 | -0.319671168 | 5.02E-05 |
| ATF4 | AL390728.6 | 0.302522987 | 0.000130275 |
| ATG12 | AL390728.6 | 0.362256866 | 3.62E-06 |
| ATG16L2 | AL390728.6 | 0.486004847 | 1.45E-10 |
| ATG3 | AL390728.6 | 0.309379861 | 8.96E-05 |
| ATG4B | AL390728.6 | 0.436189034 | 1.40E-08 |
| ATG4C | AL390728.6 | 0.331885176 | 2.46E-05 |
| BAX | AL390728.6 | 0.342531752 | 1.28E-05 |
| CAPN10 | AL390728.6 | 0.546885068 | 1.81E-13 |
| CASP3 | AL390728.6 | 0.37726161 | 1.31E-06 |
| CFLAR | AL390728.6 | 0.303431901 | 0.000124037 |
| CX3CL1 | AL390728.6 | 0.323716187 | 3.98E-05 |
| CXCR4 | AL390728.6 | 0.382685147 | 8.91E-07 |
| DAPK2 | AL390728.6 | 0.539540088 | 4.36E-13 |
| EIF4G1 | AL390728.6 | -0.300497931 | 0.000145239 |
| HDAC6 | AL390728.6 | 0.387639113 | 6.25E-07 |
| IKBKB | AL390728.6 | 0.321816927 | 4.44E-05 |
| MAP2K7 | AL390728.6 | 0.308506416 | 9.40E-05 |
| PELP1 | AL390728.6 | 0.387597332 | 6.27E-07 |
| PEX14 | AL390728.6 | 0.301016954 | 0.000141258 |
| PINK1 | AL390728.6 | -0.336222169 | 1.89E-05 |
| RAB24 | AL390728.6 | 0.352894281 | 6.68E-06 |
| STK11 | AL390728.6 | 0.348113324 | 9.06E-06 |
| ULK3 | AL390728.6 | 0.465507291 | 1.04E-09 |
| WDR45 | AL390728.6 | 0.443577111 | 7.44E-09 |
| ARNT | AL132989.1 | 0.387947459 | 6.11E-07 |
| ARSB | AL132989.1 | 0.342026231 | 1.33E-05 |
| ATG2A | AL132989.1 | 0.314616308 | 6.69E-05 |
| CAMKK2 | AL132989.1 | 0.31377768 | 7.02E-05 |
| CD46 | AL132989.1 | 0.369049683 | 2.30E-06 |
| CDKN1B | AL132989.1 | 0.317490254 | 5.69E-05 |
| CFLAR | AL132989.1 | 0.30027802 | 0.000146957 |
| CX3CL1 | AL132989.1 | 0.412081975 | 9.96E-08 |
| CXCR4 | AL132989.1 | 0.472484179 | 5.40E-10 |
| EDEM1 | AL132989.1 | 0.307755529 | 9.80E-05 |
| EEF2K | AL132989.1 | 0.466399427 | 9.57E-10 |
| EIF2AK3 | AL132989.1 | 0.33919778 | 1.58E-05 |
| FAS | AL132989.1 | 0.329084496 | 2.90E-05 |
| HDAC6 | AL132989.1 | 0.411715969 | 1.02E-07 |
| IKBKB | AL132989.1 | 0.402523998 | 2.08E-07 |
| KLHL24 | AL132989.1 | 0.351390018 | 7.35E-06 |
| MAPK8 | AL132989.1 | 0.345299199 | 1.08E-05 |
| MTOR | AL132989.1 | 0.395994701 | 3.39E-07 |
| NBR1 | AL132989.1 | 0.325645125 | 3.55E-05 |
| NKX2-3 | AL132989.1 | 0.315882275 | 6.23E-05 |
| PARP1 | AL132989.1 | 0.32259581 | 4.24E-05 |
| PIK3R4 | AL132989.1 | 0.306524051 | 0.000104843 |
| RAF1 | AL132989.1 | 0.413667303 | 8.79E-08 |
| SIRT1 | AL132989.1 | 0.463039864 | 1.31E-09 |
| TP53 | AL132989.1 | 0.366146811 | 2.79E-06 |
| TP73 | AL132989.1 | 0.428596688 | 2.64E-08 |
| TSC1 | AL132989.1 | 0.432009906 | 1.99E-08 |
| TSC2 | AL132989.1 | 0.465939895 | 9.99E-10 |
| UVRAG | AL132989.1 | 0.400516534 | 2.42E-07 |
| AMBRA1 | CTBP1-DT | 0.335958125 | 1.92E-05 |
| ARNT | CTBP1-DT | 0.461251104 | 1.54E-09 |
| ARSB | CTBP1-DT | 0.417965973 | 6.26E-08 |
| ATG2B | CTBP1-DT | 0.407953031 | 1.37E-07 |
| ATG4C | CTBP1-DT | 0.37885904 | 1.17E-06 |
| BECN1 | CTBP1-DT | 0.322088213 | 4.37E-05 |
| BIRC6 | CTBP1-DT | 0.393418067 | 4.10E-07 |
| DAPK1 | CTBP1-DT | 0.404957703 | 1.73E-07 |
| EDEM1 | CTBP1-DT | 0.320936064 | 4.67E-05 |
| EEF2K | CTBP1-DT | 0.374636329 | 1.57E-06 |
| EIF2AK3 | CTBP1-DT | 0.324563308 | 3.79E-05 |
| FOXO3 | CTBP1-DT | 0.307844636 | 9.75E-05 |
| HDAC6 | CTBP1-DT | 0.411103402 | 1.07E-07 |
| IKBKE | CTBP1-DT | 0.36545546 | 2.93E-06 |
| ITPR1 | CTBP1-DT | 0.300481523 | 0.000145367 |
| KLHL24 | CTBP1-DT | 0.31013605 | 8.59E-05 |
| MAPK1 | CTBP1-DT | 0.318789985 | 5.28E-05 |
| MBTPS2 | CTBP1-DT | 0.319120227 | 5.18E-05 |
| MTOR | CTBP1-DT | 0.394713523 | 3.73E-07 |
| NAF1 | CTBP1-DT | 0.359051813 | 4.48E-06 |
| NBR1 | CTBP1-DT | 0.376114293 | 1.41E-06 |
| PARP1 | CTBP1-DT | 0.40866121 | 1.30E-07 |
| PIK3R4 | CTBP1-DT | 0.357953072 | 4.81E-06 |
| RAB33B | CTBP1-DT | 0.308876256 | 9.21E-05 |
| RAF1 | CTBP1-DT | 0.314145774 | 6.87E-05 |
| RPS6KB1 | CTBP1-DT | 0.315382844 | 6.41E-05 |
| SIRT1 | CTBP1-DT | 0.301907292 | 0.000134665 |
| TP73 | CTBP1-DT | 0.381837367 | 9.47E-07 |
| TSC2 | CTBP1-DT | 0.308678914 | 9.31E-05 |
| ULK2 | CTBP1-DT | 0.319661705 | 5.03E-05 |
| UVRAG | CTBP1-DT | 0.306546186 | 0.000104716 |
| WDFY3 | CTBP1-DT | 0.335052847 | 2.03E-05 |
| ATG16L2 | AC022150.2 | 0.310974993 | 8.20E-05 |
| DAPK2 | AC022150.2 | 0.330045893 | 2.74E-05 |
| TSC1 | AC022150.2 | 0.329370553 | 2.85E-05 |
| ATG16L2 | AC069281.2 | 0.493985323 | 6.50E-11 |
| ATG2B | AC069281.2 | -0.309697878 | 8.81E-05 |
| ATG4B | AC069281.2 | 0.59062985 | 6.02E-16 |
| ATG4D | AC069281.2 | 0.304528971 | 0.00011688 |
| BAX | AC069281.2 | 0.444057004 | 7.14E-09 |
| BID | AC069281.2 | 0.404128239 | 1.84E-07 |
| BNIP1 | AC069281.2 | 0.479347895 | 2.79E-10 |
| CAPN10 | AC069281.2 | 0.548898862 | 1.42E-13 |
| CAPNS1 | AC069281.2 | 0.409007916 | 1.26E-07 |
| CDKN2A | AC069281.2 | 0.345089452 | 1.10E-05 |
| CLN3 | AC069281.2 | 0.420196079 | 5.23E-08 |
| CXCR4 | AC069281.2 | 0.326381585 | 3.40E-05 |
| DAPK2 | AC069281.2 | 0.306798485 | 0.000103281 |
| EIF4G1 | AC069281.2 | -0.361073964 | 3.92E-06 |
| FKBP1A | AC069281.2 | 0.340814549 | 1.43E-05 |
| GABARAP | AC069281.2 | 0.337155307 | 1.79E-05 |
| GABARAPL2 | AC069281.2 | 0.309502624 | 8.90E-05 |
| HIF1A | AC069281.2 | -0.355305802 | 5.72E-06 |
| ITGA6 | AC069281.2 | -0.302975447 | 0.000127134 |
| KIF5B | AC069281.2 | -0.401064673 | 2.32E-07 |
| LAMP2 | AC069281.2 | -0.312457497 | 7.55E-05 |
| MAP1LC3A | AC069281.2 | 0.397723328 | 2.98E-07 |
| MAPK1 | AC069281.2 | -0.347722477 | 9.28E-06 |
| MBTPS2 | AC069281.2 | -0.377538939 | 1.28E-06 |
| MTMR14 | AC069281.2 | 0.359191671 | 4.44E-06 |
| NCKAP1 | AC069281.2 | -0.420565611 | 5.08E-08 |
| PEX14 | AC069281.2 | 0.444270516 | 7.01E-09 |
| RAB24 | AC069281.2 | 0.512441397 | 9.36E-12 |
| STK11 | AC069281.2 | 0.435308353 | 1.51E-08 |
| TM9SF1 | AC069281.2 | -0.303213616 | 0.000125509 |
| TP63 | AC069281.2 | -0.304591267 | 0.000116485 |
| WDFY3 | AC069281.2 | -0.369123881 | 2.29E-06 |
| WDR45 | AC069281.2 | 0.418179085 | 6.15E-08 |
| ATG9B | AC022075.2 | 0.346303019 | 1.02E-05 |
| BID | AC022075.2 | 0.407406387 | 1.43E-07 |
| ARSA | Z83851.1 | 0.331571267 | 2.50E-05 |
| EIF4G1 | Z83851.1 | 0.391885285 | 4.59E-07 |
| MAPK1 | Z83851.1 | 0.336258953 | 1.89E-05 |
| CASP1 | LINC00707 | 0.333586958 | 2.22E-05 |
| CASP4 | LINC00707 | 0.482900612 | 1.97E-10 |
| ITGA3 | LINC00707 | 0.443145785 | 7.73E-09 |
| ITGA6 | LINC00707 | 0.376889155 | 1.34E-06 |
| PARP1 | LINC00707 | -0.306548762 | 0.000104701 |
| RAC1 | LINC00707 | 0.313062887 | 7.30E-05 |
| ARNT | AL441992.1 | -0.360742039 | 4.01E-06 |
| ATF6 | AL441992.1 | -0.374728805 | 1.56E-06 |
| BAX | AL441992.1 | 0.376107096 | 1.42E-06 |
| BIRC5 | AL441992.1 | 0.462411853 | 1.38E-09 |
| BIRC6 | AL441992.1 | -0.424350715 | 3.74E-08 |
| BNIP1 | AL441992.1 | 0.362179212 | 3.64E-06 |
| CDKN2A | AL441992.1 | 0.339845163 | 1.52E-05 |
| DLC1 | AL441992.1 | -0.332553323 | 2.36E-05 |
| FKBP1A | AL441992.1 | 0.340745133 | 1.43E-05 |
| FOXO3 | AL441992.1 | -0.356480799 | 5.30E-06 |
| GABARAPL2 | AL441992.1 | 0.334401337 | 2.11E-05 |
| GOPC | AL441992.1 | -0.310898205 | 8.24E-05 |
| HGS | AL441992.1 | -0.319545284 | 5.06E-05 |
| KIF5B | AL441992.1 | -0.351350919 | 7.37E-06 |
| MAPK1 | AL441992.1 | -0.370017319 | 2.15E-06 |
| NAF1 | AL441992.1 | -0.33437974 | 2.12E-05 |
| NBR1 | AL441992.1 | -0.300628679 | 0.000144226 |
| NCKAP1 | AL441992.1 | -0.464596785 | 1.13E-09 |
| PEX14 | AL441992.1 | 0.397352857 | 3.07E-07 |
| SPNS1 | AL441992.1 | -0.305589991 | 0.000110326 |
| UVRAG | AL441992.1 | -0.314687965 | 6.67E-05 |
| WDFY3 | AL441992.1 | -0.386070836 | 7.00E-07 |
| WDR45 | AL441992.1 | 0.357370629 | 5.00E-06 |
| ATG16L2 | NEAT1 | 0.528420083 | 1.59E-12 |
| ATG9B | NEAT1 | 0.306407562 | 0.000105512 |
| CAPN10 | NEAT1 | 0.334497993 | 2.10E-05 |
| RAB24 | NEAT1 | 0.318798334 | 5.28E-05 |
| VEGFA | NEAT1 | 0.312850932 | 7.39E-05 |
| ATG3 | C5orf66-AS1 | 0.468491144 | 7.87E-10 |
| ATG4A | C5orf66-AS1 | 0.342472684 | 1.29E-05 |
| ATG4B | C5orf66-AS1 | 0.446386081 | 5.83E-09 |
| ATG4D | C5orf66-AS1 | 0.387306801 | 6.40E-07 |
| BAG1 | C5orf66-AS1 | 0.312567613 | 7.51E-05 |
| BAX | C5orf66-AS1 | 0.454118385 | 2.94E-09 |
| BIRC5 | C5orf66-AS1 | 0.301852801 | 0.00013506 |
| BNIP1 | C5orf66-AS1 | 0.391156708 | 4.84E-07 |
| CAPN10 | C5orf66-AS1 | 0.336037201 | 1.91E-05 |
| CAPNS1 | C5orf66-AS1 | 0.415559241 | 7.57E-08 |
| CDKN2A | C5orf66-AS1 | 0.555080691 | 6.62E-14 |
| CLN3 | C5orf66-AS1 | 0.446636988 | 5.70E-09 |
| ERBB2 | C5orf66-AS1 | 0.301207953 | 0.000139819 |
| GOPC | C5orf66-AS1 | -0.345306276 | 1.08E-05 |
| HDAC6 | C5orf66-AS1 | 0.323886226 | 3.94E-05 |
| IKBKE | C5orf66-AS1 | 0.40450735 | 1.79E-07 |
| MAP2K7 | C5orf66-AS1 | 0.322998542 | 4.15E-05 |
| MTMR14 | C5orf66-AS1 | 0.369963461 | 2.16E-06 |
| PEX14 | C5orf66-AS1 | 0.32225779 | 4.33E-05 |
| RAB24 | C5orf66-AS1 | 0.435925032 | 1.43E-08 |
| RAC1 | C5orf66-AS1 | 0.306769242 | 0.000103446 |
| STK11 | C5orf66-AS1 | 0.351924742 | 7.11E-06 |
| WDR45 | C5orf66-AS1 | 0.524416968 | 2.50E-12 |
| ATIC | AC108860.2 | 0.479824426 | 2.66E-10 |
| BAK1 | AC108860.2 | -0.322731696 | 4.21E-05 |
| BIRC5 | AC108860.2 | 0.417932207 | 6.27E-08 |
| CASP4 | AC108860.2 | -0.301908225 | 0.000134658 |
| CDKN1B | AC108860.2 | 0.316397304 | 6.05E-05 |
| GABARAPL2 | AC108860.2 | 0.328620232 | 2.98E-05 |
| HDAC6 | AC108860.2 | 0.45498882 | 2.72E-09 |
| ITGA3 | AC108860.2 | -0.397285877 | 3.08E-07 |
| ITGA6 | AC108860.2 | -0.301486129 | 0.000137747 |
| ITGB4 | AC108860.2 | -0.354675026 | 5.95E-06 |
| MAPK8IP1 | AC108860.2 | 0.670064482 | 1.51E-21 |
| PRKAR1A | AC108860.2 | 0.487707564 | 1.22E-10 |
| VAMP7 | AC108860.2 | 0.365323726 | 2.95E-06 |
| ATIC | AL139289.2 | 0.44267691 | 8.05E-09 |
| BAX | AL139289.2 | 0.366634628 | 2.70E-06 |
| BIRC5 | AL139289.2 | 0.41238161 | 9.73E-08 |
| CAPN10 | AL139289.2 | 0.37079697 | 2.04E-06 |
| CAPN2 | AL139289.2 | -0.302905314 | 0.000127616 |
| CHMP4B | AL139289.2 | 0.315078866 | 6.52E-05 |
| DIRAS3 | AL139289.2 | 0.445649006 | 6.22E-09 |
| FKBP1A | AL139289.2 | 0.388582776 | 5.84E-07 |
| FKBP1B | AL139289.2 | 0.400355748 | 2.45E-07 |
| GABARAPL2 | AL139289.2 | 0.325639722 | 3.56E-05 |
| MAPK1 | AL139289.2 | -0.325791457 | 3.52E-05 |
| MAPK8IP1 | AL139289.2 | 0.462079201 | 1.43E-09 |
| MLST8 | AL139289.2 | 0.331335587 | 2.54E-05 |
| ATF4 | U62317.4 | 0.401850479 | 2.19E-07 |
| BAX | U62317.4 | 0.346524134 | 1.00E-05 |
| BIRC6 | U62317.4 | -0.370030961 | 2.15E-06 |
| BNIP1 | U62317.4 | 0.46095749 | 1.58E-09 |
| DDIT3 | U62317.4 | 0.667512315 | 2.43E-21 |
| GOPC | U62317.4 | -0.333622851 | 2.21E-05 |
| MAPK1 | U62317.4 | -0.341044513 | 1.41E-05 |
| NAF1 | U62317.4 | -0.303404163 | 0.000124224 |
| NCKAP1 | U62317.4 | -0.343213605 | 1.23E-05 |
| SESN2 | U62317.4 | 0.342142626 | 1.32E-05 |
| USP10 | U62317.4 | -0.338031889 | 1.69E-05 |
| WDFY3 | U62317.4 | -0.305019995 | 0.000113803 |
| ATG2A | DTX2P1-UPK3BP1-PMS2P11 | 0.337515287 | 1.75E-05 |
| ATG3 | DTX2P1-UPK3BP1-PMS2P11 | 0.404220462 | 1.83E-07 |
| ATG4C | DTX2P1-UPK3BP1-PMS2P11 | 0.324546838 | 3.79E-05 |
| ATG4D | DTX2P1-UPK3BP1-PMS2P11 | 0.353264314 | 6.52E-06 |
| ATG7 | DTX2P1-UPK3BP1-PMS2P11 | 0.343965153 | 1.18E-05 |
| BCL2 | DTX2P1-UPK3BP1-PMS2P11 | 0.393022944 | 4.22E-07 |
| BECN1 | DTX2P1-UPK3BP1-PMS2P11 | 0.307490775 | 9.94E-05 |
| CAPN10 | DTX2P1-UPK3BP1-PMS2P11 | 0.353150779 | 6.57E-06 |
| CD46 | DTX2P1-UPK3BP1-PMS2P11 | 0.354297087 | 6.10E-06 |
| CDKN1B | DTX2P1-UPK3BP1-PMS2P11 | 0.473451187 | 4.92E-10 |
| CDKN2A | DTX2P1-UPK3BP1-PMS2P11 | 0.334930379 | 2.05E-05 |
| CFLAR | DTX2P1-UPK3BP1-PMS2P11 | 0.415236614 | 7.77E-08 |
| CLN3 | DTX2P1-UPK3BP1-PMS2P11 | 0.300886046 | 0.000142252 |
| CXCR4 | DTX2P1-UPK3BP1-PMS2P11 | 0.401412862 | 2.26E-07 |
| EEF2K | DTX2P1-UPK3BP1-PMS2P11 | 0.355712372 | 5.57E-06 |
| EIF2AK3 | DTX2P1-UPK3BP1-PMS2P11 | 0.398438384 | 2.83E-07 |
| EIF2S1 | DTX2P1-UPK3BP1-PMS2P11 | -0.368802758 | 2.34E-06 |
| FOXO3 | DTX2P1-UPK3BP1-PMS2P11 | 0.340912423 | 1.42E-05 |
| HDAC6 | DTX2P1-UPK3BP1-PMS2P11 | 0.553984584 | 7.59E-14 |
| HSPA8 | DTX2P1-UPK3BP1-PMS2P11 | -0.308744926 | 9.28E-05 |
| IKBKB | DTX2P1-UPK3BP1-PMS2P11 | 0.563522142 | 2.28E-14 |
| IKBKE | DTX2P1-UPK3BP1-PMS2P11 | 0.434153054 | 1.66E-08 |
| ITGA3 | DTX2P1-UPK3BP1-PMS2P11 | -0.33543655 | 1.98E-05 |
| ITGA6 | DTX2P1-UPK3BP1-PMS2P11 | -0.314676944 | 6.67E-05 |
| KLHL24 | DTX2P1-UPK3BP1-PMS2P11 | 0.390979368 | 4.91E-07 |
| MAP2K7 | DTX2P1-UPK3BP1-PMS2P11 | 0.463583587 | 1.24E-09 |
| MAPK8 | DTX2P1-UPK3BP1-PMS2P11 | 0.382026674 | 9.34E-07 |
| MTMR14 | DTX2P1-UPK3BP1-PMS2P11 | 0.331721764 | 2.48E-05 |
| NBR1 | DTX2P1-UPK3BP1-PMS2P11 | 0.375453434 | 1.48E-06 |
| NFE2L2 | DTX2P1-UPK3BP1-PMS2P11 | 0.372354248 | 1.83E-06 |
| NKX2-3 | DTX2P1-UPK3BP1-PMS2P11 | 0.434906356 | 1.56E-08 |
| PARP1 | DTX2P1-UPK3BP1-PMS2P11 | 0.422426764 | 4.37E-08 |
| PIK3R4 | DTX2P1-UPK3BP1-PMS2P11 | 0.450524904 | 4.05E-09 |
| PINK1 | DTX2P1-UPK3BP1-PMS2P11 | -0.325346563 | 3.62E-05 |
| PRKAB1 | DTX2P1-UPK3BP1-PMS2P11 | 0.535820985 | 6.76E-13 |
| PTK6 | DTX2P1-UPK3BP1-PMS2P11 | -0.305033428 | 0.00011372 |
| RAF1 | DTX2P1-UPK3BP1-PMS2P11 | 0.460248174 | 1.69E-09 |
| RHEB | DTX2P1-UPK3BP1-PMS2P11 | 0.327299989 | 3.23E-05 |
| STK11 | DTX2P1-UPK3BP1-PMS2P11 | 0.416497349 | 7.03E-08 |
| TP53 | DTX2P1-UPK3BP1-PMS2P11 | 0.390293145 | 5.16E-07 |
| TP73 | DTX2P1-UPK3BP1-PMS2P11 | 0.441868784 | 8.63E-09 |
| TSC2 | DTX2P1-UPK3BP1-PMS2P11 | 0.52028359 | 3.96E-12 |
| ULK3 | DTX2P1-UPK3BP1-PMS2P11 | 0.353628431 | 6.37E-06 |
| WDR45 | DTX2P1-UPK3BP1-PMS2P11 | 0.326157981 | 3.45E-05 |
| ATG3 | NCK1-DT | 0.427501103 | 2.89E-08 |
| ATG4D | NCK1-DT | 0.401569329 | 2.23E-07 |
| BID | NCK1-DT | 0.340866358 | 1.42E-05 |
| CAPN10 | NCK1-DT | 0.331013384 | 2.59E-05 |
| CLN3 | NCK1-DT | 0.332765451 | 2.33E-05 |
| DLC1 | NCK1-DT | -0.312161627 | 7.68E-05 |
| IKBKE | NCK1-DT | 0.335317754 | 2.00E-05 |
| ITGA3 | NCK1-DT | -0.314882943 | 6.59E-05 |
| MAPK8 | NCK1-DT | 0.339066401 | 1.59E-05 |
| PIK3R4 | NCK1-DT | 0.310004619 | 8.66E-05 |
| PINK1 | NCK1-DT | -0.36910665 | 2.29E-06 |
| PRKAB1 | NCK1-DT | 0.323120415 | 4.12E-05 |
| RAF1 | NCK1-DT | 0.365786179 | 2.86E-06 |
| ULK3 | NCK1-DT | 0.322248632 | 4.33E-05 |
| ATG4B | AC027796.4 | 0.301803362 | 0.000135419 |
| EGFR | AC027796.4 | 0.396708047 | 3.22E-07 |
| PELP1 | AC027796.4 | 0.376536226 | 1.37E-06 |
| VEGFA | AC027796.4 | 0.339153152 | 1.58E-05 |
| EIF4G1 | AL590666.2 | 0.349738661 | 8.17E-06 |
| KIF5B | AL590666.2 | 0.368758046 | 2.34E-06 |
| NBR1 | AL590666.2 | 0.336786714 | 1.83E-05 |
| PARP1 | AL590666.2 | 0.322674934 | 4.22E-05 |
| SQSTM1 | AL590666.2 | 0.443951628 | 7.21E-09 |
| ATG3 | MUC20-OT1 | 0.468237002 | 8.06E-10 |
| CDKN2A | MUC20-OT1 | 0.301071808 | 0.000140843 |
| EIF2AK3 | MUC20-OT1 | 0.399031911 | 2.70E-07 |
| HDAC1 | MUC20-OT1 | 0.314281786 | 6.82E-05 |
| HDAC6 | MUC20-OT1 | 0.3542983 | 6.10E-06 |
| IKBKB | MUC20-OT1 | 0.320119084 | 4.90E-05 |
| IKBKE | MUC20-OT1 | 0.338512947 | 1.65E-05 |
| KLHL24 | MUC20-OT1 | 0.409300889 | 1.24E-07 |
| MAP2K7 | MUC20-OT1 | 0.466750776 | 9.26E-10 |
| PIK3R4 | MUC20-OT1 | 0.397777667 | 2.97E-07 |
| PINK1 | MUC20-OT1 | -0.42182745 | 4.59E-08 |
| RAF1 | MUC20-OT1 | 0.421642682 | 4.66E-08 |
| STK11 | MUC20-OT1 | 0.384528551 | 7.82E-07 |
| TSC1 | MUC20-OT1 | 0.359875096 | 4.24E-06 |
| ATG16L2 | LINC00526 | 0.398030614 | 2.92E-07 |
| ATG4C | LINC00526 | 0.316026738 | 6.18E-05 |
| CAPN10 | LINC00526 | 0.364513554 | 3.12E-06 |
| CDKN1B | LINC00526 | 0.362553457 | 3.55E-06 |
| FOXO1 | LINC00526 | -0.309740131 | 8.78E-05 |
| HDAC1 | LINC00526 | 0.303563516 | 0.000123158 |
| HDAC6 | LINC00526 | 0.309288526 | 9.01E-05 |
| MAPK8 | LINC00526 | 0.340526422 | 1.45E-05 |
| MAPK8IP1 | LINC00526 | 0.30869033 | 9.31E-05 |
| MLST8 | LINC00526 | 0.344715699 | 1.12E-05 |
| PEA15 | LINC00526 | -0.300380985 | 0.00014615 |
| ULK3 | LINC00526 | 0.316410652 | 6.05E-05 |
| EGFR | AL121790.2 | 0.392596266 | 4.36E-07 |
| ATF4 | AC007038.2 | 0.350474918 | 7.80E-06 |
| ATG12 | AC007038.2 | 0.333047799 | 2.29E-05 |
| ATG16L2 | AC007038.2 | 0.515042268 | 7.05E-12 |
| ATG3 | AC007038.2 | 0.35625659 | 5.37E-06 |
| ATG4B | AC007038.2 | 0.541171271 | 3.60E-13 |
| ATG4C | AC007038.2 | 0.370244126 | 2.12E-06 |
| ATIC | AC007038.2 | 0.40364936 | 1.91E-07 |
| BAG3 | AC007038.2 | -0.352632544 | 6.79E-06 |
| BAX | AC007038.2 | 0.495254867 | 5.71E-11 |
| BIRC5 | AC007038.2 | 0.388312174 | 5.96E-07 |
| BNIP1 | AC007038.2 | 0.367789591 | 2.50E-06 |
| CAPN10 | AC007038.2 | 0.704128773 | 1.61E-24 |
| CDKN2A | AC007038.2 | 0.304935238 | 0.000114329 |
| CLN3 | AC007038.2 | 0.333475568 | 2.23E-05 |
| CXCR4 | AC007038.2 | 0.449070466 | 4.61E-09 |
| DAPK2 | AC007038.2 | 0.384958607 | 7.58E-07 |
| EIF4G1 | AC007038.2 | -0.40703228 | 1.47E-07 |
| FKBP1A | AC007038.2 | 0.314450431 | 6.76E-05 |
| GABARAP | AC007038.2 | 0.303552072 | 0.000123234 |
| HDAC6 | AC007038.2 | 0.40013117 | 2.49E-07 |
| HIF1A | AC007038.2 | -0.326563914 | 3.37E-05 |
| IKBKB | AC007038.2 | 0.323665508 | 3.99E-05 |
| KIF5B | AC007038.2 | -0.328175778 | 3.06E-05 |
| LAMP2 | AC007038.2 | -0.300223685 | 0.000147385 |
| MAP2K7 | AC007038.2 | 0.340270252 | 1.48E-05 |
| MAPK1 | AC007038.2 | -0.351676532 | 7.22E-06 |
| MAPK8IP1 | AC007038.2 | 0.350036438 | 8.02E-06 |
| MLST8 | AC007038.2 | 0.377552421 | 1.28E-06 |
| MTMR14 | AC007038.2 | 0.390813009 | 4.97E-07 |
| NCKAP1 | AC007038.2 | -0.362521556 | 3.56E-06 |
| PEX14 | AC007038.2 | 0.327580983 | 3.17E-05 |
| PINK1 | AC007038.2 | -0.345325562 | 1.08E-05 |
| RAB24 | AC007038.2 | 0.420836196 | 4.97E-08 |
| STK11 | AC007038.2 | 0.436190535 | 1.40E-08 |
| TP63 | AC007038.2 | -0.304574492 | 0.000116592 |
| TSC2 | AC007038.2 | 0.310846121 | 8.26E-05 |
| ULK3 | AC007038.2 | 0.488303743 | 1.15E-10 |
| USP10 | AC007038.2 | -0.337096166 | 1.79E-05 |
| WDFY3 | AC007038.2 | -0.35068728 | 7.69E-06 |
| WDR45 | AC007038.2 | 0.512371607 | 9.43E-12 |
| ARSB | AC018695.6 | -0.357346326 | 5.01E-06 |
| ATG4B | AC018695.6 | 0.404236812 | 1.82E-07 |
| CASP1 | AC018695.6 | 0.431117687 | 2.14E-08 |
| CASP4 | AC018695.6 | 0.371578569 | 1.93E-06 |
| SPHK1 | AL139393.2 | 0.345610314 | 1.06E-05 |
| ATG16L2 | AC156455.1 | 0.359613185 | 4.32E-06 |
| ATIC | AC156455.1 | 0.425087003 | 3.52E-08 |
| BIRC5 | AC156455.1 | 0.351436975 | 7.33E-06 |
| FOXO1 | AC156455.1 | -0.357320899 | 5.01E-06 |
| MAPK8IP1 | AC156455.1 | 0.678888012 | 2.80E-22 |
| PRKAR1A | AC156455.1 | 0.4291166 | 2.53E-08 |
| ATG4B | AL023803.2 | 0.360628094 | 4.04E-06 |
| BAX | AL023803.2 | 0.42716283 | 2.97E-08 |
| BIRC5 | AL023803.2 | 0.454264639 | 2.90E-09 |
| CAPN10 | AL023803.2 | 0.325287397 | 3.63E-05 |
| FKBP1A | AL023803.2 | 0.358210973 | 4.73E-06 |
| GABARAP | AL023803.2 | 0.300123353 | 0.000148177 |
| PELP1 | AL023803.2 | 0.430922707 | 2.18E-08 |
| PINK1 | AL023803.2 | -0.339341135 | 1.56E-05 |
| WDR45 | AL023803.2 | 0.396629659 | 3.24E-07 |
| ATF4 | PXN-AS1 | 0.328861034 | 2.94E-05 |
| ATG10 | PXN-AS1 | 0.390980577 | 4.91E-07 |
| ATG4B | PXN-AS1 | 0.39110936 | 4.86E-07 |
| ATG4D | PXN-AS1 | 0.3033469 | 0.000124609 |
| ATIC | PXN-AS1 | 0.301125934 | 0.000140435 |
| BAG1 | PXN-AS1 | 0.323310007 | 4.07E-05 |
| BAG3 | PXN-AS1 | -0.337404017 | 1.76E-05 |
| BAX | PXN-AS1 | 0.562901576 | 2.47E-14 |
| BIRC5 | PXN-AS1 | 0.589391398 | 7.16E-16 |
| BNIP1 | PXN-AS1 | 0.498792818 | 3.97E-11 |
| CAPN10 | PXN-AS1 | 0.440256885 | 9.91E-09 |
| CAPN2 | PXN-AS1 | -0.311204144 | 8.10E-05 |
| CAPNS1 | PXN-AS1 | 0.355360028 | 5.70E-06 |
| CDKN2A | PXN-AS1 | 0.412997082 | 9.27E-08 |
| CLN3 | PXN-AS1 | 0.342025846 | 1.33E-05 |
| CXCR4 | PXN-AS1 | 0.300290968 | 0.000146856 |
| EIF4G1 | PXN-AS1 | -0.339570275 | 1.54E-05 |
| FKBP1A | PXN-AS1 | 0.534645526 | 7.75E-13 |
| GABARAP | PXN-AS1 | 0.360228852 | 4.14E-06 |
| HDAC6 | PXN-AS1 | 0.374674289 | 1.56E-06 |
| HGS | PXN-AS1 | -0.302360825 | 0.000131418 |
| ITGA3 | PXN-AS1 | -0.310740136 | 8.31E-05 |
| ITGA6 | PXN-AS1 | -0.379222656 | 1.14E-06 |
| MAP1LC3A | PXN-AS1 | 0.328017313 | 3.09E-05 |
| MAPK8IP1 | PXN-AS1 | 0.384492184 | 7.84E-07 |
| MLST8 | PXN-AS1 | 0.418198921 | 6.14E-08 |
| MTMR14 | PXN-AS1 | 0.404346047 | 1.81E-07 |
| NCKAP1 | PXN-AS1 | -0.344440581 | 1.14E-05 |
| PEX14 | PXN-AS1 | 0.306518044 | 0.000104877 |
| PINK1 | PXN-AS1 | -0.341992493 | 1.33E-05 |
| PRKAB1 | PXN-AS1 | 0.335666352 | 1.96E-05 |
| RAB24 | PXN-AS1 | 0.373851989 | 1.65E-06 |
| USP10 | PXN-AS1 | -0.304385093 | 0.000117796 |
| WDFY3 | PXN-AS1 | -0.368221278 | 2.43E-06 |
| WDR45 | PXN-AS1 | 0.594338687 | 3.56E-16 |
| UVRAG | TSPEAR-AS1 | 0.312862702 | 7.38E-05 |
| BAK1 | AC105460.1 | 0.344586112 | 1.13E-05 |
| CASP1 | AC105460.1 | 0.486992364 | 1.31E-10 |
| CASP4 | AC105460.1 | 0.35386961 | 6.27E-06 |
| HSPA8 | AC105460.1 | 0.314163239 | 6.87E-05 |
| ATG4B | AC027307.2 | 0.475303519 | 4.12E-10 |
| ATIC | AC027307.2 | 0.354895452 | 5.87E-06 |
| BAX | AC027307.2 | 0.328302828 | 3.04E-05 |
| BNIP1 | AC027307.2 | 0.355217779 | 5.75E-06 |
| CANX | AC027307.2 | -0.307388746 | 1.00E-04 |
| CAPN10 | AC027307.2 | 0.314299417 | 6.81E-05 |
| CLN3 | AC027307.2 | 0.45180767 | 3.61E-09 |
| MLST8 | AC027307.2 | 0.355472 | 5.65E-06 |
| PEX14 | AC027307.2 | 0.367965524 | 2.47E-06 |
| STK11 | AC027307.2 | 0.566426584 | 1.57E-14 |
| ARNT | LINC01139 | 0.36404163 | 3.22E-06 |
| BNIP3L | LINC01139 | 0.304066715 | 0.000119847 |
| CD46 | LINC01139 | 0.35286721 | 6.69E-06 |
| CDKN1B | LINC01139 | 0.407395109 | 1.43E-07 |
| CX3CL1 | LINC01139 | 0.310194096 | 8.57E-05 |
| CXCR4 | LINC01139 | 0.367164566 | 2.61E-06 |
| DNAJB9 | LINC01139 | 0.414131811 | 8.48E-08 |
| EEF2K | LINC01139 | 0.398979311 | 2.72E-07 |
| FAS | LINC01139 | 0.360087721 | 4.18E-06 |
| HDAC6 | LINC01139 | 0.308793132 | 9.26E-05 |
| KLHL24 | LINC01139 | 0.336731922 | 1.83E-05 |
| MAPK8 | LINC01139 | 0.412722173 | 9.47E-08 |
| NBR1 | LINC01139 | 0.373951351 | 1.64E-06 |
| NKX2-3 | LINC01139 | 0.321483271 | 4.53E-05 |
| PARP1 | LINC01139 | 0.367387162 | 2.57E-06 |
| PRKAB1 | LINC01139 | 0.337253027 | 1.78E-05 |
| RAB33B | LINC01139 | 0.378953413 | 1.16E-06 |
| SIRT1 | LINC01139 | 0.514403206 | 7.56E-12 |
| TP53 | LINC01139 | 0.368467986 | 2.39E-06 |
| TP73 | LINC01139 | 0.389880424 | 5.32E-07 |
| TSC2 | LINC01139 | 0.347252023 | 9.56E-06 |
| ATG3 | ERICH6-AS1 | 0.370441748 | 2.09E-06 |
| ATG4A | ERICH6-AS1 | 0.407197708 | 1.45E-07 |
| ATIC | ERICH6-AS1 | 0.422184687 | 4.46E-08 |
| BIRC5 | ERICH6-AS1 | 0.497359498 | 4.60E-11 |
| BNIP1 | ERICH6-AS1 | 0.325860032 | 3.51E-05 |
| GABARAPL2 | ERICH6-AS1 | 0.325660322 | 3.55E-05 |
| HDAC6 | ERICH6-AS1 | 0.471293677 | 6.04E-10 |
| KLHL24 | ERICH6-AS1 | 0.311771355 | 7.85E-05 |
| MAPK8IP1 | ERICH6-AS1 | 0.558265288 | 4.45E-14 |
| PRKAR1A | ERICH6-AS1 | 0.390479494 | 5.09E-07 |
| ST13 | ERICH6-AS1 | 0.325891969 | 3.50E-05 |
| VAMP7 | ERICH6-AS1 | 0.321720815 | 4.46E-05 |
| WDR45 | ERICH6-AS1 | 0.397110004 | 3.12E-07 |
| CXCR4 | AF001548.2 | 0.30968856 | 8.81E-05 |
| DRAM1 | AF001548.2 | 0.328498168 | 3.01E-05 |
| MAPK8 | AF001548.2 | 0.370928338 | 2.02E-06 |
| NKX2-3 | AF001548.2 | 0.327235433 | 3.24E-05 |
| STK11 | AF001548.2 | 0.355705245 | 5.57E-06 |
| TP73 | AF001548.2 | 0.301346998 | 0.00013878 |
| TSC2 | AF001548.2 | 0.352051775 | 7.05E-06 |
| BIRC6 | AL137003.1 | 0.35209814 | 7.03E-06 |
| DAPK1 | AL137003.1 | 0.301281196 | 0.000139271 |
| EIF2AK3 | AL137003.1 | 0.337175133 | 1.79E-05 |
| FOXO3 | AL137003.1 | 0.369706167 | 2.20E-06 |
| GABARAPL1 | AL137003.1 | 0.321280353 | 4.58E-05 |
| KLHL24 | AL137003.1 | 0.302751953 | 0.000128677 |
| NCKAP1 | AL137003.1 | 0.321607427 | 4.49E-05 |
| NFE2L2 | AL137003.1 | 0.474632604 | 4.40E-10 |
| RB1CC1 | AL137003.1 | 0.381018619 | 1.00E-06 |
| SPNS1 | AL137003.1 | 0.32517775 | 3.65E-05 |
| TP63 | AL137003.1 | 0.347378409 | 9.49E-06 |
| UVRAG | AL137003.1 | 0.353985739 | 6.23E-06 |
| ATF4 | PIK3CD-AS2 | 0.300077115 | 0.000148543 |
| ATIC | PIK3CD-AS2 | 0.376130424 | 1.41E-06 |
| BCL2 | PIK3CD-AS2 | 0.331550372 | 2.51E-05 |
| FAS | PIK3CD-AS2 | 0.316769456 | 5.93E-05 |
| MAPK8IP1 | PIK3CD-AS2 | 0.416791633 | 6.87E-08 |
| PARP1 | PIK3CD-AS2 | 0.331953262 | 2.45E-05 |
| ST13 | PIK3CD-AS2 | 0.311658159 | 7.90E-05 |
| ATG2B | AC034231.1 | 0.314130518 | 6.88E-05 |
| ATG4B | AC020915.3 | 0.314549211 | 6.72E-05 |
| BAX | AC020915.3 | 0.330798288 | 2.62E-05 |
| BIRC6 | AC020915.3 | -0.320069645 | 4.91E-05 |
| BNIP1 | AC020915.3 | 0.358005769 | 4.79E-06 |
| CASP1 | AC020915.3 | 0.33503163 | 2.03E-05 |
| MAPK1 | AC020915.3 | -0.326081235 | 3.46E-05 |
| NPC1 | AC020915.3 | -0.323718975 | 3.98E-05 |
| PEX14 | AC020915.3 | 0.384398816 | 7.89E-07 |
| WDR45 | AC020915.3 | 0.411641754 | 1.03E-07 |
| ST13 | AC144831.1 | -0.310073075 | 8.62E-05 |
| ATG10 | AL589843.1 | 0.306756691 | 0.000103517 |
| BNIP1 | AL589843.1 | 0.415592048 | 7.56E-08 |
| CAPNS1 | AL589843.1 | 0.379695912 | 1.10E-06 |
| CDKN2A | AL589843.1 | 0.44259684 | 8.10E-09 |
| FKBP1A | AL589843.1 | 0.392365038 | 4.43E-07 |
| MAP1LC3A | AL589843.1 | 0.527070749 | 1.85E-12 |
| PEX14 | AL589843.1 | 0.316196466 | 6.12E-05 |
| PRKAB1 | AL589843.1 | 0.315932466 | 6.21E-05 |
| RAC1 | AL589843.1 | 0.315059629 | 6.53E-05 |
| WDR45 | AL589843.1 | 0.446064083 | 6.00E-09 |
| ATG4B | AP002360.1 | 0.300354964 | 0.000146354 |
| ATIC | AP002360.1 | 0.484545666 | 1.68E-10 |
| BAX | AP002360.1 | 0.382684419 | 8.92E-07 |
| BIRC5 | AP002360.1 | 0.560789419 | 3.23E-14 |
| BNIP1 | AP002360.1 | 0.361063238 | 3.92E-06 |
| CAPN10 | AP002360.1 | 0.322022647 | 4.39E-05 |
| CAPN2 | AP002360.1 | -0.371471823 | 1.95E-06 |
| CLN3 | AP002360.1 | 0.361708415 | 3.76E-06 |
| FKBP1A | AP002360.1 | 0.469941672 | 6.87E-10 |
| GABARAPL2 | AP002360.1 | 0.312303715 | 7.62E-05 |
| GNAI3 | AP002360.1 | -0.305583361 | 0.000110366 |
| ITGA3 | AP002360.1 | -0.329812448 | 2.78E-05 |
| MAP1LC3A | AP002360.1 | 0.301652682 | 0.00013652 |
| MAPK1 | AP002360.1 | -0.361936121 | 3.70E-06 |
| MAPK8IP1 | AP002360.1 | 0.541641456 | 3.40E-13 |
| MLST8 | AP002360.1 | 0.397331658 | 3.07E-07 |
| MTMR14 | AP002360.1 | 0.303888172 | 0.000121012 |
| NFKB1 | AP002360.1 | -0.319332921 | 5.12E-05 |
| PELP1 | AP002360.1 | 0.343902966 | 1.18E-05 |
| SH3GLB1 | AP002360.1 | -0.329257943 | 2.87E-05 |
| WDR45 | AP002360.1 | 0.455888518 | 2.51E-09 |
| ZFYVE1 | AP002360.1 | -0.311019602 | 8.18E-05 |
| CALCOCO2 | LINC02100 | 0.330523415 | 2.67E-05 |
| EEF2K | LINC02100 | 0.386717767 | 6.68E-07 |
| TP73 | LINC02100 | 0.414842333 | 8.02E-08 |
| TSC2 | LINC02100 | 0.328450477 | 3.01E-05 |
| GABARAPL1 | AC092803.2 | 0.33058092 | 2.66E-05 |
| BCL2L1 | AC092142.1 | 0.335491822 | 1.98E-05 |
| BID | AC092142.1 | 0.378932629 | 1.16E-06 |
| GAA | AC092142.1 | 0.385937954 | 7.07E-07 |
| PPP1R15A | AC092142.1 | 0.323162089 | 4.11E-05 |
| BAX | AC027644.3 | 0.425990015 | 3.27E-08 |
| BIRC5 | AC027644.3 | 0.31375744 | 7.02E-05 |
| CAPN2 | AC027644.3 | -0.349500965 | 8.30E-06 |
| EIF4EBP1 | AC027644.3 | 0.41980116 | 5.40E-08 |
| FKBP1A | AC027644.3 | 0.401161058 | 2.30E-07 |
| HGS | AC027644.3 | -0.307287432 | 0.000100551 |
| ITGA3 | AC027644.3 | -0.369915195 | 2.17E-06 |
| ITGA6 | AC027644.3 | -0.309503029 | 8.90E-05 |
| ITGB4 | AC027644.3 | -0.323495937 | 4.03E-05 |
| MAPK1 | AC027644.3 | -0.346177867 | 1.02E-05 |
| MTOR | AC027644.3 | -0.305953365 | 0.000108162 |
| NFKB1 | AC027644.3 | -0.406424976 | 1.54E-07 |
| RB1 | AC027644.3 | -0.303139569 | 0.000126012 |
| SH3GLB1 | AC027644.3 | -0.321834815 | 4.44E-05 |
| WDR45 | AC027644.3 | 0.344984547 | 1.10E-05 |
| ATG7 | AC022031.2 | -0.308286364 | 9.52E-05 |
| GABARAPL1 | AC022031.2 | 0.394613314 | 3.76E-07 |
| NFKB1 | AC022031.2 | -0.30227991 | 0.000131992 |
| WIPI1 | AC022031.2 | 0.312016404 | 7.74E-05 |
| BID | AC004034.1 | 0.327774577 | 3.14E-05 |
| CAPN10 | AC004034.1 | 0.348651286 | 8.75E-06 |
| DIRAS3 | AC004034.1 | 0.686506722 | 6.24E-23 |
| FKBP1B | AC004034.1 | 0.441866883 | 8.63E-09 |
| MAP1LC3A | AC004034.1 | 0.356019977 | 5.46E-06 |
| MLST8 | AC004034.1 | 0.311021933 | 8.18E-05 |
| CASP3 | AL161785.1 | 0.328135706 | 3.07E-05 |
| CFLAR | AL161785.1 | 0.392181618 | 4.49E-07 |
| IKBKE | AL161785.1 | 0.413954055 | 8.60E-08 |
| NKX2-3 | AL161785.1 | 0.311761262 | 7.85E-05 |
| SERPINA1 | AL161785.1 | 0.411489356 | 1.04E-07 |
| WDR45 | AL161785.1 | 0.51129285 | 1.06E-11 |
| ATG12 | AC114489.1 | -0.303314681 | 0.000124826 |
| ATG4C | AC114489.1 | -0.434921753 | 1.56E-08 |
| BCL2 | AC114489.1 | -0.312105364 | 7.70E-05 |
| CALCOCO2 | AC114489.1 | -0.305386199 | 0.000111558 |
| DRAM1 | AC114489.1 | -0.31946445 | 5.08E-05 |
| FAS | AC114489.1 | -0.380793545 | 1.02E-06 |
| HDAC6 | AC114489.1 | -0.315809387 | 6.26E-05 |
| MAP1LC3B | AC114489.1 | 0.408447639 | 1.32E-07 |
| MAPK8 | AC114489.1 | -0.322328478 | 4.31E-05 |
| MAPK9 | AC114489.1 | -0.377684492 | 1.27E-06 |
| MTMR14 | AC114489.1 | -0.342887087 | 1.26E-05 |
| TP63 | AC114489.1 | 0.306362242 | 0.000105774 |
| TP73 | AC114489.1 | -0.341123507 | 1.40E-05 |
| ATG10 | RAB11B-AS1 | 0.354808563 | 5.90E-06 |
| ATG2B | RAB11B-AS1 | -0.389611078 | 5.42E-07 |
| ATG4B | RAB11B-AS1 | 0.547289951 | 1.73E-13 |
| ATG4D | RAB11B-AS1 | 0.361593759 | 3.79E-06 |
| ATG7 | RAB11B-AS1 | 0.433684961 | 1.73E-08 |
| BAX | RAB11B-AS1 | 0.455881499 | 2.51E-09 |
| BID | RAB11B-AS1 | 0.377251285 | 1.31E-06 |
| BNIP1 | RAB11B-AS1 | 0.497539863 | 4.51E-11 |
| CAPN10 | RAB11B-AS1 | 0.422009584 | 4.52E-08 |
| CAPNS1 | RAB11B-AS1 | 0.521883795 | 3.32E-12 |
| CDKN2A | RAB11B-AS1 | 0.573435319 | 6.28E-15 |
| CLN3 | RAB11B-AS1 | 0.359021078 | 4.49E-06 |
| CTSD | RAB11B-AS1 | 0.31363011 | 7.07E-05 |
| EIF4G1 | RAB11B-AS1 | -0.301654233 | 0.000136509 |
| FKBP1A | RAB11B-AS1 | 0.456559787 | 2.36E-09 |
| GABARAP | RAB11B-AS1 | 0.368056119 | 2.46E-06 |
| GOPC | RAB11B-AS1 | -0.399820192 | 2.55E-07 |
| HIF1A | RAB11B-AS1 | -0.340198544 | 1.48E-05 |
| IKBKE | RAB11B-AS1 | 0.319783608 | 4.99E-05 |
| ITGB1 | RAB11B-AS1 | -0.302744207 | 0.00012873 |
| MAP1LC3A | RAB11B-AS1 | 0.493004508 | 7.18E-11 |
| MAPK1 | RAB11B-AS1 | -0.401658954 | 2.22E-07 |
| MTMR14 | RAB11B-AS1 | 0.490202681 | 9.53E-11 |
| NAF1 | RAB11B-AS1 | -0.311340196 | 8.04E-05 |
| NCKAP1 | RAB11B-AS1 | -0.399486559 | 2.61E-07 |
| PEX14 | RAB11B-AS1 | 0.43722771 | 1.28E-08 |
| PRKAB1 | RAB11B-AS1 | 0.363977974 | 3.23E-06 |
| RAB24 | RAB11B-AS1 | 0.411787153 | 1.02E-07 |
| RAC1 | RAB11B-AS1 | 0.397105602 | 3.12E-07 |
| STK11 | RAB11B-AS1 | 0.452007562 | 3.55E-09 |
| TM9SF1 | RAB11B-AS1 | -0.306169538 | 0.000106893 |
| USP10 | RAB11B-AS1 | -0.389110083 | 5.62E-07 |
| WDFY3 | RAB11B-AS1 | -0.314138131 | 6.87E-05 |
| WDR45 | RAB11B-AS1 | 0.433813523 | 1.71E-08 |
| ZFYVE1 | RAB11B-AS1 | -0.311245045 | 8.08E-05 |
| BAG1 | AL513165.1 | 0.322670908 | 4.23E-05 |
| BAX | AL513165.1 | 0.394368128 | 3.83E-07 |
| BIRC5 | AL513165.1 | 0.38814962 | 6.03E-07 |
| BNIP1 | AL513165.1 | 0.32251815 | 4.26E-05 |
| CXCR4 | AL513165.1 | 0.335039702 | 2.03E-05 |
| HIF1A | AL513165.1 | -0.324784215 | 3.74E-05 |
| MAP2K7 | AL513165.1 | 0.407041148 | 1.47E-07 |
| MTMR14 | AL513165.1 | 0.371411547 | 1.96E-06 |
| NKX2-3 | AL513165.1 | 0.304244522 | 0.000118698 |
| PELP1 | AL513165.1 | 0.36155886 | 3.80E-06 |
| PINK1 | AL513165.1 | -0.315827265 | 6.25E-05 |
| RAB1A | AL513165.1 | -0.366481063 | 2.73E-06 |
| STK11 | AL513165.1 | 0.38743519 | 6.35E-07 |
| TP53 | AL513165.1 | 0.335686604 | 1.95E-05 |
| WDFY3 | AL513165.1 | -0.300285617 | 0.000146898 |
| WDR45 | AL513165.1 | 0.318361724 | 5.41E-05 |
| ATG16L2 | AP005329.2 | 0.369747089 | 2.19E-06 |
| BID | AP005329.2 | 0.340379206 | 1.47E-05 |
| DAPK2 | AP005329.2 | 0.404822999 | 1.75E-07 |
| FOXO1 | AP005329.2 | -0.306031902 | 0.000107699 |
| PELP1 | AP005329.2 | 0.419834073 | 5.39E-08 |
| ARSB | MIR100HG | 0.401526238 | 2.24E-07 |
| CCL2 | MIR100HG | 0.387427088 | 6.35E-07 |
| CXCR4 | MIR100HG | 0.328836785 | 2.95E-05 |
| DLC1 | MIR100HG | 0.320263732 | 4.85E-05 |
| DRAM1 | MIR100HG | 0.320620607 | 4.76E-05 |
| FAS | MIR100HG | 0.324945751 | 3.70E-05 |
| SERPINA1 | MIR100HG | 0.363047682 | 3.44E-06 |
| ARNT | AC026401.3 | -0.335081826 | 2.03E-05 |
| ATF4 | AC026401.3 | 0.382142962 | 9.26E-07 |
| ATF6 | AC026401.3 | -0.344118846 | 1.16E-05 |
| ATG2B | AC026401.3 | -0.392959284 | 4.24E-07 |
| ATG4B | AC026401.3 | 0.501279774 | 3.06E-11 |
| BAX | AC026401.3 | 0.392934691 | 4.25E-07 |
| BIRC5 | AC026401.3 | 0.386379475 | 6.85E-07 |
| BIRC6 | AC026401.3 | -0.36254476 | 3.56E-06 |
| BNIP1 | AC026401.3 | 0.428251631 | 2.72E-08 |
| CANX | AC026401.3 | -0.332007974 | 2.44E-05 |
| CAPN10 | AC026401.3 | 0.388401828 | 5.92E-07 |
| CDKN2A | AC026401.3 | 0.309283787 | 9.01E-05 |
| DAPK1 | AC026401.3 | -0.308716692 | 9.30E-05 |
| DLC1 | AC026401.3 | -0.326714505 | 3.34E-05 |
| FKBP1A | AC026401.3 | 0.303376693 | 0.000124408 |
| FOXO1 | AC026401.3 | -0.31063608 | 8.36E-05 |
| KIF5B | AC026401.3 | -0.308031063 | 9.65E-05 |
| MBTPS2 | AC026401.3 | -0.309909553 | 8.70E-05 |
| MLST8 | AC026401.3 | 0.364094327 | 3.21E-06 |
| NBR1 | AC026401.3 | -0.375686301 | 1.46E-06 |
| NCKAP1 | AC026401.3 | -0.361995824 | 3.69E-06 |
| PEX14 | AC026401.3 | 0.301520628 | 0.000137492 |
| PINK1 | AC026401.3 | -0.31021108 | 8.56E-05 |
| PPP1R15A | AC026401.3 | 0.307759813 | 9.80E-05 |
| RAB24 | AC026401.3 | 0.331142093 | 2.57E-05 |
| STK11 | AC026401.3 | 0.360408391 | 4.10E-06 |
| WDFY3 | AC026401.3 | -0.356713757 | 5.22E-06 |
| ZFYVE1 | AC026401.3 | -0.322943425 | 4.16E-05 |
| ATF4 | AC009403.1 | 0.361777004 | 3.74E-06 |
| ATG2A | AC009403.1 | -0.319172304 | 5.17E-05 |
| CAPN1 | AC009403.1 | -0.307266382 | 0.000100667 |
| CAPN2 | AC009403.1 | -0.348259592 | 8.97E-06 |
| DDIT3 | AC009403.1 | 0.303623628 | 0.000122758 |
| DIRAS3 | AC009403.1 | 0.343045295 | 1.24E-05 |
| MAPK8IP1 | AC009403.1 | 0.302547142 | 0.000130105 |
| NFKB1 | AC009403.1 | -0.32456577 | 3.79E-05 |
| RHEB | AC009403.1 | 0.443148211 | 7.73E-09 |
| SH3GLB1 | AC009403.1 | -0.352735969 | 6.75E-06 |
| ATG4C | AL359915.2 | 0.394987038 | 3.65E-07 |
| CALCOCO2 | AL359915.2 | 0.313558399 | 7.10E-05 |
| FAS | AL359915.2 | 0.347827012 | 9.22E-06 |
| MAPK3 | AL359915.2 | -0.301855674 | 0.000135039 |
| MAPK9 | AL359915.2 | 0.302255911 | 0.000132162 |
| PARP1 | AL359915.2 | 0.401431148 | 2.26E-07 |
| MAP1LC3A | LINC02253 | 0.306734275 | 0.000103644 |
| DNAJB9 | AC092953.2 | 0.33296241 | 2.30E-05 |
| EIF2AK3 | AC092953.2 | 0.306876219 | 0.000102842 |
| MAPK8 | AC092953.2 | 0.321977858 | 4.40E-05 |
| RAB1A | AC092953.2 | 0.300159925 | 0.000147888 |
| RAB33B | AC092953.2 | 0.354999445 | 5.83E-06 |
| RHEB | AC092953.2 | 0.313735636 | 7.03E-05 |
| ATG9A | AC027031.2 | -0.332906497 | 2.31E-05 |
| GAPDH | AC027031.2 | 0.360790962 | 3.99E-06 |
| PIK3C3 | AC027031.2 | 0.337526153 | 1.75E-05 |
| BAX | AC091182.2 | -0.317536571 | 5.67E-05 |
| CDKN1B | AC091182.2 | -0.352795407 | 6.72E-06 |
| HDAC6 | AC091182.2 | -0.320797649 | 4.71E-05 |
| IL24 | AC091182.2 | 0.424799294 | 3.61E-08 |
| ITGA3 | AC091182.2 | 0.398203587 | 2.88E-07 |
| ITGA6 | AC091182.2 | 0.335239263 | 2.01E-05 |
| ITGB4 | AC091182.2 | 0.304345613 | 0.000118049 |
| PINK1 | AC091182.2 | 0.349443391 | 8.33E-06 |
| PRKAB1 | AC091182.2 | -0.305552283 | 0.000110553 |
| ATG4C | TTTY15 | 0.323668283 | 3.99E-05 |
| BCL2 | TTTY15 | 0.358718685 | 4.58E-06 |
| CDKN1B | TTTY15 | 0.361296286 | 3.86E-06 |
| HDAC6 | TTTY15 | 0.399262945 | 2.66E-07 |
| IKBKB | TTTY15 | 0.365615486 | 2.90E-06 |
| MAPK8IP1 | TTTY15 | 0.309965364 | 8.68E-05 |
| MAPK9 | TTTY15 | 0.318748163 | 5.29E-05 |
| PRKAR1A | TTTY15 | 0.380177983 | 1.06E-06 |
| ULK2 | TTTY15 | 0.375298904 | 1.50E-06 |
| ATG10 | OSER1-DT | 0.346684443 | 9.91E-06 |
| ATG4A | OSER1-DT | 0.341305881 | 1.39E-05 |
| ATG4C | OSER1-DT | 0.427763555 | 2.83E-08 |
| ATIC | OSER1-DT | 0.339146351 | 1.58E-05 |
| BAG3 | OSER1-DT | -0.327683362 | 3.15E-05 |
| BAX | OSER1-DT | 0.489369883 | 1.04E-10 |
| BCL2 | OSER1-DT | 0.434855112 | 1.57E-08 |
| BIRC5 | OSER1-DT | 0.502183164 | 2.79E-11 |
| BNIP1 | OSER1-DT | 0.475063356 | 4.22E-10 |
| CAPN10 | OSER1-DT | 0.328016758 | 3.09E-05 |
| CASP3 | OSER1-DT | 0.309590364 | 8.86E-05 |
| CDKN1B | OSER1-DT | 0.327248376 | 3.24E-05 |
| CLN3 | OSER1-DT | 0.337497177 | 1.75E-05 |
| CXCR4 | OSER1-DT | 0.406285755 | 1.56E-07 |
| EIF4G1 | OSER1-DT | -0.311140679 | 8.13E-05 |
| FAS | OSER1-DT | 0.374898911 | 1.54E-06 |
| FKBP1A | OSER1-DT | 0.360151837 | 4.16E-06 |
| GABARAP | OSER1-DT | 0.38109142 | 9.98E-07 |
| HDAC6 | OSER1-DT | 0.603862321 | 8.99E-17 |
| HIF1A | OSER1-DT | -0.323893681 | 3.94E-05 |
| ITGA6 | OSER1-DT | -0.33864554 | 1.63E-05 |
| ITGB4 | OSER1-DT | -0.316027644 | 6.18E-05 |
| MAPK8IP1 | OSER1-DT | 0.403841642 | 1.88E-07 |
| MAPK9 | OSER1-DT | 0.308398876 | 9.46E-05 |
| MTMR14 | OSER1-DT | 0.414264186 | 8.39E-08 |
| NKX2-3 | OSER1-DT | 0.308680232 | 9.31E-05 |
| PARP1 | OSER1-DT | 0.336559 | 1.85E-05 |
| PRKAB1 | OSER1-DT | 0.307273237 | 0.00010063 |
| PTK6 | OSER1-DT | -0.349236146 | 8.44E-06 |
| TP53 | OSER1-DT | 0.315109948 | 6.51E-05 |
| TP73 | OSER1-DT | 0.30419975 | 0.000118986 |
| TSC2 | OSER1-DT | 0.349863174 | 8.11E-06 |
| WDR45 | OSER1-DT | 0.548945018 | 1.41E-13 |
| DAPK2 | AC104695.2 | 0.312738067 | 7.44E-05 |
| PELP1 | AC104695.2 | 0.325330702 | 3.62E-05 |
| ATG3 | AL451062.1 | 0.372556565 | 1.81E-06 |
| FOXO3 | AL451062.1 | 0.405664026 | 1.64E-07 |
| ATF6 | AP002336.2 | 0.454777713 | 2.77E-09 |
| DNAJB1 | AP002336.2 | 0.329757153 | 2.79E-05 |
| DNAJB9 | AP002336.2 | 0.375379506 | 1.49E-06 |
| FADD | AP002336.2 | 0.56671075 | 1.51E-14 |
| PEA15 | AP002336.2 | 0.321056546 | 4.64E-05 |
| PIK3C3 | AP002336.2 | 0.395469287 | 3.53E-07 |
| SQSTM1 | AP002336.2 | 0.379569528 | 1.11E-06 |
| ATIC | AC009309.1 | 0.354511223 | 6.02E-06 |
| HDAC6 | AC009309.1 | 0.349059523 | 8.53E-06 |
| MAPK8IP1 | AC009309.1 | 0.303137642 | 0.000126026 |
| WIPI1 | AC009309.1 | 0.307055234 | 0.000101839 |
| AMBRA1 | PAXIP1-AS2 | 0.406407366 | 1.55E-07 |
| ARNT | PAXIP1-AS2 | 0.462080979 | 1.43E-09 |
| ARSB | PAXIP1-AS2 | 0.428022592 | 2.77E-08 |
| ATG2B | PAXIP1-AS2 | 0.42559727 | 3.38E-08 |
| ATG4C | PAXIP1-AS2 | 0.361155933 | 3.90E-06 |
| BCL2 | PAXIP1-AS2 | 0.399764577 | 2.56E-07 |
| BECN1 | PAXIP1-AS2 | 0.326314591 | 3.42E-05 |
| BIRC6 | PAXIP1-AS2 | 0.49357918 | 6.77E-11 |
| CAMKK2 | PAXIP1-AS2 | 0.310207363 | 8.56E-05 |
| CANX | PAXIP1-AS2 | 0.336314072 | 1.88E-05 |
| CASP8 | PAXIP1-AS2 | 0.321492572 | 4.52E-05 |
| CCR2 | PAXIP1-AS2 | 0.359710993 | 4.29E-06 |
| DAPK1 | PAXIP1-AS2 | 0.36100175 | 3.94E-06 |
| DRAM1 | PAXIP1-AS2 | 0.321455174 | 4.53E-05 |
| EEF2K | PAXIP1-AS2 | 0.460270899 | 1.69E-09 |
| EIF2AK2 | PAXIP1-AS2 | 0.30038014 | 0.000146157 |
| EIF2AK3 | PAXIP1-AS2 | 0.443743046 | 7.34E-09 |
| ERN1 | PAXIP1-AS2 | 0.399386365 | 2.63E-07 |
| FAS | PAXIP1-AS2 | 0.351617073 | 7.25E-06 |
| FOXO3 | PAXIP1-AS2 | 0.323994895 | 3.91E-05 |
| GOPC | PAXIP1-AS2 | 0.333041391 | 2.29E-05 |
| HDAC6 | PAXIP1-AS2 | 0.478695097 | 2.97E-10 |
| ITPR1 | PAXIP1-AS2 | 0.422299808 | 4.42E-08 |
| KLHL24 | PAXIP1-AS2 | 0.436676734 | 1.34E-08 |
| LAMP2 | PAXIP1-AS2 | 0.362014241 | 3.68E-06 |
| MAPK1 | PAXIP1-AS2 | 0.34632157 | 1.01E-05 |
| MAPK8 | PAXIP1-AS2 | 0.321933561 | 4.41E-05 |
| MAPK9 | PAXIP1-AS2 | 0.31183151 | 7.82E-05 |
| MBTPS2 | PAXIP1-AS2 | 0.326886612 | 3.30E-05 |
| MTOR | PAXIP1-AS2 | 0.468067636 | 8.19E-10 |
| NAF1 | PAXIP1-AS2 | 0.334190965 | 2.14E-05 |
| NBR1 | PAXIP1-AS2 | 0.500586773 | 3.29E-11 |
| PARP1 | PAXIP1-AS2 | 0.432746334 | 1.87E-08 |
| PIK3R4 | PAXIP1-AS2 | 0.451035234 | 3.87E-09 |
| PRKAR1A | PAXIP1-AS2 | 0.31393534 | 6.95E-05 |
| RAB33B | PAXIP1-AS2 | 0.423157416 | 4.12E-08 |
| RB1CC1 | PAXIP1-AS2 | 0.350920239 | 7.58E-06 |
| RPS6KB1 | PAXIP1-AS2 | 0.33417488 | 2.14E-05 |
| RPTOR | PAXIP1-AS2 | 0.317830111 | 5.58E-05 |
| SIRT1 | PAXIP1-AS2 | 0.427416317 | 2.91E-08 |
| TM9SF1 | PAXIP1-AS2 | 0.362769487 | 3.50E-06 |
| TP53 | PAXIP1-AS2 | 0.303274687 | 0.000125096 |
| TP73 | PAXIP1-AS2 | 0.423413506 | 4.04E-08 |
| TSC1 | PAXIP1-AS2 | 0.39290292 | 4.26E-07 |
| TSC2 | PAXIP1-AS2 | 0.39154529 | 4.71E-07 |
| UVRAG | PAXIP1-AS2 | 0.389127693 | 5.61E-07 |
| VAMP7 | PAXIP1-AS2 | 0.353789926 | 6.30E-06 |
| WDFY3 | PAXIP1-AS2 | 0.353180082 | 6.56E-06 |
| ATF4 | SNHG17 | 0.403410098 | 1.94E-07 |
| ATG2B | SNHG17 | -0.320768555 | 4.72E-05 |
| ATG4B | SNHG17 | 0.521853645 | 3.33E-12 |
| BNIP1 | SNHG17 | 0.433004273 | 1.83E-08 |
| CAPN10 | SNHG17 | 0.350958789 | 7.56E-06 |
| MAP1LC3A | SNHG17 | 0.34061077 | 1.45E-05 |
| STK11 | SNHG17 | 0.30926194 | 9.02E-05 |
| WDR45 | SNHG17 | 0.456130094 | 2.45E-09 |
| ATF4 | AC083809.1 | 0.330673392 | 2.64E-05 |
| CASP1 | AC083809.1 | 0.336966596 | 1.81E-05 |
| ATG10 | AC011374.2 | 0.361589436 | 3.79E-06 |
| ATG12 | AC011374.2 | 0.323898543 | 3.94E-05 |
| ATG16L2 | AC011374.2 | 0.364366645 | 3.15E-06 |
| ATG3 | AC011374.2 | 0.314870459 | 6.60E-05 |
| ATG4B | AC011374.2 | 0.44460392 | 6.81E-09 |
| BAX | AC011374.2 | 0.453303955 | 3.16E-09 |
| BIRC6 | AC011374.2 | -0.370444881 | 2.09E-06 |
| BNIP1 | AC011374.2 | 0.457146468 | 2.24E-09 |
| CAPN10 | AC011374.2 | 0.447722283 | 5.19E-09 |
| CAPNS1 | AC011374.2 | 0.351336433 | 7.38E-06 |
| CDKN2A | AC011374.2 | 0.306455292 | 0.000105238 |
| CLN3 | AC011374.2 | 0.304727849 | 0.000115625 |
| CXCR4 | AC011374.2 | 0.361735001 | 3.75E-06 |
| DAPK2 | AC011374.2 | 0.339756771 | 1.52E-05 |
| EIF4G1 | AC011374.2 | -0.358175903 | 4.74E-06 |
| FKBP1A | AC011374.2 | 0.429336699 | 2.48E-08 |
| GABARAP | AC011374.2 | 0.39017672 | 5.20E-07 |
| KIF5B | AC011374.2 | -0.319736284 | 5.00E-05 |
| MAPK1 | AC011374.2 | -0.362110835 | 3.66E-06 |
| MTMR14 | AC011374.2 | 0.384875381 | 7.63E-07 |
| NCKAP1 | AC011374.2 | -0.438986169 | 1.10E-08 |
| PEX14 | AC011374.2 | 0.486042437 | 1.45E-10 |
| RAB24 | AC011374.2 | 0.424414286 | 3.72E-08 |
| RGS19 | AC011374.2 | 0.358070664 | 4.77E-06 |
| STK11 | AC011374.2 | 0.41171592 | 1.02E-07 |
| TP63 | AC011374.2 | -0.38176688 | 9.51E-07 |
| USP10 | AC011374.2 | -0.330902405 | 2.61E-05 |
| WDFY3 | AC011374.2 | -0.364726336 | 3.07E-06 |
| WDR45 | AC011374.2 | 0.369079959 | 2.29E-06 |
| ARNT | DHRS4-AS1 | 0.361746172 | 3.75E-06 |
| ATG2B | DHRS4-AS1 | 0.356567615 | 5.27E-06 |
| BAK1 | DHRS4-AS1 | -0.311177833 | 8.11E-05 |
| BECN1 | DHRS4-AS1 | 0.329395506 | 2.85E-05 |
| BIRC6 | DHRS4-AS1 | 0.447422679 | 5.32E-09 |
| CAMKK2 | DHRS4-AS1 | 0.356537565 | 5.28E-06 |
| CASP4 | DHRS4-AS1 | -0.318937986 | 5.24E-05 |
| CDKN1B | DHRS4-AS1 | 0.362959925 | 3.46E-06 |
| EEF2 | DHRS4-AS1 | 0.360811362 | 3.99E-06 |
| EEF2K | DHRS4-AS1 | 0.319309348 | 5.13E-05 |
| EIF2AK3 | DHRS4-AS1 | 0.446894921 | 5.58E-09 |
| ERN1 | DHRS4-AS1 | 0.33277532 | 2.33E-05 |
| HDAC6 | DHRS4-AS1 | 0.301648291 | 0.000136553 |
| ITGA3 | DHRS4-AS1 | -0.312208018 | 7.66E-05 |
| KLHL24 | DHRS4-AS1 | 0.395532825 | 3.51E-07 |
| MAPK1 | DHRS4-AS1 | 0.332795757 | 2.33E-05 |
| MBTPS2 | DHRS4-AS1 | 0.391286078 | 4.80E-07 |
| NBR1 | DHRS4-AS1 | 0.366746895 | 2.68E-06 |
| NCKAP1 | DHRS4-AS1 | 0.338250221 | 1.67E-05 |
| NFE2L2 | DHRS4-AS1 | 0.423091494 | 4.14E-08 |
| PARP1 | DHRS4-AS1 | 0.354108275 | 6.18E-06 |
| PIK3R4 | DHRS4-AS1 | 0.32299547 | 4.15E-05 |
| PRKAB1 | DHRS4-AS1 | 0.371578313 | 1.93E-06 |
| RB1CC1 | DHRS4-AS1 | 0.385694814 | 7.19E-07 |
| RHEB | DHRS4-AS1 | 0.332002716 | 2.44E-05 |
| SIRT1 | DHRS4-AS1 | 0.381799554 | 9.49E-07 |
| TM9SF1 | DHRS4-AS1 | 0.418881866 | 5.82E-08 |
| TP63 | DHRS4-AS1 | 0.302689528 | 0.00012911 |
| UVRAG | DHRS4-AS1 | 0.38115583 | 9.93E-07 |
| ATF4 | AC084036.1 | 0.361939898 | 3.70E-06 |
| ATG4B | AC084036.1 | 0.486851876 | 1.33E-10 |
| ATG4D | AC084036.1 | 0.303476826 | 0.000123736 |
| BAX | AC084036.1 | 0.380165748 | 1.07E-06 |
| BID | AC084036.1 | 0.307960784 | 9.69E-05 |
| CAPN10 | AC084036.1 | 0.48014633 | 2.58E-10 |
| DAPK2 | AC084036.1 | 0.524702851 | 2.42E-12 |
| GABARAP | AC084036.1 | 0.355847008 | 5.52E-06 |
| NCKAP1 | AC084036.1 | -0.314353041 | 6.79E-05 |
| PELP1 | AC084036.1 | 0.311255606 | 8.08E-05 |
| PEX14 | AC084036.1 | 0.325796848 | 3.52E-05 |
| RAB24 | AC084036.1 | 0.342140249 | 1.32E-05 |
| STK11 | AC084036.1 | 0.401265518 | 2.29E-07 |
| USP10 | AC084036.1 | -0.315970778 | 6.20E-05 |
| WDR45 | AC084036.1 | 0.338093931 | 1.69E-05 |
| ZFYVE1 | AC084036.1 | -0.331103498 | 2.57E-05 |
| BID | IER3-AS1 | 0.452958355 | 3.26E-09 |
| GABARAPL2 | IER3-AS1 | 0.319462994 | 5.08E-05 |
| PPP1R15A | IER3-AS1 | 0.391711862 | 4.65E-07 |
| ATF4 | LINC01578 | 0.442481088 | 8.18E-09 |
| ATG10 | LINC01578 | 0.329129412 | 2.90E-05 |
| ATG16L2 | LINC01578 | 0.322351698 | 4.30E-05 |
| ATG2B | LINC01578 | -0.303796195 | 0.000121617 |
| ATG4B | LINC01578 | 0.516042546 | 6.32E-12 |
| ATG4D | LINC01578 | 0.477369155 | 3.38E-10 |
| BAG1 | LINC01578 | 0.360585759 | 4.05E-06 |
| BAX | LINC01578 | 0.444587314 | 6.82E-09 |
| BNIP1 | LINC01578 | 0.535846523 | 6.74E-13 |
| CAPN10 | LINC01578 | 0.496978426 | 4.78E-11 |
| CAPNS1 | LINC01578 | 0.339749892 | 1.53E-05 |
| CASP3 | LINC01578 | 0.379508524 | 1.12E-06 |
| CDKN1B | LINC01578 | 0.337121319 | 1.79E-05 |
| CDKN2A | LINC01578 | 0.460748242 | 1.61E-09 |
| CFLAR | LINC01578 | 0.342379383 | 1.30E-05 |
| CLN3 | LINC01578 | 0.395008886 | 3.65E-07 |
| CXCR4 | LINC01578 | 0.368620224 | 2.37E-06 |
| DDIT3 | LINC01578 | 0.308560585 | 9.38E-05 |
| DLC1 | LINC01578 | -0.302865729 | 0.000127889 |
| EIF4G1 | LINC01578 | -0.348972678 | 8.58E-06 |
| GABARAP | LINC01578 | 0.34110046 | 1.40E-05 |
| HDAC6 | LINC01578 | 0.405718936 | 1.63E-07 |
| HIF1A | LINC01578 | -0.316587201 | 5.99E-05 |
| HSP90AB1 | LINC01578 | -0.347930807 | 9.16E-06 |
| HSPA8 | LINC01578 | -0.345717897 | 1.05E-05 |
| IKBKE | LINC01578 | 0.351401295 | 7.35E-06 |
| ITGA3 | LINC01578 | -0.392588238 | 4.36E-07 |
| ITGA6 | LINC01578 | -0.363133854 | 3.42E-06 |
| ITGB1 | LINC01578 | -0.347347114 | 9.51E-06 |
| ITGB4 | LINC01578 | -0.336935124 | 1.81E-05 |
| MAP1LC3A | LINC01578 | 0.32895249 | 2.93E-05 |
| MAP2K7 | LINC01578 | 0.37914533 | 1.14E-06 |
| MAPK1 | LINC01578 | -0.309839221 | 8.74E-05 |
| MTMR14 | LINC01578 | 0.365044495 | 3.01E-06 |
| NKX2-3 | LINC01578 | 0.378312898 | 1.21E-06 |
| NRG1 | LINC01578 | -0.331025578 | 2.59E-05 |
| PEX14 | LINC01578 | 0.34002411 | 1.50E-05 |
| PINK1 | LINC01578 | -0.402250481 | 2.12E-07 |
| PRKAB1 | LINC01578 | 0.402436579 | 2.09E-07 |
| RAB24 | LINC01578 | 0.489646876 | 1.01E-10 |
| RPTOR | LINC01578 | -0.351910578 | 7.11E-06 |
| STK11 | LINC01578 | 0.403531637 | 1.93E-07 |
| TSC2 | LINC01578 | 0.333174616 | 2.27E-05 |
| ULK3 | LINC01578 | 0.379757409 | 1.10E-06 |
| WDFY3 | LINC01578 | -0.314123512 | 6.88E-05 |
| WDR45 | LINC01578 | 0.584961813 | 1.32E-15 |
| ATG10 | AC096677.1 | 0.388042729 | 6.07E-07 |
| ATG4B | AC096677.1 | 0.335884685 | 1.93E-05 |
| ATG4C | AC096677.1 | 0.320243198 | 4.86E-05 |
| CAPN10 | AC096677.1 | 0.412387186 | 9.72E-08 |
| CD46 | AC096677.1 | 0.356477337 | 5.30E-06 |
| CDKN1B | AC096677.1 | 0.349073462 | 8.52E-06 |
| CX3CL1 | AC096677.1 | 0.324308712 | 3.84E-05 |
| CXCR4 | AC096677.1 | 0.444537365 | 6.85E-09 |
| EEF2K | AC096677.1 | 0.321531935 | 4.51E-05 |
| FAS | AC096677.1 | 0.312209631 | 7.66E-05 |
| HDAC6 | AC096677.1 | 0.398955808 | 2.72E-07 |
| IKBKB | AC096677.1 | 0.319609798 | 5.04E-05 |
| IKBKE | AC096677.1 | 0.375662116 | 1.46E-06 |
| ITGA3 | AC096677.1 | -0.318711636 | 5.31E-05 |
| KLHL24 | AC096677.1 | 0.321881233 | 4.42E-05 |
| MAP2K7 | AC096677.1 | 0.333955742 | 2.17E-05 |
| MAPK8 | AC096677.1 | 0.351687135 | 7.22E-06 |
| PARP1 | AC096677.1 | 0.447132247 | 5.46E-09 |
| PINK1 | AC096677.1 | -0.388705366 | 5.79E-07 |
| PRKAB1 | AC096677.1 | 0.373613938 | 1.68E-06 |
| RAF1 | AC096677.1 | 0.331219016 | 2.56E-05 |
| SIRT1 | AC096677.1 | 0.326581147 | 3.36E-05 |
| TP53 | AC096677.1 | 0.424510334 | 3.69E-08 |
| TP73 | AC096677.1 | 0.372301226 | 1.84E-06 |
| TSC2 | AC096677.1 | 0.516026596 | 6.33E-12 |
| ULK3 | AC096677.1 | 0.400223033 | 2.47E-07 |
| UVRAG | AC096677.1 | 0.396640962 | 3.23E-07 |
| WDR45 | AC096677.1 | 0.326759737 | 3.33E-05 |
| ATG9B | AL139385.1 | 0.472055039 | 5.62E-10 |
| BAX | AL139385.1 | -0.337605999 | 1.74E-05 |
| DRAM1 | AL139385.1 | -0.333719948 | 2.20E-05 |
| EIF4G1 | AL139385.1 | 0.337749897 | 1.72E-05 |
| FAS | AL139385.1 | -0.312434253 | 7.56E-05 |
| GABARAP | AL139385.1 | -0.335635137 | 1.96E-05 |
| NCKAP1 | AL139385.1 | 0.322127077 | 4.36E-05 |
| PEX14 | AL139385.1 | -0.300468968 | 0.000145464 |
| PTK6 | AL139385.1 | 0.473487836 | 4.90E-10 |
| RAB11A | AL139385.1 | 0.445138464 | 6.50E-09 |
| RAB1A | AL139385.1 | 0.455639741 | 2.56E-09 |
| TP53INP2 | AL139385.1 | 0.406473889 | 1.54E-07 |
| WDFY3 | AL139385.1 | 0.322943718 | 4.16E-05 |
| BECN1 | SNHG16 | 0.349363991 | 8.37E-06 |
| BIRC6 | SNHG16 | 0.379609908 | 1.11E-06 |
| CAMKK2 | SNHG16 | 0.311917119 | 7.78E-05 |
| CTSD | SNHG16 | -0.316266804 | 6.10E-05 |
| EEF2 | SNHG16 | 0.300198588 | 0.000147582 |
| GABARAPL1 | SNHG16 | 0.336882925 | 1.82E-05 |
| HGS | SNHG16 | 0.381609785 | 9.62E-07 |
| HSP90AB1 | SNHG16 | 0.359836397 | 4.25E-06 |
| NBR1 | SNHG16 | 0.356248028 | 5.38E-06 |
| NCKAP1 | SNHG16 | 0.37421412 | 1.61E-06 |
| NFE2L2 | SNHG16 | 0.318613694 | 5.34E-05 |
| RAB1A | SNHG16 | 0.309894971 | 8.71E-05 |
| RPS6KB1 | SNHG16 | 0.385010726 | 7.55E-07 |
| RPTOR | SNHG16 | 0.376398803 | 1.39E-06 |
| SQSTM1 | SNHG16 | 0.308819597 | 9.24E-05 |
| ST13 | SNHG16 | 0.302222878 | 0.000132398 |
| WIPI1 | SNHG16 | 0.357954135 | 4.81E-06 |
| ATG12 | AC011450.1 | 0.34680664 | 9.84E-06 |
| ATG4C | AC011450.1 | 0.393280725 | 4.15E-07 |
| BAG3 | AC011450.1 | -0.307632128 | 9.87E-05 |
| BCL2 | AC011450.1 | 0.454808665 | 2.76E-09 |
| CAPN1 | AC011450.1 | -0.305434547 | 0.000111265 |
| CAPN10 | AC011450.1 | 0.305835117 | 0.000108862 |
| CAPN2 | AC011450.1 | -0.308771327 | 9.27E-05 |
| CDKN1B | AC011450.1 | 0.389190952 | 5.59E-07 |
| CFLAR | AC011450.1 | 0.32632407 | 3.42E-05 |
| CXCR4 | AC011450.1 | 0.406779148 | 1.50E-07 |
| EEF2K | AC011450.1 | 0.362163154 | 3.65E-06 |
| FAS | AC011450.1 | 0.329533007 | 2.83E-05 |
| HDAC6 | AC011450.1 | 0.552900777 | 8.68E-14 |
| IKBKB | AC011450.1 | 0.3145679 | 6.71E-05 |
| KLHL24 | AC011450.1 | 0.374737754 | 1.56E-06 |
| MAP2K7 | AC011450.1 | 0.471619526 | 5.86E-10 |
| MAPK8 | AC011450.1 | 0.42005058 | 5.30E-08 |
| MAPK9 | AC011450.1 | 0.352689892 | 6.77E-06 |
| NKX2-3 | AC011450.1 | 0.450282356 | 4.14E-09 |
| PARP1 | AC011450.1 | 0.362232279 | 3.63E-06 |
| PIK3R4 | AC011450.1 | 0.337047236 | 1.80E-05 |
| PRKAB1 | AC011450.1 | 0.408831079 | 1.28E-07 |
| PTK6 | AC011450.1 | -0.350747297 | 7.66E-06 |
| RAF1 | AC011450.1 | 0.337872883 | 1.71E-05 |
| TP53 | AC011450.1 | 0.397723434 | 2.98E-07 |
| TP73 | AC011450.1 | 0.439476413 | 1.06E-08 |
| TSC1 | AC011450.1 | 0.434235504 | 1.65E-08 |
| TSC2 | AC011450.1 | 0.479905796 | 2.64E-10 |
| ULK2 | AC011450.1 | 0.302124254 | 0.000133102 |
| WDR45 | AC011450.1 | 0.386506258 | 6.78E-07 |
| ATG16L2 | AC008105.3 | 0.402253884 | 2.12E-07 |
| ATG4B | AC008105.3 | 0.334778468 | 2.07E-05 |
| BAX | AC008105.3 | 0.45712417 | 2.24E-09 |
| CAPN10 | AC008105.3 | 0.508750054 | 1.39E-11 |
| CCR2 | AC008105.3 | 0.414906227 | 7.98E-08 |
| CFLAR | AC008105.3 | 0.306250086 | 0.000106424 |
| CXCR4 | AC008105.3 | 0.614646449 | 1.79E-17 |
| DAPK2 | AC008105.3 | 0.341224659 | 1.39E-05 |
| IFNG | AC008105.3 | 0.430527579 | 2.25E-08 |
| IKBKB | AC008105.3 | 0.340561693 | 1.45E-05 |
| MAP2K7 | AC008105.3 | 0.376836079 | 1.35E-06 |
| MTMR14 | AC008105.3 | 0.421340077 | 4.77E-08 |
| NCKAP1 | AC008105.3 | -0.3231726 | 4.10E-05 |
| RAB24 | AC008105.3 | 0.51975491 | 4.20E-12 |
| RAF1 | AC008105.3 | 0.314867144 | 6.60E-05 |
| RGS19 | AC008105.3 | 0.455992618 | 2.48E-09 |
| STK11 | AC008105.3 | 0.420580682 | 5.08E-08 |
| ULK3 | AC008105.3 | 0.372225723 | 1.85E-06 |
| WDFY3 | AC008105.3 | -0.300673097 | 0.000143884 |
| WDR45 | AC008105.3 | 0.33973211 | 1.53E-05 |
| ATG16L2 | AC108488.1 | 0.494937567 | 5.90E-11 |
| ATG4B | AC108488.1 | 0.429529542 | 2.45E-08 |
| BID | AC108488.1 | 0.504231844 | 2.25E-11 |
| CANX | AC108488.1 | -0.300390902 | 0.000146073 |
| CAPN10 | AC108488.1 | 0.44133528 | 9.03E-09 |
| DAPK2 | AC108488.1 | 0.302822933 | 0.000128185 |
| KIF5B | AC108488.1 | -0.331661725 | 2.49E-05 |
| MLST8 | AC108488.1 | 0.301604237 | 0.000136876 |
| ATG12 | EIF3J-DT | 0.30561315 | 0.000110187 |
| ATG4A | EIF3J-DT | 0.304623704 | 0.00011628 |
| ATG4C | EIF3J-DT | 0.416884065 | 6.82E-08 |
| ATG9B | EIF3J-DT | 0.314558152 | 6.71E-05 |
| ATIC | EIF3J-DT | 0.472958883 | 5.16E-10 |
| BCL2 | EIF3J-DT | 0.308084491 | 9.62E-05 |
| CAPN10 | EIF3J-DT | 0.384645606 | 7.75E-07 |
| CD46 | EIF3J-DT | 0.363772633 | 3.28E-06 |
| CDKN1B | EIF3J-DT | 0.387868153 | 6.15E-07 |
| DIRAS3 | EIF3J-DT | 0.439692664 | 1.04E-08 |
| GABARAPL2 | EIF3J-DT | 0.364199971 | 3.18E-06 |
| HDAC6 | EIF3J-DT | 0.491382236 | 8.46E-11 |
| IKBKB | EIF3J-DT | 0.317962762 | 5.54E-05 |
| ITGA3 | EIF3J-DT | -0.314506522 | 6.73E-05 |
| MAP1LC3A | EIF3J-DT | 0.308517376 | 9.40E-05 |
| MAPK8 | EIF3J-DT | 0.354292522 | 6.10E-06 |
| MAPK8IP1 | EIF3J-DT | 0.503285139 | 2.48E-11 |
| MTMR14 | EIF3J-DT | 0.332441711 | 2.38E-05 |
| PRKAR1A | EIF3J-DT | 0.428415978 | 2.68E-08 |
| RAB33B | EIF3J-DT | 0.335006856 | 2.04E-05 |
| RAF1 | EIF3J-DT | 0.392663716 | 4.34E-07 |
| SIRT1 | EIF3J-DT | 0.368420848 | 2.40E-06 |
| ULK3 | EIF3J-DT | 0.476887409 | 3.54E-10 |
| VAMP7 | EIF3J-DT | 0.351144514 | 7.47E-06 |
| ATG4C | PRKAG2-AS1 | 0.340528923 | 1.45E-05 |
| ATIC | PRKAG2-AS1 | 0.531043807 | 1.18E-12 |
| BIRC5 | PRKAG2-AS1 | 0.452734714 | 3.33E-09 |
| HDAC6 | PRKAG2-AS1 | 0.437886211 | 1.21E-08 |
| MAPK8IP1 | PRKAG2-AS1 | 0.787064397 | 6.47E-34 |
| PRKAR1A | PRKAG2-AS1 | 0.566465775 | 1.56E-14 |
| VAMP7 | PRKAG2-AS1 | 0.347718527 | 9.29E-06 |
| CHMP4B | HOTAIRM1 | 0.352327241 | 6.93E-06 |
| ATF4 | AL359921.2 | 0.415498174 | 7.61E-08 |
| ATG16L1 | AL359921.2 | 0.315045929 | 6.53E-05 |
| ATG16L2 | AL359921.2 | 0.349389549 | 8.35E-06 |
| ATG4B | AL359921.2 | 0.472876358 | 5.20E-10 |
| BAX | AL359921.2 | 0.383997598 | 8.12E-07 |
| CAPN10 | AL359921.2 | 0.517935535 | 5.14E-12 |
| CXCR4 | AL359921.2 | 0.313457839 | 7.14E-05 |
| DAPK2 | AL359921.2 | 0.498267361 | 4.19E-11 |
| MAP2K7 | AL359921.2 | 0.354314056 | 6.09E-06 |
| PELP1 | AL359921.2 | 0.340843124 | 1.43E-05 |
| PEX14 | AL359921.2 | 0.343748031 | 1.19E-05 |
| RAB24 | AL359921.2 | 0.305356114 | 0.000111741 |
| STK11 | AL359921.2 | 0.349081819 | 8.52E-06 |
| WDR45 | AL359921.2 | 0.388574308 | 5.84E-07 |
| AMBRA1 | ZSCAN16-AS1 | -0.307677257 | 9.84E-05 |
| ARNT | ZSCAN16-AS1 | -0.305589155 | 0.000110331 |
| ATF4 | ZSCAN16-AS1 | 0.446745708 | 5.65E-09 |
| ATG2A | ZSCAN16-AS1 | -0.327690817 | 3.15E-05 |
| ATG4B | ZSCAN16-AS1 | 0.316851807 | 5.90E-05 |
| ATIC | ZSCAN16-AS1 | 0.313291295 | 7.21E-05 |
| BAG3 | ZSCAN16-AS1 | -0.346443203 | 1.01E-05 |
| BAX | ZSCAN16-AS1 | 0.492565575 | 7.51E-11 |
| BIRC5 | ZSCAN16-AS1 | 0.395939062 | 3.41E-07 |
| BIRC6 | ZSCAN16-AS1 | -0.373395818 | 1.71E-06 |
| BNIP1 | ZSCAN16-AS1 | 0.572570314 | 7.04E-15 |
| CAMKK2 | ZSCAN16-AS1 | -0.319685797 | 5.02E-05 |
| CAPN1 | ZSCAN16-AS1 | -0.333577676 | 2.22E-05 |
| CAPN2 | ZSCAN16-AS1 | -0.370890932 | 2.03E-06 |
| DDIT3 | ZSCAN16-AS1 | 0.545247937 | 2.21E-13 |
| GOPC | ZSCAN16-AS1 | -0.368867186 | 2.33E-06 |
| HGS | ZSCAN16-AS1 | -0.378610679 | 1.19E-06 |
| KIF5B | ZSCAN16-AS1 | -0.337390629 | 1.76E-05 |
| LAMP2 | ZSCAN16-AS1 | -0.33925713 | 1.57E-05 |
| MAPK1 | ZSCAN16-AS1 | -0.420200878 | 5.23E-08 |
| NBR1 | ZSCAN16-AS1 | -0.302964577 | 0.000127209 |
| NCKAP1 | ZSCAN16-AS1 | -0.405770644 | 1.62E-07 |
| NFKB1 | ZSCAN16-AS1 | -0.355556762 | 5.62E-06 |
| NPC1 | ZSCAN16-AS1 | -0.333987506 | 2.17E-05 |
| RB1 | ZSCAN16-AS1 | -0.339653526 | 1.53E-05 |
| SPNS1 | ZSCAN16-AS1 | -0.33220653 | 2.41E-05 |
| USP10 | ZSCAN16-AS1 | -0.313960039 | 6.94E-05 |
| WDFY3 | ZSCAN16-AS1 | -0.331236217 | 2.55E-05 |
| WDR45 | ZSCAN16-AS1 | 0.32392966 | 3.93E-05 |
| PINK1 | AC009902.2 | 0.322389802 | 4.30E-05 |
| ATG4B | AL355472.1 | 0.312325328 | 7.61E-05 |
| BID | AL355472.1 | 0.412393184 | 9.72E-08 |
| CAPN10 | AL355472.1 | 0.347087641 | 9.66E-06 |
| CLN3 | AL355472.1 | 0.304008177 | 0.000120228 |
| GABARAPL1 | AL355472.1 | 0.346021891 | 1.03E-05 |
| GABARAPL2 | AL355472.1 | 0.401079485 | 2.32E-07 |
| ITGA3 | AL355472.1 | -0.345921079 | 1.04E-05 |
| MAP1LC3A | AL355472.1 | 0.300802905 | 0.000142887 |
| WDR45 | AL355472.1 | 0.350926027 | 7.58E-06 |
| AMBRA1 | AC090589.3 | 0.318439611 | 5.39E-05 |
| ATG12 | AC090589.3 | 0.309846143 | 8.73E-05 |
| CAPN10 | AC090589.3 | 0.351990474 | 7.08E-06 |
| CXCR4 | AC090589.3 | 0.486869196 | 1.33E-10 |
| HDAC6 | AC090589.3 | 0.403404901 | 1.94E-07 |
| IKBKB | AC090589.3 | 0.386445076 | 6.81E-07 |
| TP73 | AC090589.3 | 0.329053904 | 2.91E-05 |
| TSC1 | AC090589.3 | 0.341604105 | 1.36E-05 |
| TSC2 | AC090589.3 | 0.340914471 | 1.42E-05 |
| ULK3 | AC090589.3 | 0.310856425 | 8.26E-05 |
| WDR45 | AC090589.3 | 0.324250933 | 3.86E-05 |
| ATG16L1 | AC027763.2 | 0.301889808 | 0.000134792 |
| ATG4B | AC027763.2 | 0.30790778 | 9.72E-05 |
| BID | AC027763.2 | 0.335798066 | 1.94E-05 |
| DAPK2 | AC027763.2 | 0.301244948 | 0.000139542 |
| PELP1 | AC027763.2 | 0.432216972 | 1.96E-08 |
| ATG3 | AC112484.3 | 0.372857543 | 1.77E-06 |
| ATG4D | AC112484.3 | 0.391168998 | 4.84E-07 |
| MAP2K7 | AC112484.3 | 0.388419652 | 5.91E-07 |
| PELP1 | AC112484.3 | 0.325791232 | 3.52E-05 |
| PIK3C3 | AC112484.3 | 0.45383871 | 3.02E-09 |
| PINK1 | AC112484.3 | -0.339605877 | 1.54E-05 |
| STK11 | AC112484.3 | 0.324519054 | 3.80E-05 |
| ATG10 | AL354892.2 | 0.313192544 | 7.25E-05 |
| ATIC | AL354892.2 | 0.336908826 | 1.81E-05 |
| BAK1 | AL354892.2 | -0.414985266 | 7.93E-08 |
| CAMKK2 | AL354892.2 | 0.301737498 | 0.0001359 |
| CASP4 | AL354892.2 | -0.32220344 | 4.34E-05 |
| CD46 | AL354892.2 | 0.361829552 | 3.73E-06 |
| CDKN1B | AL354892.2 | 0.409370634 | 1.23E-07 |
| DAPK1 | AL354892.2 | 0.321342039 | 4.56E-05 |
| EEF2 | AL354892.2 | 0.422799896 | 4.24E-08 |
| GABARAPL1 | AL354892.2 | 0.308124697 | 9.60E-05 |
| HDAC6 | AL354892.2 | 0.426119492 | 3.24E-08 |
| ITGA3 | AL354892.2 | -0.448014901 | 5.05E-09 |
| KLHL24 | AL354892.2 | 0.428705916 | 2.62E-08 |
| MAPK8IP1 | AL354892.2 | 0.320908665 | 4.68E-05 |
| MBTPS2 | AL354892.2 | 0.306029124 | 0.000107716 |
| PARP1 | AL354892.2 | 0.444876029 | 6.65E-09 |
| PINK1 | AL354892.2 | -0.303090403 | 0.000126347 |
| PRKAB1 | AL354892.2 | 0.335279886 | 2.00E-05 |
| PTK6 | AL354892.2 | -0.305202751 | 0.000112677 |
| RB1CC1 | AL354892.2 | 0.314732928 | 6.65E-05 |
| SIRT1 | AL354892.2 | 0.416426471 | 7.07E-08 |
| ST13 | AL354892.2 | 0.476403293 | 3.71E-10 |
| ULK2 | AL354892.2 | 0.316445787 | 6.04E-05 |
| UVRAG | AL354892.2 | 0.421037475 | 4.89E-08 |
| NFE2L2 | AC079305.1 | 0.388724474 | 5.78E-07 |
| SH3GLB1 | AC079305.1 | -0.312851936 | 7.39E-05 |
| ATF6 | AC055822.1 | -0.310305536 | 8.51E-05 |
| ATG4B | AC055822.1 | 0.352864337 | 6.69E-06 |
| BAG3 | AC055822.1 | -0.359408552 | 4.37E-06 |
| BAX | AC055822.1 | 0.449818952 | 4.31E-09 |
| BECN1 | AC055822.1 | -0.354458707 | 6.04E-06 |
| BIRC5 | AC055822.1 | 0.387530143 | 6.30E-07 |
| BIRC6 | AC055822.1 | -0.436622718 | 1.35E-08 |
| BNIP1 | AC055822.1 | 0.441554414 | 8.86E-09 |
| CAPN10 | AC055822.1 | 0.382689787 | 8.91E-07 |
| EIF2AK3 | AC055822.1 | -0.329762655 | 2.79E-05 |
| EIF4G1 | AC055822.1 | -0.460947079 | 1.58E-09 |
| FKBP1A | AC055822.1 | 0.512097076 | 9.71E-12 |
| FOXO3 | AC055822.1 | -0.318834455 | 5.27E-05 |
| GABARAP | AC055822.1 | 0.306670391 | 0.000104007 |
| KIF5B | AC055822.1 | -0.413753927 | 8.73E-08 |
| MAPK1 | AC055822.1 | -0.398956586 | 2.72E-07 |
| MTMR14 | AC055822.1 | 0.339672039 | 1.53E-05 |
| NCKAP1 | AC055822.1 | -0.411165575 | 1.07E-07 |
| NFE2L2 | AC055822.1 | -0.346131654 | 1.03E-05 |
| NPC1 | AC055822.1 | -0.305874918 | 0.000108626 |
| PEX14 | AC055822.1 | 0.40200363 | 2.16E-07 |
| RAB24 | AC055822.1 | 0.333939948 | 2.17E-05 |
| RPTOR | AC055822.1 | -0.309545171 | 8.88E-05 |
| STK11 | AC055822.1 | 0.307167629 | 0.000101214 |
| TP63 | AC055822.1 | -0.367404666 | 2.57E-06 |
| USP10 | AC055822.1 | -0.300644794 | 0.000144102 |
| WDFY3 | AC055822.1 | -0.401116655 | 2.31E-07 |
| WDR45 | AC055822.1 | 0.356895155 | 5.16E-06 |
| CASP1 | AC008736.1 | 0.387179814 | 6.46E-07 |
| CFLAR | AC008736.1 | 0.365295859 | 2.96E-06 |
| VAMP3 | AC008736.1 | 0.373892675 | 1.65E-06 |
| ATIC | AC010531.6 | 0.378347114 | 1.21E-06 |
| BAX | AC010531.6 | 0.406272904 | 1.56E-07 |
| BIRC5 | AC010531.6 | 0.533145625 | 9.22E-13 |
| FKBP1A | AC010531.6 | 0.460578141 | 1.64E-09 |
| GABARAPL2 | AC010531.6 | 0.400814186 | 2.37E-07 |
| MAPK1 | AC010531.6 | -0.331730633 | 2.48E-05 |
| MAPK8IP1 | AC010531.6 | 0.499829273 | 3.56E-11 |
| MLST8 | AC010531.6 | 0.328900729 | 2.93E-05 |
| WDR45 | AC010531.6 | 0.345998125 | 1.03E-05 |
| GABARAPL1 | LINC01003 | 0.389682522 | 5.39E-07 |
| ITGA3 | LINC01003 | -0.436493481 | 1.37E-08 |
| NFE2L2 | LINC01003 | 0.381234934 | 9.88E-07 |
| RHEB | LINC01003 | 0.461093802 | 1.56E-09 |
| ULK3 | LINC01003 | 0.306529117 | 0.000104814 |
| ARNT | MIR200CHG | -0.362164009 | 3.65E-06 |
| ARSB | MIR200CHG | -0.354008495 | 6.22E-06 |
| ATF4 | MIR200CHG | 0.352315853 | 6.93E-06 |
| ATG4B | MIR200CHG | 0.369239348 | 2.27E-06 |
| BAX | MIR200CHG | 0.316201391 | 6.12E-05 |
| BIRC6 | MIR200CHG | -0.321496134 | 4.52E-05 |
| BNIP1 | MIR200CHG | 0.388779969 | 5.76E-07 |
| CANX | MIR200CHG | -0.367542634 | 2.54E-06 |
| CLN3 | MIR200CHG | 0.311884465 | 7.80E-05 |
| DDIT3 | MIR200CHG | 0.504042577 | 2.29E-11 |
| EDEM1 | MIR200CHG | -0.328732922 | 2.96E-05 |
| EIF4G1 | MIR200CHG | -0.301190795 | 0.000139948 |
| FOXO3 | MIR200CHG | -0.301523183 | 0.000137473 |
| GABARAPL2 | MIR200CHG | 0.31617377 | 6.13E-05 |
| GOPC | MIR200CHG | -0.35393167 | 6.25E-06 |
| LAMP2 | MIR200CHG | -0.320803622 | 4.71E-05 |
| MAPK1 | MIR200CHG | -0.444632521 | 6.79E-09 |
| NAF1 | MIR200CHG | -0.324778099 | 3.74E-05 |
| NFKB1 | MIR200CHG | -0.353301333 | 6.51E-06 |
| PTEN | MIR200CHG | -0.313421818 | 7.16E-05 |
| RAB33B | MIR200CHG | -0.328326067 | 3.04E-05 |
| TM9SF1 | MIR200CHG | -0.306714894 | 0.000103754 |
| USP10 | MIR200CHG | -0.321719904 | 4.46E-05 |
| CLN3 | LINC00941 | -0.318709565 | 5.31E-05 |
| HSPA5 | LINC00941 | 0.31854012 | 5.36E-05 |
| IKBKE | LINC00941 | -0.313509199 | 7.12E-05 |
| ITGA3 | LINC00941 | 0.453124238 | 3.21E-09 |
| ITGA6 | LINC00941 | 0.413557717 | 8.87E-08 |
| ITGB1 | LINC00941 | 0.38921747 | 5.58E-07 |
| ITGB4 | LINC00941 | 0.337513297 | 1.75E-05 |
| MYC | LINC00941 | 0.331396094 | 2.53E-05 |
| NRG1 | LINC00941 | 0.456847971 | 2.30E-09 |
| SAR1A | LINC00941 | 0.311719195 | 7.87E-05 |
| ATG16L2 | AC093297.2 | 0.394619197 | 3.76E-07 |
| ATG4C | AC093297.2 | 0.306183017 | 0.000106815 |
| ATG4D | AC093297.2 | 0.333525404 | 2.23E-05 |
| BID | AC093297.2 | 0.300604094 | 0.000144416 |
| CAPN10 | AC093297.2 | 0.448351905 | 4.91E-09 |
| CD46 | AC093297.2 | 0.303415865 | 0.000124145 |
| DAPK2 | AC093297.2 | 0.37766343 | 1.27E-06 |
| DIRAS3 | AC093297.2 | 0.374902821 | 1.54E-06 |
| MAP2K7 | AC093297.2 | 0.338895816 | 1.61E-05 |
| MAPK8 | AC093297.2 | 0.324790421 | 3.74E-05 |
| PELP1 | AC093297.2 | 0.447322587 | 5.37E-09 |
| ULK3 | AC093297.2 | 0.372405543 | 1.83E-06 |
| ATG4B | AL390198.1 | 0.30816198 | 9.58E-05 |
| DAPK2 | AL390198.1 | 0.315547489 | 6.35E-05 |
| PELP1 | AL390198.1 | 0.530542696 | 1.24E-12 |
| ATIC | AC016065.1 | 0.358045009 | 4.78E-06 |
| BAX | AC016065.1 | 0.300270643 | 0.000147015 |
| CAPN1 | AC016065.1 | -0.302148933 | 0.000132926 |
| CAPN10 | AC016065.1 | 0.347634718 | 9.34E-06 |
| CAPN2 | AC016065.1 | -0.312774509 | 7.42E-05 |
| DIRAS3 | AC016065.1 | 0.484884679 | 1.62E-10 |
| GABARAPL2 | AC016065.1 | 0.310255684 | 8.54E-05 |
| NAMPT | LINC00942 | 0.544923653 | 2.30E-13 |
| TP63 | LINC00942 | 0.328529379 | 3.00E-05 |
| ATG9B | LINC01269 | 0.46163736 | 1.49E-09 |
| CAPN2 | LINC01269 | 0.399252975 | 2.66E-07 |
| CDKN1A | LINC01269 | 0.315742315 | 6.28E-05 |
| CHMP2B | LINC01269 | 0.497076106 | 4.74E-11 |
| EIF2AK3 | LINC01269 | 0.388362262 | 5.93E-07 |
| PTK6 | LINC01269 | 0.322915201 | 4.17E-05 |
| RAB11A | LINC01269 | 0.43982465 | 1.03E-08 |
| RAB5A | LINC01269 | 0.416738793 | 6.90E-08 |
| RAF1 | LINC01269 | 0.338588306 | 1.64E-05 |
| TP53INP2 | LINC01269 | 0.537671364 | 5.44E-13 |
| EEF2K | AP000695.1 | 0.355798468 | 5.54E-06 |
| FAS | AP000695.1 | 0.332519132 | 2.37E-05 |
| ITGA3 | AP000695.1 | 0.300129092 | 0.000148131 |
| MAPK8 | AP000695.1 | 0.390184435 | 5.20E-07 |
| NKX2-3 | AP000695.1 | 0.505403681 | 1.99E-11 |
| TP73 | AP000695.1 | 0.471302991 | 6.04E-10 |
| TSC2 | AP000695.1 | 0.37751212 | 1.28E-06 |
| ATG12 | AP002807.1 | 0.38507116 | 7.52E-07 |
| ATG16L2 | AP002807.1 | 0.398610543 | 2.79E-07 |
| ATG4B | AP002807.1 | 0.388936836 | 5.69E-07 |
| ATG4C | AP002807.1 | 0.331474889 | 2.52E-05 |
| CAPN10 | AP002807.1 | 0.449410062 | 4.47E-09 |
| CXCR4 | AP002807.1 | 0.304303043 | 0.000118322 |
| HDAC6 | AP002807.1 | 0.445939017 | 6.06E-09 |
| IKBKB | AP002807.1 | 0.34738364 | 9.48E-06 |
| MAP2K7 | AP002807.1 | 0.35018717 | 7.94E-06 |
| MAPK9 | AP002807.1 | 0.302622733 | 0.000129576 |
| PEX14 | AP002807.1 | 0.311660043 | 7.90E-05 |
| RAF1 | AP002807.1 | 0.320506125 | 4.79E-05 |
| STK11 | AP002807.1 | 0.456914761 | 2.29E-09 |
| TSC2 | AP002807.1 | 0.304747253 | 0.000115503 |
| ULK3 | AP002807.1 | 0.372001181 | 1.88E-06 |
| WDR45 | AP002807.1 | 0.41225272 | 9.83E-08 |
| ARNT | SOX21-AS1 | 0.392029019 | 4.54E-07 |
| ATF6 | SOX21-AS1 | 0.341726688 | 1.35E-05 |
| BAK1 | SOX21-AS1 | -0.310372289 | 8.48E-05 |
| BECN1 | SOX21-AS1 | 0.335736154 | 1.95E-05 |
| BIRC6 | SOX21-AS1 | 0.466681987 | 9.32E-10 |
| CASP3 | SOX21-AS1 | 0.323425464 | 4.04E-05 |
| CD46 | SOX21-AS1 | 0.326833826 | 3.31E-05 |
| CDKN1B | SOX21-AS1 | 0.305812429 | 0.000108997 |
| EIF2AK3 | SOX21-AS1 | 0.662416539 | 6.19E-21 |
| EIF4G1 | SOX21-AS1 | 0.312363342 | 7.59E-05 |
| FOXO3 | SOX21-AS1 | 0.396394352 | 3.29E-07 |
| GABARAPL1 | SOX21-AS1 | 0.313515517 | 7.12E-05 |
| IKBKE | SOX21-AS1 | 0.31728153 | 5.76E-05 |
| ITGA3 | SOX21-AS1 | -0.41578439 | 7.44E-08 |
| KIF5B | SOX21-AS1 | 0.374611974 | 1.57E-06 |
| KLHL24 | SOX21-AS1 | 0.380160489 | 1.07E-06 |
| NBR1 | SOX21-AS1 | 0.433995201 | 1.69E-08 |
| NCKAP1 | SOX21-AS1 | 0.308132334 | 9.60E-05 |
| NFE2L2 | SOX21-AS1 | 0.50171682 | 2.93E-11 |
| PIK3R4 | SOX21-AS1 | 0.417622942 | 6.43E-08 |
| PINK1 | SOX21-AS1 | -0.338832668 | 1.61E-05 |
| RAB5A | SOX21-AS1 | 0.30104142 | 0.000141073 |
| RB1CC1 | SOX21-AS1 | 0.395466011 | 3.53E-07 |
| RPS6KB1 | SOX21-AS1 | 0.396197601 | 3.34E-07 |
| TP53INP2 | SOX21-AS1 | 0.443435415 | 7.54E-09 |
| UVRAG | SOX21-AS1 | 0.302825869 | 0.000128164 |
| VAMP3 | SOX21-AS1 | -0.301421722 | 0.000138224 |
| ARNT | AC104088.1 | 0.321610891 | 4.49E-05 |
| ATF6 | AC104088.1 | 0.347319427 | 9.52E-06 |
| ATG2B | AC104088.1 | 0.301446264 | 0.000138042 |
| BECN1 | AC104088.1 | 0.324271089 | 3.85E-05 |
| EIF4G1 | AC104088.1 | 0.316787815 | 5.92E-05 |
| GABARAPL1 | AC104088.1 | 0.305344616 | 0.000111811 |
| KIF5B | AC104088.1 | 0.346151286 | 1.02E-05 |
| MAPK1 | AC104088.1 | 0.314804633 | 6.62E-05 |
| NBR1 | AC104088.1 | 0.324986739 | 3.69E-05 |
| NCKAP1 | AC104088.1 | 0.44095815 | 9.33E-09 |
| RAB24 | AC104088.1 | -0.353197847 | 6.55E-06 |
| TP63 | AC104088.1 | 0.457719387 | 2.13E-09 |
| USP10 | AC104088.1 | 0.388324625 | 5.95E-07 |
| DDIT3 | AC002467.1 | 0.356138197 | 5.42E-06 |
| TM9SF1 | AC002467.1 | -0.314146232 | 6.87E-05 |
| ATIC | AC012213.4 | 0.419752663 | 5.42E-08 |
| BIRC5 | AC012213.4 | 0.34363387 | 1.20E-05 |
| GABARAPL2 | AC012213.4 | 0.462393491 | 1.39E-09 |
| HDAC6 | AC012213.4 | 0.324116913 | 3.89E-05 |
| MAPK8IP1 | AC012213.4 | 0.608547789 | 4.49E-17 |
| PRKAR1A | AC012213.4 | 0.371188616 | 1.99E-06 |
| ATG9B | AP003068.2 | 0.395589281 | 3.50E-07 |
| CAPN1 | AP003068.2 | 0.469887082 | 6.90E-10 |
| NFE2L2 | AP003068.2 | 0.303241953 | 0.000125317 |
| TP53INP2 | AP003068.2 | 0.441099135 | 9.22E-09 |
| HDAC6 | AC068473.5 | 0.318843976 | 5.27E-05 |
| ITGA3 | AC068473.5 | -0.348723262 | 8.71E-06 |
| ARSB | AP001542.3 | -0.312533823 | 7.52E-05 |
| ATG16L2 | AP001542.3 | 0.526974201 | 1.87E-12 |
| DAPK2 | AP001542.3 | 0.302810427 | 0.000128271 |
| FOXO1 | AP001542.3 | -0.395934127 | 3.41E-07 |
| ATF4 | GAS5 | 0.423761943 | 3.92E-08 |
| ATG16L2 | GAS5 | 0.317888878 | 5.56E-05 |
| ATG4B | GAS5 | 0.357825327 | 4.85E-06 |
| ATIC | GAS5 | 0.300790471 | 0.000142983 |
| BNIP1 | GAS5 | 0.329633954 | 2.81E-05 |
| CAPN10 | GAS5 | 0.466768253 | 9.25E-10 |
| EEF2 | GAS5 | 0.322413078 | 4.29E-05 |
| EIF4G1 | GAS5 | -0.332877801 | 2.32E-05 |
| LAMP2 | GAS5 | -0.354500627 | 6.02E-06 |
| MAPK1 | GAS5 | -0.363091731 | 3.43E-06 |
| ULK3 | GAS5 | 0.333124717 | 2.28E-05 |
| WDR45 | GAS5 | 0.347534071 | 9.40E-06 |
| ARNT | OIP5-AS1 | 0.578580222 | 3.16E-15 |
| ARSB | OIP5-AS1 | 0.47509738 | 4.21E-10 |
| ATF6 | OIP5-AS1 | 0.425663042 | 3.36E-08 |
| ATG4C | OIP5-AS1 | 0.401349473 | 2.27E-07 |
| BCL2 | OIP5-AS1 | 0.429430359 | 2.47E-08 |
| BIRC6 | OIP5-AS1 | 0.518789186 | 4.68E-12 |
| BNIP3 | OIP5-AS1 | -0.314453877 | 6.75E-05 |
| CAMKK2 | OIP5-AS1 | 0.304500926 | 0.000117058 |
| CASP3 | OIP5-AS1 | 0.394346718 | 3.83E-07 |
| CASP4 | OIP5-AS1 | -0.345154442 | 1.09E-05 |
| CCR2 | OIP5-AS1 | 0.35577262 | 5.55E-06 |
| CD46 | OIP5-AS1 | 0.465525104 | 1.04E-09 |
| CDKN1B | OIP5-AS1 | 0.454414858 | 2.86E-09 |
| DRAM1 | OIP5-AS1 | 0.332217698 | 2.41E-05 |
| EDEM1 | OIP5-AS1 | 0.361956285 | 3.70E-06 |
| EEF2K | OIP5-AS1 | 0.412888269 | 9.35E-08 |
| EIF2AK3 | OIP5-AS1 | 0.53852233 | 4.92E-13 |
| FAS | OIP5-AS1 | 0.31077467 | 8.30E-05 |
| FOXO3 | OIP5-AS1 | 0.325714354 | 3.54E-05 |
| HDAC6 | OIP5-AS1 | 0.50954277 | 1.28E-11 |
| IKBKE | OIP5-AS1 | 0.383422697 | 8.46E-07 |
| ITGA3 | OIP5-AS1 | -0.33537447 | 1.99E-05 |
| ITPR1 | OIP5-AS1 | 0.375242081 | 1.50E-06 |
| KIF5B | OIP5-AS1 | 0.340986049 | 1.41E-05 |
| KLHL24 | OIP5-AS1 | 0.486531947 | 1.38E-10 |
| MAP2K7 | OIP5-AS1 | 0.339309427 | 1.57E-05 |
| MAPK1 | OIP5-AS1 | 0.318476884 | 5.38E-05 |
| MAPK8 | OIP5-AS1 | 0.366077196 | 2.81E-06 |
| MBTPS2 | OIP5-AS1 | 0.324816475 | 3.73E-05 |
| MTOR | OIP5-AS1 | 0.366706158 | 2.69E-06 |
| NBR1 | OIP5-AS1 | 0.440451024 | 9.74E-09 |
| PARP1 | OIP5-AS1 | 0.5052658 | 2.02E-11 |
| PIK3C3 | OIP5-AS1 | 0.45335275 | 3.15E-09 |
| PIK3R4 | OIP5-AS1 | 0.462696305 | 1.35E-09 |
| PINK1 | OIP5-AS1 | -0.305351488 | 0.000111769 |
| PTK6 | OIP5-AS1 | -0.33011246 | 2.73E-05 |
| RAB33B | OIP5-AS1 | 0.437955444 | 1.21E-08 |
| RAF1 | OIP5-AS1 | 0.401797674 | 2.20E-07 |
| RB1CC1 | OIP5-AS1 | 0.339128392 | 1.58E-05 |
| RPS6KB1 | OIP5-AS1 | 0.314806884 | 6.62E-05 |
| SIRT1 | OIP5-AS1 | 0.482681187 | 2.01E-10 |
| TP53 | OIP5-AS1 | 0.415520771 | 7.60E-08 |
| TSC1 | OIP5-AS1 | 0.465359569 | 1.05E-09 |
| TSC2 | OIP5-AS1 | 0.360186758 | 4.16E-06 |
| ULK2 | OIP5-AS1 | 0.408076889 | 1.36E-07 |
| ULK3 | OIP5-AS1 | 0.359129194 | 4.45E-06 |
| UVRAG | OIP5-AS1 | 0.36786298 | 2.49E-06 |
| VAMP7 | OIP5-AS1 | 0.345159636 | 1.09E-05 |
| ATIC | SNHG21 | 0.329318206 | 2.86E-05 |
| CAPN1 | SNHG21 | -0.333127795 | 2.28E-05 |
| CAPN10 | SNHG21 | 0.339930876 | 1.51E-05 |
| ITGA3 | SNHG21 | -0.389971866 | 5.28E-07 |
| ITGB4 | SNHG21 | -0.31797151 | 5.53E-05 |
| MAPK8IP1 | SNHG21 | 0.383826107 | 8.22E-07 |
| PELP1 | SNHG21 | 0.3054856 | 0.000110956 |
| PIK3C3 | SNHG21 | 0.428689406 | 2.62E-08 |
| RHEB | SNHG21 | 0.35469697 | 5.95E-06 |
| ST13 | SNHG21 | 0.353010475 | 6.63E-06 |
| ULK3 | SNHG21 | 0.492635072 | 7.46E-11 |
| UVRAG | SNHG21 | 0.41255642 | 9.59E-08 |
| VAMP3 | SNHG21 | -0.30943764 | 8.93E-05 |
| BAG3 | SH3PXD2A-AS1 | 0.417686131 | 6.40E-08 |
| BAX | SH3PXD2A-AS1 | -0.350632141 | 7.72E-06 |
| BCL2 | SH3PXD2A-AS1 | -0.311716818 | 7.87E-05 |
| CXCR4 | SH3PXD2A-AS1 | -0.322522485 | 4.26E-05 |
| DRAM1 | SH3PXD2A-AS1 | -0.314060186 | 6.91E-05 |
| EIF2S1 | SH3PXD2A-AS1 | 0.303879665 | 0.000121068 |
| FAS | SH3PXD2A-AS1 | -0.300620648 | 0.000144288 |
| GABARAP | SH3PXD2A-AS1 | -0.386822541 | 6.63E-07 |
| HDAC6 | SH3PXD2A-AS1 | -0.333489013 | 2.23E-05 |
| HIF1A | SH3PXD2A-AS1 | 0.314481785 | 6.74E-05 |
| PTK6 | SH3PXD2A-AS1 | 0.384338111 | 7.92E-07 |
| ATG10 | AC090617.5 | 0.43739292 | 1.27E-08 |
| ATG4B | AC090617.5 | 0.448810647 | 4.71E-09 |
| ATG4C | AC090617.5 | 0.302668441 | 0.000129257 |
| ATG4D | AC090617.5 | 0.492269407 | 7.74E-11 |
| ATG7 | AC090617.5 | 0.361183066 | 3.89E-06 |
| BAX | AC090617.5 | 0.452884703 | 3.28E-09 |
| BID | AC090617.5 | 0.369068471 | 2.29E-06 |
| BNIP1 | AC090617.5 | 0.408312314 | 1.33E-07 |
| CAPN10 | AC090617.5 | 0.453423618 | 3.13E-09 |
| CAPNS1 | AC090617.5 | 0.430880007 | 2.19E-08 |
| CDKN2A | AC090617.5 | 0.508508362 | 1.43E-11 |
| CLN3 | AC090617.5 | 0.3584991 | 4.64E-06 |
| DAPK2 | AC090617.5 | 0.314524702 | 6.73E-05 |
| GABARAP | AC090617.5 | 0.596393343 | 2.66E-16 |
| HDAC6 | AC090617.5 | 0.303725885 | 0.00012208 |
| IKBKE | AC090617.5 | 0.314318106 | 6.81E-05 |
| ITGA6 | AC090617.5 | -0.308486469 | 9.41E-05 |
| MAP1LC3A | AC090617.5 | 0.407090737 | 1.47E-07 |
| MAP2K7 | AC090617.5 | 0.352535284 | 6.83E-06 |
| MTMR14 | AC090617.5 | 0.485687601 | 1.50E-10 |
| PELP1 | AC090617.5 | 0.541432302 | 3.49E-13 |
| PEX14 | AC090617.5 | 0.354955021 | 5.85E-06 |
| PINK1 | AC090617.5 | -0.36826374 | 2.42E-06 |
| PRKAB1 | AC090617.5 | 0.3907283 | 5.00E-07 |
| STK11 | AC090617.5 | 0.399312482 | 2.65E-07 |
| WDR45 | AC090617.5 | 0.487328283 | 1.27E-10 |
| CX3CL1 | AP003555.2 | 0.413834522 | 8.68E-08 |
| EGFR | CD44-AS1 | 0.45524982 | 2.66E-09 |
| GOPC | CD44-AS1 | 0.36679256 | 2.68E-06 |
| MTMR14 | CD44-AS1 | -0.300571716 | 0.000144667 |
| VEGFA | CD44-AS1 | 0.308225859 | 9.55E-05 |
| ARSB | PICSAR | -0.361488609 | 3.81E-06 |
| BAK1 | PICSAR | 0.373412228 | 1.71E-06 |
| CASP1 | PICSAR | 0.595400342 | 3.06E-16 |
| CASP4 | PICSAR | 0.511408423 | 1.05E-11 |
| FOXO1 | PICSAR | -0.33058624 | 2.66E-05 |
| HSPA8 | PICSAR | 0.370451017 | 2.09E-06 |
| PTK6 | PICSAR | 0.327985224 | 3.10E-05 |
| ATG10 | AC092747.4 | 0.354930313 | 5.86E-06 |
| ATG12 | AC092747.4 | 0.377810762 | 1.26E-06 |
| ATG16L2 | AC092747.4 | 0.447002033 | 5.52E-09 |
| ATG4B | AC092747.4 | 0.380707173 | 1.03E-06 |
| ATG4C | AC092747.4 | 0.38231626 | 9.15E-07 |
| CAPN10 | AC092747.4 | 0.387762777 | 6.20E-07 |
| CDKN1B | AC092747.4 | 0.307654664 | 9.85E-05 |
| CFLAR | AC092747.4 | 0.305411897 | 0.000111402 |
| DAPK2 | AC092747.4 | 0.381293749 | 9.84E-07 |
| EIF4G1 | AC092747.4 | -0.33583308 | 1.94E-05 |
| HDAC1 | AC092747.4 | 0.368180514 | 2.44E-06 |
| HDAC6 | AC092747.4 | 0.360267179 | 4.13E-06 |
| IKBKB | AC092747.4 | 0.301714903 | 0.000136065 |
| PEX14 | AC092747.4 | 0.368752299 | 2.34E-06 |
| STK11 | AC092747.4 | 0.341396367 | 1.38E-05 |
| TSC2 | AC092747.4 | 0.334779167 | 2.06E-05 |
| ULK3 | AC092747.4 | 0.352158435 | 7.00E-06 |
| APOL1 | U62317.2 | 0.350524908 | 7.77E-06 |
| ATF4 | U62317.2 | 0.339721434 | 1.53E-05 |
| ATG10 | U62317.2 | 0.326263193 | 3.43E-05 |
| ATG12 | U62317.2 | 0.313230881 | 7.23E-05 |
| ATG16L2 | U62317.2 | 0.331870602 | 2.46E-05 |
| ATG3 | U62317.2 | 0.363863051 | 3.26E-06 |
| ATG4A | U62317.2 | 0.38021835 | 1.06E-06 |
| ATG4B | U62317.2 | 0.399718721 | 2.57E-07 |
| BAX | U62317.2 | 0.410758956 | 1.10E-07 |
| BIRC6 | U62317.2 | -0.402367629 | 2.10E-07 |
| BNIP1 | U62317.2 | 0.408608243 | 1.30E-07 |
| CAPN10 | U62317.2 | 0.375558763 | 1.47E-06 |
| CASP1 | U62317.2 | 0.335832424 | 1.94E-05 |
| CDKN2A | U62317.2 | 0.47774174 | 3.26E-10 |
| CFLAR | U62317.2 | 0.300749029 | 0.0001433 |
| CTSD | U62317.2 | 0.306891028 | 0.000102759 |
| CX3CL1 | U62317.2 | 0.399525713 | 2.61E-07 |
| DLC1 | U62317.2 | -0.321714948 | 4.47E-05 |
| EEF2 | U62317.2 | -0.31153371 | 7.95E-05 |
| IFNG | U62317.2 | 0.344154432 | 1.16E-05 |
| MAPK1 | U62317.2 | -0.307660575 | 9.85E-05 |
| MTMR14 | U62317.2 | 0.304437092 | 0.000117464 |
| NCKAP1 | U62317.2 | -0.368856692 | 2.33E-06 |
| PEX14 | U62317.2 | 0.44277054 | 7.98E-09 |
| RAB24 | U62317.2 | 0.518480884 | 4.84E-12 |
| RPTOR | U62317.2 | -0.305758234 | 0.000109319 |
| STK11 | U62317.2 | 0.365025223 | 3.01E-06 |
| TNFSF10 | U62317.2 | 0.322787532 | 4.20E-05 |
| ULK3 | U62317.2 | 0.341004349 | 1.41E-05 |
| WDFY3 | U62317.2 | -0.339095834 | 1.59E-05 |
| WDR45 | U62317.2 | 0.504185879 | 2.26E-11 |
| ATG2B | AL049840.1 | 0.389389101 | 5.51E-07 |
| EDEM1 | AL049840.1 | 0.303772569 | 0.000121772 |
| EGFR | AL049840.1 | 0.33431718 | 2.12E-05 |
| GOPC | AL049840.1 | 0.332856651 | 2.32E-05 |
| MTOR | AL049840.1 | 0.332341382 | 2.39E-05 |
| SPNS1 | AL049840.1 | 0.364135415 | 3.20E-06 |
| TSC1 | AL049840.1 | 0.422421105 | 4.38E-08 |
| VEGFA | AL049840.1 | 0.308927513 | 9.19E-05 |
| CDKN1B | U47924.2 | 0.434909208 | 1.56E-08 |
| CLN3 | U47924.2 | 0.423120072 | 4.13E-08 |
| ITGA3 | U47924.2 | -0.343194761 | 1.23E-05 |
| PINK1 | U47924.2 | -0.338961346 | 1.60E-05 |
| ATF4 | MIATNB | 0.30508264 | 0.000113416 |
| ATG4B | MIATNB | 0.391685529 | 4.66E-07 |
| BNIP1 | MIATNB | 0.389326641 | 5.53E-07 |
| CAPN10 | MIATNB | 0.372094585 | 1.87E-06 |
| CFLAR | MIATNB | 0.318651495 | 5.32E-05 |
| GOPC | MIATNB | -0.349972986 | 8.05E-06 |
| USP10 | MIATNB | -0.32236618 | 4.30E-05 |
| WDR45 | MIATNB | 0.415085552 | 7.86E-08 |
| ARSB | PINK1-AS | 0.316439081 | 6.04E-05 |
| ATG4C | PINK1-AS | 0.453636267 | 3.07E-09 |
| BCL2 | PINK1-AS | 0.481739456 | 2.21E-10 |
| BIRC6 | PINK1-AS | 0.395378816 | 3.55E-07 |
| CDKN1B | PINK1-AS | 0.385978787 | 7.05E-07 |
| CXCR4 | PINK1-AS | 0.311875934 | 7.80E-05 |
| DRAM1 | PINK1-AS | 0.403069879 | 1.99E-07 |
| EEF2K | PINK1-AS | 0.451112567 | 3.84E-09 |
| EIF2AK3 | PINK1-AS | 0.328506385 | 3.00E-05 |
| FAS | PINK1-AS | 0.345261869 | 1.08E-05 |
| HDAC1 | PINK1-AS | 0.308540105 | 9.39E-05 |
| HDAC6 | PINK1-AS | 0.331299209 | 2.54E-05 |
| ITPR1 | PINK1-AS | 0.37373018 | 1.67E-06 |
| MAPK8 | PINK1-AS | 0.472885517 | 5.19E-10 |
| MTOR | PINK1-AS | 0.480379532 | 2.52E-10 |
| NAF1 | PINK1-AS | 0.309461992 | 8.92E-05 |
| NFKB1 | PINK1-AS | 0.324599573 | 3.78E-05 |
| NKX2-3 | PINK1-AS | 0.364532339 | 3.11E-06 |
| PARP1 | PINK1-AS | 0.38491163 | 7.61E-07 |
| PRKAB1 | PINK1-AS | 0.331296457 | 2.55E-05 |
| SH3GLB1 | PINK1-AS | 0.318884572 | 5.25E-05 |
| SIRT1 | PINK1-AS | 0.306571099 | 0.000104573 |
| TP53 | PINK1-AS | 0.34650187 | 1.00E-05 |
| TP73 | PINK1-AS | 0.369111641 | 2.29E-06 |
| TSC1 | PINK1-AS | 0.325117998 | 3.67E-05 |
| TSC2 | PINK1-AS | 0.416846782 | 6.84E-08 |
| ATG16L2 | LINC02298 | 0.352048977 | 7.05E-06 |
| BID | LINC02298 | 0.358592178 | 4.61E-06 |
| DIRAS3 | LINC02298 | 0.324375125 | 3.83E-05 |
| FKBP1B | LINC02298 | 0.31786589 | 5.57E-05 |
| ARNT | AL021707.3 | 0.308554909 | 9.38E-05 |
| BECN1 | AL021707.3 | 0.340315066 | 1.47E-05 |
| CD46 | AL021707.3 | 0.357582007 | 4.93E-06 |
| CDKN1B | AL021707.3 | 0.309635433 | 8.84E-05 |
| CXCR4 | AL021707.3 | 0.382193673 | 9.23E-07 |
| EEF2K | AL021707.3 | 0.335271995 | 2.00E-05 |
| EIF2AK3 | AL021707.3 | 0.366363821 | 2.75E-06 |
| HDAC6 | AL021707.3 | 0.430491851 | 2.26E-08 |
| IKBKB | AL021707.3 | 0.448218419 | 4.96E-09 |
| MAP2K7 | AL021707.3 | 0.353719203 | 6.33E-06 |
| MAPK8 | AL021707.3 | 0.351649963 | 7.23E-06 |
| PIK3R4 | AL021707.3 | 0.406218937 | 1.57E-07 |
| RAF1 | AL021707.3 | 0.431855437 | 2.02E-08 |
| SIRT1 | AL021707.3 | 0.307704079 | 9.83E-05 |
| TSC1 | AL021707.3 | 0.480998052 | 2.38E-10 |
| TSC2 | AL021707.3 | 0.337996715 | 1.70E-05 |
| ULK3 | AL021707.3 | 0.361337999 | 3.85E-06 |
| UVRAG | AL021707.3 | 0.323128163 | 4.12E-05 |
| ARNT | AC006504.8 | -0.403114433 | 1.99E-07 |
| ATF4 | AC006504.8 | 0.342274291 | 1.31E-05 |
| ATF6 | AC006504.8 | -0.312115757 | 7.70E-05 |
| ATG2B | AC006504.8 | -0.316033676 | 6.18E-05 |
| ATG4B | AC006504.8 | 0.389686552 | 5.39E-07 |
| BAX | AC006504.8 | 0.379115448 | 1.15E-06 |
| BIRC5 | AC006504.8 | 0.376743638 | 1.35E-06 |
| BIRC6 | AC006504.8 | -0.315069661 | 6.52E-05 |
| BNIP1 | AC006504.8 | 0.320273719 | 4.85E-05 |
| CAPN10 | AC006504.8 | 0.380943609 | 1.01E-06 |
| FKBP1A | AC006504.8 | 0.344858515 | 1.11E-05 |
| FOXO1 | AC006504.8 | -0.352048004 | 7.05E-06 |
| LAMP2 | AC006504.8 | -0.311844985 | 7.82E-05 |
| MAPK1 | AC006504.8 | -0.378793787 | 1.17E-06 |
| MLST8 | AC006504.8 | 0.327309282 | 3.22E-05 |
| RB1 | AC006504.8 | -0.33082295 | 2.62E-05 |
| USP10 | AC006504.8 | -0.326518657 | 3.38E-05 |
| WDR45 | AC006504.8 | 0.379784009 | 1.09E-06 |
| ZFYVE1 | AC006504.8 | -0.309199433 | 9.05E-05 |
| CASP1 | LINC01094 | 0.312662862 | 7.47E-05 |
| PINK1 | LINC01094 | 0.314145138 | 6.87E-05 |
| ATIC | AC023043.1 | 0.343366911 | 1.22E-05 |
| BIRC5 | AC023043.1 | 0.30212962 | 0.000133064 |
| GABARAPL2 | AC023043.1 | 0.311467466 | 7.98E-05 |
| MAPK8IP1 | AC023043.1 | 0.427711674 | 2.84E-08 |
| PRKAR1A | AC023043.1 | 0.328379697 | 3.03E-05 |
| ULK2 | AC023043.1 | 0.36623961 | 2.78E-06 |
| VAMP7 | AC023043.1 | 0.3011947 | 0.000139918 |
| CAPN10 | AC062037.2 | 0.301831039 | 0.000135218 |
| IKBKB | AC062037.2 | 0.342218909 | 1.31E-05 |
| TSC1 | AC062037.2 | 0.318418805 | 5.40E-05 |
| STK11 | CIRBP-AS1 | 0.407048218 | 1.47E-07 |
| AMBRA1 | AL359091.5 | -0.339524689 | 1.55E-05 |
| ARNT | AL359091.5 | -0.339802852 | 1.52E-05 |
| BECN1 | AL359091.5 | -0.391073984 | 4.87E-07 |
| BIRC6 | AL359091.5 | -0.394485675 | 3.79E-07 |
| CD46 | AL359091.5 | -0.316091228 | 6.16E-05 |
| EEF2K | AL359091.5 | -0.31294094 | 7.35E-05 |
| EIF2AK3 | AL359091.5 | -0.37558721 | 1.47E-06 |
| EIF4EBP1 | AL359091.5 | 0.338649307 | 1.63E-05 |
| FOXO3 | AL359091.5 | -0.325694736 | 3.54E-05 |
| GABARAPL2 | AL359091.5 | 0.335072086 | 2.03E-05 |
| MAP2K7 | AL359091.5 | -0.333947887 | 2.17E-05 |
| NBR1 | AL359091.5 | -0.424972239 | 3.56E-08 |
| PARP1 | AL359091.5 | -0.328384606 | 3.03E-05 |
| PIK3R4 | AL359091.5 | -0.36973814 | 2.19E-06 |
| RPS6KB1 | AL359091.5 | -0.372779826 | 1.78E-06 |
| SIRT1 | AL359091.5 | -0.310312849 | 8.51E-05 |
| ARNT | SNHG9 | -0.33735825 | 1.77E-05 |
| ATF4 | SNHG9 | 0.329627024 | 2.81E-05 |
| ATG16L2 | SNHG9 | 0.384614223 | 7.77E-07 |
| ATG3 | SNHG9 | 0.311429341 | 8.00E-05 |
| ATG4B | SNHG9 | 0.483171847 | 1.92E-10 |
| BAX | SNHG9 | 0.506768564 | 1.72E-11 |
| BIRC5 | SNHG9 | 0.428435917 | 2.68E-08 |
| BIRC6 | SNHG9 | -0.398963794 | 2.72E-07 |
| BNIP1 | SNHG9 | 0.373475406 | 1.70E-06 |
| CANX | SNHG9 | -0.323096042 | 4.12E-05 |
| CAPN10 | SNHG9 | 0.437573579 | 1.25E-08 |
| CLN3 | SNHG9 | 0.367813754 | 2.50E-06 |
| FKBP1A | SNHG9 | 0.311736719 | 7.86E-05 |
| FOXO1 | SNHG9 | -0.326382068 | 3.40E-05 |
| GABARAPL2 | SNHG9 | 0.459944826 | 1.74E-09 |
| GOPC | SNHG9 | -0.353640756 | 6.37E-06 |
| ITGB1 | SNHG9 | -0.312826253 | 7.40E-05 |
| KIF5B | SNHG9 | -0.349858347 | 8.11E-06 |
| LAMP2 | SNHG9 | -0.324441724 | 3.81E-05 |
| MAPK1 | SNHG9 | -0.435098431 | 1.54E-08 |
| MLST8 | SNHG9 | 0.467381653 | 8.73E-10 |
| NBR1 | SNHG9 | -0.323160908 | 4.11E-05 |
| NCKAP1 | SNHG9 | -0.356108447 | 5.43E-06 |
| NPC1 | SNHG9 | -0.319753689 | 5.00E-05 |
| PEX14 | SNHG9 | 0.364954609 | 3.03E-06 |
| RAB24 | SNHG9 | 0.341718347 | 1.35E-05 |
| STK11 | SNHG9 | 0.380060988 | 1.07E-06 |
| TM9SF1 | SNHG9 | -0.342025972 | 1.33E-05 |
| WDFY3 | SNHG9 | -0.338311809 | 1.67E-05 |
| WDR45 | SNHG9 | 0.395955717 | 3.40E-07 |
| ATF4 | UBR5-AS1 | 0.348104721 | 9.06E-06 |
| ATG12 | UBR5-AS1 | 0.330278223 | 2.70E-05 |
| ATG16L2 | UBR5-AS1 | 0.382693068 | 8.91E-07 |
| BAX | UBR5-AS1 | 0.321008149 | 4.65E-05 |
| CAPN10 | UBR5-AS1 | 0.412178024 | 9.88E-08 |
| CDKN1B | UBR5-AS1 | 0.329215678 | 2.88E-05 |
| EIF4G1 | UBR5-AS1 | -0.35586201 | 5.51E-06 |
| GABARAP | UBR5-AS1 | 0.317244389 | 5.77E-05 |
| IKBKB | UBR5-AS1 | 0.350113459 | 7.98E-06 |
| MAP2K7 | UBR5-AS1 | 0.304107536 | 0.000119583 |
| MAPK8IP1 | UBR5-AS1 | 0.371792475 | 1.91E-06 |
| ATG10 | LINC00992 | 0.302196591 | 0.000132585 |
| DIRAS3 | LINC00992 | 0.469547489 | 7.13E-10 |
| BID | BX322562.1 | 0.350105125 | 7.98E-06 |
| EIF4G1 | BX322562.1 | -0.301513565 | 0.000137544 |
| ATG2B | LINC00638 | 0.339427518 | 1.56E-05 |
| GAA | LINC00638 | 0.319493301 | 5.07E-05 |
| RPTOR | LINC00638 | 0.317954924 | 5.54E-05 |
| TM9SF1 | LINC00638 | 0.329965302 | 2.76E-05 |
| ATG2B | AC106900.2 | -0.314211198 | 6.85E-05 |
| ATG4B | AC106900.2 | 0.436569578 | 1.36E-08 |
| BAX | AC106900.2 | 0.398595281 | 2.79E-07 |
| BIRC6 | AC106900.2 | -0.300272341 | 0.000147002 |
| BNIP1 | AC106900.2 | 0.537424297 | 5.60E-13 |
| CAPNS1 | AC106900.2 | 0.515025537 | 7.07E-12 |
| CDKN2A | AC106900.2 | 0.446016801 | 6.02E-09 |
| EIF4G1 | AC106900.2 | -0.318414397 | 5.40E-05 |
| FKBP1A | AC106900.2 | 0.450561662 | 4.04E-09 |
| GABARAP | AC106900.2 | 0.353835295 | 6.29E-06 |
| HSPA8 | AC106900.2 | -0.346528541 | 1.00E-05 |
| MAP1LC3A | AC106900.2 | 0.41810486 | 6.19E-08 |
| MAPK1 | AC106900.2 | -0.33427708 | 2.13E-05 |
| PEX14 | AC106900.2 | 0.408580796 | 1.31E-07 |
| RAC1 | AC106900.2 | 0.389015309 | 5.66E-07 |
| SPNS1 | AC106900.2 | -0.306200148 | 0.000106715 |
| WDR45 | AC106900.2 | 0.381194825 | 9.91E-07 |
| ARNT | LYRM4-AS1 | 0.341942475 | 1.33E-05 |
| ATF6 | LYRM4-AS1 | 0.32737853 | 3.21E-05 |
| ATG2B | LYRM4-AS1 | 0.305237952 | 0.000112462 |
| BIRC6 | LYRM4-AS1 | 0.447581074 | 5.25E-09 |
| EEF2K | LYRM4-AS1 | 0.342593916 | 1.28E-05 |
| EIF2AK2 | LYRM4-AS1 | 0.305178487 | 0.000112826 |
| EIF4G1 | LYRM4-AS1 | 0.373394604 | 1.71E-06 |
| KLHL24 | LYRM4-AS1 | 0.388733635 | 5.78E-07 |
| MAPK1 | LYRM4-AS1 | 0.303503058 | 0.000123561 |
| NBR1 | LYRM4-AS1 | 0.355424479 | 5.67E-06 |
| PARP1 | LYRM4-AS1 | 0.320415502 | 4.81E-05 |
| FKBP1B | MANCR | 0.305220454 | 0.000112569 |
| HDAC6 | MANCR | -0.314319142 | 6.81E-05 |
| ITGA6 | MANCR | 0.410356568 | 1.14E-07 |
| ITGB1 | MANCR | 0.470934004 | 6.25E-10 |
| PTK6 | MANCR | 0.388618266 | 5.83E-07 |
| ATG16L2 | AC020910.4 | 0.42736162 | 2.92E-08 |
| ATG4B | AC020910.4 | 0.344624279 | 1.13E-05 |
| BAX | AC020910.4 | 0.323496639 | 4.03E-05 |
| CAPN10 | AC020910.4 | 0.446625856 | 5.71E-09 |
| CAPN2 | AC020910.4 | -0.315361736 | 6.42E-05 |
| DIRAS3 | AC020910.4 | 0.355174712 | 5.76E-06 |
| EIF4G1 | AC020910.4 | -0.30256208 | 0.000130001 |
| FOXO1 | AC020910.4 | -0.337298019 | 1.77E-05 |
| GABARAPL2 | AC020910.4 | 0.355100341 | 5.79E-06 |
| KIF5B | AC020910.4 | -0.301249124 | 0.00013951 |
| MAPK1 | AC020910.4 | -0.395279272 | 3.58E-07 |
| PEX14 | AC020910.4 | 0.319547693 | 5.06E-05 |
| WDR45 | AC020910.4 | 0.413970062 | 8.59E-08 |
| ATG4B | LINC01451 | 0.344367515 | 1.15E-05 |
| ATG4D | LINC01451 | 0.30857087 | 9.37E-05 |
| BAX | LINC01451 | 0.308702214 | 9.30E-05 |
| BID | LINC01451 | 0.352691958 | 6.77E-06 |
| CAPN10 | LINC01451 | 0.389913463 | 5.30E-07 |
| CDKN2A | LINC01451 | 0.351398765 | 7.35E-06 |
| FKBP1A | LINC01451 | 0.308182311 | 9.57E-05 |
| PINK1 | LINC01451 | -0.352122122 | 7.02E-06 |
| ATG9B | AC003965.2 | 0.392923576 | 4.26E-07 |
| CAPN2 | AC003965.2 | 0.364629155 | 3.09E-06 |
| EIF2AK3 | AC003965.2 | 0.33535069 | 1.99E-05 |
| TP53INP2 | AC003965.2 | 0.303230167 | 0.000125397 |
| ARSB | LINC00909 | 0.314540564 | 6.72E-05 |
| ATG10 | LINC00909 | 0.364402147 | 3.14E-06 |
| ATG4C | LINC00909 | 0.579857736 | 2.66E-15 |
| BCL2 | LINC00909 | 0.605255401 | 7.32E-17 |
| BNIP3 | LINC00909 | -0.33862152 | 1.63E-05 |
| CAPN10 | LINC00909 | 0.357816046 | 4.85E-06 |
| CASP3 | LINC00909 | 0.363871236 | 3.25E-06 |
| CCR2 | LINC00909 | 0.328868295 | 2.94E-05 |
| CD46 | LINC00909 | 0.316545506 | 6.00E-05 |
| CDKN1B | LINC00909 | 0.454982102 | 2.72E-09 |
| CXCR4 | LINC00909 | 0.495086522 | 5.81E-11 |
| DRAM1 | LINC00909 | 0.327566912 | 3.17E-05 |
| EEF2K | LINC00909 | 0.406783937 | 1.50E-07 |
| FAS | LINC00909 | 0.311340139 | 8.04E-05 |
| HDAC6 | LINC00909 | 0.567961894 | 1.29E-14 |
| IFNG | LINC00909 | 0.310559809 | 8.39E-05 |
| IKBKB | LINC00909 | 0.408536461 | 1.31E-07 |
| IKBKE | LINC00909 | 0.400387926 | 2.44E-07 |
| MAP1LC3B | LINC00909 | -0.326622754 | 3.36E-05 |
| MAP2K7 | LINC00909 | 0.530575801 | 1.24E-12 |
| MAPK8 | LINC00909 | 0.413376063 | 9.00E-08 |
| MAPK9 | LINC00909 | 0.310623556 | 8.37E-05 |
| MTMR14 | LINC00909 | 0.342074723 | 1.32E-05 |
| NKX2-3 | LINC00909 | 0.425900365 | 3.30E-08 |
| PARP1 | LINC00909 | 0.429852457 | 2.38E-08 |
| PIK3C3 | LINC00909 | 0.360177676 | 4.16E-06 |
| PIK3R4 | LINC00909 | 0.318000852 | 5.53E-05 |
| PRKAB1 | LINC00909 | 0.433979358 | 1.69E-08 |
| PTK6 | LINC00909 | -0.362538256 | 3.56E-06 |
| RAF1 | LINC00909 | 0.407443842 | 1.43E-07 |
| STK11 | LINC00909 | 0.407718267 | 1.40E-07 |
| TP53 | LINC00909 | 0.453043075 | 3.24E-09 |
| TP73 | LINC00909 | 0.476501309 | 3.67E-10 |
| TSC1 | LINC00909 | 0.434699301 | 1.59E-08 |
| TSC2 | LINC00909 | 0.534209196 | 8.15E-13 |
| ULK2 | LINC00909 | 0.338155928 | 1.68E-05 |
| ULK3 | LINC00909 | 0.39105111 | 4.88E-07 |
| WDR45 | LINC00909 | 0.321803832 | 4.44E-05 |
| ATF4 | LINC01589 | 0.320839621 | 4.70E-05 |
| DDIT3 | LINC01589 | 0.435261194 | 1.52E-08 |
| BIRC6 | AC147067.1 | -0.376592917 | 1.37E-06 |
| CASP1 | AC147067.1 | 0.501456705 | 3.01E-11 |
| CASP4 | AC147067.1 | 0.426767381 | 3.07E-08 |
| EIF2AK3 | AC147067.1 | -0.375061187 | 1.52E-06 |
| EIF4G1 | AC147067.1 | -0.301843077 | 0.000135131 |
| KIF5B | AC147067.1 | -0.321212484 | 4.60E-05 |
| MAPK1 | AC147067.1 | -0.377642875 | 1.27E-06 |
| PIK3R4 | AC147067.1 | -0.333157916 | 2.28E-05 |
| RPS6KB1 | AC147067.1 | -0.361162144 | 3.90E-06 |
| SIRT1 | AC147067.1 | -0.329329514 | 2.86E-05 |
| USP10 | AC147067.1 | -0.301279447 | 0.000139284 |
| VAMP3 | AC147067.1 | 0.33721594 | 1.78E-05 |
| ARNT | AC025183.1 | -0.321940391 | 4.41E-05 |
| ARSB | AC025183.1 | -0.306140578 | 0.000107062 |
| ATG4C | AC025183.1 | -0.351037636 | 7.52E-06 |
| CALCOCO2 | AC025183.1 | -0.401393779 | 2.26E-07 |
| DAPK2 | AC025183.1 | 0.345146042 | 1.09E-05 |
| PIK3R4 | AC025183.1 | -0.339233105 | 1.57E-05 |
| RAB5A | AC025183.1 | -0.312688315 | 7.46E-05 |
| ARSB | ZNF710-AS1 | 0.312609505 | 7.49E-05 |
| ATG10 | ZNF710-AS1 | 0.431000385 | 2.16E-08 |
| ATG2A | ZNF710-AS1 | 0.366212602 | 2.78E-06 |
| ATG4C | ZNF710-AS1 | 0.428069376 | 2.76E-08 |
| ATG7 | ZNF710-AS1 | 0.359693914 | 4.29E-06 |
| BCL2 | ZNF710-AS1 | 0.372656375 | 1.80E-06 |
| BNIP3 | ZNF710-AS1 | -0.338283928 | 1.67E-05 |
| CAPN10 | ZNF710-AS1 | 0.302922382 | 0.000127499 |
| CAPNS1 | ZNF710-AS1 | 0.303845908 | 0.00012129 |
| CCR2 | ZNF710-AS1 | 0.323249776 | 4.09E-05 |
| CDKN1B | ZNF710-AS1 | 0.456228086 | 2.43E-09 |
| CFLAR | ZNF710-AS1 | 0.301448143 | 0.000138028 |
| CX3CL1 | ZNF710-AS1 | 0.338930626 | 1.60E-05 |
| CXCR4 | ZNF710-AS1 | 0.510855999 | 1.11E-11 |
| DRAM1 | ZNF710-AS1 | 0.39922153 | 2.67E-07 |
| EEF2K | ZNF710-AS1 | 0.390720782 | 5.00E-07 |
| FAS | ZNF710-AS1 | 0.425126865 | 3.51E-08 |
| GABARAP | ZNF710-AS1 | 0.355836861 | 5.52E-06 |
| HDAC6 | ZNF710-AS1 | 0.529116527 | 1.47E-12 |
| HIF1A | ZNF710-AS1 | -0.318616244 | 5.34E-05 |
| HSP90AB1 | ZNF710-AS1 | -0.356826005 | 5.18E-06 |
| HSPA5 | ZNF710-AS1 | -0.418880675 | 5.82E-08 |
| IFNG | ZNF710-AS1 | 0.351103177 | 7.49E-06 |
| IKBKB | ZNF710-AS1 | 0.316878872 | 5.89E-05 |
| IKBKE | ZNF710-AS1 | 0.465650422 | 1.03E-09 |
| MAP2K7 | ZNF710-AS1 | 0.341281413 | 1.39E-05 |
| MAPK8 | ZNF710-AS1 | 0.405645782 | 1.64E-07 |
| MTMR14 | ZNF710-AS1 | 0.483108407 | 1.93E-10 |
| MYC | ZNF710-AS1 | -0.315519124 | 6.36E-05 |
| NKX2-3 | ZNF710-AS1 | 0.582522001 | 1.85E-15 |
| PARP1 | ZNF710-AS1 | 0.356176768 | 5.40E-06 |
| PRKAB1 | ZNF710-AS1 | 0.320811744 | 4.70E-05 |
| RAF1 | ZNF710-AS1 | 0.387967801 | 6.11E-07 |
| SH3GLB1 | ZNF710-AS1 | 0.324960215 | 3.70E-05 |
| SIRT1 | ZNF710-AS1 | 0.301763632 | 0.000135709 |
| STK11 | ZNF710-AS1 | 0.337477099 | 1.75E-05 |
| TP53 | ZNF710-AS1 | 0.555501868 | 6.28E-14 |
| TP73 | ZNF710-AS1 | 0.594580938 | 3.44E-16 |
| TSC2 | ZNF710-AS1 | 0.606674874 | 5.93E-17 |
| ULK3 | ZNF710-AS1 | 0.347348711 | 9.51E-06 |
| ARSB | LINC00963 | 0.3041732 | 0.000119158 |
| ATG4D | LINC00963 | 0.432390833 | 1.93E-08 |
| ATG7 | LINC00963 | 0.403670455 | 1.91E-07 |
| BAK1 | LINC00963 | -0.311753804 | 7.86E-05 |
| BCL2 | LINC00963 | 0.349947315 | 8.06E-06 |
| BNIP3 | LINC00963 | -0.300472507 | 0.000145437 |
| BNIP3L | LINC00963 | 0.372002716 | 1.88E-06 |
| CAPNS1 | LINC00963 | 0.327562437 | 3.18E-05 |
| CDKN1B | LINC00963 | 0.320229984 | 4.86E-05 |
| CDKN2A | LINC00963 | 0.429157036 | 2.52E-08 |
| EEF2K | LINC00963 | 0.329783419 | 2.78E-05 |
| GABARAP | LINC00963 | 0.318864752 | 5.26E-05 |
| HDAC6 | LINC00963 | 0.306733545 | 0.000103648 |
| IKBKE | LINC00963 | 0.425664091 | 3.36E-08 |
| KLHL24 | LINC00963 | 0.331904915 | 2.45E-05 |
| MAP2K7 | LINC00963 | 0.448194085 | 4.98E-09 |
| MTMR14 | LINC00963 | 0.345240119 | 1.09E-05 |
| NKX2-3 | LINC00963 | 0.303988073 | 0.000120359 |
| PIK3C3 | LINC00963 | 0.324818968 | 3.73E-05 |
| PINK1 | LINC00963 | -0.312587395 | 7.50E-05 |
| PRKAB1 | LINC00963 | 0.430080178 | 2.34E-08 |
| PTK6 | LINC00963 | -0.37828217 | 1.22E-06 |
| RAF1 | LINC00963 | 0.309147043 | 9.08E-05 |
| TP53 | LINC00963 | 0.371813667 | 1.90E-06 |
| TSC1 | LINC00963 | 0.399363687 | 2.64E-07 |
| TSC2 | LINC00963 | 0.315564294 | 6.34E-05 |
| WDR45 | LINC00963 | 0.309527115 | 8.89E-05 |
| BAX | AC107375.1 | 0.300908433 | 0.000142082 |
| BIRC5 | AC107375.1 | 0.336144236 | 1.90E-05 |
| CAPN10 | AC107375.1 | 0.395047228 | 3.64E-07 |
| CXCR4 | AC107375.1 | 0.365130332 | 2.99E-06 |
| HDAC6 | AC107375.1 | 0.495924855 | 5.33E-11 |
| IKBKB | AC107375.1 | 0.412766945 | 9.44E-08 |
| MAP2K7 | AC107375.1 | 0.391890915 | 4.59E-07 |
| MTMR14 | AC107375.1 | 0.311112145 | 8.14E-05 |
| RAF1 | AC107375.1 | 0.31991861 | 4.95E-05 |
| STK11 | AC107375.1 | 0.306260501 | 0.000106364 |
| TSC1 | AC107375.1 | 0.34076339 | 1.43E-05 |
| WDR45 | AC107375.1 | 0.387046355 | 6.53E-07 |
| ATG10 | AC010542.5 | 0.380561226 | 1.04E-06 |
| ATG16L2 | AC010542.5 | 0.382422596 | 9.08E-07 |
| ATG3 | AC010542.5 | 0.391371673 | 4.77E-07 |
| ATG4B | AC010542.5 | 0.447511621 | 5.28E-09 |
| BAX | AC010542.5 | 0.620371502 | 7.38E-18 |
| BIRC5 | AC010542.5 | 0.358790418 | 4.55E-06 |
| BNIP1 | AC010542.5 | 0.382385616 | 9.11E-07 |
| CAPN10 | AC010542.5 | 0.557212726 | 5.07E-14 |
| CAPNS1 | AC010542.5 | 0.331514379 | 2.51E-05 |
| CDKN2A | AC010542.5 | 0.434544953 | 1.61E-08 |
| CLN3 | AC010542.5 | 0.372030932 | 1.87E-06 |
| CXCR4 | AC010542.5 | 0.452144654 | 3.51E-09 |
| FKBP1A | AC010542.5 | 0.30625367 | 0.000106403 |
| GABARAP | AC010542.5 | 0.377924777 | 1.25E-06 |
| HDAC6 | AC010542.5 | 0.415961874 | 7.34E-08 |
| HIF1A | AC010542.5 | -0.329662606 | 2.80E-05 |
| HSPA8 | AC010542.5 | -0.309805687 | 8.75E-05 |
| IFNG | AC010542.5 | 0.320050436 | 4.91E-05 |
| ITGA6 | AC010542.5 | -0.304451388 | 0.000117373 |
| ITGB1 | AC010542.5 | -0.308430227 | 9.44E-05 |
| KIF5B | AC010542.5 | -0.314865562 | 6.60E-05 |
| MAP2K7 | AC010542.5 | 0.369720326 | 2.20E-06 |
| MAPK1 | AC010542.5 | -0.318653077 | 5.32E-05 |
| MTMR14 | AC010542.5 | 0.494578165 | 6.12E-11 |
| NCKAP1 | AC010542.5 | -0.376724287 | 1.36E-06 |
| PEX14 | AC010542.5 | 0.354369291 | 6.07E-06 |
| RAB24 | AC010542.5 | 0.42945959 | 2.46E-08 |
| RAF1 | AC010542.5 | 0.308888445 | 9.21E-05 |
| RPTOR | AC010542.5 | -0.343262248 | 1.23E-05 |
| STK11 | AC010542.5 | 0.410209054 | 1.15E-07 |
| ULK3 | AC010542.5 | 0.367032285 | 2.63E-06 |
| WDFY3 | AC010542.5 | -0.33174813 | 2.48E-05 |
| WDR45 | AC010542.5 | 0.560682316 | 3.28E-14 |
| ATG4C | OTUD6B-AS1 | 0.323858183 | 3.94E-05 |
| BAK1 | OTUD6B-AS1 | -0.328697625 | 2.97E-05 |
| CD46 | OTUD6B-AS1 | 0.315801056 | 6.26E-05 |
| DIRAS3 | OTUD6B-AS1 | 0.303543758 | 0.000123289 |
| HDAC1 | OTUD6B-AS1 | 0.330440088 | 2.68E-05 |
| HDAC6 | OTUD6B-AS1 | 0.371238608 | 1.98E-06 |
| MAPK8 | OTUD6B-AS1 | 0.305208087 | 0.000112645 |
| PRKAR1A | OTUD6B-AS1 | 0.395116302 | 3.62E-07 |
| RB1CC1 | OTUD6B-AS1 | 0.412757777 | 9.44E-08 |
| ST13 | OTUD6B-AS1 | 0.37077807 | 2.04E-06 |
| CASP8 | Z98884.2 | 0.363039662 | 3.44E-06 |
| CFLAR | Z98884.2 | 0.324513319 | 3.80E-05 |
| HDAC1 | Z98884.2 | 0.316235081 | 6.11E-05 |
| VAMP3 | Z98884.2 | 0.321635648 | 4.49E-05 |
| ATG10 | DBH-AS1 | 0.320685937 | 4.74E-05 |
| ATG16L2 | DBH-AS1 | 0.310567321 | 8.39E-05 |
| ATG3 | DBH-AS1 | 0.354586919 | 5.99E-06 |
| ATG4B | DBH-AS1 | 0.362657606 | 3.53E-06 |
| ATG4C | DBH-AS1 | 0.361897005 | 3.71E-06 |
| ATG4D | DBH-AS1 | 0.34543963 | 1.07E-05 |
| ATG7 | DBH-AS1 | 0.346678943 | 9.92E-06 |
| BAX | DBH-AS1 | 0.521496476 | 3.46E-12 |
| BCL2 | DBH-AS1 | 0.345894107 | 1.04E-05 |
| BNIP1 | DBH-AS1 | 0.317414161 | 5.71E-05 |
| CAPN10 | DBH-AS1 | 0.438879934 | 1.11E-08 |
| CCR2 | DBH-AS1 | 0.425440927 | 3.42E-08 |
| CDKN1B | DBH-AS1 | 0.370666429 | 2.06E-06 |
| CDKN2A | DBH-AS1 | 0.458169505 | 2.04E-09 |
| CX3CL1 | DBH-AS1 | 0.367502204 | 2.55E-06 |
| CXCR4 | DBH-AS1 | 0.595587932 | 2.98E-16 |
| DAPK2 | DBH-AS1 | 0.318212171 | 5.46E-05 |
| GABARAP | DBH-AS1 | 0.323020907 | 4.14E-05 |
| HDAC6 | DBH-AS1 | 0.370614063 | 2.07E-06 |
| HIF1A | DBH-AS1 | -0.319273418 | 5.14E-05 |
| IFNG | DBH-AS1 | 0.622480247 | 5.31E-18 |
| IKBKB | DBH-AS1 | 0.360788259 | 3.99E-06 |
| IKBKE | DBH-AS1 | 0.327975762 | 3.10E-05 |
| ITGA6 | DBH-AS1 | -0.311430834 | 8.00E-05 |
| MAP2K7 | DBH-AS1 | 0.431306573 | 2.11E-08 |
| MTMR14 | DBH-AS1 | 0.520364178 | 3.93E-12 |
| NCKAP1 | DBH-AS1 | -0.386542202 | 6.77E-07 |
| NKX2-3 | DBH-AS1 | 0.361991546 | 3.69E-06 |
| PEX14 | DBH-AS1 | 0.306735884 | 0.000103635 |
| PTK6 | DBH-AS1 | -0.300413763 | 0.000145894 |
| RAB1A | DBH-AS1 | -0.346085284 | 1.03E-05 |
| RAB24 | DBH-AS1 | 0.466143639 | 9.80E-10 |
| RAF1 | DBH-AS1 | 0.377712079 | 1.27E-06 |
| RGS19 | DBH-AS1 | 0.325667475 | 3.55E-05 |
| STK11 | DBH-AS1 | 0.455744332 | 2.54E-09 |
| TP53 | DBH-AS1 | 0.45308 | 3.23E-09 |
| TP73 | DBH-AS1 | 0.302272973 | 0.000132041 |
| TSC2 | DBH-AS1 | 0.365996482 | 2.82E-06 |
| ULK3 | DBH-AS1 | 0.372060033 | 1.87E-06 |
| WDFY3 | DBH-AS1 | -0.341069219 | 1.41E-05 |
| WDR45 | DBH-AS1 | 0.380785934 | 1.02E-06 |
| ARNT | AC121757.1 | 0.410334276 | 1.14E-07 |
| BNIP3 | AC121757.1 | -0.327759299 | 3.14E-05 |
| CD46 | AC121757.1 | 0.420917669 | 4.94E-08 |
| CDKN1B | AC121757.1 | 0.303112128 | 0.000126199 |
| EEF2K | AC121757.1 | 0.464874466 | 1.10E-09 |
| EIF2AK3 | AC121757.1 | 0.378712917 | 1.18E-06 |
| HDAC1 | AC121757.1 | 0.328221991 | 3.05E-05 |
| HDAC6 | AC121757.1 | 0.343159661 | 1.24E-05 |
| IKBKE | AC121757.1 | 0.343721813 | 1.19E-05 |
| KLHL24 | AC121757.1 | 0.335007611 | 2.04E-05 |
| MAPK8 | AC121757.1 | 0.395142667 | 3.61E-07 |
| NBR1 | AC121757.1 | 0.353282898 | 6.51E-06 |
| PARP1 | AC121757.1 | 0.346857728 | 9.80E-06 |
| PIK3R4 | AC121757.1 | 0.348510921 | 8.83E-06 |
| PRKAB1 | AC121757.1 | 0.332298 | 2.40E-05 |
| SH3GLB1 | AC121757.1 | 0.357359507 | 5.00E-06 |
| SIRT1 | AC121757.1 | 0.305870866 | 0.00010865 |
| TP53 | AC121757.1 | 0.35297056 | 6.65E-06 |
| TP73 | AC121757.1 | 0.314510853 | 6.73E-05 |
| TSC1 | AC121757.1 | 0.388103692 | 6.05E-07 |
| TSC2 | AC121757.1 | 0.332293754 | 2.40E-05 |
| ARSB | SH3BP5-AS1 | 0.317936383 | 5.55E-05 |
| ATG10 | SH3BP5-AS1 | 0.385528093 | 7.28E-07 |
| ATG12 | SH3BP5-AS1 | 0.382231141 | 9.21E-07 |
| ATG16L2 | SH3BP5-AS1 | 0.390008859 | 5.27E-07 |
| ATG4B | SH3BP5-AS1 | 0.348151013 | 9.04E-06 |
| ATG4C | SH3BP5-AS1 | 0.489035526 | 1.07E-10 |
| ATG7 | SH3BP5-AS1 | 0.352008802 | 7.07E-06 |
| BAG3 | SH3BP5-AS1 | -0.315849633 | 6.24E-05 |
| BCL2 | SH3BP5-AS1 | 0.382856583 | 8.81E-07 |
| CAPN10 | SH3BP5-AS1 | 0.49105988 | 8.74E-11 |
| CCR2 | SH3BP5-AS1 | 0.34089592 | 1.42E-05 |
| CD46 | SH3BP5-AS1 | 0.318841997 | 5.27E-05 |
| CDKN1B | SH3BP5-AS1 | 0.389281449 | 5.55E-07 |
| CXCR4 | SH3BP5-AS1 | 0.639566442 | 3.33E-19 |
| DAPK2 | SH3BP5-AS1 | 0.335613395 | 1.96E-05 |
| DRAM1 | SH3BP5-AS1 | 0.335383489 | 1.99E-05 |
| FAS | SH3BP5-AS1 | 0.315096977 | 6.51E-05 |
| HDAC6 | SH3BP5-AS1 | 0.492037425 | 7.92E-11 |
| HSPA5 | SH3BP5-AS1 | -0.311343628 | 8.04E-05 |
| IKBKB | SH3BP5-AS1 | 0.450871941 | 3.93E-09 |
| IKBKE | SH3BP5-AS1 | 0.335629842 | 1.96E-05 |
| MAP2K7 | SH3BP5-AS1 | 0.474948985 | 4.27E-10 |
| MAPK8 | SH3BP5-AS1 | 0.388029493 | 6.08E-07 |
| MTMR14 | SH3BP5-AS1 | 0.47794868 | 3.20E-10 |
| NKX2-3 | SH3BP5-AS1 | 0.398930908 | 2.73E-07 |
| PARP1 | SH3BP5-AS1 | 0.300012233 | 0.000149059 |
| RAB24 | SH3BP5-AS1 | 0.305167622 | 0.000112893 |
| RAF1 | SH3BP5-AS1 | 0.529094045 | 1.47E-12 |
| SIRT1 | SH3BP5-AS1 | 0.306353595 | 0.000105824 |
| STK11 | SH3BP5-AS1 | 0.399417642 | 2.63E-07 |
| TP53 | SH3BP5-AS1 | 0.382534221 | 9.01E-07 |
| TP73 | SH3BP5-AS1 | 0.408564542 | 1.31E-07 |
| TSC1 | SH3BP5-AS1 | 0.413852793 | 8.67E-08 |
| TSC2 | SH3BP5-AS1 | 0.529218545 | 1.45E-12 |
| ULK2 | SH3BP5-AS1 | 0.305776549 | 0.00010921 |
| ULK3 | SH3BP5-AS1 | 0.454210496 | 2.92E-09 |
| UVRAG | SH3BP5-AS1 | 0.310390446 | 8.47E-05 |
| WDR45 | SH3BP5-AS1 | 0.382197385 | 9.23E-07 |
| ATG12 | AC010326.3 | 0.334182035 | 2.14E-05 |
| ATG16L2 | AC010326.3 | 0.440958022 | 9.33E-09 |
| ATG3 | AC010326.3 | 0.300730317 | 0.000143444 |
| ATG4B | AC010326.3 | 0.412070695 | 9.97E-08 |
| ATG4C | AC010326.3 | 0.317539326 | 5.67E-05 |
| CAPN10 | AC010326.3 | 0.446051336 | 6.00E-09 |
| CASP8 | AC010326.3 | 0.36788259 | 2.49E-06 |
| CFLAR | AC010326.3 | 0.424753253 | 3.62E-08 |
| CXCR4 | AC010326.3 | 0.37621963 | 1.40E-06 |
| DAPK2 | AC010326.3 | 0.364659347 | 3.09E-06 |
| HDAC6 | AC010326.3 | 0.385259565 | 7.42E-07 |
| IKBKB | AC010326.3 | 0.387547599 | 6.29E-07 |
| MAP2K7 | AC010326.3 | 0.382218166 | 9.21E-07 |
| PEX14 | AC010326.3 | 0.375479816 | 1.48E-06 |
| RAB24 | AC010326.3 | 0.325642879 | 3.55E-05 |
| STK11 | AC010326.3 | 0.39334963 | 4.12E-07 |
| TSC2 | AC010326.3 | 0.308548746 | 9.38E-05 |
| ULK3 | AC010326.3 | 0.313228612 | 7.23E-05 |
| WDR45 | AC010326.3 | 0.40873076 | 1.29E-07 |
| ARSB | AL049555.1 | -0.30722414 | 0.000100901 |
| BCL2 | AL049555.1 | -0.319304983 | 5.13E-05 |
| FAS | AL049555.1 | -0.335537549 | 1.97E-05 |
| MAPK9 | AL049555.1 | -0.320479816 | 4.79E-05 |
| VEGFA | LINC01629 | 0.394429147 | 3.81E-07 |
| ATG16L2 | AL606834.2 | 0.326364603 | 3.41E-05 |
| CAPN10 | AL606834.2 | 0.382227299 | 9.21E-07 |
| CXCR4 | AL606834.2 | 0.352252314 | 6.96E-06 |
| ULK3 | AL606834.2 | 0.375287462 | 1.50E-06 |
| ATG4B | AC040977.1 | 0.509852376 | 1.24E-11 |
| BAX | AC040977.1 | 0.459465267 | 1.81E-09 |
| BIRC6 | AC040977.1 | -0.311905677 | 7.79E-05 |
| BNIP1 | AC040977.1 | 0.369476658 | 2.23E-06 |
| CANX | AC040977.1 | -0.350964376 | 7.56E-06 |
| CAPN10 | AC040977.1 | 0.430797633 | 2.20E-08 |
| CLN3 | AC040977.1 | 0.318501726 | 5.37E-05 |
| EIF4G1 | AC040977.1 | -0.330648648 | 2.65E-05 |
| GABARAP | AC040977.1 | 0.451086746 | 3.85E-09 |
| KIF5B | AC040977.1 | -0.359302115 | 4.40E-06 |
| MBTPS2 | AC040977.1 | -0.323333022 | 4.07E-05 |
| NCKAP1 | AC040977.1 | -0.459267333 | 1.85E-09 |
| PEX14 | AC040977.1 | 0.357799509 | 4.86E-06 |
| RAB24 | AC040977.1 | 0.441724952 | 8.73E-09 |
| STK11 | AC040977.1 | 0.368753406 | 2.34E-06 |
| TP63 | AC040977.1 | -0.302571257 | 0.000129936 |
| USP10 | AC040977.1 | -0.369744919 | 2.19E-06 |
| WDFY3 | AC040977.1 | -0.369831095 | 2.18E-06 |
| WDR45 | AC040977.1 | 0.391850909 | 4.60E-07 |
| ATF6 | AC106798.1 | 0.352981885 | 6.64E-06 |
| BIRC6 | AC106798.1 | 0.375701989 | 1.46E-06 |
| EIF4G1 | AC106798.1 | 0.426854619 | 3.05E-08 |
| FOXO3 | AC106798.1 | 0.344557922 | 1.13E-05 |
| GABARAPL1 | AC106798.1 | 0.362342534 | 3.60E-06 |
| KLHL24 | AC106798.1 | 0.405771636 | 1.62E-07 |
| MBTPS2 | AC106798.1 | 0.351424646 | 7.34E-06 |
| NBR1 | AC106798.1 | 0.343742415 | 1.19E-05 |
| NCKAP1 | AC106798.1 | 0.47677854 | 3.58E-10 |
| NFE2L2 | AC106798.1 | 0.314235826 | 6.84E-05 |
| PEX14 | AC106798.1 | -0.357971164 | 4.81E-06 |
| RAB24 | AC106798.1 | -0.300135648 | 0.00014808 |
| RB1CC1 | AC106798.1 | 0.310840369 | 8.27E-05 |
| ST13 | AC106798.1 | 0.389552067 | 5.44E-07 |
| STK11 | AC106798.1 | -0.308416093 | 9.45E-05 |
| TP63 | AC106798.1 | 0.372441186 | 1.82E-06 |
| USP10 | AC106798.1 | 0.320203062 | 4.87E-05 |
| HDAC6 | AC008759.3 | 0.342278683 | 1.31E-05 |
| ARSB | AC023906.2 | -0.356067193 | 5.44E-06 |
| ATG2A | AC023906.2 | -0.344919384 | 1.11E-05 |
| ATG4C | AC023906.2 | -0.311538147 | 7.95E-05 |
| CD46 | AC023906.2 | -0.314724431 | 6.65E-05 |
| FAS | AC023906.2 | -0.300526148 | 0.00014502 |
| HDAC6 | AC023906.2 | -0.331062724 | 2.58E-05 |
| HSPA5 | AC023906.2 | 0.438163399 | 1.18E-08 |
| NBR1 | AC023906.2 | -0.308143067 | 9.59E-05 |
| PARP1 | AC023906.2 | -0.314279127 | 6.82E-05 |
| SH3GLB1 | AC023906.2 | -0.326258491 | 3.43E-05 |
| SIRT1 | AC023906.2 | -0.315425767 | 6.39E-05 |
| ERBB2 | AP003119.2 | 0.687313501 | 5.31E-23 |
| ATG10 | AF131215.6 | 0.338215834 | 1.68E-05 |
| ATG3 | AF131215.6 | 0.348694558 | 8.73E-06 |
| ATG4B | AF131215.6 | 0.474175769 | 4.59E-10 |
| ATG4C | AF131215.6 | 0.324436835 | 3.81E-05 |
| ATG4D | AF131215.6 | 0.30669155 | 0.000103887 |
| ATG7 | AF131215.6 | 0.402704438 | 2.05E-07 |
| BAG3 | AF131215.6 | -0.307778069 | 9.79E-05 |
| BAX | AF131215.6 | 0.450959271 | 3.90E-09 |
| BCL2 | AF131215.6 | 0.418163404 | 6.16E-08 |
| CAPN10 | AF131215.6 | 0.474495287 | 4.45E-10 |
| CDKN1B | AF131215.6 | 0.346505057 | 1.00E-05 |
| CDKN2A | AF131215.6 | 0.473138557 | 5.07E-10 |
| CX3CL1 | AF131215.6 | 0.348902003 | 8.62E-06 |
| CXCR4 | AF131215.6 | 0.671691956 | 1.11E-21 |
| HDAC6 | AF131215.6 | 0.368060173 | 2.46E-06 |
| HIF1A | AF131215.6 | -0.352641346 | 6.79E-06 |
| HSPA5 | AF131215.6 | -0.360060717 | 4.19E-06 |
| IFNG | AF131215.6 | 0.324379764 | 3.83E-05 |
| IKBKB | AF131215.6 | 0.364431427 | 3.14E-06 |
| IKBKE | AF131215.6 | 0.33530629 | 2.00E-05 |
| MAP2K7 | AF131215.6 | 0.49699343 | 4.78E-11 |
| MTMR14 | AF131215.6 | 0.57436475 | 5.55E-15 |
| NCKAP1 | AF131215.6 | -0.3035322 | 0.000123366 |
| NKX2-3 | AF131215.6 | 0.36416678 | 3.19E-06 |
| PEX14 | AF131215.6 | 0.30429088 | 0.0001184 |
| PINK1 | AF131215.6 | -0.300352273 | 0.000146375 |
| PRKAB1 | AF131215.6 | 0.305792516 | 0.000109115 |
| RAB24 | AF131215.6 | 0.433432176 | 1.77E-08 |
| RAF1 | AF131215.6 | 0.486000884 | 1.45E-10 |
| STK11 | AF131215.6 | 0.505415043 | 1.98E-11 |
| TP53 | AF131215.6 | 0.472866434 | 5.20E-10 |
| TP73 | AF131215.6 | 0.381550851 | 9.66E-07 |
| TSC2 | AF131215.6 | 0.540986103 | 3.68E-13 |
| ULK3 | AF131215.6 | 0.369814712 | 2.18E-06 |
| WDR45 | AF131215.6 | 0.405536161 | 1.65E-07 |
| ATF4 | AL035071.1 | 0.345968536 | 1.04E-05 |
| ATG16L2 | AL035071.1 | 0.391157678 | 4.84E-07 |
| ATG4B | AL035071.1 | 0.391453406 | 4.74E-07 |
| ATG4D | AL035071.1 | 0.365548972 | 2.91E-06 |
| BAX | AL035071.1 | 0.379584496 | 1.11E-06 |
| BIRC6 | AL035071.1 | -0.348356364 | 8.92E-06 |
| BNIP1 | AL035071.1 | 0.343625784 | 1.20E-05 |
| CAPN10 | AL035071.1 | 0.518300813 | 4.93E-12 |
| CHMP4B | AL035071.1 | 0.643672091 | 1.67E-19 |
| DAPK2 | AL035071.1 | 0.406275429 | 1.56E-07 |
| EIF4G1 | AL035071.1 | -0.363768817 | 3.28E-06 |
| GABARAP | AL035071.1 | 0.399027628 | 2.71E-07 |
| MAP1LC3A | AL035071.1 | 0.491492555 | 8.37E-11 |
| MBTPS2 | AL035071.1 | -0.323171563 | 4.10E-05 |
| MTMR14 | AL035071.1 | 0.316396408 | 6.05E-05 |
| NCKAP1 | AL035071.1 | -0.304447964 | 0.000117395 |
| NKX2-3 | AL035071.1 | 0.309679345 | 8.81E-05 |
| PELP1 | AL035071.1 | 0.363106347 | 3.43E-06 |
| PEX14 | AL035071.1 | 0.352663425 | 6.78E-06 |
| RAB24 | AL035071.1 | 0.35539865 | 5.68E-06 |
| STK11 | AL035071.1 | 0.447466537 | 5.30E-09 |
| ULK3 | AL035071.1 | 0.313143819 | 7.27E-05 |
| USP10 | AL035071.1 | -0.328947833 | 2.93E-05 |
| WDFY3 | AL035071.1 | -0.330523984 | 2.67E-05 |
| WDR45 | AL035071.1 | 0.342332509 | 1.30E-05 |
| ATIC | AC099568.2 | 0.360582908 | 4.05E-06 |
| BAK1 | AC099568.2 | -0.320042204 | 4.92E-05 |
| BIRC5 | AC099568.2 | 0.342475222 | 1.29E-05 |
| CAPN10 | AC099568.2 | 0.370219651 | 2.12E-06 |
| ITGA3 | AC099568.2 | -0.367398793 | 2.57E-06 |
| MAPK8IP1 | AC099568.2 | 0.508632237 | 1.41E-11 |
| PINK1 | AC099568.2 | -0.367443004 | 2.56E-06 |
| RHEB | AC099568.2 | 0.321661427 | 4.48E-05 |
| ULK3 | AC099568.2 | 0.325314169 | 3.62E-05 |
| BCL2 | AC005586.1 | 0.353325957 | 6.50E-06 |
| BIRC6 | AC005586.1 | 0.312913417 | 7.36E-05 |
| DRAM1 | AC005586.1 | 0.35104774 | 7.52E-06 |
| EIF2AK3 | AC005586.1 | 0.456714201 | 2.33E-09 |
| FOXO3 | AC005586.1 | 0.367265777 | 2.59E-06 |
| ITGA3 | AC005586.1 | -0.378638766 | 1.19E-06 |
| ITPR1 | AC005586.1 | 0.370835303 | 2.03E-06 |
| KLHL24 | AC005586.1 | 0.356079951 | 5.44E-06 |
| NBR1 | AC005586.1 | 0.374892112 | 1.54E-06 |
| NFE2L2 | AC005586.1 | 0.517086034 | 5.64E-12 |
| PARP1 | AC005586.1 | 0.33949598 | 1.55E-05 |
| PINK1 | AC005586.1 | -0.307972217 | 9.68E-05 |
| PRKCD | AC005586.1 | 0.300594308 | 0.000144492 |
| RHEB | AC005586.1 | 0.40380198 | 1.89E-07 |
| RPS6KB1 | AC005586.1 | 0.338848578 | 1.61E-05 |
| VEGFA | AC092535.4 | 0.331072009 | 2.58E-05 |
| CAMKK2 | AC073365.1 | 0.309035388 | 9.13E-05 |
| FKBP1B | AC073365.1 | 0.38635842 | 6.86E-07 |
| FOXO1 | AC073365.1 | -0.322127214 | 4.36E-05 |
| ITGB1 | AC073365.1 | 0.386087716 | 6.99E-07 |
| ATIC | AL109615.3 | 0.397038259 | 3.14E-07 |
| BAG3 | AL109615.3 | -0.315709062 | 6.29E-05 |
| CAPN10 | AL109615.3 | 0.396849577 | 3.18E-07 |
| DIRAS3 | AL109615.3 | 0.729149757 | 5.52E-27 |
| FKBP1B | AL109615.3 | 0.43980781 | 1.03E-08 |
| GABARAPL2 | AL109615.3 | 0.370241034 | 2.12E-06 |
| MAP1LC3A | AL109615.3 | 0.375524629 | 1.47E-06 |
| MAPK8IP1 | AL109615.3 | 0.391971586 | 4.56E-07 |
| PRKCQ | AL109615.3 | 0.330796756 | 2.62E-05 |
| ATG4B | RTCA-AS1 | 0.354543964 | 6.00E-06 |
| BAX | RTCA-AS1 | 0.394886983 | 3.68E-07 |
| BNIP1 | RTCA-AS1 | 0.456564925 | 2.36E-09 |
| CAPN10 | RTCA-AS1 | 0.319315379 | 5.13E-05 |
| CDKN2A | RTCA-AS1 | 0.325242333 | 3.64E-05 |
| DDIT3 | RTCA-AS1 | 0.402562219 | 2.07E-07 |
| GABARAP | RTCA-AS1 | 0.31983902 | 4.97E-05 |
| HDAC6 | RTCA-AS1 | 0.301847637 | 0.000135098 |
| HGS | RTCA-AS1 | -0.33351243 | 2.23E-05 |
| IKBKE | RTCA-AS1 | 0.326229222 | 3.43E-05 |
| ITGA3 | RTCA-AS1 | -0.330401996 | 2.68E-05 |
| ITGA6 | RTCA-AS1 | -0.314773774 | 6.63E-05 |
| PRKAB1 | RTCA-AS1 | 0.337054981 | 1.80E-05 |
| WDR45 | RTCA-AS1 | 0.409483139 | 1.22E-07 |
| ATF4 | AL118516.1 | 0.328849692 | 2.94E-05 |
| ATG2A | AL118516.1 | -0.312530992 | 7.52E-05 |
| GABARAPL2 | AL118516.1 | 0.357173343 | 5.06E-06 |
| NFKB1 | AL118516.1 | -0.325710555 | 3.54E-05 |
| WDR45 | AL118516.1 | 0.304372107 | 0.000117879 |
| AMBRA1 | ARHGAP5-AS1 | -0.315709127 | 6.29E-05 |
| CALCOCO2 | ARHGAP5-AS1 | -0.330554973 | 2.66E-05 |
| DRAM1 | ARHGAP5-AS1 | -0.326400525 | 3.40E-05 |
| FAS | ARHGAP5-AS1 | -0.31510468 | 6.51E-05 |
| MAPK8 | ARHGAP5-AS1 | -0.302322618 | 0.000131689 |
| SQSTM1 | ARHGAP5-AS1 | -0.315536029 | 6.35E-05 |
| ARNT | AL158152.2 | -0.429617713 | 2.43E-08 |
| ARSB | AL158152.2 | -0.317520674 | 5.68E-05 |
| ATF4 | AL158152.2 | 0.348850362 | 8.64E-06 |
| ATF6 | AL158152.2 | -0.313184403 | 7.25E-05 |
| BIRC6 | AL158152.2 | -0.518042983 | 5.08E-12 |
| CANX | AL158152.2 | -0.337599676 | 1.74E-05 |
| EDEM1 | AL158152.2 | -0.355079283 | 5.80E-06 |
| EEF2K | AL158152.2 | -0.302133974 | 0.000133033 |
| EIF2AK3 | AL158152.2 | -0.383739017 | 8.27E-07 |
| EIF4G1 | AL158152.2 | -0.401273889 | 2.28E-07 |
| FOXO3 | AL158152.2 | -0.353201007 | 6.55E-06 |
| KIF5B | AL158152.2 | -0.380372777 | 1.05E-06 |
| KLHL24 | AL158152.2 | -0.349223166 | 8.44E-06 |
| LAMP2 | AL158152.2 | -0.31704315 | 5.83E-05 |
| MAPK1 | AL158152.2 | -0.481073635 | 2.36E-10 |
| MBTPS2 | AL158152.2 | -0.470157587 | 6.73E-10 |
| MTOR | AL158152.2 | -0.340357386 | 1.47E-05 |
| NAF1 | AL158152.2 | -0.317552186 | 5.67E-05 |
| NBR1 | AL158152.2 | -0.359727364 | 4.28E-06 |
| NCKAP1 | AL158152.2 | -0.395225188 | 3.59E-07 |
| PARP1 | AL158152.2 | -0.318156406 | 5.48E-05 |
| PIK3R4 | AL158152.2 | -0.384610847 | 7.77E-07 |
| RAB24 | AL158152.2 | 0.303674465 | 0.000122421 |
| RB1CC1 | AL158152.2 | -0.317012044 | 5.85E-05 |
| SIRT1 | AL158152.2 | -0.36495646 | 3.03E-06 |
| USP10 | AL158152.2 | -0.344702595 | 1.12E-05 |
| VAMP7 | AL158152.2 | -0.308620569 | 9.34E-05 |
| WDFY3 | AL158152.2 | -0.435245774 | 1.52E-08 |
| IKBKB | AC018653.3 | 0.303587234 | 0.000123 |
| SPNS1 | AC018653.3 | 0.400578866 | 2.41E-07 |
| TSC1 | AC018653.3 | 0.306690772 | 0.000103891 |
| ARSB | AL365356.4 | -0.310660758 | 8.35E-05 |
| BCL2 | AL365356.4 | -0.335633139 | 1.96E-05 |
| BNIP3 | AL365356.4 | 0.318045434 | 5.51E-05 |
| EEF2 | AL365356.4 | -0.308833915 | 9.24E-05 |
| EEF2K | AL365356.4 | -0.316683099 | 5.96E-05 |
| EIF2AK3 | AL365356.4 | -0.328950968 | 2.93E-05 |
| HDAC6 | AL365356.4 | -0.32086681 | 4.69E-05 |
| KLHL24 | AL365356.4 | -0.338685685 | 1.63E-05 |
| MAPK9 | AL365356.4 | -0.355130529 | 5.78E-06 |
| NBR1 | AL365356.4 | -0.342133531 | 1.32E-05 |
| PARP1 | AL365356.4 | -0.388797723 | 5.75E-07 |
| PIK3R4 | AL365356.4 | -0.311413777 | 8.01E-05 |
| PTK6 | AL365356.4 | 0.395286602 | 3.57E-07 |
| TP53 | AL365356.4 | -0.304262524 | 0.000118582 |
| UVRAG | AL365356.4 | -0.311081324 | 8.16E-05 |
| VAMP7 | AL365356.4 | -0.316013985 | 6.19E-05 |
| ATG4D | LINC00324 | 0.329301054 | 2.87E-05 |
| CCR2 | LINC00324 | 0.40310284 | 1.99E-07 |
| CLN3 | LINC00324 | 0.362711969 | 3.52E-06 |
| GABARAP | LINC00324 | 0.438332156 | 1.17E-08 |
| HDAC6 | LINC00324 | 0.328051351 | 3.09E-05 |
| HSPA5 | LINC00324 | -0.335433164 | 1.98E-05 |
| IFNG | LINC00324 | 0.360208775 | 4.15E-06 |
| IKBKB | LINC00324 | 0.316865431 | 5.89E-05 |
| IKBKE | LINC00324 | 0.439144717 | 1.09E-08 |
| KLHL24 | LINC00324 | 0.317473018 | 5.69E-05 |
| MTMR14 | LINC00324 | 0.325892505 | 3.50E-05 |
| NBR1 | LINC00324 | 0.302785294 | 0.000128445 |
| NKX2-3 | LINC00324 | 0.373804839 | 1.66E-06 |
| PARP1 | LINC00324 | 0.405665822 | 1.64E-07 |
| TSC2 | LINC00324 | 0.342386551 | 1.30E-05 |
| WDR45 | LINC00324 | 0.419900426 | 5.36E-08 |
| ATF4 | AC012615.1 | 0.361821201 | 3.73E-06 |
| ATG16L2 | AC012615.1 | 0.540441017 | 3.92E-13 |
| ATG4B | AC012615.1 | 0.465003428 | 1.09E-09 |
| ATG4C | AC012615.1 | 0.319578545 | 5.05E-05 |
| ATG4D | AC012615.1 | 0.383542163 | 8.39E-07 |
| ATIC | AC012615.1 | 0.320593988 | 4.76E-05 |
| BAX | AC012615.1 | 0.366187878 | 2.79E-06 |
| BID | AC012615.1 | 0.337816368 | 1.72E-05 |
| CAPN10 | AC012615.1 | 0.645567399 | 1.21E-19 |
| CDKN1B | AC012615.1 | 0.342702983 | 1.27E-05 |
| CLN3 | AC012615.1 | 0.323166037 | 4.11E-05 |
| CXCR4 | AC012615.1 | 0.361655303 | 3.77E-06 |
| EIF4G1 | AC012615.1 | -0.323082329 | 4.13E-05 |
| GABARAP | AC012615.1 | 0.353160019 | 6.57E-06 |
| HDAC6 | AC012615.1 | 0.407459604 | 1.43E-07 |
| IKBKB | AC012615.1 | 0.358634139 | 4.60E-06 |
| ITGA3 | AC012615.1 | -0.426173787 | 3.22E-08 |
| ITGB4 | AC012615.1 | -0.30536281 | 0.0001117 |
| MAP1LC3A | AC012615.1 | 0.390241683 | 5.18E-07 |
| MAP2K7 | AC012615.1 | 0.466872511 | 9.16E-10 |
| MLST8 | AC012615.1 | 0.394196226 | 3.87E-07 |
| MTMR14 | AC012615.1 | 0.330080759 | 2.74E-05 |
| PELP1 | AC012615.1 | 0.390361499 | 5.13E-07 |
| PINK1 | AC012615.1 | -0.398471687 | 2.82E-07 |
| PRKAB1 | AC012615.1 | 0.423282975 | 4.08E-08 |
| STK11 | AC012615.1 | 0.49424188 | 6.33E-11 |
| TSC2 | AC012615.1 | 0.325918992 | 3.50E-05 |
| ULK3 | AC012615.1 | 0.458760264 | 1.93E-09 |
| WDR45 | AC012615.1 | 0.422208496 | 4.45E-08 |
| ZFYVE1 | AC012615.1 | -0.320597712 | 4.76E-05 |
| ATG4A | AL354920.1 | 0.346548641 | 1.00E-05 |
| CDKN2A | AL354920.1 | 0.306654487 | 0.000104098 |
| EIF4G1 | AC108676.1 | 0.315539834 | 6.35E-05 |
| TNFSF10 | AC108676.1 | 0.379769362 | 1.10E-06 |
| DRAM1 | LINC01127 | 0.332830337 | 2.32E-05 |
| FAS | LINC01127 | 0.304001605 | 0.000120271 |
| NKX2-3 | LINC01127 | 0.412978622 | 9.28E-08 |
| ATF4 | LINC02001 | 0.306187573 | 0.000106788 |
| BAX | LINC02001 | 0.394838584 | 3.70E-07 |
| BIRC5 | LINC02001 | 0.371699507 | 1.92E-06 |
| CAPN2 | LINC02001 | -0.330295851 | 2.70E-05 |
| GABARAPL1 | LINC02001 | 0.309185139 | 9.06E-05 |
| GNAI3 | LINC02001 | -0.339662629 | 1.53E-05 |
| ITGA3 | LINC02001 | -0.401066483 | 2.32E-07 |
| ITGB4 | LINC02001 | -0.339290637 | 1.57E-05 |
| MAP2K7 | LINC02001 | 0.326167348 | 3.45E-05 |
| PELP1 | LINC02001 | 0.344915977 | 1.11E-05 |
| PINK1 | LINC02001 | -0.403347186 | 1.95E-07 |
| WDR45 | LINC02001 | 0.371779832 | 1.91E-06 |
| ZFYVE1 | LINC02001 | -0.316051951 | 6.17E-05 |
| ATG4B | MIR3142HG | 0.313524337 | 7.12E-05 |
| ATG4C | MIR3142HG | 0.354586273 | 5.99E-06 |
| BCL2 | MIR3142HG | 0.303772938 | 0.00012177 |
| CAPN10 | MIR3142HG | 0.369142684 | 2.28E-06 |
| CX3CL1 | MIR3142HG | 0.359219619 | 4.43E-06 |
| CXCR4 | MIR3142HG | 0.595764349 | 2.91E-16 |
| DAPK2 | MIR3142HG | 0.315478412 | 6.38E-05 |
| IKBKB | MIR3142HG | 0.332213501 | 2.41E-05 |
| IKBKE | MIR3142HG | 0.355448099 | 5.66E-06 |
| LAMP1 | MIR3142HG | -0.304955811 | 0.000114201 |
| MTMR14 | MIR3142HG | 0.313496164 | 7.13E-05 |
| RAB24 | MIR3142HG | 0.336028692 | 1.91E-05 |
| RAF1 | MIR3142HG | 0.3326378 | 2.35E-05 |
| TP53 | MIR3142HG | 0.438025289 | 1.20E-08 |
| TP73 | MIR3142HG | 0.388791232 | 5.75E-07 |
| TSC2 | MIR3142HG | 0.595398631 | 3.06E-16 |
| ULK3 | MIR3142HG | 0.408958094 | 1.27E-07 |
| UVRAG | MIR3142HG | 0.303570037 | 0.000123114 |
| ATG16L2 | AF064858.3 | 0.363176855 | 3.41E-06 |
| IL24 | AF064858.3 | 0.529381702 | 1.42E-12 |
| ATF4 | SNHG6 | 0.329335302 | 2.86E-05 |
| ATG4B | SNHG6 | 0.366269915 | 2.77E-06 |
| BAG1 | SNHG6 | 0.337841021 | 1.71E-05 |
| BAX | SNHG6 | 0.587682205 | 9.08E-16 |
| BIRC5 | SNHG6 | 0.539684357 | 4.29E-13 |
| BNIP1 | SNHG6 | 0.449780114 | 4.33E-09 |
| CAPN10 | SNHG6 | 0.405405463 | 1.67E-07 |
| CAPN2 | SNHG6 | -0.351540739 | 7.28E-06 |
| CXCR4 | SNHG6 | 0.35412964 | 6.17E-06 |
| EIF4EBP1 | SNHG6 | 0.300076987 | 0.000148544 |
| EIF4G1 | SNHG6 | -0.341319182 | 1.38E-05 |
| FKBP1A | SNHG6 | 0.454064287 | 2.95E-09 |
| MAPK1 | SNHG6 | -0.382666036 | 8.93E-07 |
| MLST8 | SNHG6 | 0.331063545 | 2.58E-05 |
| MTMR14 | SNHG6 | 0.35471155 | 5.94E-06 |
| NCKAP1 | SNHG6 | -0.31675977 | 5.93E-05 |
| WDFY3 | SNHG6 | -0.30021406 | 0.00014746 |
| WDR45 | SNHG6 | 0.406309539 | 1.56E-07 |
| ATF4 | ARRDC1-AS1 | 0.344364784 | 1.15E-05 |
| ATG16L2 | ARRDC1-AS1 | 0.325198481 | 3.65E-05 |
| ATG2B | ARRDC1-AS1 | -0.338419874 | 1.66E-05 |
| ATG3 | ARRDC1-AS1 | 0.473911813 | 4.71E-10 |
| ATG4B | ARRDC1-AS1 | 0.482899037 | 1.97E-10 |
| ATG4D | ARRDC1-AS1 | 0.317450371 | 5.70E-05 |
| BAG1 | ARRDC1-AS1 | 0.361514194 | 3.81E-06 |
| BAX | ARRDC1-AS1 | 0.568117052 | 1.26E-14 |
| BIRC5 | ARRDC1-AS1 | 0.449117658 | 4.59E-09 |
| BIRC6 | ARRDC1-AS1 | -0.311974185 | 7.76E-05 |
| BNIP1 | ARRDC1-AS1 | 0.459344975 | 1.83E-09 |
| CAPN10 | ARRDC1-AS1 | 0.508981583 | 1.36E-11 |
| CDKN2A | ARRDC1-AS1 | 0.52844661 | 1.58E-12 |
| CLN3 | ARRDC1-AS1 | 0.323651675 | 3.99E-05 |
| FKBP1A | ARRDC1-AS1 | 0.360842562 | 3.98E-06 |
| KIF5B | ARRDC1-AS1 | -0.32894433 | 2.93E-05 |
| LAMP2 | ARRDC1-AS1 | -0.353118945 | 6.58E-06 |
| MAP1LC3A | ARRDC1-AS1 | 0.34112501 | 1.40E-05 |
| MAP2K7 | ARRDC1-AS1 | 0.345207142 | 1.09E-05 |
| MAPK1 | ARRDC1-AS1 | -0.385785132 | 7.15E-07 |
| MLST8 | ARRDC1-AS1 | 0.387237533 | 6.44E-07 |
| MTMR14 | ARRDC1-AS1 | 0.343589213 | 1.20E-05 |
| NCKAP1 | ARRDC1-AS1 | -0.41413627 | 8.47E-08 |
| PEX14 | ARRDC1-AS1 | 0.382350352 | 9.13E-07 |
| PINK1 | ARRDC1-AS1 | -0.307337911 | 0.000100273 |
| RAB24 | ARRDC1-AS1 | 0.405941804 | 1.60E-07 |
| STK11 | ARRDC1-AS1 | 0.38271406 | 8.90E-07 |
| WDFY3 | ARRDC1-AS1 | -0.37877699 | 1.17E-06 |
| WDR45 | ARRDC1-AS1 | 0.45748712 | 2.17E-09 |
| GABARAPL1 | AL121761.1 | 0.357824863 | 4.85E-06 |
| TP53INP2 | AL121761.1 | 0.362665484 | 3.53E-06 |
| ARNT | LINC01278 | 0.314578239 | 6.71E-05 |
| ARSB | LINC01278 | 0.307144157 | 0.000101344 |
| ATF6 | LINC01278 | 0.318609997 | 5.34E-05 |
| BECN1 | LINC01278 | 0.368645276 | 2.36E-06 |
| BIRC6 | LINC01278 | 0.409624178 | 1.21E-07 |
| CDKN1A | LINC01278 | -0.307720252 | 9.82E-05 |
| HDAC6 | LINC01278 | 0.373463684 | 1.70E-06 |
| IKBKB | LINC01278 | 0.336759621 | 1.83E-05 |
| KLHL24 | LINC01278 | 0.37563285 | 1.46E-06 |
| MBTPS2 | LINC01278 | 0.484432411 | 1.69E-10 |
| NBR1 | LINC01278 | 0.367655137 | 2.52E-06 |
| PIK3R4 | LINC01278 | 0.312164634 | 7.68E-05 |
| RB1CC1 | LINC01278 | 0.313683042 | 7.05E-05 |
| UVRAG | LINC01278 | 0.323105492 | 4.12E-05 |
| VAMP7 | LINC01278 | 0.345620754 | 1.06E-05 |
| ATF4 | AL591895.1 | 0.324281634 | 3.85E-05 |
| ATG16L2 | AL591895.1 | 0.436900774 | 1.32E-08 |
| ATG4B | AL591895.1 | 0.536624261 | 6.15E-13 |
| ATG4C | AL591895.1 | 0.310906879 | 8.23E-05 |
| ATG4D | AL591895.1 | 0.315310702 | 6.44E-05 |
| BAX | AL591895.1 | 0.424496845 | 3.70E-08 |
| CAPN10 | AL591895.1 | 0.615827571 | 1.49E-17 |
| CASP3 | AL591895.1 | 0.354390392 | 6.06E-06 |
| CFLAR | AL591895.1 | 0.334952815 | 2.04E-05 |
| CX3CL1 | AL591895.1 | 0.397686944 | 2.99E-07 |
| CXCR4 | AL591895.1 | 0.572944539 | 6.70E-15 |
| DAPK2 | AL591895.1 | 0.513876403 | 8.01E-12 |
| EIF4G1 | AL591895.1 | -0.321812039 | 4.44E-05 |
| GABARAP | AL591895.1 | 0.341291527 | 1.39E-05 |
| HDAC6 | AL591895.1 | 0.402124457 | 2.14E-07 |
| HIF1A | AL591895.1 | -0.310990228 | 8.20E-05 |
| HSPA5 | AL591895.1 | -0.302674613 | 0.000129214 |
| IKBKB | AL591895.1 | 0.406976955 | 1.48E-07 |
| IKBKE | AL591895.1 | 0.349124086 | 8.50E-06 |
| ITGA3 | AL591895.1 | -0.320353807 | 4.83E-05 |
| ITGA6 | AL591895.1 | -0.322972846 | 4.15E-05 |
| MAP2K7 | AL591895.1 | 0.338988705 | 1.60E-05 |
| MTMR14 | AL591895.1 | 0.325190789 | 3.65E-05 |
| NCKAP1 | AL591895.1 | -0.310876107 | 8.25E-05 |
| NKX2-3 | AL591895.1 | 0.305134925 | 0.000113094 |
| PELP1 | AL591895.1 | 0.328737298 | 2.96E-05 |
| PINK1 | AL591895.1 | -0.402692029 | 2.05E-07 |
| PRKAB1 | AL591895.1 | 0.322350414 | 4.30E-05 |
| RAB24 | AL591895.1 | 0.386999924 | 6.55E-07 |
| STK11 | AL591895.1 | 0.337822168 | 1.72E-05 |
| TP53 | AL591895.1 | 0.328914764 | 2.93E-05 |
| TP73 | AL591895.1 | 0.325543021 | 3.58E-05 |
| TSC2 | AL591895.1 | 0.475065068 | 4.22E-10 |
| ULK3 | AL591895.1 | 0.460745106 | 1.61E-09 |
| USP10 | AL591895.1 | -0.31185599 | 7.81E-05 |
| WDR45 | AL591895.1 | 0.505709291 | 1.92E-11 |
| EEF2 | AC090970.2 | -0.306013899 | 0.000107805 |
| PTK6 | AC090970.2 | 0.313932434 | 6.95E-05 |
| RAB11A | AC090970.2 | 0.323874204 | 3.94E-05 |
| ATG4B | AL118505.1 | 0.3196466 | 5.03E-05 |
| ATG4D | AL118505.1 | 0.302456252 | 0.000130744 |
| CXCR4 | AL118505.1 | 0.363425483 | 3.35E-06 |
| FKBP1A | AL118505.1 | 0.381282508 | 9.85E-07 |
| NKX2-3 | AL118505.1 | 0.312578725 | 7.50E-05 |
| TSC2 | AL118505.1 | 0.36450994 | 3.12E-06 |
| WDR45 | AL118505.1 | 0.346060792 | 1.03E-05 |
| DAPK1 | LINC01206 | 0.3494461 | 8.32E-06 |
| UVRAG | LINC01206 | 0.353707902 | 6.34E-06 |
| CASP1 | NOP14-AS1 | 0.320334767 | 4.84E-05 |
| CASP4 | NOP14-AS1 | 0.50758783 | 1.58E-11 |
| CD46 | NOP14-AS1 | -0.341058299 | 1.41E-05 |
| CDKN1B | NOP14-AS1 | -0.32554463 | 3.58E-05 |
| CX3CL1 | NOP14-AS1 | -0.323873111 | 3.94E-05 |
| KLHL24 | NOP14-AS1 | -0.362434736 | 3.58E-06 |
| MAP2K7 | NOP14-AS1 | -0.34626531 | 1.02E-05 |
| MAPK9 | NOP14-AS1 | -0.314742954 | 6.64E-05 |
| PARP1 | NOP14-AS1 | -0.356473154 | 5.30E-06 |
| PELP1 | NOP14-AS1 | -0.322090684 | 4.37E-05 |
| PIK3R4 | NOP14-AS1 | -0.305018516 | 0.000113812 |
| PTK6 | NOP14-AS1 | 0.446948855 | 5.55E-09 |
| RAC1 | NOP14-AS1 | 0.386929444 | 6.58E-07 |
| RHEB | NOP14-AS1 | -0.366666969 | 2.70E-06 |
| ARSB | MIR205HG | -0.347079876 | 9.67E-06 |
| ATG16L2 | MIR205HG | 0.317904863 | 5.56E-05 |
| ATG4B | MIR205HG | 0.321090385 | 4.63E-05 |
| BIRC6 | MIR205HG | -0.398515436 | 2.81E-07 |
| EIF2AK3 | MIR205HG | -0.335583344 | 1.97E-05 |
| EIF4G1 | MIR205HG | -0.335057708 | 2.03E-05 |
| FOXO1 | MIR205HG | -0.31405502 | 6.91E-05 |
| FOXO3 | MIR205HG | -0.376988718 | 1.33E-06 |
| KIF5B | MIR205HG | -0.335760961 | 1.95E-05 |
| KLHL24 | MIR205HG | -0.308085674 | 9.62E-05 |
| MAPK1 | MIR205HG | -0.324450924 | 3.81E-05 |
| MBTPS2 | MIR205HG | -0.375127214 | 1.51E-06 |
| NBR1 | MIR205HG | -0.31379667 | 7.01E-05 |
| NCKAP1 | MIR205HG | -0.321561141 | 4.51E-05 |
| PEX14 | MIR205HG | 0.431875177 | 2.01E-08 |
| RB1CC1 | MIR205HG | -0.359674555 | 4.30E-06 |
| ST13 | MIR205HG | -0.310931325 | 8.22E-05 |
| WDFY3 | MIR205HG | -0.324356609 | 3.83E-05 |
| ARNT | AL160006.1 | 0.300054353 | 0.000148724 |
| ATG10 | AL160006.1 | 0.324509268 | 3.80E-05 |
| ATG2A | AL160006.1 | 0.417032109 | 6.74E-08 |
| ATG4C | AL160006.1 | 0.435297778 | 1.51E-08 |
| ATG7 | AL160006.1 | 0.325556489 | 3.57E-05 |
| CD46 | AL160006.1 | 0.304641048 | 0.000116171 |
| EEF2K | AL160006.1 | 0.342627171 | 1.28E-05 |
| GNAI3 | AL160006.1 | 0.365251982 | 2.97E-06 |
| HDAC1 | AL160006.1 | 0.339869561 | 1.51E-05 |
| MAP2K7 | AL160006.1 | 0.320944578 | 4.67E-05 |
| MAPK8 | AL160006.1 | 0.423922608 | 3.87E-08 |
| MTOR | AL160006.1 | 0.381656827 | 9.59E-07 |
| NKX2-3 | AL160006.1 | 0.344458211 | 1.14E-05 |
| RAF1 | AL160006.1 | 0.348926454 | 8.60E-06 |
| SH3GLB1 | AL160006.1 | 0.434839332 | 1.57E-08 |
| STK11 | AL160006.1 | 0.323402118 | 4.05E-05 |
| TP53 | AL160006.1 | 0.303037134 | 0.000126711 |
| TP73 | AL160006.1 | 0.436117822 | 1.41E-08 |
| TSC1 | AL160006.1 | 0.420829573 | 4.97E-08 |
| TSC2 | AL160006.1 | 0.423659805 | 3.96E-08 |
| CAPN10 | AL122010.1 | 0.300721937 | 0.000143508 |
| ULK3 | AL122010.1 | 0.355603395 | 5.61E-06 |
| ATG4B | AC124798.1 | 0.302687696 | 0.000129123 |
| ATG4D | AC124798.1 | 0.447699558 | 5.20E-09 |
| ATIC | AC124798.1 | 0.42466438 | 3.65E-08 |
| CAPN10 | AC124798.1 | 0.400831935 | 2.36E-07 |
| CDKN1B | AC124798.1 | 0.326383123 | 3.40E-05 |
| CLN3 | AC124798.1 | 0.369195868 | 2.27E-06 |
| HDAC6 | AC124798.1 | 0.38496284 | 7.58E-07 |
| ITGA3 | AC124798.1 | -0.354977726 | 5.84E-06 |
| MAP2K7 | AC124798.1 | 0.386862721 | 6.61E-07 |
| MAPK8IP1 | AC124798.1 | 0.377470003 | 1.29E-06 |
| MLST8 | AC124798.1 | 0.369256732 | 2.27E-06 |
| PELP1 | AC124798.1 | 0.331731869 | 2.48E-05 |
| RHEB | AC124798.1 | 0.313722648 | 7.04E-05 |
| WDR45 | AC124798.1 | 0.402564656 | 2.07E-07 |
| ATG10 | AC005840.4 | 0.416477534 | 7.04E-08 |
| ATG12 | AC005840.4 | 0.30758216 | 9.89E-05 |
| ATG3 | AC005840.4 | 0.36497862 | 3.02E-06 |
| ATG4A | AC005840.4 | 0.39691846 | 3.17E-07 |
| ATG4B | AC005840.4 | 0.519120473 | 4.51E-12 |
| ATG4C | AC005840.4 | 0.342593707 | 1.28E-05 |
| ATG4D | AC005840.4 | 0.396521934 | 3.26E-07 |
| ATG7 | AC005840.4 | 0.359386702 | 4.38E-06 |
| BAX | AC005840.4 | 0.453667216 | 3.06E-09 |
| BNIP1 | AC005840.4 | 0.428726604 | 2.61E-08 |
| BNIP3 | AC005840.4 | -0.303172021 | 0.000125792 |
| CAPN10 | AC005840.4 | 0.453716232 | 3.05E-09 |
| CAPNS1 | AC005840.4 | 0.399953186 | 2.52E-07 |
| CDKN1B | AC005840.4 | 0.341468265 | 1.37E-05 |
| CDKN2A | AC005840.4 | 0.591011657 | 5.70E-16 |
| CLN3 | AC005840.4 | 0.365971449 | 2.83E-06 |
| CX3CL1 | AC005840.4 | 0.342006358 | 1.33E-05 |
| CXCR4 | AC005840.4 | 0.372806874 | 1.78E-06 |
| DAPK2 | AC005840.4 | 0.324535869 | 3.79E-05 |
| GABARAP | AC005840.4 | 0.368440864 | 2.39E-06 |
| HDAC1 | AC005840.4 | 0.337085495 | 1.80E-05 |
| HDAC6 | AC005840.4 | 0.436984882 | 1.31E-08 |
| HIF1A | AC005840.4 | -0.372271783 | 1.84E-06 |
| IFNG | AC005840.4 | 0.329152109 | 2.89E-05 |
| IKBKB | AC005840.4 | 0.365410769 | 2.94E-06 |
| IKBKE | AC005840.4 | 0.434836831 | 1.57E-08 |
| MAP1LC3B | AC005840.4 | -0.330646519 | 2.65E-05 |
| MAP2K7 | AC005840.4 | 0.37435271 | 1.60E-06 |
| MTMR14 | AC005840.4 | 0.495230811 | 5.72E-11 |
| NCKAP1 | AC005840.4 | -0.354964589 | 5.84E-06 |
| NKX2-3 | AC005840.4 | 0.362454592 | 3.58E-06 |
| PEX14 | AC005840.4 | 0.438493028 | 1.15E-08 |
| PINK1 | AC005840.4 | -0.328711286 | 2.97E-05 |
| PRKAB1 | AC005840.4 | 0.33969079 | 1.53E-05 |
| RAB24 | AC005840.4 | 0.449812466 | 4.31E-09 |
| STK11 | AC005840.4 | 0.514292252 | 7.65E-12 |
| TP53 | AC005840.4 | 0.389338544 | 5.53E-07 |
| TP73 | AC005840.4 | 0.406430618 | 1.54E-07 |
| TSC2 | AC005840.4 | 0.479006038 | 2.88E-10 |
| ULK3 | AC005840.4 | 0.328208332 | 3.06E-05 |
| USP10 | AC005840.4 | -0.33589543 | 1.93E-05 |
| WDR45 | AC005840.4 | 0.497751405 | 4.42E-11 |
| CD46 | AC087481.3 | 0.314018604 | 6.92E-05 |
| CXCR4 | AC087481.3 | 0.397893572 | 2.94E-07 |
| HDAC6 | AC087481.3 | 0.462064057 | 1.43E-09 |
| IKBKB | AC087481.3 | 0.39083297 | 4.96E-07 |
| KLHL24 | AC087481.3 | 0.334563611 | 2.09E-05 |
| MAP2K7 | AC087481.3 | 0.312605867 | 7.49E-05 |
| PIK3R4 | AC087481.3 | 0.311918196 | 7.78E-05 |
| RAF1 | AC087481.3 | 0.304888564 | 0.000114619 |
| SIRT1 | AC087481.3 | 0.301355095 | 0.000138719 |
| TP73 | AC087481.3 | 0.329210917 | 2.88E-05 |
| TSC1 | AC087481.3 | 0.398424879 | 2.83E-07 |
| TSC2 | AC087481.3 | 0.380204867 | 1.06E-06 |
| ULK2 | AC087481.3 | 0.306168176 | 0.000106901 |
| ULK3 | AC087481.3 | 0.360192891 | 4.15E-06 |
| ATG16L2 | MIR222HG | 0.373253502 | 1.72E-06 |
| ATG3 | MIR222HG | 0.301655718 | 0.000136498 |
| ATG4B | MIR222HG | 0.308406586 | 9.46E-05 |
| CAPN10 | MIR222HG | 0.335154063 | 2.02E-05 |
| HDAC6 | MIR222HG | 0.310004308 | 8.66E-05 |
| IKBKB | MIR222HG | 0.435111081 | 1.53E-08 |
| WDR45 | MIR222HG | 0.386559749 | 6.76E-07 |
| ATG4C | PCED1B-AS1 | 0.325404983 | 3.60E-05 |
| ATG7 | PCED1B-AS1 | 0.350340339 | 7.86E-06 |
| BAX | PCED1B-AS1 | 0.460835362 | 1.60E-09 |
| BCL2 | PCED1B-AS1 | 0.344675232 | 1.12E-05 |
| CAPN10 | PCED1B-AS1 | 0.370964624 | 2.02E-06 |
| CCL2 | PCED1B-AS1 | 0.36244749 | 3.58E-06 |
| CCR2 | PCED1B-AS1 | 0.593757256 | 3.87E-16 |
| CTSD | PCED1B-AS1 | 0.315661988 | 6.31E-05 |
| CXCR4 | PCED1B-AS1 | 0.657122923 | 1.61E-20 |
| DRAM1 | PCED1B-AS1 | 0.377589735 | 1.28E-06 |
| GABARAP | PCED1B-AS1 | 0.366720229 | 2.69E-06 |
| HSPA5 | PCED1B-AS1 | -0.301089161 | 0.000140712 |
| IFNG | PCED1B-AS1 | 0.524646726 | 2.43E-12 |
| IKBKE | PCED1B-AS1 | 0.314935816 | 6.57E-05 |
| ITPR1 | PCED1B-AS1 | 0.450425879 | 4.09E-09 |
| MAP2K7 | PCED1B-AS1 | 0.321367896 | 4.56E-05 |
| MTMR14 | PCED1B-AS1 | 0.510632008 | 1.14E-11 |
| NCKAP1 | PCED1B-AS1 | -0.348434588 | 8.88E-06 |
| NKX2-3 | PCED1B-AS1 | 0.312733241 | 7.44E-05 |
| PRKCD | PCED1B-AS1 | 0.327586588 | 3.17E-05 |
| RAB1A | PCED1B-AS1 | -0.322437157 | 4.28E-05 |
| RAB24 | PCED1B-AS1 | 0.394720532 | 3.73E-07 |
| RGS19 | PCED1B-AS1 | 0.550761679 | 1.13E-13 |
| STK11 | PCED1B-AS1 | 0.347175796 | 9.61E-06 |
| TP53 | PCED1B-AS1 | 0.350004399 | 8.03E-06 |
| WDR45 | PCED1B-AS1 | 0.31453038 | 6.72E-05 |
| RAB1A | LINC01748 | 0.341130896 | 1.40E-05 |
| ATG16L2 | AP001453.3 | 0.401206801 | 2.30E-07 |
| DAPK2 | AP001453.3 | 0.336978302 | 1.81E-05 |
| FOXO1 | AP001453.3 | -0.30365641 | 0.00012254 |
| MLST8 | AP001453.3 | 0.303924651 | 0.000120774 |
| AMBRA1 | AC015912.3 | -0.303129602 | 0.00012608 |
| ARNT | AC015912.3 | -0.368648351 | 2.36E-06 |
| ARSB | AC015912.3 | -0.332780531 | 2.33E-05 |
| ATF4 | AC015912.3 | 0.363161173 | 3.41E-06 |
| ATG4B | AC015912.3 | 0.379595691 | 1.11E-06 |
| ATG4C | AC015912.3 | -0.300437532 | 0.000145709 |
| BID | AC015912.3 | 0.301881004 | 0.000134855 |
| BIRC6 | AC015912.3 | -0.31215016 | 7.68E-05 |
| CANX | AC015912.3 | -0.340351047 | 1.47E-05 |
| DDIT3 | AC015912.3 | 0.318031123 | 5.52E-05 |
| GABARAPL2 | AC015912.3 | 0.386910585 | 6.59E-07 |
| GOPC | AC015912.3 | -0.321406377 | 4.55E-05 |
| MAPK1 | AC015912.3 | -0.353928884 | 6.25E-06 |
| MTOR | AC015912.3 | -0.421796864 | 4.60E-08 |
| PIK3R4 | AC015912.3 | -0.308945267 | 9.18E-05 |
| PPP1R15A | AC015912.3 | 0.434298007 | 1.64E-08 |
| PTEN | AC015912.3 | -0.322838195 | 4.19E-05 |
| ARNT | CYTOR | -0.332950358 | 2.31E-05 |
| ATF4 | CYTOR | 0.305863788 | 0.000108692 |
| BIRC6 | CYTOR | -0.346472397 | 1.00E-05 |
| EEF2K | CYTOR | -0.384011869 | 8.11E-07 |
| EIF2AK3 | CYTOR | -0.371079929 | 2.00E-06 |
| KIF5B | CYTOR | -0.302445273 | 0.000130821 |
| MTOR | CYTOR | -0.301629719 | 0.000136689 |
| NBR1 | CYTOR | -0.313071773 | 7.30E-05 |
| PIK3R4 | CYTOR | -0.343193095 | 1.23E-05 |
| RAB33B | CYTOR | -0.307826699 | 9.76E-05 |
| RPS6KB1 | CYTOR | -0.314662986 | 6.67E-05 |
| WDFY3 | CYTOR | -0.321442941 | 4.54E-05 |
| ATG3 | C2orf48 | 0.302024526 | 0.000133819 |
| ATG4A | C2orf48 | 0.305304956 | 0.000112052 |
| BIRC5 | C2orf48 | 0.321931269 | 4.41E-05 |
| CDKN2A | C2orf48 | 0.363275472 | 3.39E-06 |
| CHMP4B | C2orf48 | 0.454283175 | 2.90E-09 |
| DAPK2 | C2orf48 | 0.306891232 | 0.000102758 |
| FAS | C2orf48 | 0.354063522 | 6.19E-06 |
| MAPK8 | C2orf48 | 0.366669599 | 2.70E-06 |
| NKX2-3 | C2orf48 | 0.350704833 | 7.68E-06 |
| PEX14 | C2orf48 | 0.310941011 | 8.22E-05 |
| RAF1 | C2orf48 | 0.347728675 | 9.28E-06 |
| STK11 | C2orf48 | 0.430659578 | 2.23E-08 |
| TP73 | C2orf48 | 0.349882502 | 8.10E-06 |
| WDR45 | C2orf48 | 0.315149512 | 6.49E-05 |
| ARNT | AC073569.2 | 0.302441712 | 0.000130847 |
| ATF6 | AC073569.2 | 0.33148015 | 2.52E-05 |
| ATG4B | AC073569.2 | -0.331477481 | 2.52E-05 |
| BAX | AC073569.2 | -0.367968844 | 2.47E-06 |
| BIRC6 | AC073569.2 | 0.355315058 | 5.71E-06 |
| BNIP1 | AC073569.2 | -0.33544492 | 1.98E-05 |
| CAMKK2 | AC073569.2 | 0.352388797 | 6.90E-06 |
| CANX | AC073569.2 | 0.306936168 | 0.000102505 |
| CLN3 | AC073569.2 | -0.302160772 | 0.000132841 |
| EGFR | AC073569.2 | 0.333554581 | 2.22E-05 |
| EIF4G1 | AC073569.2 | 0.423164922 | 4.12E-08 |
| FOXO3 | AC073569.2 | 0.338395336 | 1.66E-05 |
| GOPC | AC073569.2 | 0.438363011 | 1.16E-08 |
| HGS | AC073569.2 | 0.338662988 | 1.63E-05 |
| HIF1A | AC073569.2 | 0.411346147 | 1.05E-07 |
| ITGB1 | AC073569.2 | 0.357832616 | 4.85E-06 |
| KIF5B | AC073569.2 | 0.349874469 | 8.10E-06 |
| MAPK1 | AC073569.2 | 0.381066865 | 1.00E-06 |
| NAF1 | AC073569.2 | 0.371345151 | 1.96E-06 |
| NBR1 | AC073569.2 | 0.316278718 | 6.09E-05 |
| NCKAP1 | AC073569.2 | 0.463899608 | 1.21E-09 |
| NPC1 | AC073569.2 | 0.481296674 | 2.31E-10 |
| RAB24 | AC073569.2 | -0.405946414 | 1.60E-07 |
| SPNS1 | AC073569.2 | 0.311459365 | 7.99E-05 |
| SQSTM1 | AC073569.2 | 0.309706354 | 8.80E-05 |
| TM9SF1 | AC073569.2 | 0.338874786 | 1.61E-05 |
| TP63 | AC073569.2 | 0.385151678 | 7.48E-07 |
| ULK1 | AC073569.2 | 0.303183009 | 0.000125717 |
| USP10 | AC073569.2 | 0.547652806 | 1.65E-13 |
| WDFY3 | AC073569.2 | 0.364730536 | 3.07E-06 |
| WDR45 | AC073569.2 | -0.339667964 | 1.53E-05 |
| ATG16L2 | RAD51-AS1 | 0.441606234 | 8.82E-09 |
| ATG4B | RAD51-AS1 | 0.313849798 | 6.99E-05 |
| ATG4C | RAD51-AS1 | 0.355384614 | 5.69E-06 |
| CAPN10 | RAD51-AS1 | 0.439349044 | 1.07E-08 |
| CDKN1B | RAD51-AS1 | 0.350736582 | 7.67E-06 |
| CXCR4 | RAD51-AS1 | 0.495569762 | 5.53E-11 |
| DAPK2 | RAD51-AS1 | 0.369723659 | 2.19E-06 |
| HDAC6 | RAD51-AS1 | 0.458749524 | 1.94E-09 |
| IKBKB | RAD51-AS1 | 0.45110883 | 3.85E-09 |
| MAP2K7 | RAD51-AS1 | 0.374067537 | 1.63E-06 |
| PINK1 | RAD51-AS1 | -0.308346975 | 9.49E-05 |
| RAF1 | RAD51-AS1 | 0.397381628 | 3.06E-07 |
| STK11 | RAD51-AS1 | 0.305695664 | 0.000109693 |
| TSC1 | RAD51-AS1 | 0.416894033 | 6.81E-08 |
| TSC2 | RAD51-AS1 | 0.382078676 | 9.31E-07 |
| ULK3 | RAD51-AS1 | 0.498981619 | 3.89E-11 |
| UVRAG | RAD51-AS1 | 0.350377324 | 7.85E-06 |
| WDR45 | RAD51-AS1 | 0.346102449 | 1.03E-05 |
| EIF2S1 | LINC02154 | 0.315593547 | 6.33E-05 |
| GABARAPL2 | LINC02154 | 0.329952396 | 2.76E-05 |
| HIF1A | LINC02154 | 0.325425915 | 3.60E-05 |
| NPC1 | LINC02154 | 0.328281507 | 3.04E-05 |
| PEX3 | LINC02154 | 0.559020068 | 4.04E-14 |
| APOL1 | CALML3-AS1 | 0.307861399 | 9.74E-05 |
| CAPN2 | CALML3-AS1 | 0.345525729 | 1.07E-05 |
| RPS6KB1 | CALML3-AS1 | 0.390296863 | 5.16E-07 |
| ATG9B | AC108136.1 | 0.361889499 | 3.71E-06 |
| FOXO1 | AC108136.1 | -0.333304293 | 2.26E-05 |
| EEF2K | HM13-IT1 | 0.30539841 | 0.000111484 |
| EIF2AK3 | HM13-IT1 | 0.336084202 | 1.91E-05 |
| IKBKB | HM13-IT1 | 0.331095256 | 2.58E-05 |
| SPNS1 | HM13-IT1 | 0.398819343 | 2.75E-07 |
| TSC1 | HM13-IT1 | 0.308634586 | 9.34E-05 |
| DNAJB1 | AC046143.1 | 0.31346912 | 7.14E-05 |
| EIF2AK3 | AC046143.1 | 0.385512512 | 7.29E-07 |
| NFE2L2 | AC046143.1 | 0.395252052 | 3.58E-07 |
| ATG10 | AC104825.1 | 0.37628327 | 1.40E-06 |
| ATG3 | AC104825.1 | 0.348881485 | 8.63E-06 |
| ATG4A | AC104825.1 | 0.343765446 | 1.19E-05 |
| ATG4C | AC104825.1 | 0.37836368 | 1.21E-06 |
| ATG4D | AC104825.1 | 0.433211889 | 1.80E-08 |
| ATG7 | AC104825.1 | 0.40462708 | 1.77E-07 |
| BNIP3 | AC104825.1 | -0.324867885 | 3.72E-05 |
| CALCOCO2 | AC104825.1 | 0.308068219 | 9.63E-05 |
| CAPN10 | AC104825.1 | 0.340121018 | 1.49E-05 |
| CAPNS1 | AC104825.1 | 0.392346961 | 4.44E-07 |
| CASP3 | AC104825.1 | 0.366910408 | 2.65E-06 |
| CD46 | AC104825.1 | 0.32958745 | 2.82E-05 |
| CDKN1B | AC104825.1 | 0.35469213 | 5.95E-06 |
| CDKN2A | AC104825.1 | 0.363484655 | 3.34E-06 |
| CFLAR | AC104825.1 | 0.370411883 | 2.09E-06 |
| CXCR4 | AC104825.1 | 0.36052743 | 4.06E-06 |
| EEF2K | AC104825.1 | 0.345868894 | 1.04E-05 |
| GABARAP | AC104825.1 | 0.368247784 | 2.43E-06 |
| HDAC1 | AC104825.1 | 0.321734488 | 4.46E-05 |
| HDAC6 | AC104825.1 | 0.571850571 | 7.74E-15 |
| HSP90AB1 | AC104825.1 | -0.344085569 | 1.17E-05 |
| IKBKB | AC104825.1 | 0.327023009 | 3.28E-05 |
| IKBKE | AC104825.1 | 0.522947945 | 2.94E-12 |
| MAP2K7 | AC104825.1 | 0.439806871 | 1.03E-08 |
| MAPK8 | AC104825.1 | 0.369379583 | 2.25E-06 |
| MAPK9 | AC104825.1 | 0.313483966 | 7.13E-05 |
| MTMR14 | AC104825.1 | 0.48426695 | 1.72E-10 |
| MYC | AC104825.1 | -0.37053495 | 2.08E-06 |
| NKX2-3 | AC104825.1 | 0.47082983 | 6.31E-10 |
| NRG1 | AC104825.1 | -0.303771205 | 0.000121781 |
| PEX14 | AC104825.1 | 0.310688719 | 8.34E-05 |
| PRKAB1 | AC104825.1 | 0.356399427 | 5.32E-06 |
| RAF1 | AC104825.1 | 0.452951387 | 3.26E-09 |
| STK11 | AC104825.1 | 0.408445661 | 1.32E-07 |
| TP53 | AC104825.1 | 0.390965079 | 4.91E-07 |
| TP73 | AC104825.1 | 0.48673285 | 1.35E-10 |
| TSC2 | AC104825.1 | 0.461990444 | 1.44E-09 |
| ULK3 | AC104825.1 | 0.37038259 | 2.10E-06 |
| WDR45 | AC104825.1 | 0.423909269 | 3.88E-08 |
| ATG10 | AC016876.1 | 0.452584088 | 3.37E-09 |
| ATG3 | AC016876.1 | 0.418585838 | 5.95E-08 |
| ATG4B | AC016876.1 | 0.401227859 | 2.29E-07 |
| ATG4C | AC016876.1 | 0.36307241 | 3.43E-06 |
| ATG4D | AC016876.1 | 0.389164264 | 5.60E-07 |
| ATG7 | AC016876.1 | 0.475327918 | 4.11E-10 |
| ATIC | AC016876.1 | 0.315164712 | 6.49E-05 |
| BAG1 | AC016876.1 | 0.346515918 | 1.00E-05 |
| BAG3 | AC016876.1 | -0.360695473 | 4.02E-06 |
| BAX | AC016876.1 | 0.553267595 | 8.29E-14 |
| BCL2 | AC016876.1 | 0.317848585 | 5.57E-05 |
| BIRC5 | AC016876.1 | 0.458949117 | 1.90E-09 |
| BNIP1 | AC016876.1 | 0.529422375 | 1.42E-12 |
| CAPN10 | AC016876.1 | 0.395506609 | 3.52E-07 |
| CAPNS1 | AC016876.1 | 0.406029789 | 1.59E-07 |
| CDKN1B | AC016876.1 | 0.405750158 | 1.63E-07 |
| CDKN2A | AC016876.1 | 0.643346821 | 1.76E-19 |
| CLN3 | AC016876.1 | 0.356380819 | 5.33E-06 |
| CXCR4 | AC016876.1 | 0.401819972 | 2.19E-07 |
| FKBP1A | AC016876.1 | 0.36303161 | 3.44E-06 |
| GABARAP | AC016876.1 | 0.432125524 | 1.97E-08 |
| HDAC6 | AC016876.1 | 0.476313391 | 3.74E-10 |
| HIF1A | AC016876.1 | -0.309386458 | 8.96E-05 |
| HSPA5 | AC016876.1 | -0.34147638 | 1.37E-05 |
| IKBKE | AC016876.1 | 0.350996578 | 7.54E-06 |
| ITGA6 | AC016876.1 | -0.35683811 | 5.17E-06 |
| MAP1LC3B | AC016876.1 | -0.325098197 | 3.67E-05 |
| MAP2K7 | AC016876.1 | 0.416423825 | 7.07E-08 |
| MAPK8IP1 | AC016876.1 | 0.317724672 | 5.61E-05 |
| MTMR14 | AC016876.1 | 0.583318843 | 1.66E-15 |
| NCKAP1 | AC016876.1 | -0.310409883 | 8.47E-05 |
| NKX2-3 | AC016876.1 | 0.372863887 | 1.77E-06 |
| PELP1 | AC016876.1 | 0.349691772 | 8.20E-06 |
| PEX14 | AC016876.1 | 0.36238287 | 3.59E-06 |
| PINK1 | AC016876.1 | -0.326813059 | 3.32E-05 |
| PRKAB1 | AC016876.1 | 0.409840694 | 1.19E-07 |
| PTK6 | AC016876.1 | -0.319697532 | 5.02E-05 |
| RAB1A | AC016876.1 | -0.356832673 | 5.18E-06 |
| RAB24 | AC016876.1 | 0.326419409 | 3.40E-05 |
| RAF1 | AC016876.1 | 0.404789341 | 1.75E-07 |
| STK11 | AC016876.1 | 0.433060535 | 1.82E-08 |
| TP53 | AC016876.1 | 0.445489776 | 6.30E-09 |
| TP73 | AC016876.1 | 0.312561165 | 7.51E-05 |
| TSC2 | AC016876.1 | 0.410107677 | 1.16E-07 |
| WDR45 | AC016876.1 | 0.51498474 | 7.10E-12 |
| ATG10 | LNCTAM34A | 0.349223708 | 8.44E-06 |
| ATG3 | LNCTAM34A | 0.353528237 | 6.41E-06 |
| ATG4C | LNCTAM34A | 0.377300677 | 1.30E-06 |
| ATG4D | LNCTAM34A | 0.430648784 | 2.23E-08 |
| ATG7 | LNCTAM34A | 0.329873134 | 2.77E-05 |
| BAK1 | LNCTAM34A | -0.300876223 | 0.000142327 |
| BAX | LNCTAM34A | 0.418039999 | 6.22E-08 |
| BCL2 | LNCTAM34A | 0.310492937 | 8.43E-05 |
| BNIP3 | LNCTAM34A | -0.327677779 | 3.15E-05 |
| CAPNS1 | LNCTAM34A | 0.333734417 | 2.20E-05 |
| CASP3 | LNCTAM34A | 0.341920769 | 1.33E-05 |
| CD46 | LNCTAM34A | 0.478088064 | 3.15E-10 |
| CDKN1B | LNCTAM34A | 0.375938847 | 1.43E-06 |
| CDKN2A | LNCTAM34A | 0.451289452 | 3.78E-09 |
| CX3CL1 | LNCTAM34A | 0.413775045 | 8.72E-08 |
| CXCR4 | LNCTAM34A | 0.34815989 | 9.03E-06 |
| FAS | LNCTAM34A | 0.309756023 | 8.78E-05 |
| HDAC1 | LNCTAM34A | 0.333138104 | 2.28E-05 |
| HDAC6 | LNCTAM34A | 0.405833173 | 1.62E-07 |
| IKBKB | LNCTAM34A | 0.302287914 | 0.000131935 |
| IKBKE | LNCTAM34A | 0.428850989 | 2.59E-08 |
| ITGA3 | LNCTAM34A | -0.350709309 | 7.68E-06 |
| ITGA6 | LNCTAM34A | -0.334545674 | 2.09E-05 |
| ITGB4 | LNCTAM34A | -0.303862501 | 0.000121181 |
| KLHL24 | LNCTAM34A | 0.362595529 | 3.54E-06 |
| MAP2K7 | LNCTAM34A | 0.431107693 | 2.15E-08 |
| MTMR14 | LNCTAM34A | 0.383776242 | 8.25E-07 |
| NRG1 | LNCTAM34A | -0.310837098 | 8.27E-05 |
| PARP1 | LNCTAM34A | 0.350625555 | 7.72E-06 |
| PEX14 | LNCTAM34A | 0.322538851 | 4.26E-05 |
| PINK1 | LNCTAM34A | -0.367411706 | 2.57E-06 |
| PTK6 | LNCTAM34A | -0.371349656 | 1.96E-06 |
| RAF1 | LNCTAM34A | 0.417692865 | 6.39E-08 |
| STK11 | LNCTAM34A | 0.30547677 | 0.000111009 |
| TP53 | LNCTAM34A | 0.419488304 | 5.54E-08 |
| WDR45 | LNCTAM34A | 0.371017423 | 2.01E-06 |
| ATG2B | AC084859.1 | 0.31583255 | 6.25E-05 |
| BAX | AC084859.1 | -0.341564252 | 1.36E-05 |
| EGFR | AC084859.1 | 0.326864668 | 3.31E-05 |
| NCKAP1 | AC084859.1 | 0.30371274 | 0.000122167 |
| ATG16L2 | ADNP-AS1 | 0.456260222 | 2.43E-09 |
| ATG3 | ADNP-AS1 | 0.405707458 | 1.63E-07 |
| ATG4A | ADNP-AS1 | 0.338513602 | 1.65E-05 |
| ATG4B | ADNP-AS1 | 0.446168881 | 5.94E-09 |
| ATG9B | ADNP-AS1 | 0.346211325 | 1.02E-05 |
| CAPN10 | ADNP-AS1 | 0.456813861 | 2.31E-09 |
| CASP3 | ADNP-AS1 | 0.320467171 | 4.80E-05 |
| CDKN1B | ADNP-AS1 | 0.316612975 | 5.98E-05 |
| CDKN2A | ADNP-AS1 | 0.316564714 | 6.00E-05 |
| CFLAR | ADNP-AS1 | 0.376826485 | 1.35E-06 |
| CLN3 | ADNP-AS1 | 0.333378001 | 2.25E-05 |
| HDAC1 | ADNP-AS1 | 0.370733652 | 2.05E-06 |
| HDAC6 | ADNP-AS1 | 0.534623233 | 7.77E-13 |
| IKBKB | ADNP-AS1 | 0.467579939 | 8.57E-10 |
| ITGA3 | ADNP-AS1 | -0.308216322 | 9.56E-05 |
| MAP2K7 | ADNP-AS1 | 0.420186164 | 5.24E-08 |
| MAPK8 | ADNP-AS1 | 0.354705524 | 5.94E-06 |
| MLST8 | ADNP-AS1 | 0.312363103 | 7.59E-05 |
| MTMR14 | ADNP-AS1 | 0.312972269 | 7.34E-05 |
| PEX14 | ADNP-AS1 | 0.334333639 | 2.12E-05 |
| PINK1 | ADNP-AS1 | -0.351658362 | 7.23E-06 |
| PRKAB1 | ADNP-AS1 | 0.31838591 | 5.41E-05 |
| RAB24 | ADNP-AS1 | 0.306935224 | 0.00010251 |
| RAF1 | ADNP-AS1 | 0.455556745 | 2.58E-09 |
| STK11 | ADNP-AS1 | 0.39525505 | 3.58E-07 |
| TSC1 | ADNP-AS1 | 0.305673489 | 0.000109826 |
| TSC2 | ADNP-AS1 | 0.349104581 | 8.51E-06 |
| ULK3 | ADNP-AS1 | 0.460919476 | 1.59E-09 |
| WDR45 | ADNP-AS1 | 0.40215828 | 2.14E-07 |
| BAK1 | AC090114.2 | -0.320790614 | 4.71E-05 |
| ITGA3 | AC090114.2 | -0.37730063 | 1.30E-06 |
| NFE2L2 | AC090114.2 | 0.337377489 | 1.76E-05 |
| RHEB | AC090114.2 | 0.347453131 | 9.44E-06 |
| ATG3 | KDM4A-AS1 | 0.311882753 | 7.80E-05 |
| BIRC5 | KDM4A-AS1 | 0.332321479 | 2.39E-05 |
| CAPN2 | KDM4A-AS1 | -0.311975051 | 7.76E-05 |
| FOXO3 | KDM4A-AS1 | 0.333800164 | 2.19E-05 |
| PINK1 | KDM4A-AS1 | -0.31874707 | 5.30E-05 |
| FKBP1B | AC106875.1 | 0.419776197 | 5.41E-08 |
| HIF1A | AC106875.1 | 0.361549823 | 3.80E-06 |
| RAB1A | AC106875.1 | 0.360711014 | 4.01E-06 |
| RPS6KB1 | AC106875.1 | 0.430102688 | 2.33E-08 |
| ATG4C | LINP1 | -0.335494415 | 1.98E-05 |
| ATG4D | LINP1 | -0.314816533 | 6.62E-05 |
| ATG7 | LINP1 | -0.315213002 | 6.47E-05 |
| CAMKK2 | LINP1 | -0.315313916 | 6.43E-05 |
| CASP1 | LINP1 | 0.323431742 | 4.04E-05 |
| CASP4 | LINP1 | 0.383981013 | 8.13E-07 |
| CDKN1B | LINP1 | -0.349517974 | 8.29E-06 |
| EIF2AK3 | LINP1 | -0.352006378 | 7.07E-06 |
| HDAC6 | LINP1 | -0.364645264 | 3.09E-06 |
| IKBKB | LINP1 | -0.322451129 | 4.28E-05 |
| IKBKE | LINP1 | -0.303741795 | 0.000121975 |
| ITGA3 | LINP1 | 0.484002584 | 1.77E-10 |
| ITGA6 | LINP1 | 0.381373009 | 9.78E-07 |
| ITPR1 | LINP1 | -0.310043066 | 8.64E-05 |
| MAPK8 | LINP1 | -0.356001172 | 5.46E-06 |
| MTMR14 | LINP1 | -0.363207979 | 3.40E-06 |
| NRG1 | LINP1 | 0.383281482 | 8.54E-07 |
| PARP1 | LINP1 | -0.324416973 | 3.82E-05 |
| PRKAB1 | LINP1 | -0.322947992 | 4.16E-05 |
| RAB33B | LINP1 | -0.326511921 | 3.38E-05 |
| RAB5A | LINP1 | -0.304903387 | 0.000114527 |
| RAF1 | LINP1 | -0.371261931 | 1.98E-06 |
| SIRT1 | LINP1 | -0.302905113 | 0.000127618 |
| TP53 | LINP1 | -0.304015194 | 0.000120183 |
| ULK3 | LINP1 | -0.359309479 | 4.40E-06 |
| ATG10 | LINC01184 | 0.388426957 | 5.91E-07 |
| ATG12 | LINC01184 | 0.380312974 | 1.05E-06 |
| ATG4C | LINC01184 | 0.391632006 | 4.68E-07 |
| DAPK2 | LINC01184 | 0.350753164 | 7.66E-06 |
| MAPK8 | LINC01184 | 0.329649734 | 2.81E-05 |
| RAB33B | LINC01184 | 0.334946573 | 2.04E-05 |
| ATG12 | AC116407.2 | 0.370370012 | 2.10E-06 |
| ATG16L2 | AC116407.2 | 0.314212713 | 6.85E-05 |
| ATG4B | AC116407.2 | 0.337094783 | 1.79E-05 |
| BNIP1 | AC116407.2 | 0.379065369 | 1.15E-06 |
| CFLAR | AC116407.2 | 0.332644665 | 2.35E-05 |
| WDR45 | AC116407.2 | 0.47501402 | 4.24E-10 |
| GABARAPL1 | AL033397.1 | 0.335839592 | 1.94E-05 |
| NAMPT | AL033397.1 | 0.49322519 | 7.02E-11 |
| NFE2L2 | AL033397.1 | 0.325896742 | 3.50E-05 |
| RHEB | AL033397.1 | 0.350616978 | 7.73E-06 |
| SQSTM1 | AL033397.1 | 0.361644181 | 3.77E-06 |
| TP63 | AL033397.1 | 0.371231443 | 1.98E-06 |
| ATG10 | AC016205.1 | 0.422832042 | 4.23E-08 |
| ATG12 | AC016205.1 | 0.369285884 | 2.26E-06 |
| ATG4C | AC016205.1 | 0.56352388 | 2.28E-14 |
| ATG4D | AC016205.1 | 0.319840016 | 4.97E-05 |
| ATG7 | AC016205.1 | 0.354228101 | 6.13E-06 |
| BCL2 | AC016205.1 | 0.664502765 | 4.23E-21 |
| BNIP3L | AC016205.1 | 0.333441733 | 2.24E-05 |
| CASP3 | AC016205.1 | 0.386559732 | 6.76E-07 |
| CD46 | AC016205.1 | 0.320664365 | 4.74E-05 |
| CDKN1B | AC016205.1 | 0.42573975 | 3.34E-08 |
| CXCR4 | AC016205.1 | 0.340521007 | 1.45E-05 |
| DRAM1 | AC016205.1 | 0.449268298 | 4.53E-09 |
| EEF2K | AC016205.1 | 0.3051579 | 0.000112953 |
| FAS | AC016205.1 | 0.507286517 | 1.63E-11 |
| HDAC6 | AC016205.1 | 0.568772072 | 1.16E-14 |
| IKBKE | AC016205.1 | 0.324769106 | 3.74E-05 |
| MAP2K7 | AC016205.1 | 0.328171732 | 3.06E-05 |
| MAPK8 | AC016205.1 | 0.492152922 | 7.83E-11 |
| MAPK9 | AC016205.1 | 0.417492474 | 6.50E-08 |
| MTMR14 | AC016205.1 | 0.333790711 | 2.19E-05 |
| NKX2-3 | AC016205.1 | 0.511235461 | 1.07E-11 |
| PARP1 | AC016205.1 | 0.398853919 | 2.74E-07 |
| PEX3 | AC016205.1 | 0.310304353 | 8.51E-05 |
| PIK3C3 | AC016205.1 | 0.398635884 | 2.79E-07 |
| PRKAB1 | AC016205.1 | 0.333520916 | 2.23E-05 |
| PRKAR1A | AC016205.1 | 0.325158326 | 3.66E-05 |
| PTK6 | AC016205.1 | -0.302497961 | 0.000130451 |
| RAB33B | AC016205.1 | 0.391562216 | 4.70E-07 |
| RAF1 | AC016205.1 | 0.409765238 | 1.19E-07 |
| SIRT1 | AC016205.1 | 0.363557381 | 3.32E-06 |
| TP53 | AC016205.1 | 0.423945021 | 3.87E-08 |
| TP73 | AC016205.1 | 0.438153161 | 1.19E-08 |
| TSC2 | AC016205.1 | 0.448321836 | 4.92E-09 |
| ULK2 | AC016205.1 | 0.438378566 | 1.16E-08 |
| ATG16L2 | LINC00997 | 0.376919947 | 1.34E-06 |
| ATG2A | LINC00997 | 0.321930888 | 4.41E-05 |
| NKX2-3 | LINC00997 | 0.30638054 | 0.000105668 |
| ATG3 | AC009065.2 | 0.340961097 | 1.42E-05 |
| TSC1 | AC009065.2 | 0.306262575 | 0.000106352 |
| ATG4D | B3GALT5-AS1 | 0.339116634 | 1.59E-05 |
| CASP3 | B3GALT5-AS1 | 0.333650869 | 2.21E-05 |
| CFLAR | B3GALT5-AS1 | 0.304311375 | 0.000118268 |
| EEF2K | B3GALT5-AS1 | 0.301915812 | 0.000134603 |
| HDAC6 | B3GALT5-AS1 | 0.401901568 | 2.18E-07 |
| IKBKE | B3GALT5-AS1 | 0.348225979 | 8.99E-06 |
| MAPK9 | B3GALT5-AS1 | 0.323773054 | 3.96E-05 |
| NKX2-3 | B3GALT5-AS1 | 0.468059975 | 8.19E-10 |
| TP73 | B3GALT5-AS1 | 0.457481464 | 2.17E-09 |
| TSC2 | B3GALT5-AS1 | 0.465735287 | 1.02E-09 |
| WDR45 | B3GALT5-AS1 | 0.346264378 | 1.02E-05 |
| ATG9B | AC110619.1 | 0.460575481 | 1.64E-09 |
| BID | AC110619.1 | 0.330881349 | 2.61E-05 |
| CAPN2 | AC110619.1 | 0.306802841 | 0.000103256 |
| EIF2AK3 | AC110619.1 | 0.311300598 | 8.06E-05 |
| RAB11A | AC110619.1 | 0.494382004 | 6.24E-11 |
| TP53INP2 | AC110619.1 | 0.458649201 | 1.95E-09 |
| ATG4A | MIR503HG | 0.376350785 | 1.39E-06 |
| ATIC | MIR503HG | 0.549064872 | 1.39E-13 |
| BIRC5 | MIR503HG | 0.521194891 | 3.58E-12 |
| FKBP1A | MIR503HG | 0.30182643 | 0.000135252 |
| MAPK8IP1 | MIR503HG | 0.890847568 | 2.70E-54 |
| PRKAR1A | MIR503HG | 0.590786156 | 5.89E-16 |
| VAMP7 | MIR503HG | 0.423770021 | 3.92E-08 |
| ARNT | AC007342.4 | 0.383687807 | 8.30E-07 |
| ATF6 | AC007342.4 | 0.339516828 | 1.55E-05 |
| BECN1 | AC007342.4 | 0.320776518 | 4.71E-05 |
| BIRC6 | AC007342.4 | 0.368331756 | 2.41E-06 |
| EIF2AK3 | AC007342.4 | 0.355938007 | 5.49E-06 |
| EIF4G1 | AC007342.4 | 0.36787826 | 2.49E-06 |
| ITGA3 | AC007342.4 | -0.352125016 | 7.02E-06 |
| KLHL24 | AC007342.4 | 0.32402546 | 3.91E-05 |
| MAPK1 | AC007342.4 | 0.355532181 | 5.63E-06 |
| NBR1 | AC007342.4 | 0.326000043 | 3.48E-05 |
| NFE2L2 | AC007342.4 | 0.347038048 | 9.69E-06 |
| TP53INP2 | AC007342.4 | 0.318770102 | 5.29E-05 |
| TP63 | AC007342.4 | 0.31886815 | 5.26E-05 |
| ATG3 | LINC02014 | 0.397039345 | 3.14E-07 |
| EEF2K | LINC02014 | 0.30444295 | 0.000117427 |
| PIK3R4 | LINC02014 | 0.316253578 | 6.10E-05 |
| SIRT2 | LINC02014 | 0.355892636 | 5.50E-06 |
| DIRAS3 | LINC01410 | 0.519967718 | 4.10E-12 |
| FKBP1B | LINC01410 | 0.36652484 | 2.72E-06 |
| ATG3 | AC097103.2 | 0.328039913 | 3.09E-05 |
| BIRC5 | AC097103.2 | 0.319440581 | 5.09E-05 |
| CAPN2 | AC097103.2 | -0.309652286 | 8.83E-05 |
| GABARAPL2 | AC097103.2 | 0.348580596 | 8.79E-06 |
| ITGA3 | AC097103.2 | -0.351002605 | 7.54E-06 |
| ITGB4 | AC097103.2 | -0.377466959 | 1.29E-06 |
| KLHL24 | AC097103.2 | 0.341468882 | 1.37E-05 |
| MAPK8IP1 | AC097103.2 | 0.358615035 | 4.61E-06 |
| ST13 | AC097103.2 | 0.315177418 | 6.48E-05 |
| WIPI1 | AC097103.2 | 0.328787415 | 2.95E-05 |
| ATG10 | FOXD3-AS1 | 0.342323397 | 1.30E-05 |
| ATG16L2 | FOXD3-AS1 | 0.317098303 | 5.82E-05 |
| ATG4C | FOXD3-AS1 | 0.403817422 | 1.88E-07 |
| ATIC | FOXD3-AS1 | 0.48274374 | 2.00E-10 |
| BAX | FOXD3-AS1 | 0.373973658 | 1.64E-06 |
| BCL2 | FOXD3-AS1 | 0.313251496 | 7.23E-05 |
| BIRC5 | FOXD3-AS1 | 0.384717805 | 7.71E-07 |
| BNIP1 | FOXD3-AS1 | 0.412777425 | 9.43E-08 |
| CAPN10 | FOXD3-AS1 | 0.534768169 | 7.64E-13 |
| CAPN2 | FOXD3-AS1 | -0.304967375 | 0.000114129 |
| CDKN1B | FOXD3-AS1 | 0.314027063 | 6.92E-05 |
| CHMP4B | FOXD3-AS1 | 0.366185254 | 2.79E-06 |
| CLN3 | FOXD3-AS1 | 0.325004858 | 3.69E-05 |
| DIRAS3 | FOXD3-AS1 | 0.466799948 | 9.22E-10 |
| EIF4G1 | FOXD3-AS1 | -0.321017842 | 4.65E-05 |
| FKBP1A | FOXD3-AS1 | 0.336753239 | 1.83E-05 |
| FKBP1B | FOXD3-AS1 | 0.321926167 | 4.41E-05 |
| GABARAP | FOXD3-AS1 | 0.346833723 | 9.82E-06 |
| GABARAPL2 | FOXD3-AS1 | 0.319447873 | 5.09E-05 |
| HDAC6 | FOXD3-AS1 | 0.401849069 | 2.19E-07 |
| MAP1LC3A | FOXD3-AS1 | 0.550042044 | 1.23E-13 |
| MAPK8 | FOXD3-AS1 | 0.370872922 | 2.03E-06 |
| MAPK8IP1 | FOXD3-AS1 | 0.516734042 | 5.86E-12 |
| MLST8 | FOXD3-AS1 | 0.362113006 | 3.66E-06 |
| MTMR14 | FOXD3-AS1 | 0.383508495 | 8.41E-07 |
| NKX2-3 | FOXD3-AS1 | 0.347449484 | 9.45E-06 |
| PRKAB1 | FOXD3-AS1 | 0.32870777 | 2.97E-05 |
| PRKCQ | FOXD3-AS1 | 0.318422758 | 5.39E-05 |
| ULK3 | FOXD3-AS1 | 0.393908708 | 3.96E-07 |
| WDR45 | FOXD3-AS1 | 0.451775224 | 3.62E-09 |
| ATG16L2 | BOK-AS1 | 0.341497341 | 1.37E-05 |
| BID | BOK-AS1 | 0.383420828 | 8.46E-07 |
| DIRAS3 | BOK-AS1 | 0.381446149 | 9.73E-07 |
| FKBP1B | BOK-AS1 | 0.532453898 | 9.99E-13 |
| FOXO1 | BOK-AS1 | -0.386799508 | 6.64E-07 |
| MYC | BOK-AS1 | 0.316504427 | 6.02E-05 |
| PRKCQ | BOK-AS1 | 0.344948821 | 1.11E-05 |
| ARNT | MIR600HG | 0.326060727 | 3.47E-05 |
| ATG3 | MIR600HG | 0.339210473 | 1.58E-05 |
| ATG4C | MIR600HG | 0.365153139 | 2.99E-06 |
| ATG4D | MIR600HG | 0.341269898 | 1.39E-05 |
| BCL2 | MIR600HG | 0.423176564 | 4.12E-08 |
| CAPN10 | MIR600HG | 0.314395969 | 6.78E-05 |
| CASP4 | MIR600HG | -0.313296395 | 7.21E-05 |
| CCR2 | MIR600HG | 0.30503271 | 0.000113725 |
| CD46 | MIR600HG | 0.377338802 | 1.30E-06 |
| CDKN1B | MIR600HG | 0.488182576 | 1.17E-10 |
| CDKN2A | MIR600HG | 0.324871074 | 3.72E-05 |
| CXCR4 | MIR600HG | 0.332808464 | 2.33E-05 |
| EEF2 | MIR600HG | 0.347556836 | 9.38E-06 |
| EEF2K | MIR600HG | 0.415039054 | 7.89E-08 |
| EIF2AK3 | MIR600HG | 0.305232301 | 0.000112496 |
| HDAC6 | MIR600HG | 0.419138741 | 5.70E-08 |
| HSPA5 | MIR600HG | -0.342572617 | 1.28E-05 |
| IKBKB | MIR600HG | 0.38284198 | 8.82E-07 |
| IKBKE | MIR600HG | 0.33054362 | 2.66E-05 |
| ITGA3 | MIR600HG | -0.361608971 | 3.78E-06 |
| MAP1LC3B | MIR600HG | -0.301304496 | 0.000139097 |
| MAP2K7 | MIR600HG | 0.521474665 | 3.47E-12 |
| MAPK8 | MIR600HG | 0.330829767 | 2.62E-05 |
| MTMR14 | MIR600HG | 0.354752256 | 5.92E-06 |
| NKX2-3 | MIR600HG | 0.364844288 | 3.05E-06 |
| PARP1 | MIR600HG | 0.411170676 | 1.07E-07 |
| PIK3C3 | MIR600HG | 0.335748338 | 1.95E-05 |
| PIK3R4 | MIR600HG | 0.440540436 | 9.67E-09 |
| PINK1 | MIR600HG | -0.336046956 | 1.91E-05 |
| RAF1 | MIR600HG | 0.501041593 | 3.14E-11 |
| TP53 | MIR600HG | 0.48517123 | 1.58E-10 |
| TP73 | MIR600HG | 0.327311165 | 3.22E-05 |
| TSC1 | MIR600HG | 0.535849219 | 6.73E-13 |
| TSC2 | MIR600HG | 0.440702218 | 9.54E-09 |
| ULK3 | MIR600HG | 0.405903413 | 1.61E-07 |
| VEGFA | LINC02575 | 0.33781093 | 1.72E-05 |
| AMBRA1 | AC093278.2 | 0.313227921 | 7.23E-05 |
| ARNT | AC093278.2 | 0.327524455 | 3.18E-05 |
| ARSB | AC093278.2 | 0.539495819 | 4.39E-13 |
| ATG4C | AC093278.2 | 0.431863154 | 2.01E-08 |
| ATG7 | AC093278.2 | 0.314928 | 6.58E-05 |
| BCL2 | AC093278.2 | 0.463880236 | 1.21E-09 |
| BIRC6 | AC093278.2 | 0.309011426 | 9.15E-05 |
| BNIP3 | AC093278.2 | -0.360825547 | 3.98E-06 |
| CCL2 | AC093278.2 | 0.32496327 | 3.70E-05 |
| CCR2 | AC093278.2 | 0.683856716 | 1.06E-22 |
| CFLAR | AC093278.2 | 0.306690296 | 0.000103894 |
| CXCR4 | AC093278.2 | 0.455136514 | 2.68E-09 |
| DAPK1 | AC093278.2 | 0.411840002 | 1.01E-07 |
| DLC1 | AC093278.2 | 0.332521054 | 2.37E-05 |
| DRAM1 | AC093278.2 | 0.498127976 | 4.25E-11 |
| EDEM1 | AC093278.2 | 0.380894269 | 1.01E-06 |
| EEF2K | AC093278.2 | 0.318581638 | 5.35E-05 |
| ERN1 | AC093278.2 | 0.301434366 | 0.00013813 |
| FAS | AC093278.2 | 0.409904664 | 1.18E-07 |
| IKBKE | AC093278.2 | 0.310952337 | 8.21E-05 |
| ITPR1 | AC093278.2 | 0.701728136 | 2.70E-24 |
| MTMR14 | AC093278.2 | 0.315287885 | 6.44E-05 |
| MTOR | AC093278.2 | 0.302803583 | 0.000128319 |
| NBR1 | AC093278.2 | 0.332466333 | 2.37E-05 |
| PARP1 | AC093278.2 | 0.341849685 | 1.34E-05 |
| PRKCD | AC093278.2 | 0.3480732 | 9.08E-06 |
| RGS19 | AC093278.2 | 0.475774171 | 3.94E-10 |
| SIRT1 | AC093278.2 | 0.309912682 | 8.70E-05 |
| TP53 | AC093278.2 | 0.345364929 | 1.08E-05 |
| TSC2 | AC093278.2 | 0.33705073 | 1.80E-05 |
| ULK2 | AC093278.2 | 0.405729867 | 1.63E-07 |
| ATG16L2 | AC016877.3 | 0.308777654 | 9.26E-05 |
| ATG4C | AC016877.3 | 0.39387911 | 3.97E-07 |
| ATIC | AC016877.3 | 0.441537933 | 8.88E-09 |
| BIRC5 | AC016877.3 | 0.484272318 | 1.72E-10 |
| CDKN1B | AC016877.3 | 0.357450034 | 4.97E-06 |
| HDAC6 | AC016877.3 | 0.387108002 | 6.50E-07 |
| MAPK8IP1 | AC016877.3 | 0.638173449 | 4.21E-19 |
| MTMR14 | AC016877.3 | 0.324465152 | 3.81E-05 |
| PRKAR1A | AC016877.3 | 0.506273263 | 1.81E-11 |
| PRKCQ | AC016877.3 | 0.370079058 | 2.14E-06 |
| RAF1 | AC016877.3 | 0.301762149 | 0.00013572 |
| VAMP7 | AC016877.3 | 0.330054162 | 2.74E-05 |
| ATG10 | LINC00339 | 0.321198746 | 4.60E-05 |
| ATG16L2 | LINC00339 | 0.316111776 | 6.15E-05 |
| ATG4C | LINC00339 | 0.460793474 | 1.61E-09 |
| BAG3 | LINC00339 | -0.322046061 | 4.38E-05 |
| BAK1 | LINC00339 | -0.312607644 | 7.49E-05 |
| BAX | LINC00339 | 0.3664143 | 2.75E-06 |
| BCL2 | LINC00339 | 0.343742176 | 1.19E-05 |
| CAPN1 | LINC00339 | -0.307531802 | 9.92E-05 |
| CAPN10 | LINC00339 | 0.42309598 | 4.14E-08 |
| CDKN1B | LINC00339 | 0.445366992 | 6.37E-09 |
| CXCR4 | LINC00339 | 0.548651592 | 1.46E-13 |
| DRAM1 | LINC00339 | 0.309464121 | 8.92E-05 |
| HDAC6 | LINC00339 | 0.424221459 | 3.78E-08 |
| MAP2K7 | LINC00339 | 0.359604717 | 4.32E-06 |
| MLST8 | LINC00339 | 0.305365453 | 0.000111684 |
| PEX14 | LINC00339 | 0.351751307 | 7.19E-06 |
| PRKAB1 | LINC00339 | 0.335855555 | 1.93E-05 |
| PTK6 | LINC00339 | -0.315063048 | 6.53E-05 |
| STK11 | LINC00339 | 0.325720089 | 3.54E-05 |
| TP53 | LINC00339 | 0.324077664 | 3.89E-05 |
| TP73 | LINC00339 | 0.392198832 | 4.49E-07 |
| TSC2 | LINC00339 | 0.373980254 | 1.64E-06 |
| WDR45 | LINC00339 | 0.326851218 | 3.31E-05 |
| ATG4D | AC004130.1 | 0.300401167 | 0.000145993 |
| ATG9B | AC004130.1 | 0.32107387 | 4.63E-05 |
| GABARAPL1 | AC004130.1 | 0.331415872 | 2.53E-05 |
| NFE2L2 | AC004130.1 | 0.300293755 | 0.000146834 |
| TP53INP2 | AC004130.1 | 0.351115894 | 7.48E-06 |
| ATG16L2 | AL512791.1 | 0.318214638 | 5.46E-05 |
| VEGFA | AL512791.1 | 0.370280514 | 2.11E-06 |
| CAPN10 | AC132872.1 | 0.347917956 | 9.17E-06 |
| WDR45 | AC132872.1 | 0.363229403 | 3.40E-06 |
| WIPI1 | AC132872.1 | 0.424932728 | 3.57E-08 |
| ATG16L2 | AL022322.1 | 0.365176647 | 2.98E-06 |
| ATG4B | AL022322.1 | 0.397876805 | 2.95E-07 |
| ATG4C | AL022322.1 | 0.321432735 | 4.54E-05 |
| BAG3 | AL022322.1 | -0.304154301 | 0.00011928 |
| CAPN10 | AL022322.1 | 0.428292974 | 2.71E-08 |
| CX3CL1 | AL022322.1 | 0.303531993 | 0.000123368 |
| CXCR4 | AL022322.1 | 0.498778149 | 3.97E-11 |
| DAPK2 | AL022322.1 | 0.405906869 | 1.61E-07 |
| HDAC6 | AL022322.1 | 0.448887963 | 4.68E-09 |
| IKBKB | AL022322.1 | 0.405387969 | 1.67E-07 |
| MAP2K7 | AL022322.1 | 0.373700193 | 1.67E-06 |
| PELP1 | AL022322.1 | 0.386601002 | 6.74E-07 |
| PINK1 | AL022322.1 | -0.314128299 | 6.88E-05 |
| RAF1 | AL022322.1 | 0.310795656 | 8.29E-05 |
| TP53 | AL022322.1 | 0.332423047 | 2.38E-05 |
| TSC2 | AL022322.1 | 0.405085745 | 1.71E-07 |
| ULK3 | AL022322.1 | 0.394881915 | 3.68E-07 |
| WDR45 | AL022322.1 | 0.380794887 | 1.02E-06 |
| BNIP3 | AP000808.2 | 0.359352794 | 4.39E-06 |
| EIF4EBP1 | AP000808.2 | 0.35842866 | 4.66E-06 |
| FADD | AP000808.2 | 0.468490392 | 7.87E-10 |
| PEX3 | AP000808.2 | 0.310507465 | 8.42E-05 |
| CASP8 | AC008555.5 | 0.37585999 | 1.44E-06 |
| ITGA3 | LINC00346 | 0.323886269 | 3.94E-05 |
| ITGB4 | LINC00346 | 0.32714837 | 3.25E-05 |
| PINK1 | LINC00346 | 0.320093655 | 4.90E-05 |
| ATG7 | ZNF503-AS1 | 0.33434546 | 2.12E-05 |
| DIRAS3 | ZNF503-AS1 | 0.41784876 | 6.32E-08 |
| MTMR14 | ZNF503-AS1 | 0.308921861 | 9.19E-05 |
| RAF1 | ZNF503-AS1 | 0.325828317 | 3.52E-05 |
| ARNT | AC060780.1 | 0.35677181 | 5.20E-06 |
| ARSB | AC060780.1 | 0.348854031 | 8.64E-06 |
| ATG4C | AC060780.1 | 0.363887398 | 3.25E-06 |
| ATG4D | AC060780.1 | 0.314992816 | 6.55E-05 |
| BAK1 | AC060780.1 | -0.391036084 | 4.89E-07 |
| BCL2 | AC060780.1 | 0.455347119 | 2.63E-09 |
| BECN1 | AC060780.1 | 0.33986772 | 1.51E-05 |
| BIRC6 | AC060780.1 | 0.376257449 | 1.40E-06 |
| CAMKK2 | AC060780.1 | 0.331156254 | 2.57E-05 |
| CASP3 | AC060780.1 | 0.332425991 | 2.38E-05 |
| CASP4 | AC060780.1 | -0.318614152 | 5.34E-05 |
| CD46 | AC060780.1 | 0.48025106 | 2.55E-10 |
| CDKN1A | AC060780.1 | -0.434078623 | 1.67E-08 |
| CDKN1B | AC060780.1 | 0.477314573 | 3.40E-10 |
| CXCR4 | AC060780.1 | 0.405255127 | 1.69E-07 |
| EEF2K | AC060780.1 | 0.437009476 | 1.31E-08 |
| EIF2AK3 | AC060780.1 | 0.414850021 | 8.01E-08 |
| ERN1 | AC060780.1 | 0.322950072 | 4.16E-05 |
| FAS | AC060780.1 | 0.365569305 | 2.91E-06 |
| HDAC6 | AC060780.1 | 0.526847792 | 1.90E-12 |
| IKBKB | AC060780.1 | 0.328376787 | 3.03E-05 |
| IKBKE | AC060780.1 | 0.310711884 | 8.32E-05 |
| ITPR1 | AC060780.1 | 0.30404941 | 0.00011996 |
| KLHL24 | AC060780.1 | 0.479253431 | 2.82E-10 |
| MAP2K7 | AC060780.1 | 0.384376585 | 7.90E-07 |
| MAPK8 | AC060780.1 | 0.447234173 | 5.41E-09 |
| MAPK9 | AC060780.1 | 0.426588758 | 3.12E-08 |
| MBTPS2 | AC060780.1 | 0.352850065 | 6.70E-06 |
| MTOR | AC060780.1 | 0.368616747 | 2.37E-06 |
| NBR1 | AC060780.1 | 0.532122921 | 1.04E-12 |
| NKX2-3 | AC060780.1 | 0.349168382 | 8.47E-06 |
| PARP1 | AC060780.1 | 0.589550382 | 7.00E-16 |
| PIK3R4 | AC060780.1 | 0.31650549 | 6.02E-05 |
| PRKAB1 | AC060780.1 | 0.41128434 | 1.06E-07 |
| PRKAR1A | AC060780.1 | 0.30426975 | 0.000118536 |
| PTK6 | AC060780.1 | -0.359627994 | 4.31E-06 |
| RAB33B | AC060780.1 | 0.406722237 | 1.51E-07 |
| RAF1 | AC060780.1 | 0.309385823 | 8.96E-05 |
| RB1CC1 | AC060780.1 | 0.379302578 | 1.13E-06 |
| SESN2 | AC060780.1 | -0.325248315 | 3.64E-05 |
| SIRT1 | AC060780.1 | 0.541165515 | 3.60E-13 |
| SQSTM1 | AC060780.1 | 0.338662098 | 1.63E-05 |
| ST13 | AC060780.1 | 0.376317412 | 1.39E-06 |
| TP53 | AC060780.1 | 0.303490086 | 0.000123648 |
| TP73 | AC060780.1 | 0.399405111 | 2.63E-07 |
| TSC1 | AC060780.1 | 0.342914094 | 1.25E-05 |
| TSC2 | AC060780.1 | 0.399764017 | 2.56E-07 |
| ULK2 | AC060780.1 | 0.520264479 | 3.97E-12 |
| UVRAG | AC060780.1 | 0.445511572 | 6.29E-09 |
| VAMP7 | AC060780.1 | 0.36120096 | 3.89E-06 |
| ATG4D | AC105277.1 | -0.305086803 | 0.00011339 |
| BAK1 | AC105277.1 | 0.396187529 | 3.34E-07 |
| BID | AC105277.1 | -0.336411075 | 1.87E-05 |
| BIRC6 | AC105277.1 | -0.375633213 | 1.46E-06 |
| CAMKK2 | AC105277.1 | -0.428581975 | 2.64E-08 |
| CASP3 | AC105277.1 | -0.309291878 | 9.01E-05 |
| CD46 | AC105277.1 | -0.345342882 | 1.08E-05 |
| CDKN1B | AC105277.1 | -0.330479114 | 2.67E-05 |
| EIF2AK3 | AC105277.1 | -0.471432597 | 5.96E-10 |
| IKBKB | AC105277.1 | -0.314159861 | 6.87E-05 |
| IL24 | AC105277.1 | 0.353880403 | 6.27E-06 |
| ITGA3 | AC105277.1 | 0.457645337 | 2.14E-09 |
| KIF5B | AC105277.1 | -0.315222204 | 6.47E-05 |
| MAP2K7 | AC105277.1 | -0.33834098 | 1.66E-05 |
| MAPK8 | AC105277.1 | -0.332414242 | 2.38E-05 |
| NAF1 | AC105277.1 | -0.349768358 | 8.16E-06 |
| NBR1 | AC105277.1 | -0.305643068 | 0.000110008 |
| NFE2L2 | AC105277.1 | -0.391751053 | 4.64E-07 |
| PARP1 | AC105277.1 | -0.396687719 | 3.22E-07 |
| PIK3R4 | AC105277.1 | -0.312495959 | 7.54E-05 |
| PINK1 | AC105277.1 | 0.381309333 | 9.83E-07 |
| PRKAB1 | AC105277.1 | -0.354780531 | 5.91E-06 |
| PRKCD | AC105277.1 | -0.351432659 | 7.33E-06 |
| RAF1 | AC105277.1 | -0.352338394 | 6.92E-06 |
| RHEB | AC105277.1 | -0.305570196 | 0.000110446 |
| RPS6KB1 | AC105277.1 | -0.376903904 | 1.34E-06 |
| SIRT1 | AC105277.1 | -0.359779073 | 4.27E-06 |
| CAPN10 | PRKCQ-AS1 | 0.314069844 | 6.90E-05 |
| DIRAS3 | PRKCQ-AS1 | 0.337011854 | 1.80E-05 |
| FKBP1B | PRKCQ-AS1 | 0.316497463 | 6.02E-05 |
| FOXO1 | PRKCQ-AS1 | -0.323826574 | 3.95E-05 |
| PRKCQ | PRKCQ-AS1 | 0.838717614 | 3.16E-42 |
| TP63 | PRKCQ-AS1 | -0.312114369 | 7.70E-05 |
| CX3CL1 | AC009041.2 | 0.326604082 | 3.36E-05 |
| CXCR4 | AC009041.2 | 0.592720035 | 4.48E-16 |
| DAPK2 | AC009041.2 | 0.303577656 | 0.000123063 |
| SPHK1 | AC009041.2 | 0.316764256 | 5.93E-05 |
| TP53 | AC009041.2 | 0.354361361 | 6.08E-06 |
| TSC2 | AC009041.2 | 0.494020935 | 6.48E-11 |
| UVRAG | AC009041.2 | 0.341792349 | 1.34E-05 |
| ATG3 | BX284668.5 | 0.314818299 | 6.62E-05 |
| ATIC | BX284668.5 | 0.472049294 | 5.62E-10 |
| BIRC5 | BX284668.5 | 0.332826758 | 2.32E-05 |
| CLN3 | BX284668.5 | 0.309641642 | 8.83E-05 |
| GABARAPL2 | BX284668.5 | 0.405506026 | 1.66E-07 |
| MAPK8IP1 | BX284668.5 | 0.592001565 | 4.96E-16 |
| PRKAR1A | BX284668.5 | 0.402465718 | 2.09E-07 |
| ATG16L2 | LINC00665 | 0.30423459 | 0.000118762 |
| BID | LINC00665 | 0.372882152 | 1.77E-06 |
| DAPK2 | LINC00665 | 0.302288331 | 0.000131932 |
| NAF1 | LINC00665 | 0.310967871 | 8.21E-05 |
| ATIC | KANSL1-AS1 | 0.319303415 | 5.13E-05 |
| FKBP1A | KANSL1-AS1 | 0.335415378 | 1.99E-05 |
| ATF6 | KC877982.1 | 0.326844555 | 3.31E-05 |
| PIK3C3 | KC877982.1 | 0.36880329 | 2.34E-06 |
| ATG4D | LINC02012 | 0.339136538 | 1.58E-05 |
| EIF2AK3 | LINC02012 | 0.307115267 | 0.000101505 |
| PINK1 | LINC02012 | -0.326093311 | 3.46E-05 |
| ATG4C | HCG18 | 0.347932679 | 9.16E-06 |
| BCL2 | HCG18 | 0.300826116 | 0.00014271 |
| BIRC6 | HCG18 | 0.396542848 | 3.26E-07 |
| EEF2K | HCG18 | 0.331782933 | 2.47E-05 |
| HDAC6 | HCG18 | 0.336933633 | 1.81E-05 |
| HSP90AB1 | HCG18 | 0.317469283 | 5.70E-05 |
| MTOR | HCG18 | 0.364209629 | 3.18E-06 |
| NAF1 | HCG18 | 0.373763762 | 1.66E-06 |
| PARP1 | HCG18 | 0.374412506 | 1.59E-06 |
| RPS6KB1 | HCG18 | 0.304940995 | 0.000114293 |
| TP53 | HCG18 | 0.340243152 | 1.48E-05 |
| TSC1 | HCG18 | 0.319417564 | 5.10E-05 |
| AMBRA1 | LINC00294 | 0.330163795 | 2.72E-05 |
| ATIC | LINC00294 | 0.385037348 | 7.54E-07 |
| BIRC5 | LINC00294 | 0.302279785 | 0.000131993 |
| HDAC6 | LINC00294 | 0.39530624 | 3.57E-07 |
| MAPK8IP1 | LINC00294 | 0.404797837 | 1.75E-07 |
| MAPK9 | LINC00294 | 0.349022093 | 8.55E-06 |
| NKX2-3 | LINC00294 | 0.30536897 | 0.000111663 |
| PRKAR1A | LINC00294 | 0.363470527 | 3.34E-06 |
| PRKCQ | LINC00294 | 0.300556052 | 0.000144788 |
| TP73 | LINC00294 | 0.304878811 | 0.00011468 |
| CX3CL1 | HLA-DQB1-AS1 | 0.336092601 | 1.91E-05 |
| CXCR4 | HLA-DQB1-AS1 | 0.39730868 | 3.08E-07 |
| FAS | HLA-DQB1-AS1 | 0.302296823 | 0.000131872 |
| IFNG | HLA-DQB1-AS1 | 0.463347356 | 1.27E-09 |
| NCKAP1 | HLA-DQB1-AS1 | -0.332005039 | 2.44E-05 |
| RAB24 | HLA-DQB1-AS1 | 0.362529601 | 3.56E-06 |
| RGS19 | HLA-DQB1-AS1 | 0.332798568 | 2.33E-05 |
| TP73 | HLA-DQB1-AS1 | 0.30375136 | 0.000121912 |
| TSC2 | HLA-DQB1-AS1 | 0.309280603 | 9.01E-05 |
| TUSC1 | HLA-DQB1-AS1 | 0.327628394 | 3.16E-05 |
| ATG7 | AL357033.3 | 0.311679331 | 7.89E-05 |
| CCR2 | AL357033.3 | 0.327198701 | 3.24E-05 |
| CFLAR | AL357033.3 | 0.321828732 | 4.44E-05 |
| CHMP2B | AL357033.3 | 0.381956434 | 9.39E-07 |
| RAF1 | AL357033.3 | 0.352782953 | 6.73E-06 |
| ATG16L2 | AP001033.2 | 0.314406173 | 6.77E-05 |
| GABARAPL2 | AP001033.2 | 0.300582739 | 0.000144581 |
| VEGFA | AP001033.2 | 0.383360766 | 8.50E-07 |
| ATG3 | MELTF-AS1 | 0.389499799 | 5.46E-07 |
| ATG4B | MELTF-AS1 | 0.333429241 | 2.24E-05 |
| BAG1 | MELTF-AS1 | 0.339661161 | 1.53E-05 |
| BAX | MELTF-AS1 | 0.50440732 | 2.21E-11 |
| BIRC5 | MELTF-AS1 | 0.482576527 | 2.04E-10 |
| CAPN10 | MELTF-AS1 | 0.353051356 | 6.61E-06 |
| CDKN1B | MELTF-AS1 | 0.3114104 | 8.01E-05 |
| CDKN2A | MELTF-AS1 | 0.366160445 | 2.79E-06 |
| CXCR4 | MELTF-AS1 | 0.341828287 | 1.34E-05 |
| HDAC6 | MELTF-AS1 | 0.375361253 | 1.49E-06 |
| ITGA3 | MELTF-AS1 | -0.345929422 | 1.04E-05 |
| ITGB4 | MELTF-AS1 | -0.302157406 | 0.000132865 |
| MAP2K7 | MELTF-AS1 | 0.394208406 | 3.87E-07 |
| PELP1 | MELTF-AS1 | 0.388459537 | 5.89E-07 |
| PINK1 | MELTF-AS1 | -0.421379832 | 4.76E-08 |
| PTK6 | MELTF-AS1 | -0.307268261 | 0.000100657 |
| STK11 | MELTF-AS1 | 0.362326278 | 3.61E-06 |
| WDR45 | MELTF-AS1 | 0.385682346 | 7.20E-07 |
| ATG16L2 | AC008280.3 | 0.401107157 | 2.31E-07 |
| BID | AC008280.3 | 0.370560909 | 2.07E-06 |
| CAPN10 | AC008280.3 | 0.303672406 | 0.000122434 |
| ATG10 | AL121929.2 | 0.317350795 | 5.73E-05 |
| ATG3 | AL121929.2 | 0.391527959 | 4.71E-07 |
| BAG1 | AL121929.2 | 0.333024501 | 2.30E-05 |
| BAX | AL121929.2 | 0.371648981 | 1.92E-06 |
| CDKN2A | AL121929.2 | 0.397699251 | 2.99E-07 |
| CHMP4B | AL121929.2 | 0.362843163 | 3.49E-06 |
| HDAC6 | AL121929.2 | 0.308994265 | 9.15E-05 |
| MAP2K7 | AL121929.2 | 0.331324796 | 2.54E-05 |
| MAPK8 | AL121929.2 | 0.300776792 | 0.000143087 |
| MTMR14 | AL121929.2 | 0.324171283 | 3.87E-05 |
| NKX2-3 | AL121929.2 | 0.385069684 | 7.52E-07 |
| PEX14 | AL121929.2 | 0.307235996 | 0.000100835 |
| RAF1 | AL121929.2 | 0.379294296 | 1.13E-06 |
| STK11 | AL121929.2 | 0.338137224 | 1.68E-05 |
| TSC1 | AL121929.2 | 0.303948936 | 0.000120615 |
| WDR45 | AL121929.2 | 0.341213933 | 1.39E-05 |
| ATG10 | AC036176.1 | 0.36748598 | 2.55E-06 |
| ATG4B | AC036176.1 | 0.385690819 | 7.19E-07 |
| ATG4C | AC036176.1 | 0.469863176 | 6.92E-10 |
| ATG4D | AC036176.1 | 0.345424109 | 1.07E-05 |
| ATG7 | AC036176.1 | 0.386023054 | 7.02E-07 |
| BAG3 | AC036176.1 | -0.4129863 | 9.28E-08 |
| BAX | AC036176.1 | 0.441241207 | 9.11E-09 |
| BCL2 | AC036176.1 | 0.520897435 | 3.70E-12 |
| BNIP1 | AC036176.1 | 0.45662279 | 2.35E-09 |
| CAPN10 | AC036176.1 | 0.397755632 | 2.98E-07 |
| CAPNS1 | AC036176.1 | 0.315382046 | 6.41E-05 |
| CDKN1B | AC036176.1 | 0.399838904 | 2.55E-07 |
| CDKN2A | AC036176.1 | 0.467884694 | 8.33E-10 |
| CFLAR | AC036176.1 | 0.317714595 | 5.62E-05 |
| CXCR4 | AC036176.1 | 0.555906298 | 5.98E-14 |
| DRAM1 | AC036176.1 | 0.372430266 | 1.82E-06 |
| EIF2S1 | AC036176.1 | -0.31889039 | 5.25E-05 |
| EIF4G1 | AC036176.1 | -0.378275596 | 1.22E-06 |
| FAS | AC036176.1 | 0.383379612 | 8.49E-07 |
| FKBP1A | AC036176.1 | 0.387295731 | 6.41E-07 |
| GABARAP | AC036176.1 | 0.370758612 | 2.05E-06 |
| HDAC6 | AC036176.1 | 0.501707402 | 2.93E-11 |
| HIF1A | AC036176.1 | -0.402286522 | 2.12E-07 |
| HSP90AB1 | AC036176.1 | -0.302920537 | 0.000127511 |
| IKBKE | AC036176.1 | 0.426050352 | 3.26E-08 |
| ITGA6 | AC036176.1 | -0.328215644 | 3.06E-05 |
| MAP1LC3B | AC036176.1 | -0.351012499 | 7.53E-06 |
| MAP2K7 | AC036176.1 | 0.395766879 | 3.45E-07 |
| MAPK8 | AC036176.1 | 0.301262862 | 0.000139408 |
| MTMR14 | AC036176.1 | 0.453472878 | 3.12E-09 |
| MYC | AC036176.1 | -0.376680305 | 1.36E-06 |
| NCKAP1 | AC036176.1 | -0.363753102 | 3.28E-06 |
| NKX2-3 | AC036176.1 | 0.499000516 | 3.88E-11 |
| PEX14 | AC036176.1 | 0.409387587 | 1.23E-07 |
| PRKAB1 | AC036176.1 | 0.430042631 | 2.34E-08 |
| PTK6 | AC036176.1 | -0.308413569 | 9.45E-05 |
| RAB1A | AC036176.1 | -0.326105572 | 3.46E-05 |
| RAB24 | AC036176.1 | 0.400640014 | 2.40E-07 |
| STK11 | AC036176.1 | 0.357601776 | 4.92E-06 |
| TP53 | AC036176.1 | 0.433032713 | 1.83E-08 |
| TP73 | AC036176.1 | 0.518281158 | 4.94E-12 |
| TSC2 | AC036176.1 | 0.560464692 | 3.37E-14 |
| ULK2 | AC036176.1 | 0.347018045 | 9.71E-06 |
| USP10 | AC036176.1 | -0.394824077 | 3.70E-07 |
| WDR45 | AC036176.1 | 0.520342473 | 3.94E-12 |
| ATF4 | NIFK-AS1 | 0.300731385 | 0.000143436 |
| ATG10 | NIFK-AS1 | 0.308783049 | 9.26E-05 |
| ATG4B | NIFK-AS1 | 0.331477112 | 2.52E-05 |
| ATG4C | NIFK-AS1 | 0.397750764 | 2.98E-07 |
| ATIC | NIFK-AS1 | 0.419536239 | 5.52E-08 |
| BAK1 | NIFK-AS1 | -0.322230377 | 4.33E-05 |
| BAX | NIFK-AS1 | 0.33857422 | 1.64E-05 |
| BCL2 | NIFK-AS1 | 0.316190206 | 6.12E-05 |
| BIRC5 | NIFK-AS1 | 0.319290332 | 5.13E-05 |
| CAPN1 | NIFK-AS1 | -0.320792735 | 4.71E-05 |
| CAPN10 | NIFK-AS1 | 0.493032984 | 7.16E-11 |
| CAPN2 | NIFK-AS1 | -0.391462222 | 4.74E-07 |
| CDKN1A | NIFK-AS1 | -0.313037502 | 7.31E-05 |
| CDKN1B | NIFK-AS1 | 0.327209081 | 3.24E-05 |
| CLN3 | NIFK-AS1 | 0.312415888 | 7.57E-05 |
| CXCR4 | NIFK-AS1 | 0.348269414 | 8.97E-06 |
| HDAC6 | NIFK-AS1 | 0.509349586 | 1.31E-11 |
| ITGA3 | NIFK-AS1 | -0.4181383 | 6.17E-08 |
| ITGA6 | NIFK-AS1 | -0.34631178 | 1.01E-05 |
| ITGB1 | NIFK-AS1 | -0.338566887 | 1.64E-05 |
| ITGB4 | NIFK-AS1 | -0.38391232 | 8.17E-07 |
| MAP2K7 | NIFK-AS1 | 0.347909969 | 9.17E-06 |
| MAPK8IP1 | NIFK-AS1 | 0.352255937 | 6.96E-06 |
| MLST8 | NIFK-AS1 | 0.309476463 | 8.91E-05 |
| PARP1 | NIFK-AS1 | 0.331768969 | 2.47E-05 |
| PTK6 | NIFK-AS1 | -0.347890065 | 9.19E-06 |
| RHEB | NIFK-AS1 | 0.333989275 | 2.17E-05 |
| ULK3 | NIFK-AS1 | 0.356553163 | 5.27E-06 |
| WDR45 | NIFK-AS1 | 0.452464486 | 3.41E-09 |
| ATG4C | CNNM3-DT | 0.3255116 | 3.58E-05 |
| ATG9B | CNNM3-DT | 0.362210231 | 3.64E-06 |
| ATIC | CNNM3-DT | 0.401462926 | 2.25E-07 |
| BAK1 | CNNM3-DT | -0.352299169 | 6.94E-06 |
| CAPN10 | CNNM3-DT | 0.405534212 | 1.65E-07 |
| CDKN1B | CNNM3-DT | 0.403774415 | 1.89E-07 |
| CLN3 | CNNM3-DT | 0.308409529 | 9.45E-05 |
| HDAC6 | CNNM3-DT | 0.404617731 | 1.77E-07 |
| ITGA3 | CNNM3-DT | -0.366367001 | 2.75E-06 |
| MAP2K7 | CNNM3-DT | 0.30061061 | 0.000144366 |
| MAPK8IP1 | CNNM3-DT | 0.419749928 | 5.43E-08 |
| MLST8 | CNNM3-DT | 0.300994335 | 0.000141429 |
| MTMR14 | CNNM3-DT | 0.324027984 | 3.91E-05 |
| PRKAB1 | CNNM3-DT | 0.362812225 | 3.49E-06 |
| PRKAR1A | CNNM3-DT | 0.345495222 | 1.07E-05 |
| RAF1 | CNNM3-DT | 0.330786399 | 2.62E-05 |
| ULK3 | CNNM3-DT | 0.399110072 | 2.69E-07 |
| VAMP7 | CNNM3-DT | 0.312032278 | 7.73E-05 |
| WIPI1 | CNNM3-DT | 0.369103787 | 2.29E-06 |
| RAB1A | AC122685.1 | 0.306064495 | 0.000107508 |
| SH3GLB1 | AC122685.1 | -0.311186014 | 8.11E-05 |
| ATG16L2 | AP005482.3 | 0.450716584 | 3.98E-09 |
| ATG4C | AP005482.3 | 0.303099517 | 0.000126285 |
| ATG4D | AP005482.3 | 0.352511287 | 6.84E-06 |
| BID | AP005482.3 | 0.358868857 | 4.53E-06 |
| CAPN10 | AP005482.3 | 0.396677082 | 3.22E-07 |
| CASP3 | AP005482.3 | 0.347282298 | 9.55E-06 |
| CDKN1B | AP005482.3 | 0.400146992 | 2.49E-07 |
| CLN3 | AP005482.3 | 0.353954759 | 6.24E-06 |
| DAPK2 | AP005482.3 | 0.425216612 | 3.49E-08 |
| MLST8 | AP005482.3 | 0.371336332 | 1.97E-06 |
| PELP1 | AP005482.3 | 0.351449359 | 7.33E-06 |
| STK11 | AP005482.3 | 0.306699135 | 0.000103844 |
| ULK3 | AP005482.3 | 0.367434208 | 2.56E-06 |
| GAPDH | AC005840.2 | 0.368127598 | 2.45E-06 |
| BECN1 | AC067852.2 | 0.471599952 | 5.87E-10 |
| BIRC6 | AC067852.2 | 0.390166876 | 5.21E-07 |
| EDEM1 | AC067852.2 | 0.309124804 | 9.09E-05 |
| EIF2AK3 | AC067852.2 | 0.36438113 | 3.15E-06 |
| HGS | AC067852.2 | 0.347856823 | 9.21E-06 |
| KLHL24 | AC067852.2 | 0.305096341 | 0.000113332 |
| NBR1 | AC067852.2 | 0.484680556 | 1.65E-10 |
| PARP1 | AC067852.2 | 0.380048695 | 1.07E-06 |
| PRKCD | AC067852.2 | 0.375393751 | 1.49E-06 |
| RAF1 | AC067852.2 | 0.341521125 | 1.37E-05 |
| RPS6KB1 | AC067852.2 | 0.467953276 | 8.28E-10 |
| RPTOR | AC067852.2 | 0.336816238 | 1.83E-05 |
| SPNS1 | AC067852.2 | 0.333142612 | 2.28E-05 |
| ARSB | AC082651.3 | -0.306150942 | 0.000107002 |
| ATG4C | AC082651.3 | -0.323775392 | 3.96E-05 |
| BCL2 | AC082651.3 | -0.323428316 | 4.04E-05 |
| DRAM1 | AC082651.3 | -0.385432721 | 7.33E-07 |
| GABARAPL2 | AC082651.3 | 0.383591017 | 8.36E-07 |
| MAPK8 | AC082651.3 | -0.307520064 | 9.93E-05 |
| PARP1 | AC082651.3 | -0.356281819 | 5.37E-06 |
| TP53 | AC082651.3 | -0.327901253 | 3.11E-05 |
| ATG2A | AC009414.2 | -0.301072045 | 0.000140841 |
| IKBKE | AC009414.2 | -0.322965871 | 4.15E-05 |
| TP53 | AC009414.2 | -0.305498093 | 0.00011088 |
| ATG16L2 | NDUFV2-AS1 | 0.438886186 | 1.11E-08 |
| BID | NDUFV2-AS1 | 0.338781832 | 1.62E-05 |
| DAPK2 | NDUFV2-AS1 | 0.300610863 | 0.000144364 |
| FOXO1 | NDUFV2-AS1 | -0.34964377 | 8.22E-06 |
| MLST8 | NDUFV2-AS1 | 0.321588613 | 4.50E-05 |
| ULK3 | NDUFV2-AS1 | 0.3181901 | 5.47E-05 |
| BID | AC010980.2 | 0.374970173 | 1.53E-06 |
| ITGA3 | AC010980.2 | -0.340874825 | 1.42E-05 |
| KLHL24 | AC010980.2 | 0.307182863 | 0.000101129 |
| NFE2L2 | AC010980.2 | 0.366823725 | 2.67E-06 |
| PELP1 | AC010980.2 | 0.30254385 | 0.000130128 |
| PINK1 | AC010980.2 | -0.337147497 | 1.79E-05 |
| UVRAG | AC010980.2 | 0.339549017 | 1.54E-05 |
| VAMP3 | AC010980.2 | -0.302137311 | 0.000133009 |
| ATG9B | LINC00857 | 0.546508931 | 1.90E-13 |
| BCL2L1 | LINC00857 | 0.350013067 | 8.03E-06 |
| CAPN1 | LINC00857 | 0.306513974 | 0.0001049 |
| FKBP1B | LINC00857 | 0.367778471 | 2.50E-06 |
| GABARAP | LINC00857 | -0.311937241 | 7.78E-05 |
| PTK6 | LINC00857 | 0.332935762 | 2.31E-05 |
| RAB11A | LINC00857 | 0.335828286 | 1.94E-05 |
| RAB1A | LINC00857 | 0.332715625 | 2.34E-05 |
| TP53INP2 | LINC00857 | 0.494040176 | 6.46E-11 |
| ATG10 | LINC00944 | 0.36566159 | 2.89E-06 |
| ATG12 | LINC00944 | 0.351544558 | 7.28E-06 |
| ATG4A | LINC00944 | 0.368080893 | 2.45E-06 |
| ATG4B | LINC00944 | 0.312518586 | 7.53E-05 |
| ATG4C | LINC00944 | 0.385090958 | 7.51E-07 |
| ATG7 | LINC00944 | 0.394620166 | 3.76E-07 |
| BCL2 | LINC00944 | 0.364521787 | 3.12E-06 |
| BNIP1 | LINC00944 | 0.360516284 | 4.07E-06 |
| CALCOCO2 | LINC00944 | 0.403833275 | 1.88E-07 |
| CDKN2A | LINC00944 | 0.350071294 | 8.00E-06 |
| CFLAR | LINC00944 | 0.321291376 | 4.58E-05 |
| CXCR4 | LINC00944 | 0.405617641 | 1.64E-07 |
| DRAM1 | LINC00944 | 0.391273673 | 4.80E-07 |
| EEF2K | LINC00944 | 0.304859019 | 0.000114804 |
| FAS | LINC00944 | 0.53622808 | 6.44E-13 |
| HDAC6 | LINC00944 | 0.34369121 | 1.20E-05 |
| HIF1A | LINC00944 | -0.306320676 | 0.000106015 |
| IFNG | LINC00944 | 0.348582121 | 8.79E-06 |
| IKBKE | LINC00944 | 0.360400898 | 4.10E-06 |
| MAPK8 | LINC00944 | 0.399777715 | 2.56E-07 |
| MTMR14 | LINC00944 | 0.361179094 | 3.89E-06 |
| NCKAP1 | LINC00944 | -0.354761813 | 5.92E-06 |
| NKX2-3 | LINC00944 | 0.491738098 | 8.16E-11 |
| PEX14 | LINC00944 | 0.445238772 | 6.44E-09 |
| RAB24 | LINC00944 | 0.341937298 | 1.33E-05 |
| STK11 | LINC00944 | 0.300337302 | 0.000146492 |
| TP53 | LINC00944 | 0.39870649 | 2.77E-07 |
| TP73 | LINC00944 | 0.602658377 | 1.07E-16 |
| TSC2 | LINC00944 | 0.586309403 | 1.10E-15 |
| WDR45 | LINC00944 | 0.330256507 | 2.71E-05 |
| ATG12 | LINC01355 | 0.420945193 | 4.93E-08 |
| ATG16L2 | LINC01355 | 0.571711039 | 7.88E-15 |
| ATG4B | LINC01355 | 0.408063615 | 1.36E-07 |
| ATG4C | LINC01355 | 0.459264526 | 1.85E-09 |
| CAPN10 | LINC01355 | 0.575533889 | 4.75E-15 |
| CDKN1B | LINC01355 | 0.304431449 | 0.0001175 |
| CFLAR | LINC01355 | 0.338865482 | 1.61E-05 |
| CXCR4 | LINC01355 | 0.477918282 | 3.20E-10 |
| DAPK2 | LINC01355 | 0.473307745 | 4.99E-10 |
| HDAC1 | LINC01355 | 0.306715573 | 0.00010375 |
| HDAC6 | LINC01355 | 0.43824057 | 1.18E-08 |
| IKBKB | LINC01355 | 0.460911531 | 1.59E-09 |
| MAP2K7 | LINC01355 | 0.368201398 | 2.43E-06 |
| MAPK8 | LINC01355 | 0.338684603 | 1.63E-05 |
| MTMR14 | LINC01355 | 0.315254613 | 6.46E-05 |
| NKX2-3 | LINC01355 | 0.302760171 | 0.000128619 |
| PEX14 | LINC01355 | 0.370313562 | 2.11E-06 |
| RAB24 | LINC01355 | 0.337846239 | 1.71E-05 |
| RAF1 | LINC01355 | 0.352201205 | 6.98E-06 |
| STK11 | LINC01355 | 0.363749736 | 3.28E-06 |
| TP73 | LINC01355 | 0.340753811 | 1.43E-05 |
| TSC2 | LINC01355 | 0.457518976 | 2.16E-09 |
| ULK3 | LINC01355 | 0.509175044 | 1.33E-11 |
| WDR45 | LINC01355 | 0.376447695 | 1.38E-06 |
| BID | LINC01836 | 0.393669817 | 4.03E-07 |
| AMBRA1 | AC037459.3 | 0.392011732 | 4.55E-07 |
| ARNT | AC037459.3 | 0.385439935 | 7.32E-07 |
| ARSB | AC037459.3 | 0.412663742 | 9.51E-08 |
| ATG4C | AC037459.3 | 0.35318991 | 6.55E-06 |
| BCL2 | AC037459.3 | 0.366595438 | 2.71E-06 |
| BNIP3L | AC037459.3 | 0.317263685 | 5.76E-05 |
| CXCR4 | AC037459.3 | 0.44498443 | 6.59E-09 |
| EEF2K | AC037459.3 | 0.366670732 | 2.70E-06 |
| EIF2AK3 | AC037459.3 | 0.369985703 | 2.16E-06 |
| HDAC6 | AC037459.3 | 0.429144969 | 2.52E-08 |
| IKBKB | AC037459.3 | 0.420152919 | 5.25E-08 |
| IKBKE | AC037459.3 | 0.301206362 | 0.000139831 |
| MAP2K7 | AC037459.3 | 0.356050632 | 5.45E-06 |
| MAPK8 | AC037459.3 | 0.310927717 | 8.23E-05 |
| MTOR | AC037459.3 | 0.332441545 | 2.38E-05 |
| NBR1 | AC037459.3 | 0.331954536 | 2.45E-05 |
| NKX2-3 | AC037459.3 | 0.305194175 | 0.00011273 |
| PARP1 | AC037459.3 | 0.343805175 | 1.19E-05 |
| PIK3C3 | AC037459.3 | 0.336916802 | 1.81E-05 |
| PIK3R4 | AC037459.3 | 0.4314474 | 2.09E-08 |
| RAF1 | AC037459.3 | 0.455117344 | 2.69E-09 |
| RB1CC1 | AC037459.3 | 0.300586117 | 0.000144555 |
| SIRT1 | AC037459.3 | 0.342441333 | 1.29E-05 |
| TP53 | AC037459.3 | 0.372412105 | 1.83E-06 |
| TP73 | AC037459.3 | 0.384026988 | 8.10E-07 |
| TSC1 | AC037459.3 | 0.53397431 | 8.38E-13 |
| TSC2 | AC037459.3 | 0.471510114 | 5.92E-10 |
| ULK2 | AC037459.3 | 0.374783133 | 1.55E-06 |
| UVRAG | AC037459.3 | 0.352718788 | 6.75E-06 |
| ARNT | AC010186.3 | 0.338781287 | 1.62E-05 |
| ARSB | AC010186.3 | 0.431102198 | 2.15E-08 |
| ATG4C | AC010186.3 | 0.350854907 | 7.61E-06 |
| BCL2 | AC010186.3 | 0.422775917 | 4.25E-08 |
| BIRC6 | AC010186.3 | 0.36294182 | 3.46E-06 |
| BNIP3 | AC010186.3 | -0.360074871 | 4.19E-06 |
| CAMKK2 | AC010186.3 | 0.32793137 | 3.11E-05 |
| CASP3 | AC010186.3 | 0.35925265 | 4.42E-06 |
| CCR2 | AC010186.3 | 0.311276779 | 8.07E-05 |
| CD46 | AC010186.3 | 0.430599905 | 2.24E-08 |
| CDKN1B | AC010186.3 | 0.585891866 | 1.16E-15 |
| CFLAR | AC010186.3 | 0.350449227 | 7.81E-06 |
| CX3CL1 | AC010186.3 | 0.391238219 | 4.81E-07 |
| CXCR4 | AC010186.3 | 0.48485273 | 1.63E-10 |
| DRAM1 | AC010186.3 | 0.321879243 | 4.42E-05 |
| EEF2K | AC010186.3 | 0.360824023 | 3.98E-06 |
| EIF2AK3 | AC010186.3 | 0.35117989 | 7.45E-06 |
| FAS | AC010186.3 | 0.437063785 | 1.30E-08 |
| HDAC1 | AC010186.3 | 0.331649582 | 2.49E-05 |
| HDAC6 | AC010186.3 | 0.564156968 | 2.10E-14 |
| HSPA5 | AC010186.3 | -0.348994876 | 8.57E-06 |
| IKBKB | AC010186.3 | 0.435697241 | 1.46E-08 |
| IKBKE | AC010186.3 | 0.548096783 | 1.56E-13 |
| ITGA3 | AC010186.3 | -0.304608405 | 0.000116377 |
| ITPR1 | AC010186.3 | 0.340427092 | 1.46E-05 |
| KLHL24 | AC010186.3 | 0.336636136 | 1.85E-05 |
| MAP2K7 | AC010186.3 | 0.364167627 | 3.19E-06 |
| MAPK8 | AC010186.3 | 0.457150292 | 2.24E-09 |
| MTMR14 | AC010186.3 | 0.347087134 | 9.66E-06 |
| MTOR | AC010186.3 | 0.373280769 | 1.72E-06 |
| NBR1 | AC010186.3 | 0.390675865 | 5.02E-07 |
| NKX2-3 | AC010186.3 | 0.416626489 | 6.96E-08 |
| PARP1 | AC010186.3 | 0.430680648 | 2.22E-08 |
| PIK3R4 | AC010186.3 | 0.339953937 | 1.51E-05 |
| PRKAB1 | AC010186.3 | 0.474110804 | 4.62E-10 |
| PTK6 | AC010186.3 | -0.331692321 | 2.49E-05 |
| RAF1 | AC010186.3 | 0.352506313 | 6.85E-06 |
| SH3GLB1 | AC010186.3 | 0.342655194 | 1.27E-05 |
| SIRT1 | AC010186.3 | 0.33585672 | 1.93E-05 |
| TP53 | AC010186.3 | 0.542667092 | 3.01E-13 |
| TP73 | AC010186.3 | 0.43325013 | 1.79E-08 |
| TSC2 | AC010186.3 | 0.496283787 | 5.14E-11 |
| ULK2 | AC010186.3 | 0.318990893 | 5.22E-05 |
| AMBRA1 | AC099850.3 | 0.319884329 | 4.96E-05 |
| ARNT | AC099850.3 | 0.310325806 | 8.50E-05 |
| ARSB | AC099850.3 | 0.305409058 | 0.000111419 |
| ATF4 | AC099850.3 | -0.306354747 | 0.000105817 |
| ATG2B | AC099850.3 | 0.300680475 | 0.000143827 |
| BECN1 | AC099850.3 | 0.30479843 | 0.000115182 |
| BIRC5 | AC099850.3 | 0.327294203 | 3.23E-05 |
| BIRC6 | AC099850.3 | 0.383446027 | 8.45E-07 |
| CALCOCO2 | AC099850.3 | 0.381025113 | 1.00E-06 |
| CANX | AC099850.3 | 0.39979885 | 2.55E-07 |
| EDEM1 | AC099850.3 | 0.417985806 | 6.25E-08 |
| FAS | AC099850.3 | 0.307426041 | 9.98E-05 |
| GOPC | AC099850.3 | 0.429102218 | 2.53E-08 |
| ITGA3 | AC099850.3 | 0.303457346 | 0.000123867 |
| MAPK1 | AC099850.3 | 0.324710254 | 3.75E-05 |
| MAPK8 | AC099850.3 | 0.338059529 | 1.69E-05 |
| MAPK9 | AC099850.3 | 0.373617635 | 1.68E-06 |
| MTOR | AC099850.3 | 0.445261274 | 6.43E-09 |
| NAF1 | AC099850.3 | 0.306885864 | 0.000102788 |
| NBR1 | AC099850.3 | 0.333087143 | 2.29E-05 |
| NFKB1 | AC099850.3 | 0.377508984 | 1.28E-06 |
| PARP1 | AC099850.3 | 0.401466458 | 2.25E-07 |
| PIK3R4 | AC099850.3 | 0.300964729 | 0.000141654 |
| PRKAR1A | AC099850.3 | 0.319962425 | 4.94E-05 |
| PTEN | AC099850.3 | 0.384761271 | 7.69E-07 |
| RAB24 | AC099850.3 | -0.323426175 | 4.04E-05 |
| RB1CC1 | AC099850.3 | 0.304865828 | 0.000114761 |
| RPS6KB1 | AC099850.3 | 0.424874245 | 3.58E-08 |
| RPTOR | AC099850.3 | 0.362374791 | 3.60E-06 |
| SIRT1 | AC099850.3 | 0.369668334 | 2.20E-06 |
| USP10 | AC099850.3 | 0.362038282 | 3.68E-06 |
| VAMP7 | AC099850.3 | 0.339748225 | 1.53E-05 |
| BCL2L1 | MAN1B1-DT | 0.320498264 | 4.79E-05 |
| ATG16L2 | AC007383.2 | 0.309772331 | 8.77E-05 |
| ATIC | AC007383.2 | 0.401220088 | 2.29E-07 |
| CAPN10 | AC007383.2 | 0.364748492 | 3.07E-06 |
| DIRAS3 | AC007383.2 | 0.352442384 | 6.87E-06 |
| GABARAPL2 | AC007383.2 | 0.446926027 | 5.56E-09 |
| MAPK8IP1 | AC007383.2 | 0.402688085 | 2.05E-07 |
| PRKAR1A | AC007383.2 | 0.316901664 | 5.88E-05 |
| ATG4C | LINC02541 | -0.301955637 | 0.000134315 |
| MTMR14 | LINC02541 | -0.314711472 | 6.66E-05 |
| TP63 | LINC02541 | 0.422222953 | 4.45E-08 |
| ATG16L2 | ZFPM2-AS1 | 0.310086205 | 8.62E-05 |
| ATG16L2 | AC074117.1 | 0.361302146 | 3.86E-06 |
| ATG9B | AC074117.1 | 0.373021927 | 1.75E-06 |
| BECN1 | AC074117.1 | 0.320594332 | 4.76E-05 |
| CAPN10 | AC074117.1 | 0.34248859 | 1.29E-05 |
| EIF2AK3 | AC074117.1 | 0.398150067 | 2.89E-07 |
| GABARAPL1 | AC074117.1 | 0.320474855 | 4.80E-05 |
| ITGA3 | AC074117.1 | -0.381533962 | 9.67E-07 |
| NFE2L2 | AC074117.1 | 0.366148719 | 2.79E-06 |
| PINK1 | AC074117.1 | -0.319799173 | 4.99E-05 |
| TP53INP2 | AC074117.1 | 0.304936551 | 0.000114321 |
| ULK3 | AC074117.1 | 0.349621944 | 8.23E-06 |
| WIPI1 | AC074117.1 | 0.340592837 | 1.45E-05 |
| FKBP1B | PROSER2-AS1 | 0.362051938 | 3.67E-06 |
| ITGA6 | PROSER2-AS1 | 0.302993365 | 0.000127011 |
| ITGB1 | PROSER2-AS1 | 0.513930571 | 7.96E-12 |
| ARNT | EBLN3P | 0.403932828 | 1.87E-07 |
| BCL2 | EBLN3P | 0.372378548 | 1.83E-06 |
| BIRC6 | EBLN3P | 0.368378301 | 2.40E-06 |
| CD46 | EBLN3P | 0.499544652 | 3.67E-11 |
| CDKN1B | EBLN3P | 0.39996492 | 2.52E-07 |
| EEF2 | EBLN3P | 0.363957728 | 3.24E-06 |
| EEF2K | EBLN3P | 0.321710147 | 4.47E-05 |
| EIF2AK2 | EBLN3P | 0.309389963 | 8.96E-05 |
| EIF2AK3 | EBLN3P | 0.308786532 | 9.26E-05 |
| ERN1 | EBLN3P | 0.300952815 | 0.000141744 |
| FAS | EBLN3P | 0.349662726 | 8.21E-06 |
| HDAC6 | EBLN3P | 0.354118604 | 6.17E-06 |
| ITPR1 | EBLN3P | 0.308650582 | 9.33E-05 |
| KLHL24 | EBLN3P | 0.419144567 | 5.69E-08 |
| MAP2K7 | EBLN3P | 0.389034464 | 5.65E-07 |
| MAPK9 | EBLN3P | 0.343792088 | 1.19E-05 |
| MTOR | EBLN3P | 0.330008567 | 2.75E-05 |
| NAF1 | EBLN3P | 0.342570916 | 1.28E-05 |
| NBR1 | EBLN3P | 0.49602939 | 5.27E-11 |
| PARP1 | EBLN3P | 0.442715006 | 8.02E-09 |
| PIK3R4 | EBLN3P | 0.306705535 | 0.000103807 |
| RAB33B | EBLN3P | 0.332013675 | 2.44E-05 |
| RAF1 | EBLN3P | 0.389411168 | 5.50E-07 |
| RB1CC1 | EBLN3P | 0.492685583 | 7.42E-11 |
| RPS6KB1 | EBLN3P | 0.383311021 | 8.53E-07 |
| SIRT1 | EBLN3P | 0.314364622 | 6.79E-05 |
| SQSTM1 | EBLN3P | 0.372842484 | 1.77E-06 |
| TP53 | EBLN3P | 0.341452114 | 1.37E-05 |
| TSC1 | EBLN3P | 0.430813631 | 2.20E-08 |
| ULK2 | EBLN3P | 0.399526116 | 2.61E-07 |
| BCL2 | MIF-AS1 | 0.355140638 | 5.78E-06 |
| CDKN1B | MIF-AS1 | 0.369074646 | 2.29E-06 |
| EEF2K | MIF-AS1 | 0.339744482 | 1.53E-05 |
| HDAC6 | MIF-AS1 | 0.386901794 | 6.59E-07 |
| IFNG | MIF-AS1 | 0.308236495 | 9.54E-05 |
| MAP2K7 | MIF-AS1 | 0.353999063 | 6.22E-06 |
| MAPK8 | MIF-AS1 | 0.321202402 | 4.60E-05 |
| NKX2-3 | MIF-AS1 | 0.46927037 | 7.31E-10 |
| PARP1 | MIF-AS1 | 0.323471479 | 4.03E-05 |
| PRKAB1 | MIF-AS1 | 0.310489023 | 8.43E-05 |
| TP53 | MIF-AS1 | 0.371616981 | 1.93E-06 |
| TP73 | MIF-AS1 | 0.431287369 | 2.11E-08 |
| TSC2 | MIF-AS1 | 0.36729625 | 2.59E-06 |
| ARNT | AP003068.1 | -0.372732879 | 1.79E-06 |
| ARSB | AP003068.1 | -0.370522594 | 2.08E-06 |
| ATF6 | AP003068.1 | -0.419562285 | 5.51E-08 |
| ATG2B | AP003068.1 | -0.321147781 | 4.61E-05 |
| BECN1 | AP003068.1 | -0.403662591 | 1.91E-07 |
| BIRC6 | AP003068.1 | -0.356657184 | 5.24E-06 |
| CANX | AP003068.1 | -0.359544937 | 4.33E-06 |
| CD46 | AP003068.1 | -0.409383232 | 1.23E-07 |
| EEF2K | AP003068.1 | -0.342083249 | 1.32E-05 |
| EIF2AK3 | AP003068.1 | -0.370912685 | 2.02E-06 |
| EIF4EBP1 | AP003068.1 | 0.327586752 | 3.17E-05 |
| HDAC6 | AP003068.1 | -0.349292236 | 8.41E-06 |
| ITPR1 | AP003068.1 | -0.305327826 | 0.000111913 |
| KLHL24 | AP003068.1 | -0.374589698 | 1.57E-06 |
| MAPK1 | AP003068.1 | -0.348348358 | 8.92E-06 |
| MAPK8 | AP003068.1 | -0.343737154 | 1.19E-05 |
| MAPK9 | AP003068.1 | -0.35893689 | 4.51E-06 |
| MBTPS2 | AP003068.1 | -0.302550905 | 0.000130079 |
| MTOR | AP003068.1 | -0.349753502 | 8.16E-06 |
| NAF1 | AP003068.1 | -0.332101577 | 2.43E-05 |
| NBR1 | AP003068.1 | -0.414012001 | 8.56E-08 |
| NCKAP1 | AP003068.1 | -0.349200471 | 8.45E-06 |
| NPC1 | AP003068.1 | -0.308174551 | 9.58E-05 |
| PIK3R4 | AP003068.1 | -0.414275508 | 8.38E-08 |
| RAB33B | AP003068.1 | -0.386700704 | 6.69E-07 |
| RB1CC1 | AP003068.1 | -0.360917872 | 3.96E-06 |
| RPS6KB1 | AP003068.1 | -0.310428906 | 8.46E-05 |
| SIRT1 | AP003068.1 | -0.33061779 | 2.65E-05 |
| VAMP7 | AP003068.1 | -0.329526949 | 2.83E-05 |
| ATG10 | AC124016.2 | 0.380761846 | 1.02E-06 |
| ATG16L2 | AC124016.2 | 0.300875915 | 0.00014233 |
| ATG4B | AC124016.2 | 0.539301089 | 4.49E-13 |
| ATG4C | AC124016.2 | 0.352556336 | 6.82E-06 |
| ATG4D | AC124016.2 | 0.351962905 | 7.09E-06 |
| BAG3 | AC124016.2 | -0.334364232 | 2.12E-05 |
| BAX | AC124016.2 | 0.520499261 | 3.87E-12 |
| BIRC5 | AC124016.2 | 0.321218306 | 4.60E-05 |
| BNIP1 | AC124016.2 | 0.450458262 | 4.07E-09 |
| CAPN10 | AC124016.2 | 0.566273419 | 1.60E-14 |
| CAPNS1 | AC124016.2 | 0.331779917 | 2.47E-05 |
| CDKN2A | AC124016.2 | 0.423179888 | 4.11E-08 |
| CHMP4B | AC124016.2 | 0.306470115 | 0.000105152 |
| CXCR4 | AC124016.2 | 0.445297119 | 6.41E-09 |
| DAPK2 | AC124016.2 | 0.401634155 | 2.22E-07 |
| EIF4G1 | AC124016.2 | -0.398403098 | 2.83E-07 |
| GABARAP | AC124016.2 | 0.339640643 | 1.54E-05 |
| HDAC6 | AC124016.2 | 0.421991128 | 4.53E-08 |
| HIF1A | AC124016.2 | -0.358094842 | 4.77E-06 |
| KIF5B | AC124016.2 | -0.30096625 | 0.000141642 |
| MAP1LC3A | AC124016.2 | 0.300935937 | 0.000141873 |
| MAP2K7 | AC124016.2 | 0.359035789 | 4.48E-06 |
| MAPK1 | AC124016.2 | -0.31702276 | 5.84E-05 |
| MTMR14 | AC124016.2 | 0.458635799 | 1.96E-09 |
| NCKAP1 | AC124016.2 | -0.441313597 | 9.05E-09 |
| NKX2-3 | AC124016.2 | 0.350342909 | 7.86E-06 |
| PEX14 | AC124016.2 | 0.426068329 | 3.25E-08 |
| PINK1 | AC124016.2 | -0.347155082 | 9.62E-06 |
| PRKAB1 | AC124016.2 | 0.342673663 | 1.27E-05 |
| RAB24 | AC124016.2 | 0.423090866 | 4.14E-08 |
| STK11 | AC124016.2 | 0.521586819 | 3.43E-12 |
| TP53 | AC124016.2 | 0.317935984 | 5.55E-05 |
| TP63 | AC124016.2 | -0.310140408 | 8.59E-05 |
| TP73 | AC124016.2 | 0.36208041 | 3.67E-06 |
| TSC2 | AC124016.2 | 0.421602804 | 4.67E-08 |
| ULK3 | AC124016.2 | 0.341877833 | 1.34E-05 |
| USP10 | AC124016.2 | -0.342933322 | 1.25E-05 |
| WDFY3 | AC124016.2 | -0.306074439 | 0.00010745 |
| WDR45 | AC124016.2 | 0.411213831 | 1.07E-07 |
| ARNT | SNHG8 | -0.341203423 | 1.39E-05 |
| ATF4 | SNHG8 | 0.503327811 | 2.47E-11 |
| ATG16L2 | SNHG8 | 0.316233682 | 6.11E-05 |
| ATG2B | SNHG8 | -0.36470392 | 3.08E-06 |
| ATG4B | SNHG8 | 0.469101686 | 7.43E-10 |
| ATIC | SNHG8 | 0.35860837 | 4.61E-06 |
| BAG1 | SNHG8 | 0.344274092 | 1.15E-05 |
| BAG3 | SNHG8 | -0.37655401 | 1.37E-06 |
| BAX | SNHG8 | 0.490791811 | 8.98E-11 |
| BIRC5 | SNHG8 | 0.387225419 | 6.44E-07 |
| BIRC6 | SNHG8 | -0.406710852 | 1.51E-07 |
| BNIP1 | SNHG8 | 0.573775256 | 6.00E-15 |
| CAPN10 | SNHG8 | 0.50402203 | 2.30E-11 |
| CAPN2 | SNHG8 | -0.369187726 | 2.28E-06 |
| DDIT3 | SNHG8 | 0.325571689 | 3.57E-05 |
| DIRAS3 | SNHG8 | 0.32292933 | 4.16E-05 |
| EDEM1 | SNHG8 | -0.30157084 | 0.000137122 |
| EEF2K | SNHG8 | -0.305966646 | 0.000108084 |
| EIF2AK3 | SNHG8 | -0.332546207 | 2.36E-05 |
| EIF4G1 | SNHG8 | -0.42297574 | 4.18E-08 |
| FKBP1A | SNHG8 | 0.385845201 | 7.11E-07 |
| FOXO1 | SNHG8 | -0.324840518 | 3.73E-05 |
| GABARAPL2 | SNHG8 | 0.331642105 | 2.49E-05 |
| GOPC | SNHG8 | -0.332975593 | 2.30E-05 |
| HGS | SNHG8 | -0.328333455 | 3.03E-05 |
| HIF1A | SNHG8 | -0.357634272 | 4.91E-06 |
| ITGA6 | SNHG8 | -0.347652843 | 9.33E-06 |
| ITGB1 | SNHG8 | -0.318999984 | 5.22E-05 |
| KIF5B | SNHG8 | -0.338210987 | 1.68E-05 |
| LAMP2 | SNHG8 | -0.379369711 | 1.13E-06 |
| MAP1LC3A | SNHG8 | 0.43453252 | 1.61E-08 |
| MAPK1 | SNHG8 | -0.515337635 | 6.83E-12 |
| MLST8 | SNHG8 | 0.373124339 | 1.74E-06 |
| NCKAP1 | SNHG8 | -0.438220716 | 1.18E-08 |
| NPC1 | SNHG8 | -0.323969292 | 3.92E-05 |
| PIK3R4 | SNHG8 | -0.332974385 | 2.30E-05 |
| RAB24 | SNHG8 | 0.414959962 | 7.94E-08 |
| RB1 | SNHG8 | -0.368497333 | 2.39E-06 |
| SPNS1 | SNHG8 | -0.308369094 | 9.48E-05 |
| TM9SF1 | SNHG8 | -0.305360855 | 0.000111712 |
| TP63 | SNHG8 | -0.380323434 | 1.05E-06 |
| USP10 | SNHG8 | -0.379391052 | 1.12E-06 |
| WDFY3 | SNHG8 | -0.388853924 | 5.73E-07 |
| WDR45 | SNHG8 | 0.477714764 | 3.27E-10 |
| ZFYVE1 | SNHG8 | -0.35582267 | 5.53E-06 |
| ATG16L2 | AC018645.2 | 0.311786503 | 7.84E-05 |
| ATIC | AC018645.2 | 0.330236785 | 2.71E-05 |
| BAK1 | AC018645.2 | -0.321731084 | 4.46E-05 |
| BID | AC018645.2 | 0.354879635 | 5.88E-06 |
| CAPN10 | AC018645.2 | 0.353275986 | 6.52E-06 |
| DIRAS3 | AC018645.2 | 0.307953284 | 9.69E-05 |
| FKBP1B | AC018645.2 | 0.392930544 | 4.25E-07 |
| MAPK8IP1 | AC018645.2 | 0.322380305 | 4.30E-05 |
| ATG4C | LINC01857 | 0.346843066 | 9.81E-06 |
| ATG7 | LINC01857 | 0.334690427 | 2.08E-05 |
| BCL2 | LINC01857 | 0.352043572 | 7.05E-06 |
| CAPNS1 | LINC01857 | 0.318085374 | 5.50E-05 |
| CCR2 | LINC01857 | 0.580941465 | 2.29E-15 |
| CTSD | LINC01857 | 0.337564976 | 1.74E-05 |
| CXCR4 | LINC01857 | 0.521775865 | 3.36E-12 |
| DRAM1 | LINC01857 | 0.347454316 | 9.44E-06 |
| GABARAP | LINC01857 | 0.324726456 | 3.75E-05 |
| IFNG | LINC01857 | 0.393070725 | 4.21E-07 |
| IKBKE | LINC01857 | 0.300627911 | 0.000144232 |
| ITPR1 | LINC01857 | 0.434687309 | 1.59E-08 |
| MTMR14 | LINC01857 | 0.48431332 | 1.72E-10 |
| PRKCD | LINC01857 | 0.349905883 | 8.08E-06 |
| RGS19 | LINC01857 | 0.459796557 | 1.76E-09 |
| ATG3 | AC019080.1 | 0.360045566 | 4.19E-06 |
| ATG4D | AC019080.1 | 0.349366347 | 8.37E-06 |
| EIF2AK3 | AC019080.1 | 0.474643134 | 4.39E-10 |
| FOXO3 | AC019080.1 | 0.360136781 | 4.17E-06 |
| GABARAPL1 | AC019080.1 | 0.402298887 | 2.11E-07 |
| ITGA3 | AC019080.1 | -0.375081937 | 1.52E-06 |
| KLHL24 | AC019080.1 | 0.406048851 | 1.59E-07 |
| MAP2K7 | AC019080.1 | 0.304928853 | 0.000114369 |
| NFE2L2 | AC019080.1 | 0.729817566 | 4.70E-27 |
| PIK3R4 | AC019080.1 | 0.309333464 | 8.98E-05 |
| PINK1 | AC019080.1 | -0.321372851 | 4.55E-05 |
| RB1CC1 | AC019080.1 | 0.322446251 | 4.28E-05 |
| RHEB | AC019080.1 | 0.325681328 | 3.55E-05 |
| TP63 | AC019080.1 | 0.463296011 | 1.28E-09 |
| VAMP3 | AC019080.1 | -0.301566719 | 0.000137152 |
| ATG2B | POU6F2-AS2 | 0.335386302 | 1.99E-05 |
| HIF1A | POU6F2-AS2 | 0.355305148 | 5.72E-06 |
| NCKAP1 | POU6F2-AS2 | 0.468599584 | 7.79E-10 |
| TP53 | POU6F2-AS2 | -0.312573203 | 7.50E-05 |
| TP63 | POU6F2-AS2 | 0.308857379 | 9.22E-05 |
| ARNT | LINC01214 | -0.301702462 | 0.000136156 |
| ARSB | LINC01214 | -0.306573448 | 0.00010456 |
| BIRC6 | LINC01214 | -0.326125025 | 3.46E-05 |
| BNIP3 | LINC01214 | 0.358520346 | 4.64E-06 |
| CAMKK2 | LINC01214 | -0.354749407 | 5.93E-06 |
| CD46 | LINC01214 | -0.350260924 | 7.90E-06 |
| EEF2K | LINC01214 | -0.302768247 | 0.000128563 |
| MAPK3 | LINC01214 | 0.415087275 | 7.86E-08 |
| MAPK9 | LINC01214 | -0.308528516 | 9.39E-05 |
| NAF1 | LINC01214 | -0.302930779 | 0.000127441 |
| NBR1 | LINC01214 | -0.329407078 | 2.85E-05 |
| PARP1 | LINC01214 | -0.427239477 | 2.95E-08 |
| PIK3R4 | LINC01214 | -0.317302038 | 5.75E-05 |
| PTK6 | LINC01214 | 0.415589966 | 7.56E-08 |
| RAF1 | LINC01214 | -0.322279235 | 4.32E-05 |
| RHEB | LINC01214 | -0.319537488 | 5.06E-05 |
| TP53 | LINC01214 | -0.302123671 | 0.000133107 |
| USP10 | LINC01214 | -0.30839836 | 9.46E-05 |
| ATF4 | AC078778.1 | 0.391555643 | 4.70E-07 |
| ATG16L2 | AC078778.1 | 0.409653911 | 1.20E-07 |
| ATG3 | AC078778.1 | 0.366685167 | 2.70E-06 |
| ATG4B | AC078778.1 | 0.481880602 | 2.18E-10 |
| ATG4D | AC078778.1 | 0.414480828 | 8.25E-08 |
| BAG1 | AC078778.1 | 0.376409826 | 1.39E-06 |
| BAG3 | AC078778.1 | -0.300357107 | 0.000146337 |
| BAX | AC078778.1 | 0.538394539 | 5.00E-13 |
| BIRC5 | AC078778.1 | 0.490879028 | 8.91E-11 |
| BNIP1 | AC078778.1 | 0.546962925 | 1.79E-13 |
| CAPN10 | AC078778.1 | 0.561157532 | 3.08E-14 |
| CDKN1B | AC078778.1 | 0.300537428 | 0.000144932 |
| CDKN2A | AC078778.1 | 0.443789134 | 7.31E-09 |
| CLN3 | AC078778.1 | 0.365619426 | 2.90E-06 |
| CXCR4 | AC078778.1 | 0.387854799 | 6.16E-07 |
| DAPK2 | AC078778.1 | 0.334570485 | 2.09E-05 |
| EIF4G1 | AC078778.1 | -0.36749649 | 2.55E-06 |
| FKBP1A | AC078778.1 | 0.422900367 | 4.21E-08 |
| GABARAP | AC078778.1 | 0.332357559 | 2.39E-05 |
| HDAC6 | AC078778.1 | 0.366693514 | 2.69E-06 |
| HIF1A | AC078778.1 | -0.324969518 | 3.70E-05 |
| IKBKE | AC078778.1 | 0.303896917 | 0.000120955 |
| ITGB1 | AC078778.1 | -0.317623535 | 5.65E-05 |
| KIF5B | AC078778.1 | -0.306542437 | 0.000104737 |
| MAP1LC3B | AC078778.1 | -0.301120369 | 0.000140477 |
| MAP2K7 | AC078778.1 | 0.350176755 | 7.95E-06 |
| MAPK1 | AC078778.1 | -0.335969312 | 1.92E-05 |
| MLST8 | AC078778.1 | 0.329002863 | 2.92E-05 |
| MTMR14 | AC078778.1 | 0.381047594 | 1.00E-06 |
| NCKAP1 | AC078778.1 | -0.371296834 | 1.97E-06 |
| NKX2-3 | AC078778.1 | 0.35668653 | 5.23E-06 |
| PELP1 | AC078778.1 | 0.372868581 | 1.77E-06 |
| PEX14 | AC078778.1 | 0.323321636 | 4.07E-05 |
| PINK1 | AC078778.1 | -0.361421883 | 3.83E-06 |
| PRKAB1 | AC078778.1 | 0.327338804 | 3.22E-05 |
| RAB24 | AC078778.1 | 0.413558164 | 8.87E-08 |
| STK11 | AC078778.1 | 0.372691242 | 1.79E-06 |
| ULK3 | AC078778.1 | 0.314746624 | 6.64E-05 |
| USP10 | AC078778.1 | -0.346239654 | 1.02E-05 |
| WDFY3 | AC078778.1 | -0.354697953 | 5.95E-06 |
| WDR45 | AC078778.1 | 0.672900225 | 8.83E-22 |
| BID | AC034206.1 | 0.332782845 | 2.33E-05 |
| DIRAS3 | AC034206.1 | 0.409185469 | 1.25E-07 |
| FKBP1B | AC034206.1 | 0.335872887 | 1.93E-05 |
| FOXO1 | AC034206.1 | -0.300136961 | 0.000148069 |
| PRKCQ | AC034206.1 | 0.301184385 | 0.000139996 |
| BAX | AC009283.1 | 0.348791411 | 8.68E-06 |
| CXCR4 | AC009283.1 | 0.381432965 | 9.74E-07 |
| ERBB2 | AC009283.1 | 0.34004217 | 1.50E-05 |
| GABARAPL1 | AC012368.1 | 0.439165271 | 1.09E-08 |
| NFE2L2 | AC012368.1 | 0.377641727 | 1.27E-06 |
| PIK3C3 | AC012368.1 | 0.307558256 | 9.91E-05 |
| UVRAG | AC012368.1 | 0.346575965 | 9.98E-06 |
| ATG4C | AL031985.3 | 0.368932067 | 2.32E-06 |
| CAMKK2 | AL031985.3 | 0.305520995 | 0.000110742 |
| DAPK1 | AL031985.3 | 0.353638024 | 6.37E-06 |
| EEF2 | AL031985.3 | 0.361737218 | 3.75E-06 |
| HDAC6 | AL031985.3 | 0.307726449 | 9.82E-05 |
| PARP1 | AL031985.3 | 0.357667429 | 4.90E-06 |
| SIRT1 | AL031985.3 | 0.31871601 | 5.30E-05 |
| ST13 | AL031985.3 | 0.363631127 | 3.31E-06 |
| ULK2 | AL031985.3 | 0.329078097 | 2.90E-05 |
| UVRAG | AL031985.3 | 0.472758874 | 5.26E-10 |
| NAMPT | AC009118.2 | 0.371398173 | 1.96E-06 |
| SIRT2 | AC009118.2 | 0.345078817 | 1.10E-05 |
| WIPI1 | AC009118.2 | 0.370436147 | 2.09E-06 |
| ATG10 | AC091982.3 | 0.446436393 | 5.80E-09 |
| ATG4C | AC091982.3 | 0.36035978 | 4.11E-06 |
| ATG4D | AC091982.3 | 0.314237595 | 6.84E-05 |
| BAK1 | AC091982.3 | -0.306444457 | 0.0001053 |
| BAX | AC091982.3 | 0.320605733 | 4.76E-05 |
| BCL2 | AC091982.3 | 0.30390572 | 0.000120897 |
| CDKN1B | AC091982.3 | 0.370322652 | 2.11E-06 |
| CDKN2A | AC091982.3 | 0.441194778 | 9.14E-09 |
| HDAC6 | AC091982.3 | 0.363518601 | 3.33E-06 |
| IKBKE | AC091982.3 | 0.356552029 | 5.27E-06 |
| ITGA3 | AC091982.3 | -0.300881891 | 0.000142284 |
| ITGA6 | AC091982.3 | -0.319763531 | 5.00E-05 |
| MTMR14 | AC091982.3 | 0.365902436 | 2.84E-06 |
| PINK1 | AC091982.3 | -0.311592326 | 7.93E-05 |
| PRKAB1 | AC091982.3 | 0.353038734 | 6.62E-06 |
| RAF1 | AC091982.3 | 0.33890695 | 1.61E-05 |
| TP53 | AC091982.3 | 0.372381307 | 1.83E-06 |
| FOXO1 | LINC02570 | -0.3405895 | 1.45E-05 |
| RPTOR | LINC02570 | 0.308071275 | 9.63E-05 |
| EIF2AK3 | AC131532.1 | 0.366110832 | 2.80E-06 |
| GABARAPL1 | AC131532.1 | 0.390041302 | 5.25E-07 |
| KLHL24 | AC131532.1 | 0.336616859 | 1.85E-05 |
| MAPK1 | AC131532.1 | 0.300348629 | 0.000146403 |
| MBTPS2 | AC131532.1 | 0.326788612 | 3.32E-05 |
| NFE2L2 | AC131532.1 | 0.40750524 | 1.42E-07 |
| PEX14 | AC131532.1 | -0.303581795 | 0.000123036 |
| ST13 | AC131532.1 | 0.356104575 | 5.43E-06 |
| TP63 | AC131532.1 | 0.401048451 | 2.32E-07 |
| WIPI1 | AC131532.1 | 0.428187408 | 2.73E-08 |
| ATG4D | NRSN2-AS1 | 0.363313244 | 3.38E-06 |
| ATIC | NRSN2-AS1 | 0.333008738 | 2.30E-05 |
| MAPK8IP1 | NRSN2-AS1 | 0.424168971 | 3.80E-08 |
| PRKAR1A | NRSN2-AS1 | 0.333695055 | 2.20E-05 |
| RHEB | NRSN2-AS1 | 0.41925038 | 5.65E-08 |
| SQSTM1 | NRSN2-AS1 | 0.301629242 | 0.000136692 |
| VAMP7 | NRSN2-AS1 | 0.329495623 | 2.83E-05 |
| WIPI1 | NRSN2-AS1 | 0.364663288 | 3.09E-06 |
| BAG1 | AC138696.2 | 0.305892569 | 0.000108521 |
| BAX | AC138696.2 | 0.492170324 | 7.82E-11 |
| BIRC5 | AC138696.2 | 0.328318471 | 3.04E-05 |
| BNIP1 | AC138696.2 | 0.349891657 | 8.09E-06 |
| CLN3 | AC138696.2 | 0.312321306 | 7.61E-05 |
| EIF4EBP1 | AC138696.2 | 0.433842237 | 1.71E-08 |
| GNAI3 | AC138696.2 | -0.320467776 | 4.80E-05 |
| KIF5B | AC138696.2 | -0.343314936 | 1.22E-05 |
| NFKB1 | AC138696.2 | -0.310919102 | 8.23E-05 |
| RB1 | AC138696.2 | -0.317445179 | 5.70E-05 |
| WDR45 | AC138696.2 | 0.313493821 | 7.13E-05 |
| ATG16L2 | AC022144.1 | 0.303914271 | 0.000120841 |
| HDAC6 | AC022144.1 | 0.322727865 | 4.21E-05 |
| PRKAB1 | AC022144.1 | 0.324018903 | 3.91E-05 |
| SIRT2 | AC022144.1 | 0.437289968 | 1.28E-08 |
| BAX | AC104699.1 | 0.347158485 | 9.62E-06 |
| CCR2 | AC104699.1 | 0.467756706 | 8.43E-10 |
| CXCR4 | AC104699.1 | 0.493128214 | 7.09E-11 |
| IFNG | AC104699.1 | 0.384920338 | 7.60E-07 |
| ITPR1 | AC104699.1 | 0.380055453 | 1.07E-06 |
| MTMR14 | AC104699.1 | 0.397944844 | 2.93E-07 |
| RGS19 | AC104699.1 | 0.369764245 | 2.19E-06 |
| STK11 | AC104699.1 | 0.308452533 | 9.43E-05 |
| ATG9A | LMO7-AS1 | -0.324110341 | 3.89E-05 |
| BAK1 | LMO7-AS1 | -0.315051732 | 6.53E-05 |
| CASP3 | LMO7-AS1 | 0.317313204 | 5.75E-05 |
| CXCR4 | LMO7-AS1 | 0.322367456 | 4.30E-05 |
| PINK1 | LMO7-AS1 | -0.36389864 | 3.25E-06 |
| CASP8 | LINC00491 | 0.319484425 | 5.08E-05 |
| EIF4G1 | LINC00491 | 0.30962414 | 8.84E-05 |
| HIF1A | LINC00491 | 0.388829289 | 5.74E-07 |
| PEX3 | LINC00491 | 0.3589166 | 4.52E-06 |
| ATF6 | LINC02561 | 0.319806648 | 4.98E-05 |
| GABARAPL1 | LINC02561 | 0.482042208 | 2.14E-10 |
| NAMPT | LINC02561 | 0.664847865 | 3.97E-21 |
| NCKAP1 | LINC02561 | 0.364012847 | 3.22E-06 |
| NFE2L2 | LINC02561 | 0.316258074 | 6.10E-05 |
| PEX14 | LINC02561 | -0.327452817 | 3.20E-05 |
| PIK3C3 | LINC02561 | 0.30939669 | 8.95E-05 |
| RHEB | LINC02561 | 0.365228245 | 2.97E-06 |
| SQSTM1 | LINC02561 | 0.343704816 | 1.19E-05 |
| ST13 | LINC02561 | 0.333756225 | 2.20E-05 |
| TP63 | LINC02561 | 0.540164532 | 4.05E-13 |
| VAMP3 | LINC02561 | -0.311445257 | 7.99E-05 |
| ATIC | AC144652.1 | 0.350549567 | 7.76E-06 |
| CASP4 | AC144652.1 | -0.345000164 | 1.10E-05 |
| HDAC6 | AC144652.1 | 0.335958911 | 1.92E-05 |
| MAPK8IP1 | AC144652.1 | 0.402295392 | 2.12E-07 |
| PRKAR1A | AC144652.1 | 0.344295475 | 1.15E-05 |
| RHEB | AC144652.1 | 0.34914123 | 8.49E-06 |
| ST13 | AC144652.1 | 0.32385222 | 3.95E-05 |
| WIPI1 | AC144652.1 | 0.301397854 | 0.000138401 |
| ATF4 | AC010894.2 | 0.369690401 | 2.20E-06 |
| BNIP1 | AC010894.2 | 0.300029646 | 0.00014892 |
| CAPN2 | AC010894.2 | -0.373367005 | 1.71E-06 |
| GNAI3 | AC010894.2 | -0.316114893 | 6.15E-05 |
| ST13 | AC010894.2 | 0.309098303 | 9.10E-05 |
| WDR45 | AC010894.2 | 0.31679939 | 5.92E-05 |
| WIPI1 | AC010894.2 | 0.31748516 | 5.69E-05 |
| ATF4 | AC020765.2 | 0.33783755 | 1.72E-05 |
| ATG16L2 | AC020765.2 | 0.379147205 | 1.14E-06 |
| ATG3 | AC020765.2 | 0.315512961 | 6.36E-05 |
| ATG4B | AC020765.2 | 0.407508159 | 1.42E-07 |
| ATG4D | AC020765.2 | 0.31666272 | 5.96E-05 |
| ATG9A | AC020765.2 | -0.308067626 | 9.63E-05 |
| BAX | AC020765.2 | 0.457631953 | 2.14E-09 |
| BIRC5 | AC020765.2 | 0.363139798 | 3.42E-06 |
| BNIP1 | AC020765.2 | 0.315316223 | 6.43E-05 |
| CAPN10 | AC020765.2 | 0.445370495 | 6.37E-09 |
| CAPN2 | AC020765.2 | -0.324860777 | 3.72E-05 |
| CDKN1B | AC020765.2 | 0.313621763 | 7.08E-05 |
| CLN3 | AC020765.2 | 0.416372965 | 7.10E-08 |
| GNAI3 | AC020765.2 | -0.358785917 | 4.56E-06 |
| ITGA3 | AC020765.2 | -0.389016284 | 5.66E-07 |
| ITGA6 | AC020765.2 | -0.311192284 | 8.11E-05 |
| ITGB1 | AC020765.2 | -0.311832955 | 7.82E-05 |
| ITGB4 | AC020765.2 | -0.369643161 | 2.21E-06 |
| MAP2K7 | AC020765.2 | 0.40083454 | 2.36E-07 |
| MAPK1 | AC020765.2 | -0.302088056 | 0.000133362 |
| MLST8 | AC020765.2 | 0.367705701 | 2.52E-06 |
| NFKB1 | AC020765.2 | -0.304618076 | 0.000116316 |
| PELP1 | AC020765.2 | 0.381826178 | 9.47E-07 |
| PINK1 | AC020765.2 | -0.325235759 | 3.64E-05 |
| STK11 | AC020765.2 | 0.318588971 | 5.34E-05 |
| WDR45 | AC020765.2 | 0.418301881 | 6.09E-08 |
| CXCR4 | AC242842.1 | 0.344537086 | 1.13E-05 |
| ATG3 | CPNE8-AS1 | 0.394646538 | 3.75E-07 |
| RAB7A | CPNE8-AS1 | 0.321371181 | 4.56E-05 |
| ARNT | SNHG15 | -0.365275196 | 2.96E-06 |
| ARSB | SNHG15 | -0.350758148 | 7.66E-06 |
| ATF4 | SNHG15 | 0.482680413 | 2.01E-10 |
| ATG16L2 | SNHG15 | 0.308420875 | 9.45E-05 |
| ATG2B | SNHG15 | -0.391774984 | 4.63E-07 |
| ATG4B | SNHG15 | 0.466523231 | 9.46E-10 |
| BAG1 | SNHG15 | 0.338477373 | 1.65E-05 |
| BAX | SNHG15 | 0.330420989 | 2.68E-05 |
| BIRC6 | SNHG15 | -0.403558051 | 1.92E-07 |
| BNIP1 | SNHG15 | 0.434987764 | 1.55E-08 |
| CAPN10 | SNHG15 | 0.409103408 | 1.26E-07 |
| CASP1 | SNHG15 | 0.323771394 | 3.96E-05 |
| DDIT3 | SNHG15 | 0.301750136 | 0.000135807 |
| DLC1 | SNHG15 | -0.320203601 | 4.87E-05 |
| EDEM1 | SNHG15 | -0.303642859 | 0.00012263 |
| EEF2K | SNHG15 | -0.378332094 | 1.21E-06 |
| EIF2AK3 | SNHG15 | -0.354045132 | 6.20E-06 |
| EIF4G1 | SNHG15 | -0.324778838 | 3.74E-05 |
| GOPC | SNHG15 | -0.334692322 | 2.08E-05 |
| LAMP2 | SNHG15 | -0.411268646 | 1.06E-07 |
| MAP1LC3A | SNHG15 | 0.335279825 | 2.00E-05 |
| MAPK1 | SNHG15 | -0.500377879 | 3.37E-11 |
| MTOR | SNHG15 | -0.320229672 | 4.86E-05 |
| NBR1 | SNHG15 | -0.304345668 | 0.000118048 |
| NCKAP1 | SNHG15 | -0.321499086 | 4.52E-05 |
| PIK3R4 | SNHG15 | -0.361920536 | 3.71E-06 |
| PRKAR1A | SNHG15 | -0.313695975 | 7.05E-05 |
| RAB24 | SNHG15 | 0.375751694 | 1.45E-06 |
| RAC1 | SNHG15 | 0.309949739 | 8.68E-05 |
| SH3GLB1 | SNHG15 | -0.312619846 | 7.49E-05 |
| STK11 | SNHG15 | 0.335590069 | 1.97E-05 |
| USP10 | SNHG15 | -0.300727651 | 0.000143464 |
| WDFY3 | SNHG15 | -0.407125365 | 1.46E-07 |
| WDR45 | SNHG15 | 0.34537153 | 1.08E-05 |
| WIPI2 | SNHG15 | 0.318673219 | 5.32E-05 |
| BAX | AC005911.1 | 0.318019294 | 5.52E-05 |
| CDKN1B | AC005911.1 | 0.408820267 | 1.28E-07 |
| CLN3 | AC005911.1 | 0.309931827 | 8.69E-05 |
| GABARAPL1 | AC005911.1 | 0.332235844 | 2.41E-05 |
| HDAC6 | AC005911.1 | 0.31596376 | 6.20E-05 |
| ITGA3 | AC005911.1 | -0.347286525 | 9.54E-06 |
| MAP2K7 | AC005911.1 | 0.328742372 | 2.96E-05 |
| PINK1 | AC005911.1 | -0.362435094 | 3.58E-06 |
| TP53 | AC005911.1 | 0.315298472 | 6.44E-05 |
| WDR45 | AC005911.1 | 0.323410753 | 4.05E-05 |
| ARNT | AL928654.1 | 0.40599955 | 1.59E-07 |
| ARSB | AL928654.1 | 0.328589234 | 2.99E-05 |
| ATG2B | AL928654.1 | 0.403412291 | 1.94E-07 |
| DAPK1 | AL928654.1 | 0.361757807 | 3.75E-06 |
| KLHL24 | AL928654.1 | 0.383189799 | 8.60E-07 |
| MBTPS2 | AL928654.1 | 0.404005937 | 1.86E-07 |
| PARP1 | AL928654.1 | 0.366450212 | 2.74E-06 |
| PTK6 | AL928654.1 | -0.344831698 | 1.11E-05 |
| RB1CC1 | AL928654.1 | 0.340613138 | 1.45E-05 |
| ST13 | AL928654.1 | 0.377947251 | 1.24E-06 |
| TM9SF1 | AL928654.1 | 0.354164681 | 6.15E-06 |
| UVRAG | AL928654.1 | 0.374055433 | 1.63E-06 |
| BAX | LINC01605 | -0.308819865 | 9.24E-05 |
| CDKN1B | LINC01605 | -0.358633665 | 4.60E-06 |
| HDAC6 | LINC01605 | -0.360806919 | 3.99E-06 |
| IL24 | LINC01605 | 0.386612016 | 6.73E-07 |
| ITGA3 | LINC01605 | 0.373978284 | 1.64E-06 |
| ITGA6 | LINC01605 | 0.343393989 | 1.22E-05 |
| PARP1 | LINC01605 | -0.312850315 | 7.39E-05 |
| PINK1 | LINC01605 | 0.37033231 | 2.11E-06 |
| PRKAB1 | LINC01605 | -0.311435957 | 8.00E-05 |
| RHEB | LINC01605 | -0.301159484 | 0.000140183 |
| ATG4D | AC125257.1 | 0.411310683 | 1.06E-07 |
| BAK1 | AC125257.1 | -0.308918505 | 9.19E-05 |
| BECN1 | AC125257.1 | 0.330945838 | 2.60E-05 |
| CAPN10 | AC125257.1 | 0.330814103 | 2.62E-05 |
| CASP3 | AC125257.1 | 0.432502914 | 1.91E-08 |
| CASP4 | AC125257.1 | -0.302174116 | 0.000132746 |
| CD46 | AC125257.1 | 0.350266034 | 7.90E-06 |
| CDKN1A | AC125257.1 | -0.310770597 | 8.30E-05 |
| CDKN1B | AC125257.1 | 0.395185615 | 3.60E-07 |
| EIF2AK3 | AC125257.1 | 0.424312643 | 3.75E-08 |
| GABARAPL1 | AC125257.1 | 0.417744265 | 6.37E-08 |
| HDAC6 | AC125257.1 | 0.368970621 | 2.31E-06 |
| ITGA3 | AC125257.1 | -0.401591877 | 2.23E-07 |
| KLHL24 | AC125257.1 | 0.420159952 | 5.25E-08 |
| MAP2K7 | AC125257.1 | 0.407713733 | 1.40E-07 |
| NBR1 | AC125257.1 | 0.419191459 | 5.67E-08 |
| NFE2L2 | AC125257.1 | 0.443969215 | 7.19E-09 |
| PARP1 | AC125257.1 | 0.350165886 | 7.95E-06 |
| PELP1 | AC125257.1 | 0.389285089 | 5.55E-07 |
| PINK1 | AC125257.1 | -0.389079546 | 5.63E-07 |
| PTK6 | AC125257.1 | -0.339491811 | 1.55E-05 |
| RAB33B | AC125257.1 | 0.347794078 | 9.24E-06 |
| RAF1 | AC125257.1 | 0.320576159 | 4.77E-05 |
| RB1CC1 | AC125257.1 | 0.326328752 | 3.41E-05 |
| RHEB | AC125257.1 | 0.354995148 | 5.83E-06 |
| RPS6KB1 | AC125257.1 | 0.417095773 | 6.71E-08 |
| SIRT1 | AC125257.1 | 0.329636032 | 2.81E-05 |
| ULK3 | AC125257.1 | 0.337615829 | 1.74E-05 |
| UVRAG | AC125257.1 | 0.351219959 | 7.43E-06 |
| WIPI1 | AC125257.1 | 0.316555313 | 6.00E-05 |
| BAK1 | LINC00520 | 0.325030482 | 3.68E-05 |
| CASP1 | LINC00520 | 0.429558735 | 2.44E-08 |
| EIF4EBP1 | LINC00520 | 0.341548242 | 1.37E-05 |
| CLN3 | ZNF674-AS1 | 0.371121954 | 2.00E-06 |
| GABARAPL1 | ZNF674-AS1 | 0.309279964 | 9.01E-05 |
| HDAC6 | ZNF674-AS1 | 0.435592954 | 1.47E-08 |
| IKBKB | ZNF674-AS1 | 0.555154281 | 6.56E-14 |
| ITGA3 | ZNF674-AS1 | -0.316911403 | 5.88E-05 |
| KLHL24 | ZNF674-AS1 | 0.321032526 | 4.64E-05 |
| VAMP7 | ZNF674-AS1 | 0.351100171 | 7.49E-06 |
| ATG4C | AC008115.3 | 0.320420038 | 4.81E-05 |
| BAK1 | AC008115.3 | -0.378904373 | 1.16E-06 |
| BCL2 | AC008115.3 | 0.356774699 | 5.20E-06 |
| CAPN10 | AC008115.3 | 0.316038406 | 6.18E-05 |
| CDKN1B | AC008115.3 | 0.676057921 | 4.84E-22 |
| CXCR4 | AC008115.3 | 0.492202681 | 7.79E-11 |
| EEF2K | AC008115.3 | 0.346867367 | 9.80E-06 |
| EIF2AK3 | AC008115.3 | 0.30445116 | 0.000117375 |
| HDAC6 | AC008115.3 | 0.412979387 | 9.28E-08 |
| IKBKB | AC008115.3 | 0.350515958 | 7.78E-06 |
| ITGA3 | AC008115.3 | -0.336778661 | 1.83E-05 |
| MAP1LC3B | AC008115.3 | -0.301793839 | 0.000135489 |
| MAP2K7 | AC008115.3 | 0.480868895 | 2.41E-10 |
| MAPK8 | AC008115.3 | 0.379736518 | 1.10E-06 |
| NKX2-3 | AC008115.3 | 0.415888409 | 7.38E-08 |
| PINK1 | AC008115.3 | -0.34905711 | 8.53E-06 |
| PRKAB1 | AC008115.3 | 0.342093172 | 1.32E-05 |
| RAF1 | AC008115.3 | 0.33228473 | 2.40E-05 |
| SIRT1 | AC008115.3 | 0.32101729 | 4.65E-05 |
| TP53 | AC008115.3 | 0.413230725 | 9.10E-08 |
| TP73 | AC008115.3 | 0.328583091 | 2.99E-05 |
| TSC1 | AC008115.3 | 0.345492223 | 1.07E-05 |
| TSC2 | AC008115.3 | 0.412160641 | 9.90E-08 |
| ULK3 | AC008115.3 | 0.303037633 | 0.000126708 |
| UVRAG | AC008115.3 | 0.381266734 | 9.86E-07 |
| ATG16L2 | DOCK9-DT | 0.306712774 | 0.000103766 |
| ATG9B | DOCK9-DT | 0.353190695 | 6.55E-06 |
| CANX | DOCK9-DT | -0.315803775 | 6.26E-05 |
| GABARAPL2 | DOCK9-DT | 0.3229326 | 4.16E-05 |
| GOPC | DOCK9-DT | -0.30240794 | 0.000131085 |
| ULK3 | DOCK9-DT | 0.335607057 | 1.96E-05 |
| ATG4C | FGF14-AS2 | 0.371703101 | 1.92E-06 |
| ATIC | FGF14-AS2 | 0.532313375 | 1.02E-12 |
| BAG3 | FGF14-AS2 | -0.337191214 | 1.78E-05 |
| BAK1 | FGF14-AS2 | -0.308250433 | 9.54E-05 |
| BCL2 | FGF14-AS2 | 0.304109937 | 0.000119567 |
| CAPN2 | FGF14-AS2 | -0.338367184 | 1.66E-05 |
| CDKN1A | FGF14-AS2 | -0.300421256 | 0.000145836 |
| DIRAS3 | FGF14-AS2 | 0.599449641 | 1.71E-16 |
| EEF2 | FGF14-AS2 | 0.386792356 | 6.65E-07 |
| HDAC6 | FGF14-AS2 | 0.399496424 | 2.61E-07 |
| MAPK8IP1 | FGF14-AS2 | 0.621961012 | 5.76E-18 |
| PARP1 | FGF14-AS2 | 0.317053065 | 5.83E-05 |
| PRKAR1A | FGF14-AS2 | 0.454413199 | 2.86E-09 |
| PRKCQ | FGF14-AS2 | 0.310938567 | 8.22E-05 |
| PTK6 | FGF14-AS2 | -0.324028536 | 3.91E-05 |
| ST13 | FGF14-AS2 | 0.414234171 | 8.41E-08 |
| ULK2 | FGF14-AS2 | 0.342583357 | 1.28E-05 |
| VAMP7 | FGF14-AS2 | 0.357583233 | 4.93E-06 |
| ATIC | AL359513.1 | 0.420020088 | 5.31E-08 |
| BAX | AL359513.1 | 0.304803748 | 0.000115149 |
| BIRC5 | AL359513.1 | 0.453180645 | 3.20E-09 |
| CAPN2 | AL359513.1 | -0.303228283 | 0.00012541 |
| FKBP1A | AL359513.1 | 0.341121985 | 1.40E-05 |
| GABARAPL2 | AL359513.1 | 0.432294256 | 1.94E-08 |
| MAPK1 | AL359513.1 | -0.322950006 | 4.16E-05 |
| MAPK8IP1 | AL359513.1 | 0.519583637 | 4.28E-12 |
| WDR45 | AL359513.1 | 0.34205195 | 1.32E-05 |
| PIK3C3 | LINC00393 | 0.317641136 | 5.64E-05 |
| UVRAG | LINC00393 | 0.394964189 | 3.66E-07 |
| VAMP3 | AC105446.1 | 0.34847861 | 8.85E-06 |
| ATG16L2 | AC093484.4 | 0.304138034 | 0.000119385 |
| ATG4B | AC093484.4 | 0.374360476 | 1.60E-06 |
| CAPN10 | AC093484.4 | 0.374608798 | 1.57E-06 |
| PELP1 | AC093484.4 | 0.333683971 | 2.21E-05 |
| VEGFA | AC093484.4 | 0.35490179 | 5.87E-06 |
| ATG3 | LINC01305 | 0.312166937 | 7.68E-05 |
| CX3CL1 | LINC01305 | 0.306531738 | 0.000104799 |
| CXCR4 | LINC01305 | 0.527126358 | 1.84E-12 |
| RAF1 | LINC01305 | 0.315012625 | 6.54E-05 |
| TP53 | LINC01305 | 0.41449419 | 8.24E-08 |
| TSC2 | LINC01305 | 0.362215866 | 3.63E-06 |
| ARNT | AL021707.6 | -0.408719056 | 1.29E-07 |
| ATF4 | AL021707.6 | 0.600987592 | 1.37E-16 |
| ATF6 | AL021707.6 | -0.322463598 | 4.28E-05 |
| ATG16L2 | AL021707.6 | 0.417995892 | 6.24E-08 |
| ATG2B | AL021707.6 | -0.472845888 | 5.21E-10 |
| ATG3 | AL021707.6 | 0.305011212 | 0.000113858 |
| ATG4B | AL021707.6 | 0.6706577 | 1.35E-21 |
| BAG1 | AL021707.6 | 0.416290386 | 7.15E-08 |
| BAG3 | AL021707.6 | -0.348282613 | 8.96E-06 |
| BAX | AL021707.6 | 0.596504681 | 2.62E-16 |
| BIRC5 | AL021707.6 | 0.357401156 | 4.99E-06 |
| BIRC6 | AL021707.6 | -0.48411634 | 1.75E-10 |
| BNIP1 | AL021707.6 | 0.578529068 | 3.18E-15 |
| CANX | AL021707.6 | -0.434305487 | 1.64E-08 |
| CAPN10 | AL021707.6 | 0.583522779 | 1.61E-15 |
| CAPN2 | AL021707.6 | -0.30481496 | 0.000115079 |
| CDKN2A | AL021707.6 | 0.433360061 | 1.78E-08 |
| CLN3 | AL021707.6 | 0.329120762 | 2.90E-05 |
| DAPK2 | AL021707.6 | 0.449567683 | 4.41E-09 |
| DDIT3 | AL021707.6 | 0.346090556 | 1.03E-05 |
| DLC1 | AL021707.6 | -0.345793124 | 1.05E-05 |
| EEF2 | AL021707.6 | -0.309246387 | 9.03E-05 |
| EIF4G1 | AL021707.6 | -0.373937025 | 1.64E-06 |
| FOXO1 | AL021707.6 | -0.305424723 | 0.000111324 |
| GOPC | AL021707.6 | -0.409506329 | 1.22E-07 |
| HGS | AL021707.6 | -0.315795306 | 6.26E-05 |
| HIF1A | AL021707.6 | -0.333739049 | 2.20E-05 |
| ITGA6 | AL021707.6 | -0.326849156 | 3.31E-05 |
| ITGB1 | AL021707.6 | -0.380443439 | 1.04E-06 |
| KIF5B | AL021707.6 | -0.448282179 | 4.94E-09 |
| LAMP2 | AL021707.6 | -0.395794765 | 3.44E-07 |
| MAP1LC3A | AL021707.6 | 0.303796827 | 0.000121612 |
| MAPK1 | AL021707.6 | -0.494704027 | 6.04E-11 |
| MBTPS2 | AL021707.6 | -0.386020905 | 7.03E-07 |
| MTOR | AL021707.6 | -0.374599837 | 1.57E-06 |
| NAF1 | AL021707.6 | -0.333256766 | 2.26E-05 |
| NBR1 | AL021707.6 | -0.339875686 | 1.51E-05 |
| NCKAP1 | AL021707.6 | -0.497603588 | 4.49E-11 |
| NPC1 | AL021707.6 | -0.31386265 | 6.98E-05 |
| PELP1 | AL021707.6 | 0.3641945 | 3.19E-06 |
| PEX14 | AL021707.6 | 0.349924273 | 8.07E-06 |
| PINK1 | AL021707.6 | -0.36606711 | 2.81E-06 |
| PTEN | AL021707.6 | -0.345784858 | 1.05E-05 |
| RAB24 | AL021707.6 | 0.562860507 | 2.48E-14 |
| RB1 | AL021707.6 | -0.335464814 | 1.98E-05 |
| RPTOR | AL021707.6 | -0.366567701 | 2.72E-06 |
| SPNS1 | AL021707.6 | -0.308554148 | 9.38E-05 |
| STK11 | AL021707.6 | 0.43142857 | 2.09E-08 |
| TM9SF1 | AL021707.6 | -0.340148363 | 1.49E-05 |
| TP63 | AL021707.6 | -0.320502494 | 4.79E-05 |
| ULK3 | AL021707.6 | 0.320439387 | 4.81E-05 |
| USP10 | AL021707.6 | -0.478449285 | 3.04E-10 |
| WDFY3 | AL021707.6 | -0.450164107 | 4.18E-09 |
| WDR45 | AL021707.6 | 0.539956686 | 4.15E-13 |
| ZFYVE1 | AL021707.6 | -0.30800533 | 9.67E-05 |
| CX3CL1 | BLACAT1 | 0.392000175 | 4.55E-07 |
| HDAC6 | BLACAT1 | 0.419179128 | 5.68E-08 |
| MAPK8 | BLACAT1 | 0.313749738 | 7.03E-05 |
| MAPK9 | BLACAT1 | 0.303310075 | 0.000124857 |
| NKX2-3 | BLACAT1 | 0.452733336 | 3.33E-09 |
| TP73 | BLACAT1 | 0.470069118 | 6.78E-10 |
| TSC2 | BLACAT1 | 0.41928801 | 5.63E-08 |
| BNIP1 | HOXB-AS4 | 0.349153805 | 8.48E-06 |
| ATG16L2 | AL137003.2 | 0.305027071 | 0.000113759 |
| BAK1 | AL137003.2 | -0.310106715 | 8.61E-05 |
| CAPN10 | AL137003.2 | 0.350741747 | 7.67E-06 |
| CDKN1B | AL137003.2 | 0.303436963 | 0.000124003 |
| CTSD | AL137003.2 | -0.316151349 | 6.14E-05 |
| CXCR4 | AL137003.2 | 0.309423637 | 8.94E-05 |
| ITGA3 | AL137003.2 | -0.341687856 | 1.35E-05 |
| NFE2L2 | AL137003.2 | 0.342988093 | 1.25E-05 |
| ULK3 | AL137003.2 | 0.354253863 | 6.12E-06 |
| UVRAG | AL137003.2 | 0.404681557 | 1.76E-07 |
| ATF4 | AC132192.2 | 0.47087093 | 6.29E-10 |
| ATG10 | AC132192.2 | 0.327518416 | 3.18E-05 |
| ATG12 | AC132192.2 | 0.332873839 | 2.32E-05 |
| ATG16L2 | AC132192.2 | 0.483674959 | 1.83E-10 |
| ATG4B | AC132192.2 | 0.492215293 | 7.78E-11 |
| ATG4C | AC132192.2 | 0.312767226 | 7.42E-05 |
| ATG4D | AC132192.2 | 0.321899749 | 4.42E-05 |
| ATIC | AC132192.2 | 0.307099134 | 0.000101594 |
| BAG1 | AC132192.2 | 0.30203453 | 0.000133747 |
| BAG3 | AC132192.2 | -0.36073217 | 4.01E-06 |
| BAX | AC132192.2 | 0.472153707 | 5.57E-10 |
| BIRC5 | AC132192.2 | 0.369527523 | 2.22E-06 |
| BIRC6 | AC132192.2 | -0.330099878 | 2.73E-05 |
| BNIP1 | AC132192.2 | 0.515323727 | 6.84E-12 |
| CAPN10 | AC132192.2 | 0.61496136 | 1.70E-17 |
| CAPN2 | AC132192.2 | -0.368602736 | 2.37E-06 |
| CDKN2A | AC132192.2 | 0.316138525 | 6.14E-05 |
| CLN3 | AC132192.2 | 0.367064485 | 2.63E-06 |
| CXCR4 | AC132192.2 | 0.384965266 | 7.58E-07 |
| EIF4G1 | AC132192.2 | -0.403666463 | 1.91E-07 |
| FKBP1A | AC132192.2 | 0.327964464 | 3.10E-05 |
| HDAC6 | AC132192.2 | 0.350133597 | 7.97E-06 |
| HIF1A | AC132192.2 | -0.311810718 | 7.83E-05 |
| ITGA6 | AC132192.2 | -0.318071805 | 5.50E-05 |
| ITGB1 | AC132192.2 | -0.353594235 | 6.38E-06 |
| KIF5B | AC132192.2 | -0.333168298 | 2.28E-05 |
| MAP1LC3A | AC132192.2 | 0.38920066 | 5.58E-07 |
| MAP2K7 | AC132192.2 | 0.308698987 | 9.30E-05 |
| MAPK1 | AC132192.2 | -0.378851931 | 1.17E-06 |
| MLST8 | AC132192.2 | 0.410460034 | 1.13E-07 |
| MTMR14 | AC132192.2 | 0.339555102 | 1.54E-05 |
| NCKAP1 | AC132192.2 | -0.347883767 | 9.19E-06 |
| PEX14 | AC132192.2 | 0.312776076 | 7.42E-05 |
| PINK1 | AC132192.2 | -0.335297598 | 2.00E-05 |
| RAB24 | AC132192.2 | 0.461057275 | 1.57E-09 |
| RB1 | AC132192.2 | -0.325394982 | 3.61E-05 |
| STK11 | AC132192.2 | 0.380178399 | 1.06E-06 |
| TSC2 | AC132192.2 | 0.350440319 | 7.81E-06 |
| ULK3 | AC132192.2 | 0.415048552 | 7.89E-08 |
| USP10 | AC132192.2 | -0.375960007 | 1.43E-06 |
| WDFY3 | AC132192.2 | -0.366375112 | 2.75E-06 |
| WDR45 | AC132192.2 | 0.621678795 | 6.02E-18 |
| ATG9B | DSG1-AS1 | 0.333906809 | 2.18E-05 |
| NFE2L2 | DSG1-AS1 | 0.300183683 | 0.0001477 |
| DIRAS3 | MIR4458HG | 0.464970699 | 1.09E-09 |
| AMBRA1 | AP000759.1 | 0.320794151 | 4.71E-05 |
| ATF4 | AP000759.1 | -0.387932658 | 6.12E-07 |
| ATG2B | AP000759.1 | 0.33461661 | 2.09E-05 |
| CAMKK2 | AP000759.1 | 0.313839244 | 6.99E-05 |
| MTOR | AP000759.1 | 0.441312733 | 9.05E-09 |
| NFKB1 | AP000759.1 | 0.317210195 | 5.78E-05 |
| PTEN | AP000759.1 | 0.301805646 | 0.000135403 |
| RELA | AP000759.1 | 0.610063565 | 3.58E-17 |
| SH3GLB1 | AP000759.1 | 0.344506374 | 1.14E-05 |
| USP10 | AP000759.1 | 0.320226918 | 4.87E-05 |
| ATG16L2 | AC116914.2 | 0.431974078 | 2.00E-08 |
| ATG4B | AC116914.2 | 0.487844459 | 1.21E-10 |
| BAX | AC116914.2 | 0.306789617 | 0.000103331 |
| CAPN10 | AC116914.2 | 0.522137009 | 3.22E-12 |
| CXCR4 | AC116914.2 | 0.527817579 | 1.70E-12 |
| DAPK2 | AC116914.2 | 0.361946302 | 3.70E-06 |
| GABARAP | AC116914.2 | 0.301097506 | 0.000140649 |
| HDAC6 | AC116914.2 | 0.313988897 | 6.93E-05 |
| IFNG | AC116914.2 | 0.336933465 | 1.81E-05 |
| IKBKB | AC116914.2 | 0.433115165 | 1.81E-08 |
| MAP2K7 | AC116914.2 | 0.446972013 | 5.54E-09 |
| NKX2-3 | AC116914.2 | 0.308988042 | 9.16E-05 |
| PELP1 | AC116914.2 | 0.375401236 | 1.49E-06 |
| RAB24 | AC116914.2 | 0.389755166 | 5.36E-07 |
| STK11 | AC116914.2 | 0.455504948 | 2.60E-09 |
| TSC2 | AC116914.2 | 0.38387062 | 8.19E-07 |
| ULK3 | AC116914.2 | 0.306431143 | 0.000105377 |
| WDR45 | AC116914.2 | 0.388823241 | 5.74E-07 |
| ATG16L2 | AC015660.2 | 0.4161649 | 7.22E-08 |
| ATG9B | AC015660.2 | 0.407793992 | 1.39E-07 |
| BID | AC015660.2 | 0.431953785 | 2.00E-08 |
| FOXO1 | AC015660.2 | -0.323138096 | 4.11E-05 |
| RHEB | AC015660.2 | 0.307669836 | 9.85E-05 |
| ULK3 | AC015660.2 | 0.301058117 | 0.000140947 |
| CALCOCO2 | AC093673.1 | -0.333084631 | 2.29E-05 |
| ATG7 | AC008011.2 | -0.313801015 | 7.01E-05 |
| FAS | AC008011.2 | -0.304005439 | 0.000120246 |
| IKBKE | AC008011.2 | -0.401245195 | 2.29E-07 |
| MTMR14 | AC008011.2 | -0.399941542 | 2.53E-07 |
| SPHK1 | AC008011.2 | 0.301591013 | 0.000136973 |
| TP73 | AC008011.2 | -0.313249987 | 7.23E-05 |
| VEGFA | AC008011.2 | 0.333565055 | 2.22E-05 |
| AMBRA1 | AC040169.1 | -0.308637008 | 9.34E-05 |
| GABARAPL2 | AC040169.1 | 0.401044796 | 2.32E-07 |
| MAP1LC3B | AC040169.1 | 0.310060351 | 8.63E-05 |
| ARNT | INE1 | 0.315943758 | 6.21E-05 |
| BECN1 | INE1 | 0.392769905 | 4.30E-07 |
| CD46 | INE1 | 0.369726892 | 2.19E-06 |
| EIF2AK3 | INE1 | 0.336564162 | 1.85E-05 |
| HDAC6 | INE1 | 0.560931304 | 3.17E-14 |
| IKBKB | INE1 | 0.631196492 | 1.32E-18 |
| KLHL24 | INE1 | 0.424955254 | 3.56E-08 |
| MAPK8 | INE1 | 0.30350965 | 0.000123517 |
| MAPK9 | INE1 | 0.316520011 | 6.01E-05 |
| NBR1 | INE1 | 0.322040411 | 4.38E-05 |
| PARP1 | INE1 | 0.313174617 | 7.26E-05 |
| PIK3R4 | INE1 | 0.346612731 | 9.96E-06 |
| RB1CC1 | INE1 | 0.346521671 | 1.00E-05 |
| SIRT1 | INE1 | 0.357771568 | 4.87E-06 |
| SPNS1 | INE1 | 0.304181043 | 0.000119107 |
| TSC1 | INE1 | 0.30951529 | 8.89E-05 |
| TSC2 | INE1 | 0.318467425 | 5.38E-05 |
| ULK2 | INE1 | 0.324519793 | 3.80E-05 |
| UVRAG | INE1 | 0.335602605 | 1.96E-05 |
| VAMP7 | INE1 | 0.303915757 | 0.000120832 |
| ATG9A | AC092171.2 | 0.300184709 | 0.000147692 |
| IL24 | AC092171.2 | 0.305820744 | 0.000108947 |
| RAC1 | AC092171.2 | 0.471700373 | 5.81E-10 |
| WIPI2 | AC092171.2 | 0.476643368 | 3.62E-10 |
| ATG4C | AC104041.1 | -0.305102532 | 0.000113294 |
| GABARAPL1 | AC104041.1 | 0.395852965 | 3.43E-07 |
| KLHL24 | AC104041.1 | 0.305688238 | 0.000109737 |
| TP63 | AC104041.1 | 0.300176115 | 0.00014776 |
| UVRAG | AC104041.1 | 0.356516954 | 5.28E-06 |
| WIPI1 | AC104041.1 | 0.309373241 | 8.96E-05 |
| ATG10 | PTOV1-AS2 | 0.305562987 | 0.000110489 |
| ATG12 | PTOV1-AS2 | 0.402856487 | 2.03E-07 |
| ATG16L2 | PTOV1-AS2 | 0.451223618 | 3.81E-09 |
| ATG3 | PTOV1-AS2 | 0.333072599 | 2.29E-05 |
| ATG4B | PTOV1-AS2 | 0.4837532 | 1.81E-10 |
| ATG4C | PTOV1-AS2 | 0.42526208 | 3.47E-08 |
| ATG4D | PTOV1-AS2 | 0.362787256 | 3.50E-06 |
| BAX | PTOV1-AS2 | 0.391218243 | 4.82E-07 |
| BCL2 | PTOV1-AS2 | 0.34624514 | 1.02E-05 |
| CAPN10 | PTOV1-AS2 | 0.611786078 | 2.76E-17 |
| CDKN1B | PTOV1-AS2 | 0.392179547 | 4.49E-07 |
| CDKN2A | PTOV1-AS2 | 0.340427865 | 1.46E-05 |
| CFLAR | PTOV1-AS2 | 0.324024662 | 3.91E-05 |
| CX3CL1 | PTOV1-AS2 | 0.342032874 | 1.33E-05 |
| CXCR4 | PTOV1-AS2 | 0.596618849 | 2.57E-16 |
| DAPK2 | PTOV1-AS2 | 0.365777024 | 2.87E-06 |
| HDAC6 | PTOV1-AS2 | 0.523751537 | 2.69E-12 |
| HSPA5 | PTOV1-AS2 | -0.321408415 | 4.55E-05 |
| IFNG | PTOV1-AS2 | 0.305403948 | 0.00011145 |
| IKBKB | PTOV1-AS2 | 0.469426912 | 7.21E-10 |
| IKBKE | PTOV1-AS2 | 0.31460001 | 6.70E-05 |
| MAP2K7 | PTOV1-AS2 | 0.524037789 | 2.61E-12 |
| MAPK8 | PTOV1-AS2 | 0.408284236 | 1.34E-07 |
| MAPK9 | PTOV1-AS2 | 0.324551507 | 3.79E-05 |
| MTMR14 | PTOV1-AS2 | 0.389701017 | 5.39E-07 |
| NKX2-3 | PTOV1-AS2 | 0.42391432 | 3.88E-08 |
| PEX14 | PTOV1-AS2 | 0.387436338 | 6.34E-07 |
| PRKAB1 | PTOV1-AS2 | 0.383667548 | 8.31E-07 |
| RAB24 | PTOV1-AS2 | 0.435468304 | 1.49E-08 |
| RAF1 | PTOV1-AS2 | 0.446868798 | 5.59E-09 |
| STK11 | PTOV1-AS2 | 0.530665525 | 1.23E-12 |
| TP53 | PTOV1-AS2 | 0.406757421 | 1.50E-07 |
| TP73 | PTOV1-AS2 | 0.412615644 | 9.55E-08 |
| TSC1 | PTOV1-AS2 | 0.311404793 | 8.01E-05 |
| TSC2 | PTOV1-AS2 | 0.528799623 | 1.52E-12 |
| ULK3 | PTOV1-AS2 | 0.514528066 | 7.46E-12 |
| WDR45 | PTOV1-AS2 | 0.438448652 | 1.16E-08 |
| ATG16L2 | AC008649.1 | 0.338033349 | 1.69E-05 |
| ATG4D | AC008649.1 | 0.323372898 | 4.06E-05 |
| DAPK2 | AC008649.1 | 0.322282209 | 4.32E-05 |
| ITGA3 | AC008649.1 | -0.309290547 | 9.01E-05 |
| PELP1 | AC008649.1 | 0.513348702 | 8.48E-12 |
| PINK1 | AC008649.1 | -0.315549325 | 6.35E-05 |
| VAMP3 | AC008649.1 | -0.300495556 | 0.000145258 |
| BIRC6 | AL513550.1 | 0.34909085 | 8.51E-06 |
| CTSD | AL513550.1 | -0.311599417 | 7.92E-05 |
| EEF2 | AL513550.1 | 0.310060278 | 8.63E-05 |
| EIF2AK3 | AL513550.1 | 0.378495147 | 1.20E-06 |
| ERN1 | AL513550.1 | 0.367379166 | 2.57E-06 |
| FOXO3 | AL513550.1 | 0.475790208 | 3.93E-10 |
| GABARAPL1 | AL513550.1 | 0.36938177 | 2.25E-06 |
| GOPC | AL513550.1 | 0.350350316 | 7.86E-06 |
| KLHL24 | AL513550.1 | 0.440701525 | 9.54E-09 |
| MBTPS2 | AL513550.1 | 0.326685248 | 3.34E-05 |
| NBR1 | AL513550.1 | 0.344091607 | 1.17E-05 |
| NCKAP1 | AL513550.1 | 0.351861162 | 7.14E-06 |
| NFE2L2 | AL513550.1 | 0.424672117 | 3.64E-08 |
| RB1CC1 | AL513550.1 | 0.395003492 | 3.65E-07 |
| RPS6KB1 | AL513550.1 | 0.329189339 | 2.88E-05 |
| SIRT1 | AL513550.1 | 0.323446436 | 4.04E-05 |
| ST13 | AL513550.1 | 0.345028868 | 1.10E-05 |
| TP63 | AL513550.1 | 0.328829733 | 2.95E-05 |
| UVRAG | AL513550.1 | 0.412239317 | 9.84E-08 |
| VAMP3 | AL513550.1 | -0.313302079 | 7.20E-05 |
| WIPI1 | AL513550.1 | 0.335392524 | 1.99E-05 |
| ATIC | TFAP2A-AS1 | 0.525629607 | 2.18E-12 |
| BAX | TFAP2A-AS1 | 0.327387358 | 3.21E-05 |
| BIRC5 | TFAP2A-AS1 | 0.515390199 | 6.79E-12 |
| FKBP1A | TFAP2A-AS1 | 0.351922791 | 7.11E-06 |
| GABARAPL2 | TFAP2A-AS1 | 0.326010707 | 3.48E-05 |
| HDAC6 | TFAP2A-AS1 | 0.353246222 | 6.53E-06 |
| MAPK8IP1 | TFAP2A-AS1 | 0.698982575 | 4.82E-24 |
| MLST8 | TFAP2A-AS1 | 0.358770447 | 4.56E-06 |
| NCKAP1 | TFAP2A-AS1 | -0.314886646 | 6.59E-05 |
| PRKAR1A | TFAP2A-AS1 | 0.449670252 | 4.37E-09 |
| WDR45 | TFAP2A-AS1 | 0.300253331 | 0.000147151 |
| BAK1 | AC036214.2 | -0.327207272 | 3.24E-05 |
| KLHL24 | AC036214.2 | 0.336656058 | 1.84E-05 |
| MAP2K7 | AC036214.2 | 0.364212153 | 3.18E-06 |
| NFE2L2 | AC036214.2 | 0.353222496 | 6.54E-06 |
| RB1CC1 | AC036214.2 | 0.303245665 | 0.000125292 |
| TP63 | AC036214.2 | 0.306323794 | 0.000105996 |
| VAMP3 | AC036214.2 | -0.354443352 | 6.04E-06 |
| BAX | GLIS3-AS1 | 0.44167956 | 8.77E-09 |
| BIRC5 | GLIS3-AS1 | 0.388345292 | 5.94E-07 |
| EIF4EBP1 | GLIS3-AS1 | 0.380685246 | 1.03E-06 |
| ATF4 | AC008610.1 | 0.34884245 | 8.65E-06 |
| ATG2B | AC008610.1 | -0.309813457 | 8.75E-05 |
| ATG4B | AC008610.1 | 0.457638551 | 2.14E-09 |
| ATIC | AC008610.1 | 0.339452582 | 1.55E-05 |
| BAG1 | AC008610.1 | 0.367593812 | 2.54E-06 |
| BAG3 | AC008610.1 | -0.371545011 | 1.94E-06 |
| BAX | AC008610.1 | 0.596828135 | 2.50E-16 |
| BIRC5 | AC008610.1 | 0.534062907 | 8.29E-13 |
| BIRC6 | AC008610.1 | -0.370416822 | 2.09E-06 |
| BNIP1 | AC008610.1 | 0.576494486 | 4.18E-15 |
| CAPN10 | AC008610.1 | 0.507973143 | 1.51E-11 |
| CAPN2 | AC008610.1 | -0.361274944 | 3.87E-06 |
| CDKN2A | AC008610.1 | 0.315887493 | 6.23E-05 |
| CLN3 | AC008610.1 | 0.434018011 | 1.68E-08 |
| CXCR4 | AC008610.1 | 0.389621136 | 5.42E-07 |
| EIF4G1 | AC008610.1 | -0.377582367 | 1.28E-06 |
| FKBP1A | AC008610.1 | 0.414835783 | 8.02E-08 |
| GABARAP | AC008610.1 | 0.349507118 | 8.29E-06 |
| GOPC | AC008610.1 | -0.379496576 | 1.12E-06 |
| HIF1A | AC008610.1 | -0.34705793 | 9.68E-06 |
| ITGA6 | AC008610.1 | -0.346474156 | 1.00E-05 |
| ITGB1 | AC008610.1 | -0.352943913 | 6.66E-06 |
| ITGB4 | AC008610.1 | -0.341890048 | 1.34E-05 |
| KIF5B | AC008610.1 | -0.377570625 | 1.28E-06 |
| LAMP2 | AC008610.1 | -0.342426455 | 1.29E-05 |
| MAPK1 | AC008610.1 | -0.491510089 | 8.36E-11 |
| MAPK8IP1 | AC008610.1 | 0.382355128 | 9.13E-07 |
| MLST8 | AC008610.1 | 0.381395854 | 9.77E-07 |
| MTMR14 | AC008610.1 | 0.414384995 | 8.31E-08 |
| NCKAP1 | AC008610.1 | -0.462474618 | 1.38E-09 |
| NPC1 | AC008610.1 | -0.302316176 | 0.000131734 |
| PELP1 | AC008610.1 | 0.318406135 | 5.40E-05 |
| PEX14 | AC008610.1 | 0.371395593 | 1.96E-06 |
| RAB1A | AC008610.1 | -0.32926559 | 2.87E-05 |
| RAB24 | AC008610.1 | 0.467487439 | 8.65E-10 |
| STK11 | AC008610.1 | 0.341632125 | 1.36E-05 |
| TP63 | AC008610.1 | -0.36375077 | 3.28E-06 |
| USP10 | AC008610.1 | -0.374281397 | 1.61E-06 |
| WDFY3 | AC008610.1 | -0.406558911 | 1.53E-07 |
| WDR45 | AC008610.1 | 0.594162128 | 3.65E-16 |
| ATF4 | SNHG11 | 0.446590722 | 5.73E-09 |
| ATG16L2 | SNHG11 | 0.353253819 | 6.53E-06 |
| ATG2B | SNHG11 | -0.303850713 | 0.000121258 |
| ATG3 | SNHG11 | 0.305423986 | 0.000111329 |
| ATG4B | SNHG11 | 0.556191755 | 5.77E-14 |
| BAG1 | SNHG11 | 0.309256802 | 9.02E-05 |
| BAG3 | SNHG11 | -0.381509617 | 9.69E-07 |
| BAX | SNHG11 | 0.470014251 | 6.82E-10 |
| BIRC5 | SNHG11 | 0.383099888 | 8.66E-07 |
| BIRC6 | SNHG11 | -0.355777314 | 5.54E-06 |
| BNIP1 | SNHG11 | 0.4956022 | 5.51E-11 |
| CANX | SNHG11 | -0.352809762 | 6.71E-06 |
| CAPN10 | SNHG11 | 0.552846312 | 8.74E-14 |
| CAPN2 | SNHG11 | -0.316163556 | 6.13E-05 |
| CLN3 | SNHG11 | 0.375172366 | 1.51E-06 |
| CXCR4 | SNHG11 | 0.323523995 | 4.02E-05 |
| EIF4G1 | SNHG11 | -0.345708266 | 1.05E-05 |
| FKBP1A | SNHG11 | 0.378852104 | 1.17E-06 |
| HDAC6 | SNHG11 | 0.307112389 | 0.000101521 |
| IKBKB | SNHG11 | 0.303403157 | 0.00012423 |
| ITGA3 | SNHG11 | -0.312138874 | 7.69E-05 |
| ITGB1 | SNHG11 | -0.311598799 | 7.92E-05 |
| ITGB4 | SNHG11 | -0.311376665 | 8.02E-05 |
| KIF5B | SNHG11 | -0.347331325 | 9.52E-06 |
| MAP1LC3A | SNHG11 | 0.314503704 | 6.74E-05 |
| MAPK1 | SNHG11 | -0.318379773 | 5.41E-05 |
| MLST8 | SNHG11 | 0.336313979 | 1.88E-05 |
| NCKAP1 | SNHG11 | -0.331634413 | 2.49E-05 |
| PINK1 | SNHG11 | -0.301197926 | 0.000139894 |
| RAB24 | SNHG11 | 0.405923508 | 1.60E-07 |
| RB1 | SNHG11 | -0.351126396 | 7.48E-06 |
| STK11 | SNHG11 | 0.396779636 | 3.20E-07 |
| ULK3 | SNHG11 | 0.334658873 | 2.08E-05 |
| USP10 | SNHG11 | -0.351935843 | 7.10E-06 |
| WDFY3 | SNHG11 | -0.348184381 | 9.02E-06 |
| WDR45 | SNHG11 | 0.587418982 | 9.42E-16 |
| EIF2S1 | LINC02562 | -0.325163916 | 3.66E-05 |
| HDAC6 | LINC02562 | 0.401564633 | 2.24E-07 |
| IKBKB | LINC02562 | 0.398247658 | 2.87E-07 |
| IKBKE | LINC02562 | 0.305633947 | 0.000110062 |
| KLHL24 | LINC02562 | 0.322876446 | 4.18E-05 |
| PIK3R4 | LINC02562 | 0.306456844 | 0.000105229 |
| PRKAB1 | LINC02562 | 0.373744915 | 1.67E-06 |
| ATG4A | AC063948.1 | 0.39571759 | 3.46E-07 |
| ATG4C | AC063948.1 | 0.38072809 | 1.02E-06 |
| ATIC | AC063948.1 | 0.430142973 | 2.32E-08 |
| BIRC5 | AC063948.1 | 0.408492975 | 1.32E-07 |
| HDAC6 | AC063948.1 | 0.581842394 | 2.03E-15 |
| MAPK8IP1 | AC063948.1 | 0.640405769 | 2.90E-19 |
| MTMR14 | AC063948.1 | 0.330350332 | 2.69E-05 |
| PRKAR1A | AC063948.1 | 0.544029562 | 2.56E-13 |
| RAF1 | AC063948.1 | 0.36029882 | 4.12E-06 |
| VAMP7 | AC063948.1 | 0.366452296 | 2.74E-06 |
| WDR45 | AC063948.1 | 0.379518552 | 1.11E-06 |
| WIPI1 | AC063948.1 | 0.334996685 | 2.04E-05 |
| NFE2L2 | LINC00462 | 0.317818007 | 5.58E-05 |
| FKBP1B | AC074389.2 | 0.360486378 | 4.07E-06 |
| ARSB | AC015922.3 | 0.311200728 | 8.10E-05 |
| ATIC | AC015922.3 | 0.36744115 | 2.56E-06 |
| DAPK1 | AC015922.3 | 0.309923018 | 8.70E-05 |
| MAPK8IP1 | AC015922.3 | 0.345645081 | 1.06E-05 |
| PRKAR1A | AC015922.3 | 0.425945234 | 3.28E-08 |
| ST13 | AC015922.3 | 0.348469427 | 8.86E-06 |
| ULK2 | AC015922.3 | 0.382546968 | 9.00E-07 |
| ARNT | AC017100.1 | 0.36809784 | 2.45E-06 |
| ARSB | AC017100.1 | 0.403677623 | 1.90E-07 |
| BAK1 | AC017100.1 | -0.310088595 | 8.62E-05 |
| BCL2 | AC017100.1 | 0.570712762 | 8.98E-15 |
| BIRC6 | AC017100.1 | 0.306032794 | 0.000107694 |
| CASP3 | AC017100.1 | 0.336509332 | 1.86E-05 |
| CD46 | AC017100.1 | 0.300624259 | 0.000144261 |
| CDKN1B | AC017100.1 | 0.409530319 | 1.21E-07 |
| DRAM1 | AC017100.1 | 0.382257259 | 9.19E-07 |
| EEF2 | AC017100.1 | 0.369914636 | 2.17E-06 |
| EEF2K | AC017100.1 | 0.323904708 | 3.93E-05 |
| EIF2AK3 | AC017100.1 | 0.320757828 | 4.72E-05 |
| FAS | AC017100.1 | 0.350239001 | 7.91E-06 |
| HDAC6 | AC017100.1 | 0.502845235 | 2.60E-11 |
| KLHL24 | AC017100.1 | 0.38426479 | 7.97E-07 |
| MAPK8 | AC017100.1 | 0.420311032 | 5.19E-08 |
| MBTPS2 | AC017100.1 | 0.370754192 | 2.05E-06 |
| NBR1 | AC017100.1 | 0.318105119 | 5.49E-05 |
| NKX2-3 | AC017100.1 | 0.422446727 | 4.37E-08 |
| PARP1 | AC017100.1 | 0.463576861 | 1.24E-09 |
| PIK3C3 | AC017100.1 | 0.389245662 | 5.57E-07 |
| PRKAB1 | AC017100.1 | 0.329734622 | 2.79E-05 |
| PRKAR1A | AC017100.1 | 0.352760702 | 6.74E-06 |
| PTK6 | AC017100.1 | -0.322552791 | 4.25E-05 |
| SIRT1 | AC017100.1 | 0.396714169 | 3.22E-07 |
| ST13 | AC017100.1 | 0.336565725 | 1.85E-05 |
| TP53 | AC017100.1 | 0.427301849 | 2.94E-08 |
| TP73 | AC017100.1 | 0.385260734 | 7.42E-07 |
| TSC2 | AC017100.1 | 0.38000147 | 1.08E-06 |
| ULK2 | AC017100.1 | 0.43915476 | 1.09E-08 |
| UVRAG | AC017100.1 | 0.376097494 | 1.42E-06 |
| ATG10 | AC104126.1 | 0.348010825 | 9.12E-06 |
| ATG4C | AC104126.1 | 0.303374429 | 0.000124423 |
| ATG4D | AC104126.1 | 0.356889243 | 5.16E-06 |
| ATG7 | AC104126.1 | 0.383183238 | 8.60E-07 |
| BCL2 | AC104126.1 | 0.352665024 | 6.78E-06 |
| BNIP1 | AC104126.1 | 0.34279481 | 1.26E-05 |
| CAPNS1 | AC104126.1 | 0.358312342 | 4.70E-06 |
| CFLAR | AC104126.1 | 0.340942326 | 1.42E-05 |
| FAS | AC104126.1 | 0.375096838 | 1.52E-06 |
| HDAC6 | AC104126.1 | 0.400284706 | 2.46E-07 |
| IKBKE | AC104126.1 | 0.440876407 | 9.40E-09 |
| MAP1LC3A | AC104126.1 | 0.394757085 | 3.72E-07 |
| MAPK8 | AC104126.1 | 0.390375233 | 5.13E-07 |
| NKX2-3 | AC104126.1 | 0.686749268 | 5.94E-23 |
| PEX14 | AC104126.1 | 0.351793332 | 7.17E-06 |
| PRKAB1 | AC104126.1 | 0.378778596 | 1.17E-06 |
| RAC1 | AC104126.1 | 0.366896578 | 2.66E-06 |
| TP53 | AC104126.1 | 0.339209881 | 1.58E-05 |
| TP73 | AC104126.1 | 0.636409822 | 5.63E-19 |
| TSC2 | AC104126.1 | 0.614008266 | 1.97E-17 |
| WDR45 | AC104126.1 | 0.377561534 | 1.28E-06 |
| ATG2B | AC064807.1 | 0.344297723 | 1.15E-05 |
| EEF2K | AC064807.1 | 0.31354343 | 7.11E-05 |
| HDAC6 | AC064807.1 | 0.300996887 | 0.00014141 |
| MTOR | AC064807.1 | 0.426023322 | 3.26E-08 |
| PARP1 | AC064807.1 | 0.305289718 | 0.000112145 |
| PRKAB1 | AC064807.1 | 0.30241783 | 0.000131015 |
| RB1CC1 | AC064807.1 | 0.406439281 | 1.54E-07 |
| TP53 | AC064807.1 | 0.323085698 | 4.13E-05 |
| TP73 | AC064807.1 | 0.309480287 | 8.91E-05 |
| ARNT | ZEB1-AS1 | 0.305527372 | 0.000110703 |
| CD46 | ZEB1-AS1 | 0.432075967 | 1.98E-08 |
| FOXO1 | ZEB1-AS1 | 0.419547569 | 5.51E-08 |
| GAA | ZEB1-AS1 | 0.417542034 | 6.47E-08 |
| GABARAPL1 | ZEB1-AS1 | 0.352678396 | 6.77E-06 |
| KLHL24 | ZEB1-AS1 | 0.440298043 | 9.87E-09 |
| NAMPT | ZEB1-AS1 | 0.316551663 | 6.00E-05 |
| NBR1 | ZEB1-AS1 | 0.317965225 | 5.54E-05 |
| PTK6 | ZEB1-AS1 | -0.333929336 | 2.17E-05 |
| SQSTM1 | ZEB1-AS1 | 0.30825766 | 9.53E-05 |
| ST13 | ZEB1-AS1 | 0.347225703 | 9.58E-06 |
| TP63 | ZEB1-AS1 | 0.325230076 | 3.64E-05 |
| ATG4B | TP53TG1 | 0.361094185 | 3.91E-06 |
| ATG4D | TP53TG1 | 0.323904043 | 3.93E-05 |
| BID | TP53TG1 | 0.444891379 | 6.64E-09 |
| BNIP1 | TP53TG1 | 0.398219604 | 2.87E-07 |
| CAPNS1 | TP53TG1 | 0.412221871 | 9.85E-08 |
| CLN3 | TP53TG1 | 0.316196977 | 6.12E-05 |
| EIF4G1 | TP53TG1 | -0.302096659 | 0.0001333 |
| FKBP1A | TP53TG1 | 0.347727627 | 9.28E-06 |
| KIF5B | TP53TG1 | -0.303127967 | 0.000126091 |
| MAP1LC3A | TP53TG1 | 0.364446231 | 3.13E-06 |
| PRKAB1 | TP53TG1 | 0.301643562 | 0.000136587 |
| ATG3 | AL391001.1 | 0.302620424 | 0.000129592 |
| ATG4A | AL391001.1 | 0.354011315 | 6.21E-06 |
| ATG4C | AL391001.1 | 0.365391962 | 2.94E-06 |
| ATIC | AL391001.1 | 0.516194154 | 6.22E-12 |
| BIRC5 | AL391001.1 | 0.440689683 | 9.55E-09 |
| CDKN1B | AL391001.1 | 0.350171897 | 7.95E-06 |
| GABARAP | AL391001.1 | 0.340087264 | 1.49E-05 |
| HDAC6 | AL391001.1 | 0.521541828 | 3.44E-12 |
| ITGA3 | AL391001.1 | -0.322625456 | 4.24E-05 |
| MAPK8IP1 | AL391001.1 | 0.756428524 | 5.24E-30 |
| MTMR14 | AL391001.1 | 0.353836746 | 6.29E-06 |
| PRKAR1A | AL391001.1 | 0.57214519 | 7.44E-15 |
| RAF1 | AL391001.1 | 0.307615596 | 9.88E-05 |
| ULK2 | AL391001.1 | 0.325065674 | 3.68E-05 |
| VAMP7 | AL391001.1 | 0.406994134 | 1.48E-07 |
| WDR45 | AL391001.1 | 0.352869866 | 6.69E-06 |
| CASP1 | AP001160.3 | 0.310853024 | 8.26E-05 |
| ATG10 | AC005332.5 | 0.342872711 | 1.26E-05 |
| ATG4B | AC005332.5 | 0.312711476 | 7.45E-05 |
| ATG4C | AC005332.5 | 0.304869664 | 0.000114737 |
| ATG4D | AC005332.5 | 0.336650032 | 1.84E-05 |
| BECN1 | AC005332.5 | 0.310755656 | 8.30E-05 |
| CAPN10 | AC005332.5 | 0.390346559 | 5.14E-07 |
| HDAC6 | AC005332.5 | 0.427247725 | 2.95E-08 |
| MAP2K7 | AC005332.5 | 0.314782669 | 6.63E-05 |
| MAPK8 | AC005332.5 | 0.341841289 | 1.34E-05 |
| MAPK9 | AC005332.5 | 0.32423424 | 3.86E-05 |
| MTMR14 | AC005332.5 | 0.315336392 | 6.43E-05 |
| NRG1 | AC005332.5 | -0.304279672 | 0.000118472 |
| PARP1 | AC005332.5 | 0.301329224 | 0.000138912 |
| PRKAB1 | AC005332.5 | 0.322432262 | 4.28E-05 |
| RAF1 | AC005332.5 | 0.389026607 | 5.66E-07 |
| RHEB | AC005332.5 | 0.379041942 | 1.15E-06 |
| STK11 | AC005332.5 | 0.337753171 | 1.72E-05 |
| TP73 | AC005332.5 | 0.377202213 | 1.31E-06 |
| ULK3 | AC005332.5 | 0.373720814 | 1.67E-06 |
| WDR45 | AC005332.5 | 0.320199132 | 4.87E-05 |
| ATG3 | SLC25A25-AS1 | 0.373605213 | 1.68E-06 |
| ATG4B | SLC25A25-AS1 | 0.387318287 | 6.40E-07 |
| ATG4D | SLC25A25-AS1 | 0.340262116 | 1.48E-05 |
| CAPN10 | SLC25A25-AS1 | 0.40317736 | 1.98E-07 |
| CDKN1B | SLC25A25-AS1 | 0.309494476 | 8.90E-05 |
| CX3CL1 | SLC25A25-AS1 | 0.321557547 | 4.51E-05 |
| CXCR4 | SLC25A25-AS1 | 0.496817055 | 4.86E-11 |
| DAPK2 | SLC25A25-AS1 | 0.340964136 | 1.42E-05 |
| HDAC6 | SLC25A25-AS1 | 0.42478736 | 3.61E-08 |
| IKBKB | SLC25A25-AS1 | 0.44488109 | 6.65E-09 |
| ITGA3 | SLC25A25-AS1 | -0.304736369 | 0.000115571 |
| KLHL24 | SLC25A25-AS1 | 0.302648967 | 0.000129393 |
| MAP2K7 | SLC25A25-AS1 | 0.52067153 | 3.80E-12 |
| PELP1 | SLC25A25-AS1 | 0.402536008 | 2.08E-07 |
| PIK3C3 | SLC25A25-AS1 | 0.365632151 | 2.89E-06 |
| PINK1 | SLC25A25-AS1 | -0.443857241 | 7.27E-09 |
| RAF1 | SLC25A25-AS1 | 0.408994223 | 1.27E-07 |
| STK11 | SLC25A25-AS1 | 0.390332094 | 5.14E-07 |
| TP53 | SLC25A25-AS1 | 0.356606432 | 5.25E-06 |
| TSC1 | SLC25A25-AS1 | 0.395724482 | 3.46E-07 |
| TSC2 | SLC25A25-AS1 | 0.366857938 | 2.66E-06 |
| ULK3 | SLC25A25-AS1 | 0.430006043 | 2.35E-08 |
| UVRAG | SLC25A25-AS1 | 0.332346752 | 2.39E-05 |
| WDR45 | SLC25A25-AS1 | 0.314003079 | 6.93E-05 |
| NRG1 | SUCLA2-AS1 | 0.321220419 | 4.59E-05 |
| NPC1 | PAX8-AS1 | 0.354663185 | 5.96E-06 |
| PEX3 | PAX8-AS1 | 0.304923134 | 0.000114404 |
| ATG12 | TMEM9B-AS1 | 0.313318188 | 7.20E-05 |
| ATG4C | TMEM9B-AS1 | 0.370861363 | 2.03E-06 |
| BCL2 | TMEM9B-AS1 | 0.508314138 | 1.46E-11 |
| CDKN1B | TMEM9B-AS1 | 0.384177426 | 8.02E-07 |
| CXCR4 | TMEM9B-AS1 | 0.506325884 | 1.80E-11 |
| DAPK1 | TMEM9B-AS1 | 0.333349993 | 2.25E-05 |
| EEF2 | TMEM9B-AS1 | 0.468382306 | 7.95E-10 |
| HDAC6 | TMEM9B-AS1 | 0.368667459 | 2.36E-06 |
| IKBKB | TMEM9B-AS1 | 0.364015661 | 3.22E-06 |
| KLHL24 | TMEM9B-AS1 | 0.317286127 | 5.75E-05 |
| MAP2K7 | TMEM9B-AS1 | 0.389578609 | 5.43E-07 |
| MAPK8 | TMEM9B-AS1 | 0.313181462 | 7.25E-05 |
| NKX2-3 | TMEM9B-AS1 | 0.347682323 | 9.31E-06 |
| PARP1 | TMEM9B-AS1 | 0.364105052 | 3.20E-06 |
| PIK3C3 | TMEM9B-AS1 | 0.300398051 | 0.000146017 |
| PRKAB1 | TMEM9B-AS1 | 0.341242141 | 1.39E-05 |
| RAF1 | TMEM9B-AS1 | 0.309449206 | 8.93E-05 |
| SIRT1 | TMEM9B-AS1 | 0.304790151 | 0.000115234 |
| TP53 | TMEM9B-AS1 | 0.441377784 | 9.00E-09 |
| TP73 | TMEM9B-AS1 | 0.346819847 | 9.83E-06 |
| TSC1 | TMEM9B-AS1 | 0.304956591 | 0.000114196 |
| TSC2 | TMEM9B-AS1 | 0.57564262 | 4.68E-15 |
| ULK2 | TMEM9B-AS1 | 0.301868245 | 0.000134948 |
| ULK3 | TMEM9B-AS1 | 0.374909099 | 1.54E-06 |
| UVRAG | TMEM9B-AS1 | 0.493182926 | 7.05E-11 |
| ATG16L2 | LINC01770 | 0.394880054 | 3.68E-07 |
| ATG9B | LINC01770 | 0.339109823 | 1.59E-05 |
| BCL2L1 | LINC01770 | 0.437012187 | 1.31E-08 |
| BID | LINC01770 | 0.562534156 | 2.59E-14 |
| CAPN10 | LINC01770 | 0.318534011 | 5.36E-05 |
| FKBP1B | LINC01770 | 0.365171103 | 2.98E-06 |
| GABARAPL2 | LINC01770 | 0.373999081 | 1.64E-06 |
| MAP1LC3A | LINC01770 | 0.387662075 | 6.24E-07 |
| PRKCQ | LINC01770 | 0.309214614 | 9.04E-05 |
| ATIC | ATP2A1-AS1 | 0.461170666 | 1.55E-09 |
| BAX | ATP2A1-AS1 | 0.450273735 | 4.14E-09 |
| BIRC5 | ATP2A1-AS1 | 0.624239619 | 4.02E-18 |
| CLN3 | ATP2A1-AS1 | 0.322382145 | 4.30E-05 |
| FKBP1A | ATP2A1-AS1 | 0.374113321 | 1.62E-06 |
| MAPK8IP1 | ATP2A1-AS1 | 0.631395803 | 1.28E-18 |
| MLST8 | ATP2A1-AS1 | 0.384500265 | 7.83E-07 |
| PRKAR1A | ATP2A1-AS1 | 0.333268066 | 2.26E-05 |
| WDR45 | ATP2A1-AS1 | 0.339561837 | 1.54E-05 |
| ARNT | AC103691.1 | -0.325735568 | 3.54E-05 |
| ATF4 | AC103691.1 | 0.540881458 | 3.72E-13 |
| ATG16L2 | AC103691.1 | 0.35396581 | 6.23E-06 |
| ATG2B | AC103691.1 | -0.384105292 | 8.06E-07 |
| ATG4B | AC103691.1 | 0.536375511 | 6.33E-13 |
| BAG1 | AC103691.1 | 0.325468667 | 3.59E-05 |
| BAG3 | AC103691.1 | -0.339378814 | 1.56E-05 |
| BAX | AC103691.1 | 0.489676573 | 1.01E-10 |
| BIRC5 | AC103691.1 | 0.337137138 | 1.79E-05 |
| BIRC6 | AC103691.1 | -0.397095879 | 3.13E-07 |
| BNIP1 | AC103691.1 | 0.496414243 | 5.07E-11 |
| CANX | AC103691.1 | -0.345729871 | 1.05E-05 |
| CAPN10 | AC103691.1 | 0.484469994 | 1.69E-10 |
| CAPN2 | AC103691.1 | -0.368263522 | 2.42E-06 |
| CASP1 | AC103691.1 | 0.352005211 | 7.07E-06 |
| DAPK2 | AC103691.1 | 0.309403039 | 8.95E-05 |
| DDIT3 | AC103691.1 | 0.378714959 | 1.18E-06 |
| DLC1 | AC103691.1 | -0.323413052 | 4.05E-05 |
| EIF4G1 | AC103691.1 | -0.350015554 | 8.03E-06 |
| FOXO1 | AC103691.1 | -0.326058388 | 3.47E-05 |
| GOPC | AC103691.1 | -0.336743842 | 1.83E-05 |
| ITGB1 | AC103691.1 | -0.338989711 | 1.60E-05 |
| KIF5B | AC103691.1 | -0.365818492 | 2.86E-06 |
| LAMP2 | AC103691.1 | -0.320263423 | 4.85E-05 |
| MAPK1 | AC103691.1 | -0.461542082 | 1.50E-09 |
| MTOR | AC103691.1 | -0.31909182 | 5.19E-05 |
| NCKAP1 | AC103691.1 | -0.396115175 | 3.36E-07 |
| NFKB1 | AC103691.1 | -0.310991779 | 8.20E-05 |
| NPC1 | AC103691.1 | -0.321874115 | 4.42E-05 |
| PELP1 | AC103691.1 | 0.369682789 | 2.20E-06 |
| PINK1 | AC103691.1 | -0.320505228 | 4.79E-05 |
| PTEN | AC103691.1 | -0.316445903 | 6.04E-05 |
| RAB24 | AC103691.1 | 0.335275293 | 2.00E-05 |
| RB1 | AC103691.1 | -0.33216308 | 2.42E-05 |
| STK11 | AC103691.1 | 0.304545271 | 0.000116777 |
| TM9SF1 | AC103691.1 | -0.315913336 | 6.22E-05 |
| ULK3 | AC103691.1 | 0.307644234 | 9.86E-05 |
| USP10 | AC103691.1 | -0.421128234 | 4.86E-08 |
| WDFY3 | AC103691.1 | -0.399076314 | 2.70E-07 |
| WDR45 | AC103691.1 | 0.481701461 | 2.22E-10 |
| BID | AC128709.2 | 0.41934595 | 5.60E-08 |
| MYC | AC128709.2 | 0.386960377 | 6.57E-07 |
| PPP1R15A | AC128709.2 | 0.305776798 | 0.000109209 |
| ARSB | AC007384.1 | 0.349261365 | 8.42E-06 |
| CCL2 | AC007384.1 | 0.333909972 | 2.18E-05 |
| CCR2 | AC007384.1 | 0.367867751 | 2.49E-06 |
| CFLAR | AC007384.1 | 0.319461745 | 5.08E-05 |
| CXCR4 | AC007384.1 | 0.456391562 | 2.40E-09 |
| DIRAS3 | AC007384.1 | 0.394977891 | 3.66E-07 |
| DRAM1 | AC007384.1 | 0.344211989 | 1.16E-05 |
| EDEM1 | AC007384.1 | 0.425002466 | 3.55E-08 |
| ERN1 | AC007384.1 | 0.307007605 | 0.000102105 |
| ITPR1 | AC007384.1 | 0.37581355 | 1.44E-06 |
| SERPINA1 | AC007384.1 | 0.399354159 | 2.64E-07 |
| UVRAG | AC007384.1 | 0.305605546 | 0.000110233 |
| BIRC6 | AC022364.1 | 0.38020633 | 1.06E-06 |
| BNIP1 | AC022364.1 | -0.336992211 | 1.81E-05 |
| GABARAPL1 | AC022364.1 | 0.398605682 | 2.79E-07 |
| NCKAP1 | AC022364.1 | 0.379893795 | 1.09E-06 |
| RAB24 | AC022364.1 | -0.322232731 | 4.33E-05 |
| TP63 | AC022364.1 | 0.330193894 | 2.72E-05 |
| USP10 | AC022364.1 | 0.300523235 | 0.000145043 |
| ARSB | AL355312.3 | -0.382789741 | 8.85E-07 |
| ATG9B | AL355312.3 | 0.405221641 | 1.69E-07 |
| CDKN1A | AL355312.3 | 0.346927269 | 9.76E-06 |
| CHMP2B | AL355312.3 | 0.401574696 | 2.23E-07 |
| EEF2 | AL355312.3 | -0.318783579 | 5.28E-05 |
| MAPK3 | AL355312.3 | 0.328424292 | 3.02E-05 |
| PTK6 | AL355312.3 | 0.368885151 | 2.32E-06 |
| RAB11A | AL355312.3 | 0.475672177 | 3.98E-10 |
| RAB24 | AL355312.3 | 0.420089499 | 5.28E-08 |
| RAC1 | AL355312.3 | 0.315549005 | 6.35E-05 |
| AMBRA1 | AL355353.1 | -0.337226941 | 1.78E-05 |
| ARNT | AL355353.1 | -0.358501554 | 4.64E-06 |
| ATF4 | AL355353.1 | 0.327361121 | 3.21E-05 |
| ATG4B | AL355353.1 | 0.335747406 | 1.95E-05 |
| BAG1 | AL355353.1 | 0.31367416 | 7.06E-05 |
| BAX | AL355353.1 | 0.519516143 | 4.31E-12 |
| BIRC5 | AL355353.1 | 0.43152339 | 2.07E-08 |
| BIRC6 | AL355353.1 | -0.341451371 | 1.37E-05 |
| BNIP1 | AL355353.1 | 0.44749694 | 5.29E-09 |
| CAMKK2 | AL355353.1 | -0.322517644 | 4.26E-05 |
| CANX | AL355353.1 | -0.379787484 | 1.09E-06 |
| DDIT3 | AL355353.1 | 0.322156193 | 4.35E-05 |
| EIF4EBP1 | AL355353.1 | 0.441827831 | 8.66E-09 |
| FKBP1A | AL355353.1 | 0.355387954 | 5.69E-06 |
| GOPC | AL355353.1 | -0.332794037 | 2.33E-05 |
| HGS | AL355353.1 | -0.381314681 | 9.82E-07 |
| KIF5B | AL355353.1 | -0.301794706 | 0.000135483 |
| MAPK1 | AL355353.1 | -0.406011033 | 1.59E-07 |
| MTOR | AL355353.1 | -0.31883633 | 5.27E-05 |
| NAF1 | AL355353.1 | -0.304701948 | 0.000115787 |
| NBR1 | AL355353.1 | -0.322288209 | 4.32E-05 |
| NCKAP1 | AL355353.1 | -0.337839511 | 1.71E-05 |
| NFKB1 | AL355353.1 | -0.361091065 | 3.91E-06 |
| RB1 | AL355353.1 | -0.359685655 | 4.29E-06 |
| SPNS1 | AL355353.1 | -0.310343231 | 8.50E-05 |
| WDFY3 | AL355353.1 | -0.301918063 | 0.000134587 |
| WDR45 | AL355353.1 | 0.384964187 | 7.58E-07 |
| SIRT2 | CFAP58-DT | 0.321437739 | 4.54E-05 |
| ARNT | NNT-AS1 | 0.40226424 | 2.12E-07 |
| ARSB | NNT-AS1 | 0.344538583 | 1.13E-05 |
| ATF6 | NNT-AS1 | 0.31499558 | 6.55E-05 |
| ATG10 | NNT-AS1 | 0.330284738 | 2.70E-05 |
| ATG4C | NNT-AS1 | 0.312206518 | 7.66E-05 |
| BAK1 | NNT-AS1 | -0.390333126 | 5.14E-07 |
| BCL2 | NNT-AS1 | 0.34468432 | 1.12E-05 |
| CASP3 | NNT-AS1 | 0.32502627 | 3.68E-05 |
| CD46 | NNT-AS1 | 0.388540468 | 5.86E-07 |
| CDKN1B | NNT-AS1 | 0.40417598 | 1.83E-07 |
| CXCR4 | NNT-AS1 | 0.337197564 | 1.78E-05 |
| DRAM1 | NNT-AS1 | 0.30649825 | 0.000104991 |
| EEF2 | NNT-AS1 | 0.315708141 | 6.29E-05 |
| EEF2K | NNT-AS1 | 0.331382866 | 2.53E-05 |
| HDAC6 | NNT-AS1 | 0.406843863 | 1.49E-07 |
| ITGA3 | NNT-AS1 | -0.313242144 | 7.23E-05 |
| KLHL24 | NNT-AS1 | 0.443580794 | 7.44E-09 |
| MAPK8 | NNT-AS1 | 0.364163379 | 3.19E-06 |
| MAPK9 | NNT-AS1 | 0.333736049 | 2.20E-05 |
| MBTPS2 | NNT-AS1 | 0.339210121 | 1.58E-05 |
| NBR1 | NNT-AS1 | 0.397102694 | 3.12E-07 |
| PARP1 | NNT-AS1 | 0.373685301 | 1.67E-06 |
| PIK3C3 | NNT-AS1 | 0.392128052 | 4.51E-07 |
| PTK6 | NNT-AS1 | -0.444042693 | 7.15E-09 |
| RAB33B | NNT-AS1 | 0.323812969 | 3.95E-05 |
| RB1CC1 | NNT-AS1 | 0.360301218 | 4.12E-06 |
| SIRT1 | NNT-AS1 | 0.436086363 | 1.41E-08 |
| SQSTM1 | NNT-AS1 | 0.409585867 | 1.21E-07 |
| ST13 | NNT-AS1 | 0.315958168 | 6.20E-05 |
| TP53 | NNT-AS1 | 0.344775415 | 1.12E-05 |
| TP73 | NNT-AS1 | 0.349729821 | 8.18E-06 |
| TSC2 | NNT-AS1 | 0.317339642 | 5.74E-05 |
| ULK2 | NNT-AS1 | 0.468207699 | 8.08E-10 |
| UVRAG | NNT-AS1 | 0.465734259 | 1.02E-09 |
| BAG1 | AC103706.1 | 0.330304955 | 2.70E-05 |
| BAX | AC103706.1 | 0.493538124 | 6.80E-11 |
| BIRC5 | AC103706.1 | 0.427381187 | 2.92E-08 |
| EIF4EBP1 | AC103706.1 | 0.386757228 | 6.66E-07 |
| FKBP1A | AC103706.1 | 0.3132987 | 7.21E-05 |
| WDR45 | AC103706.1 | 0.342903927 | 1.26E-05 |
| ATG16L1 | AC003070.1 | 0.300172501 | 0.000147788 |
| ATG16L2 | AC003070.1 | 0.42214866 | 4.47E-08 |
| ATG9B | AC003070.1 | 0.65184736 | 4.08E-20 |
| BECN1 | AC003070.1 | 0.36460679 | 3.10E-06 |
| CFLAR | AC003070.1 | 0.31304982 | 7.31E-05 |
| EIF2AK3 | AC003070.1 | 0.457521161 | 2.16E-09 |
| HGS | AC003070.1 | 0.341500188 | 1.37E-05 |
| IKBKB | AC003070.1 | 0.308076669 | 9.63E-05 |
| NFE2L2 | AC003070.1 | 0.344659618 | 1.13E-05 |
| RAB11A | AC003070.1 | 0.32811901 | 3.07E-05 |
| RAB1A | AC003070.1 | 0.371219605 | 1.98E-06 |
| RAB5A | AC003070.1 | 0.354995562 | 5.83E-06 |
| RAF1 | AC003070.1 | 0.476127804 | 3.81E-10 |
| RPS6KB1 | AC003070.1 | 0.426782521 | 3.07E-08 |
| TP53INP2 | AC003070.1 | 0.483578311 | 1.84E-10 |
| ULK3 | AC003070.1 | 0.429358174 | 2.48E-08 |
| ATG3 | AC131235.3 | 0.30544551 | 0.000111198 |
| EIF2AK3 | AC131235.3 | 0.362592264 | 3.54E-06 |
| EIF4G1 | AC131235.3 | 0.369734014 | 2.19E-06 |
| KLHL24 | AC131235.3 | 0.358234946 | 4.72E-06 |
| NFE2L2 | AC131235.3 | 0.347974637 | 9.14E-06 |
| RAF1 | AC131235.3 | 0.314844184 | 6.61E-05 |
| VAMP7 | AC131235.3 | 0.302354807 | 0.000131461 |
| WIPI1 | AC131235.3 | 0.308960208 | 9.17E-05 |
| ATG4D | AL035661.1 | 0.410253723 | 1.15E-07 |
| BAK1 | AL035661.1 | -0.313073376 | 7.30E-05 |
| CD46 | AL035661.1 | 0.346202503 | 1.02E-05 |
| EIF2AK3 | AL035661.1 | 0.389286468 | 5.55E-07 |
| FOXO3 | AL035661.1 | 0.304775835 | 0.000115324 |
| GABARAPL1 | AL035661.1 | 0.390622305 | 5.04E-07 |
| IKBKE | AL035661.1 | 0.342867683 | 1.26E-05 |
| ITGA3 | AL035661.1 | -0.365251348 | 2.97E-06 |
| KLHL24 | AL035661.1 | 0.507017649 | 1.67E-11 |
| MBTPS2 | AL035661.1 | 0.350981235 | 7.55E-06 |
| NBR1 | AL035661.1 | 0.360075998 | 4.19E-06 |
| NFE2L2 | AL035661.1 | 0.477669963 | 3.28E-10 |
| PRKAB1 | AL035661.1 | 0.343826234 | 1.19E-05 |
| RHEB | AL035661.1 | 0.360697096 | 4.02E-06 |
| TP63 | AL035661.1 | 0.387681317 | 6.23E-07 |
| ULK1 | AL035661.1 | 0.313268587 | 7.22E-05 |
| CASP4 | AC005229.4 | -0.348130868 | 9.05E-06 |
| DAPK1 | AC005229.4 | 0.317636393 | 5.64E-05 |
| ITGA3 | AC005229.4 | -0.357110548 | 5.08E-06 |
| MBTPS2 | AC005229.4 | 0.313153774 | 7.27E-05 |
| RHEB | AC005229.4 | 0.44136762 | 9.01E-09 |
| ARNT | LPP-AS2 | 0.420049327 | 5.30E-08 |
| ARSB | LPP-AS2 | 0.416999442 | 6.76E-08 |
| BIRC6 | LPP-AS2 | 0.33563512 | 1.96E-05 |
| CD46 | LPP-AS2 | 0.340519737 | 1.45E-05 |
| EIF2AK3 | LPP-AS2 | 0.466263752 | 9.69E-10 |
| EIF4G1 | LPP-AS2 | 0.489385718 | 1.03E-10 |
| FOXO1 | LPP-AS2 | 0.327831982 | 3.13E-05 |
| KLHL24 | LPP-AS2 | 0.575593876 | 4.71E-15 |
| MBTPS2 | LPP-AS2 | 0.442112008 | 8.45E-09 |
| NBR1 | LPP-AS2 | 0.324968236 | 3.70E-05 |
| NFE2L2 | LPP-AS2 | 0.400650803 | 2.39E-07 |
| PARP1 | LPP-AS2 | 0.339306579 | 1.57E-05 |
| PIK3R4 | LPP-AS2 | 0.355185176 | 5.76E-06 |
| PINK1 | LPP-AS2 | -0.307333314 | 0.000100299 |
| RB1CC1 | LPP-AS2 | 0.304570907 | 0.000116614 |
| TP63 | LPP-AS2 | 0.37302182 | 1.75E-06 |
| ATG4C | AC079610.2 | 0.318924026 | 5.24E-05 |
| CDKN1B | AC079610.2 | 0.300521784 | 0.000145054 |
| EIF2AK3 | AC079610.2 | 0.341231625 | 1.39E-05 |
| HDAC1 | AC079610.2 | 0.384058596 | 8.08E-07 |
| HDAC6 | AC079610.2 | 0.389683903 | 5.39E-07 |
| IKBKE | AC079610.2 | 0.32729991 | 3.23E-05 |
| ITGA3 | AC079610.2 | -0.310831869 | 8.27E-05 |
| MAPK8 | AC079610.2 | 0.302263792 | 0.000132106 |
| PRKAB1 | AC079610.2 | 0.447832734 | 5.14E-09 |
| TP53INP2 | AC079610.2 | 0.352066199 | 7.04E-06 |
| ATG16L2 | LINC00106 | 0.396426111 | 3.29E-07 |
| CAPN10 | LINC00106 | 0.379449864 | 1.12E-06 |
| CDKN1B | LINC00106 | 0.332715105 | 2.34E-05 |
| HDAC6 | LINC00106 | 0.309543405 | 8.88E-05 |
| IKBKB | LINC00106 | 0.349123087 | 8.50E-06 |
| ULK3 | LINC00106 | 0.355564535 | 5.62E-06 |
| ATF4 | SNHG20 | 0.350204677 | 7.93E-06 |
| ATG16L2 | SNHG20 | 0.438025759 | 1.20E-08 |
| ATG4B | SNHG20 | 0.362140908 | 3.65E-06 |
| ATG4D | SNHG20 | 0.315018237 | 6.54E-05 |
| ATG9B | SNHG20 | 0.412087899 | 9.95E-08 |
| ATIC | SNHG20 | 0.376891351 | 1.34E-06 |
| BIRC5 | SNHG20 | 0.333782427 | 2.19E-05 |
| CAPN10 | SNHG20 | 0.497661902 | 4.46E-11 |
| DAPK2 | SNHG20 | 0.342374974 | 1.30E-05 |
| HDAC6 | SNHG20 | 0.327545288 | 3.18E-05 |
| IKBKB | SNHG20 | 0.350209135 | 7.93E-06 |
| ITGA3 | SNHG20 | -0.385792309 | 7.14E-07 |
| MAP2K7 | SNHG20 | 0.319380046 | 5.11E-05 |
| MAPK8IP1 | SNHG20 | 0.355232621 | 5.74E-06 |
| PELP1 | SNHG20 | 0.382967746 | 8.74E-07 |
| PINK1 | SNHG20 | -0.377986437 | 1.24E-06 |
| RAF1 | SNHG20 | 0.309481635 | 8.91E-05 |
| RHEB | SNHG20 | 0.386189121 | 6.94E-07 |
| RPS6KB1 | SNHG20 | 0.334096099 | 2.15E-05 |
| ULK3 | SNHG20 | 0.443606925 | 7.42E-09 |
| VAMP3 | SNHG20 | -0.306721879 | 0.000103714 |
| WDR45 | SNHG20 | 0.339863329 | 1.51E-05 |
| WIPI1 | SNHG20 | 0.318300657 | 5.43E-05 |
| AMBRA1 | AC102953.2 | 0.310998702 | 8.19E-05 |
| CXCR4 | AC102953.2 | 0.369569382 | 2.22E-06 |
| DAPK1 | AC102953.2 | 0.346934071 | 9.76E-06 |
| EEF2 | AC102953.2 | 0.348098744 | 9.07E-06 |
| HDAC6 | AC102953.2 | 0.305949823 | 0.000108183 |
| IKBKE | AC102953.2 | 0.302705559 | 0.000128999 |
| MAP2K7 | AC102953.2 | 0.406030725 | 1.59E-07 |
| PARP1 | AC102953.2 | 0.389041111 | 5.65E-07 |
| PTK6 | AC102953.2 | -0.339368839 | 1.56E-05 |
| RAF1 | AC102953.2 | 0.321011852 | 4.65E-05 |
| STK11 | AC102953.2 | 0.312561858 | 7.51E-05 |
| TP53 | AC102953.2 | 0.381883611 | 9.44E-07 |
| TP73 | AC102953.2 | 0.441631673 | 8.81E-09 |
| TSC2 | AC102953.2 | 0.449452989 | 4.45E-09 |
| UVRAG | AC102953.2 | 0.306673601 | 0.000103989 |
| ARNT | AL035681.1 | 0.341269703 | 1.39E-05 |
| BIRC6 | AL035681.1 | 0.331406319 | 2.53E-05 |
| CASP3 | AL035681.1 | 0.300427881 | 0.000145784 |
| EIF2AK3 | AL035681.1 | 0.445211378 | 6.46E-09 |
| EIF4G1 | AL035681.1 | 0.360273701 | 4.13E-06 |
| FOXO3 | AL035681.1 | 0.395042285 | 3.64E-07 |
| ITGA3 | AL035681.1 | -0.326006831 | 3.48E-05 |
| KIF5B | AL035681.1 | 0.346200434 | 1.02E-05 |
| KLHL24 | AL035681.1 | 0.462083489 | 1.43E-09 |
| NBR1 | AL035681.1 | 0.343131974 | 1.24E-05 |
| NFE2L2 | AL035681.1 | 0.530417481 | 1.26E-12 |
| PIK3R4 | AL035681.1 | 0.342979866 | 1.25E-05 |
| RAB5A | AL035681.1 | 0.309160414 | 9.07E-05 |
| RB1CC1 | AL035681.1 | 0.328951773 | 2.93E-05 |
| RHEB | AL035681.1 | 0.342182759 | 1.31E-05 |
| RPS6KB1 | AL035681.1 | 0.400400256 | 2.44E-07 |
| SQSTM1 | AL035681.1 | 0.321600945 | 4.50E-05 |
| UVRAG | AL035681.1 | 0.335234905 | 2.01E-05 |
| ATG4C | AL354766.2 | 0.327686429 | 3.15E-05 |
| BCL2 | AL354766.2 | 0.31231932 | 7.61E-05 |
| CASP3 | AL354766.2 | 0.312759016 | 7.43E-05 |
| HDAC6 | AL354766.2 | 0.361301107 | 3.86E-06 |
| IKBKE | AL354766.2 | 0.394809014 | 3.70E-07 |
| MAPK8 | AL354766.2 | 0.381012336 | 1.00E-06 |
| NKX2-3 | AL354766.2 | 0.355022597 | 5.82E-06 |
| RAB33B | AL354766.2 | 0.317438415 | 5.71E-05 |
| WDR45 | AL354766.2 | 0.330809584 | 2.62E-05 |
| CASP8 | AL031123.1 | 0.320458559 | 4.80E-05 |
| CFLAR | AL031123.1 | 0.393805409 | 3.99E-07 |
| VAMP3 | AL031123.1 | 0.536037239 | 6.59E-13 |
| ATG16L2 | LINC00685 | 0.316853127 | 5.90E-05 |
| ATG4B | LINC00685 | 0.367079873 | 2.62E-06 |
| CAPN10 | LINC00685 | 0.382626005 | 8.95E-07 |
| CDKN1B | LINC00685 | 0.312766338 | 7.42E-05 |
| CXCR4 | LINC00685 | 0.476445106 | 3.69E-10 |
| DAPK2 | LINC00685 | 0.316670985 | 5.96E-05 |
| HDAC6 | LINC00685 | 0.417298277 | 6.60E-08 |
| IKBKB | LINC00685 | 0.354987783 | 5.83E-06 |
| MTMR14 | LINC00685 | 0.348135618 | 9.04E-06 |
| PELP1 | LINC00685 | 0.331553208 | 2.51E-05 |
| PINK1 | LINC00685 | -0.304641376 | 0.000116169 |
| RAF1 | LINC00685 | 0.318156868 | 5.48E-05 |
| STK11 | LINC00685 | 0.377254657 | 1.31E-06 |
| TP53 | LINC00685 | 0.319679054 | 5.02E-05 |
| TSC2 | LINC00685 | 0.333404889 | 2.24E-05 |
| ULK2 | LINC00685 | 0.314434576 | 6.76E-05 |
| ULK3 | LINC00685 | 0.349309727 | 8.40E-06 |
| WDR45 | LINC00685 | 0.350440937 | 7.81E-06 |
| ATG3 | LINC02004 | 0.495939011 | 5.32E-11 |
| PIK3R4 | LINC02004 | 0.384906836 | 7.61E-07 |
| RAF1 | LINC02004 | 0.389204818 | 5.58E-07 |
| ATF6 | AL683887.1 | 0.394963982 | 3.66E-07 |
| EIF4G1 | AL683887.1 | 0.328737134 | 2.96E-05 |
| FOXO1 | AL683887.1 | 0.326188256 | 3.44E-05 |
| MBTPS2 | AL683887.1 | 0.307588147 | 9.89E-05 |
| NCKAP1 | AL683887.1 | 0.358692768 | 4.58E-06 |
| ST13 | AL683887.1 | 0.341314418 | 1.39E-05 |
| TP63 | AL683887.1 | 0.337924875 | 1.71E-05 |
| USP10 | AL683887.1 | 0.382191051 | 9.23E-07 |
| ARNT | LINC02035 | 0.35143892 | 7.33E-06 |
| ATG3 | LINC02035 | 0.396197381 | 3.34E-07 |
| BECN1 | LINC02035 | 0.351873503 | 7.13E-06 |
| CD46 | LINC02035 | 0.438618416 | 1.14E-08 |
| EEF2K | LINC02035 | 0.426278333 | 3.20E-08 |
| EIF2AK3 | LINC02035 | 0.332736652 | 2.34E-05 |
| GAA | LINC02035 | 0.311279608 | 8.07E-05 |
| KLHL24 | LINC02035 | 0.333429305 | 2.24E-05 |
| MAP2K7 | LINC02035 | 0.319787929 | 4.99E-05 |
| MAPK8 | LINC02035 | 0.371072254 | 2.00E-06 |
| NBR1 | LINC02035 | 0.365592358 | 2.90E-06 |
| PIK3R4 | LINC02035 | 0.52060536 | 3.82E-12 |
| RAB7A | LINC02035 | 0.374712302 | 1.56E-06 |
| RAF1 | LINC02035 | 0.312692235 | 7.46E-05 |
| TP73 | LINC02035 | 0.338486174 | 1.65E-05 |
| TSC1 | LINC02035 | 0.384250405 | 7.97E-07 |
| ULK1 | LINC02035 | 0.364048714 | 3.22E-06 |
| ATG3 | AC007996.1 | 0.416578602 | 6.99E-08 |
| ATG4D | AC007996.1 | 0.313846935 | 6.99E-05 |
| ATIC | AC007996.1 | 0.309153727 | 9.07E-05 |
| CAPN10 | AC007996.1 | 0.363493825 | 3.34E-06 |
| CD46 | AC007996.1 | 0.308660903 | 9.32E-05 |
| CDKN1B | AC007996.1 | 0.357928749 | 4.82E-06 |
| EIF2AK3 | AC007996.1 | 0.413029346 | 9.25E-08 |
| FOXO3 | AC007996.1 | 0.312653281 | 7.47E-05 |
| HDAC6 | AC007996.1 | 0.389780999 | 5.35E-07 |
| ITGA3 | AC007996.1 | -0.413322443 | 9.04E-08 |
| KLHL24 | AC007996.1 | 0.362547422 | 3.55E-06 |
| MAP2K7 | AC007996.1 | 0.333484246 | 2.23E-05 |
| MAPK8 | AC007996.1 | 0.300244368 | 0.000147222 |
| NFE2L2 | AC007996.1 | 0.428821056 | 2.59E-08 |
| PARP1 | AC007996.1 | 0.301434363 | 0.00013813 |
| PELP1 | AC007996.1 | 0.304695305 | 0.000115829 |
| PIK3R4 | AC007996.1 | 0.377160731 | 1.32E-06 |
| PINK1 | AC007996.1 | -0.433182347 | 1.80E-08 |
| PRKAB1 | AC007996.1 | 0.313811388 | 7.00E-05 |
| RAF1 | AC007996.1 | 0.364588772 | 3.10E-06 |
| RB1CC1 | AC007996.1 | 0.358436849 | 4.66E-06 |
| RHEB | AC007996.1 | 0.322793882 | 4.20E-05 |
| RPS6KB1 | AC007996.1 | 0.400767181 | 2.37E-07 |
| TP53 | AC007996.1 | 0.373953477 | 1.64E-06 |
| ULK3 | AC007996.1 | 0.367333384 | 2.58E-06 |
| VAMP3 | AC007996.1 | -0.356221199 | 5.39E-06 |
| ATG9B | FALEC | 0.752846276 | 1.38E-29 |
| CHMP2B | FALEC | 0.358269121 | 4.71E-06 |
| PTK6 | FALEC | 0.330033818 | 2.74E-05 |
| RAB11A | FALEC | 0.432382097 | 1.93E-08 |
| RAB1A | FALEC | 0.32573962 | 3.53E-05 |
| RAB5A | FALEC | 0.323633803 | 4.00E-05 |
| RAF1 | FALEC | 0.321015898 | 4.65E-05 |
| TP53INP2 | FALEC | 0.5709106 | 8.75E-15 |
| ATG16L2 | AC073335.2 | 0.337875344 | 1.71E-05 |
| CAPN10 | AC073335.2 | 0.361014438 | 3.93E-06 |
| CASP8 | AC073335.2 | 0.319250639 | 5.15E-05 |
| EIF2AK3 | AC073335.2 | 0.369441944 | 2.24E-06 |
| HDAC6 | AC073335.2 | 0.391776961 | 4.63E-07 |
| IKBKB | AC073335.2 | 0.348392808 | 8.90E-06 |
| ITGA3 | AC073335.2 | -0.311729885 | 7.87E-05 |
| KLHL24 | AC073335.2 | 0.368648754 | 2.36E-06 |
| MAP2K7 | AC073335.2 | 0.368115739 | 2.45E-06 |
| ULK3 | AC073335.2 | 0.391071364 | 4.87E-07 |
| UVRAG | AC073335.2 | 0.375082493 | 1.52E-06 |
| WIPI1 | AC073335.2 | 0.336257026 | 1.89E-05 |
| ATG4B | AC021016.2 | 0.412005271 | 1.00E-07 |
| ATG4C | AC021016.2 | 0.397264054 | 3.09E-07 |
| ATG7 | AC021016.2 | 0.381787911 | 9.50E-07 |
| CAPN10 | AC021016.2 | 0.401960203 | 2.17E-07 |
| CX3CL1 | AC021016.2 | 0.321070873 | 4.63E-05 |
| CXCR4 | AC021016.2 | 0.533558754 | 8.79E-13 |
| FAS | AC021016.2 | 0.327573993 | 3.17E-05 |
| HIF1A | AC021016.2 | -0.371602657 | 1.93E-06 |
| IKBKE | AC021016.2 | 0.333442813 | 2.24E-05 |
| MAPK8 | AC021016.2 | 0.305366749 | 0.000111676 |
| MTMR14 | AC021016.2 | 0.430816665 | 2.20E-08 |
| NCKAP1 | AC021016.2 | -0.304303772 | 0.000118317 |
| NKX2-3 | AC021016.2 | 0.300217645 | 0.000147432 |
| PEX14 | AC021016.2 | 0.327272659 | 3.23E-05 |
| RAF1 | AC021016.2 | 0.334296997 | 2.13E-05 |
| SH3GLB1 | AC021016.2 | 0.316500108 | 6.02E-05 |
| STK11 | AC021016.2 | 0.416206913 | 7.20E-08 |
| TP53 | AC021016.2 | 0.437398115 | 1.26E-08 |
| TP73 | AC021016.2 | 0.435245091 | 1.52E-08 |
| TSC2 | AC021016.2 | 0.543402653 | 2.76E-13 |
| BCL2 | TNFRSF10A-AS1 | 0.369779699 | 2.19E-06 |
| CASP3 | TNFRSF10A-AS1 | 0.340567715 | 1.45E-05 |
| CX3CL1 | TNFRSF10A-AS1 | 0.342325863 | 1.30E-05 |
| FAS | TNFRSF10A-AS1 | 0.332697165 | 2.34E-05 |
| LAMP1 | TNFRSF10A-AS1 | -0.315628824 | 6.32E-05 |
| MAPK8 | TNFRSF10A-AS1 | 0.389170014 | 5.60E-07 |
| NAF1 | TNFRSF10A-AS1 | 0.401159155 | 2.30E-07 |
| NFKB1 | TNFRSF10A-AS1 | 0.359413903 | 4.37E-06 |
| NKX2-3 | TNFRSF10A-AS1 | 0.347333683 | 9.51E-06 |
| RAB33B | TNFRSF10A-AS1 | 0.303764562 | 0.000121825 |
| RAF1 | TNFRSF10A-AS1 | 0.357127127 | 5.08E-06 |
| RPS6KB1 | TNFRSF10A-AS1 | 0.326446338 | 3.39E-05 |
| TP53 | TNFRSF10A-AS1 | 0.357399472 | 4.99E-06 |
| WIPI2 | TNFRSF10A-AS1 | -0.311354671 | 8.03E-05 |
| EGFR | ZBED5-AS1 | 0.377477803 | 1.29E-06 |
| MBTPS2 | ZBED5-AS1 | 0.312543403 | 7.52E-05 |
| ATG5 | AC006305.1 | 0.307552573 | 9.91E-05 |
| VAMP7 | AC006305.1 | 0.317313672 | 5.75E-05 |
| ATG9B | AC090204.1 | 0.319099137 | 5.19E-05 |
| BID | AC090204.1 | 0.302180159 | 0.000132703 |
| FKBP1B | AC090204.1 | 0.323587374 | 4.01E-05 |
| CASP1 | LINC01705 | 0.454729591 | 2.78E-09 |
| NFE2L2 | AC128709.3 | 0.305678376 | 0.000109796 |
| BID | AC124067.2 | 0.465327218 | 1.06E-09 |
| GABARAPL1 | AC124067.2 | 0.370230705 | 2.12E-06 |
| NFE2L2 | AC124067.2 | 0.338405113 | 1.66E-05 |
| ARNT | AC025171.1 | 0.338565407 | 1.64E-05 |
| ATG2A | AC025171.1 | 0.317833767 | 5.58E-05 |
| ATG2B | AC025171.1 | 0.301223471 | 0.000139702 |
| CD46 | AC025171.1 | 0.341869689 | 1.34E-05 |
| CDKN1B | AC025171.1 | 0.371544858 | 1.94E-06 |
| EEF2K | AC025171.1 | 0.398700845 | 2.77E-07 |
| HDAC6 | AC025171.1 | 0.35116383 | 7.46E-06 |
| MAPK8 | AC025171.1 | 0.344019964 | 1.17E-05 |
| MTOR | AC025171.1 | 0.361375325 | 3.84E-06 |
| NKX2-3 | AC025171.1 | 0.33924128 | 1.57E-05 |
| TP53 | AC025171.1 | 0.300240423 | 0.000147253 |
| TP73 | AC025171.1 | 0.479062848 | 2.87E-10 |
| TSC1 | AC025171.1 | 0.338441682 | 1.65E-05 |
| ARSB | AC145098.1 | 0.501138954 | 3.11E-11 |
| ATG4C | AC145098.1 | 0.337018114 | 1.80E-05 |
| BCL2 | AC145098.1 | 0.313037296 | 7.31E-05 |
| BIRC6 | AC145098.1 | 0.355643922 | 5.59E-06 |
| CANX | AC145098.1 | 0.334918808 | 2.05E-05 |
| CCR2 | AC145098.1 | 0.724125118 | 1.81E-26 |
| CXCR4 | AC145098.1 | 0.357416307 | 4.98E-06 |
| DAPK1 | AC145098.1 | 0.322366702 | 4.30E-05 |
| DRAM1 | AC145098.1 | 0.438912198 | 1.11E-08 |
| EDEM1 | AC145098.1 | 0.425155762 | 3.50E-08 |
| EEF2K | AC145098.1 | 0.354447079 | 6.04E-06 |
| ERN1 | AC145098.1 | 0.338376529 | 1.66E-05 |
| IFNG | AC145098.1 | 0.382844705 | 8.81E-07 |
| ITPR1 | AC145098.1 | 0.599824084 | 1.62E-16 |
| MAPK9 | AC145098.1 | 0.30100443 | 0.000141353 |
| MTOR | AC145098.1 | 0.322391146 | 4.29E-05 |
| PARP1 | AC145098.1 | 0.347120421 | 9.64E-06 |
| PRKCD | AC145098.1 | 0.392703411 | 4.32E-07 |
| RGS19 | AC145098.1 | 0.405771263 | 1.62E-07 |
| SIRT1 | AC145098.1 | 0.316593943 | 5.99E-05 |
| TP53 | AC145098.1 | 0.319011759 | 5.22E-05 |
| ULK2 | AC145098.1 | 0.346475318 | 1.00E-05 |
| ATG16L2 | AC084018.1 | 0.352610462 | 6.80E-06 |
| ATG4B | AC084018.1 | 0.305918213 | 0.00010837 |
| CAPN10 | AC084018.1 | 0.421530073 | 4.70E-08 |
| CDKN1B | AC084018.1 | 0.341340464 | 1.38E-05 |
| CXCR4 | AC084018.1 | 0.538067719 | 5.19E-13 |
| DAPK2 | AC084018.1 | 0.358757843 | 4.56E-06 |
| HDAC6 | AC084018.1 | 0.388417418 | 5.91E-07 |
| IKBKB | AC084018.1 | 0.436669044 | 1.35E-08 |
| MAP2K7 | AC084018.1 | 0.411804315 | 1.02E-07 |
| PINK1 | AC084018.1 | -0.314819892 | 6.62E-05 |
| RAF1 | AC084018.1 | 0.360895271 | 3.97E-06 |
| TP53 | AC084018.1 | 0.313216804 | 7.24E-05 |
| TSC1 | AC084018.1 | 0.419571711 | 5.50E-08 |
| TSC2 | AC084018.1 | 0.464763265 | 1.11E-09 |
| ULK3 | AC084018.1 | 0.452189641 | 3.49E-09 |
| UVRAG | AC084018.1 | 0.396068833 | 3.37E-07 |
| ATG3 | DICER1-AS1 | 0.312159541 | 7.68E-05 |
| CLN3 | DICER1-AS1 | 0.346776534 | 9.85E-06 |
| HDAC6 | DICER1-AS1 | 0.348096075 | 9.07E-06 |
| MAP2K7 | DICER1-AS1 | 0.328443892 | 3.01E-05 |
| WDR45 | DICER1-AS1 | 0.428336886 | 2.70E-08 |
| CDKN1A | AC039056.2 | 0.305092237 | 0.000113357 |
| DNAJB1 | AC039056.2 | 0.322820847 | 4.19E-05 |
| GABARAPL2 | AC039056.2 | 0.324554373 | 3.79E-05 |
| PPP1R15A | AC039056.2 | 0.457623471 | 2.14E-09 |
| VEGFA | AC039056.2 | 0.458578805 | 1.97E-09 |
| GAA | AC008537.2 | 0.35032623 | 7.87E-06 |
| DIRAS3 | LINC02178 | 0.896101459 | 7.64E-56 |
| FKBP1B | LINC02178 | 0.426151813 | 3.23E-08 |
| PRKCQ | LINC02178 | 0.343304207 | 1.22E-05 |
| ATIC | AC019069.1 | 0.349432542 | 8.33E-06 |
| ATF4 | AL590560.1 | 0.300813636 | 0.000142805 |
| ATG12 | AL590560.1 | 0.301124568 | 0.000140445 |
| ATG16L2 | AL590560.1 | 0.336475584 | 1.86E-05 |
| ATG4B | AL590560.1 | 0.391768233 | 4.63E-07 |
| BAG3 | AL590560.1 | -0.343281907 | 1.23E-05 |
| BAX | AL590560.1 | 0.332677393 | 2.34E-05 |
| CAPN10 | AL590560.1 | 0.536839205 | 6.00E-13 |
| CXCR4 | AL590560.1 | 0.393951169 | 3.95E-07 |
| DAPK2 | AL590560.1 | 0.33902369 | 1.60E-05 |
| DIRAS3 | AL590560.1 | 0.369795072 | 2.18E-06 |
| GABARAP | AL590560.1 | 0.349217024 | 8.45E-06 |
| MAP2K7 | AL590560.1 | 0.309328388 | 8.99E-05 |
| MTMR14 | AL590560.1 | 0.300568855 | 0.000144689 |
| PELP1 | AL590560.1 | 0.348827634 | 8.66E-06 |
| PINK1 | AL590560.1 | -0.310640964 | 8.36E-05 |
| ULK3 | AL590560.1 | 0.39786137 | 2.95E-07 |
| WDR45 | AL590560.1 | 0.370124467 | 2.14E-06 |
| ATF4 | AC005261.1 | 0.315515088 | 6.36E-05 |
| ATG16L2 | AC005261.1 | 0.365478465 | 2.92E-06 |
| ATG3 | AC005261.1 | 0.368084414 | 2.45E-06 |
| ATG4B | AC005261.1 | 0.487362231 | 1.27E-10 |
| BAX | AC005261.1 | 0.310208796 | 8.56E-05 |
| CAPN10 | AC005261.1 | 0.416160824 | 7.22E-08 |
| CASP3 | AC005261.1 | 0.300280102 | 0.000146941 |
| DAPK2 | AC005261.1 | 0.46317207 | 1.29E-09 |
| MAP2K7 | AC005261.1 | 0.307895492 | 9.73E-05 |
| PEX14 | AC005261.1 | 0.316754153 | 5.93E-05 |
| RAB24 | AC005261.1 | 0.422067683 | 4.50E-08 |
| STK11 | AC005261.1 | 0.403543429 | 1.92E-07 |
| ULK3 | AC005261.1 | 0.409169421 | 1.25E-07 |
| WDR45 | AC005261.1 | 0.393287866 | 4.14E-07 |
| ARSB | FZD10-DT | 0.360148237 | 4.17E-06 |
| BAK1 | FZD10-DT | -0.314311049 | 6.81E-05 |
| CD46 | FZD10-DT | 0.340241971 | 1.48E-05 |
| CDKN1B | FZD10-DT | 0.363936952 | 3.24E-06 |
| DIRAS3 | FZD10-DT | 0.302473883 | 0.00013062 |
| EIF2AK3 | FZD10-DT | 0.309058139 | 9.12E-05 |
| FOXO1 | FZD10-DT | 0.354531919 | 6.01E-06 |
| FOXO3 | FZD10-DT | 0.307344476 | 0.000100237 |
| GABARAPL1 | FZD10-DT | 0.391739919 | 4.64E-07 |
| ITGA3 | FZD10-DT | -0.305872443 | 0.000108641 |
| KLHL24 | FZD10-DT | 0.412912784 | 9.33E-08 |
| NBR1 | FZD10-DT | 0.339880902 | 1.51E-05 |
| NFE2L2 | FZD10-DT | 0.384801966 | 7.67E-07 |
| PTK6 | FZD10-DT | -0.304581564 | 0.000116547 |
| RB1CC1 | FZD10-DT | 0.431663531 | 2.05E-08 |
| TP63 | FZD10-DT | 0.342490195 | 1.29E-05 |
| ATG16L2 | POLR2J4 | 0.32084659 | 4.69E-05 |
| ATG2B | POLR2J4 | -0.356025582 | 5.46E-06 |
| ATG4B | POLR2J4 | 0.488896074 | 1.09E-10 |
| ATG7 | POLR2J4 | 0.374300003 | 1.60E-06 |
| BAX | POLR2J4 | 0.357396142 | 4.99E-06 |
| BNIP1 | POLR2J4 | 0.366217203 | 2.78E-06 |
| CAPN10 | POLR2J4 | 0.412619653 | 9.55E-08 |
| CAPNS1 | POLR2J4 | 0.374034459 | 1.63E-06 |
| CDKN2A | POLR2J4 | 0.526088685 | 2.07E-12 |
| HIF1A | POLR2J4 | -0.323859018 | 3.94E-05 |
| IKBKE | POLR2J4 | 0.379330402 | 1.13E-06 |
| MAP1LC3A | POLR2J4 | 0.399021976 | 2.71E-07 |
| MAP1LC3B | POLR2J4 | -0.314101829 | 6.89E-05 |
| MTMR14 | POLR2J4 | 0.40888637 | 1.28E-07 |
| PINK1 | POLR2J4 | -0.333413676 | 2.24E-05 |
| PRKAB1 | POLR2J4 | 0.379556336 | 1.11E-06 |
| RAB24 | POLR2J4 | 0.379093284 | 1.15E-06 |
| RAC1 | POLR2J4 | 0.472552392 | 5.36E-10 |
| RAF1 | POLR2J4 | 0.302808984 | 0.000128281 |
| STK11 | POLR2J4 | 0.397195531 | 3.10E-07 |
| TSC2 | POLR2J4 | 0.307736398 | 9.81E-05 |
| USP10 | POLR2J4 | -0.345717328 | 1.05E-05 |
| WDR45 | POLR2J4 | 0.461585038 | 1.49E-09 |
| ZFYVE1 | POLR2J4 | -0.308635466 | 9.34E-05 |
| ARNT | AC004241.3 | -0.454056607 | 2.96E-09 |
| ARSB | AC004241.3 | -0.366154873 | 2.79E-06 |
| ATF4 | AC004241.3 | 0.509428087 | 1.29E-11 |
| ATF6 | AC004241.3 | -0.350286261 | 7.89E-06 |
| ATG16L2 | AC004241.3 | 0.343180934 | 1.23E-05 |
| ATG4B | AC004241.3 | 0.397303688 | 3.08E-07 |
| ATIC | AC004241.3 | 0.33764234 | 1.74E-05 |
| BAX | AC004241.3 | 0.310398942 | 8.47E-05 |
| BIRC5 | AC004241.3 | 0.340647375 | 1.44E-05 |
| BIRC6 | AC004241.3 | -0.399184031 | 2.67E-07 |
| BNIP1 | AC004241.3 | 0.421424382 | 4.74E-08 |
| CAPN10 | AC004241.3 | 0.407854688 | 1.38E-07 |
| CLN3 | AC004241.3 | 0.318917108 | 5.24E-05 |
| DLC1 | AC004241.3 | -0.324041932 | 3.90E-05 |
| EEF2K | AC004241.3 | -0.37073412 | 2.05E-06 |
| FKBP1B | AC004241.3 | 0.374439009 | 1.59E-06 |
| FOXO1 | AC004241.3 | -0.393613264 | 4.04E-07 |
| FOXO3 | AC004241.3 | -0.341703495 | 1.35E-05 |
| GABARAPL2 | AC004241.3 | 0.43183247 | 2.02E-08 |
| GOPC | AC004241.3 | -0.319844435 | 4.97E-05 |
| LAMP2 | AC004241.3 | -0.342478307 | 1.29E-05 |
| MAP1LC3A | AC004241.3 | 0.326156882 | 3.45E-05 |
| MAPK1 | AC004241.3 | -0.35898323 | 4.50E-06 |
| MLST8 | AC004241.3 | 0.328017397 | 3.09E-05 |
| MTOR | AC004241.3 | -0.356490953 | 5.29E-06 |
| PIK3R4 | AC004241.3 | -0.400127143 | 2.49E-07 |
| RB1 | AC004241.3 | -0.320766864 | 4.72E-05 |
| WDR45 | AC004241.3 | 0.403585157 | 1.92E-07 |
| EIF2AK3 | AC006026.3 | 0.329321346 | 2.86E-05 |
| RAB11A | AC006026.3 | 0.311423089 | 8.00E-05 |
| TP53INP2 | AC006026.3 | 0.408051148 | 1.36E-07 |
| ULK3 | AC006026.3 | 0.31883301 | 5.27E-05 |
| ATG3 | AC022075.1 | 0.340497479 | 1.46E-05 |
| ATG4D | AC022075.1 | 0.389556528 | 5.44E-07 |
| EIF2AK3 | AC022075.1 | 0.372282223 | 1.84E-06 |
| GABARAPL1 | AC022075.1 | 0.357524109 | 4.95E-06 |
| ITGA3 | AC022075.1 | -0.319459197 | 5.08E-05 |
| KLHL24 | AC022075.1 | 0.364988457 | 3.02E-06 |
| MAP2K7 | AC022075.1 | 0.444210707 | 7.05E-09 |
| NFE2L2 | AC022075.1 | 0.397287454 | 3.08E-07 |
| PTEN | AC022075.1 | -0.319837864 | 4.97E-05 |
| AMBRA1 | AC024075.3 | 0.334037856 | 2.16E-05 |
| ARNT | AC024075.3 | 0.406713993 | 1.51E-07 |
| ARSB | AC024075.3 | 0.420905572 | 4.94E-08 |
| BCL2 | AC024075.3 | 0.454818175 | 2.76E-09 |
| BIRC6 | AC024075.3 | 0.429409434 | 2.47E-08 |
| CAMKK2 | AC024075.3 | 0.321396956 | 4.55E-05 |
| CCR2 | AC024075.3 | 0.395507537 | 3.52E-07 |
| CDKN1B | AC024075.3 | 0.314172224 | 6.86E-05 |
| CFLAR | AC024075.3 | 0.321648849 | 4.48E-05 |
| CXCR4 | AC024075.3 | 0.355354931 | 5.70E-06 |
| DRAM1 | AC024075.3 | 0.35798655 | 4.80E-06 |
| EEF2K | AC024075.3 | 0.496574837 | 4.99E-11 |
| EIF2AK3 | AC024075.3 | 0.450285108 | 4.14E-09 |
| EIF4EBP1 | AC024075.3 | -0.314505327 | 6.73E-05 |
| HDAC6 | AC024075.3 | 0.420289529 | 5.20E-08 |
| IKBKB | AC024075.3 | 0.310772483 | 8.30E-05 |
| IKBKE | AC024075.3 | 0.403203955 | 1.97E-07 |
| ITPR1 | AC024075.3 | 0.322904022 | 4.17E-05 |
| KLHL24 | AC024075.3 | 0.359309437 | 4.40E-06 |
| MAP2K7 | AC024075.3 | 0.405259347 | 1.69E-07 |
| MAPK1 | AC024075.3 | 0.326951181 | 3.29E-05 |
| MAPK8 | AC024075.3 | 0.394927121 | 3.67E-07 |
| MTOR | AC024075.3 | 0.359908911 | 4.23E-06 |
| NBR1 | AC024075.3 | 0.400561832 | 2.41E-07 |
| NKX2-3 | AC024075.3 | 0.388909091 | 5.70E-07 |
| PARP1 | AC024075.3 | 0.385102537 | 7.50E-07 |
| PIK3R4 | AC024075.3 | 0.447211817 | 5.42E-09 |
| PRKAB1 | AC024075.3 | 0.350677234 | 7.70E-06 |
| RAF1 | AC024075.3 | 0.388324063 | 5.95E-07 |
| SIRT1 | AC024075.3 | 0.390418653 | 5.11E-07 |
| TP53 | AC024075.3 | 0.372281442 | 1.84E-06 |
| TP73 | AC024075.3 | 0.495142751 | 5.77E-11 |
| TSC1 | AC024075.3 | 0.449816888 | 4.31E-09 |
| TSC2 | AC024075.3 | 0.495533902 | 5.55E-11 |
| ULK2 | AC024075.3 | 0.382064324 | 9.32E-07 |
| UVRAG | AC024075.3 | 0.308638725 | 9.34E-05 |
| CAPN1 | AC245041.1 | 0.302865635 | 0.00012789 |
| CAPN10 | AC245041.1 | -0.310751906 | 8.31E-05 |
| CAPN2 | AC245041.1 | 0.412213526 | 9.86E-08 |
| GABARAP | AC245041.1 | -0.303991328 | 0.000120338 |
| GOPC | AC245041.1 | 0.455696535 | 2.55E-09 |
| ITGA3 | AC245041.1 | 0.421151279 | 4.85E-08 |
| ITGB4 | AC245041.1 | 0.350856541 | 7.61E-06 |
| NFKB1 | AC245041.1 | 0.446906234 | 5.57E-09 |
| WDFY3 | AC245041.1 | 0.322257225 | 4.33E-05 |
| ARNT | AP003352.1 | -0.399811995 | 2.55E-07 |
| ATF4 | AP003352.1 | 0.504134688 | 2.27E-11 |
| ATF6 | AP003352.1 | -0.301038974 | 0.000141091 |
| ATG16L2 | AP003352.1 | 0.484446255 | 1.69E-10 |
| ATG2B | AP003352.1 | -0.317029098 | 5.84E-05 |
| ATG4B | AP003352.1 | 0.482712603 | 2.01E-10 |
| BAG3 | AP003352.1 | -0.352522667 | 6.84E-06 |
| BAX | AP003352.1 | 0.477163751 | 3.45E-10 |
| BIRC5 | AP003352.1 | 0.354995941 | 5.83E-06 |
| BIRC6 | AP003352.1 | -0.425468864 | 3.42E-08 |
| BNIP1 | AP003352.1 | 0.452574424 | 3.38E-09 |
| CAPN1 | AP003352.1 | -0.320987278 | 4.66E-05 |
| CAPN10 | AP003352.1 | 0.513106173 | 8.71E-12 |
| CAPN2 | AP003352.1 | -0.345315976 | 1.08E-05 |
| DAPK2 | AP003352.1 | 0.303657428 | 0.000122533 |
| DDIT3 | AP003352.1 | 0.326678913 | 3.35E-05 |
| EIF4G1 | AP003352.1 | -0.443927735 | 7.22E-09 |
| FKBP1A | AP003352.1 | 0.318778308 | 5.29E-05 |
| GABARAPL2 | AP003352.1 | 0.317995587 | 5.53E-05 |
| KIF5B | AP003352.1 | -0.424153418 | 3.80E-08 |
| LAMP2 | AP003352.1 | -0.345816805 | 1.05E-05 |
| MAPK1 | AP003352.1 | -0.46121854 | 1.55E-09 |
| NCKAP1 | AP003352.1 | -0.381950944 | 9.39E-07 |
| PELP1 | AP003352.1 | 0.348643603 | 8.76E-06 |
| PEX14 | AP003352.1 | 0.343054135 | 1.24E-05 |
| PIK3R4 | AP003352.1 | -0.310812971 | 8.28E-05 |
| RAB24 | AP003352.1 | 0.423007475 | 4.17E-08 |
| RB1 | AP003352.1 | -0.324919904 | 3.71E-05 |
| TP63 | AP003352.1 | -0.325097442 | 3.67E-05 |
| USP10 | AP003352.1 | -0.382982233 | 8.73E-07 |
| WDFY3 | AP003352.1 | -0.396031081 | 3.38E-07 |
| WDR45 | AP003352.1 | 0.421130167 | 4.86E-08 |
| ATG4D | MCF2L-AS1 | 0.329307898 | 2.86E-05 |
| CDKN1A | MCF2L-AS1 | -0.378549443 | 1.19E-06 |
| CDKN1B | MCF2L-AS1 | 0.320115411 | 4.90E-05 |
| CXCR4 | MCF2L-AS1 | 0.404420081 | 1.80E-07 |
| EEF2 | MCF2L-AS1 | 0.303127193 | 0.000126097 |
| GNAI3 | MCF2L-AS1 | -0.301691106 | 0.000136239 |
| ITGA3 | MCF2L-AS1 | -0.361353313 | 3.85E-06 |
| KLHL24 | MCF2L-AS1 | 0.503100393 | 2.53E-11 |
| MAP2K7 | MCF2L-AS1 | 0.3365934 | 1.85E-05 |
| MAPK9 | MCF2L-AS1 | 0.338283452 | 1.67E-05 |
| NBR1 | MCF2L-AS1 | 0.327999463 | 3.10E-05 |
| PARP1 | MCF2L-AS1 | 0.374648319 | 1.57E-06 |
| PINK1 | MCF2L-AS1 | -0.327711412 | 3.15E-05 |
| PTK6 | MCF2L-AS1 | -0.378122998 | 1.23E-06 |
| SIRT1 | MCF2L-AS1 | 0.370556387 | 2.07E-06 |
| TP53 | MCF2L-AS1 | 0.318325711 | 5.42E-05 |
| UVRAG | MCF2L-AS1 | 0.403918664 | 1.87E-07 |
| WDR45 | MCF2L-AS1 | 0.318548699 | 5.36E-05 |
| CCL2 | AC022509.2 | 0.320804967 | 4.71E-05 |
| CXCR4 | AC022509.2 | 0.592447521 | 4.66E-16 |
| EEF2 | AC022509.2 | 0.303341473 | 0.000124645 |
| SPHK1 | AC022509.2 | 0.357983107 | 4.80E-06 |
| TP53 | AC022509.2 | 0.342285075 | 1.30E-05 |
| TP73 | AC022509.2 | 0.303632828 | 0.000122697 |
| TSC2 | AC022509.2 | 0.425054788 | 3.53E-08 |
| UVRAG | AC022509.2 | 0.370483121 | 2.08E-06 |
| WIPI1 | AC131157.1 | 0.308581777 | 9.36E-05 |
| BID | AL021392.1 | 0.319964679 | 4.94E-05 |
| ATF4 | AL031186.1 | 0.302485999 | 0.000130535 |
| ATG4B | AL031186.1 | 0.384753304 | 7.69E-07 |
| CAPN10 | AL031186.1 | 0.381680882 | 9.57E-07 |
| CXCR4 | AL031186.1 | 0.472064719 | 5.62E-10 |
| HDAC6 | AL031186.1 | 0.379596021 | 1.11E-06 |
| IKBKB | AL031186.1 | 0.39525528 | 3.58E-07 |
| TSC2 | AL031186.1 | 0.356613562 | 5.25E-06 |
| ULK3 | AL031186.1 | 0.31186299 | 7.81E-05 |
| WDR45 | AL031186.1 | 0.405729217 | 1.63E-07 |
| ARSB | AC026471.4 | 0.325505105 | 3.58E-05 |
| BAK1 | AC026471.4 | -0.32568209 | 3.55E-05 |
| CDKN1A | AC026471.4 | -0.320990808 | 4.66E-05 |
| CDKN1B | AC026471.4 | 0.361279356 | 3.87E-06 |
| CXCR4 | AC026471.4 | 0.536823228 | 6.01E-13 |
| EEF2 | AC026471.4 | 0.328550944 | 3.00E-05 |
| EIF2AK3 | AC026471.4 | 0.341213924 | 1.39E-05 |
| FOXO3 | AC026471.4 | 0.324943298 | 3.70E-05 |
| IKBKB | AC026471.4 | 0.355078722 | 5.80E-06 |
| ITGA3 | AC026471.4 | -0.34101523 | 1.41E-05 |
| KLHL24 | AC026471.4 | 0.396214389 | 3.34E-07 |
| MAP2K7 | AC026471.4 | 0.310910288 | 8.23E-05 |
| NBR1 | AC026471.4 | 0.309761538 | 8.77E-05 |
| PINK1 | AC026471.4 | -0.358131928 | 4.76E-06 |
| PRKAB1 | AC026471.4 | 0.31169461 | 7.88E-05 |
| TP53 | AC026471.4 | 0.375148353 | 1.51E-06 |
| TSC1 | AC026471.4 | 0.363116152 | 3.42E-06 |
| TSC2 | AC026471.4 | 0.377919098 | 1.25E-06 |
| ULK1 | AC026471.4 | 0.301713368 | 0.000136076 |
| UVRAG | AC026471.4 | 0.492132623 | 7.85E-11 |
| BID | LINC01752 | 0.353082623 | 6.60E-06 |
| CANX | LINC01752 | -0.330386261 | 2.69E-05 |
| MAPK9 | LINC01752 | -0.306557675 | 0.00010465 |
| ARNT | AC073896.4 | -0.335212428 | 2.01E-05 |
| ATF4 | AC073896.4 | 0.407810826 | 1.39E-07 |
| ATF6 | AC073896.4 | -0.328668269 | 2.98E-05 |
| ATG10 | AC073896.4 | 0.325406759 | 3.60E-05 |
| ATG16L2 | AC073896.4 | 0.390998059 | 4.90E-07 |
| ATG2B | AC073896.4 | -0.404109132 | 1.84E-07 |
| ATG4B | AC073896.4 | 0.626344982 | 2.88E-18 |
| ATG4D | AC073896.4 | 0.371703798 | 1.92E-06 |
| ATG7 | AC073896.4 | 0.332386702 | 2.38E-05 |
| BAG1 | AC073896.4 | 0.335142296 | 2.02E-05 |
| BAG3 | AC073896.4 | -0.327838374 | 3.12E-05 |
| BAX | AC073896.4 | 0.635050198 | 7.05E-19 |
| BECN1 | AC073896.4 | -0.344872753 | 1.11E-05 |
| BID | AC073896.4 | 0.327957031 | 3.10E-05 |
| BIRC5 | AC073896.4 | 0.442949909 | 7.86E-09 |
| BIRC6 | AC073896.4 | -0.385876912 | 7.10E-07 |
| BNIP1 | AC073896.4 | 0.617457825 | 1.16E-17 |
| CANX | AC073896.4 | -0.330352386 | 2.69E-05 |
| CAPN10 | AC073896.4 | 0.566370613 | 1.58E-14 |
| CAPN2 | AC073896.4 | -0.337415678 | 1.76E-05 |
| CAPNS1 | AC073896.4 | 0.452296823 | 3.46E-09 |
| CDKN2A | AC073896.4 | 0.505780547 | 1.91E-11 |
| CLN3 | AC073896.4 | 0.395513279 | 3.52E-07 |
| CXCR4 | AC073896.4 | 0.377911491 | 1.25E-06 |
| EIF4G1 | AC073896.4 | -0.435115159 | 1.53E-08 |
| FKBP1A | AC073896.4 | 0.577544953 | 3.63E-15 |
| GABARAP | AC073896.4 | 0.44080106 | 9.46E-09 |
| GOPC | AC073896.4 | -0.320555089 | 4.77E-05 |
| HIF1A | AC073896.4 | -0.367139555 | 2.61E-06 |
| ITGA6 | AC073896.4 | -0.355380894 | 5.69E-06 |
| ITGB1 | AC073896.4 | -0.344783942 | 1.12E-05 |
| KIF5B | AC073896.4 | -0.350303068 | 7.88E-06 |
| LAMP2 | AC073896.4 | -0.324019001 | 3.91E-05 |
| MAP1LC3A | AC073896.4 | 0.472706723 | 5.28E-10 |
| MAPK1 | AC073896.4 | -0.444766363 | 6.71E-09 |
| MLST8 | AC073896.4 | 0.368401249 | 2.40E-06 |
| MTMR14 | AC073896.4 | 0.478260519 | 3.10E-10 |
| NAF1 | AC073896.4 | -0.317231723 | 5.77E-05 |
| NCKAP1 | AC073896.4 | -0.475503215 | 4.04E-10 |
| NPC1 | AC073896.4 | -0.343407616 | 1.22E-05 |
| PELP1 | AC073896.4 | 0.403380312 | 1.95E-07 |
| PEX14 | AC073896.4 | 0.416865965 | 6.83E-08 |
| PINK1 | AC073896.4 | -0.348545158 | 8.81E-06 |
| RAB1A | AC073896.4 | -0.319689232 | 5.02E-05 |
| RAB24 | AC073896.4 | 0.41235606 | 9.75E-08 |
| RAC1 | AC073896.4 | 0.312543358 | 7.52E-05 |
| SPNS1 | AC073896.4 | -0.310937228 | 8.22E-05 |
| STK11 | AC073896.4 | 0.39076397 | 4.98E-07 |
| TP63 | AC073896.4 | -0.328457011 | 3.01E-05 |
| USP10 | AC073896.4 | -0.428998999 | 2.56E-08 |
| WDFY3 | AC073896.4 | -0.435313404 | 1.51E-08 |
| WDR45 | AC073896.4 | 0.571633889 | 7.96E-15 |
| ZFYVE1 | AC073896.4 | -0.319242139 | 5.15E-05 |
| ATG16L2 | AL049870.3 | 0.452194399 | 3.49E-09 |
| ATG9B | AL049870.3 | 0.416322845 | 7.13E-08 |
| BID | AL049870.3 | 0.324153656 | 3.88E-05 |
| FKBP1B | AL049870.3 | 0.406518066 | 1.53E-07 |
| FOXO1 | AL049870.3 | -0.402238651 | 2.12E-07 |
| MLST8 | AL049870.3 | 0.3157248 | 6.29E-05 |
| PRKCQ | AL049870.3 | 0.382271084 | 9.18E-07 |
| ATG12 | AL645608.7 | 0.304592175 | 0.00011648 |
| CASP8 | AL645608.7 | 0.308467607 | 9.42E-05 |
| CFLAR | AL645608.7 | 0.315490423 | 6.37E-05 |
| PEX14 | AL645608.7 | 0.359941439 | 4.22E-06 |
| VAMP3 | AL645608.7 | 0.441952235 | 8.57E-09 |
| ATG10 | C5orf56 | 0.4591371 | 1.87E-09 |
| ATG12 | C5orf56 | 0.370513405 | 2.08E-06 |
| ATG4B | C5orf56 | 0.40797449 | 1.37E-07 |
| ATG4C | C5orf56 | 0.487516748 | 1.25E-10 |
| ATG4D | C5orf56 | 0.386825737 | 6.63E-07 |
| ATG7 | C5orf56 | 0.529838894 | 1.35E-12 |
| BAX | C5orf56 | 0.428572892 | 2.65E-08 |
| BCL2 | C5orf56 | 0.483307896 | 1.89E-10 |
| BNIP1 | C5orf56 | 0.465107732 | 1.08E-09 |
| BNIP3 | C5orf56 | -0.318587577 | 5.34E-05 |
| CALCOCO2 | C5orf56 | 0.394335639 | 3.84E-07 |
| CAPN10 | C5orf56 | 0.41339891 | 8.98E-08 |
| CAPNS1 | C5orf56 | 0.475227478 | 4.15E-10 |
| CCR2 | C5orf56 | 0.401221617 | 2.29E-07 |
| CDKN1B | C5orf56 | 0.408926695 | 1.27E-07 |
| CDKN2A | C5orf56 | 0.581755833 | 2.05E-15 |
| CFLAR | C5orf56 | 0.45706179 | 2.26E-09 |
| CTSD | C5orf56 | 0.359551293 | 4.33E-06 |
| CX3CL1 | C5orf56 | 0.363015739 | 3.45E-06 |
| CXCR4 | C5orf56 | 0.592310554 | 4.75E-16 |
| DRAM1 | C5orf56 | 0.477716734 | 3.27E-10 |
| EEF2K | C5orf56 | 0.344897504 | 1.11E-05 |
| FAS | C5orf56 | 0.511736425 | 1.01E-11 |
| GABARAP | C5orf56 | 0.396735896 | 3.21E-07 |
| HDAC6 | C5orf56 | 0.457661434 | 2.14E-09 |
| HIF1A | C5orf56 | -0.379177973 | 1.14E-06 |
| HSPA5 | C5orf56 | -0.336151308 | 1.90E-05 |
| IFNG | C5orf56 | 0.560438024 | 3.38E-14 |
| IKBKB | C5orf56 | 0.357474301 | 4.96E-06 |
| IKBKE | C5orf56 | 0.534498436 | 7.88E-13 |
| ITPR1 | C5orf56 | 0.317736584 | 5.61E-05 |
| MAP1LC3A | C5orf56 | 0.35135792 | 7.37E-06 |
| MAP1LC3B | C5orf56 | -0.337760102 | 1.72E-05 |
| MAP2K7 | C5orf56 | 0.443297773 | 7.63E-09 |
| MAPK8 | C5orf56 | 0.380588203 | 1.03E-06 |
| MAPK9 | C5orf56 | 0.369394443 | 2.24E-06 |
| MTMR14 | C5orf56 | 0.617882552 | 1.09E-17 |
| NCKAP1 | C5orf56 | -0.372407673 | 1.83E-06 |
| NKX2-3 | C5orf56 | 0.546878862 | 1.81E-13 |
| PEX14 | C5orf56 | 0.444270569 | 7.01E-09 |
| PRKAB1 | C5orf56 | 0.43714676 | 1.29E-08 |
| RAB1A | C5orf56 | -0.34268299 | 1.27E-05 |
| RAB24 | C5orf56 | 0.474576467 | 4.42E-10 |
| RAF1 | C5orf56 | 0.431993782 | 1.99E-08 |
| RGS19 | C5orf56 | 0.403832085 | 1.88E-07 |
| STK11 | C5orf56 | 0.541682709 | 3.38E-13 |
| TP53 | C5orf56 | 0.52847611 | 1.58E-12 |
| TP73 | C5orf56 | 0.574829684 | 5.22E-15 |
| TSC2 | C5orf56 | 0.631686189 | 1.22E-18 |
| TUSC1 | C5orf56 | 0.300514162 | 0.000145113 |
| WDR45 | C5orf56 | 0.470723373 | 6.38E-10 |
| ATG10 | AC137932.1 | 0.430282251 | 2.30E-08 |
| ATG2A | AC137932.1 | 0.311284353 | 8.06E-05 |
| ATG7 | AC137932.1 | 0.319564468 | 5.05E-05 |
| CDKN1B | AC137932.1 | 0.323151604 | 4.11E-05 |
| DIRAS3 | AC137932.1 | 0.30745914 | 9.96E-05 |
| EDEM1 | AC137932.1 | 0.310479126 | 8.43E-05 |
| EEF2K | AC137932.1 | 0.302329928 | 0.000131637 |
| HDAC6 | AC137932.1 | 0.352637286 | 6.79E-06 |
| MAPK8 | AC137932.1 | 0.436957289 | 1.31E-08 |
| MTMR14 | AC137932.1 | 0.383977822 | 8.13E-07 |
| MTOR | AC137932.1 | 0.318612208 | 5.34E-05 |
| NKX2-3 | AC137932.1 | 0.381438519 | 9.74E-07 |
| RAF1 | AC137932.1 | 0.461809309 | 1.46E-09 |
| TP53 | AC137932.1 | 0.307607064 | 9.88E-05 |
| TP73 | AC137932.1 | 0.378893353 | 1.16E-06 |
| TSC1 | AC137932.1 | 0.341635554 | 1.36E-05 |
| TSC2 | AC137932.1 | 0.301075889 | 0.000140812 |
| BAX | LINC02446 | 0.30909067 | 9.11E-05 |
| CALCOCO2 | LINC02446 | 0.344965627 | 1.10E-05 |
| CCR2 | LINC02446 | 0.614638241 | 1.79E-17 |
| CDKN1B | LINC02446 | 0.325821572 | 3.52E-05 |
| CDKN2A | LINC02446 | 0.331472775 | 2.52E-05 |
| CTSD | LINC02446 | 0.311728048 | 7.87E-05 |
| FAS | LINC02446 | 0.306718701 | 0.000103733 |
| IFNG | LINC02446 | 0.82621783 | 5.75E-40 |
| MAPK9 | LINC02446 | 0.312476637 | 7.55E-05 |
| MTMR14 | LINC02446 | 0.388876889 | 5.72E-07 |
| NKX2-3 | LINC02446 | 0.347980353 | 9.13E-06 |
| RGS19 | LINC02446 | 0.321227278 | 4.59E-05 |
| STK11 | LINC02446 | 0.342596212 | 1.28E-05 |
| TP53 | LINC02446 | 0.386994473 | 6.55E-07 |
| ARNT | AC011445.2 | -0.3228562 | 4.18E-05 |
| BAG1 | AC011445.2 | 0.383442697 | 8.45E-07 |
| BAX | AC011445.2 | 0.567766991 | 1.32E-14 |
| BECN1 | AC011445.2 | -0.367506617 | 2.55E-06 |
| BIRC5 | AC011445.2 | 0.501208462 | 3.09E-11 |
| BIRC6 | AC011445.2 | -0.353668047 | 6.35E-06 |
| BNIP1 | AC011445.2 | 0.341666596 | 1.36E-05 |
| CAPN2 | AC011445.2 | -0.321268921 | 4.58E-05 |
| EIF4EBP1 | AC011445.2 | 0.461324417 | 1.53E-09 |
| FKBP1A | AC011445.2 | 0.433161316 | 1.81E-08 |
| HGS | AC011445.2 | -0.334336307 | 2.12E-05 |
| MAPK1 | AC011445.2 | -0.389668736 | 5.40E-07 |
| NBR1 | AC011445.2 | -0.315642534 | 6.32E-05 |
| NCKAP1 | AC011445.2 | -0.344523652 | 1.13E-05 |
| RB1 | AC011445.2 | -0.308115704 | 9.61E-05 |
| SPNS1 | AC011445.2 | -0.306630902 | 0.000104232 |
| WDR45 | AC011445.2 | 0.406555134 | 1.53E-07 |
| AMBRA1 | FOXN3-AS1 | -0.380919689 | 1.01E-06 |
| ARNT | FOXN3-AS1 | -0.334682072 | 2.08E-05 |
| ATG2A | FOXN3-AS1 | -0.373559162 | 1.69E-06 |
| BIRC6 | FOXN3-AS1 | -0.330892344 | 2.61E-05 |
| CALCOCO2 | FOXN3-AS1 | -0.367553352 | 2.54E-06 |
| CAMKK2 | FOXN3-AS1 | -0.332834197 | 2.32E-05 |
| CANX | FOXN3-AS1 | -0.350045002 | 8.01E-06 |
| CD46 | FOXN3-AS1 | -0.309198239 | 9.05E-05 |
| DRAM1 | FOXN3-AS1 | -0.305847489 | 0.000108789 |
| EDEM1 | FOXN3-AS1 | -0.306462164 | 0.000105198 |
| EEF2K | FOXN3-AS1 | -0.3233448 | 4.06E-05 |
| EIF2AK2 | FOXN3-AS1 | -0.344557292 | 1.13E-05 |
| EIF4EBP1 | FOXN3-AS1 | 0.454926148 | 2.74E-09 |
| FAS | FOXN3-AS1 | -0.360612728 | 4.04E-06 |
| HGS | FOXN3-AS1 | -0.39818955 | 2.88E-07 |
| MAPK8 | FOXN3-AS1 | -0.40924769 | 1.24E-07 |
| MAPK9 | FOXN3-AS1 | -0.385215683 | 7.44E-07 |
| MTOR | FOXN3-AS1 | -0.300033336 | 0.000148891 |
| NAF1 | FOXN3-AS1 | -0.396883373 | 3.18E-07 |
| NBR1 | FOXN3-AS1 | -0.342346549 | 1.30E-05 |
| NFKB1 | FOXN3-AS1 | -0.482632957 | 2.02E-10 |
| PARP1 | FOXN3-AS1 | -0.310288564 | 8.52E-05 |
| RAB33B | FOXN3-AS1 | -0.329741975 | 2.79E-05 |
| RB1 | FOXN3-AS1 | -0.378350863 | 1.21E-06 |
| RPS6KB1 | FOXN3-AS1 | -0.354146629 | 6.16E-06 |
| SH3GLB1 | FOXN3-AS1 | -0.398740727 | 2.76E-07 |
| SIRT1 | FOXN3-AS1 | -0.329813901 | 2.78E-05 |
| SPNS1 | FOXN3-AS1 | -0.310205247 | 8.56E-05 |
| SQSTM1 | FOXN3-AS1 | -0.322925836 | 4.16E-05 |
| TSC2 | FOXN3-AS1 | -0.325668267 | 3.55E-05 |
| ARNT | AC012467.2 | 0.315612634 | 6.33E-05 |
| ARSB | AC012467.2 | 0.326185915 | 3.44E-05 |
| ATG10 | AC012467.2 | 0.348012565 | 9.12E-06 |
| ATG12 | AC012467.2 | 0.317776703 | 5.60E-05 |
| ATG16L2 | AC012467.2 | 0.31125134 | 8.08E-05 |
| ATG3 | AC012467.2 | 0.305270763 | 0.000112261 |
| ATG4C | AC012467.2 | 0.513391808 | 8.44E-12 |
| BCL2 | AC012467.2 | 0.345368853 | 1.08E-05 |
| CAPN10 | AC012467.2 | 0.45363162 | 3.07E-09 |
| CASP3 | AC012467.2 | 0.356061882 | 5.44E-06 |
| CD46 | AC012467.2 | 0.471936311 | 5.68E-10 |
| CDKN1B | AC012467.2 | 0.40442462 | 1.80E-07 |
| CXCR4 | AC012467.2 | 0.510888119 | 1.11E-11 |
| DAPK2 | AC012467.2 | 0.311878553 | 7.80E-05 |
| DRAM1 | AC012467.2 | 0.378337102 | 1.21E-06 |
| EEF2K | AC012467.2 | 0.320680541 | 4.74E-05 |
| EIF2AK3 | AC012467.2 | 0.338617576 | 1.64E-05 |
| FAS | AC012467.2 | 0.428895267 | 2.58E-08 |
| HDAC6 | AC012467.2 | 0.442078889 | 8.47E-09 |
| IKBKB | AC012467.2 | 0.391645512 | 4.67E-07 |
| IKBKE | AC012467.2 | 0.374468951 | 1.59E-06 |
| ITGA3 | AC012467.2 | -0.364532824 | 3.11E-06 |
| ITGA6 | AC012467.2 | -0.307595439 | 9.89E-05 |
| ITPR1 | AC012467.2 | 0.306452079 | 0.000105256 |
| KLHL24 | AC012467.2 | 0.31002551 | 8.65E-05 |
| MAP2K7 | AC012467.2 | 0.325548856 | 3.57E-05 |
| MAPK8 | AC012467.2 | 0.47301165 | 5.13E-10 |
| MTMR14 | AC012467.2 | 0.364273432 | 3.17E-06 |
| PARP1 | AC012467.2 | 0.476501029 | 3.67E-10 |
| PINK1 | AC012467.2 | -0.358051073 | 4.78E-06 |
| PRKCD | AC012467.2 | 0.380367341 | 1.05E-06 |
| PTK6 | AC012467.2 | -0.310301178 | 8.52E-05 |
| RAB33B | AC012467.2 | 0.388433432 | 5.90E-07 |
| RAF1 | AC012467.2 | 0.483325643 | 1.89E-10 |
| RPS6KB1 | AC012467.2 | 0.347152755 | 9.62E-06 |
| SIRT1 | AC012467.2 | 0.458173575 | 2.04E-09 |
| STK11 | AC012467.2 | 0.329358934 | 2.86E-05 |
| TP53 | AC012467.2 | 0.44082151 | 9.44E-09 |
| TP73 | AC012467.2 | 0.365514376 | 2.92E-06 |
| TSC1 | AC012467.2 | 0.336660094 | 1.84E-05 |
| TSC2 | AC012467.2 | 0.432801958 | 1.86E-08 |
| ULK3 | AC012467.2 | 0.44110149 | 9.22E-09 |
| ATF4 | AC067838.1 | 0.422702065 | 4.28E-08 |
| ATG10 | AC067838.1 | 0.403131202 | 1.99E-07 |
| ATG16L2 | AC067838.1 | 0.405419318 | 1.67E-07 |
| ATG4B | AC067838.1 | 0.481567168 | 2.25E-10 |
| ATG4C | AC067838.1 | 0.323751433 | 3.97E-05 |
| ATG4D | AC067838.1 | 0.353540702 | 6.41E-06 |
| ATG7 | AC067838.1 | 0.319568389 | 5.05E-05 |
| ATIC | AC067838.1 | 0.320203684 | 4.87E-05 |
| BAG3 | AC067838.1 | -0.446414783 | 5.81E-09 |
| BAX | AC067838.1 | 0.577285951 | 3.76E-15 |
| BIRC5 | AC067838.1 | 0.462791456 | 1.34E-09 |
| BIRC6 | AC067838.1 | -0.310406968 | 8.47E-05 |
| BNIP1 | AC067838.1 | 0.511368452 | 1.05E-11 |
| CAPN10 | AC067838.1 | 0.516282923 | 6.16E-12 |
| CAPN2 | AC067838.1 | -0.378026034 | 1.24E-06 |
| CDKN2A | AC067838.1 | 0.376221408 | 1.40E-06 |
| CHMP4B | AC067838.1 | 0.381444676 | 9.73E-07 |
| CLN3 | AC067838.1 | 0.334672636 | 2.08E-05 |
| CXCR4 | AC067838.1 | 0.443585865 | 7.44E-09 |
| EIF4G1 | AC067838.1 | -0.421921168 | 4.56E-08 |
| FKBP1A | AC067838.1 | 0.45435426 | 2.88E-09 |
| GABARAP | AC067838.1 | 0.447762314 | 5.17E-09 |
| HDAC6 | AC067838.1 | 0.308227046 | 9.55E-05 |
| HIF1A | AC067838.1 | -0.360941497 | 3.95E-06 |
| ITGA6 | AC067838.1 | -0.346831607 | 9.82E-06 |
| ITGB1 | AC067838.1 | -0.323360064 | 4.06E-05 |
| KIF5B | AC067838.1 | -0.3099237 | 8.70E-05 |
| MAP1LC3A | AC067838.1 | 0.515722897 | 6.55E-12 |
| MAP2K7 | AC067838.1 | 0.320605268 | 4.76E-05 |
| MAPK1 | AC067838.1 | -0.361333321 | 3.85E-06 |
| MLST8 | AC067838.1 | 0.357197308 | 5.05E-06 |
| MTMR14 | AC067838.1 | 0.519107529 | 4.51E-12 |
| NCKAP1 | AC067838.1 | -0.386198119 | 6.94E-07 |
| NKX2-3 | AC067838.1 | 0.335750863 | 1.95E-05 |
| NPC1 | AC067838.1 | -0.308128612 | 9.60E-05 |
| PELP1 | AC067838.1 | 0.373984657 | 1.64E-06 |
| PEX14 | AC067838.1 | 0.421373922 | 4.76E-08 |
| RAB24 | AC067838.1 | 0.430286945 | 2.30E-08 |
| STK11 | AC067838.1 | 0.46941027 | 7.22E-10 |
| TP63 | AC067838.1 | -0.33211183 | 2.42E-05 |
| TSC2 | AC067838.1 | 0.346106007 | 1.03E-05 |
| USP10 | AC067838.1 | -0.391356105 | 4.77E-07 |
| WDFY3 | AC067838.1 | -0.334944098 | 2.04E-05 |
| WDR45 | AC067838.1 | 0.539276961 | 4.50E-13 |
| ZFYVE1 | AC067838.1 | -0.351927091 | 7.11E-06 |
| ATG16L2 | AL691482.3 | 0.315497334 | 6.37E-05 |
| HDAC1 | AL691482.3 | 0.336629686 | 1.85E-05 |
| ULK3 | AL691482.3 | 0.344162 | 1.16E-05 |
| NAMPT | AL136018.1 | 0.447505026 | 5.29E-09 |
| NFE2L2 | AL136018.1 | 0.367705351 | 2.52E-06 |
| TM9SF1 | AL136018.1 | 0.327388806 | 3.21E-05 |
| TP63 | AL136018.1 | 0.395615912 | 3.49E-07 |
| ATG2B | KTN1-AS1 | 0.330236979 | 2.71E-05 |
| ATG3 | KTN1-AS1 | -0.344330579 | 1.15E-05 |
| ATG7 | KTN1-AS1 | -0.304547015 | 0.000116766 |
| EIF2S1 | KTN1-AS1 | 0.36148806 | 3.81E-06 |
| IKBKE | KTN1-AS1 | -0.326495344 | 3.38E-05 |
| MTMR14 | KTN1-AS1 | -0.345520314 | 1.07E-05 |
| RAB24 | KTN1-AS1 | -0.300222468 | 0.000147394 |
| RAF1 | KTN1-AS1 | -0.313676243 | 7.06E-05 |
| STK11 | KTN1-AS1 | -0.304099197 | 0.000119637 |
| ZFYVE1 | KTN1-AS1 | 0.308921753 | 9.19E-05 |
| ATG16L2 | AC136475.3 | 0.33667037 | 1.84E-05 |
| BCL2 | AC136475.3 | 0.35077154 | 7.65E-06 |
| CDKN1B | AC136475.3 | 0.348473854 | 8.85E-06 |
| MAP2K7 | AC136475.3 | 0.322995269 | 4.15E-05 |
| MAPK8 | AC136475.3 | 0.30656776 | 0.000104592 |
| ARNT | AC012510.1 | -0.409437189 | 1.22E-07 |
| ATF4 | AC012510.1 | 0.488763855 | 1.10E-10 |
| ATF6 | AC012510.1 | -0.364806927 | 3.06E-06 |
| ATG2B | AC012510.1 | -0.387999234 | 6.09E-07 |
| ATG4B | AC012510.1 | 0.671989467 | 1.05E-21 |
| ATIC | AC012510.1 | 0.332959746 | 2.30E-05 |
| BAG1 | AC012510.1 | 0.319196878 | 5.16E-05 |
| BAG3 | AC012510.1 | -0.31305404 | 7.31E-05 |
| BAX | AC012510.1 | 0.447504502 | 5.29E-09 |
| BIRC5 | AC012510.1 | 0.353896531 | 6.26E-06 |
| BIRC6 | AC012510.1 | -0.422976535 | 4.18E-08 |
| BNIP1 | AC012510.1 | 0.576322438 | 4.27E-15 |
| CANX | AC012510.1 | -0.398904301 | 2.73E-07 |
| CAPN10 | AC012510.1 | 0.466424796 | 9.55E-10 |
| CAPN2 | AC012510.1 | -0.320879733 | 4.69E-05 |
| CLN3 | AC012510.1 | 0.320004775 | 4.93E-05 |
| DAPK2 | AC012510.1 | 0.344254441 | 1.15E-05 |
| EDEM1 | AC012510.1 | -0.352342483 | 6.92E-06 |
| EIF4G1 | AC012510.1 | -0.39561007 | 3.49E-07 |
| ERN1 | AC012510.1 | -0.345358108 | 1.08E-05 |
| HGS | AC012510.1 | -0.311103868 | 8.15E-05 |
| HIF1A | AC012510.1 | -0.324735514 | 3.75E-05 |
| ITGB1 | AC012510.1 | -0.316145704 | 6.14E-05 |
| KIF5B | AC012510.1 | -0.427898218 | 2.80E-08 |
| LAMP2 | AC012510.1 | -0.315287803 | 6.44E-05 |
| MAP1LC3A | AC012510.1 | 0.33858781 | 1.64E-05 |
| MAPK1 | AC012510.1 | -0.418376221 | 6.06E-08 |
| MBTPS2 | AC012510.1 | -0.333177461 | 2.27E-05 |
| MLST8 | AC012510.1 | 0.330357947 | 2.69E-05 |
| MTOR | AC012510.1 | -0.300853316 | 0.000142502 |
| NAF1 | AC012510.1 | -0.34327124 | 1.23E-05 |
| NCKAP1 | AC012510.1 | -0.445629735 | 6.23E-09 |
| NPC1 | AC012510.1 | -0.389829987 | 5.33E-07 |
| PELP1 | AC012510.1 | 0.427556348 | 2.88E-08 |
| PEX14 | AC012510.1 | 0.337616859 | 1.74E-05 |
| PTEN | AC012510.1 | -0.304659433 | 0.000116055 |
| RAB5A | AC012510.1 | -0.306310607 | 0.000106073 |
| SIRT1 | AC012510.1 | -0.322302737 | 4.32E-05 |
| STK11 | AC012510.1 | 0.40395389 | 1.86E-07 |
| TM9SF1 | AC012510.1 | -0.300892981 | 0.0001422 |
| USP10 | AC012510.1 | -0.402344518 | 2.11E-07 |
| WDFY3 | AC012510.1 | -0.410221524 | 1.15E-07 |
| WDR45 | AC012510.1 | 0.421045736 | 4.89E-08 |
| BAX | LINC02167 | 0.362010468 | 3.68E-06 |
| BIRC5 | LINC02167 | 0.356373268 | 5.33E-06 |
| SERPINA1 | LINC02167 | 0.345794733 | 1.05E-05 |
| ARNT | LINC00649 | 0.31121944 | 8.09E-05 |
| ATG10 | LINC00649 | 0.37317887 | 1.73E-06 |
| ATG2A | LINC00649 | 0.37164543 | 1.92E-06 |
| ATG4A | LINC00649 | 0.369090888 | 2.29E-06 |
| ATG7 | LINC00649 | 0.350171782 | 7.95E-06 |
| CALCOCO2 | LINC00649 | 0.372479671 | 1.82E-06 |
| CFLAR | LINC00649 | 0.367747241 | 2.51E-06 |
| EEF2K | LINC00649 | 0.36211353 | 3.66E-06 |
| FAS | LINC00649 | 0.3419479 | 1.33E-05 |
| HDAC6 | LINC00649 | 0.383791225 | 8.24E-07 |
| MAPK8 | LINC00649 | 0.369627789 | 2.21E-06 |
| MTMR14 | LINC00649 | 0.318077597 | 5.50E-05 |
| MTOR | LINC00649 | 0.380343434 | 1.05E-06 |
| NFKB1 | LINC00649 | 0.322938559 | 4.16E-05 |
| NKX2-3 | LINC00649 | 0.3020022 | 0.000133979 |
| PIK3R4 | LINC00649 | 0.378271487 | 1.22E-06 |
| RAB33B | LINC00649 | 0.36032905 | 4.12E-06 |
| RAB5A | LINC00649 | 0.317943091 | 5.54E-05 |
| RAF1 | LINC00649 | 0.413332053 | 9.03E-08 |
| TNFSF10 | LINC00649 | 0.36132798 | 3.85E-06 |
| TP53 | LINC00649 | 0.308698389 | 9.30E-05 |
| TP73 | LINC00649 | 0.515682523 | 6.58E-12 |
| TSC1 | LINC00649 | 0.316540595 | 6.00E-05 |
| TSC2 | LINC00649 | 0.354756191 | 5.92E-06 |
| VAMP3 | LINC00649 | 0.303213179 | 0.000125512 |
| HDAC6 | AC106786.1 | 0.312079706 | 7.71E-05 |
| KLHL24 | AC106786.1 | 0.317566741 | 5.66E-05 |
| BAK1 | HCP5 | 0.383670913 | 8.31E-07 |
| CALCOCO2 | HCP5 | 0.418488476 | 6.00E-08 |
| CASP1 | HCP5 | 0.347202337 | 9.59E-06 |
| EIF2AK2 | HCP5 | 0.350583838 | 7.74E-06 |
| TNFSF10 | HCP5 | 0.387022554 | 6.54E-07 |
| DAPK1 | AL135905.1 | 0.330514518 | 2.67E-05 |
| BAX | DLG5-AS1 | 0.53052419 | 1.25E-12 |
| BIRC5 | DLG5-AS1 | 0.42374837 | 3.93E-08 |
| BNIP1 | DLG5-AS1 | 0.385574242 | 7.25E-07 |
| CAPN10 | DLG5-AS1 | 0.338949558 | 1.60E-05 |
| CLN3 | DLG5-AS1 | 0.317543058 | 5.67E-05 |
| EIF4EBP1 | DLG5-AS1 | 0.309392852 | 8.96E-05 |
| FKBP1A | DLG5-AS1 | 0.376551637 | 1.37E-06 |
| MLST8 | DLG5-AS1 | 0.306180925 | 0.000106827 |
| PELP1 | DLG5-AS1 | 0.319972773 | 4.94E-05 |
| WDR45 | DLG5-AS1 | 0.413348855 | 9.02E-08 |
| ATG10 | LINC01679 | 0.406857495 | 1.49E-07 |
| ATG7 | LINC01679 | 0.358011729 | 4.79E-06 |
| CAPNS1 | LINC01679 | 0.375363712 | 1.49E-06 |
| CDKN1B | LINC01679 | 0.324135421 | 3.88E-05 |
| CDKN2A | LINC01679 | 0.461919544 | 1.45E-09 |
| CXCR4 | LINC01679 | 0.348120054 | 9.05E-06 |
| DRAM1 | LINC01679 | 0.330745412 | 2.63E-05 |
| HSP90AB1 | LINC01679 | -0.328709664 | 2.97E-05 |
| IKBKE | LINC01679 | 0.341090746 | 1.40E-05 |
| MAP1LC3A | LINC01679 | 0.330806011 | 2.62E-05 |
| MTMR14 | LINC01679 | 0.389147653 | 5.61E-07 |
| NCKAP1 | LINC01679 | -0.305140139 | 0.000113062 |
| NKX2-3 | LINC01679 | 0.465110887 | 1.08E-09 |
| PEX14 | LINC01679 | 0.392551742 | 4.37E-07 |
| PRKAB1 | LINC01679 | 0.311483801 | 7.97E-05 |
| RAC1 | LINC01679 | 0.347312469 | 9.53E-06 |
| ST13 | LINC01679 | -0.302195186 | 0.000132595 |
| STK11 | LINC01679 | 0.379370275 | 1.13E-06 |
| TP53 | LINC01679 | 0.35712751 | 5.08E-06 |
| TP73 | LINC01679 | 0.529112733 | 1.47E-12 |
| TSC2 | LINC01679 | 0.461590681 | 1.49E-09 |
| ATF4 | AC023509.4 | 0.343157683 | 1.24E-05 |
| ATG4B | AC023509.4 | 0.400925887 | 2.35E-07 |
| ATIC | AC023509.4 | 0.347601846 | 9.36E-06 |
| BAX | AC023509.4 | 0.327379168 | 3.21E-05 |
| BNIP1 | AC023509.4 | 0.389421144 | 5.50E-07 |
| CAPN10 | AC023509.4 | 0.384342388 | 7.92E-07 |
| CAPN2 | AC023509.4 | -0.335091825 | 2.03E-05 |
| CLN3 | AC023509.4 | 0.365140807 | 2.99E-06 |
| FOXO1 | AC023509.4 | -0.329113268 | 2.90E-05 |
| ITGA6 | AC023509.4 | -0.328300865 | 3.04E-05 |
| ITGB1 | AC023509.4 | -0.329125464 | 2.90E-05 |
| LAMP2 | AC023509.4 | -0.352745457 | 6.74E-06 |
| MAP1LC3A | AC023509.4 | 0.316221607 | 6.11E-05 |
| MAPK1 | AC023509.4 | -0.340149304 | 1.49E-05 |
| MLST8 | AC023509.4 | 0.402342534 | 2.11E-07 |
| NFKB1 | AC023509.4 | -0.324728227 | 3.75E-05 |
| RB1 | AC023509.4 | -0.368392029 | 2.40E-06 |
| RELA | AC023509.4 | -0.309255742 | 9.02E-05 |
| WDR45 | AC023509.4 | 0.361110498 | 3.91E-06 |
| KIF5B | AL365181.3 | 0.320056105 | 4.91E-05 |
| SQSTM1 | AL365181.3 | 0.413427853 | 8.96E-08 |
| ATG10 | LINC01871 | 0.351750252 | 7.19E-06 |
| ATG3 | LINC01871 | 0.317326927 | 5.74E-05 |
| ATG4B | LINC01871 | 0.352656576 | 6.78E-06 |
| ATG4D | LINC01871 | 0.301820072 | 0.000135298 |
| ATG7 | LINC01871 | 0.416264943 | 7.16E-08 |
| BAX | LINC01871 | 0.488623995 | 1.12E-10 |
| BCL2 | LINC01871 | 0.322906447 | 4.17E-05 |
| BNIP1 | LINC01871 | 0.329741936 | 2.79E-05 |
| CALCOCO2 | LINC01871 | 0.3162099 | 6.12E-05 |
| CAPN10 | LINC01871 | 0.313929883 | 6.96E-05 |
| CAPNS1 | LINC01871 | 0.404205728 | 1.83E-07 |
| CCR2 | LINC01871 | 0.3847209 | 7.71E-07 |
| CDKN2A | LINC01871 | 0.482129225 | 2.13E-10 |
| CFLAR | LINC01871 | 0.314914388 | 6.58E-05 |
| CTSD | LINC01871 | 0.421636745 | 4.66E-08 |
| CXCR4 | LINC01871 | 0.341785871 | 1.35E-05 |
| DRAM1 | LINC01871 | 0.329753475 | 2.79E-05 |
| FAS | LINC01871 | 0.309508792 | 8.90E-05 |
| HDAC6 | LINC01871 | 0.317221493 | 5.78E-05 |
| HIF1A | LINC01871 | -0.306167335 | 0.000106906 |
| IFNG | LINC01871 | 0.646056745 | 1.11E-19 |
| IKBKE | LINC01871 | 0.358157904 | 4.75E-06 |
| MTMR14 | LINC01871 | 0.479744938 | 2.68E-10 |
| NCKAP1 | LINC01871 | -0.445338156 | 6.39E-09 |
| NKX2-3 | LINC01871 | 0.389070532 | 5.64E-07 |
| PEX14 | LINC01871 | 0.381596817 | 9.63E-07 |
| RAB24 | LINC01871 | 0.481108654 | 2.35E-10 |
| RGS19 | LINC01871 | 0.30815313 | 9.59E-05 |
| ST13 | LINC01871 | -0.320836348 | 4.70E-05 |
| STK11 | LINC01871 | 0.441424642 | 8.96E-09 |
| TP53 | LINC01871 | 0.325410381 | 3.60E-05 |
| TP73 | LINC01871 | 0.342742952 | 1.27E-05 |
| TSC2 | LINC01871 | 0.348293628 | 8.95E-06 |
| USP10 | LINC01871 | -0.30031839 | 0.00014664 |
| WDR45 | LINC01871 | 0.379514621 | 1.12E-06 |
| ATG4D | COLCA1 | 0.403743613 | 1.89E-07 |
| ATG7 | COLCA1 | 0.399985608 | 2.52E-07 |
| BCL2 | COLCA1 | 0.505873881 | 1.89E-11 |
| CDKN1B | COLCA1 | 0.413420274 | 8.97E-08 |
| EEF2K | COLCA1 | 0.333206588 | 2.27E-05 |
| FAS | COLCA1 | 0.499014703 | 3.88E-11 |
| IKBKE | COLCA1 | 0.453870985 | 3.01E-09 |
| MAPK8 | COLCA1 | 0.485221387 | 1.57E-10 |
| MAPK9 | COLCA1 | 0.39367258 | 4.03E-07 |
| NKX2-3 | COLCA1 | 0.75526049 | 7.19E-30 |
| PARP1 | COLCA1 | 0.40726347 | 1.45E-07 |
| PRKAB1 | COLCA1 | 0.471857783 | 5.73E-10 |
| SQSTM1 | COLCA1 | 0.375145466 | 1.51E-06 |
| TP53 | COLCA1 | 0.360446704 | 4.08E-06 |
| TP73 | COLCA1 | 0.46612858 | 9.81E-10 |
| TSC2 | COLCA1 | 0.543507949 | 2.72E-13 |
| ULK1 | COLCA1 | 0.308806273 | 9.25E-05 |
| WDR45 | COLCA1 | 0.309761073 | 8.77E-05 |
| ATF4 | AC073896.2 | 0.303129219 | 0.000126083 |
| ATG2B | AC073896.2 | -0.324342075 | 3.83E-05 |
| ATG3 | AC073896.2 | 0.409187218 | 1.25E-07 |
| ATG4A | AC073896.2 | 0.326822506 | 3.32E-05 |
| ATG4B | AC073896.2 | 0.489687025 | 1.00E-10 |
| BAG1 | AC073896.2 | 0.317709417 | 5.62E-05 |
| BAG3 | AC073896.2 | -0.317262644 | 5.76E-05 |
| BAX | AC073896.2 | 0.654009677 | 2.79E-20 |
| BIRC5 | AC073896.2 | 0.535075124 | 7.37E-13 |
| BIRC6 | AC073896.2 | -0.314653823 | 6.68E-05 |
| BNIP1 | AC073896.2 | 0.589037035 | 7.52E-16 |
| CAPN10 | AC073896.2 | 0.402630565 | 2.06E-07 |
| CDKN2A | AC073896.2 | 0.424382458 | 3.73E-08 |
| CLN3 | AC073896.2 | 0.334013433 | 2.16E-05 |
| CXCR4 | AC073896.2 | 0.366050081 | 2.81E-06 |
| EIF4G1 | AC073896.2 | -0.340081817 | 1.49E-05 |
| FKBP1A | AC073896.2 | 0.405177019 | 1.70E-07 |
| GOPC | AC073896.2 | -0.300381051 | 0.00014615 |
| HDAC6 | AC073896.2 | 0.311303895 | 8.06E-05 |
| HIF1A | AC073896.2 | -0.357676774 | 4.90E-06 |
| ITGA6 | AC073896.2 | -0.368277049 | 2.42E-06 |
| KIF5B | AC073896.2 | -0.308388086 | 9.47E-05 |
| MAP2K7 | AC073896.2 | 0.312363578 | 7.59E-05 |
| MAPK1 | AC073896.2 | -0.33576412 | 1.95E-05 |
| MLST8 | AC073896.2 | 0.328942017 | 2.93E-05 |
| MTMR14 | AC073896.2 | 0.404658316 | 1.77E-07 |
| NCKAP1 | AC073896.2 | -0.430133612 | 2.33E-08 |
| NPC1 | AC073896.2 | -0.318453879 | 5.38E-05 |
| PELP1 | AC073896.2 | 0.316989835 | 5.85E-05 |
| PEX14 | AC073896.2 | 0.386916449 | 6.59E-07 |
| PINK1 | AC073896.2 | -0.326612745 | 3.36E-05 |
| STK11 | AC073896.2 | 0.341627967 | 1.36E-05 |
| USP10 | AC073896.2 | -0.334073799 | 2.15E-05 |
| WDFY3 | AC073896.2 | -0.417634561 | 6.42E-08 |
| WDR45 | AC073896.2 | 0.565292648 | 1.82E-14 |
| AMBRA1 | AC024075.1 | 0.36627408 | 2.77E-06 |
| ARNT | AC024075.1 | 0.459103467 | 1.87E-09 |
| ARSB | AC024075.1 | 0.442348579 | 8.28E-09 |
| ATG2B | AC024075.1 | 0.310003691 | 8.66E-05 |
| BCL2 | AC024075.1 | 0.440656413 | 9.57E-09 |
| BIRC6 | AC024075.1 | 0.523729517 | 2.70E-12 |
| CAMKK2 | AC024075.1 | 0.353722873 | 6.33E-06 |
| CCR2 | AC024075.1 | 0.416118847 | 7.25E-08 |
| CD46 | AC024075.1 | 0.31691979 | 5.88E-05 |
| CDKN1B | AC024075.1 | 0.315698467 | 6.30E-05 |
| CXCR4 | AC024075.1 | 0.318922888 | 5.24E-05 |
| EEF2 | AC024075.1 | 0.306560983 | 0.000104631 |
| EEF2K | AC024075.1 | 0.475011906 | 4.24E-10 |
| EIF2AK3 | AC024075.1 | 0.489769841 | 9.96E-11 |
| HDAC6 | AC024075.1 | 0.386122448 | 6.97E-07 |
| IKBKB | AC024075.1 | 0.30129306 | 0.000139182 |
| IKBKE | AC024075.1 | 0.346009972 | 1.03E-05 |
| ITPR1 | AC024075.1 | 0.363136862 | 3.42E-06 |
| KLHL24 | AC024075.1 | 0.406811665 | 1.50E-07 |
| MAP2K7 | AC024075.1 | 0.363824983 | 3.26E-06 |
| MAPK1 | AC024075.1 | 0.344485694 | 1.14E-05 |
| MAPK8 | AC024075.1 | 0.334938683 | 2.05E-05 |
| MBTPS2 | AC024075.1 | 0.307461987 | 9.96E-05 |
| MTOR | AC024075.1 | 0.368800634 | 2.34E-06 |
| NBR1 | AC024075.1 | 0.445365474 | 6.37E-09 |
| PARP1 | AC024075.1 | 0.365104586 | 3.00E-06 |
| PIK3C3 | AC024075.1 | 0.317945448 | 5.54E-05 |
| PIK3R4 | AC024075.1 | 0.46720039 | 8.88E-10 |
| PRKAB1 | AC024075.1 | 0.327952379 | 3.10E-05 |
| RAF1 | AC024075.1 | 0.302629647 | 0.000129528 |
| RB1CC1 | AC024075.1 | 0.358128341 | 4.76E-06 |
| SIRT1 | AC024075.1 | 0.400307394 | 2.46E-07 |
| SPNS1 | AC024075.1 | 0.325217454 | 3.64E-05 |
| TP53 | AC024075.1 | 0.352279542 | 6.95E-06 |
| TP73 | AC024075.1 | 0.397589707 | 3.01E-07 |
| TSC1 | AC024075.1 | 0.478981564 | 2.89E-10 |
| TSC2 | AC024075.1 | 0.415620326 | 7.54E-08 |
| ULK2 | AC024075.1 | 0.411071574 | 1.08E-07 |
| UVRAG | AC024075.1 | 0.342168318 | 1.31E-05 |
| BAX | AC133552.5 | 0.361969952 | 3.69E-06 |
| BIRC6 | AC133552.5 | -0.305488537 | 0.000110938 |
| CLN3 | AC133552.5 | 0.310492504 | 8.43E-05 |
| FOXO3 | AC133552.5 | -0.306862446 | 0.00010292 |
| GABARAPL2 | AC133552.5 | 0.361643789 | 3.77E-06 |
| GOPC | AC133552.5 | -0.325537443 | 3.58E-05 |
| KIF5B | AC133552.5 | -0.331386401 | 2.53E-05 |
| MAPK1 | AC133552.5 | -0.387523374 | 6.31E-07 |
| NFKB1 | AC133552.5 | -0.35635863 | 5.34E-06 |
| RB1 | AC133552.5 | -0.367254662 | 2.59E-06 |
| WDFY3 | AC133552.5 | -0.30633822 | 0.000105913 |
| WDR45 | AC133552.5 | 0.323634276 | 4.00E-05 |
| ATG10 | AC008763.1 | 0.356736116 | 5.21E-06 |
| ATG16L2 | AC008763.1 | 0.305540279 | 0.000110626 |
| ATG3 | AC008763.1 | 0.364833008 | 3.05E-06 |
| ATG4B | AC008763.1 | 0.321881654 | 4.42E-05 |
| ATG4C | AC008763.1 | 0.389273042 | 5.56E-07 |
| ATG4D | AC008763.1 | 0.42980632 | 2.39E-08 |
| BCL2 | AC008763.1 | 0.325308903 | 3.62E-05 |
| BNIP3L | AC008763.1 | 0.312897597 | 7.37E-05 |
| CAPN10 | AC008763.1 | 0.397769543 | 2.97E-07 |
| CD46 | AC008763.1 | 0.307558038 | 9.91E-05 |
| CDKN1B | AC008763.1 | 0.389080474 | 5.63E-07 |
| CDKN2A | AC008763.1 | 0.355226907 | 5.75E-06 |
| CHMP4B | AC008763.1 | 0.318924055 | 5.24E-05 |
| CXCR4 | AC008763.1 | 0.334936988 | 2.05E-05 |
| EEF2K | AC008763.1 | 0.365822646 | 2.86E-06 |
| HDAC1 | AC008763.1 | 0.319753588 | 5.00E-05 |
| HDAC6 | AC008763.1 | 0.606543735 | 6.05E-17 |
| IKBKB | AC008763.1 | 0.397550985 | 3.02E-07 |
| IKBKE | AC008763.1 | 0.320772822 | 4.71E-05 |
| MAP2K7 | AC008763.1 | 0.570362833 | 9.41E-15 |
| MAPK8 | AC008763.1 | 0.395993438 | 3.39E-07 |
| MAPK9 | AC008763.1 | 0.383565817 | 8.37E-07 |
| MTMR14 | AC008763.1 | 0.361323647 | 3.85E-06 |
| NKX2-3 | AC008763.1 | 0.519487695 | 4.33E-12 |
| PARP1 | AC008763.1 | 0.326789446 | 3.32E-05 |
| PEX14 | AC008763.1 | 0.384884705 | 7.62E-07 |
| PRKAB1 | AC008763.1 | 0.398678459 | 2.78E-07 |
| RAF1 | AC008763.1 | 0.421118511 | 4.86E-08 |
| STK11 | AC008763.1 | 0.479476075 | 2.76E-10 |
| TP53 | AC008763.1 | 0.372918921 | 1.76E-06 |
| TP73 | AC008763.1 | 0.423073555 | 4.15E-08 |
| TSC1 | AC008763.1 | 0.341002936 | 1.41E-05 |
| TSC2 | AC008763.1 | 0.49733485 | 4.61E-11 |
| ULK3 | AC008763.1 | 0.399441541 | 2.62E-07 |
| WDR45 | AC008763.1 | 0.376701959 | 1.36E-06 |
| BAK1 | AL109976.1 | -0.373876058 | 1.65E-06 |
| BIRC6 | AL109976.1 | 0.331864802 | 2.46E-05 |
| EIF2AK3 | AL109976.1 | 0.507561706 | 1.58E-11 |
| FOXO3 | AL109976.1 | 0.331555179 | 2.51E-05 |
| GABARAPL1 | AL109976.1 | 0.411265065 | 1.06E-07 |
| ITGA3 | AL109976.1 | -0.37026984 | 2.11E-06 |
| KLHL24 | AL109976.1 | 0.398066118 | 2.91E-07 |
| MBTPS2 | AL109976.1 | 0.300610691 | 0.000144365 |
| NFE2L2 | AL109976.1 | 0.371847179 | 1.90E-06 |
| PIK3R4 | AL109976.1 | 0.310906325 | 8.24E-05 |
| RB1CC1 | AL109976.1 | 0.334625251 | 2.08E-05 |
| TP53INP2 | AL109976.1 | 0.428569539 | 2.65E-08 |
| TP63 | AL109976.1 | 0.302600603 | 0.000129731 |
| ULK1 | AL109976.1 | 0.300623791 | 0.000144264 |
| VAMP3 | AL109976.1 | -0.36007695 | 4.19E-06 |
| WIPI1 | AL109976.1 | 0.316821415 | 5.91E-05 |
| ARNT | UBL7-AS1 | 0.359075936 | 4.47E-06 |
| ARSB | UBL7-AS1 | 0.334066261 | 2.16E-05 |
| ATF6 | UBL7-AS1 | 0.304582044 | 0.000116544 |
| ATG10 | UBL7-AS1 | 0.342989755 | 1.25E-05 |
| ATG4C | UBL7-AS1 | 0.419083623 | 5.72E-08 |
| ATG4D | UBL7-AS1 | 0.305475098 | 0.000111019 |
| ATG7 | UBL7-AS1 | 0.343604229 | 1.20E-05 |
| BCL2 | UBL7-AS1 | 0.399091508 | 2.69E-07 |
| BECN1 | UBL7-AS1 | 0.318018365 | 5.52E-05 |
| CASP3 | UBL7-AS1 | 0.360889697 | 3.97E-06 |
| CD46 | UBL7-AS1 | 0.386229447 | 6.92E-07 |
| CDKN1B | UBL7-AS1 | 0.494084211 | 6.43E-11 |
| EEF2 | UBL7-AS1 | 0.314401826 | 6.77E-05 |
| EIF2AK3 | UBL7-AS1 | 0.386942654 | 6.57E-07 |
| HDAC6 | UBL7-AS1 | 0.457986729 | 2.07E-09 |
| IFNG | UBL7-AS1 | 0.328068294 | 3.08E-05 |
| IKBKE | UBL7-AS1 | 0.384626861 | 7.76E-07 |
| KLHL24 | UBL7-AS1 | 0.361854398 | 3.72E-06 |
| MAP2K7 | UBL7-AS1 | 0.403788505 | 1.89E-07 |
| MAPK8 | UBL7-AS1 | 0.342334205 | 1.30E-05 |
| MAPK9 | UBL7-AS1 | 0.300383841 | 0.000146128 |
| MTMR14 | UBL7-AS1 | 0.432902067 | 1.85E-08 |
| NBR1 | UBL7-AS1 | 0.334393115 | 2.11E-05 |
| NKX2-3 | UBL7-AS1 | 0.366998923 | 2.64E-06 |
| PARP1 | UBL7-AS1 | 0.401482906 | 2.25E-07 |
| PIK3C3 | UBL7-AS1 | 0.495455505 | 5.59E-11 |
| PIK3R4 | UBL7-AS1 | 0.389901184 | 5.31E-07 |
| PINK1 | UBL7-AS1 | -0.377995583 | 1.24E-06 |
| PRKAB1 | UBL7-AS1 | 0.317898081 | 5.56E-05 |
| PTK6 | UBL7-AS1 | -0.350117862 | 7.98E-06 |
| RAB33B | UBL7-AS1 | 0.301272619 | 0.000139335 |
| RAF1 | UBL7-AS1 | 0.516784737 | 5.83E-12 |
| SIRT1 | UBL7-AS1 | 0.374968414 | 1.53E-06 |
| TP53 | UBL7-AS1 | 0.380084169 | 1.07E-06 |
| TSC1 | UBL7-AS1 | 0.382098458 | 9.29E-07 |
| TSC2 | UBL7-AS1 | 0.302237649 | 0.000132292 |
| ULK3 | UBL7-AS1 | 0.483469002 | 1.86E-10 |
| UVRAG | UBL7-AS1 | 0.34369519 | 1.20E-05 |
| VAMP7 | UBL7-AS1 | 0.437232182 | 1.28E-08 |
| ATG12 | AC021078.1 | 0.301477179 | 0.000137813 |
| ATG16L2 | AC021078.1 | 0.303314779 | 0.000124825 |
| VEGFA | AC021078.1 | 0.388168026 | 6.02E-07 |
| ATG16L2 | AC084824.4 | 0.340604694 | 1.45E-05 |
| CAPN10 | AC084824.4 | 0.367031094 | 2.63E-06 |
| CDKN1B | AC084824.4 | 0.35469129 | 5.95E-06 |
| CXCR4 | AC084824.4 | 0.328601897 | 2.99E-05 |
| MAP2K7 | AC084824.4 | 0.351624269 | 7.24E-06 |
| TSC2 | AC084824.4 | 0.332065135 | 2.43E-05 |
| FAS | G2E3-AS1 | -0.373621112 | 1.68E-06 |
| NCKAP1 | G2E3-AS1 | 0.355685246 | 5.58E-06 |
| SH3GLB1 | G2E3-AS1 | -0.395706545 | 3.47E-07 |
| TM9SF1 | G2E3-AS1 | 0.346961464 | 9.74E-06 |
| TP73 | G2E3-AS1 | -0.312867286 | 7.38E-05 |
| ATG4B | AL035413.1 | 0.345461127 | 1.07E-05 |
| BAG3 | AL035413.1 | -0.359604121 | 4.32E-06 |
| BAX | AL035413.1 | 0.523666328 | 2.72E-12 |
| BIRC5 | AL035413.1 | 0.407933835 | 1.37E-07 |
| BNIP1 | AL035413.1 | 0.453268889 | 3.17E-09 |
| CAPN10 | AL035413.1 | 0.335231069 | 2.01E-05 |
| CAPN2 | AL035413.1 | -0.311350262 | 8.03E-05 |
| CXCR4 | AL035413.1 | 0.318416661 | 5.40E-05 |
| DDIT3 | AL035413.1 | 0.381576172 | 9.64E-07 |
| EIF4G1 | AL035413.1 | -0.388274269 | 5.97E-07 |
| FKBP1A | AL035413.1 | 0.379404765 | 1.12E-06 |
| HIF1A | AL035413.1 | -0.32098016 | 4.66E-05 |
| NCKAP1 | AL035413.1 | -0.382671681 | 8.92E-07 |
| PEX14 | AL035413.1 | 0.399319505 | 2.65E-07 |
| TM9SF1 | AL035413.1 | -0.307219902 | 0.000100924 |
| USP10 | AL035413.1 | -0.380038388 | 1.07E-06 |
| WDR45 | AL035413.1 | 0.340293429 | 1.48E-05 |
| BID | DLGAP1-AS2 | 0.325048145 | 3.68E-05 |
| CAPN10 | DLGAP1-AS2 | 0.337974105 | 1.70E-05 |
| DIRAS3 | DLGAP1-AS2 | 0.539005136 | 4.65E-13 |
| FKBP1B | DLGAP1-AS2 | 0.376357197 | 1.39E-06 |
| GABARAPL1 | DLGAP1-AS2 | 0.3069345 | 0.000102515 |
| PELP1 | DLGAP1-AS2 | 0.431733586 | 2.04E-08 |
| ATF4 | CH17-340M24.3 | 0.406850379 | 1.49E-07 |
| ATG4A | CH17-340M24.3 | 0.423215777 | 4.10E-08 |
| ATG4B | CH17-340M24.3 | 0.3954944 | 3.52E-07 |
| BAG1 | CH17-340M24.3 | 0.354267425 | 6.11E-06 |
| BAG3 | CH17-340M24.3 | -0.336006901 | 1.92E-05 |
| BAX | CH17-340M24.3 | 0.648599114 | 7.18E-20 |
| BIRC5 | CH17-340M24.3 | 0.460471676 | 1.65E-09 |
| BIRC6 | CH17-340M24.3 | -0.314226936 | 6.84E-05 |
| BNIP1 | CH17-340M24.3 | 0.530653906 | 1.23E-12 |
| CAPN10 | CH17-340M24.3 | 0.381839142 | 9.47E-07 |
| CAPN2 | CH17-340M24.3 | -0.324894977 | 3.71E-05 |
| CDKN2A | CH17-340M24.3 | 0.315987843 | 6.19E-05 |
| CHMP4B | CH17-340M24.3 | 0.312217367 | 7.66E-05 |
| CLN3 | CH17-340M24.3 | 0.545913629 | 2.04E-13 |
| DDIT3 | CH17-340M24.3 | 0.414169013 | 8.45E-08 |
| EIF4G1 | CH17-340M24.3 | -0.312248932 | 7.64E-05 |
| FKBP1A | CH17-340M24.3 | 0.343791835 | 1.19E-05 |
| GABARAP | CH17-340M24.3 | 0.397228655 | 3.09E-07 |
| GOPC | CH17-340M24.3 | -0.342590775 | 1.28E-05 |
| HDAC6 | CH17-340M24.3 | 0.309300475 | 9.00E-05 |
| HGS | CH17-340M24.3 | -0.337174615 | 1.79E-05 |
| HIF1A | CH17-340M24.3 | -0.325330665 | 3.62E-05 |
| ITGA6 | CH17-340M24.3 | -0.402579158 | 2.07E-07 |
| ITGB1 | CH17-340M24.3 | -0.33942626 | 1.56E-05 |
| ITGB4 | CH17-340M24.3 | -0.363040357 | 3.44E-06 |
| KIF5B | CH17-340M24.3 | -0.33008412 | 2.74E-05 |
| MAPK1 | CH17-340M24.3 | -0.386744846 | 6.67E-07 |
| MAPK8IP1 | CH17-340M24.3 | 0.325564281 | 3.57E-05 |
| MTMR14 | CH17-340M24.3 | 0.32633485 | 3.41E-05 |
| NAF1 | CH17-340M24.3 | -0.386248509 | 6.91E-07 |
| NCKAP1 | CH17-340M24.3 | -0.3747112 | 1.56E-06 |
| PEX14 | CH17-340M24.3 | 0.344854072 | 1.11E-05 |
| RAB1A | CH17-340M24.3 | -0.305830993 | 0.000108886 |
| RAB24 | CH17-340M24.3 | 0.340010804 | 1.50E-05 |
| TP63 | CH17-340M24.3 | -0.304241138 | 0.00011872 |
| USP10 | CH17-340M24.3 | -0.360247979 | 4.14E-06 |
| WDFY3 | CH17-340M24.3 | -0.306784149 | 0.000103362 |
| WDR45 | CH17-340M24.3 | 0.533948186 | 8.40E-13 |
| ATG4B | AC024896.1 | 0.397179051 | 3.11E-07 |
| ATG4C | AC024896.1 | 0.392904013 | 4.26E-07 |
| BAX | AC024896.1 | 0.311217495 | 8.09E-05 |
| BCL2 | AC024896.1 | 0.40168246 | 2.22E-07 |
| CAPN10 | AC024896.1 | 0.422078149 | 4.50E-08 |
| CASP3 | AC024896.1 | 0.362508165 | 3.56E-06 |
| CDKN1B | AC024896.1 | 0.404159842 | 1.84E-07 |
| CDKN2A | AC024896.1 | 0.306487333 | 0.000105053 |
| CX3CL1 | AC024896.1 | 0.371325374 | 1.97E-06 |
| CXCR4 | AC024896.1 | 0.575465412 | 4.79E-15 |
| DAPK2 | AC024896.1 | 0.360820153 | 3.99E-06 |
| FAS | AC024896.1 | 0.436511301 | 1.36E-08 |
| HDAC6 | AC024896.1 | 0.390512739 | 5.08E-07 |
| IKBKB | AC024896.1 | 0.301443684 | 0.000138061 |
| IKBKE | AC024896.1 | 0.46067463 | 1.62E-09 |
| MAP1LC3B | AC024896.1 | -0.324908778 | 3.71E-05 |
| MAPK8 | AC024896.1 | 0.326214427 | 3.44E-05 |
| MTMR14 | AC024896.1 | 0.326752432 | 3.33E-05 |
| NKX2-3 | AC024896.1 | 0.439334368 | 1.07E-08 |
| PARP1 | AC024896.1 | 0.397915994 | 2.94E-07 |
| PINK1 | AC024896.1 | -0.420367167 | 5.16E-08 |
| PRKAB1 | AC024896.1 | 0.396489671 | 3.27E-07 |
| TP53 | AC024896.1 | 0.459998421 | 1.73E-09 |
| TP73 | AC024896.1 | 0.438672462 | 1.13E-08 |
| TSC2 | AC024896.1 | 0.612170657 | 2.60E-17 |
| ULK3 | AC024896.1 | 0.367748823 | 2.51E-06 |
| WDR45 | AC024896.1 | 0.371231901 | 1.98E-06 |
| ATG10 | ZNF667-AS1 | 0.400710543 | 2.38E-07 |
| ATG4A | ZNF667-AS1 | 0.32980392 | 2.78E-05 |
| ATG4C | ZNF667-AS1 | 0.346811311 | 9.83E-06 |
| BCL2 | ZNF667-AS1 | 0.348657361 | 8.75E-06 |
| CDKN1B | ZNF667-AS1 | 0.342038229 | 1.32E-05 |
| CDKN2A | ZNF667-AS1 | 0.351983796 | 7.08E-06 |
| CXCR4 | ZNF667-AS1 | 0.344179129 | 1.16E-05 |
| DRAM1 | ZNF667-AS1 | 0.301336841 | 0.000138855 |
| EEF2K | ZNF667-AS1 | 0.315182625 | 6.48E-05 |
| HDAC6 | ZNF667-AS1 | 0.456570348 | 2.36E-09 |
| IKBKE | ZNF667-AS1 | 0.386117207 | 6.98E-07 |
| MTMR14 | ZNF667-AS1 | 0.304129386 | 0.000119441 |
| NKX2-3 | ZNF667-AS1 | 0.333140616 | 2.28E-05 |
| PARP1 | ZNF667-AS1 | 0.352100994 | 7.03E-06 |
| PRKAB1 | ZNF667-AS1 | 0.336488772 | 1.86E-05 |
| TP53 | ZNF667-AS1 | 0.436190497 | 1.40E-08 |
| TP73 | ZNF667-AS1 | 0.393203917 | 4.17E-07 |
| TSC2 | ZNF667-AS1 | 0.519718708 | 4.22E-12 |
| WDR45 | ZNF667-AS1 | 0.304509925 | 0.000117001 |
| BCL2 | PSORS1C3 | 0.306029686 | 0.000107713 |
| CDKN2A | PSORS1C3 | 0.326779618 | 3.33E-05 |
| FOXO3 | PSORS1C3 | 0.304266847 | 0.000118554 |
| PRKAB1 | PSORS1C3 | 0.350611808 | 7.73E-06 |
| VEGFA | AC104695.3 | 0.387749724 | 6.20E-07 |
| ARSB | LINC02381 | 0.324763144 | 3.74E-05 |
| BCL2 | LINC02381 | 0.366646693 | 2.70E-06 |
| BNIP1 | LINC02381 | 0.303158038 | 0.000125887 |
| CAPN2 | LINC02381 | -0.328116101 | 3.07E-05 |
| CXCR4 | LINC02381 | 0.466571388 | 9.42E-10 |
| DRAM1 | LINC02381 | 0.371943891 | 1.89E-06 |
| FAS | LINC02381 | 0.328857221 | 2.94E-05 |
| HDAC6 | LINC02381 | 0.322461684 | 4.28E-05 |
| IKBKB | LINC02381 | 0.332599203 | 2.35E-05 |
| KLHL24 | LINC02381 | 0.312555395 | 7.51E-05 |
| MAPK9 | LINC02381 | 0.406178942 | 1.57E-07 |
| MTMR14 | LINC02381 | 0.307592046 | 9.89E-05 |
| PTK6 | LINC02381 | -0.375992791 | 1.43E-06 |
| SIRT1 | LINC02381 | 0.407940802 | 1.37E-07 |
| ULK2 | LINC02381 | 0.433868813 | 1.70E-08 |
| UVRAG | LINC02381 | 0.422050538 | 4.51E-08 |
| WDR45 | LINC02381 | 0.370889712 | 2.03E-06 |
| ITGB1 | AC108865.1 | 0.43517921 | 1.53E-08 |
| ARSB | LINC02323 | -0.379820733 | 1.09E-06 |
| BAK1 | LINC02323 | 0.367164467 | 2.61E-06 |
| BCL2 | LINC02323 | -0.31678009 | 5.92E-05 |
| CD46 | LINC02323 | -0.312569446 | 7.51E-05 |
| CDKN1B | LINC02323 | -0.350106757 | 7.98E-06 |
| CXCR4 | LINC02323 | -0.330396909 | 2.69E-05 |
| DRAM1 | LINC02323 | -0.303269204 | 0.000125133 |
| EEF2 | LINC02323 | -0.32395766 | 3.92E-05 |
| EIF2S1 | LINC02323 | 0.304386341 | 0.000117788 |
| GAA | LINC02323 | -0.319373756 | 5.11E-05 |
| HDAC6 | LINC02323 | -0.311783893 | 7.84E-05 |
| KLHL24 | LINC02323 | -0.364902006 | 3.04E-06 |
| MAPK9 | LINC02323 | -0.383749256 | 8.26E-07 |
| NBR1 | LINC02323 | -0.37765428 | 1.27E-06 |
| PARP1 | LINC02323 | -0.321987444 | 4.40E-05 |
| PTK6 | LINC02323 | 0.534701863 | 7.70E-13 |
| SIRT1 | LINC02323 | -0.33439317 | 2.11E-05 |
| SQSTM1 | LINC02323 | -0.307048181 | 0.000101878 |
| ULK2 | LINC02323 | -0.341700322 | 1.35E-05 |
| UVRAG | LINC02323 | -0.324594311 | 3.78E-05 |
| CCL2 | AC005288.1 | 0.409259707 | 1.24E-07 |
| ERBB2 | AC005288.1 | 0.344590076 | 1.13E-05 |
| ARNT | FGD5-AS1 | 0.499716668 | 3.60E-11 |
| ARSB | FGD5-AS1 | 0.490095963 | 9.64E-11 |
| ATG10 | FGD5-AS1 | 0.405220937 | 1.69E-07 |
| ATG2A | FGD5-AS1 | 0.371295799 | 1.97E-06 |
| ATG3 | FGD5-AS1 | 0.337928585 | 1.71E-05 |
| ATG4C | FGD5-AS1 | 0.549917977 | 1.25E-13 |
| ATG7 | FGD5-AS1 | 0.59174035 | 5.15E-16 |
| BCL2 | FGD5-AS1 | 0.524270178 | 2.54E-12 |
| BECN1 | FGD5-AS1 | 0.381095427 | 9.98E-07 |
| BIRC6 | FGD5-AS1 | 0.390787104 | 4.98E-07 |
| BNIP3 | FGD5-AS1 | -0.37618823 | 1.41E-06 |
| BNIP3L | FGD5-AS1 | 0.303439158 | 0.000123989 |
| CAMKK2 | FGD5-AS1 | 0.368674083 | 2.36E-06 |
| CASP3 | FGD5-AS1 | 0.388313727 | 5.96E-07 |
| CASP8 | FGD5-AS1 | 0.32202184 | 4.39E-05 |
| CCR2 | FGD5-AS1 | 0.314123203 | 6.88E-05 |
| CD46 | FGD5-AS1 | 0.481050244 | 2.36E-10 |
| CDKN1B | FGD5-AS1 | 0.51177651 | 1.01E-11 |
| CFLAR | FGD5-AS1 | 0.348401265 | 8.89E-06 |
| CHMP2B | FGD5-AS1 | 0.377511646 | 1.28E-06 |
| CXCR4 | FGD5-AS1 | 0.324849941 | 3.72E-05 |
| DRAM1 | FGD5-AS1 | 0.399837625 | 2.55E-07 |
| EDEM1 | FGD5-AS1 | 0.540410294 | 3.94E-13 |
| EEF2K | FGD5-AS1 | 0.439220059 | 1.08E-08 |
| EIF2AK3 | FGD5-AS1 | 0.521613009 | 3.42E-12 |
| FAS | FGD5-AS1 | 0.477749736 | 3.26E-10 |
| FOXO3 | FGD5-AS1 | 0.320141581 | 4.89E-05 |
| HDAC1 | FGD5-AS1 | 0.307920255 | 9.71E-05 |
| HDAC6 | FGD5-AS1 | 0.512860121 | 8.94E-12 |
| IKBKB | FGD5-AS1 | 0.325676315 | 3.55E-05 |
| IKBKE | FGD5-AS1 | 0.455245703 | 2.66E-09 |
| ITPR1 | FGD5-AS1 | 0.36557914 | 2.90E-06 |
| KLHL24 | FGD5-AS1 | 0.432177299 | 1.96E-08 |
| MAP2K7 | FGD5-AS1 | 0.375087028 | 1.52E-06 |
| MAPK8 | FGD5-AS1 | 0.580799086 | 2.34E-15 |
| MAPK9 | FGD5-AS1 | 0.357332381 | 5.01E-06 |
| MTMR14 | FGD5-AS1 | 0.58338395 | 1.64E-15 |
| MTOR | FGD5-AS1 | 0.452081279 | 3.53E-09 |
| NAF1 | FGD5-AS1 | 0.342575187 | 1.28E-05 |
| NBR1 | FGD5-AS1 | 0.478838915 | 2.93E-10 |
| NFKB1 | FGD5-AS1 | 0.356008353 | 5.46E-06 |
| NKX2-3 | FGD5-AS1 | 0.410970809 | 1.09E-07 |
| PARP1 | FGD5-AS1 | 0.538829856 | 4.75E-13 |
| PIK3R4 | FGD5-AS1 | 0.600265076 | 1.52E-16 |
| PRKAB1 | FGD5-AS1 | 0.357491296 | 4.96E-06 |
| PRKAR1A | FGD5-AS1 | 0.342734088 | 1.27E-05 |
| PRKCD | FGD5-AS1 | 0.374405637 | 1.59E-06 |
| RAB33B | FGD5-AS1 | 0.486675051 | 1.36E-10 |
| RAB5A | FGD5-AS1 | 0.645159463 | 1.30E-19 |
| RAF1 | FGD5-AS1 | 0.846495138 | 9.87E-44 |
| RB1CC1 | FGD5-AS1 | 0.341720618 | 1.35E-05 |
| RPS6KB1 | FGD5-AS1 | 0.436111012 | 1.41E-08 |
| SH3GLB1 | FGD5-AS1 | 0.304747854 | 0.000115499 |
| SIRT1 | FGD5-AS1 | 0.531183477 | 1.16E-12 |
| TP53 | FGD5-AS1 | 0.467951582 | 8.28E-10 |
| TP73 | FGD5-AS1 | 0.381988889 | 9.37E-07 |
| TSC1 | FGD5-AS1 | 0.448049223 | 5.04E-09 |
| TSC2 | FGD5-AS1 | 0.426436292 | 3.16E-08 |
| ULK2 | FGD5-AS1 | 0.366031481 | 2.82E-06 |
| ULK3 | FGD5-AS1 | 0.343218001 | 1.23E-05 |
| IFNG | LINC01291 | 0.412881227 | 9.35E-08 |
| NKX2-3 | LINC01291 | 0.355847162 | 5.52E-06 |
| TP73 | LINC01291 | 0.313647 | 7.07E-05 |
| ATG9B | AC093159.1 | 0.352181099 | 6.99E-06 |
| DAPK2 | AC093159.1 | 0.350192567 | 7.94E-06 |
| RAB24 | AC093159.1 | 0.3051008 | 0.000113304 |
| ATG3 | LINC00957 | 0.41000981 | 1.17E-07 |
| CDKN2A | LINC00957 | 0.32561685 | 3.56E-05 |
| LAMP2 | LINC00957 | -0.310920404 | 8.23E-05 |
| MTMR14 | LINC00957 | 0.321437469 | 4.54E-05 |
| RAF1 | LINC00957 | 0.367414456 | 2.57E-06 |
| ULK3 | LINC00957 | 0.385729234 | 7.17E-07 |
| CAPN10 | MIAT | 0.408274138 | 1.34E-07 |
| DIRAS3 | MIAT | 0.823986176 | 1.39E-39 |
| FKBP1B | MIAT | 0.331403402 | 2.53E-05 |
| PRKCQ | MIAT | 0.308185264 | 9.57E-05 |
| ATG16L2 | AC093227.1 | 0.432557969 | 1.90E-08 |
| ATG4C | AC093227.1 | 0.313432068 | 7.15E-05 |
| CASP8 | AC093227.1 | 0.341277195 | 1.39E-05 |
| CFLAR | AC093227.1 | 0.334355281 | 2.12E-05 |
| CXCR4 | AC093227.1 | 0.393884973 | 3.96E-07 |
| DAPK2 | AC093227.1 | 0.383400728 | 8.47E-07 |
| HDAC6 | AC093227.1 | 0.392343779 | 4.44E-07 |
| IKBKB | AC093227.1 | 0.345782894 | 1.05E-05 |
| RAF1 | AC093227.1 | 0.300845453 | 0.000142562 |
| SIRT2 | AC093227.1 | 0.347146869 | 9.63E-06 |
| TP73 | AC093227.1 | 0.326948832 | 3.29E-05 |
| TSC2 | AC093227.1 | 0.451148889 | 3.83E-09 |
| ULK3 | AC093227.1 | 0.412231471 | 9.84E-08 |
| UVRAG | AC093227.1 | 0.337016054 | 1.80E-05 |
| DDIT3 | LINC02428 | 0.354318373 | 6.09E-06 |
| DIRAS3 | LINC02428 | 0.319630465 | 5.03E-05 |
| MAP1LC3B | LINC02428 | 0.410928207 | 1.09E-07 |
| RAB11A | LINC02487 | 0.315979103 | 6.20E-05 |
| ATF4 | AC002398.1 | 0.366948992 | 2.65E-06 |
| ATF6 | AC002398.1 | -0.325911214 | 3.50E-05 |
| ATG10 | AC002398.1 | 0.336249286 | 1.89E-05 |
| ATG12 | AC002398.1 | 0.332521361 | 2.37E-05 |
| ATG16L2 | AC002398.1 | 0.391146228 | 4.85E-07 |
| ATG4B | AC002398.1 | 0.537385364 | 5.62E-13 |
| ATG7 | AC002398.1 | 0.330867768 | 2.61E-05 |
| BAG1 | AC002398.1 | 0.307420026 | 9.98E-05 |
| BAG3 | AC002398.1 | -0.367105791 | 2.62E-06 |
| BAX | AC002398.1 | 0.648655626 | 7.11E-20 |
| BIRC5 | AC002398.1 | 0.367028184 | 2.63E-06 |
| BIRC6 | AC002398.1 | -0.375406548 | 1.49E-06 |
| BNIP1 | AC002398.1 | 0.553831304 | 7.74E-14 |
| CANX | AC002398.1 | -0.302981305 | 0.000127094 |
| CAPN10 | AC002398.1 | 0.538700722 | 4.82E-13 |
| CAPNS1 | AC002398.1 | 0.405142995 | 1.70E-07 |
| CDKN2A | AC002398.1 | 0.388830733 | 5.74E-07 |
| CLN3 | AC002398.1 | 0.3263083 | 3.42E-05 |
| CXCR4 | AC002398.1 | 0.516943082 | 5.73E-12 |
| DAPK2 | AC002398.1 | 0.393691356 | 4.02E-07 |
| EIF4G1 | AC002398.1 | -0.408949276 | 1.27E-07 |
| FKBP1A | AC002398.1 | 0.376121479 | 1.41E-06 |
| GABARAP | AC002398.1 | 0.308277162 | 9.52E-05 |
| HGS | AC002398.1 | -0.305934055 | 0.000108276 |
| ITGA6 | AC002398.1 | -0.311840273 | 7.82E-05 |
| ITGB1 | AC002398.1 | -0.304918896 | 0.00011443 |
| KIF5B | AC002398.1 | -0.394417515 | 3.81E-07 |
| MAP1LC3B | AC002398.1 | -0.317651674 | 5.64E-05 |
| MAP2K7 | AC002398.1 | 0.311652546 | 7.90E-05 |
| MAPK1 | AC002398.1 | -0.404892868 | 1.74E-07 |
| MBTPS2 | AC002398.1 | -0.319791245 | 4.99E-05 |
| MTMR14 | AC002398.1 | 0.458089524 | 2.06E-09 |
| NCKAP1 | AC002398.1 | -0.433628158 | 1.74E-08 |
| PELP1 | AC002398.1 | 0.325998286 | 3.48E-05 |
| PEX14 | AC002398.1 | 0.509682393 | 1.26E-11 |
| RAB1A | AC002398.1 | -0.322133469 | 4.36E-05 |
| RAB24 | AC002398.1 | 0.395266274 | 3.58E-07 |
| STK11 | AC002398.1 | 0.380104448 | 1.07E-06 |
| TP63 | AC002398.1 | -0.339418625 | 1.56E-05 |
| TP73 | AC002398.1 | 0.304839671 | 0.000114924 |
| TSC2 | AC002398.1 | 0.38528822 | 7.40E-07 |
| USP10 | AC002398.1 | -0.378635547 | 1.19E-06 |
| WDFY3 | AC002398.1 | -0.420249703 | 5.21E-08 |
| WDR45 | AC002398.1 | 0.478411795 | 3.06E-10 |
| ATG10 | MAST4-AS1 | 0.366827298 | 2.67E-06 |
| ATG3 | MAST4-AS1 | 0.309492248 | 8.91E-05 |
| CDKN2A | MAST4-AS1 | 0.301931423 | 0.00013449 |
| HDAC1 | MAST4-AS1 | 0.329987707 | 2.75E-05 |
| IKBKE | MAST4-AS1 | 0.302746159 | 0.000128717 |
| MTMR14 | MAST4-AS1 | 0.320959206 | 4.66E-05 |
| PINK1 | MAST4-AS1 | -0.365857129 | 2.85E-06 |
| RAF1 | MAST4-AS1 | 0.325450055 | 3.59E-05 |
| ULK3 | MAST4-AS1 | 0.36040217 | 4.10E-06 |
| FKBP1B | AC079949.2 | 0.316141145 | 6.14E-05 |
| ITGB1 | AC079949.2 | 0.475436145 | 4.07E-10 |
| ITGB4 | AC079949.2 | 0.301782205 | 0.000135574 |
| MYC | AC079949.2 | 0.315677699 | 6.30E-05 |
| NAF1 | AC079949.2 | 0.379559151 | 1.11E-06 |
| CAPNS1 | AP000695.2 | 0.302763478 | 0.000128597 |
| NKX2-3 | AP000695.2 | 0.365710945 | 2.88E-06 |
| PEX14 | AP000695.2 | 0.317897069 | 5.56E-05 |
| TP73 | AP000695.2 | 0.312378132 | 7.59E-05 |
| ATG16L2 | BACE1-AS | 0.509463548 | 1.29E-11 |
| ATG4B | BACE1-AS | 0.308557447 | 9.38E-05 |
| BID | BACE1-AS | 0.310452831 | 8.44E-05 |
| CAPN10 | BACE1-AS | 0.448227013 | 4.96E-09 |
| DAPK2 | BACE1-AS | 0.389607371 | 5.42E-07 |
| GNAI3 | BACE1-AS | -0.308387486 | 9.47E-05 |
| HDAC6 | BACE1-AS | 0.310453967 | 8.44E-05 |
| IKBKB | BACE1-AS | 0.303526486 | 0.000123405 |
| PELP1 | BACE1-AS | 0.402458026 | 2.09E-07 |
| TSC2 | BACE1-AS | 0.320078587 | 4.91E-05 |
| ULK3 | BACE1-AS | 0.374257704 | 1.61E-06 |
| UVRAG | BACE1-AS | 0.354900086 | 5.87E-06 |
| ARNT | AL162424.1 | -0.349647064 | 8.22E-06 |
| ARSB | AL162424.1 | -0.350600707 | 7.73E-06 |
| ATF6 | AL162424.1 | -0.33193522 | 2.45E-05 |
| ATG4C | AL162424.1 | -0.3007426 | 0.00014335 |
| BIRC6 | AL162424.1 | -0.341786402 | 1.35E-05 |
| CANX | AL162424.1 | -0.312470906 | 7.55E-05 |
| CD46 | AL162424.1 | -0.412893308 | 9.34E-08 |
| EEF2 | AL162424.1 | -0.310234968 | 8.55E-05 |
| EEF2K | AL162424.1 | -0.312063109 | 7.72E-05 |
| EIF2AK3 | AL162424.1 | -0.346367769 | 1.01E-05 |
| EIF4EBP1 | AL162424.1 | 0.35845861 | 4.65E-06 |
| GOPC | AL162424.1 | -0.306131336 | 0.000107117 |
| HDAC6 | AL162424.1 | -0.314457234 | 6.75E-05 |
| KLHL24 | AL162424.1 | -0.315421737 | 6.40E-05 |
| MAPK1 | AL162424.1 | -0.335064716 | 2.03E-05 |
| MAPK3 | AL162424.1 | 0.317951489 | 5.54E-05 |
| MAPK8 | AL162424.1 | -0.313187149 | 7.25E-05 |
| MAPK9 | AL162424.1 | -0.354538235 | 6.01E-06 |
| NBR1 | AL162424.1 | -0.394011548 | 3.93E-07 |
| PARP1 | AL162424.1 | -0.413110837 | 9.19E-08 |
| PIK3R4 | AL162424.1 | -0.345791798 | 1.05E-05 |
| PRKCD | AL162424.1 | -0.307406905 | 9.99E-05 |
| PTK6 | AL162424.1 | 0.439702427 | 1.04E-08 |
| RHEB | AL162424.1 | -0.303763678 | 0.000121831 |
| SH3GLB1 | AL162424.1 | -0.325704144 | 3.54E-05 |
| ST13 | AL162424.1 | -0.327890741 | 3.11E-05 |
| TP53 | AL162424.1 | -0.315073768 | 6.52E-05 |
| USP10 | AL162424.1 | -0.322108901 | 4.37E-05 |
| ATG9B | C10orf91 | 0.367818585 | 2.50E-06 |
| BID | C10orf91 | 0.487454966 | 1.26E-10 |
| FKBP1B | C10orf91 | 0.362766966 | 3.50E-06 |
| FOXO1 | C10orf91 | -0.351900772 | 7.12E-06 |
| CXCR4 | LINC02188 | 0.384075359 | 8.07E-07 |
| TSC2 | LINC02188 | 0.324342297 | 3.83E-05 |
| ATG12 | AC021028.1 | 0.321206013 | 4.60E-05 |
| ATG4C | AC021028.1 | 0.313946217 | 6.95E-05 |
| BNIP1 | AC021028.1 | 0.400496644 | 2.42E-07 |
| CFLAR | AC021028.1 | 0.364420346 | 3.14E-06 |
| FAS | AC021028.1 | 0.466862894 | 9.17E-10 |
| IKBKE | AC021028.1 | 0.325291623 | 3.63E-05 |
| MAP1LC3A | AC021028.1 | 0.429121435 | 2.53E-08 |
| MAPK8 | AC021028.1 | 0.345653345 | 1.06E-05 |
| MLST8 | AC021028.1 | 0.334054107 | 2.16E-05 |
| NKX2-3 | AC021028.1 | 0.448122851 | 5.01E-09 |
| PEX14 | AC021028.1 | 0.327121031 | 3.26E-05 |
| TP73 | AC021028.1 | 0.401163169 | 2.30E-07 |
| TSC2 | AC021028.1 | 0.356629375 | 5.25E-06 |
| WDR45 | AC021028.1 | 0.416654848 | 6.94E-08 |
| ATF4 | AP000254.1 | 0.353510667 | 6.42E-06 |
| ATG10 | AP000254.1 | 0.317129728 | 5.81E-05 |
| ATG12 | AP000254.1 | 0.402600563 | 2.07E-07 |
| ATG16L2 | AP000254.1 | 0.443771076 | 7.32E-09 |
| ATG4B | AP000254.1 | 0.563148603 | 2.39E-14 |
| ATG4C | AP000254.1 | 0.381865762 | 9.45E-07 |
| ATG4D | AP000254.1 | 0.342306971 | 1.30E-05 |
| BAG3 | AP000254.1 | -0.328993637 | 2.92E-05 |
| BAX | AP000254.1 | 0.403361914 | 1.95E-07 |
| BCL2 | AP000254.1 | 0.31756125 | 5.67E-05 |
| BNIP1 | AP000254.1 | 0.422424962 | 4.37E-08 |
| CAPN10 | AP000254.1 | 0.619375125 | 8.62E-18 |
| CXCR4 | AP000254.1 | 0.569486154 | 1.05E-14 |
| DAPK2 | AP000254.1 | 0.517907705 | 5.15E-12 |
| DRAM1 | AP000254.1 | 0.30496664 | 0.000114134 |
| EIF4G1 | AP000254.1 | -0.38237339 | 9.11E-07 |
| GABARAP | AP000254.1 | 0.402269721 | 2.12E-07 |
| HDAC6 | AP000254.1 | 0.343289484 | 1.23E-05 |
| HIF1A | AP000254.1 | -0.320749336 | 4.72E-05 |
| IKBKB | AP000254.1 | 0.313598712 | 7.09E-05 |
| ITGA6 | AP000254.1 | -0.329461185 | 2.84E-05 |
| MAP2K7 | AP000254.1 | 0.324416757 | 3.82E-05 |
| MTMR14 | AP000254.1 | 0.365422848 | 2.93E-06 |
| NCKAP1 | AP000254.1 | -0.363707638 | 3.29E-06 |
| PELP1 | AP000254.1 | 0.408020287 | 1.37E-07 |
| PEX14 | AP000254.1 | 0.325461949 | 3.59E-05 |
| PINK1 | AP000254.1 | -0.333563028 | 2.22E-05 |
| PRKAB1 | AP000254.1 | 0.308035279 | 9.65E-05 |
| RAB24 | AP000254.1 | 0.349031843 | 8.55E-06 |
| STK11 | AP000254.1 | 0.451358639 | 3.76E-09 |
| TP53 | AP000254.1 | 0.304537054 | 0.000116829 |
| TP73 | AP000254.1 | 0.313241101 | 7.23E-05 |
| TSC2 | AP000254.1 | 0.454128253 | 2.94E-09 |
| ULK3 | AP000254.1 | 0.383168496 | 8.61E-07 |
| USP10 | AP000254.1 | -0.33095696 | 2.60E-05 |
| WDFY3 | AP000254.1 | -0.331176217 | 2.56E-05 |
| WDR45 | AP000254.1 | 0.396671647 | 3.23E-07 |
| ATG4C | AC011297.1 | 0.316591848 | 5.99E-05 |
| ATIC | AC011297.1 | 0.39817843 | 2.88E-07 |
| BCL2 | AC011297.1 | 0.411156621 | 1.07E-07 |
| BIRC5 | AC011297.1 | 0.380068636 | 1.07E-06 |
| CDKN1B | AC011297.1 | 0.323638203 | 4.00E-05 |
| HDAC6 | AC011297.1 | 0.451921451 | 3.58E-09 |
| MAPK8IP1 | AC011297.1 | 0.521723822 | 3.38E-12 |
| MAPK9 | AC011297.1 | 0.323270003 | 4.08E-05 |
| PRKAR1A | AC011297.1 | 0.465102106 | 1.08E-09 |
| ULK2 | AC011297.1 | 0.559185065 | 3.96E-14 |
| VAMP7 | AC011297.1 | 0.375714908 | 1.45E-06 |
| ATIC | AC008443.4 | 0.357557191 | 4.94E-06 |
| BAG1 | AC008443.4 | 0.347446576 | 9.45E-06 |
| BAX | AC008443.4 | 0.523120616 | 2.89E-12 |
| BIRC5 | AC008443.4 | 0.626790234 | 2.68E-18 |
| BNIP1 | AC008443.4 | 0.453300013 | 3.16E-09 |
| CAPN2 | AC008443.4 | -0.337583868 | 1.74E-05 |
| EIF4EBP1 | AC008443.4 | 0.313085239 | 7.29E-05 |
| FKBP1A | AC008443.4 | 0.443067784 | 7.78E-09 |
| MAPK1 | AC008443.4 | -0.346078918 | 1.03E-05 |
| MAPK8IP1 | AC008443.4 | 0.452579514 | 3.37E-09 |
| MLST8 | AC008443.4 | 0.319269978 | 5.14E-05 |
| WDR45 | AC008443.4 | 0.443020446 | 7.81E-09 |
| ATG4A | RHOXF1-AS1 | 0.37189768 | 1.89E-06 |
| HDAC6 | RHOXF1-AS1 | 0.439650998 | 1.04E-08 |
| ATF4 | LINC02195 | 0.335834879 | 1.94E-05 |
| BAX | LINC02195 | 0.478756206 | 2.95E-10 |
| BNIP1 | LINC02195 | 0.448046331 | 5.04E-09 |
| CASP1 | LINC02195 | 0.35630849 | 5.36E-06 |
| CDKN2A | LINC02195 | 0.301163284 | 0.000140154 |
| CTSD | LINC02195 | 0.449662162 | 4.37E-09 |
| CXCR4 | LINC02195 | 0.304580947 | 0.000116551 |
| IFNG | LINC02195 | 0.535475169 | 7.03E-13 |
| KIF5B | LINC02195 | -0.312818071 | 7.40E-05 |
| MAPK1 | LINC02195 | -0.31989565 | 4.96E-05 |
| MTMR14 | LINC02195 | 0.317410172 | 5.71E-05 |
| NCKAP1 | LINC02195 | -0.459982337 | 1.73E-09 |
| PEX14 | LINC02195 | 0.302576278 | 0.000129901 |
| RAB1A | LINC02195 | -0.310997922 | 8.19E-05 |
| RAB24 | LINC02195 | 0.419403043 | 5.58E-08 |
| STK11 | LINC02195 | 0.406983787 | 1.48E-07 |
| USP10 | LINC02195 | -0.310080143 | 8.62E-05 |
| WDR45 | LINC02195 | 0.336538495 | 1.86E-05 |
| AMBRA1 | AL450326.1 | 0.330067302 | 2.74E-05 |
| ARNT | AL450326.1 | 0.538678261 | 4.83E-13 |
| ARSB | AL450326.1 | 0.543671789 | 2.67E-13 |
| ATF6 | AL450326.1 | 0.363956378 | 3.24E-06 |
| ATG2A | AL450326.1 | 0.351085446 | 7.50E-06 |
| ATG2B | AL450326.1 | 0.468244904 | 8.05E-10 |
| BCL2 | AL450326.1 | 0.331283365 | 2.55E-05 |
| BECN1 | AL450326.1 | 0.344292392 | 1.15E-05 |
| BIRC6 | AL450326.1 | 0.64587493 | 1.15E-19 |
| BNIP3 | AL450326.1 | -0.316747396 | 5.93E-05 |
| CAMKK2 | AL450326.1 | 0.413758889 | 8.73E-08 |
| CANX | AL450326.1 | 0.326003036 | 3.48E-05 |
| CCR2 | AL450326.1 | 0.343043011 | 1.24E-05 |
| CD46 | AL450326.1 | 0.322892457 | 4.17E-05 |
| CDKN1B | AL450326.1 | 0.327028127 | 3.28E-05 |
| DAPK1 | AL450326.1 | 0.317677877 | 5.63E-05 |
| DLC1 | AL450326.1 | 0.344070637 | 1.17E-05 |
| DRAM1 | AL450326.1 | 0.381950946 | 9.39E-07 |
| EDEM1 | AL450326.1 | 0.402319601 | 2.11E-07 |
| EEF2 | AL450326.1 | 0.36783575 | 2.49E-06 |
| EEF2K | AL450326.1 | 0.392714905 | 4.32E-07 |
| EIF2AK2 | AL450326.1 | 0.357714001 | 4.89E-06 |
| EIF2AK3 | AL450326.1 | 0.503458202 | 2.44E-11 |
| ERN1 | AL450326.1 | 0.340084374 | 1.49E-05 |
| FAS | AL450326.1 | 0.315950355 | 6.21E-05 |
| FOXO1 | AL450326.1 | 0.323512509 | 4.02E-05 |
| FOXO3 | AL450326.1 | 0.400901022 | 2.35E-07 |
| GOPC | AL450326.1 | 0.404198081 | 1.83E-07 |
| HDAC6 | AL450326.1 | 0.374971622 | 1.53E-06 |
| ITPR1 | AL450326.1 | 0.339498636 | 1.55E-05 |
| KIF5B | AL450326.1 | 0.384057621 | 8.08E-07 |
| KLHL24 | AL450326.1 | 0.446881743 | 5.58E-09 |
| LAMP2 | AL450326.1 | 0.316613537 | 5.98E-05 |
| MAPK1 | AL450326.1 | 0.382073069 | 9.31E-07 |
| MAPK8 | AL450326.1 | 0.375346234 | 1.49E-06 |
| MBTPS2 | AL450326.1 | 0.374512229 | 1.58E-06 |
| MTOR | AL450326.1 | 0.448961356 | 4.65E-09 |
| NAF1 | AL450326.1 | 0.30428485 | 0.000118439 |
| NBR1 | AL450326.1 | 0.579931464 | 2.63E-15 |
| NFKB1 | AL450326.1 | 0.344215316 | 1.16E-05 |
| PARP1 | AL450326.1 | 0.518605252 | 4.77E-12 |
| PIK3R4 | AL450326.1 | 0.394978691 | 3.66E-07 |
| PRKAR1A | AL450326.1 | 0.376722394 | 1.36E-06 |
| PTEN | AL450326.1 | 0.307613013 | 9.88E-05 |
| RAB33B | AL450326.1 | 0.368932188 | 2.32E-06 |
| RAB5A | AL450326.1 | 0.364465939 | 3.13E-06 |
| RAF1 | AL450326.1 | 0.381095509 | 9.98E-07 |
| RB1CC1 | AL450326.1 | 0.384875586 | 7.63E-07 |
| RPS6KB1 | AL450326.1 | 0.328521913 | 3.00E-05 |
| RPTOR | AL450326.1 | 0.353327857 | 6.49E-06 |
| SIRT1 | AL450326.1 | 0.539023653 | 4.64E-13 |
| TM9SF1 | AL450326.1 | 0.318834691 | 5.27E-05 |
| TSC1 | AL450326.1 | 0.318009594 | 5.52E-05 |
| ULK2 | AL450326.1 | 0.321301531 | 4.57E-05 |
| USP10 | AL450326.1 | 0.323774026 | 3.96E-05 |
| UVRAG | AL450326.1 | 0.394181152 | 3.88E-07 |
| VAMP7 | AL450326.1 | 0.349833584 | 8.12E-06 |
| WDFY3 | AL450326.1 | 0.404631046 | 1.77E-07 |
| BID | BAIAP2-DT | 0.361157217 | 3.90E-06 |
| EIF2AK3 | BAIAP2-DT | 0.433493406 | 1.76E-08 |
| HDAC6 | BAIAP2-DT | 0.371602531 | 1.93E-06 |
| MAPK8 | BAIAP2-DT | 0.351483194 | 7.31E-06 |
| PARP1 | BAIAP2-DT | 0.300752573 | 0.000143273 |
| PIK3R4 | BAIAP2-DT | 0.343763704 | 1.19E-05 |
| PRKAR1A | BAIAP2-DT | 0.378935244 | 1.16E-06 |
| RB1CC1 | BAIAP2-DT | 0.330201638 | 2.72E-05 |
| RPS6KB1 | BAIAP2-DT | 0.371002311 | 2.01E-06 |
| RPTOR | BAIAP2-DT | 0.426636489 | 3.10E-08 |
| WIPI1 | BAIAP2-DT | 0.320199376 | 4.87E-05 |
| ARNT | AC011468.1 | -0.365282573 | 2.96E-06 |
| ATF4 | AC011468.1 | 0.392445077 | 4.41E-07 |
| ATF6 | AC011468.1 | -0.377848187 | 1.25E-06 |
| ATG16L2 | AC011468.1 | 0.514947108 | 7.13E-12 |
| ATG4B | AC011468.1 | 0.492712927 | 7.40E-11 |
| BAX | AC011468.1 | 0.436721341 | 1.34E-08 |
| BIRC6 | AC011468.1 | -0.37521648 | 1.51E-06 |
| BNIP1 | AC011468.1 | 0.360627853 | 4.04E-06 |
| CANX | AC011468.1 | -0.36521882 | 2.97E-06 |
| CAPN10 | AC011468.1 | 0.423114002 | 4.14E-08 |
| DAPK2 | AC011468.1 | 0.33615072 | 1.90E-05 |
| EEF2 | AC011468.1 | -0.329919825 | 2.76E-05 |
| EIF4G1 | AC011468.1 | -0.40355211 | 1.92E-07 |
| FKBP1A | AC011468.1 | 0.333854264 | 2.18E-05 |
| FOXO1 | AC011468.1 | -0.32534688 | 3.62E-05 |
| KIF5B | AC011468.1 | -0.416306508 | 7.14E-08 |
| MAPK1 | AC011468.1 | -0.430180333 | 2.32E-08 |
| MBTPS2 | AC011468.1 | -0.363993804 | 3.23E-06 |
| NBR1 | AC011468.1 | -0.330625226 | 2.65E-05 |
| NCKAP1 | AC011468.1 | -0.387694989 | 6.23E-07 |
| PEX14 | AC011468.1 | 0.402422633 | 2.09E-07 |
| RAB24 | AC011468.1 | 0.459223353 | 1.85E-09 |
| RB1 | AC011468.1 | -0.30346689 | 0.000123803 |
| TP63 | AC011468.1 | -0.369961296 | 2.16E-06 |
| USP10 | AC011468.1 | -0.459747004 | 1.77E-09 |
| WDFY3 | AC011468.1 | -0.312415896 | 7.57E-05 |
| WDR45 | AC011468.1 | 0.444168649 | 7.07E-09 |
| BNIP3 | TM4SF19-AS1 | 0.343760723 | 1.19E-05 |
| HIF1A | TM4SF19-AS1 | 0.374232549 | 1.61E-06 |
| PTK6 | TM4SF19-AS1 | 0.307762168 | 9.80E-05 |
| PEX3 | AL357060.1 | 0.386995948 | 6.55E-07 |
| ATG4A | LINC00278 | 0.324401395 | 3.82E-05 |
| ATG9B | LINC00278 | 0.355266477 | 5.73E-06 |
| BECN1 | LINC00278 | 0.301030368 | 0.000141156 |
| RAB11A | LINC00278 | 0.319282665 | 5.14E-05 |
| RAB5A | LINC00278 | 0.318246489 | 5.45E-05 |
| RAF1 | LINC00278 | 0.318379375 | 5.41E-05 |
| TP53INP2 | LINC00278 | 0.357862637 | 4.84E-06 |
| ARSB | LINC00958 | -0.305301204 | 0.000112075 |
| CXCR4 | LINC00958 | -0.301065999 | 0.000140887 |
| IL24 | LINC00958 | 0.415258546 | 7.76E-08 |
| ITGA3 | LINC00958 | 0.38315993 | 8.62E-07 |
| CASP4 | MIR4713HG | 0.325151232 | 3.66E-05 |
| ITGA6 | MIR4713HG | 0.333118148 | 2.28E-05 |
| ATG4D | AL355803.1 | 0.415289152 | 7.74E-08 |
| BCL2 | AL355803.1 | 0.337498141 | 1.75E-05 |
| MAP1LC3A | AL355803.1 | 0.311396077 | 8.01E-05 |
| MAPK8 | AL355803.1 | 0.401502552 | 2.25E-07 |
| NKX2-3 | AL355803.1 | 0.489081154 | 1.07E-10 |
| PRKAB1 | AL355803.1 | 0.419959513 | 5.33E-08 |
| TSC2 | AL355803.1 | 0.330715367 | 2.63E-05 |
| CAPN10 | LRRC75A-AS1 | 0.307863778 | 9.74E-05 |
| EEF2 | LRRC75A-AS1 | 0.521806882 | 3.34E-12 |
| PELP1 | LRRC75A-AS1 | 0.356697862 | 5.22E-06 |
| ULK2 | LRRC75A-AS1 | 0.333704471 | 2.20E-05 |
| UVRAG | LRRC75A-AS1 | 0.306672961 | 0.000103992 |
| ARSB | AC022509.3 | 0.358353896 | 4.69E-06 |
| FAS | AC022509.3 | 0.309397704 | 8.95E-05 |
| GOPC | AC022509.3 | 0.39812769 | 2.89E-07 |
| PTEN | AC022509.3 | 0.362880881 | 3.48E-06 |
| CAPN10 | GABPB1-AS1 | 0.300582316 | 0.000144585 |
| CXCR4 | GABPB1-AS1 | 0.339871906 | 1.51E-05 |
| DAPK2 | GABPB1-AS1 | 0.318171613 | 5.47E-05 |
| HDAC6 | GABPB1-AS1 | 0.357883914 | 4.83E-06 |
| MAP2K7 | GABPB1-AS1 | 0.309424357 | 8.94E-05 |
| TSC1 | GABPB1-AS1 | 0.381853169 | 9.46E-07 |
| ULK3 | GABPB1-AS1 | 0.301392951 | 0.000138438 |
| ARNT | AC005332.6 | 0.475686734 | 3.97E-10 |
| ARSB | AC005332.6 | 0.362667445 | 3.53E-06 |
| ATF6 | AC005332.6 | 0.325086794 | 3.67E-05 |
| ATG10 | AC005332.6 | 0.359720863 | 4.28E-06 |
| ATG4C | AC005332.6 | 0.368336825 | 2.41E-06 |
| ATG4D | AC005332.6 | 0.385074811 | 7.52E-07 |
| BCL2 | AC005332.6 | 0.335753511 | 1.95E-05 |
| BECN1 | AC005332.6 | 0.351861273 | 7.14E-06 |
| CALCOCO2 | AC005332.6 | 0.368410133 | 2.40E-06 |
| CASP3 | AC005332.6 | 0.426983903 | 3.02E-08 |
| CASP4 | AC005332.6 | -0.354034642 | 6.21E-06 |
| CD46 | AC005332.6 | 0.532977721 | 9.40E-13 |
| CDKN1B | AC005332.6 | 0.398909145 | 2.73E-07 |
| CX3CL1 | AC005332.6 | 0.31619851 | 6.12E-05 |
| DRAM1 | AC005332.6 | 0.310680566 | 8.34E-05 |
| EDEM1 | AC005332.6 | 0.300630134 | 0.000144215 |
| EEF2K | AC005332.6 | 0.379079173 | 1.15E-06 |
| EIF2AK3 | AC005332.6 | 0.432255002 | 1.95E-08 |
| FAS | AC005332.6 | 0.327993946 | 3.10E-05 |
| GABARAP | AC005332.6 | 0.370766493 | 2.04E-06 |
| HDAC6 | AC005332.6 | 0.5127867 | 9.01E-12 |
| IKBKB | AC005332.6 | 0.337129929 | 1.79E-05 |
| IKBKE | AC005332.6 | 0.438787205 | 1.12E-08 |
| ITGA3 | AC005332.6 | -0.32060668 | 4.76E-05 |
| ITPR1 | AC005332.6 | 0.334079626 | 2.15E-05 |
| KLHL24 | AC005332.6 | 0.47349195 | 4.90E-10 |
| MAPK8 | AC005332.6 | 0.429118123 | 2.53E-08 |
| MAPK9 | AC005332.6 | 0.485032939 | 1.60E-10 |
| MTMR14 | AC005332.6 | 0.389428809 | 5.49E-07 |
| NBR1 | AC005332.6 | 0.503057247 | 2.54E-11 |
| PARP1 | AC005332.6 | 0.543398196 | 2.76E-13 |
| PIK3C3 | AC005332.6 | 0.333471459 | 2.23E-05 |
| PIK3R4 | AC005332.6 | 0.329980399 | 2.75E-05 |
| PRKAB1 | AC005332.6 | 0.349911935 | 8.08E-06 |
| PRKAR1A | AC005332.6 | 0.452001077 | 3.55E-09 |
| PRKCD | AC005332.6 | 0.312477784 | 7.54E-05 |
| PTK6 | AC005332.6 | -0.460630026 | 1.63E-09 |
| RAB33B | AC005332.6 | 0.417191528 | 6.65E-08 |
| RAB5A | AC005332.6 | 0.326220148 | 3.44E-05 |
| RAF1 | AC005332.6 | 0.429917367 | 2.37E-08 |
| RB1CC1 | AC005332.6 | 0.306524934 | 0.000104838 |
| RHEB | AC005332.6 | 0.354617202 | 5.98E-06 |
| RPS6KB1 | AC005332.6 | 0.464958259 | 1.09E-09 |
| SIRT1 | AC005332.6 | 0.375327601 | 1.49E-06 |
| SQSTM1 | AC005332.6 | 0.506239223 | 1.82E-11 |
| TBK1 | AC005332.6 | 0.361582504 | 3.79E-06 |
| TP53 | AC005332.6 | 0.422672957 | 4.29E-08 |
| TP73 | AC005332.6 | 0.320448322 | 4.80E-05 |
| TSC1 | AC005332.6 | 0.309917873 | 8.70E-05 |
| TSC2 | AC005332.6 | 0.301422657 | 0.000138217 |
| ULK2 | AC005332.6 | 0.405054066 | 1.71E-07 |
| UVRAG | AC005332.6 | 0.382453809 | 9.06E-07 |
| VAMP7 | AC005332.6 | 0.318355862 | 5.41E-05 |
| ATF4 | DNAJC9-AS1 | 0.317892971 | 5.56E-05 |
| ATG10 | DNAJC9-AS1 | 0.331376176 | 2.53E-05 |
| ATG16L2 | DNAJC9-AS1 | 0.360018655 | 4.20E-06 |
| ATG3 | DNAJC9-AS1 | 0.352173811 | 6.99E-06 |
| ATG4B | DNAJC9-AS1 | 0.415636213 | 7.53E-08 |
| ATG4C | DNAJC9-AS1 | 0.327161925 | 3.25E-05 |
| ATIC | DNAJC9-AS1 | 0.365892946 | 2.84E-06 |
| BAX | DNAJC9-AS1 | 0.542117741 | 3.21E-13 |
| BIRC5 | DNAJC9-AS1 | 0.497235762 | 4.66E-11 |
| BIRC6 | DNAJC9-AS1 | -0.314179033 | 6.86E-05 |
| BNIP1 | DNAJC9-AS1 | 0.466011804 | 9.92E-10 |
| CAPN10 | DNAJC9-AS1 | 0.535929341 | 6.67E-13 |
| CDKN2A | DNAJC9-AS1 | 0.392445701 | 4.41E-07 |
| CLN3 | DNAJC9-AS1 | 0.326381453 | 3.40E-05 |
| EIF4G1 | DNAJC9-AS1 | -0.310642184 | 8.36E-05 |
| FKBP1A | DNAJC9-AS1 | 0.342098813 | 1.32E-05 |
| GABARAPL2 | DNAJC9-AS1 | 0.315659073 | 6.31E-05 |
| HDAC6 | DNAJC9-AS1 | 0.319463331 | 5.08E-05 |
| ITGB1 | DNAJC9-AS1 | -0.305812493 | 0.000108996 |
| LAMP2 | DNAJC9-AS1 | -0.314811322 | 6.62E-05 |
| MAP1LC3A | DNAJC9-AS1 | 0.313739412 | 7.03E-05 |
| MAPK1 | DNAJC9-AS1 | -0.340678133 | 1.44E-05 |
| MAPK8IP1 | DNAJC9-AS1 | 0.363328032 | 3.37E-06 |
| MLST8 | DNAJC9-AS1 | 0.346064888 | 1.03E-05 |
| MTMR14 | DNAJC9-AS1 | 0.396987358 | 3.15E-07 |
| NCKAP1 | DNAJC9-AS1 | -0.351316043 | 7.39E-06 |
| PEX14 | DNAJC9-AS1 | 0.326517059 | 3.38E-05 |
| RAB24 | DNAJC9-AS1 | 0.378924694 | 1.16E-06 |
| STK11 | DNAJC9-AS1 | 0.310493495 | 8.43E-05 |
| ULK3 | DNAJC9-AS1 | 0.329891759 | 2.77E-05 |
| WDFY3 | DNAJC9-AS1 | -0.339367821 | 1.56E-05 |
| WDR45 | DNAJC9-AS1 | 0.524691754 | 2.42E-12 |
| ATG16L2 | AP000894.4 | 0.304816025 | 0.000115072 |
| GABARAPL1 | AP000894.4 | 0.3333231 | 2.25E-05 |
| ITGA3 | AP000894.4 | -0.306184867 | 0.000106804 |
| TP53INP2 | AP000894.4 | 0.361615738 | 3.78E-06 |
| WIPI1 | AP000894.4 | 0.305934101 | 0.000108276 |
| PIK3R4 | MIR4435-2HG | -0.337939258 | 1.70E-05 |
| RAF1 | MIR4435-2HG | -0.336379566 | 1.87E-05 |
| RPS6KB1 | MIR4435-2HG | -0.325895438 | 3.50E-05 |
| ULK3 | MIR4435-2HG | -0.309362924 | 8.97E-05 |
| ATG10 | AC020907.1 | 0.324589777 | 3.78E-05 |
| ATG4D | AC020907.1 | 0.388387262 | 5.92E-07 |
| CAPN10 | AC020907.1 | 0.325490249 | 3.59E-05 |
| CAPNS1 | AC020907.1 | 0.350917805 | 7.58E-06 |
| CD46 | AC020907.1 | 0.332013683 | 2.44E-05 |
| CDKN1B | AC020907.1 | 0.436933324 | 1.32E-08 |
| CDKN2A | AC020907.1 | 0.411506344 | 1.04E-07 |
| CX3CL1 | AC020907.1 | 0.350427726 | 7.82E-06 |
| CXCR4 | AC020907.1 | 0.366600872 | 2.71E-06 |
| IKBKE | AC020907.1 | 0.348420747 | 8.88E-06 |
| ITGA3 | AC020907.1 | -0.315985594 | 6.20E-05 |
| MAP2K7 | AC020907.1 | 0.405626679 | 1.64E-07 |
| MAPK8 | AC020907.1 | 0.346206723 | 1.02E-05 |
| MTMR14 | AC020907.1 | 0.334171848 | 2.14E-05 |
| PINK1 | AC020907.1 | -0.366138172 | 2.80E-06 |
| PRKAB1 | AC020907.1 | 0.510846203 | 1.11E-11 |
| RAF1 | AC020907.1 | 0.361255762 | 3.87E-06 |
| TP53 | AC020907.1 | 0.412720618 | 9.47E-08 |
| TP73 | AC020907.1 | 0.304762865 | 0.000115405 |
| TSC2 | AC020907.1 | 0.392089024 | 4.52E-07 |
| ULK3 | AC020907.1 | 0.39356424 | 4.06E-07 |
| BID | LINC01426 | 0.3380319 | 1.69E-05 |
| FKBP1B | LINC01426 | 0.302411671 | 0.000131058 |
| PRKAR1A | AC132938.3 | 0.315236511 | 6.46E-05 |
| RPS6KB1 | AC132938.3 | 0.301492364 | 0.000137701 |
| ATG4D | AC090912.1 | 0.349222057 | 8.44E-06 |
| BAK1 | AC090912.1 | -0.307994267 | 9.67E-05 |
| DNAJB9 | AC090912.1 | 0.363537921 | 3.33E-06 |
| NPC1 | AC090912.1 | 0.389445672 | 5.49E-07 |
| PTK6 | AC090912.1 | -0.317391272 | 5.72E-05 |
| RHEB | AC090912.1 | 0.324998533 | 3.69E-05 |
| WIPI1 | AC090912.1 | 0.354933154 | 5.86E-06 |
| ATG16L2 | ITGB2-AS1 | 0.378313253 | 1.21E-06 |
| ATG4B | ITGB2-AS1 | 0.311592729 | 7.93E-05 |
| ATG4C | ITGB2-AS1 | 0.362399116 | 3.59E-06 |
| BAX | ITGB2-AS1 | 0.408792383 | 1.29E-07 |
| CAPN10 | ITGB2-AS1 | 0.499375344 | 3.73E-11 |
| CCR2 | ITGB2-AS1 | 0.323927873 | 3.93E-05 |
| CXCR4 | ITGB2-AS1 | 0.755954986 | 5.96E-30 |
| EIF4G1 | ITGB2-AS1 | -0.318049536 | 5.51E-05 |
| IKBKB | ITGB2-AS1 | 0.338361251 | 1.66E-05 |
| IKBKE | ITGB2-AS1 | 0.304191969 | 0.000119037 |
| ITPR1 | ITGB2-AS1 | 0.32635183 | 3.41E-05 |
| MTMR14 | ITGB2-AS1 | 0.452963637 | 3.26E-09 |
| NCKAP1 | ITGB2-AS1 | -0.334036065 | 2.16E-05 |
| RAB24 | ITGB2-AS1 | 0.471894056 | 5.71E-10 |
| RGS19 | ITGB2-AS1 | 0.375463169 | 1.48E-06 |
| STK11 | ITGB2-AS1 | 0.336964458 | 1.81E-05 |
| TP53 | ITGB2-AS1 | 0.357593972 | 4.93E-06 |
| TP73 | ITGB2-AS1 | 0.324580444 | 3.78E-05 |
| TSC2 | ITGB2-AS1 | 0.456102692 | 2.46E-09 |
| ULK3 | ITGB2-AS1 | 0.352348275 | 6.92E-06 |
| WDFY3 | ITGB2-AS1 | -0.303146317 | 0.000125966 |
| ATG5 | AL513123.1 | 0.386964728 | 6.56E-07 |
| BAG3 | AL513123.1 | 0.324046434 | 3.90E-05 |
| BNIP1 | AL513123.1 | -0.316577952 | 5.99E-05 |
| ITGA6 | AL513123.1 | 0.371800971 | 1.90E-06 |
| ATG16L2 | AC147651.3 | 0.347921449 | 9.17E-06 |
| ATG4B | AC147651.3 | 0.449648649 | 4.38E-09 |
| ATG4C | AC147651.3 | 0.314338754 | 6.80E-05 |
| BAG3 | AC147651.3 | -0.332169583 | 2.42E-05 |
| BAX | AC147651.3 | 0.397358384 | 3.06E-07 |
| BNIP1 | AC147651.3 | 0.343201848 | 1.23E-05 |
| CAPN10 | AC147651.3 | 0.473088142 | 5.10E-10 |
| CCL2 | AC147651.3 | 0.304516858 | 0.000116957 |
| CX3CL1 | AC147651.3 | 0.304097431 | 0.000119648 |
| CXCR4 | AC147651.3 | 0.673925674 | 7.27E-22 |
| DRAM1 | AC147651.3 | 0.363106721 | 3.42E-06 |
| EIF4G1 | AC147651.3 | -0.338389977 | 1.66E-05 |
| FKBP1A | AC147651.3 | 0.387454692 | 6.34E-07 |
| IFNG | AC147651.3 | 0.316712909 | 5.95E-05 |
| KIF5B | AC147651.3 | -0.313924735 | 6.96E-05 |
| MTMR14 | AC147651.3 | 0.389534802 | 5.45E-07 |
| NCKAP1 | AC147651.3 | -0.407759977 | 1.39E-07 |
| RAB24 | AC147651.3 | 0.328334627 | 3.03E-05 |
| RGS19 | AC147651.3 | 0.320186739 | 4.88E-05 |
| STK11 | AC147651.3 | 0.307848077 | 9.75E-05 |
| TSC2 | AC147651.3 | 0.362623128 | 3.54E-06 |
| WDFY3 | AC147651.3 | -0.326340905 | 3.41E-05 |
| WDR45 | AC147651.3 | 0.346496228 | 1.00E-05 |
| ATG10 | LIPE-AS1 | 0.309582839 | 8.86E-05 |
| ATG4B | LIPE-AS1 | 0.39836061 | 2.84E-07 |
| ATG7 | LIPE-AS1 | 0.401148087 | 2.31E-07 |
| BAG3 | LIPE-AS1 | -0.432768823 | 1.87E-08 |
| BAX | LIPE-AS1 | 0.441093389 | 9.22E-09 |
| BCL2 | LIPE-AS1 | 0.3946645 | 3.74E-07 |
| BIRC5 | LIPE-AS1 | 0.356272146 | 5.37E-06 |
| BIRC6 | LIPE-AS1 | -0.314109348 | 6.89E-05 |
| BNIP1 | LIPE-AS1 | 0.521691998 | 3.39E-12 |
| CANX | LIPE-AS1 | -0.306980987 | 0.000102254 |
| CAPN10 | LIPE-AS1 | 0.320158619 | 4.88E-05 |
| CAPN2 | LIPE-AS1 | -0.345063246 | 1.10E-05 |
| CAPNS1 | LIPE-AS1 | 0.319459761 | 5.08E-05 |
| CDKN2A | LIPE-AS1 | 0.411258776 | 1.06E-07 |
| CXCR4 | LIPE-AS1 | 0.323927609 | 3.93E-05 |
| EIF4G1 | LIPE-AS1 | -0.399243346 | 2.66E-07 |
| FAS | LIPE-AS1 | 0.335866122 | 1.93E-05 |
| FKBP1A | LIPE-AS1 | 0.377637864 | 1.27E-06 |
| GABARAP | LIPE-AS1 | 0.329906924 | 2.76E-05 |
| GOPC | LIPE-AS1 | -0.306600361 | 0.000104406 |
| HGS | LIPE-AS1 | -0.372086968 | 1.87E-06 |
| HSP90AB1 | LIPE-AS1 | -0.325450171 | 3.59E-05 |
| IKBKE | LIPE-AS1 | 0.305395629 | 0.000111501 |
| ITGA6 | LIPE-AS1 | -0.343005486 | 1.25E-05 |
| LAMP2 | LIPE-AS1 | -0.313931566 | 6.96E-05 |
| MAP1LC3A | LIPE-AS1 | 0.43700526 | 1.31E-08 |
| MAPK1 | LIPE-AS1 | -0.372351414 | 1.83E-06 |
| MTMR14 | LIPE-AS1 | 0.383178609 | 8.61E-07 |
| MYC | LIPE-AS1 | -0.319737839 | 5.00E-05 |
| NCKAP1 | LIPE-AS1 | -0.438999568 | 1.10E-08 |
| NKX2-3 | LIPE-AS1 | 0.454034533 | 2.96E-09 |
| PEX14 | LIPE-AS1 | 0.340794165 | 1.43E-05 |
| PRKAB1 | LIPE-AS1 | 0.300212044 | 0.000147476 |
| RAB1A | LIPE-AS1 | -0.366423215 | 2.74E-06 |
| RAB24 | LIPE-AS1 | 0.378452145 | 1.20E-06 |
| RAC1 | LIPE-AS1 | 0.303271226 | 0.000125119 |
| SPNS1 | LIPE-AS1 | -0.329785808 | 2.78E-05 |
| TP63 | LIPE-AS1 | -0.327207873 | 3.24E-05 |
| TP73 | LIPE-AS1 | 0.316821154 | 5.91E-05 |
| TSC2 | LIPE-AS1 | 0.387393981 | 6.36E-07 |
| USP10 | LIPE-AS1 | -0.41799444 | 6.24E-08 |
| WDFY3 | LIPE-AS1 | -0.367847372 | 2.49E-06 |
| WDR45 | LIPE-AS1 | 0.458979911 | 1.90E-09 |
| ATG4A | AC068870.2 | 0.301399312 | 0.000138391 |
| RAB5A | AC068870.2 | 0.410046257 | 1.17E-07 |
| RAF1 | AC068870.2 | 0.344354669 | 1.15E-05 |
| BIRC6 | AC009271.1 | 0.334719264 | 2.07E-05 |
| KLHL24 | AC009271.1 | 0.386994575 | 6.55E-07 |
| MBTPS2 | AC009271.1 | 0.303753039 | 0.000121901 |
| ST13 | AC009271.1 | 0.313229864 | 7.23E-05 |
| TP63 | AC009271.1 | 0.328228846 | 3.05E-05 |
| VAMP7 | AC009271.1 | 0.338166373 | 1.68E-05 |
| EIF2AK3 | TMEM44-AS1 | 0.324629709 | 3.77E-05 |
| GAA | TMEM44-AS1 | 0.335510579 | 1.98E-05 |
| KLHL24 | TMEM44-AS1 | 0.460085155 | 1.71E-09 |
| NFE2L2 | TMEM44-AS1 | 0.326269789 | 3.43E-05 |
| PINK1 | TMEM44-AS1 | -0.300696919 | 0.000143701 |
| TP63 | TMEM44-AS1 | 0.322438137 | 4.28E-05 |
| VAMP7 | TMEM44-AS1 | 0.378750001 | 1.18E-06 |
| WIPI1 | TMEM44-AS1 | 0.398816988 | 2.75E-07 |
| ATG4B | AL391834.1 | 0.338192043 | 1.68E-05 |
| BAX | AL391834.1 | 0.439144901 | 1.09E-08 |
| CAPN10 | AL391834.1 | 0.387467416 | 6.33E-07 |
| CAPN2 | AL391834.1 | -0.304679366 | 0.00011593 |
| CDKN1B | AL391834.1 | 0.327064905 | 3.27E-05 |
| CXCR4 | AL391834.1 | 0.517206186 | 5.57E-12 |
| MAP2K7 | AL391834.1 | 0.307773892 | 9.79E-05 |
| PELP1 | AL391834.1 | 0.314431709 | 6.76E-05 |
| TP53 | AL391834.1 | 0.310312166 | 8.51E-05 |
| ARNT | AC009506.1 | 0.331371318 | 2.53E-05 |
| BAK1 | AC009506.1 | -0.496145998 | 5.21E-11 |
| BID | AC009506.1 | 0.302344091 | 0.000131536 |
| BIRC6 | AC009506.1 | 0.310177681 | 8.57E-05 |
| CASP4 | AC009506.1 | -0.317173385 | 5.79E-05 |
| CD46 | AC009506.1 | 0.400900923 | 2.35E-07 |
| CDKN1B | AC009506.1 | 0.398971307 | 2.72E-07 |
| EIF2AK3 | AC009506.1 | 0.475196158 | 4.17E-10 |
| GABARAPL1 | AC009506.1 | 0.347021648 | 9.70E-06 |
| GNAI3 | AC009506.1 | -0.301258612 | 0.000139439 |
| HDAC6 | AC009506.1 | 0.489646223 | 1.01E-10 |
| ITGA3 | AC009506.1 | -0.519337832 | 4.40E-12 |
| ITGA6 | AC009506.1 | -0.312984583 | 7.33E-05 |
| KLHL24 | AC009506.1 | 0.403122445 | 1.99E-07 |
| NBR1 | AC009506.1 | 0.340073772 | 1.50E-05 |
| NFE2L2 | AC009506.1 | 0.450490281 | 4.06E-09 |
| PARP1 | AC009506.1 | 0.381878083 | 9.44E-07 |
| PINK1 | AC009506.1 | -0.312190767 | 7.67E-05 |
| PRKAB1 | AC009506.1 | 0.366847218 | 2.67E-06 |
| PRKAR1A | AC009506.1 | 0.34769845 | 9.30E-06 |
| PTK6 | AC009506.1 | -0.340337677 | 1.47E-05 |
| RHEB | AC009506.1 | 0.382781506 | 8.85E-07 |
| SIRT1 | AC009506.1 | 0.330774092 | 2.63E-05 |
| ST13 | AC009506.1 | 0.41310684 | 9.19E-08 |
| ULK3 | AC009506.1 | 0.364079379 | 3.21E-06 |
| UVRAG | AC009506.1 | 0.306638239 | 0.00010419 |
| WIPI1 | AC009506.1 | 0.374049845 | 1.63E-06 |
| AMBRA1 | AC008764.2 | 0.381342317 | 9.80E-07 |
| ARNT | AC008764.2 | 0.405128455 | 1.70E-07 |
| ARSB | AC008764.2 | 0.434752743 | 1.58E-08 |
| ATG2A | AC008764.2 | 0.320519518 | 4.78E-05 |
| ATG4C | AC008764.2 | 0.336734588 | 1.83E-05 |
| BCL2 | AC008764.2 | 0.451104923 | 3.85E-09 |
| BECN1 | AC008764.2 | 0.301618149 | 0.000136774 |
| BIRC6 | AC008764.2 | 0.418318326 | 6.08E-08 |
| CAMKK2 | AC008764.2 | 0.374772546 | 1.55E-06 |
| CASP8 | AC008764.2 | 0.324862577 | 3.72E-05 |
| CCR2 | AC008764.2 | 0.401945286 | 2.17E-07 |
| CD46 | AC008764.2 | 0.311647523 | 7.90E-05 |
| CDKN1B | AC008764.2 | 0.364741278 | 3.07E-06 |
| CFLAR | AC008764.2 | 0.341607336 | 1.36E-05 |
| CX3CL1 | AC008764.2 | 0.307110737 | 0.00010153 |
| CXCR4 | AC008764.2 | 0.480304492 | 2.54E-10 |
| DRAM1 | AC008764.2 | 0.338749727 | 1.62E-05 |
| EEF2 | AC008764.2 | 0.303738506 | 0.000121997 |
| EEF2K | AC008764.2 | 0.479352655 | 2.79E-10 |
| EIF2AK3 | AC008764.2 | 0.450550344 | 4.04E-09 |
| EIF4EBP1 | AC008764.2 | -0.340634243 | 1.44E-05 |
| FAS | AC008764.2 | 0.310504346 | 8.42E-05 |
| HDAC6 | AC008764.2 | 0.444837934 | 6.67E-09 |
| IKBKB | AC008764.2 | 0.382091007 | 9.30E-07 |
| IKBKE | AC008764.2 | 0.350008178 | 8.03E-06 |
| ITPR1 | AC008764.2 | 0.36945042 | 2.24E-06 |
| KLHL24 | AC008764.2 | 0.366300632 | 2.77E-06 |
| MAP2K7 | AC008764.2 | 0.419576156 | 5.50E-08 |
| MAPK1 | AC008764.2 | 0.303930638 | 0.000120734 |
| MAPK8 | AC008764.2 | 0.386717175 | 6.68E-07 |
| MTOR | AC008764.2 | 0.390829944 | 4.96E-07 |
| NBR1 | AC008764.2 | 0.433688544 | 1.73E-08 |
| NKX2-3 | AC008764.2 | 0.373440668 | 1.70E-06 |
| PARP1 | AC008764.2 | 0.365660642 | 2.89E-06 |
| PIK3R4 | AC008764.2 | 0.435689227 | 1.46E-08 |
| PRKAB1 | AC008764.2 | 0.352173744 | 6.99E-06 |
| RAF1 | AC008764.2 | 0.405162693 | 1.70E-07 |
| SIRT1 | AC008764.2 | 0.410637263 | 1.11E-07 |
| TP53 | AC008764.2 | 0.429195239 | 2.51E-08 |
| TP73 | AC008764.2 | 0.479540649 | 2.74E-10 |
| TSC1 | AC008764.2 | 0.498953225 | 3.90E-11 |
| TSC2 | AC008764.2 | 0.538783559 | 4.77E-13 |
| ULK2 | AC008764.2 | 0.386406746 | 6.83E-07 |
| UVRAG | AC008764.2 | 0.388381389 | 5.93E-07 |
| ATG10 | AC026740.1 | 0.357134185 | 5.08E-06 |
| ATG2B | AC026740.1 | -0.33189565 | 2.46E-05 |
| ATG4B | AC026740.1 | 0.434504107 | 1.61E-08 |
| ATG7 | AC026740.1 | 0.302632325 | 0.000129509 |
| BAX | AC026740.1 | 0.311211952 | 8.10E-05 |
| BNIP1 | AC026740.1 | 0.40045015 | 2.43E-07 |
| CAPN10 | AC026740.1 | 0.391998888 | 4.55E-07 |
| CAPNS1 | AC026740.1 | 0.408151497 | 1.35E-07 |
| CDKN2A | AC026740.1 | 0.391196553 | 4.83E-07 |
| FKBP1A | AC026740.1 | 0.31190388 | 7.79E-05 |
| MAP1LC3A | AC026740.1 | 0.412763812 | 9.44E-08 |
| MAPK1 | AC026740.1 | -0.316069989 | 6.17E-05 |
| MTMR14 | AC026740.1 | 0.391436682 | 4.75E-07 |
| PEX14 | AC026740.1 | 0.313001349 | 7.33E-05 |
| RAC1 | AC026740.1 | 0.399046602 | 2.70E-07 |
| STK11 | AC026740.1 | 0.387149878 | 6.48E-07 |
| WDR45 | AC026740.1 | 0.300432172 | 0.000145751 |
| FKBP1A | AL513318.2 | 0.368644615 | 2.36E-06 |
| BIRC6 | AC099066.2 | -0.331439547 | 2.52E-05 |
| EIF2AK3 | AC099066.2 | -0.327040443 | 3.27E-05 |
| FOXO3 | AC099066.2 | -0.30502427 | 0.000113777 |
| KIF5B | AC099066.2 | -0.339070167 | 1.59E-05 |
| MTMR14 | AC099066.2 | -0.322608613 | 4.24E-05 |
| PINK1 | AC099066.2 | 0.376081675 | 1.42E-06 |
| RAF1 | AC099066.2 | -0.363794652 | 3.27E-06 |
| RPS6KB1 | AC099066.2 | -0.357232962 | 5.04E-06 |
| ULK3 | AC099066.2 | -0.321755733 | 4.46E-05 |
| ATF4 | AC005261.3 | 0.373748208 | 1.67E-06 |
| ATG16L2 | AC005261.3 | 0.384307858 | 7.94E-07 |
| ATG2B | AC005261.3 | -0.323246826 | 4.09E-05 |
| ATG4B | AC005261.3 | 0.504480987 | 2.19E-11 |
| ATG4D | AC005261.3 | 0.357699264 | 4.89E-06 |
| BAX | AC005261.3 | 0.459372995 | 1.83E-09 |
| BID | AC005261.3 | 0.353728593 | 6.33E-06 |
| BIRC6 | AC005261.3 | -0.39275403 | 4.31E-07 |
| BNIP1 | AC005261.3 | 0.371427948 | 1.95E-06 |
| CANX | AC005261.3 | -0.326346096 | 3.41E-05 |
| CAPN10 | AC005261.3 | 0.486381725 | 1.40E-10 |
| DAPK2 | AC005261.3 | 0.443389634 | 7.57E-09 |
| EIF4G1 | AC005261.3 | -0.429813223 | 2.39E-08 |
| GABARAP | AC005261.3 | 0.3429671 | 1.25E-05 |
| HSPA8 | AC005261.3 | -0.327771555 | 3.14E-05 |
| KIF5B | AC005261.3 | -0.423993398 | 3.85E-08 |
| MAPK1 | AC005261.3 | -0.345687887 | 1.06E-05 |
| MBTPS2 | AC005261.3 | -0.305066554 | 0.000113515 |
| NCKAP1 | AC005261.3 | -0.354097048 | 6.18E-06 |
| NPC1 | AC005261.3 | -0.30434898 | 0.000118027 |
| PEX14 | AC005261.3 | 0.397373944 | 3.06E-07 |
| RAB24 | AC005261.3 | 0.398723007 | 2.77E-07 |
| SPNS1 | AC005261.3 | -0.310325266 | 8.50E-05 |
| STK11 | AC005261.3 | 0.378567746 | 1.19E-06 |
| USP10 | AC005261.3 | -0.404337998 | 1.81E-07 |
| WDFY3 | AC005261.3 | -0.372291623 | 1.84E-06 |
| WDR45 | AC005261.3 | 0.469542078 | 7.13E-10 |
| AMBRA1 | AL451165.2 | -0.337364398 | 1.77E-05 |
| ARNT | AL451165.2 | -0.312227029 | 7.65E-05 |
| ATG16L2 | AL451165.2 | 0.320731204 | 4.73E-05 |
| ATG2A | AL451165.2 | -0.332406144 | 2.38E-05 |
| ATG4B | AL451165.2 | 0.37610227 | 1.42E-06 |
| BAX | AL451165.2 | 0.387971955 | 6.10E-07 |
| BID | AL451165.2 | 0.383836543 | 8.21E-07 |
| BNIP1 | AL451165.2 | 0.344707181 | 1.12E-05 |
| CAMKK2 | AL451165.2 | -0.336112915 | 1.90E-05 |
| CANX | AL451165.2 | -0.318075162 | 5.50E-05 |
| CAPN10 | AL451165.2 | 0.349254233 | 8.43E-06 |
| CLN3 | AL451165.2 | 0.372056553 | 1.87E-06 |
| GABARAPL2 | AL451165.2 | 0.43424555 | 1.65E-08 |
| GOPC | AL451165.2 | -0.322386247 | 4.30E-05 |
| KIF5B | AL451165.2 | -0.348381206 | 8.91E-06 |
| MAPK1 | AL451165.2 | -0.370544964 | 2.08E-06 |
| NAF1 | AL451165.2 | -0.311488725 | 7.97E-05 |
| NFKB1 | AL451165.2 | -0.351055648 | 7.51E-06 |
| WDR45 | AL451165.2 | 0.352811828 | 6.71E-06 |
| BAK1 | AC026801.2 | -0.308898486 | 9.20E-05 |
| BID | AC026801.2 | 0.317567923 | 5.66E-05 |
| HDAC6 | AC026801.2 | 0.34908836 | 8.52E-06 |
| ITGA3 | AC026801.2 | -0.333949655 | 2.17E-05 |
| KLHL24 | AC026801.2 | 0.314853366 | 6.60E-05 |
| PRKAB1 | AC026801.2 | 0.31888328 | 5.25E-05 |
| WDR45 | AC026801.2 | 0.338964606 | 1.60E-05 |
| BAX | AL606834.1 | 0.347984733 | 9.13E-06 |
| PINK1 | AL606834.1 | -0.303791647 | 0.000121646 |
| ARSB | AC104031.1 | -0.310580052 | 8.39E-05 |
| CD46 | AC104031.1 | -0.307956346 | 9.69E-05 |
| DDIT3 | AC104031.1 | 0.487229965 | 1.28E-10 |
| MAPK8 | AC104031.1 | -0.302114661 | 0.000133171 |
| MAPK9 | AC104031.1 | -0.332164716 | 2.42E-05 |
| PARP1 | AC104031.1 | -0.318927213 | 5.24E-05 |
| PRKCD | AC104031.1 | -0.317374925 | 5.73E-05 |
| RAB5A | AC104031.1 | -0.31052755 | 8.41E-05 |
| RAF1 | AC104031.1 | -0.343014646 | 1.25E-05 |
| SIRT1 | AC104031.1 | -0.301846209 | 0.000135108 |
| APOL1 | MIR9-3HG | 0.328089913 | 3.08E-05 |
| ATG10 | MIR9-3HG | 0.465713071 | 1.02E-09 |
| ATG2A | MIR9-3HG | 0.302828674 | 0.000128145 |
| ATG3 | MIR9-3HG | 0.549386418 | 1.34E-13 |
| ATG4A | MIR9-3HG | 0.344103841 | 1.17E-05 |
| ATG4B | MIR9-3HG | 0.300672095 | 0.000143892 |
| ATG4C | MIR9-3HG | 0.451033017 | 3.87E-09 |
| ATG4D | MIR9-3HG | 0.391972813 | 4.56E-07 |
| ATG7 | MIR9-3HG | 0.467570637 | 8.58E-10 |
| BCL2 | MIR9-3HG | 0.353617255 | 6.37E-06 |
| CAPNS1 | MIR9-3HG | 0.383395692 | 8.48E-07 |
| CASP3 | MIR9-3HG | 0.389430646 | 5.49E-07 |
| CD46 | MIR9-3HG | 0.356552751 | 5.27E-06 |
| CDKN1B | MIR9-3HG | 0.364157758 | 3.19E-06 |
| CDKN2A | MIR9-3HG | 0.551374903 | 1.05E-13 |
| CFLAR | MIR9-3HG | 0.335890925 | 1.93E-05 |
| EEF2K | MIR9-3HG | 0.394635102 | 3.75E-07 |
| FAS | MIR9-3HG | 0.345093715 | 1.10E-05 |
| HDAC1 | MIR9-3HG | 0.354543072 | 6.01E-06 |
| HDAC6 | MIR9-3HG | 0.577803067 | 3.50E-15 |
| HSP90AB1 | MIR9-3HG | -0.333905329 | 2.18E-05 |
| IKBKE | MIR9-3HG | 0.540408053 | 3.94E-13 |
| MAP2K7 | MIR9-3HG | 0.403701084 | 1.90E-07 |
| MAPK8 | MIR9-3HG | 0.465307769 | 1.06E-09 |
| MTMR14 | MIR9-3HG | 0.467090494 | 8.97E-10 |
| NKX2-3 | MIR9-3HG | 0.505131389 | 2.04E-11 |
| PARP1 | MIR9-3HG | 0.358274153 | 4.71E-06 |
| PEX14 | MIR9-3HG | 0.32999052 | 2.75E-05 |
| PIK3R4 | MIR9-3HG | 0.465482593 | 1.04E-09 |
| PINK1 | MIR9-3HG | -0.30336981 | 0.000124454 |
| PRKAB1 | MIR9-3HG | 0.451252982 | 3.80E-09 |
| RAB33B | MIR9-3HG | 0.372369194 | 1.83E-06 |
| RAF1 | MIR9-3HG | 0.537569505 | 5.50E-13 |
| STK11 | MIR9-3HG | 0.406859634 | 1.49E-07 |
| TP53 | MIR9-3HG | 0.443245125 | 7.66E-09 |
| TP73 | MIR9-3HG | 0.55710171 | 5.15E-14 |
| TSC1 | MIR9-3HG | 0.346344913 | 1.01E-05 |
| TSC2 | MIR9-3HG | 0.488819152 | 1.10E-10 |
| ULK3 | MIR9-3HG | 0.424112184 | 3.81E-08 |
| WDR45 | MIR9-3HG | 0.370463156 | 2.09E-06 |
| ATG4C | AC100861.1 | 0.301595483 | 0.000136941 |
| ATG7 | AC100861.1 | 0.316911408 | 5.88E-05 |
| BCL2 | AC100861.1 | 0.448036029 | 5.04E-09 |
| BNIP3L | AC100861.1 | 0.373448354 | 1.70E-06 |
| CAMKK2 | AC100861.1 | 0.394260096 | 3.86E-07 |
| CDKN1B | AC100861.1 | 0.383716935 | 8.28E-07 |
| CXCR4 | AC100861.1 | 0.5074712 | 1.60E-11 |
| DRAM1 | AC100861.1 | 0.374561322 | 1.58E-06 |
| EEF2K | AC100861.1 | 0.36313838 | 3.42E-06 |
| FAS | AC100861.1 | 0.472238539 | 5.52E-10 |
| HDAC6 | AC100861.1 | 0.363618395 | 3.31E-06 |
| IKBKB | AC100861.1 | 0.358401254 | 4.67E-06 |
| MAP2K7 | AC100861.1 | 0.349652505 | 8.22E-06 |
| MAPK8 | AC100861.1 | 0.441633798 | 8.80E-09 |
| MAPK9 | AC100861.1 | 0.331098773 | 2.58E-05 |
| MTMR14 | AC100861.1 | 0.361793768 | 3.74E-06 |
| NAF1 | AC100861.1 | 0.329726203 | 2.79E-05 |
| NKX2-3 | AC100861.1 | 0.460717936 | 1.62E-09 |
| PARP1 | AC100861.1 | 0.481730132 | 2.21E-10 |
| PIK3R4 | AC100861.1 | 0.311455257 | 7.99E-05 |
| PINK1 | AC100861.1 | -0.360565972 | 4.05E-06 |
| PRKAB1 | AC100861.1 | 0.417508734 | 6.49E-08 |
| RAB33B | AC100861.1 | 0.398097875 | 2.90E-07 |
| RAF1 | AC100861.1 | 0.440712371 | 9.53E-09 |
| SIRT1 | AC100861.1 | 0.425643703 | 3.37E-08 |
| SQSTM1 | AC100861.1 | 0.322838883 | 4.18E-05 |
| STK11 | AC100861.1 | 0.343328298 | 1.22E-05 |
| TP53 | AC100861.1 | 0.510218252 | 1.19E-11 |
| TP73 | AC100861.1 | 0.360598229 | 4.04E-06 |
| TSC2 | AC100861.1 | 0.460118826 | 1.71E-09 |
| UVRAG | AC100861.1 | 0.31393354 | 6.95E-05 |
| ARNT | AL354836.1 | -0.308021634 | 9.66E-05 |
| ARSB | AL354836.1 | -0.322470662 | 4.28E-05 |
| ATF4 | AL354836.1 | 0.336484695 | 1.86E-05 |
| ATG16L2 | AL354836.1 | 0.469624876 | 7.07E-10 |
| ATG4B | AL354836.1 | 0.616271504 | 1.39E-17 |
| BAX | AL354836.1 | 0.324204192 | 3.87E-05 |
| BIRC6 | AL354836.1 | -0.365655927 | 2.89E-06 |
| BNIP1 | AL354836.1 | 0.352656513 | 6.78E-06 |
| CANX | AL354836.1 | -0.306496103 | 0.000105003 |
| CAPN10 | AL354836.1 | 0.532127335 | 1.04E-12 |
| CLN3 | AL354836.1 | 0.328549187 | 3.00E-05 |
| DAPK2 | AL354836.1 | 0.335938993 | 1.93E-05 |
| DLC1 | AL354836.1 | -0.300479755 | 0.00014538 |
| KIF5B | AL354836.1 | -0.362609571 | 3.54E-06 |
| MAPK1 | AL354836.1 | -0.308887397 | 9.21E-05 |
| MBTPS2 | AL354836.1 | -0.390771143 | 4.98E-07 |
| MLST8 | AL354836.1 | 0.344312643 | 1.15E-05 |
| NCKAP1 | AL354836.1 | -0.349328389 | 8.39E-06 |
| PELP1 | AL354836.1 | 0.353483864 | 6.43E-06 |
| PEX14 | AL354836.1 | 0.355235142 | 5.74E-06 |
| RAB24 | AL354836.1 | 0.309119744 | 9.09E-05 |
| STK11 | AL354836.1 | 0.503491608 | 2.43E-11 |
| WDR45 | AL354836.1 | 0.356010003 | 5.46E-06 |
| ATF6 | U91328.1 | 0.305915575 | 0.000108385 |
| BIRC6 | U91328.1 | 0.376249016 | 1.40E-06 |
| EEF2K | U91328.1 | 0.330672448 | 2.64E-05 |
| EIF2AK3 | U91328.1 | 0.359781969 | 4.27E-06 |
| EIF4G1 | U91328.1 | 0.321316649 | 4.57E-05 |
| GABARAPL1 | U91328.1 | 0.305349479 | 0.000111781 |
| ITGA3 | U91328.1 | -0.357197805 | 5.05E-06 |
| KLHL24 | U91328.1 | 0.318495346 | 5.37E-05 |
| NFE2L2 | U91328.1 | 0.378301181 | 1.21E-06 |
| PARP1 | U91328.1 | 0.317268125 | 5.76E-05 |
| PIK3R4 | U91328.1 | 0.37091082 | 2.02E-06 |
| RB1CC1 | U91328.1 | 0.308926787 | 9.19E-05 |
| ST13 | U91328.1 | 0.326839845 | 3.31E-05 |
| ATIC | AC064836.3 | 0.304240885 | 0.000118721 |
| GABARAPL2 | AC064836.3 | 0.31957758 | 5.05E-05 |
| ITGA3 | AC064836.3 | -0.317123828 | 5.81E-05 |
| NFKB1 | AC064836.3 | -0.304942353 | 0.000114285 |
| EIF2AK3 | LINC00460 | -0.335048856 | 2.03E-05 |
| FADD | LINC00460 | 0.434323135 | 1.64E-08 |
| GOPC | LINC00460 | 0.303455655 | 0.000123878 |
| IL24 | LINC00460 | 0.487655527 | 1.23E-10 |
| ITGA3 | LINC00460 | 0.351126249 | 7.48E-06 |
| MAP2K7 | LINC00460 | -0.302376583 | 0.000131306 |
| NFE2L2 | LINC00460 | -0.326236261 | 3.43E-05 |
| PRKAB1 | LINC00460 | -0.304208592 | 0.000118929 |
| ULK3 | LINC00460 | -0.304608531 | 0.000116376 |
| MAP1LC3B | LINC01807 | 0.323599976 | 4.00E-05 |
| RAB1A | LINC01807 | 0.420673851 | 5.04E-08 |
| FKBP1B | H19 | 0.304739979 | 0.000115548 |
| FOXO1 | H19 | -0.310077634 | 8.62E-05 |
| ITGB1 | H19 | 0.377736661 | 1.26E-06 |
| MYC | H19 | 0.356691006 | 5.22E-06 |
| NAF1 | H19 | 0.346165321 | 1.02E-05 |
| NFKB1 | H19 | 0.333221011 | 2.27E-05 |
| ATG3 | AL035258.1 | 0.355545369 | 5.63E-06 |
| ATG4B | AL035258.1 | 0.319185027 | 5.16E-05 |
| ATG4D | AL035258.1 | 0.423650665 | 3.96E-08 |
| ATG7 | AL035258.1 | 0.315227856 | 6.47E-05 |
| CAPNS1 | AL035258.1 | 0.32993406 | 2.76E-05 |
| CDKN2A | AL035258.1 | 0.499980388 | 3.51E-11 |
| ERBB2 | AL035258.1 | 0.307184102 | 0.000101122 |
| IKBKE | AL035258.1 | 0.397685752 | 2.99E-07 |
| MAP2K7 | AL035258.1 | 0.386704445 | 6.69E-07 |
| MTMR14 | AL035258.1 | 0.322306168 | 4.32E-05 |
| PINK1 | AL035258.1 | -0.374270327 | 1.61E-06 |
| PRKAB1 | AL035258.1 | 0.341543498 | 1.37E-05 |
| PTK6 | AL035258.1 | -0.303250642 | 0.000125259 |
| STK11 | AL035258.1 | 0.324415217 | 3.82E-05 |
| TP53 | AL035258.1 | 0.307440952 | 9.97E-05 |
| WDR45 | AL035258.1 | 0.312099637 | 7.71E-05 |
| ATG16L2 | Z69706.1 | 0.376065578 | 1.42E-06 |
| ATG4B | Z69706.1 | 0.441492991 | 8.91E-09 |
| CAPN10 | Z69706.1 | 0.432217612 | 1.96E-08 |
| CXCR4 | Z69706.1 | 0.607695613 | 5.10E-17 |
| DAPK2 | Z69706.1 | 0.35494346 | 5.85E-06 |
| IKBKB | Z69706.1 | 0.313982847 | 6.94E-05 |
| MTMR14 | Z69706.1 | 0.313850713 | 6.99E-05 |
| STK11 | Z69706.1 | 0.335696538 | 1.95E-05 |
| TP53 | Z69706.1 | 0.367905211 | 2.48E-06 |
| TP73 | Z69706.1 | 0.311185467 | 8.11E-05 |
| TSC2 | Z69706.1 | 0.494097832 | 6.43E-11 |
| ULK3 | Z69706.1 | 0.312998332 | 7.33E-05 |
| ATF4 | AL035461.2 | 0.320659003 | 4.75E-05 |
| ATG4B | AL035461.2 | 0.514087214 | 7.83E-12 |
| BAG1 | AL035461.2 | 0.341972719 | 1.33E-05 |
| BAG3 | AL035461.2 | -0.322916633 | 4.17E-05 |
| BAX | AL035461.2 | 0.60368751 | 9.22E-17 |
| BIRC5 | AL035461.2 | 0.504330559 | 2.22E-11 |
| BNIP1 | AL035461.2 | 0.469517513 | 7.15E-10 |
| CAPN10 | AL035461.2 | 0.488556803 | 1.12E-10 |
| CDKN2A | AL035461.2 | 0.368963322 | 2.31E-06 |
| CHMP4B | AL035461.2 | 0.309256802 | 9.02E-05 |
| CLN3 | AL035461.2 | 0.308591542 | 9.36E-05 |
| CXCR4 | AL035461.2 | 0.532688879 | 9.72E-13 |
| EIF4G1 | AL035461.2 | -0.35094809 | 7.56E-06 |
| FKBP1A | AL035461.2 | 0.516755405 | 5.85E-12 |
| GABARAP | AL035461.2 | 0.316936244 | 5.87E-05 |
| HIF1A | AL035461.2 | -0.359796178 | 4.26E-06 |
| KIF5B | AL035461.2 | -0.314265648 | 6.83E-05 |
| MAP2K7 | AL035461.2 | 0.319037059 | 5.21E-05 |
| MAPK1 | AL035461.2 | -0.324011462 | 3.91E-05 |
| MLST8 | AL035461.2 | 0.338483293 | 1.65E-05 |
| MTMR14 | AL035461.2 | 0.440574222 | 9.64E-09 |
| NCKAP1 | AL035461.2 | -0.3998808 | 2.54E-07 |
| PELP1 | AL035461.2 | 0.300772892 | 0.000143117 |
| PEX14 | AL035461.2 | 0.303630886 | 0.00012271 |
| RAB24 | AL035461.2 | 0.371903198 | 1.89E-06 |
| STK11 | AL035461.2 | 0.384181359 | 8.01E-07 |
| TP53 | AL035461.2 | 0.300168787 | 0.000147818 |
| TSC2 | AL035461.2 | 0.352292251 | 6.94E-06 |
| USP10 | AL035461.2 | -0.3590826 | 4.47E-06 |
| WDFY3 | AL035461.2 | -0.34564486 | 1.06E-05 |
| WDR45 | AL035461.2 | 0.579612729 | 2.75E-15 |
| ARNT | AP003392.4 | 0.318072316 | 5.50E-05 |
| ARSB | AP003392.4 | 0.301218722 | 0.000139738 |
| BAK1 | AP003392.4 | -0.309011452 | 9.15E-05 |
| BIRC6 | AP003392.4 | 0.389080754 | 5.63E-07 |
| DAPK1 | AP003392.4 | 0.411243332 | 1.06E-07 |
| EIF2AK3 | AP003392.4 | 0.390902084 | 4.93E-07 |
| EIF4G1 | AP003392.4 | 0.320845361 | 4.70E-05 |
| KLHL24 | AP003392.4 | 0.382226602 | 9.21E-07 |
| MBTPS2 | AP003392.4 | 0.311154722 | 8.12E-05 |
| NBR1 | AP003392.4 | 0.323978381 | 3.92E-05 |
| NFE2L2 | AP003392.4 | 0.309707349 | 8.80E-05 |
| PARP1 | AP003392.4 | 0.36911365 | 2.29E-06 |
| RAF1 | AP003392.4 | 0.303105028 | 0.000126248 |
| SIRT1 | AP003392.4 | 0.327923045 | 3.11E-05 |
| UVRAG | AP003392.4 | 0.402298816 | 2.11E-07 |
| ATF4 | LINC00623 | 0.30144561 | 0.000138047 |
| ATG4A | LINC00623 | 0.367366116 | 2.57E-06 |
| BAX | LINC00623 | 0.371811302 | 1.90E-06 |
| BIRC5 | LINC00623 | 0.347851259 | 9.21E-06 |
| BNIP1 | LINC00623 | 0.350488202 | 7.79E-06 |
| MAPK1 | LINC00623 | -0.34506511 | 1.10E-05 |
| NCKAP1 | LINC00623 | -0.336029857 | 1.91E-05 |
| PEX14 | LINC00623 | 0.314137197 | 6.88E-05 |
| PRKCQ | LINC00623 | 0.376552534 | 1.37E-06 |
| RAB24 | LINC00623 | 0.306015874 | 0.000107794 |
| WDFY3 | LINC00623 | -0.300281461 | 0.00014693 |
| WDR45 | LINC00623 | 0.33291019 | 2.31E-05 |
| APOL1 | AC007991.2 | 0.365635651 | 2.89E-06 |
| ATG3 | AC007991.2 | 0.342930511 | 1.25E-05 |
| ATG4A | AC007991.2 | 0.352839356 | 6.70E-06 |
| CDKN2A | AC007991.2 | 0.377713025 | 1.27E-06 |
| CX3CL1 | AC007991.2 | 0.465274515 | 1.06E-09 |
| HDAC6 | AC007991.2 | 0.440089428 | 1.01E-08 |
| IKBKB | AC007991.2 | 0.373878551 | 1.65E-06 |
| RAF1 | AC007991.2 | 0.307572265 | 9.90E-05 |
| SESN2 | AC007991.2 | 0.327515787 | 3.18E-05 |
| STK11 | AC007991.2 | 0.367343332 | 2.58E-06 |
| TNFSF10 | AC007991.2 | 0.407274006 | 1.45E-07 |
| SIRT2 | BX537318.1 | 0.302300418 | 0.000131846 |
| ATF4 | SNHG1 | 0.437537091 | 1.25E-08 |
| ATG12 | SNHG1 | 0.342628211 | 1.28E-05 |
| ATG16L2 | SNHG1 | 0.466757083 | 9.26E-10 |
| ATG4B | SNHG1 | 0.398222979 | 2.87E-07 |
| ATG4C | SNHG1 | 0.300781494 | 0.000143051 |
| ATG4D | SNHG1 | 0.34202112 | 1.33E-05 |
| BAX | SNHG1 | 0.303654527 | 0.000122553 |
| BIRC5 | SNHG1 | 0.318933352 | 5.24E-05 |
| BNIP1 | SNHG1 | 0.321343252 | 4.56E-05 |
| CAPN10 | SNHG1 | 0.561361632 | 3.01E-14 |
| CXCR4 | SNHG1 | 0.388442944 | 5.90E-07 |
| IKBKB | SNHG1 | 0.319866437 | 4.97E-05 |
| MAP2K7 | SNHG1 | 0.358399702 | 4.67E-06 |
| MAPK3 | SNHG1 | -0.308327776 | 9.50E-05 |
| PINK1 | SNHG1 | -0.384554461 | 7.80E-07 |
| STK11 | SNHG1 | 0.375664998 | 1.46E-06 |
| ULK3 | SNHG1 | 0.33445418 | 2.11E-05 |
| WDFY3 | SNHG1 | -0.318000197 | 5.53E-05 |
| WDR45 | SNHG1 | 0.397180768 | 3.11E-07 |
| ZFYVE1 | SNHG1 | -0.308475485 | 9.42E-05 |
| ATG10 | ASB16-AS1 | 0.397864073 | 2.95E-07 |
| ATG12 | ASB16-AS1 | 0.36311709 | 3.42E-06 |
| ATG16L2 | ASB16-AS1 | 0.306947955 | 0.000102439 |
| ATG3 | ASB16-AS1 | 0.453307124 | 3.16E-09 |
| ATG4A | ASB16-AS1 | 0.352956054 | 6.65E-06 |
| ATG4B | ASB16-AS1 | 0.381924149 | 9.41E-07 |
| ATG4C | ASB16-AS1 | 0.445029794 | 6.56E-09 |
| ATG4D | ASB16-AS1 | 0.371986975 | 1.88E-06 |
| ATG7 | ASB16-AS1 | 0.314612698 | 6.69E-05 |
| BAX | ASB16-AS1 | 0.353053535 | 6.61E-06 |
| BCL2 | ASB16-AS1 | 0.325525906 | 3.58E-05 |
| BECN1 | ASB16-AS1 | 0.309044437 | 9.13E-05 |
| CALCOCO2 | ASB16-AS1 | 0.347949879 | 9.15E-06 |
| CAPN10 | ASB16-AS1 | 0.445731665 | 6.17E-09 |
| CD46 | ASB16-AS1 | 0.300233292 | 0.000147309 |
| CDKN1B | ASB16-AS1 | 0.467991604 | 8.25E-10 |
| CDKN2A | ASB16-AS1 | 0.430783945 | 2.20E-08 |
| CFLAR | ASB16-AS1 | 0.313425645 | 7.16E-05 |
| CX3CL1 | ASB16-AS1 | 0.310953931 | 8.21E-05 |
| CXCR4 | ASB16-AS1 | 0.505776604 | 1.91E-11 |
| DAPK2 | ASB16-AS1 | 0.332901607 | 2.31E-05 |
| EEF2K | ASB16-AS1 | 0.3428421 | 1.26E-05 |
| HDAC6 | ASB16-AS1 | 0.552141906 | 9.53E-14 |
| IKBKB | ASB16-AS1 | 0.458292227 | 2.02E-09 |
| IKBKE | ASB16-AS1 | 0.351516291 | 7.30E-06 |
| ITGA6 | ASB16-AS1 | -0.309321297 | 8.99E-05 |
| KLHL24 | ASB16-AS1 | 0.335392472 | 1.99E-05 |
| MAP2K7 | ASB16-AS1 | 0.530804261 | 1.21E-12 |
| MAPK8 | ASB16-AS1 | 0.340760279 | 1.43E-05 |
| MAPK9 | ASB16-AS1 | 0.334955268 | 2.04E-05 |
| MTMR14 | ASB16-AS1 | 0.452316846 | 3.45E-09 |
| NBR1 | ASB16-AS1 | 0.340538957 | 1.45E-05 |
| NRG1 | ASB16-AS1 | -0.326108389 | 3.46E-05 |
| PARP1 | ASB16-AS1 | 0.340142474 | 1.49E-05 |
| PIK3R4 | ASB16-AS1 | 0.337629385 | 1.74E-05 |
| PINK1 | ASB16-AS1 | -0.309539681 | 8.88E-05 |
| PRKAB1 | ASB16-AS1 | 0.33110976 | 2.57E-05 |
| PTK6 | ASB16-AS1 | -0.367880574 | 2.49E-06 |
| RAF1 | ASB16-AS1 | 0.537331352 | 5.66E-13 |
| RPS6KB1 | ASB16-AS1 | 0.311515554 | 7.96E-05 |
| STK11 | ASB16-AS1 | 0.431323712 | 2.11E-08 |
| TP53 | ASB16-AS1 | 0.472690176 | 5.29E-10 |
| TP73 | ASB16-AS1 | 0.348976407 | 8.58E-06 |
| TSC1 | ASB16-AS1 | 0.404052849 | 1.85E-07 |
| TSC2 | ASB16-AS1 | 0.440028677 | 1.01E-08 |
| ULK3 | ASB16-AS1 | 0.447467958 | 5.30E-09 |
| WDR45 | ASB16-AS1 | 0.427048674 | 3.00E-08 |
| ATG5 | AL365203.2 | 0.361554704 | 3.80E-06 |
| IL24 | AL365203.2 | 0.429136047 | 2.53E-08 |
| ITGA3 | AL365203.2 | 0.556587017 | 5.49E-14 |
| ITGA6 | AL365203.2 | 0.452879799 | 3.29E-09 |
| ITGB1 | AL365203.2 | 0.514487577 | 7.49E-12 |
| ITGB4 | AL365203.2 | 0.349417352 | 8.34E-06 |
| FKBP1B | AC026356.1 | 0.306819631 | 0.000103161 |
| GABARAPL1 | AC026356.1 | 0.333989879 | 2.17E-05 |
| ATG16L2 | MIR155HG | 0.328614008 | 2.98E-05 |
| ATG4C | MIR155HG | 0.440496583 | 9.71E-09 |
| BAX | MIR155HG | 0.395938538 | 3.41E-07 |
| CAPN10 | MIR155HG | 0.416790586 | 6.87E-08 |
| CCR2 | MIR155HG | 0.513435687 | 8.40E-12 |
| CXCR4 | MIR155HG | 0.552474649 | 9.15E-14 |
| HDAC6 | MIR155HG | 0.32424255 | 3.86E-05 |
| IFNG | MIR155HG | 0.616445066 | 1.36E-17 |
| ITPR1 | MIR155HG | 0.345132966 | 1.09E-05 |
| MAP2K7 | MIR155HG | 0.32779113 | 3.13E-05 |
| MAPK9 | MIR155HG | 0.317141509 | 5.80E-05 |
| MTMR14 | MIR155HG | 0.508105747 | 1.49E-11 |
| NCKAP1 | MIR155HG | -0.320039787 | 4.92E-05 |
| RAB24 | MIR155HG | 0.35439386 | 6.06E-06 |
| RAF1 | MIR155HG | 0.32250502 | 4.27E-05 |
| RGS19 | MIR155HG | 0.461657147 | 1.48E-09 |
| STK11 | MIR155HG | 0.397519039 | 3.03E-07 |
| TP53 | MIR155HG | 0.327424494 | 3.20E-05 |
| TSC2 | MIR155HG | 0.322067656 | 4.38E-05 |
| ULK3 | MIR155HG | 0.354362813 | 6.08E-06 |
| ATIC | MINCR | 0.352436417 | 6.88E-06 |
| BAX | MINCR | 0.378306403 | 1.21E-06 |
| BID | MINCR | 0.335726254 | 1.95E-05 |
| BIRC5 | MINCR | 0.414775674 | 8.06E-08 |
| CAPN10 | MINCR | 0.312345119 | 7.60E-05 |
| CLN3 | MINCR | 0.311056529 | 8.17E-05 |
| FKBP1A | MINCR | 0.391618982 | 4.68E-07 |
| GABARAPL2 | MINCR | 0.410975895 | 1.09E-07 |
| MAPK8IP1 | MINCR | 0.402704414 | 2.05E-07 |
| MLST8 | MINCR | 0.343016507 | 1.25E-05 |
| ARSB | AC104794.2 | 0.351283519 | 7.40E-06 |
| ATG10 | AC104794.2 | 0.396738746 | 3.21E-07 |
| ATG12 | AC104794.2 | 0.342136992 | 1.32E-05 |
| ATG3 | AC104794.2 | 0.373541575 | 1.69E-06 |
| ATG4B | AC104794.2 | 0.316315657 | 6.08E-05 |
| ATG4C | AC104794.2 | 0.54047696 | 3.91E-13 |
| ATG7 | AC104794.2 | 0.404946039 | 1.73E-07 |
| BAX | AC104794.2 | 0.307631949 | 9.87E-05 |
| BCL2 | AC104794.2 | 0.600676322 | 1.43E-16 |
| BNIP3 | AC104794.2 | -0.306747901 | 0.000103567 |
| CAPN10 | AC104794.2 | 0.394559713 | 3.77E-07 |
| CASP3 | AC104794.2 | 0.366977389 | 2.64E-06 |
| CD46 | AC104794.2 | 0.390030807 | 5.26E-07 |
| CDKN1B | AC104794.2 | 0.508249637 | 1.47E-11 |
| CDKN2A | AC104794.2 | 0.415204404 | 7.79E-08 |
| CFLAR | AC104794.2 | 0.375564494 | 1.47E-06 |
| CX3CL1 | AC104794.2 | 0.37408104 | 1.63E-06 |
| CXCR4 | AC104794.2 | 0.623534894 | 4.50E-18 |
| DRAM1 | AC104794.2 | 0.4356002 | 1.47E-08 |
| EEF2K | AC104794.2 | 0.407106442 | 1.46E-07 |
| EIF2S1 | AC104794.2 | -0.306219358 | 0.000106603 |
| FAS | AC104794.2 | 0.559365271 | 3.87E-14 |
| GABARAP | AC104794.2 | 0.302370284 | 0.000131351 |
| HDAC6 | AC104794.2 | 0.555667872 | 6.16E-14 |
| HSPA5 | AC104794.2 | -0.30591588 | 0.000108383 |
| IKBKB | AC104794.2 | 0.399504792 | 2.61E-07 |
| IKBKE | AC104794.2 | 0.507988073 | 1.51E-11 |
| ITGA3 | AC104794.2 | -0.307518928 | 9.93E-05 |
| ITGA6 | AC104794.2 | -0.337844165 | 1.71E-05 |
| KLHL24 | AC104794.2 | 0.300849913 | 0.000142528 |
| MAP2K7 | AC104794.2 | 0.378665081 | 1.18E-06 |
| MAPK8 | AC104794.2 | 0.512323176 | 9.48E-12 |
| MAPK9 | AC104794.2 | 0.309673542 | 8.82E-05 |
| MTMR14 | AC104794.2 | 0.481440197 | 2.27E-10 |
| NKX2-3 | AC104794.2 | 0.539364816 | 4.46E-13 |
| NRG1 | AC104794.2 | -0.344204964 | 1.16E-05 |
| PARP1 | AC104794.2 | 0.433413304 | 1.77E-08 |
| PIK3R4 | AC104794.2 | 0.383675694 | 8.31E-07 |
| PINK1 | AC104794.2 | -0.308928015 | 9.19E-05 |
| PRKAB1 | AC104794.2 | 0.521382101 | 3.51E-12 |
| PTK6 | AC104794.2 | -0.361969928 | 3.69E-06 |
| RAB33B | AC104794.2 | 0.314620958 | 6.69E-05 |
| RAF1 | AC104794.2 | 0.514035922 | 7.87E-12 |
| SIRT1 | AC104794.2 | 0.306184154 | 0.000106808 |
| STK11 | AC104794.2 | 0.380298461 | 1.06E-06 |
| TP53 | AC104794.2 | 0.571106423 | 8.53E-15 |
| TP73 | AC104794.2 | 0.57209222 | 7.49E-15 |
| TSC1 | AC104794.2 | 0.319995091 | 4.93E-05 |
| TSC2 | AC104794.2 | 0.623717703 | 4.37E-18 |
| ULK3 | AC104794.2 | 0.396479589 | 3.27E-07 |
| WDR45 | AC104794.2 | 0.345485071 | 1.07E-05 |
| BIRC6 | AL133243.2 | 0.407206011 | 1.45E-07 |
| CASP8 | AL133243.2 | 0.303116173 | 0.000126172 |
| CD46 | AL133243.2 | 0.31992967 | 4.95E-05 |
| CDKN1A | AL133243.2 | -0.306449281 | 0.000105272 |
| EIF2AK2 | AL133243.2 | 0.306122354 | 0.000107169 |
| EIF2AK3 | AL133243.2 | 0.401353758 | 2.27E-07 |
| FOXO3 | AL133243.2 | 0.340820964 | 1.43E-05 |
| GABARAPL1 | AL133243.2 | 0.469719496 | 7.01E-10 |
| KLHL24 | AL133243.2 | 0.442227291 | 8.36E-09 |
| NBR1 | AL133243.2 | 0.340301791 | 1.47E-05 |
| NFE2L2 | AL133243.2 | 0.441528217 | 8.88E-09 |
| ST13 | AL133243.2 | 0.306596252 | 0.00010443 |
| UVRAG | AL133243.2 | 0.327780665 | 3.14E-05 |
| RAB24 | AL138724.1 | 0.307650482 | 9.86E-05 |
| STK11 | AL138724.1 | 0.428612531 | 2.64E-08 |
| BID | ZNF528-AS1 | 0.351416584 | 7.34E-06 |
| DAPK2 | ZNF528-AS1 | 0.385697261 | 7.19E-07 |
| ATG4B | AC083964.1 | 0.340785763 | 1.43E-05 |
| ATG4D | AC083964.1 | 0.436498953 | 1.36E-08 |
| BCL2 | AC083964.1 | 0.300329627 | 0.000146552 |
| BID | AC083964.1 | 0.330377301 | 2.69E-05 |
| CAPN10 | AC083964.1 | 0.358515005 | 4.64E-06 |
| CXCR4 | AC083964.1 | 0.35617888 | 5.40E-06 |
| DRAM1 | AC083964.1 | 0.40120843 | 2.30E-07 |
| GABARAP | AC083964.1 | 0.317851773 | 5.57E-05 |
| MAP1LC3A | AC083964.1 | 0.363781461 | 3.27E-06 |
| NKX2-3 | AC083964.1 | 0.456613006 | 2.35E-09 |
| PELP1 | AC083964.1 | 0.344772476 | 1.12E-05 |
| PRKAB1 | AC083964.1 | 0.305516984 | 0.000110766 |
| TSC2 | AC083964.1 | 0.331268023 | 2.55E-05 |
| ATG10 | AL161431.1 | -0.308559311 | 9.38E-05 |
| ATG4C | AL161431.1 | -0.323940576 | 3.93E-05 |
| CD46 | AL161431.1 | -0.308182117 | 9.57E-05 |
| HDAC6 | AL161431.1 | -0.304203729 | 0.000118961 |
| IKBKE | AL161431.1 | -0.341169466 | 1.40E-05 |
| ITGA3 | AL161431.1 | 0.347547015 | 9.39E-06 |
| ITGA6 | AL161431.1 | 0.401215246 | 2.30E-07 |
| MAP1LC3B | AL161431.1 | 0.326328728 | 3.41E-05 |
| MTMR14 | AL161431.1 | -0.301398768 | 0.000138395 |
| NRG1 | AL161431.1 | 0.434765004 | 1.58E-08 |
| PINK1 | AL161431.1 | 0.386973645 | 6.56E-07 |
| TP53 | AL161431.1 | -0.319324554 | 5.12E-05 |
| ARSB | SMIM25 | 0.428803346 | 2.60E-08 |
| CCL2 | SMIM25 | 0.311506772 | 7.96E-05 |
| CCR2 | SMIM25 | 0.368008335 | 2.47E-06 |
| CTSB | SMIM25 | 0.353312081 | 6.50E-06 |
| PEA15 | SMIM25 | 0.310660849 | 8.35E-05 |
| PRKCD | SMIM25 | 0.308829561 | 9.24E-05 |
| SERPINA1 | SMIM25 | 0.366346586 | 2.76E-06 |
| ARNT | LINC00265 | 0.32520283 | 3.65E-05 |
| ATG9B | LINC00265 | 0.384678368 | 7.73E-07 |
| BECN1 | LINC00265 | 0.373169407 | 1.73E-06 |
| BIRC6 | LINC00265 | 0.475457728 | 4.06E-10 |
| CASP8 | LINC00265 | 0.352734052 | 6.75E-06 |
| CD46 | LINC00265 | 0.314880973 | 6.59E-05 |
| CDKN1B | LINC00265 | 0.326590169 | 3.36E-05 |
| EDEM1 | LINC00265 | 0.314390349 | 6.78E-05 |
| EEF2K | LINC00265 | 0.35135782 | 7.37E-06 |
| EIF2AK3 | LINC00265 | 0.501822739 | 2.89E-11 |
| ERN1 | LINC00265 | 0.304676427 | 0.000115948 |
| HDAC6 | LINC00265 | 0.327073316 | 3.27E-05 |
| HGS | LINC00265 | 0.306289164 | 0.000106197 |
| IKBKB | LINC00265 | 0.368266412 | 2.42E-06 |
| MAP2K7 | LINC00265 | 0.301108887 | 0.000140564 |
| MTOR | LINC00265 | 0.303374021 | 0.000124426 |
| NBR1 | LINC00265 | 0.389544672 | 5.45E-07 |
| NFE2L2 | LINC00265 | 0.318420425 | 5.39E-05 |
| PIK3R4 | LINC00265 | 0.368756656 | 2.34E-06 |
| PRKCD | LINC00265 | 0.302545114 | 0.00013012 |
| RAF1 | LINC00265 | 0.398659567 | 2.78E-07 |
| SPNS1 | LINC00265 | 0.373127809 | 1.74E-06 |
| TP53INP2 | LINC00265 | 0.342128925 | 1.32E-05 |
| TSC1 | LINC00265 | 0.43460292 | 1.60E-08 |
| ULK3 | LINC00265 | 0.344359385 | 1.15E-05 |
| UVRAG | LINC00265 | 0.311200027 | 8.10E-05 |
| ATG10 | AC105942.1 | 0.300456274 | 0.000145563 |
| ATG4C | AC105942.1 | 0.424706468 | 3.63E-08 |
| ATG7 | AC105942.1 | 0.304083336 | 0.00011974 |
| ATIC | AC105942.1 | 0.383804101 | 8.23E-07 |
| BCL2 | AC105942.1 | 0.416288549 | 7.15E-08 |
| BIRC5 | AC105942.1 | 0.370959376 | 2.02E-06 |
| BNIP1 | AC105942.1 | 0.434772264 | 1.58E-08 |
| CDKN1B | AC105942.1 | 0.306425748 | 0.000105408 |
| DRAM1 | AC105942.1 | 0.433855771 | 1.71E-08 |
| EIF4G1 | AC105942.1 | -0.350241093 | 7.91E-06 |
| FAS | AC105942.1 | 0.407555609 | 1.42E-07 |
| FKBP1A | AC105942.1 | 0.45996902 | 1.73E-09 |
| GABARAP | AC105942.1 | 0.421542056 | 4.70E-08 |
| HDAC6 | AC105942.1 | 0.406397466 | 1.55E-07 |
| MAP1LC3A | AC105942.1 | 0.506996053 | 1.68E-11 |
| MAPK8 | AC105942.1 | 0.348399714 | 8.90E-06 |
| MAPK8IP1 | AC105942.1 | 0.43180652 | 2.02E-08 |
| MAPK9 | AC105942.1 | 0.303769505 | 0.000121792 |
| MTMR14 | AC105942.1 | 0.328643541 | 2.98E-05 |
| NKX2-3 | AC105942.1 | 0.435667124 | 1.46E-08 |
| PRKAB1 | AC105942.1 | 0.350640638 | 7.71E-06 |
| PRKAR1A | AC105942.1 | 0.334554365 | 2.09E-05 |
| ULK2 | AC105942.1 | 0.338150554 | 1.68E-05 |
| WDR45 | AC105942.1 | 0.393770021 | 4.00E-07 |
| ATIC | DANCR | 0.318082703 | 5.50E-05 |
| BAK1 | DANCR | -0.329964647 | 2.76E-05 |
| BID | DANCR | 0.31532891 | 6.43E-05 |
| BIRC5 | DANCR | 0.301590971 | 0.000136974 |
| ITGA3 | DANCR | -0.374779331 | 1.55E-06 |
| MAPK8IP1 | DANCR | 0.308227614 | 9.55E-05 |
| RHEB | DANCR | 0.316541244 | 6.00E-05 |
| ST13 | DANCR | 0.303302144 | 0.00012491 |
| WIPI1 | DANCR | 0.358163998 | 4.75E-06 |
| ZFYVE1 | DANCR | -0.307017448 | 0.00010205 |
| ATG16L2 | AC005083.1 | 0.362773206 | 3.50E-06 |
| ATG9B | AC005083.1 | 0.405374088 | 1.67E-07 |
| CAPN10 | AC005083.1 | 0.350755178 | 7.66E-06 |
| CASP3 | AC005083.1 | 0.352120239 | 7.02E-06 |
| DIRAS3 | AC005083.1 | 0.332986902 | 2.30E-05 |
| ITGA3 | AC005083.1 | -0.320722671 | 4.73E-05 |
| RAB11A | AC005083.1 | 0.481438241 | 2.28E-10 |
| RAF1 | AC005083.1 | 0.334091996 | 2.15E-05 |
| TP53INP2 | AC005083.1 | 0.395405954 | 3.54E-07 |
| ULK3 | AC005083.1 | 0.468895099 | 7.58E-10 |
| ATG12 | CCDC18-AS1 | 0.323341887 | 4.06E-05 |
| ATG16L2 | CCDC18-AS1 | 0.417646568 | 6.42E-08 |
| ATG4C | CCDC18-AS1 | 0.425145092 | 3.51E-08 |
| CAPN10 | CCDC18-AS1 | 0.454433579 | 2.86E-09 |
| CD46 | CCDC18-AS1 | 0.304499148 | 0.000117069 |
| CXCR4 | CCDC18-AS1 | 0.418784577 | 5.86E-08 |
| DIRAS3 | CCDC18-AS1 | 0.313256929 | 7.22E-05 |
| HDAC6 | CCDC18-AS1 | 0.304006305 | 0.00012024 |
| IKBKB | CCDC18-AS1 | 0.303765325 | 0.00012182 |
| ULK3 | CCDC18-AS1 | 0.331244448 | 2.55E-05 |
| ATG4B | JPX | 0.300113487 | 0.000148255 |
| ATG4D | JPX | 0.40353136 | 1.93E-07 |
| ATIC | JPX | 0.314325793 | 6.80E-05 |
| BIRC5 | JPX | 0.359427606 | 4.37E-06 |
| CAPN10 | JPX | 0.348091369 | 9.07E-06 |
| CASP3 | JPX | 0.30356866 | 0.000123123 |
| CDKN1A | JPX | -0.356536876 | 5.28E-06 |
| CLN3 | JPX | 0.336771918 | 1.83E-05 |
| GABARAP | JPX | 0.349126692 | 8.49E-06 |
| GABARAPL1 | JPX | 0.318940432 | 5.24E-05 |
| GNAI3 | JPX | -0.341884804 | 1.34E-05 |
| HDAC6 | JPX | 0.509466033 | 1.29E-11 |
| IKBKB | JPX | 0.378107161 | 1.23E-06 |
| IKBKE | JPX | 0.304937579 | 0.000114314 |
| ITGA3 | JPX | -0.32162754 | 4.49E-05 |
| KLHL24 | JPX | 0.37581285 | 1.44E-06 |
| MAP2K7 | JPX | 0.351941249 | 7.10E-06 |
| MAPK3 | JPX | -0.31104769 | 8.17E-05 |
| MAPK9 | JPX | 0.358808071 | 4.55E-06 |
| PARP1 | JPX | 0.379672899 | 1.10E-06 |
| PELP1 | JPX | 0.400392568 | 2.44E-07 |
| PINK1 | JPX | -0.30119238 | 0.000139936 |
| PRKAB1 | JPX | 0.309872442 | 8.72E-05 |
| PTK6 | JPX | -0.323247182 | 4.09E-05 |
| VAMP7 | JPX | 0.361487206 | 3.81E-06 |
| WDR45 | JPX | 0.537122225 | 5.80E-13 |
| ITGB4 | GAS6-DT | 0.313527291 | 7.11E-05 |
| SERPINA1 | GAS6-DT | 0.314369185 | 6.79E-05 |
| ATG10 | THUMPD3-AS1 | 0.368462559 | 2.39E-06 |
| ATG12 | THUMPD3-AS1 | 0.339910764 | 1.51E-05 |
| ATG16L2 | THUMPD3-AS1 | 0.46044796 | 1.66E-09 |
| ATG3 | THUMPD3-AS1 | 0.385938801 | 7.07E-07 |
| ATG4A | THUMPD3-AS1 | 0.357079835 | 5.09E-06 |
| ATG4B | THUMPD3-AS1 | 0.331565698 | 2.50E-05 |
| ATG4C | THUMPD3-AS1 | 0.417940162 | 6.27E-08 |
| ATG7 | THUMPD3-AS1 | 0.351942424 | 7.10E-06 |
| ATG9B | THUMPD3-AS1 | 0.358387715 | 4.68E-06 |
| BAG3 | THUMPD3-AS1 | -0.3040222 | 0.000120137 |
| BAX | THUMPD3-AS1 | 0.393246848 | 4.16E-07 |
| BIRC5 | THUMPD3-AS1 | 0.370532652 | 2.08E-06 |
| CAPN10 | THUMPD3-AS1 | 0.497732366 | 4.43E-11 |
| CDKN1B | THUMPD3-AS1 | 0.319622443 | 5.04E-05 |
| CDKN2A | THUMPD3-AS1 | 0.318611782 | 5.34E-05 |
| CFLAR | THUMPD3-AS1 | 0.349718911 | 8.18E-06 |
| CXCR4 | THUMPD3-AS1 | 0.487097582 | 1.30E-10 |
| GABARAPL2 | THUMPD3-AS1 | 0.303233202 | 0.000125377 |
| HDAC6 | THUMPD3-AS1 | 0.427451233 | 2.90E-08 |
| IKBKB | THUMPD3-AS1 | 0.396408336 | 3.29E-07 |
| ITGA3 | THUMPD3-AS1 | -0.342687972 | 1.27E-05 |
| ITGA6 | THUMPD3-AS1 | -0.347174248 | 9.61E-06 |
| ITGB4 | THUMPD3-AS1 | -0.306236355 | 0.000106504 |
| MAP2K7 | THUMPD3-AS1 | 0.348041131 | 9.10E-06 |
| MAPK8 | THUMPD3-AS1 | 0.306830581 | 0.000103099 |
| MAPK8IP1 | THUMPD3-AS1 | 0.334787053 | 2.06E-05 |
| MTMR14 | THUMPD3-AS1 | 0.585267994 | 1.27E-15 |
| PINK1 | THUMPD3-AS1 | -0.326368328 | 3.41E-05 |
| RAB24 | THUMPD3-AS1 | 0.38833818 | 5.94E-07 |
| RAB5A | THUMPD3-AS1 | 0.306119147 | 0.000107188 |
| RAF1 | THUMPD3-AS1 | 0.605790564 | 6.76E-17 |
| STK11 | THUMPD3-AS1 | 0.328678794 | 2.97E-05 |
| TP53 | THUMPD3-AS1 | 0.303198724 | 0.00012561 |
| TSC2 | THUMPD3-AS1 | 0.343893787 | 1.18E-05 |
| ULK3 | THUMPD3-AS1 | 0.486579169 | 1.37E-10 |
| WDR45 | THUMPD3-AS1 | 0.447492509 | 5.29E-09 |
| FADD | LINC01615 | 0.320338989 | 4.83E-05 |
| GOPC | LINC01615 | 0.347151743 | 9.62E-06 |
| IL24 | LINC01615 | 0.596552381 | 2.60E-16 |
| ITGA3 | LINC01615 | 0.326765566 | 3.33E-05 |
| ITGA6 | LINC01615 | 0.325120942 | 3.66E-05 |
| ITGB4 | LINC01615 | 0.325718981 | 3.54E-05 |
| ULK3 | LINC01615 | -0.30083917 | 0.00014261 |
| ATG4D | LINC01116 | -0.315227912 | 6.47E-05 |
| BAK1 | LINC01116 | 0.349224918 | 8.44E-06 |
| CASP1 | LINC01116 | 0.307206982 | 0.000100996 |
| ITGA3 | LINC01116 | 0.364691604 | 3.08E-06 |
| PRKAB1 | LINC01116 | -0.308072029 | 9.63E-05 |
| DIRAS3 | STX17-AS1 | 0.413300262 | 9.05E-08 |
| FKBP1B | STX17-AS1 | 0.339651001 | 1.53E-05 |
| FOXO1 | STX17-AS1 | -0.331470196 | 2.52E-05 |
| ATF6 | AL158206.1 | 0.369488499 | 2.23E-06 |
| CASP3 | AL158206.1 | 0.384426899 | 7.87E-07 |
| PIK3C3 | AL158206.1 | 0.482838823 | 1.98E-10 |
| RHEB | AL158206.1 | 0.350402107 | 7.83E-06 |
| SQSTM1 | AL158206.1 | 0.424361062 | 3.74E-08 |
| GABARAPL1 | ASH1L-AS1 | 0.339710299 | 1.53E-05 |
| ATG9B | AC009237.14 | 0.451605476 | 3.68E-09 |
| BECN1 | AC009237.14 | 0.349009166 | 8.56E-06 |
| BIRC6 | AC009237.14 | 0.312963577 | 7.34E-05 |
| CASP8 | AC009237.14 | 0.363090378 | 3.43E-06 |
| EIF2AK3 | AC009237.14 | 0.470450484 | 6.54E-10 |
| HGS | AC009237.14 | 0.318820943 | 5.27E-05 |
| NFE2L2 | AC009237.14 | 0.334891114 | 2.05E-05 |
| RAB1A | AC009237.14 | 0.332741977 | 2.33E-05 |
| RAF1 | AC009237.14 | 0.301886779 | 0.000134814 |
| TP53INP2 | AC009237.14 | 0.321664624 | 4.48E-05 |
| WIPI1 | AC009237.14 | 0.35658862 | 5.26E-06 |
| PPP1R15A | BX539320.1 | 0.336798459 | 1.83E-05 |
| ARNT | AL049840.4 | -0.437987729 | 1.20E-08 |
| ARSB | AL049840.4 | -0.348461114 | 8.86E-06 |
| ATF6 | AL049840.4 | -0.393724502 | 4.01E-07 |
| ATG4B | AL049840.4 | 0.320576166 | 4.77E-05 |
| BAK1 | AL049840.4 | 0.348577612 | 8.80E-06 |
| BECN1 | AL049840.4 | -0.337770326 | 1.72E-05 |
| BIRC6 | AL049840.4 | -0.522395595 | 3.13E-12 |
| CAMKK2 | AL049840.4 | -0.356333695 | 5.35E-06 |
| CANX | AL049840.4 | -0.403862671 | 1.88E-07 |
| CASP1 | AL049840.4 | 0.38726923 | 6.42E-07 |
| CASP4 | AL049840.4 | 0.32619759 | 3.44E-05 |
| CD46 | AL049840.4 | -0.377374718 | 1.30E-06 |
| EEF2 | AL049840.4 | -0.311275074 | 8.07E-05 |
| EEF2K | AL049840.4 | -0.332219072 | 2.41E-05 |
| EIF2AK2 | AL049840.4 | -0.320684587 | 4.74E-05 |
| EIF2AK3 | AL049840.4 | -0.491638077 | 8.25E-11 |
| EIF4G1 | AL049840.4 | -0.346716797 | 9.89E-06 |
| ERN1 | AL049840.4 | -0.36085368 | 3.98E-06 |
| FOXO1 | AL049840.4 | -0.316528722 | 6.01E-05 |
| FOXO3 | AL049840.4 | -0.38816276 | 6.02E-07 |
| GABARAPL1 | AL049840.4 | -0.382326553 | 9.14E-07 |
| ITPR1 | AL049840.4 | -0.364702531 | 3.08E-06 |
| KIF5B | AL049840.4 | -0.393409744 | 4.11E-07 |
| KLHL24 | AL049840.4 | -0.402039205 | 2.16E-07 |
| LAMP2 | AL049840.4 | -0.365565709 | 2.91E-06 |
| MAPK1 | AL049840.4 | -0.430485153 | 2.26E-08 |
| MAPK8 | AL049840.4 | -0.317098151 | 5.82E-05 |
| MBTPS2 | AL049840.4 | -0.436159833 | 1.40E-08 |
| MTOR | AL049840.4 | -0.329083802 | 2.90E-05 |
| NAF1 | AL049840.4 | -0.321267442 | 4.58E-05 |
| NBR1 | AL049840.4 | -0.493799248 | 6.62E-11 |
| NCKAP1 | AL049840.4 | -0.434969646 | 1.55E-08 |
| NFE2L2 | AL049840.4 | -0.326561719 | 3.37E-05 |
| PARP1 | AL049840.4 | -0.403854948 | 1.88E-07 |
| PIK3R4 | AL049840.4 | -0.397628744 | 3.00E-07 |
| PRKAR1A | AL049840.4 | -0.341727544 | 1.35E-05 |
| PRKCD | AL049840.4 | -0.3161213 | 6.15E-05 |
| RAB24 | AL049840.4 | 0.322135518 | 4.36E-05 |
| RAB33B | AL049840.4 | -0.306585962 | 0.000104488 |
| RAB5A | AL049840.4 | -0.351926917 | 7.11E-06 |
| RB1 | AL049840.4 | -0.344613307 | 1.13E-05 |
| RB1CC1 | AL049840.4 | -0.434144133 | 1.66E-08 |
| RPS6KB1 | AL049840.4 | -0.336409381 | 1.87E-05 |
| SIRT1 | AL049840.4 | -0.402289492 | 2.12E-07 |
| SQSTM1 | AL049840.4 | -0.322816104 | 4.19E-05 |
| ST13 | AL049840.4 | -0.369419859 | 2.24E-06 |
| TP63 | AL049840.4 | -0.338264006 | 1.67E-05 |
| USP10 | AL049840.4 | -0.359654557 | 4.30E-06 |
| UVRAG | AL049840.4 | -0.352904407 | 6.67E-06 |
| VAMP7 | AL049840.4 | -0.363794434 | 3.27E-06 |
| WDFY3 | AL049840.4 | -0.377382932 | 1.29E-06 |
| WIPI1 | AL049840.4 | -0.317492659 | 5.69E-05 |
| ARNT | AL139246.5 | -0.350871835 | 7.60E-06 |
| ARSB | AL139246.5 | -0.357142181 | 5.07E-06 |
| ATG16L2 | AL139246.5 | 0.392218355 | 4.48E-07 |
| BIRC6 | AL139246.5 | -0.377284975 | 1.30E-06 |
| CASP4 | AL139246.5 | 0.308365931 | 9.48E-05 |
| EIF2AK2 | AL139246.5 | -0.303741906 | 0.000121974 |
| EIF2AK3 | AL139246.5 | -0.309693792 | 8.81E-05 |
| FOXO1 | AL139246.5 | -0.323270509 | 4.08E-05 |
| FOXO3 | AL139246.5 | -0.338942457 | 1.60E-05 |
| GABARAPL2 | AL139246.5 | 0.43367558 | 1.73E-08 |
| GOPC | AL139246.5 | -0.315942044 | 6.21E-05 |
| LAMP2 | AL139246.5 | -0.335411075 | 1.99E-05 |
| MAPK1 | AL139246.5 | -0.394453935 | 3.80E-07 |
| NBR1 | AL139246.5 | -0.310098058 | 8.61E-05 |
| PARP1 | AL139246.5 | -0.360500769 | 4.07E-06 |
| PTK6 | AL139246.5 | 0.34685213 | 9.81E-06 |
| RAB24 | AL139246.5 | 0.356577424 | 5.26E-06 |
| RAB33B | AL139246.5 | -0.329228727 | 2.88E-05 |
| RB1 | AL139246.5 | -0.300864656 | 0.000142416 |
| ARNT | AL365361.1 | 0.301150133 | 0.000140253 |
| ARSB | AL365361.1 | 0.399762882 | 2.56E-07 |
| ATG4C | AL365361.1 | 0.408928174 | 1.27E-07 |
| ATG7 | AL365361.1 | 0.312110764 | 7.70E-05 |
| BCL2 | AL365361.1 | 0.430336631 | 2.29E-08 |
| CCR2 | AL365361.1 | 0.759964214 | 1.99E-30 |
| CDKN1B | AL365361.1 | 0.351533602 | 7.29E-06 |
| CXCR4 | AL365361.1 | 0.467632552 | 8.53E-10 |
| DRAM1 | AL365361.1 | 0.409529294 | 1.21E-07 |
| EDEM1 | AL365361.1 | 0.391090983 | 4.87E-07 |
| EEF2K | AL365361.1 | 0.331764368 | 2.48E-05 |
| ERN1 | AL365361.1 | 0.353466557 | 6.44E-06 |
| FAS | AL365361.1 | 0.340010343 | 1.50E-05 |
| HDAC6 | AL365361.1 | 0.311394096 | 8.01E-05 |
| IFNG | AL365361.1 | 0.546246424 | 1.96E-13 |
| ITPR1 | AL365361.1 | 0.612123572 | 2.62E-17 |
| MTMR14 | AL365361.1 | 0.425036899 | 3.54E-08 |
| NKX2-3 | AL365361.1 | 0.355824475 | 5.53E-06 |
| PARP1 | AL365361.1 | 0.331973589 | 2.44E-05 |
| PRKCD | AL365361.1 | 0.345695613 | 1.05E-05 |
| RAF1 | AL365361.1 | 0.319482397 | 5.08E-05 |
| RGS19 | AL365361.1 | 0.464314695 | 1.16E-09 |
| SIRT1 | AL365361.1 | 0.346715018 | 9.89E-06 |
| TP53 | AL365361.1 | 0.387706734 | 6.22E-07 |
| ULK2 | AL365361.1 | 0.34108485 | 1.41E-05 |
| EGFR | AL133330.1 | 0.507301646 | 1.62E-11 |
| GOPC | AL133330.1 | 0.445458918 | 6.32E-09 |
| HIF1A | AL133330.1 | 0.305107835 | 0.000113261 |
| IL24 | AL133330.1 | 0.30821899 | 9.55E-05 |
| ITGA3 | AL133330.1 | 0.341503182 | 1.37E-05 |
| ITGA6 | AL133330.1 | 0.317759346 | 5.60E-05 |
| NPC1 | AL133330.1 | 0.344551174 | 1.13E-05 |
| ATF4 | AC009065.8 | 0.383542949 | 8.39E-07 |
| ATG16L2 | AC009065.8 | 0.403618657 | 1.91E-07 |
| ATG2B | AC009065.8 | -0.315034754 | 6.54E-05 |
| ATG3 | AC009065.8 | 0.39357395 | 4.06E-07 |
| ATG4B | AC009065.8 | 0.519735344 | 4.21E-12 |
| ATG4D | AC009065.8 | 0.401844584 | 2.19E-07 |
| BAX | AC009065.8 | 0.33102056 | 2.59E-05 |
| BNIP1 | AC009065.8 | 0.361457482 | 3.82E-06 |
| CAPN10 | AC009065.8 | 0.548020303 | 1.58E-13 |
| CDKN2A | AC009065.8 | 0.303003478 | 0.000126942 |
| CLN3 | AC009065.8 | 0.372230676 | 1.85E-06 |
| DAPK2 | AC009065.8 | 0.386635086 | 6.72E-07 |
| DLC1 | AC009065.8 | -0.32681675 | 3.32E-05 |
| HDAC6 | AC009065.8 | 0.320280062 | 4.85E-05 |
| IKBKB | AC009065.8 | 0.311860494 | 7.81E-05 |
| IKBKE | AC009065.8 | 0.320126352 | 4.89E-05 |
| ITGA6 | AC009065.8 | -0.324906611 | 3.71E-05 |
| MLST8 | AC009065.8 | 0.405739571 | 1.63E-07 |
| MTMR14 | AC009065.8 | 0.316553731 | 6.00E-05 |
| NCKAP1 | AC009065.8 | -0.30795215 | 9.69E-05 |
| NKX2-3 | AC009065.8 | 0.319950977 | 4.94E-05 |
| PELP1 | AC009065.8 | 0.335225834 | 2.01E-05 |
| PEX14 | AC009065.8 | 0.309144035 | 9.08E-05 |
| PINK1 | AC009065.8 | -0.38674176 | 6.67E-07 |
| RAB24 | AC009065.8 | 0.404677045 | 1.76E-07 |
| RAF1 | AC009065.8 | 0.311039595 | 8.17E-05 |
| STK11 | AC009065.8 | 0.369334441 | 2.25E-06 |
| TSC2 | AC009065.8 | 0.398128556 | 2.89E-07 |
| ULK3 | AC009065.8 | 0.482588495 | 2.03E-10 |
| WDR45 | AC009065.8 | 0.462848206 | 1.33E-09 |
| ATG3 | AL079303.1 | 0.32874171 | 2.96E-05 |
| ATG4A | AL079303.1 | 0.307042893 | 0.000101908 |
| ATG4C | AL079303.1 | 0.343013998 | 1.25E-05 |
| ATG7 | AL079303.1 | 0.424542034 | 3.68E-08 |
| BIRC5 | AL079303.1 | 0.335079471 | 2.03E-05 |
| CAPNS1 | AL079303.1 | 0.308170753 | 9.58E-05 |
| CDKN1B | AL079303.1 | 0.426530294 | 3.13E-08 |
| CDKN2A | AL079303.1 | 0.347762527 | 9.26E-06 |
| HDAC1 | AL079303.1 | 0.313704512 | 7.04E-05 |
| HDAC6 | AL079303.1 | 0.425927224 | 3.29E-08 |
| MAP2K7 | AL079303.1 | 0.337152301 | 1.79E-05 |
| MAPK8 | AL079303.1 | 0.325764844 | 3.53E-05 |
| MAPK8IP1 | AL079303.1 | 0.386247447 | 6.91E-07 |
| MTMR14 | AL079303.1 | 0.42790002 | 2.80E-08 |
| NKX2-3 | AL079303.1 | 0.311548995 | 7.95E-05 |
| PRKAB1 | AL079303.1 | 0.442370714 | 8.26E-09 |
| PRKAR1A | AL079303.1 | 0.33547734 | 1.98E-05 |
| RAF1 | AL079303.1 | 0.481993957 | 2.15E-10 |
| TP53 | AL079303.1 | 0.318270238 | 5.44E-05 |
| ATG16L2 | RUSC1-AS1 | 0.37816258 | 1.23E-06 |
| ATG3 | RUSC1-AS1 | 0.382349523 | 9.13E-07 |
| ATG4B | RUSC1-AS1 | 0.343550978 | 1.21E-05 |
| CAPN10 | RUSC1-AS1 | 0.43067156 | 2.22E-08 |
| CXCR4 | RUSC1-AS1 | 0.370064103 | 2.14E-06 |
| DAPK2 | RUSC1-AS1 | 0.335234105 | 2.01E-05 |
| HDAC6 | RUSC1-AS1 | 0.381273793 | 9.85E-07 |
| IKBKB | RUSC1-AS1 | 0.383208668 | 8.59E-07 |
| MAP2K7 | RUSC1-AS1 | 0.370156894 | 2.13E-06 |
| PINK1 | RUSC1-AS1 | -0.340050331 | 1.50E-05 |
| RAF1 | RUSC1-AS1 | 0.391703358 | 4.65E-07 |
| STK11 | RUSC1-AS1 | 0.380029376 | 1.08E-06 |
| TSC1 | RUSC1-AS1 | 0.381108161 | 9.97E-07 |
| ULK3 | RUSC1-AS1 | 0.449679951 | 4.36E-09 |
| WDR45 | RUSC1-AS1 | 0.347570332 | 9.37E-06 |
| ARNT | LINC01137 | -0.355400036 | 5.68E-06 |
| ARSB | LINC01137 | -0.326579038 | 3.36E-05 |
| ATF6 | LINC01137 | -0.342540765 | 1.28E-05 |
| BAK1 | LINC01137 | 0.370471975 | 2.09E-06 |
| BCL2 | LINC01137 | -0.305650329 | 0.000109964 |
| BIRC6 | LINC01137 | -0.423222273 | 4.10E-08 |
| CANX | LINC01137 | -0.310729537 | 8.32E-05 |
| CASP1 | LINC01137 | 0.479713078 | 2.69E-10 |
| CASP4 | LINC01137 | 0.378012571 | 1.24E-06 |
| CD46 | LINC01137 | -0.372573384 | 1.81E-06 |
| CDKN1A | LINC01137 | 0.302067856 | 0.000133507 |
| CDKN1B | LINC01137 | -0.358405081 | 4.67E-06 |
| DRAM1 | LINC01137 | -0.303586992 | 0.000123001 |
| EEF2 | LINC01137 | -0.401926734 | 2.17E-07 |
| EIF2AK3 | LINC01137 | -0.38864987 | 5.81E-07 |
| ERN1 | LINC01137 | -0.302339044 | 0.000131572 |
| FOXO3 | LINC01137 | -0.375394929 | 1.49E-06 |
| HDAC6 | LINC01137 | -0.34674709 | 9.87E-06 |
| KIF5B | LINC01137 | -0.340333007 | 1.47E-05 |
| KLHL24 | LINC01137 | -0.325681212 | 3.55E-05 |
| MAPK9 | LINC01137 | -0.303743461 | 0.000121964 |
| MBTPS2 | LINC01137 | -0.324881722 | 3.72E-05 |
| NAF1 | LINC01137 | -0.306319678 | 0.00010602 |
| NBR1 | LINC01137 | -0.374796771 | 1.55E-06 |
| PARP1 | LINC01137 | -0.416433836 | 7.07E-08 |
| PIK3C3 | LINC01137 | -0.319361755 | 5.11E-05 |
| PIK3R4 | LINC01137 | -0.316288056 | 6.09E-05 |
| PRKCD | LINC01137 | -0.330077978 | 2.74E-05 |
| PTK6 | LINC01137 | 0.323313739 | 4.07E-05 |
| RAF1 | LINC01137 | -0.310710131 | 8.33E-05 |
| RB1CC1 | LINC01137 | -0.378366248 | 1.21E-06 |
| RPS6KB1 | LINC01137 | -0.371010044 | 2.01E-06 |
| SIRT1 | LINC01137 | -0.369576399 | 2.22E-06 |
| TP53 | LINC01137 | -0.388003237 | 6.09E-07 |
| ULK2 | LINC01137 | -0.346179801 | 1.02E-05 |
| UVRAG | LINC01137 | -0.365466378 | 2.93E-06 |
| VAMP7 | LINC01137 | -0.30132622 | 0.000138934 |
| ATG4D | ILF3-DT | 0.327499783 | 3.19E-05 |
| BAX | ILF3-DT | 0.315510121 | 6.36E-05 |
| ITGA3 | ILF3-DT | -0.354170154 | 6.15E-06 |
| ITGB4 | ILF3-DT | -0.32879005 | 2.95E-05 |
| NFKB1 | ILF3-DT | -0.323598684 | 4.00E-05 |
| ATG9B | ELF3-AS1 | 0.355472583 | 5.65E-06 |
| BAK1 | ELF3-AS1 | -0.364080369 | 3.21E-06 |
| BID | ELF3-AS1 | 0.35759079 | 4.93E-06 |
| EIF2AK3 | ELF3-AS1 | 0.39670101 | 3.22E-07 |
| ITGA3 | ELF3-AS1 | -0.437863674 | 1.22E-08 |
| NFE2L2 | ELF3-AS1 | 0.30056131 | 0.000144747 |
| PELP1 | ELF3-AS1 | 0.300355621 | 0.000146349 |
| PINK1 | ELF3-AS1 | -0.399422438 | 2.63E-07 |
| RAB11A | ELF3-AS1 | 0.387624421 | 6.26E-07 |
| RGS19 | ELF3-AS1 | -0.329799697 | 2.78E-05 |
| TP53INP2 | ELF3-AS1 | 0.419320027 | 5.62E-08 |
| ULK3 | ELF3-AS1 | 0.32146796 | 4.53E-05 |
| ATG16L2 | ADIRF-AS1 | 0.350206078 | 7.93E-06 |
| FOXO1 | ADIRF-AS1 | -0.326214608 | 3.44E-05 |
| ATG10 | AC019171.1 | 0.404766128 | 1.75E-07 |
| ATG3 | AC019171.1 | 0.356891708 | 5.16E-06 |
| ATG4A | AC019171.1 | 0.391380204 | 4.76E-07 |
| ATG4C | AC019171.1 | 0.46537051 | 1.05E-09 |
| ATG7 | AC019171.1 | 0.303281352 | 0.000125051 |
| ATIC | AC019171.1 | 0.470786942 | 6.34E-10 |
| BAX | AC019171.1 | 0.381029538 | 1.00E-06 |
| BCL2 | AC019171.1 | 0.350951704 | 7.56E-06 |
| BIRC5 | AC019171.1 | 0.474634736 | 4.40E-10 |
| BNIP1 | AC019171.1 | 0.38665433 | 6.71E-07 |
| CDKN1B | AC019171.1 | 0.369071724 | 2.29E-06 |
| CDKN2A | AC019171.1 | 0.424740732 | 3.62E-08 |
| GABARAP | AC019171.1 | 0.336776172 | 1.83E-05 |
| HDAC6 | AC019171.1 | 0.574109488 | 5.74E-15 |
| IKBKE | AC019171.1 | 0.339831249 | 1.52E-05 |
| MAPK8IP1 | AC019171.1 | 0.612708727 | 2.40E-17 |
| MTMR14 | AC019171.1 | 0.460740916 | 1.61E-09 |
| NKX2-3 | AC019171.1 | 0.32590632 | 3.50E-05 |
| PRKAR1A | AC019171.1 | 0.429020691 | 2.55E-08 |
| RAF1 | AC019171.1 | 0.351662921 | 7.23E-06 |
| TP53 | AC019171.1 | 0.371279243 | 1.97E-06 |
| ULK2 | AC019171.1 | 0.302937693 | 0.000127393 |
| VAMP7 | AC019171.1 | 0.350038218 | 8.02E-06 |
| WDR45 | AC019171.1 | 0.42752499 | 2.89E-08 |
| ATF4 | SNHG10 | 0.333913013 | 2.18E-05 |
| ATG16L2 | SNHG10 | 0.357906277 | 4.83E-06 |
| ATG4B | SNHG10 | 0.320236335 | 4.86E-05 |
| ATIC | SNHG10 | 0.334911504 | 2.05E-05 |
| BAX | SNHG10 | 0.440705791 | 9.53E-09 |
| BIRC5 | SNHG10 | 0.418719 | 5.89E-08 |
| CAPN10 | SNHG10 | 0.416523935 | 7.02E-08 |
| MAPK8IP1 | SNHG10 | 0.337354894 | 1.77E-05 |
| MLST8 | SNHG10 | 0.344254538 | 1.15E-05 |
| PELP1 | SNHG10 | 0.327679265 | 3.15E-05 |
| WDR45 | SNHG10 | 0.38557212 | 7.26E-07 |
| ATG10 | TNRC6C-AS1 | 0.381333671 | 9.81E-07 |
| ATG12 | TNRC6C-AS1 | 0.339202092 | 1.58E-05 |
| ATG16L2 | TNRC6C-AS1 | 0.325969513 | 3.49E-05 |
| ATG3 | TNRC6C-AS1 | 0.411844195 | 1.01E-07 |
| ATG4B | TNRC6C-AS1 | 0.474902459 | 4.28E-10 |
| ATG4C | TNRC6C-AS1 | 0.381099547 | 9.97E-07 |
| ATG4D | TNRC6C-AS1 | 0.417108817 | 6.70E-08 |
| ATG7 | TNRC6C-AS1 | 0.324276535 | 3.85E-05 |
| BAX | TNRC6C-AS1 | 0.334123242 | 2.15E-05 |
| BCL2 | TNRC6C-AS1 | 0.39320735 | 4.17E-07 |
| BID | TNRC6C-AS1 | 0.340111174 | 1.49E-05 |
| CAPN10 | TNRC6C-AS1 | 0.492450421 | 7.60E-11 |
| CAPNS1 | TNRC6C-AS1 | 0.31165022 | 7.90E-05 |
| CASP3 | TNRC6C-AS1 | 0.384591194 | 7.78E-07 |
| CDKN1B | TNRC6C-AS1 | 0.341811881 | 1.34E-05 |
| CDKN2A | TNRC6C-AS1 | 0.391683522 | 4.66E-07 |
| CFLAR | TNRC6C-AS1 | 0.411685715 | 1.03E-07 |
| CXCR4 | TNRC6C-AS1 | 0.397838542 | 2.96E-07 |
| DAPK2 | TNRC6C-AS1 | 0.347881776 | 9.19E-06 |
| DRAM1 | TNRC6C-AS1 | 0.367925853 | 2.48E-06 |
| EIF2S1 | TNRC6C-AS1 | -0.309191616 | 9.06E-05 |
| GABARAP | TNRC6C-AS1 | 0.374686741 | 1.56E-06 |
| HDAC6 | TNRC6C-AS1 | 0.460795848 | 1.61E-09 |
| IKBKB | TNRC6C-AS1 | 0.331787504 | 2.47E-05 |
| IKBKE | TNRC6C-AS1 | 0.478496131 | 3.03E-10 |
| ITGA6 | TNRC6C-AS1 | -0.309741981 | 8.78E-05 |
| MAP2K7 | TNRC6C-AS1 | 0.337637305 | 1.74E-05 |
| MAPK8 | TNRC6C-AS1 | 0.423738939 | 3.93E-08 |
| MTMR14 | TNRC6C-AS1 | 0.380163263 | 1.07E-06 |
| NKX2-3 | TNRC6C-AS1 | 0.393661011 | 4.03E-07 |
| NRG1 | TNRC6C-AS1 | -0.304557307 | 0.0001167 |
| PELP1 | TNRC6C-AS1 | 0.307563564 | 9.90E-05 |
| PIK3R4 | TNRC6C-AS1 | 0.313392187 | 7.17E-05 |
| PINK1 | TNRC6C-AS1 | -0.371855886 | 1.90E-06 |
| PRKAB1 | TNRC6C-AS1 | 0.491076899 | 8.73E-11 |
| RAB24 | TNRC6C-AS1 | 0.323733704 | 3.97E-05 |
| RAF1 | TNRC6C-AS1 | 0.34244305 | 1.29E-05 |
| STK11 | TNRC6C-AS1 | 0.398356693 | 2.84E-07 |
| TP53 | TNRC6C-AS1 | 0.353882087 | 6.27E-06 |
| TP73 | TNRC6C-AS1 | 0.406907293 | 1.49E-07 |
| TSC2 | TNRC6C-AS1 | 0.484623972 | 1.66E-10 |
| ULK3 | TNRC6C-AS1 | 0.350847976 | 7.61E-06 |
| WDR45 | TNRC6C-AS1 | 0.354322867 | 6.09E-06 |
| CDKN1B | AC091563.1 | 0.349007158 | 8.56E-06 |
| MAPK8 | AC091563.1 | 0.305615534 | 0.000110173 |
| NKX2-3 | AC091563.1 | 0.338666662 | 1.63E-05 |
| PRKAB1 | AC091563.1 | 0.379551882 | 1.11E-06 |
| TSC2 | AC091563.1 | 0.329515223 | 2.83E-05 |
| BIRC5 | AC008946.1 | 0.397727164 | 2.98E-07 |
| MAP2K7 | AC008946.1 | 0.327877859 | 3.12E-05 |
| MLST8 | AC008946.1 | 0.321536554 | 4.51E-05 |
| PELP1 | AC008946.1 | 0.392580774 | 4.36E-07 |
| ATF4 | PVT1 | 0.326395241 | 3.40E-05 |
| PELP1 | PVT1 | 0.398721766 | 2.77E-07 |
| SQSTM1 | PVT1 | 0.309314174 | 8.99E-05 |
| ARSB | AC244153.1 | -0.34873558 | 8.71E-06 |
| FAS | AC244153.1 | -0.31396992 | 6.94E-05 |
| FKBP1B | AC244153.1 | 0.317640684 | 5.64E-05 |
| FOXO1 | AC244153.1 | -0.332536476 | 2.36E-05 |
| MAPK9 | AC244153.1 | -0.339741286 | 1.53E-05 |
| PTK6 | AC244153.1 | 0.328560887 | 2.99E-05 |
| TP53 | AC244153.1 | -0.348269842 | 8.97E-06 |
| ARNT | AC096733.2 | 0.341704826 | 1.35E-05 |
| BIRC6 | AC096733.2 | 0.365890716 | 2.84E-06 |
| EIF2AK3 | AC096733.2 | 0.467046759 | 9.01E-10 |
| ERN1 | AC096733.2 | 0.334827762 | 2.06E-05 |
| FOXO3 | AC096733.2 | 0.533334629 | 9.02E-13 |
| HDAC6 | AC096733.2 | 0.342270148 | 1.31E-05 |
| ITPR1 | AC096733.2 | 0.314687184 | 6.67E-05 |
| KLHL24 | AC096733.2 | 0.34595668 | 1.04E-05 |
| NAF1 | AC096733.2 | 0.307622609 | 9.87E-05 |
| NBR1 | AC096733.2 | 0.352230974 | 6.97E-06 |
| NFE2L2 | AC096733.2 | 0.428895542 | 2.58E-08 |
| PIK3R4 | AC096733.2 | 0.434746667 | 1.58E-08 |
| PRKAR1A | AC096733.2 | 0.342910692 | 1.25E-05 |
| RAB33B | AC096733.2 | 0.303980415 | 0.000120409 |
| RB1CC1 | AC096733.2 | 0.498900033 | 3.92E-11 |
| RPS6KB1 | AC096733.2 | 0.324471121 | 3.81E-05 |
| ATF4 | AC010761.1 | 0.376670176 | 1.36E-06 |
| ATG10 | AC010761.1 | 0.329398432 | 2.85E-05 |
| ATG16L2 | AC010761.1 | 0.528865088 | 1.51E-12 |
| ATG4B | AC010761.1 | 0.551202933 | 1.07E-13 |
| ATG4D | AC010761.1 | 0.306791518 | 0.00010332 |
| BAX | AC010761.1 | 0.397537912 | 3.02E-07 |
| BIRC5 | AC010761.1 | 0.312104573 | 7.70E-05 |
| BNIP1 | AC010761.1 | 0.381796755 | 9.49E-07 |
| CAPN10 | AC010761.1 | 0.589748537 | 6.81E-16 |
| CDKN2A | AC010761.1 | 0.405708271 | 1.63E-07 |
| CXCR4 | AC010761.1 | 0.408201669 | 1.35E-07 |
| DAPK2 | AC010761.1 | 0.327825129 | 3.13E-05 |
| HDAC6 | AC010761.1 | 0.312884225 | 7.38E-05 |
| IKBKB | AC010761.1 | 0.34735346 | 9.50E-06 |
| ITGA6 | AC010761.1 | -0.304049818 | 0.000119957 |
| MAP2K7 | AC010761.1 | 0.348241983 | 8.98E-06 |
| MTMR14 | AC010761.1 | 0.391050085 | 4.88E-07 |
| PELP1 | AC010761.1 | 0.362542413 | 3.56E-06 |
| PEX14 | AC010761.1 | 0.355282131 | 5.72E-06 |
| PINK1 | AC010761.1 | -0.322926576 | 4.16E-05 |
| RAB24 | AC010761.1 | 0.400576261 | 2.41E-07 |
| RAF1 | AC010761.1 | 0.305995655 | 0.000107913 |
| STK11 | AC010761.1 | 0.423718947 | 3.94E-08 |
| TSC2 | AC010761.1 | 0.305322257 | 0.000111947 |
| ULK3 | AC010761.1 | 0.341366053 | 1.38E-05 |
| WDFY3 | AC010761.1 | -0.318148072 | 5.48E-05 |
| WDR45 | AC010761.1 | 0.483235833 | 1.91E-10 |
| ATG10 | AC012073.1 | 0.331639252 | 2.49E-05 |
| ATG3 | AC012073.1 | 0.339033117 | 1.59E-05 |
| ATG4B | AC012073.1 | 0.370520406 | 2.08E-06 |
| ATG4C | AC012073.1 | 0.401924123 | 2.18E-07 |
| ATG7 | AC012073.1 | 0.328476114 | 3.01E-05 |
| BIRC5 | AC012073.1 | 0.362068298 | 3.67E-06 |
| CAPN10 | AC012073.1 | 0.338225993 | 1.67E-05 |
| CDKN1B | AC012073.1 | 0.359649309 | 4.30E-06 |
| CDKN2A | AC012073.1 | 0.417537616 | 6.47E-08 |
| EEF2K | AC012073.1 | 0.36516789 | 2.98E-06 |
| FAS | AC012073.1 | 0.313431095 | 7.15E-05 |
| HDAC1 | AC012073.1 | 0.429492383 | 2.45E-08 |
| HDAC6 | AC012073.1 | 0.414954767 | 7.95E-08 |
| HIF1A | AC012073.1 | -0.347040292 | 9.69E-06 |
| MAP2K7 | AC012073.1 | 0.481156425 | 2.34E-10 |
| MAPK8 | AC012073.1 | 0.391203111 | 4.83E-07 |
| MTMR14 | AC012073.1 | 0.374237074 | 1.61E-06 |
| NKX2-3 | AC012073.1 | 0.385064359 | 7.52E-07 |
| PARP1 | AC012073.1 | 0.358624721 | 4.60E-06 |
| PEX14 | AC012073.1 | 0.334085876 | 2.15E-05 |
| PIK3R4 | AC012073.1 | 0.348129438 | 9.05E-06 |
| PRKAB1 | AC012073.1 | 0.308122354 | 9.60E-05 |
| RAF1 | AC012073.1 | 0.436801317 | 1.33E-08 |
| STK11 | AC012073.1 | 0.500871956 | 3.20E-11 |
| TP53 | AC012073.1 | 0.365710676 | 2.88E-06 |
| TP73 | AC012073.1 | 0.476472218 | 3.68E-10 |
| TSC1 | AC012073.1 | 0.447789851 | 5.16E-09 |
| TSC2 | AC012073.1 | 0.408833502 | 1.28E-07 |
| BNIP3 | LINC02081 | 0.333615744 | 2.21E-05 |
| EIF2AK3 | LINC02081 | -0.329227684 | 2.88E-05 |
| FADD | LINC02081 | 0.323173396 | 4.10E-05 |
| IL24 | LINC02081 | 0.330488089 | 2.67E-05 |
| ITGA3 | LINC02081 | 0.474480501 | 4.46E-10 |
| ITGB4 | LINC02081 | 0.391764105 | 4.63E-07 |
| NFE2L2 | LINC02081 | -0.307340574 | 0.000100259 |
| PRKCD | LINC02081 | -0.334442212 | 2.11E-05 |
| ULK3 | LINC02081 | -0.301686058 | 0.000136276 |
| ARNT | AL121832.2 | -0.379498554 | 1.12E-06 |
| ATG4B | AL121832.2 | 0.381260694 | 9.86E-07 |
| BAX | AL121832.2 | 0.407753606 | 1.39E-07 |
| BECN1 | AL121832.2 | -0.3092741 | 9.01E-05 |
| BIRC5 | AL121832.2 | 0.379846006 | 1.09E-06 |
| CANX | AL121832.2 | -0.346194872 | 1.02E-05 |
| CAPN10 | AL121832.2 | 0.302582942 | 0.000129854 |
| EIF4EBP1 | AL121832.2 | 0.393217427 | 4.16E-07 |
| FKBP1A | AL121832.2 | 0.300464865 | 0.000145496 |
| FOXO1 | AL121832.2 | -0.326945557 | 3.29E-05 |
| GABARAPL2 | AL121832.2 | 0.301446337 | 0.000138042 |
| KIF5B | AL121832.2 | -0.311111558 | 8.14E-05 |
| MAPK1 | AL121832.2 | -0.355631568 | 5.60E-06 |
| MLST8 | AL121832.2 | 0.350744697 | 7.66E-06 |
| MTOR | AL121832.2 | -0.343823305 | 1.19E-05 |
| NBR1 | AL121832.2 | -0.306419295 | 0.000105445 |
| NFKB1 | AL121832.2 | -0.302604499 | 0.000129704 |
| RB1 | AL121832.2 | -0.375097737 | 1.52E-06 |
| SH3GLB1 | AL121832.2 | -0.325813756 | 3.52E-05 |
| WDR45 | AL121832.2 | 0.33561386 | 1.96E-05 |
| ATG4C | LINC01963 | 0.369477691 | 2.23E-06 |
| BCL2 | LINC01963 | 0.393327575 | 4.13E-07 |
| CCL2 | LINC01963 | 0.319781614 | 4.99E-05 |
| CXCR4 | LINC01963 | 0.400037271 | 2.51E-07 |
| DRAM1 | LINC01963 | 0.404765972 | 1.75E-07 |
| EEF2 | LINC01963 | 0.36391294 | 3.25E-06 |
| IKBKE | LINC01963 | 0.318018868 | 5.52E-05 |
| MAPK8 | LINC01963 | 0.385678109 | 7.20E-07 |
| MTOR | LINC01963 | 0.378765099 | 1.18E-06 |
| NKX2-3 | LINC01963 | 0.443487301 | 7.50E-09 |
| PARP1 | LINC01963 | 0.329531266 | 2.83E-05 |
| TP53 | LINC01963 | 0.339047052 | 1.59E-05 |
| TP73 | LINC01963 | 0.465342445 | 1.06E-09 |
| TSC2 | LINC01963 | 0.502190436 | 2.79E-11 |
| UVRAG | LINC01963 | 0.310325 | 8.50E-05 |
| BECN1 | AC080112.1 | 0.491665173 | 8.23E-11 |
| BIRC6 | AC080112.1 | 0.321963498 | 4.40E-05 |
| CD46 | AC080112.1 | 0.314456073 | 6.75E-05 |
| CFLAR | AC080112.1 | 0.308432892 | 9.44E-05 |
| EIF2AK3 | AC080112.1 | 0.422945144 | 4.19E-08 |
| ITGA3 | AC080112.1 | -0.300147525 | 0.000147986 |
| KLHL24 | AC080112.1 | 0.37650637 | 1.38E-06 |
| NBR1 | AC080112.1 | 0.417301307 | 6.60E-08 |
| RAB5A | AC080112.1 | 0.300410552 | 0.000145919 |
| RAF1 | AC080112.1 | 0.303493004 | 0.000123628 |
| BID | AC147651.2 | 0.319971412 | 4.94E-05 |
| PPP1R15A | AC147651.2 | 0.405397266 | 1.67E-07 |
| ATG10 | LINC02321 | 0.324091592 | 3.89E-05 |
| ATG4A | LINC02321 | 0.334104725 | 2.15E-05 |
| CX3CL1 | LINC02321 | 0.351625039 | 7.24E-06 |
| TNFSF10 | LINC02321 | 0.329267205 | 2.87E-05 |
| BAK1 | AC091271.1 | -0.347655096 | 9.32E-06 |
| ITGA3 | AC091271.1 | -0.317556364 | 5.67E-05 |
| MAPK8IP1 | AC091271.1 | 0.333969833 | 2.17E-05 |
| PRKAR1A | AC091271.1 | 0.300402685 | 0.000145981 |
| WIPI1 | AC091271.1 | 0.311948742 | 7.77E-05 |
| ARNT | AL354733.3 | 0.357268951 | 5.03E-06 |
| CAMKK2 | AL354733.3 | 0.31631717 | 6.08E-05 |
| CASP8 | AL354733.3 | 0.317211502 | 5.78E-05 |
| CD46 | AL354733.3 | 0.428469293 | 2.67E-08 |
| EEF2K | AL354733.3 | 0.418291899 | 6.10E-08 |
| HDAC6 | AL354733.3 | 0.33477948 | 2.06E-05 |
| IKBKB | AL354733.3 | 0.347956477 | 9.15E-06 |
| MAPK8 | AL354733.3 | 0.325956282 | 3.49E-05 |
| P4HB | AL354733.3 | -0.307959109 | 9.69E-05 |
| PARP1 | AL354733.3 | 0.326914124 | 3.30E-05 |
| PIK3R4 | AL354733.3 | 0.384589385 | 7.78E-07 |
| RAB33B | AL354733.3 | 0.397434518 | 3.05E-07 |
| RAF1 | AL354733.3 | 0.33006014 | 2.74E-05 |
| SIRT1 | AL354733.3 | 0.42951895 | 2.45E-08 |
| TP73 | AL354733.3 | 0.304836654 | 0.000114943 |
| TSC1 | AL354733.3 | 0.497165336 | 4.69E-11 |
| ULK2 | AL354733.3 | 0.306907315 | 0.000102667 |
| GABARAPL2 | AC009113.1 | 0.355892779 | 5.50E-06 |
| HDAC6 | AC009113.1 | 0.339771602 | 1.52E-05 |
| MAP1LC3B | AC009113.1 | 0.353225618 | 6.54E-06 |
| ATF4 | AC015982.1 | 0.362978665 | 3.45E-06 |
| ATG4B | AC015982.1 | 0.400997226 | 2.33E-07 |
| BAX | AC015982.1 | 0.315870208 | 6.24E-05 |
| BIRC5 | AC015982.1 | 0.347824476 | 9.22E-06 |
| BNIP1 | AC015982.1 | 0.483262729 | 1.90E-10 |
| CAPN10 | AC015982.1 | 0.323988205 | 3.91E-05 |
| CAPN2 | AC015982.1 | -0.343067663 | 1.24E-05 |
| DDIT3 | AC015982.1 | 0.351220767 | 7.43E-06 |
| ITGB4 | AC015982.1 | -0.304236923 | 0.000118747 |
| MAPK1 | AC015982.1 | -0.366441919 | 2.74E-06 |
| NFKB1 | AC015982.1 | -0.319034283 | 5.21E-05 |
| WDR45 | AC015982.1 | 0.420810203 | 4.98E-08 |
| BID | AC124248.1 | 0.407544526 | 1.42E-07 |
| ITGA3 | AC006001.2 | -0.347087436 | 9.66E-06 |
| NFE2L2 | AC006001.2 | 0.345952508 | 1.04E-05 |
| RHEB | AC006001.2 | 0.391845654 | 4.61E-07 |
| ATIC | RHPN1-AS1 | 0.36112383 | 3.91E-06 |
| GABARAPL2 | RHPN1-AS1 | 0.329037514 | 2.91E-05 |
| MAPK8IP1 | RHPN1-AS1 | 0.485651325 | 1.50E-10 |
| PRKAR1A | RHPN1-AS1 | 0.381099566 | 9.97E-07 |
| ST13 | RHPN1-AS1 | 0.337717727 | 1.73E-05 |
| ATG4A | TMPO-AS1 | 0.39759379 | 3.01E-07 |
| ATG4C | TMPO-AS1 | 0.349366697 | 8.37E-06 |
| ATIC | TMPO-AS1 | 0.577408051 | 3.70E-15 |
| BIRC5 | TMPO-AS1 | 0.587466176 | 9.36E-16 |
| CDKN1B | TMPO-AS1 | 0.309471576 | 8.92E-05 |
| HDAC6 | TMPO-AS1 | 0.485183759 | 1.57E-10 |
| MAPK8IP1 | TMPO-AS1 | 0.893459708 | 4.70E-55 |
| MTMR14 | TMPO-AS1 | 0.329263839 | 2.87E-05 |
| PRKAR1A | TMPO-AS1 | 0.670837307 | 1.30E-21 |
| ULK2 | TMPO-AS1 | 0.304970628 | 0.000114109 |
| VAMP7 | TMPO-AS1 | 0.472019905 | 5.64E-10 |
| ATG16L2 | AC092171.5 | 0.319281928 | 5.14E-05 |
| ATG4B | AC092171.5 | 0.371875319 | 1.89E-06 |
| ATG9B | AC092171.5 | 0.356010151 | 5.46E-06 |
| CAPNS1 | AC092171.5 | 0.306121078 | 0.000107177 |
| CLN3 | AC092171.5 | 0.317668566 | 5.63E-05 |
| ITGB1 | AC092171.5 | -0.309720814 | 8.79E-05 |
| MAP1LC3A | AC092171.5 | 0.302157976 | 0.000132861 |
| MTOR | AC092171.5 | -0.328398305 | 3.02E-05 |
| RAB24 | AC092171.5 | 0.316509793 | 6.01E-05 |
| RAC1 | AC092171.5 | 0.354561745 | 6.00E-06 |
| WDR45 | AC092171.5 | 0.417770834 | 6.36E-08 |
| ATG5 | AL035446.1 | 0.351813279 | 7.16E-06 |
| ITGA6 | AL035446.1 | 0.38474048 | 7.70E-07 |
| ITGB1 | AL035446.1 | 0.362389281 | 3.59E-06 |
| ATG16L2 | AC132872.3 | 0.344849784 | 1.11E-05 |
| CAPN10 | AC132872.3 | 0.319075287 | 5.20E-05 |
| CXCR4 | AC132872.3 | 0.330510449 | 2.67E-05 |
| HDAC6 | AC132872.3 | 0.300721145 | 0.000143514 |
| IKBKB | AC132872.3 | 0.383293246 | 8.54E-07 |
| VEGFA | AC132872.3 | 0.37834848 | 1.21E-06 |
| FKBP1B | LINC01694 | 0.562301489 | 2.67E-14 |
| FOXO1 | LINC01694 | -0.345577446 | 1.06E-05 |
| MYC | LINC01694 | 0.33982546 | 1.52E-05 |
| FADD | AP002387.1 | 0.3172499 | 5.77E-05 |
| ATG4D | AC100810.1 | 0.317686917 | 5.63E-05 |
| BAG1 | AC100810.1 | 0.341733128 | 1.35E-05 |
| BAX | AC100810.1 | 0.458937409 | 1.90E-09 |
| BIRC5 | AC100810.1 | 0.345890809 | 1.04E-05 |
| BNIP3L | AC100810.1 | 0.357157577 | 5.07E-06 |
| CDKN2A | AC100810.1 | 0.351251901 | 7.42E-06 |
| FKBP1A | AC100810.1 | 0.33325687 | 2.26E-05 |
| GABARAP | AC100810.1 | 0.3523503 | 6.92E-06 |
| ITGA3 | AC100810.1 | -0.329219603 | 2.88E-05 |
| ITGB4 | AC100810.1 | -0.325078049 | 3.67E-05 |
| MAP2K7 | AC100810.1 | 0.339130576 | 1.58E-05 |
| MTMR14 | AC100810.1 | 0.3489289 | 8.60E-06 |
| WDR45 | AC100810.1 | 0.410463174 | 1.13E-07 |
| ATG2B | BX322234.1 | 0.397731069 | 2.98E-07 |
| BAX | BX322234.1 | -0.301156592 | 0.000140204 |
| BNIP1 | BX322234.1 | -0.332053507 | 2.43E-05 |
| FOXO3 | BX322234.1 | 0.31010312 | 8.61E-05 |
| GABARAPL1 | BX322234.1 | 0.371775885 | 1.91E-06 |
| GOPC | BX322234.1 | 0.354340337 | 6.08E-06 |
| NCKAP1 | BX322234.1 | 0.47603516 | 3.84E-10 |
| NFE2L2 | BX322234.1 | 0.300136867 | 0.00014807 |
| PEX14 | BX322234.1 | -0.375806928 | 1.44E-06 |
| TM9SF1 | BX322234.1 | 0.389163403 | 5.60E-07 |
| TP63 | BX322234.1 | 0.38582258 | 7.13E-07 |
| UVRAG | BX322234.1 | 0.325869667 | 3.51E-05 |
| WIPI1 | BX322234.1 | 0.366273856 | 2.77E-06 |
| ATG10 | AC106820.3 | 0.328177481 | 3.06E-05 |
| ATG3 | AC106820.3 | 0.431212539 | 2.13E-08 |
| ATG4B | AC106820.3 | 0.496187581 | 5.19E-11 |
| ATG4D | AC106820.3 | 0.308056226 | 9.64E-05 |
| ATIC | AC106820.3 | 0.30655913 | 0.000104642 |
| BAX | AC106820.3 | 0.477768732 | 3.25E-10 |
| BIRC5 | AC106820.3 | 0.38828567 | 5.97E-07 |
| BNIP1 | AC106820.3 | 0.384286396 | 7.95E-07 |
| CAPN10 | AC106820.3 | 0.526787488 | 1.91E-12 |
| CAPN2 | AC106820.3 | -0.321278228 | 4.58E-05 |
| CDKN1B | AC106820.3 | 0.387341777 | 6.39E-07 |
| CDKN2A | AC106820.3 | 0.41596547 | 7.34E-08 |
| CLN3 | AC106820.3 | 0.392690452 | 4.33E-07 |
| CXCR4 | AC106820.3 | 0.442486169 | 8.18E-09 |
| GABARAP | AC106820.3 | 0.305034596 | 0.000113713 |
| HDAC6 | AC106820.3 | 0.447756507 | 5.17E-09 |
| ITGA3 | AC106820.3 | -0.344169314 | 1.16E-05 |
| ITGA6 | AC106820.3 | -0.311848224 | 7.81E-05 |
| ITGB1 | AC106820.3 | -0.300478937 | 0.000145387 |
| MAP2K7 | AC106820.3 | 0.403602727 | 1.92E-07 |
| MLST8 | AC106820.3 | 0.414566816 | 8.19E-08 |
| MTMR14 | AC106820.3 | 0.343607294 | 1.20E-05 |
| NRG1 | AC106820.3 | -0.300723732 | 0.000143494 |
| PELP1 | AC106820.3 | 0.3237424 | 3.97E-05 |
| PINK1 | AC106820.3 | -0.380207281 | 1.06E-06 |
| PRKAB1 | AC106820.3 | 0.335865402 | 1.93E-05 |
| PTK6 | AC106820.3 | -0.313293991 | 7.21E-05 |
| STK11 | AC106820.3 | 0.396715565 | 3.22E-07 |
| TP53 | AC106820.3 | 0.317117548 | 5.81E-05 |
| TP73 | AC106820.3 | 0.302174698 | 0.000132742 |
| TSC2 | AC106820.3 | 0.348206733 | 9.00E-06 |
| ULK3 | AC106820.3 | 0.35393594 | 6.25E-06 |
| WDR45 | AC106820.3 | 0.506944132 | 1.69E-11 |
| ATF4 | AC120053.1 | 0.382039373 | 9.33E-07 |
| ATG10 | AC120053.1 | 0.37826916 | 1.22E-06 |
| ATG12 | AC120053.1 | 0.37542021 | 1.48E-06 |
| ATG16L2 | AC120053.1 | 0.521459141 | 3.48E-12 |
| ATG4B | AC120053.1 | 0.517218284 | 5.56E-12 |
| ATG4C | AC120053.1 | 0.407942683 | 1.37E-07 |
| ATG4D | AC120053.1 | 0.374260135 | 1.61E-06 |
| BAG3 | AC120053.1 | -0.300412144 | 0.000145907 |
| BAX | AC120053.1 | 0.42780808 | 2.82E-08 |
| BNIP1 | AC120053.1 | 0.380206978 | 1.06E-06 |
| CAPN10 | AC120053.1 | 0.645416487 | 1.24E-19 |
| CDKN1B | AC120053.1 | 0.345501672 | 1.07E-05 |
| CXCR4 | AC120053.1 | 0.483897995 | 1.79E-10 |
| DAPK2 | AC120053.1 | 0.402683086 | 2.05E-07 |
| EIF4G1 | AC120053.1 | -0.454460279 | 2.85E-09 |
| GABARAP | AC120053.1 | 0.347964493 | 9.14E-06 |
| HDAC6 | AC120053.1 | 0.337820108 | 1.72E-05 |
| HIF1A | AC120053.1 | -0.326104079 | 3.46E-05 |
| IKBKB | AC120053.1 | 0.331626644 | 2.50E-05 |
| KIF5B | AC120053.1 | -0.3366041 | 1.85E-05 |
| MAP1LC3A | AC120053.1 | 0.334812688 | 2.06E-05 |
| MAP2K7 | AC120053.1 | 0.383900218 | 8.18E-07 |
| MAPK8 | AC120053.1 | 0.343926216 | 1.18E-05 |
| MLST8 | AC120053.1 | 0.306818697 | 0.000103166 |
| MTMR14 | AC120053.1 | 0.318305919 | 5.43E-05 |
| NCKAP1 | AC120053.1 | -0.36882226 | 2.33E-06 |
| NKX2-3 | AC120053.1 | 0.328554206 | 3.00E-05 |
| PEX14 | AC120053.1 | 0.479820508 | 2.66E-10 |
| RAB24 | AC120053.1 | 0.398113529 | 2.90E-07 |
| STK11 | AC120053.1 | 0.464299929 | 1.16E-09 |
| TP73 | AC120053.1 | 0.366505273 | 2.73E-06 |
| TSC2 | AC120053.1 | 0.403457784 | 1.94E-07 |
| ULK3 | AC120053.1 | 0.323695401 | 3.98E-05 |
| USP10 | AC120053.1 | -0.350857182 | 7.61E-06 |
| WDFY3 | AC120053.1 | -0.339782658 | 1.52E-05 |
| WDR45 | AC120053.1 | 0.413706764 | 8.77E-08 |
| ATG4B | AC080037.1 | 0.380404074 | 1.05E-06 |
| ATG4D | AC080037.1 | 0.304297066 | 0.00011836 |
| BID | AC080037.1 | 0.305861118 | 0.000108708 |
| CAPN10 | AC080037.1 | 0.343116034 | 1.24E-05 |
| CDKN2A | AC080037.1 | 0.32943505 | 2.84E-05 |
| MAPK8 | AC080037.1 | 0.307540095 | 9.92E-05 |
| PRKAB1 | AC080037.1 | 0.334972468 | 2.04E-05 |
| ATG10 | LINC00839 | 0.301047683 | 0.000141026 |
| ATG2A | LINC00839 | 0.355723792 | 5.56E-06 |
| ATG4C | LINC00839 | 0.325329026 | 3.62E-05 |
| ATG7 | LINC00839 | 0.391922025 | 4.58E-07 |
| BCL2 | LINC00839 | 0.55548152 | 6.30E-14 |
| CDKN1B | LINC00839 | 0.322658914 | 4.23E-05 |
| CFLAR | LINC00839 | 0.312799333 | 7.41E-05 |
| CXCR4 | LINC00839 | 0.380324164 | 1.05E-06 |
| DRAM1 | LINC00839 | 0.32420792 | 3.87E-05 |
| EEF2K | LINC00839 | 0.301663551 | 0.000136441 |
| FAS | LINC00839 | 0.546722719 | 1.85E-13 |
| IKBKE | LINC00839 | 0.478625982 | 2.99E-10 |
| MAPK8 | LINC00839 | 0.465923356 | 1.00E-09 |
| MTMR14 | LINC00839 | 0.304140945 | 0.000119366 |
| NKX2-3 | LINC00839 | 0.57185973 | 7.73E-15 |
| PARP1 | LINC00839 | 0.382752055 | 8.87E-07 |
| PIK3R4 | LINC00839 | 0.319205354 | 5.16E-05 |
| PRKAB1 | LINC00839 | 0.409058988 | 1.26E-07 |
| RAF1 | LINC00839 | 0.305826635 | 0.000108912 |
| TP53 | LINC00839 | 0.414422188 | 8.29E-08 |
| TP73 | LINC00839 | 0.602401531 | 1.11E-16 |
| TSC2 | LINC00839 | 0.646505589 | 1.03E-19 |
| WDR45 | LINC00839 | 0.304049997 | 0.000119956 |
| TSC1 | AL355574.1 | 0.435670721 | 1.46E-08 |
| DIRAS3 | AC092916.1 | 0.523996653 | 2.62E-12 |
| FKBP1B | AC092916.1 | 0.400187288 | 2.48E-07 |
| FOXO1 | AC092916.1 | -0.316398435 | 6.05E-05 |
| PRKCQ | AC092916.1 | 0.397918295 | 2.94E-07 |
| BID | SNHG18 | 0.337941918 | 1.70E-05 |
| GABARAPL2 | SNHG18 | 0.335244722 | 2.01E-05 |
| ATIC | UBAC2-AS1 | 0.30595433 | 0.000108156 |
| FKBP1B | UBAC2-AS1 | 0.43515112 | 1.53E-08 |
| GAPDH | UBAC2-AS1 | 0.323590952 | 4.01E-05 |
| PINK1 | UBAC2-AS1 | -0.30971491 | 8.80E-05 |
| ATG4D | AL135818.2 | -0.374197857 | 1.62E-06 |
| BAK1 | AL135818.2 | 0.335162707 | 2.02E-05 |
| BCL2 | AL135818.2 | -0.302689014 | 0.000129114 |
| CDKN1B | AL135818.2 | -0.32901932 | 2.91E-05 |
| CLN3 | AL135818.2 | -0.301991269 | 0.000134058 |
| EIF2AK3 | AL135818.2 | -0.30033944 | 0.000146475 |
| IKBKB | AL135818.2 | -0.308730141 | 9.29E-05 |
| IL24 | AL135818.2 | 0.364196143 | 3.19E-06 |
| ITGA3 | AL135818.2 | 0.567466143 | 1.37E-14 |
| ITGB4 | AL135818.2 | 0.365466474 | 2.93E-06 |
| PINK1 | AL135818.2 | 0.439621869 | 1.05E-08 |
| PRKAB1 | AL135818.2 | -0.362697197 | 3.52E-06 |
| BAK1 | DNAJC3-DT | -0.308991865 | 9.16E-05 |
| BIRC6 | DNAJC3-DT | 0.306512158 | 0.000104911 |
| EEF2 | DNAJC3-DT | 0.31630348 | 6.08E-05 |
| EIF2AK3 | DNAJC3-DT | 0.401663361 | 2.22E-07 |
| ERN1 | DNAJC3-DT | 0.327982879 | 3.10E-05 |
| GABARAPL1 | DNAJC3-DT | 0.363541613 | 3.33E-06 |
| ITGA3 | DNAJC3-DT | -0.318657573 | 5.32E-05 |
| KLHL24 | DNAJC3-DT | 0.34322778 | 1.23E-05 |
| NBR1 | DNAJC3-DT | 0.39691372 | 3.17E-07 |
| NCKAP1 | DNAJC3-DT | 0.350989478 | 7.54E-06 |
| NFE2L2 | DNAJC3-DT | 0.303030819 | 0.000126755 |
| PRKAR1A | DNAJC3-DT | 0.308339818 | 9.49E-05 |
| RB1CC1 | DNAJC3-DT | 0.371769506 | 1.91E-06 |
| SIRT1 | DNAJC3-DT | 0.323114745 | 4.12E-05 |
| ST13 | DNAJC3-DT | 0.323222654 | 4.09E-05 |
| UVRAG | DNAJC3-DT | 0.41059147 | 1.12E-07 |
| VAMP7 | DNAJC3-DT | 0.31202554 | 7.74E-05 |
| WIPI1 | DNAJC3-DT | 0.416228944 | 7.18E-08 |
| GNAI3 | LINC01234 | -0.35248966 | 6.85E-06 |
| ATF4 | AC131009.3 | 0.325937493 | 3.49E-05 |
| BAG3 | AC131009.3 | -0.314658664 | 6.68E-05 |
| BAX | AC131009.3 | 0.329320302 | 2.86E-05 |
| BNIP1 | AC131009.3 | 0.37805078 | 1.24E-06 |
| CAPN2 | AC131009.3 | -0.301845688 | 0.000135112 |
| DDIT3 | AC131009.3 | 0.548844334 | 1.43E-13 |
| MAPK1 | AC131009.3 | -0.321727618 | 4.46E-05 |
| PELP1 | AC131009.3 | 0.338944811 | 1.60E-05 |
| PTEN | AC131009.3 | -0.302056138 | 0.000133591 |
| SH3GLB1 | AC131009.3 | -0.32516771 | 3.65E-05 |
| ATG10 | AC004943.2 | 0.322166116 | 4.35E-05 |
| ATG4A | AC004943.2 | 0.324210232 | 3.86E-05 |
| USP10 | AC004943.2 | 0.315246627 | 6.46E-05 |
| VAMP7 | AC004943.2 | 0.305873085 | 0.000108637 |
| ATG10 | AC024075.2 | 0.300213552 | 0.000147464 |
| ATG12 | AC024075.2 | 0.313166764 | 7.26E-05 |
| ATG16L2 | AC024075.2 | 0.423572469 | 3.99E-08 |
| ATG4B | AC024075.2 | 0.524100152 | 2.59E-12 |
| ATG4C | AC024075.2 | 0.341589022 | 1.36E-05 |
| ATG4D | AC024075.2 | 0.387534323 | 6.30E-07 |
| BAX | AC024075.2 | 0.316718667 | 5.94E-05 |
| BCL2 | AC024075.2 | 0.305557472 | 0.000110522 |
| CAPN10 | AC024075.2 | 0.563853768 | 2.19E-14 |
| CDKN1B | AC024075.2 | 0.321966337 | 4.40E-05 |
| CFLAR | AC024075.2 | 0.351875277 | 7.13E-06 |
| CX3CL1 | AC024075.2 | 0.327740493 | 3.14E-05 |
| CXCR4 | AC024075.2 | 0.645641087 | 1.19E-19 |
| DAPK2 | AC024075.2 | 0.431023713 | 2.16E-08 |
| HDAC6 | AC024075.2 | 0.426421781 | 3.16E-08 |
| HSPA5 | AC024075.2 | -0.335265104 | 2.01E-05 |
| IKBKB | AC024075.2 | 0.410976479 | 1.09E-07 |
| IKBKE | AC024075.2 | 0.326135014 | 3.45E-05 |
| ITGA3 | AC024075.2 | -0.307481236 | 9.95E-05 |
| MAP2K7 | AC024075.2 | 0.481325111 | 2.30E-10 |
| MTMR14 | AC024075.2 | 0.398677064 | 2.78E-07 |
| NKX2-3 | AC024075.2 | 0.36029877 | 4.12E-06 |
| PINK1 | AC024075.2 | -0.313638533 | 7.07E-05 |
| PRKAB1 | AC024075.2 | 0.3461652 | 1.02E-05 |
| RAF1 | AC024075.2 | 0.371372316 | 1.96E-06 |
| STK11 | AC024075.2 | 0.45488055 | 2.75E-09 |
| TP53 | AC024075.2 | 0.386869785 | 6.61E-07 |
| TP73 | AC024075.2 | 0.436435925 | 1.37E-08 |
| TSC2 | AC024075.2 | 0.561194073 | 3.07E-14 |
| ULK3 | AC024075.2 | 0.405819912 | 1.62E-07 |
| WDR45 | AC024075.2 | 0.376290772 | 1.40E-06 |
| EIF2AK3 | AC007098.1 | 0.366948043 | 2.65E-06 |
| FOXO3 | AC007098.1 | 0.319766184 | 5.00E-05 |
| GABARAPL1 | AC007098.1 | 0.430285986 | 2.30E-08 |
| ITGA3 | AC007098.1 | -0.303126767 | 0.0001261 |
| NFE2L2 | AC007098.1 | 0.537869136 | 5.31E-13 |
| WIPI1 | AC007098.1 | 0.367300434 | 2.59E-06 |
| AMBRA1 | LINC01023 | -0.31289949 | 7.37E-05 |
| ARNT | LINC01023 | -0.300870571 | 0.00014237 |
| ATF4 | LINC01023 | 0.322229956 | 4.33E-05 |
| ATG4B | LINC01023 | 0.384198476 | 8.00E-07 |
| BAX | LINC01023 | 0.482822403 | 1.99E-10 |
| BIRC5 | LINC01023 | 0.331575065 | 2.50E-05 |
| BIRC6 | LINC01023 | -0.362003173 | 3.69E-06 |
| BNIP1 | LINC01023 | 0.47654854 | 3.66E-10 |
| CANX | LINC01023 | -0.31041618 | 8.46E-05 |
| CAPN10 | LINC01023 | 0.345771599 | 1.05E-05 |
| CAPN2 | LINC01023 | -0.307911709 | 9.72E-05 |
| CDKN2A | LINC01023 | 0.307182224 | 0.000101133 |
| CLN3 | LINC01023 | 0.353016958 | 6.63E-06 |
| FKBP1A | LINC01023 | 0.339173307 | 1.58E-05 |
| GABARAPL2 | LINC01023 | 0.370503806 | 2.08E-06 |
| GOPC | LINC01023 | -0.425164865 | 3.50E-08 |
| HGS | LINC01023 | -0.300142365 | 0.000148026 |
| ITGA6 | LINC01023 | -0.369077052 | 2.29E-06 |
| ITGB1 | LINC01023 | -0.361212837 | 3.88E-06 |
| ITGB4 | LINC01023 | -0.300735935 | 0.000143401 |
| KIF5B | LINC01023 | -0.331697286 | 2.48E-05 |
| MAP1LC3A | LINC01023 | 0.332556219 | 2.36E-05 |
| MAPK1 | LINC01023 | -0.465069765 | 1.08E-09 |
| MTMR14 | LINC01023 | 0.308238964 | 9.54E-05 |
| NCKAP1 | LINC01023 | -0.332788848 | 2.33E-05 |
| NPC1 | LINC01023 | -0.344597389 | 1.13E-05 |
| PEX14 | LINC01023 | 0.339463878 | 1.55E-05 |
| RAB24 | LINC01023 | 0.460046973 | 1.72E-09 |
| RB1 | LINC01023 | -0.344114877 | 1.16E-05 |
| WDFY3 | LINC01023 | -0.336035565 | 1.91E-05 |
| WDR45 | LINC01023 | 0.461717062 | 1.48E-09 |
| ATG10 | ZNF436-AS1 | 0.315864615 | 6.24E-05 |
| ATG12 | ZNF436-AS1 | 0.377063253 | 1.32E-06 |
| ATG16L2 | ZNF436-AS1 | 0.403368427 | 1.95E-07 |
| ATG4B | ZNF436-AS1 | 0.312112118 | 7.70E-05 |
| ATG4C | ZNF436-AS1 | 0.509084556 | 1.34E-11 |
| BCL2 | ZNF436-AS1 | 0.438157916 | 1.19E-08 |
| CAPN10 | ZNF436-AS1 | 0.500539677 | 3.31E-11 |
| CD46 | ZNF436-AS1 | 0.306890603 | 0.000102761 |
| CDKN1A | ZNF436-AS1 | -0.335944154 | 1.92E-05 |
| CDKN1B | ZNF436-AS1 | 0.485435037 | 1.53E-10 |
| CXCR4 | ZNF436-AS1 | 0.471034533 | 6.19E-10 |
| DAPK2 | ZNF436-AS1 | 0.377816228 | 1.26E-06 |
| DRAM1 | ZNF436-AS1 | 0.364413603 | 3.14E-06 |
| EEF2K | ZNF436-AS1 | 0.354517185 | 6.02E-06 |
| HDAC6 | ZNF436-AS1 | 0.581576181 | 2.10E-15 |
| HSPA5 | ZNF436-AS1 | -0.320515763 | 4.79E-05 |
| IKBKB | ZNF436-AS1 | 0.490869225 | 8.91E-11 |
| IKBKE | ZNF436-AS1 | 0.322705045 | 4.22E-05 |
| ITGA3 | ZNF436-AS1 | -0.319645019 | 5.03E-05 |
| MAP2K7 | ZNF436-AS1 | 0.456071201 | 2.47E-09 |
| MAPK8 | ZNF436-AS1 | 0.42741869 | 2.91E-08 |
| MAPK9 | ZNF436-AS1 | 0.358678378 | 4.59E-06 |
| MLST8 | ZNF436-AS1 | 0.302379189 | 0.000131288 |
| MTOR | ZNF436-AS1 | 0.319354776 | 5.11E-05 |
| NKX2-3 | ZNF436-AS1 | 0.413008402 | 9.26E-08 |
| PARP1 | ZNF436-AS1 | 0.425538921 | 3.40E-08 |
| PRKAB1 | ZNF436-AS1 | 0.436255158 | 1.39E-08 |
| PTK6 | ZNF436-AS1 | -0.319558337 | 5.06E-05 |
| RAF1 | ZNF436-AS1 | 0.303921036 | 0.000120797 |
| SIRT1 | ZNF436-AS1 | 0.36583104 | 2.85E-06 |
| STK11 | ZNF436-AS1 | 0.364768733 | 3.07E-06 |
| TP53 | ZNF436-AS1 | 0.406348684 | 1.55E-07 |
| TP73 | ZNF436-AS1 | 0.467473963 | 8.66E-10 |
| TSC1 | ZNF436-AS1 | 0.327113983 | 3.26E-05 |
| TSC2 | ZNF436-AS1 | 0.558174112 | 4.50E-14 |
| ULK2 | ZNF436-AS1 | 0.358421556 | 4.67E-06 |
| ULK3 | ZNF436-AS1 | 0.462691952 | 1.35E-09 |
| UVRAG | ZNF436-AS1 | 0.365882292 | 2.85E-06 |
| WDR45 | ZNF436-AS1 | 0.349938954 | 8.07E-06 |
| IL24 | AC113346.1 | 0.625771419 | 3.16E-18 |
| AMBRA1 | FLJ20021 | -0.351552764 | 7.28E-06 |
| ARNT | FLJ20021 | -0.362979603 | 3.45E-06 |
| ARSB | FLJ20021 | -0.394987794 | 3.65E-07 |
| ATF6 | FLJ20021 | -0.326626426 | 3.36E-05 |
| BIRC6 | FLJ20021 | -0.479883425 | 2.65E-10 |
| CANX | FLJ20021 | -0.433970376 | 1.69E-08 |
| CD46 | FLJ20021 | -0.328064955 | 3.08E-05 |
| DAPK1 | FLJ20021 | -0.327238243 | 3.24E-05 |
| EEF2 | FLJ20021 | -0.327043716 | 3.27E-05 |
| EEF2K | FLJ20021 | -0.303771187 | 0.000121781 |
| EIF2AK2 | FLJ20021 | -0.307034003 | 0.000101957 |
| EIF2AK3 | FLJ20021 | -0.346323345 | 1.01E-05 |
| FOXO1 | FLJ20021 | -0.361106881 | 3.91E-06 |
| GABARAPL2 | FLJ20021 | 0.345551212 | 1.06E-05 |
| ITPR1 | FLJ20021 | -0.364676821 | 3.08E-06 |
| KIF5B | FLJ20021 | -0.31373723 | 7.03E-05 |
| KLHL24 | FLJ20021 | -0.359462405 | 4.36E-06 |
| LAMP2 | FLJ20021 | -0.319402537 | 5.10E-05 |
| MAPK1 | FLJ20021 | -0.351833237 | 7.15E-06 |
| MAPK9 | FLJ20021 | -0.341317523 | 1.39E-05 |
| MBTPS2 | FLJ20021 | -0.343489966 | 1.21E-05 |
| MTOR | FLJ20021 | -0.376436477 | 1.38E-06 |
| NBR1 | FLJ20021 | -0.423980986 | 3.86E-08 |
| NCKAP1 | FLJ20021 | -0.333449765 | 2.24E-05 |
| PARP1 | FLJ20021 | -0.353825156 | 6.29E-06 |
| PIK3R4 | FLJ20021 | -0.384722613 | 7.71E-07 |
| PTK6 | FLJ20021 | 0.318069136 | 5.50E-05 |
| RB1CC1 | FLJ20021 | -0.343960268 | 1.18E-05 |
| SIRT1 | FLJ20021 | -0.385752584 | 7.16E-07 |
| SPNS1 | FLJ20021 | -0.340889879 | 1.42E-05 |
| ULK2 | FLJ20021 | -0.329268198 | 2.87E-05 |
| VAMP7 | FLJ20021 | -0.315723048 | 6.29E-05 |
| EIF4G1 | LINC02041 | 0.382596543 | 8.97E-07 |
| ATG10 | AL157394.1 | 0.313591311 | 7.09E-05 |
| ATG12 | AL157394.1 | 0.35609812 | 5.43E-06 |
| ATG2A | AL157394.1 | 0.321153006 | 4.61E-05 |
| ATG4A | AL157394.1 | 0.325316072 | 3.62E-05 |
| ATG4C | AL157394.1 | 0.427811464 | 2.82E-08 |
| ATG7 | AL157394.1 | 0.396423725 | 3.29E-07 |
| BCL2 | AL157394.1 | 0.503163819 | 2.52E-11 |
| BNIP1 | AL157394.1 | 0.315863922 | 6.24E-05 |
| CALCOCO2 | AL157394.1 | 0.338585094 | 1.64E-05 |
| CASP3 | AL157394.1 | 0.318136469 | 5.48E-05 |
| CDKN1B | AL157394.1 | 0.36281775 | 3.49E-06 |
| CFLAR | AL157394.1 | 0.428449331 | 2.67E-08 |
| CX3CL1 | AL157394.1 | 0.367944285 | 2.48E-06 |
| CXCR4 | AL157394.1 | 0.437482868 | 1.26E-08 |
| DRAM1 | AL157394.1 | 0.434303452 | 1.64E-08 |
| FAS | AL157394.1 | 0.853372247 | 3.90E-45 |
| HDAC6 | AL157394.1 | 0.405929409 | 1.60E-07 |
| HIF1A | AL157394.1 | -0.307521246 | 9.93E-05 |
| IKBKE | AL157394.1 | 0.459039019 | 1.89E-09 |
| MAPK8 | AL157394.1 | 0.537114166 | 5.81E-13 |
| MAPK9 | AL157394.1 | 0.405288124 | 1.68E-07 |
| MTMR14 | AL157394.1 | 0.33922816 | 1.58E-05 |
| NKX2-3 | AL157394.1 | 0.670869 | 1.30E-21 |
| PARP1 | AL157394.1 | 0.444463743 | 6.89E-09 |
| PEX14 | AL157394.1 | 0.311662927 | 7.90E-05 |
| PRKAB1 | AL157394.1 | 0.336356475 | 1.88E-05 |
| PTEN | AL157394.1 | 0.351857967 | 7.14E-06 |
| RAB33B | AL157394.1 | 0.318017572 | 5.52E-05 |
| RAF1 | AL157394.1 | 0.300390123 | 0.000146079 |
| SIRT1 | AL157394.1 | 0.351154382 | 7.47E-06 |
| TP53 | AL157394.1 | 0.429804377 | 2.39E-08 |
| TP73 | AL157394.1 | 0.583050971 | 1.72E-15 |
| TSC2 | AL157394.1 | 0.565849985 | 1.69E-14 |
| WDR45 | AL157394.1 | 0.419747737 | 5.43E-08 |
| ATG16L2 | AC138207.4 | 0.348127209 | 9.05E-06 |
| RAB1A | AC138207.4 | 0.324024836 | 3.91E-05 |
| GAPDH | LINC01816 | 0.359018703 | 4.49E-06 |
| NFKB1 | LINC01816 | -0.33527525 | 2.00E-05 |
| SH3GLB1 | LINC01816 | -0.346093435 | 1.03E-05 |
| VAMP3 | LINC01816 | -0.328608758 | 2.99E-05 |
| PELP1 | CYP4F26P | 0.373997582 | 1.64E-06 |
| IL24 | AC138028.4 | 0.34616 | 1.02E-05 |
| ATF4 | ASMTL-AS1 | 0.306363196 | 0.000105769 |
| ATG12 | ASMTL-AS1 | 0.341965322 | 1.33E-05 |
| ATG16L2 | ASMTL-AS1 | 0.485418991 | 1.54E-10 |
| ATG4B | ASMTL-AS1 | 0.50359866 | 2.40E-11 |
| ATG4C | ASMTL-AS1 | 0.307851549 | 9.75E-05 |
| BAX | ASMTL-AS1 | 0.387858378 | 6.15E-07 |
| BNIP1 | ASMTL-AS1 | 0.387839959 | 6.16E-07 |
| CAPN10 | ASMTL-AS1 | 0.614450078 | 1.84E-17 |
| CLN3 | ASMTL-AS1 | 0.365153749 | 2.99E-06 |
| CXCR4 | ASMTL-AS1 | 0.330710839 | 2.64E-05 |
| EIF4G1 | ASMTL-AS1 | -0.324377596 | 3.83E-05 |
| HDAC6 | ASMTL-AS1 | 0.338484695 | 1.65E-05 |
| IKBKB | ASMTL-AS1 | 0.345222928 | 1.09E-05 |
| KIF5B | ASMTL-AS1 | -0.353634576 | 6.37E-06 |
| MAP1LC3A | ASMTL-AS1 | 0.347177783 | 9.61E-06 |
| MAPK1 | ASMTL-AS1 | -0.320337704 | 4.83E-05 |
| MAPK8IP1 | ASMTL-AS1 | 0.308635794 | 9.34E-05 |
| MLST8 | ASMTL-AS1 | 0.342959861 | 1.25E-05 |
| MTMR14 | ASMTL-AS1 | 0.337368795 | 1.76E-05 |
| NCKAP1 | ASMTL-AS1 | -0.336132036 | 1.90E-05 |
| PEX14 | ASMTL-AS1 | 0.359710155 | 4.29E-06 |
| RAB24 | ASMTL-AS1 | 0.459866849 | 1.75E-09 |
| STK11 | ASMTL-AS1 | 0.433943156 | 1.69E-08 |
| TP63 | ASMTL-AS1 | -0.316702875 | 5.95E-05 |
| ULK3 | ASMTL-AS1 | 0.418601234 | 5.95E-08 |
| USP10 | ASMTL-AS1 | -0.333719592 | 2.20E-05 |
| WDR45 | ASMTL-AS1 | 0.472388912 | 5.45E-10 |
| PRKCD | AC091729.1 | -0.302394521 | 0.00013118 |
| RAC1 | AC091729.1 | 0.368647803 | 2.36E-06 |
| ATG3 | HAGLROS | 0.374449569 | 1.59E-06 |
| ATG4A | HAGLROS | 0.367596621 | 2.53E-06 |
| ATIC | HAGLROS | 0.363925195 | 3.24E-06 |
| BIRC5 | HAGLROS | 0.311797599 | 7.84E-05 |
| HDAC6 | HAGLROS | 0.452121503 | 3.51E-09 |
| MAPK8IP1 | HAGLROS | 0.537963644 | 5.26E-13 |
| PIK3C3 | HAGLROS | 0.321449025 | 4.53E-05 |
| PRKAR1A | HAGLROS | 0.40285972 | 2.03E-07 |
| VAMP7 | HAGLROS | 0.383079194 | 8.67E-07 |
| GABARAP | AC015660.1 | -0.305402229 | 0.000111461 |
| HIF1A | AC015660.1 | 0.341088589 | 1.40E-05 |
| RPTOR | AC015660.1 | 0.3038616 | 0.000121187 |
| EIF2AK3 | LINC00667 | 0.329457093 | 2.84E-05 |
| NPC1 | LINC00667 | 0.300170301 | 0.000147806 |
| ATG4D | LINC01932 | 0.312181697 | 7.67E-05 |
| BECN1 | LINC01932 | 0.300079988 | 0.000148521 |
| GABARAPL1 | LINC01932 | 0.356277062 | 5.37E-06 |
| NCKAP1 | LINC01932 | 0.307941432 | 9.70E-05 |
| NFE2L2 | LINC01932 | 0.36523976 | 2.97E-06 |
| RB1CC1 | LINC01932 | 0.327285441 | 3.23E-05 |
| RPS6KB1 | LINC01932 | 0.300212212 | 0.000147475 |
| WIPI1 | LINC01932 | 0.342772997 | 1.27E-05 |
| BAX | URB1-AS1 | 0.301572104 | 0.000137113 |
| BNIP1 | URB1-AS1 | 0.351337587 | 7.38E-06 |
| CLN3 | URB1-AS1 | 0.342653342 | 1.28E-05 |
| GABARAPL2 | URB1-AS1 | 0.339931814 | 1.51E-05 |
| ITGA3 | URB1-AS1 | -0.315290774 | 6.44E-05 |
| ITGB4 | URB1-AS1 | -0.343679398 | 1.20E-05 |
| NFKB1 | URB1-AS1 | -0.346067712 | 1.03E-05 |
| WDR45 | URB1-AS1 | 0.345234307 | 1.09E-05 |
| BAK1 | MIR31HG | 0.319797518 | 4.99E-05 |
| CASP4 | MIR31HG | 0.339564438 | 1.54E-05 |
| CDKN1B | MIR31HG | -0.352064981 | 7.04E-06 |
| CLN3 | MIR31HG | -0.366410135 | 2.75E-06 |
| EIF2AK3 | MIR31HG | -0.325992429 | 3.48E-05 |
| ITGA3 | MIR31HG | 0.419917274 | 5.35E-08 |
| ITGA6 | MIR31HG | 0.339304161 | 1.57E-05 |
| ITGB4 | MIR31HG | 0.348708774 | 8.72E-06 |
| KLHL24 | MIR31HG | -0.333317394 | 2.26E-05 |
| MAP2K7 | MIR31HG | -0.312683583 | 7.46E-05 |
| NRG1 | MIR31HG | 0.343015673 | 1.25E-05 |
| PARP1 | MIR31HG | -0.360967909 | 3.95E-06 |
| PELP1 | MIR31HG | -0.301402054 | 0.00013837 |
| PINK1 | MIR31HG | 0.453444035 | 3.12E-09 |
| PRKAB1 | MIR31HG | -0.301018805 | 0.000141244 |
| PTK6 | MIR31HG | 0.390849612 | 4.95E-07 |
| RHEB | MIR31HG | -0.309236744 | 9.03E-05 |
| ATG10 | AC093726.1 | 0.362113398 | 3.66E-06 |
| ATG16L2 | AC093726.1 | 0.310926274 | 8.23E-05 |
| ATG4A | AC093726.1 | 0.357284919 | 5.03E-06 |
| ATG4C | AC093726.1 | 0.490735313 | 9.04E-11 |
| BCL2 | AC093726.1 | 0.377143574 | 1.32E-06 |
| CAPN10 | AC093726.1 | 0.333291267 | 2.26E-05 |
| CASP3 | AC093726.1 | 0.302335116 | 0.0001316 |
| CXCR4 | AC093726.1 | 0.316227512 | 6.11E-05 |
| FAS | AC093726.1 | 0.30071909 | 0.00014353 |
| HDAC6 | AC093726.1 | 0.563899383 | 2.17E-14 |
| MAPK9 | AC093726.1 | 0.312944044 | 7.35E-05 |
| MTMR14 | AC093726.1 | 0.309296936 | 9.00E-05 |
| PRKAR1A | AC093726.1 | 0.334822539 | 2.06E-05 |
| RHEB | AC093726.1 | 0.324575902 | 3.78E-05 |
| TP73 | AC093726.1 | 0.352161678 | 7.00E-06 |
| TSC2 | AC093726.1 | 0.386092252 | 6.99E-07 |
| ULK3 | AC093726.1 | 0.360901645 | 3.96E-06 |
| WDR45 | AC093726.1 | 0.342928932 | 1.25E-05 |
| ARNT | ZFAS1 | -0.360526784 | 4.06E-06 |
| ATF4 | ZFAS1 | 0.389286669 | 5.55E-07 |
| ATG4B | ZFAS1 | 0.408167343 | 1.35E-07 |
| BAG1 | ZFAS1 | 0.355558868 | 5.62E-06 |
| BAX | ZFAS1 | 0.497990576 | 4.31E-11 |
| BIRC5 | ZFAS1 | 0.421426775 | 4.74E-08 |
| BIRC6 | ZFAS1 | -0.336763055 | 1.83E-05 |
| BNIP1 | ZFAS1 | 0.398796936 | 2.75E-07 |
| CAPN10 | ZFAS1 | 0.379124548 | 1.15E-06 |
| CAPN2 | ZFAS1 | -0.300218253 | 0.000147427 |
| EIF4EBP1 | ZFAS1 | 0.375461141 | 1.48E-06 |
| EIF4G1 | ZFAS1 | -0.305602004 | 0.000110254 |
| FKBP1A | ZFAS1 | 0.394924141 | 3.67E-07 |
| FOXO1 | ZFAS1 | -0.307010623 | 0.000102088 |
| KIF5B | ZFAS1 | -0.301174352 | 0.000140071 |
| MAPK1 | ZFAS1 | -0.445584076 | 6.25E-09 |
| NCKAP1 | ZFAS1 | -0.308603811 | 9.35E-05 |
| PIK3R4 | ZFAS1 | -0.337185513 | 1.78E-05 |
| RB1 | ZFAS1 | -0.303832743 | 0.000121376 |
| TP63 | ZFAS1 | -0.311600134 | 7.92E-05 |
| WDR45 | ZFAS1 | 0.389916199 | 5.30E-07 |
| ATG7 | AC130371.2 | 0.334825286 | 2.06E-05 |
| HSP90AB1 | AC130371.2 | -0.351207434 | 7.44E-06 |
| ST13 | AC130371.2 | -0.311475488 | 7.98E-05 |
| ATF4 | AL136295.7 | 0.33738531 | 1.76E-05 |
| ATG10 | AL136295.7 | 0.34384231 | 1.18E-05 |
| ATG16L2 | AL136295.7 | 0.460671679 | 1.62E-09 |
| ATG3 | AL136295.7 | 0.351647719 | 7.23E-06 |
| ATG4B | AL136295.7 | 0.566400369 | 1.58E-14 |
| ATG4C | AL136295.7 | 0.312888913 | 7.37E-05 |
| BAG3 | AL136295.7 | -0.324633098 | 3.77E-05 |
| BAX | AL136295.7 | 0.50207943 | 2.82E-11 |
| BNIP1 | AL136295.7 | 0.393977849 | 3.94E-07 |
| CAPN10 | AL136295.7 | 0.602134251 | 1.16E-16 |
| CDKN2A | AL136295.7 | 0.43481886 | 1.57E-08 |
| CLN3 | AL136295.7 | 0.374045179 | 1.63E-06 |
| CXCR4 | AL136295.7 | 0.438446575 | 1.16E-08 |
| DAPK2 | AL136295.7 | 0.383456068 | 8.44E-07 |
| DLC1 | AL136295.7 | -0.309399473 | 8.95E-05 |
| HDAC6 | AL136295.7 | 0.335203379 | 2.01E-05 |
| HIF1A | AL136295.7 | -0.330891763 | 2.61E-05 |
| IKBKB | AL136295.7 | 0.372608492 | 1.80E-06 |
| ITGA6 | AL136295.7 | -0.306477807 | 0.000105108 |
| ITGB1 | AL136295.7 | -0.300929045 | 0.000141925 |
| KIF5B | AL136295.7 | -0.366100465 | 2.80E-06 |
| MAP2K7 | AL136295.7 | 0.378572875 | 1.19E-06 |
| MTMR14 | AL136295.7 | 0.416044056 | 7.29E-08 |
| NCKAP1 | AL136295.7 | -0.387468841 | 6.33E-07 |
| PELP1 | AL136295.7 | 0.304480123 | 0.00011719 |
| PEX14 | AL136295.7 | 0.402314593 | 2.11E-07 |
| RAB24 | AL136295.7 | 0.427557991 | 2.88E-08 |
| STK11 | AL136295.7 | 0.44451902 | 6.86E-09 |
| TSC2 | AL136295.7 | 0.360975014 | 3.94E-06 |
| ULK3 | AL136295.7 | 0.379547431 | 1.11E-06 |
| WDFY3 | AL136295.7 | -0.384310345 | 7.94E-07 |
| WDR45 | AL136295.7 | 0.50261915 | 2.66E-11 |
| ATG7 | SERPINB9P1 | 0.314770837 | 6.63E-05 |
| CHMP2B | SERPINB9P1 | 0.314482358 | 6.74E-05 |
| CXCR4 | SERPINB9P1 | 0.339493093 | 1.55E-05 |
| FAS | SERPINB9P1 | 0.341637681 | 1.36E-05 |
| ITPR1 | SERPINB9P1 | 0.37019469 | 2.13E-06 |
| RGS19 | SERPINB9P1 | 0.378638125 | 1.19E-06 |
| SH3GLB1 | SERPINB9P1 | 0.333782753 | 2.19E-05 |
| RB1 | LINC00662 | -0.343723578 | 1.19E-05 |
| ATG12 | AL109811.3 | 0.379597444 | 1.11E-06 |
| ATG16L2 | AL109811.3 | 0.493039195 | 7.16E-11 |
| ATG4B | AL109811.3 | 0.5099392 | 1.23E-11 |
| ATG4C | AL109811.3 | 0.399841195 | 2.55E-07 |
| BAG3 | AL109811.3 | -0.316602489 | 5.98E-05 |
| BAX | AL109811.3 | 0.348198852 | 9.01E-06 |
| CAPN10 | AL109811.3 | 0.591188397 | 5.56E-16 |
| CFLAR | AL109811.3 | 0.319036809 | 5.21E-05 |
| CXCR4 | AL109811.3 | 0.593180622 | 4.20E-16 |
| DAPK2 | AL109811.3 | 0.523850534 | 2.66E-12 |
| EIF4G1 | AL109811.3 | -0.36727686 | 2.59E-06 |
| HDAC6 | AL109811.3 | 0.411695962 | 1.03E-07 |
| IKBKB | AL109811.3 | 0.440904619 | 9.37E-09 |
| MAP2K7 | AL109811.3 | 0.41397621 | 8.58E-08 |
| MTMR14 | AL109811.3 | 0.33762152 | 1.74E-05 |
| NCKAP1 | AL109811.3 | -0.31655097 | 6.00E-05 |
| NKX2-3 | AL109811.3 | 0.332228712 | 2.41E-05 |
| PEX14 | AL109811.3 | 0.388333466 | 5.95E-07 |
| RAB24 | AL109811.3 | 0.394026503 | 3.92E-07 |
| RAF1 | AL109811.3 | 0.303522324 | 0.000123432 |
| STK11 | AL109811.3 | 0.393451378 | 4.09E-07 |
| TP53 | AL109811.3 | 0.301744734 | 0.000135847 |
| TP73 | AL109811.3 | 0.342769271 | 1.27E-05 |
| TSC2 | AL109811.3 | 0.502291189 | 2.76E-11 |
| ULK3 | AL109811.3 | 0.429103001 | 2.53E-08 |
| USP10 | AL109811.3 | -0.305036108 | 0.000113704 |
| WDR45 | AL109811.3 | 0.431403823 | 2.09E-08 |
| ATG16L2 | AL080317.1 | 0.403261158 | 1.97E-07 |
| ATG4B | AL080317.1 | 0.372059301 | 1.87E-06 |
| BAG3 | AL080317.1 | -0.35478863 | 5.91E-06 |
| BNIP1 | AL080317.1 | 0.304925254 | 0.000114391 |
| CAPN10 | AL080317.1 | 0.472084834 | 5.61E-10 |
| CAPN2 | AL080317.1 | -0.333791192 | 2.19E-05 |
| EIF4G1 | AL080317.1 | -0.301940124 | 0.000134427 |
| GABARAP | AL080317.1 | 0.333675093 | 2.21E-05 |
| PELP1 | AL080317.1 | 0.394063963 | 3.91E-07 |
| WDR45 | AL080317.1 | 0.352596916 | 6.81E-06 |
| ATG10 | AC096992.2 | 0.308559561 | 9.38E-05 |
| ATG16L2 | AC096992.2 | 0.363092085 | 3.43E-06 |
| ATG3 | AC096992.2 | 0.472856822 | 5.21E-10 |
| ATG4B | AC096992.2 | 0.404827174 | 1.74E-07 |
| ATG4C | AC096992.2 | 0.32708497 | 3.27E-05 |
| ATG4D | AC096992.2 | 0.489102677 | 1.06E-10 |
| BCL2 | AC096992.2 | 0.364854538 | 3.05E-06 |
| BID | AC096992.2 | 0.321377042 | 4.55E-05 |
| CAPN10 | AC096992.2 | 0.451789141 | 3.62E-09 |
| CASP3 | AC096992.2 | 0.349952482 | 8.06E-06 |
| CD46 | AC096992.2 | 0.300682053 | 0.000143815 |
| CDKN1B | AC096992.2 | 0.418542031 | 5.98E-08 |
| CDKN2A | AC096992.2 | 0.309312033 | 9.00E-05 |
| CFLAR | AC096992.2 | 0.3680161 | 2.46E-06 |
| CLN3 | AC096992.2 | 0.344242599 | 1.16E-05 |
| EEF2K | AC096992.2 | 0.415780641 | 7.44E-08 |
| HDAC1 | AC096992.2 | 0.316549952 | 6.00E-05 |
| HDAC6 | AC096992.2 | 0.448046882 | 5.04E-09 |
| IKBKB | AC096992.2 | 0.355897771 | 5.50E-06 |
| IKBKE | AC096992.2 | 0.386514231 | 6.78E-07 |
| ITGA3 | AC096992.2 | -0.329379839 | 2.85E-05 |
| MAP2K7 | AC096992.2 | 0.491521879 | 8.35E-11 |
| MAPK8 | AC096992.2 | 0.457056129 | 2.26E-09 |
| NKX2-3 | AC096992.2 | 0.397064305 | 3.13E-07 |
| PIK3R4 | AC096992.2 | 0.373802947 | 1.66E-06 |
| PINK1 | AC096992.2 | -0.314876449 | 6.60E-05 |
| PRKAB1 | AC096992.2 | 0.419190673 | 5.67E-08 |
| RAF1 | AC096992.2 | 0.420132934 | 5.26E-08 |
| STK11 | AC096992.2 | 0.400350283 | 2.45E-07 |
| TP73 | AC096992.2 | 0.40901556 | 1.26E-07 |
| TSC1 | AC096992.2 | 0.316386985 | 6.06E-05 |
| TSC2 | AC096992.2 | 0.422930147 | 4.20E-08 |
| ULK3 | AC096992.2 | 0.408524414 | 1.31E-07 |
| WDR45 | AC096992.2 | 0.364905322 | 3.04E-06 |
| ATG16L2 | CD27-AS1 | 0.33947496 | 1.55E-05 |
| ATG4B | CD27-AS1 | 0.337181268 | 1.79E-05 |
| BAK1 | CD27-AS1 | -0.382703853 | 8.90E-07 |
| BID | CD27-AS1 | 0.373412052 | 1.71E-06 |
| CAPN1 | CD27-AS1 | -0.310484458 | 8.43E-05 |
| CAPN10 | CD27-AS1 | 0.487895988 | 1.20E-10 |
| CASP4 | CD27-AS1 | -0.310837796 | 8.27E-05 |
| CDKN1B | CD27-AS1 | 0.43049288 | 2.26E-08 |
| CLN3 | CD27-AS1 | 0.427789761 | 2.82E-08 |
| CXCR4 | CD27-AS1 | 0.362053352 | 3.67E-06 |
| DAPK2 | CD27-AS1 | 0.351376004 | 7.36E-06 |
| GABARAP | CD27-AS1 | 0.340557169 | 1.45E-05 |
| GABARAPL1 | CD27-AS1 | 0.346051752 | 1.03E-05 |
| GNAI3 | CD27-AS1 | -0.447437564 | 5.32E-09 |
| HDAC6 | CD27-AS1 | 0.389461736 | 5.48E-07 |
| IKBKB | CD27-AS1 | 0.465487137 | 1.04E-09 |
| ITGA3 | CD27-AS1 | -0.44539838 | 6.35E-09 |
| MAP2K7 | CD27-AS1 | 0.350294069 | 7.89E-06 |
| MLST8 | CD27-AS1 | 0.308233714 | 9.55E-05 |
| NRG1 | CD27-AS1 | -0.307239973 | 0.000100813 |
| PELP1 | CD27-AS1 | 0.393502075 | 4.08E-07 |
| PINK1 | CD27-AS1 | -0.348056673 | 9.09E-06 |
| PRKAB1 | CD27-AS1 | 0.308275247 | 9.52E-05 |
| PTK6 | CD27-AS1 | -0.308215006 | 9.56E-05 |
| RHEB | CD27-AS1 | 0.432084215 | 1.98E-08 |
| TSC2 | CD27-AS1 | 0.301296695 | 0.000139155 |
| ULK3 | CD27-AS1 | 0.42509905 | 3.52E-08 |
| UVRAG | CD27-AS1 | 0.313766138 | 7.02E-05 |
| VAMP3 | CD27-AS1 | -0.316359624 | 6.07E-05 |
| WIPI1 | CD27-AS1 | 0.333621829 | 2.21E-05 |
| BAX | AL031665.2 | 0.488560436 | 1.12E-10 |
| BIRC5 | AL031665.2 | 0.501796263 | 2.90E-11 |
| BNIP1 | AL031665.2 | 0.325081048 | 3.67E-05 |
| CAPN2 | AL031665.2 | -0.315831139 | 6.25E-05 |
| EIF4EBP1 | AL031665.2 | 0.337690877 | 1.73E-05 |
| FKBP1A | AL031665.2 | 0.508388465 | 1.45E-11 |
| GABARAPL2 | AL031665.2 | 0.309552002 | 8.88E-05 |
| MAPK1 | AL031665.2 | -0.32366654 | 3.99E-05 |
| MAPK8IP1 | AL031665.2 | 0.346196995 | 1.02E-05 |
| MLST8 | AL031665.2 | 0.318652998 | 5.32E-05 |
| NFKB1 | AL031665.2 | -0.33338582 | 2.25E-05 |
| WDFY3 | AL031665.2 | -0.303746639 | 0.000121943 |
| WDR45 | AL031665.2 | 0.384121926 | 8.05E-07 |
| MAPK8IP1 | AC023043.4 | 0.396223282 | 3.34E-07 |
| ATG4B | TRIM52-AS1 | 0.30132186 | 0.000138967 |
| ATG4C | TRIM52-AS1 | 0.318726157 | 5.30E-05 |
| ATIC | TRIM52-AS1 | 0.424262778 | 3.77E-08 |
| BAG3 | TRIM52-AS1 | -0.336554766 | 1.85E-05 |
| BAX | TRIM52-AS1 | 0.565433816 | 1.78E-14 |
| BIRC5 | TRIM52-AS1 | 0.542842796 | 2.95E-13 |
| BIRC6 | TRIM52-AS1 | -0.309423466 | 8.94E-05 |
| BNIP1 | TRIM52-AS1 | 0.579680353 | 2.72E-15 |
| CAPN10 | TRIM52-AS1 | 0.370034271 | 2.15E-06 |
| CAPN2 | TRIM52-AS1 | -0.354538821 | 6.01E-06 |
| CDKN1B | TRIM52-AS1 | 0.316800934 | 5.92E-05 |
| CDKN2A | TRIM52-AS1 | 0.339505523 | 1.55E-05 |
| CLN3 | TRIM52-AS1 | 0.304027694 | 0.000120101 |
| CXCR4 | TRIM52-AS1 | 0.380348881 | 1.05E-06 |
| EIF4G1 | TRIM52-AS1 | -0.400017068 | 2.51E-07 |
| FKBP1A | TRIM52-AS1 | 0.503617792 | 2.40E-11 |
| GABARAP | TRIM52-AS1 | 0.393463715 | 4.09E-07 |
| HIF1A | TRIM52-AS1 | -0.313137568 | 7.27E-05 |
| ITGA6 | TRIM52-AS1 | -0.343436997 | 1.21E-05 |
| KIF5B | TRIM52-AS1 | -0.376104002 | 1.42E-06 |
| MAPK1 | TRIM52-AS1 | -0.373264041 | 1.72E-06 |
| MAPK8IP1 | TRIM52-AS1 | 0.495675673 | 5.47E-11 |
| MTMR14 | TRIM52-AS1 | 0.373063275 | 1.75E-06 |
| NCKAP1 | TRIM52-AS1 | -0.422260155 | 4.43E-08 |
| PEX14 | TRIM52-AS1 | 0.34417386 | 1.16E-05 |
| PTK6 | TRIM52-AS1 | -0.302520099 | 0.000130295 |
| RAB1A | TRIM52-AS1 | -0.428934249 | 2.57E-08 |
| TP63 | TRIM52-AS1 | -0.358505871 | 4.64E-06 |
| USP10 | TRIM52-AS1 | -0.321193314 | 4.60E-05 |
| WDFY3 | TRIM52-AS1 | -0.375805026 | 1.45E-06 |
| WDR45 | TRIM52-AS1 | 0.421136565 | 4.85E-08 |
| DIRAS3 | AL365181.2 | 0.514473095 | 7.51E-12 |
| FKBP1B | AL365181.2 | 0.313728331 | 7.03E-05 |
| NAMPT | AL365181.2 | 0.323230657 | 4.09E-05 |
| SQSTM1 | AL365181.2 | 0.305363401 | 0.000111697 |
| CASP8 | AC023310.4 | 0.378193529 | 1.22E-06 |
| CFLAR | AC023310.4 | 0.378271737 | 1.22E-06 |
| DIRAS3 | AC023310.4 | 0.310353479 | 8.49E-05 |
| VAMP3 | AC023310.4 | 0.467566099 | 8.58E-10 |
| MAP2K7 | AC090246.1 | 0.328160343 | 3.07E-05 |
| NFE2L2 | AC090246.1 | 0.31505023 | 6.53E-05 |
| PINK1 | AC090246.1 | -0.304676127 | 0.00011595 |
| ATG16L2 | AC090515.4 | 0.308712913 | 9.30E-05 |
| ATG9B | AC090515.4 | 0.354966901 | 5.84E-06 |
| FKBP1B | AC090515.4 | 0.399881962 | 2.54E-07 |
| FOXO1 | AC090515.4 | -0.429200825 | 2.51E-08 |
| MYC | AC090515.4 | 0.456831843 | 2.30E-09 |
| NAF1 | AC090515.4 | 0.304732319 | 0.000115597 |
| PRKCQ | AC090515.4 | 0.338455539 | 1.65E-05 |
| ULK3 | AC090515.4 | 0.309213077 | 9.04E-05 |
| CTSB | AC105118.1 | 0.326151788 | 3.45E-05 |
| IL24 | AC105118.1 | 0.41291218 | 9.33E-08 |
| CASP1 | LINC01527 | 0.330622126 | 2.65E-05 |
| PTK6 | LINC01527 | 0.302905527 | 0.000127615 |
| BNIP3 | GASAL1 | 0.32324723 | 4.09E-05 |
| GABARAP | GASAL1 | -0.30353121 | 0.000123373 |
| ITGB4 | GASAL1 | 0.356606571 | 5.25E-06 |
| PTK6 | GASAL1 | 0.394284931 | 3.85E-07 |
| EIF2S1 | AC004846.1 | 0.313852804 | 6.99E-05 |
| HIF1A | AC004846.1 | 0.354684086 | 5.95E-06 |
| ATG12 | AC127502.2 | 0.338869014 | 1.61E-05 |
| ATG16L2 | AC127502.2 | 0.387396833 | 6.36E-07 |
| ATG4C | AC127502.2 | 0.348520251 | 8.83E-06 |
| CAPN10 | AC127502.2 | 0.392434497 | 4.41E-07 |
| CASP3 | AC127502.2 | 0.373877997 | 1.65E-06 |
| CDKN1B | AC127502.2 | 0.32616307 | 3.45E-05 |
| CFLAR | AC127502.2 | 0.337271137 | 1.78E-05 |
| CXCR4 | AC127502.2 | 0.490670415 | 9.09E-11 |
| FAS | AC127502.2 | 0.331431776 | 2.52E-05 |
| HDAC6 | AC127502.2 | 0.469473323 | 7.18E-10 |
| IKBKB | AC127502.2 | 0.444686952 | 6.76E-09 |
| IKBKE | AC127502.2 | 0.300168665 | 0.000147819 |
| MAPK9 | AC127502.2 | 0.423244424 | 4.09E-08 |
| RAB24 | AC127502.2 | 0.333341816 | 2.25E-05 |
| RAF1 | AC127502.2 | 0.323714954 | 3.98E-05 |
| STK11 | AC127502.2 | 0.329534174 | 2.83E-05 |
| TP73 | AC127502.2 | 0.348506439 | 8.84E-06 |
| TSC2 | AC127502.2 | 0.40308712 | 1.99E-07 |
| ULK3 | AC127502.2 | 0.471665468 | 5.83E-10 |
| UVRAG | AC127502.2 | 0.310639296 | 8.36E-05 |
| WDR45 | AC127502.2 | 0.445052519 | 6.55E-09 |
| ARNT | AC004918.3 | 0.338806477 | 1.62E-05 |
| BIRC6 | AC004918.3 | 0.378488634 | 1.20E-06 |
| CAMKK2 | AC004918.3 | 0.304575588 | 0.000116585 |
| CD46 | AC004918.3 | 0.314985528 | 6.55E-05 |
| EIF2AK3 | AC004918.3 | 0.390211055 | 5.19E-07 |
| NBR1 | AC004918.3 | 0.308575436 | 9.37E-05 |
| NCKAP1 | AC004918.3 | 0.303943245 | 0.000120652 |
| PIK3R4 | AC004918.3 | 0.368567304 | 2.37E-06 |
| RB1CC1 | AC004918.3 | 0.310426517 | 8.46E-05 |
| UVRAG | AC004918.3 | 0.303398544 | 0.000124261 |
| ARNT | FAM111A-DT | 0.315724394 | 6.29E-05 |
| ATG10 | FAM111A-DT | 0.404766363 | 1.75E-07 |
| ATG12 | FAM111A-DT | 0.389790773 | 5.35E-07 |
| ATG2A | FAM111A-DT | 0.333979338 | 2.17E-05 |
| ATG3 | FAM111A-DT | 0.316287993 | 6.09E-05 |
| ATG4A | FAM111A-DT | 0.357379424 | 4.99E-06 |
| ATG4C | FAM111A-DT | 0.481630547 | 2.23E-10 |
| BCL2 | FAM111A-DT | 0.458173725 | 2.04E-09 |
| CALCOCO2 | FAM111A-DT | 0.358456263 | 4.66E-06 |
| CASP3 | FAM111A-DT | 0.405166108 | 1.70E-07 |
| CD46 | FAM111A-DT | 0.364895434 | 3.04E-06 |
| CDKN1B | FAM111A-DT | 0.376745438 | 1.35E-06 |
| CDKN2A | FAM111A-DT | 0.317129945 | 5.81E-05 |
| CFLAR | FAM111A-DT | 0.405370688 | 1.67E-07 |
| CXCR4 | FAM111A-DT | 0.417691634 | 6.40E-08 |
| DRAM1 | FAM111A-DT | 0.363128467 | 3.42E-06 |
| EEF2K | FAM111A-DT | 0.371538704 | 1.94E-06 |
| EIF2AK3 | FAM111A-DT | 0.31750833 | 5.68E-05 |
| EIF2S1 | FAM111A-DT | -0.320605004 | 4.76E-05 |
| FAS | FAM111A-DT | 0.491918644 | 8.02E-11 |
| FOXO3 | FAM111A-DT | 0.322695953 | 4.22E-05 |
| HDAC6 | FAM111A-DT | 0.579593036 | 2.75E-15 |
| IKBKB | FAM111A-DT | 0.343980478 | 1.17E-05 |
| IKBKE | FAM111A-DT | 0.527643594 | 1.73E-12 |
| KLHL24 | FAM111A-DT | 0.367852969 | 2.49E-06 |
| MAPK8 | FAM111A-DT | 0.461691694 | 1.48E-09 |
| MAPK9 | FAM111A-DT | 0.378493317 | 1.20E-06 |
| MYC | FAM111A-DT | -0.379931531 | 1.08E-06 |
| NBR1 | FAM111A-DT | 0.381907647 | 9.42E-07 |
| NKX2-3 | FAM111A-DT | 0.38547632 | 7.31E-07 |
| PARP1 | FAM111A-DT | 0.466091612 | 9.85E-10 |
| PIK3R4 | FAM111A-DT | 0.402316729 | 2.11E-07 |
| PRKAB1 | FAM111A-DT | 0.388599301 | 5.83E-07 |
| PTK6 | FAM111A-DT | -0.34347854 | 1.21E-05 |
| RAB33B | FAM111A-DT | 0.399163141 | 2.68E-07 |
| RAF1 | FAM111A-DT | 0.407061486 | 1.47E-07 |
| RPS6KB1 | FAM111A-DT | 0.325742478 | 3.53E-05 |
| TP53 | FAM111A-DT | 0.43578693 | 1.45E-08 |
| TP73 | FAM111A-DT | 0.562960135 | 2.45E-14 |
| TSC1 | FAM111A-DT | 0.3111959 | 8.10E-05 |
| TSC2 | FAM111A-DT | 0.546863544 | 1.82E-13 |
| ULK3 | FAM111A-DT | 0.339547721 | 1.54E-05 |
| WDR45 | FAM111A-DT | 0.348779304 | 8.68E-06 |
| ATG10 | AC106795.2 | 0.319838494 | 4.97E-05 |
| ATG16L2 | AC106795.2 | 0.310797783 | 8.28E-05 |
| ATG4B | AC106795.2 | 0.404455248 | 1.79E-07 |
| ATG4D | AC106795.2 | 0.317063781 | 5.83E-05 |
| BAX | AC106795.2 | 0.329293548 | 2.87E-05 |
| BID | AC106795.2 | 0.462387246 | 1.39E-09 |
| BNIP1 | AC106795.2 | 0.306344272 | 0.000105878 |
| CAPN10 | AC106795.2 | 0.498701606 | 4.00E-11 |
| DIRAS3 | AC106795.2 | 0.568616138 | 1.18E-14 |
| GABARAP | AC106795.2 | 0.439234116 | 1.08E-08 |
| MAP1LC3A | AC106795.2 | 0.47777496 | 3.25E-10 |
| PELP1 | AC106795.2 | 0.479100085 | 2.86E-10 |
| PRKAB1 | AC106795.2 | 0.317341679 | 5.74E-05 |
| WDR45 | AC106795.2 | 0.300547665 | 0.000144853 |
| ARSB | MIR210HG | -0.319764591 | 5.00E-05 |
| ATG2B | MIR210HG | -0.307699068 | 9.83E-05 |
| BIRC6 | MIR210HG | -0.400278107 | 2.46E-07 |
| BNIP3 | MIR210HG | 0.369390794 | 2.24E-06 |
| CAMKK2 | MIR210HG | -0.372673446 | 1.79E-06 |
| CANX | MIR210HG | -0.397198666 | 3.10E-07 |
| GABARAPL2 | MIR210HG | 0.34032376 | 1.47E-05 |
| KIF5B | MIR210HG | -0.370369157 | 2.10E-06 |
| MAPK1 | MIR210HG | -0.400497295 | 2.42E-07 |
| MTOR | MIR210HG | -0.392402263 | 4.42E-07 |
| NBR1 | MIR210HG | -0.322647151 | 4.23E-05 |
| PARP1 | MIR210HG | -0.346044841 | 1.03E-05 |
| PTEN | MIR210HG | -0.31224792 | 7.64E-05 |
| RAB24 | MIR210HG | 0.340892831 | 1.42E-05 |
| RAC1 | MIR210HG | 0.309671823 | 8.82E-05 |
| RPTOR | MIR210HG | -0.317823808 | 5.58E-05 |
| SIRT1 | MIR210HG | -0.380544831 | 1.04E-06 |
| VAMP7 | MIR210HG | -0.32068135 | 4.74E-05 |
| ATG2B | AL049840.3 | 0.522281275 | 3.17E-12 |
| BAX | AL049840.3 | -0.352701153 | 6.76E-06 |
| BNIP1 | AL049840.3 | -0.374789515 | 1.55E-06 |
| CAPN2 | AL049840.3 | 0.306085345 | 0.000107386 |
| CLN3 | AL049840.3 | -0.34479203 | 1.12E-05 |
| DDIT3 | AL049840.3 | -0.357931136 | 4.82E-06 |
| EIF2S1 | AL049840.3 | 0.338818227 | 1.62E-05 |
| GABARAP | AL049840.3 | -0.304235541 | 0.000118756 |
| GABARAPL2 | AL049840.3 | -0.322338861 | 4.31E-05 |
| GOPC | AL049840.3 | 0.344056273 | 1.17E-05 |
| HSPA8 | AL049840.3 | 0.34938355 | 8.36E-06 |
| ITGA3 | AL049840.3 | 0.338553368 | 1.64E-05 |
| ITGA6 | AL049840.3 | 0.369830834 | 2.18E-06 |
| ITGB1 | AL049840.3 | 0.327564623 | 3.18E-05 |
| ITGB4 | AL049840.3 | 0.407666342 | 1.40E-07 |
| NFKB1 | AL049840.3 | 0.320504108 | 4.79E-05 |
| NRG1 | AL049840.3 | 0.370544552 | 2.08E-06 |
| TSC1 | AL049840.3 | 0.327441052 | 3.20E-05 |
| WDFY3 | AL049840.3 | 0.323053098 | 4.13E-05 |
| DAPK2 | AC011451.1 | 0.310358444 | 8.49E-05 |
| ARSB | AC010503.4 | -0.33530404 | 2.00E-05 |
| ATF4 | AC010503.4 | 0.362601177 | 3.54E-06 |
| ATG16L2 | AC010503.4 | 0.369589626 | 2.21E-06 |
| ATG2B | AC010503.4 | -0.427338427 | 2.93E-08 |
| ATG3 | AC010503.4 | 0.330673038 | 2.64E-05 |
| ATG4B | AC010503.4 | 0.561934423 | 2.79E-14 |
| ATG4D | AC010503.4 | 0.573570617 | 6.16E-15 |
| BAG1 | AC010503.4 | 0.340075289 | 1.50E-05 |
| BAX | AC010503.4 | 0.419967584 | 5.33E-08 |
| BID | AC010503.4 | 0.329096382 | 2.90E-05 |
| BNIP1 | AC010503.4 | 0.499178897 | 3.81E-11 |
| CANX | AC010503.4 | -0.334866667 | 2.05E-05 |
| CAPN10 | AC010503.4 | 0.464692912 | 1.12E-09 |
| CAPNS1 | AC010503.4 | 0.370243863 | 2.12E-06 |
| CDKN2A | AC010503.4 | 0.397363596 | 3.06E-07 |
| CFLAR | AC010503.4 | 0.35345615 | 6.44E-06 |
| CLN3 | AC010503.4 | 0.440666556 | 9.57E-09 |
| DDIT3 | AC010503.4 | 0.335136438 | 2.02E-05 |
| DLC1 | AC010503.4 | -0.363894494 | 3.25E-06 |
| FOXO1 | AC010503.4 | -0.319091324 | 5.19E-05 |
| GABARAP | AC010503.4 | 0.343100375 | 1.24E-05 |
| GOPC | AC010503.4 | -0.401192206 | 2.30E-07 |
| HSP90AB1 | AC010503.4 | -0.303013323 | 0.000126874 |
| ITGA3 | AC010503.4 | -0.377065671 | 1.32E-06 |
| ITGA6 | AC010503.4 | -0.317200007 | 5.78E-05 |
| ITGB1 | AC010503.4 | -0.356492789 | 5.29E-06 |
| MAP1LC3A | AC010503.4 | 0.384879644 | 7.62E-07 |
| MAP2K7 | AC010503.4 | 0.428264977 | 2.71E-08 |
| MAPK1 | AC010503.4 | -0.370120424 | 2.14E-06 |
| MTMR14 | AC010503.4 | 0.359648343 | 4.31E-06 |
| PELP1 | AC010503.4 | 0.407679773 | 1.40E-07 |
| PEX14 | AC010503.4 | 0.387303237 | 6.41E-07 |
| PINK1 | AC010503.4 | -0.319954421 | 4.94E-05 |
| PRKAB1 | AC010503.4 | 0.333364578 | 2.25E-05 |
| PTEN | AC010503.4 | -0.372204218 | 1.85E-06 |
| RAB11A | AC010503.4 | 0.303009885 | 0.000126898 |
| RAB24 | AC010503.4 | 0.430144547 | 2.32E-08 |
| RAC1 | AC010503.4 | 0.333783142 | 2.19E-05 |
| STK11 | AC010503.4 | 0.413942541 | 8.61E-08 |
| TM9SF1 | AC010503.4 | -0.33313421 | 2.28E-05 |
| ULK3 | AC010503.4 | 0.302681118 | 0.000129169 |
| USP10 | AC010503.4 | -0.363636101 | 3.31E-06 |
| WDR45 | AC010503.4 | 0.46768984 | 8.48E-10 |
| ATF4 | LINC01004 | 0.307057945 | 0.000101824 |
| ATG12 | LINC01004 | 0.323843801 | 3.95E-05 |
| ATG16L2 | LINC01004 | 0.421801592 | 4.60E-08 |
| ATG4B | LINC01004 | 0.411583979 | 1.04E-07 |
| ATG9B | LINC01004 | 0.30519236 | 0.000112741 |
| CAPN10 | LINC01004 | 0.446868177 | 5.59E-09 |
| EIF4G1 | LINC01004 | -0.31195503 | 7.77E-05 |
| GABARAPL2 | LINC01004 | 0.328031529 | 3.09E-05 |
| STK11 | LINC01004 | 0.300101931 | 0.000148347 |
| ULK3 | LINC01004 | 0.303798388 | 0.000121602 |
| BAX | HOXC-AS1 | 0.467666367 | 8.50E-10 |
| CAPN10 | HOXC-AS1 | 0.356394817 | 5.33E-06 |
| CXCR4 | HOXC-AS1 | 0.358186944 | 4.74E-06 |
| DIRAS3 | HOXC-AS1 | 0.341968167 | 1.33E-05 |
| ARNT | LINC00392 | 0.369251613 | 2.27E-06 |
| ATF6 | LINC00392 | 0.32364177 | 3.99E-05 |
| NCKAP1 | LINC00392 | 0.330176194 | 2.72E-05 |
| PIK3C3 | LINC00392 | 0.366564814 | 2.72E-06 |
| SQSTM1 | LINC00392 | 0.326360284 | 3.41E-05 |
| PINK1 | LINC02159 | 0.311534354 | 7.95E-05 |
| ATG7 | AC007319.1 | 0.308040434 | 9.65E-05 |
| CAPNS1 | AC007319.1 | 0.331172224 | 2.56E-05 |
| ERBB2 | AC007319.1 | 0.399930902 | 2.53E-07 |
| IKBKE | AC007319.1 | 0.377136291 | 1.32E-06 |
| MAPK8 | AC007319.1 | 0.300762549 | 0.000143196 |
| ITGA3 | AC009570.1 | -0.322895123 | 4.17E-05 |
| ATF4 | AC100814.1 | 0.310604105 | 8.37E-05 |
| ATG10 | AC100814.1 | 0.328085248 | 3.08E-05 |
| ATG16L2 | AC100814.1 | 0.36509143 | 3.00E-06 |
| ATG3 | AC100814.1 | 0.301553141 | 0.000137252 |
| ATG4B | AC100814.1 | 0.423402496 | 4.04E-08 |
| BAG3 | AC100814.1 | -0.345198201 | 1.09E-05 |
| BAX | AC100814.1 | 0.514526274 | 7.46E-12 |
| BIRC5 | AC100814.1 | 0.350243592 | 7.91E-06 |
| BNIP1 | AC100814.1 | 0.379380892 | 1.13E-06 |
| CAPN10 | AC100814.1 | 0.503435878 | 2.44E-11 |
| CLN3 | AC100814.1 | 0.315339778 | 6.43E-05 |
| CXCR4 | AC100814.1 | 0.348000403 | 9.12E-06 |
| EIF4G1 | AC100814.1 | -0.369022706 | 2.30E-06 |
| MAP2K7 | AC100814.1 | 0.330403823 | 2.68E-05 |
| MAPK1 | AC100814.1 | -0.344794373 | 1.12E-05 |
| MTMR14 | AC100814.1 | 0.3708264 | 2.04E-06 |
| NPC1 | AC100814.1 | -0.317746565 | 5.61E-05 |
| PEX14 | AC100814.1 | 0.405044372 | 1.72E-07 |
| WDR45 | AC100814.1 | 0.369120125 | 2.29E-06 |
| ARSB | TGFB2-AS1 | 0.341130146 | 1.40E-05 |
| ATF6 | TGFB2-AS1 | 0.343473837 | 1.21E-05 |
| DLC1 | TGFB2-AS1 | 0.434901316 | 1.56E-08 |
| MBTPS2 | TGFB2-AS1 | 0.377271136 | 1.30E-06 |
| PEA15 | TGFB2-AS1 | 0.364608083 | 3.10E-06 |
| ATG10 | AC008124.1 | 0.324503298 | 3.80E-05 |
| ATG12 | AC008124.1 | 0.349693146 | 8.19E-06 |
| ATG16L2 | AC008124.1 | 0.304193083 | 0.000119029 |
| ATG4C | AC008124.1 | 0.332965028 | 2.30E-05 |
| ATG4D | AC008124.1 | 0.312694684 | 7.45E-05 |
| BAK1 | AC008124.1 | -0.350060056 | 8.01E-06 |
| BCL2 | AC008124.1 | 0.382963808 | 8.74E-07 |
| CAPN10 | AC008124.1 | 0.391667414 | 4.67E-07 |
| CASP3 | AC008124.1 | 0.312932794 | 7.36E-05 |
| CD46 | AC008124.1 | 0.382684266 | 8.92E-07 |
| CDKN1B | AC008124.1 | 0.429025942 | 2.55E-08 |
| CFLAR | AC008124.1 | 0.336718384 | 1.84E-05 |
| CXCR4 | AC008124.1 | 0.347994514 | 9.13E-06 |
| EIF2AK3 | AC008124.1 | 0.31071267 | 8.32E-05 |
| HDAC1 | AC008124.1 | 0.324121141 | 3.88E-05 |
| HDAC6 | AC008124.1 | 0.450438944 | 4.08E-09 |
| IKBKB | AC008124.1 | 0.339456128 | 1.55E-05 |
| ITGA3 | AC008124.1 | -0.433788918 | 1.71E-08 |
| KLHL24 | AC008124.1 | 0.303362698 | 0.000124502 |
| MAP2K7 | AC008124.1 | 0.429602602 | 2.43E-08 |
| MAPK8 | AC008124.1 | 0.446388838 | 5.83E-09 |
| MTMR14 | AC008124.1 | 0.391250574 | 4.81E-07 |
| NKX2-3 | AC008124.1 | 0.312092835 | 7.71E-05 |
| PRKAB1 | AC008124.1 | 0.318449023 | 5.39E-05 |
| RAF1 | AC008124.1 | 0.465278427 | 1.06E-09 |
| SIRT1 | AC008124.1 | 0.383951614 | 8.15E-07 |
| TP53 | AC008124.1 | 0.352007839 | 7.07E-06 |
| TSC1 | AC008124.1 | 0.338973029 | 1.60E-05 |
| TSC2 | AC008124.1 | 0.303687588 | 0.000122334 |
| ULK3 | AC008124.1 | 0.376352096 | 1.39E-06 |
| UVRAG | AC008124.1 | 0.317743388 | 5.61E-05 |
| WIPI1 | LINC00174 | 0.327124764 | 3.26E-05 |
| ITGA3 | AC004812.2 | -0.331838724 | 2.46E-05 |
| PRKAB1 | AC004812.2 | 0.396398237 | 3.29E-07 |
| ARNT | AL158212.3 | 0.390038517 | 5.25E-07 |
| ATG10 | AL158212.3 | 0.349829401 | 8.12E-06 |
| ATG12 | AL158212.3 | 0.31635509 | 6.07E-05 |
| ATG2A | AL158212.3 | 0.469215208 | 7.35E-10 |
| ATG4C | AL158212.3 | 0.41891252 | 5.80E-08 |
| ATG7 | AL158212.3 | 0.367722389 | 2.51E-06 |
| BCL2 | AL158212.3 | 0.400747917 | 2.38E-07 |
| CD46 | AL158212.3 | 0.359537937 | 4.34E-06 |
| CDKN1B | AL158212.3 | 0.394854124 | 3.69E-07 |
| CFLAR | AL158212.3 | 0.326596166 | 3.36E-05 |
| CXCR4 | AL158212.3 | 0.32759854 | 3.17E-05 |
| EEF2K | AL158212.3 | 0.457390355 | 2.19E-09 |
| EIF4EBP1 | AL158212.3 | -0.302668604 | 0.000129256 |
| FAS | AL158212.3 | 0.473148726 | 5.07E-10 |
| HDAC6 | AL158212.3 | 0.334667158 | 2.08E-05 |
| HSPA5 | AL158212.3 | -0.307229761 | 0.00010087 |
| IKBKB | AL158212.3 | 0.300317274 | 0.000146649 |
| IKBKE | AL158212.3 | 0.371014714 | 2.01E-06 |
| MAPK8 | AL158212.3 | 0.55824487 | 4.46E-14 |
| MTMR14 | AL158212.3 | 0.379953532 | 1.08E-06 |
| MTOR | AL158212.3 | 0.467275601 | 8.82E-10 |
| NBR1 | AL158212.3 | 0.321481362 | 4.53E-05 |
| NFKB1 | AL158212.3 | 0.368140699 | 2.44E-06 |
| NKX2-3 | AL158212.3 | 0.448611698 | 4.80E-09 |
| PARP1 | AL158212.3 | 0.41721715 | 6.64E-08 |
| PIK3R4 | AL158212.3 | 0.364551219 | 3.11E-06 |
| RAB33B | AL158212.3 | 0.317865903 | 5.57E-05 |
| RAF1 | AL158212.3 | 0.463181361 | 1.29E-09 |
| RB1 | AL158212.3 | 0.338355318 | 1.66E-05 |
| SH3GLB1 | AL158212.3 | 0.430781895 | 2.20E-08 |
| SIRT1 | AL158212.3 | 0.37509995 | 1.52E-06 |
| TP53 | AL158212.3 | 0.481552679 | 2.25E-10 |
| TP73 | AL158212.3 | 0.564014605 | 2.14E-14 |
| TSC1 | AL158212.3 | 0.379757102 | 1.10E-06 |
| TSC2 | AL158212.3 | 0.565692444 | 1.73E-14 |
| BID | LINC01637 | 0.321227317 | 4.59E-05 |
| ATG3 | AL021807.1 | 0.306816242 | 0.00010318 |
| ATG4A | AL021807.1 | 0.460420969 | 1.66E-09 |
| ATIC | AL021807.1 | 0.429516484 | 2.45E-08 |
| BAX | AL021807.1 | 0.317305534 | 5.75E-05 |
| BIRC5 | AL021807.1 | 0.521022575 | 3.65E-12 |
| CDKN2A | AL021807.1 | 0.435472747 | 1.49E-08 |
| GABARAPL2 | AL021807.1 | 0.316984829 | 5.85E-05 |
| HDAC6 | AL021807.1 | 0.336204281 | 1.89E-05 |
| MAPK8IP1 | AL021807.1 | 0.615397853 | 1.59E-17 |
| MTMR14 | AL021807.1 | 0.372913338 | 1.76E-06 |
| PRKAR1A | AL021807.1 | 0.38677487 | 6.65E-07 |
| VAMP7 | AL021807.1 | 0.312923117 | 7.36E-05 |
| ITGA3 | AC006333.2 | -0.322202436 | 4.34E-05 |
| TP53INP2 | AC006333.2 | 0.350739557 | 7.67E-06 |
| BIRC6 | AC100801.1 | -0.318777426 | 5.29E-05 |
| ATG16L2 | AL928654.2 | 0.38868501 | 5.80E-07 |
| CAPN10 | AL928654.2 | 0.318219232 | 5.46E-05 |
| CX3CL1 | AL928654.2 | 0.308325121 | 9.50E-05 |
| CXCR4 | AL928654.2 | 0.339115389 | 1.59E-05 |
| HDAC6 | AL928654.2 | 0.313255655 | 7.22E-05 |
| IKBKB | AL928654.2 | 0.435560399 | 1.48E-08 |
| TSC1 | AL928654.2 | 0.364767697 | 3.07E-06 |
| TSC2 | AL928654.2 | 0.335464927 | 1.98E-05 |
| ULK3 | AL928654.2 | 0.355721159 | 5.56E-06 |
| ATG10 | AL391244.3 | 0.386604372 | 6.74E-07 |
| ATG12 | AL391244.3 | 0.321836514 | 4.43E-05 |
| ATG16L2 | AL391244.3 | 0.35349638 | 6.42E-06 |
| CAPN10 | AL391244.3 | 0.327232395 | 3.24E-05 |
| CFLAR | AL391244.3 | 0.421025384 | 4.90E-08 |
| PEX14 | AL391244.3 | 0.449275239 | 4.52E-09 |
| VAMP3 | AL391244.3 | 0.393841429 | 3.98E-07 |
| BID | AC005034.3 | 0.312757921 | 7.43E-05 |
| BIRC6 | AC005034.3 | 0.368924353 | 2.32E-06 |
| PARP1 | AC005034.3 | 0.323364959 | 4.06E-05 |
| RPS6KB1 | AC005034.3 | 0.341922548 | 1.33E-05 |
| NFE2L2 | FEZF1-AS1 | 0.392708795 | 4.32E-07 |
| PINK1 | FEZF1-AS1 | -0.308551847 | 9.38E-05 |
| BIRC6 | AC145207.8 | 0.333605789 | 2.22E-05 |
| EIF4G1 | AC145207.8 | 0.30501642 | 0.000113825 |
| GABARAPL1 | AC145207.8 | 0.324041204 | 3.90E-05 |
| GNAI3 | AC145207.8 | -0.32421293 | 3.86E-05 |
| KLHL24 | AC145207.8 | 0.32291061 | 4.17E-05 |
| NAMPT | AC145207.8 | 0.481244596 | 2.32E-10 |
| NBR1 | AC145207.8 | 0.317944439 | 5.54E-05 |
| NFE2L2 | AC145207.8 | 0.330625352 | 2.65E-05 |
| SQSTM1 | AC145207.8 | 0.338274087 | 1.67E-05 |
| TP63 | AC145207.8 | 0.537958869 | 5.26E-13 |
| USP10 | AC145207.8 | 0.307765213 | 9.79E-05 |
| BAX | CDC37L1-DT | 0.399868301 | 2.54E-07 |
| BIRC5 | CDC37L1-DT | 0.40895787 | 1.27E-07 |
| UVRAG | AP003486.1 | 0.350662357 | 7.70E-06 |
| WDFY3 | AP003486.1 | 0.312855559 | 7.39E-05 |
| ARNT | AC068831.6 | -0.305624954 | 0.000110116 |
| ARSB | AC068831.6 | -0.422431864 | 4.37E-08 |
| ATG4C | AC068831.6 | -0.322134239 | 4.36E-05 |
| CANX | AC068831.6 | -0.309502066 | 8.90E-05 |
| EDEM1 | AC068831.6 | -0.337331705 | 1.77E-05 |
| FAS | AC068831.6 | -0.351954443 | 7.09E-06 |
| ITPR1 | AC068831.6 | -0.358610382 | 4.61E-06 |
| MAPK8 | AC068831.6 | -0.324644312 | 3.77E-05 |
| MAPK9 | AC068831.6 | -0.323993264 | 3.91E-05 |
| MTOR | AC068831.6 | -0.325034491 | 3.68E-05 |
| PARP1 | AC068831.6 | -0.309563988 | 8.87E-05 |
| PIK3R4 | AC068831.6 | -0.314992685 | 6.55E-05 |
| PTK6 | AC068831.6 | 0.309776804 | 8.77E-05 |
| TP53 | AC068831.6 | -0.331786459 | 2.47E-05 |
| ARSB | AL359076.1 | 0.311809754 | 7.83E-05 |
| ATG4D | AL359076.1 | 0.382697573 | 8.91E-07 |
| BAG1 | AL359076.1 | 0.368080956 | 2.45E-06 |
| BCL2 | AL359076.1 | 0.360466169 | 4.08E-06 |
| CCR2 | AL359076.1 | 0.318446523 | 5.39E-05 |
| CDKN1A | AL359076.1 | -0.318602601 | 5.34E-05 |
| CDKN1B | AL359076.1 | 0.377634233 | 1.27E-06 |
| CDKN2A | AL359076.1 | 0.32673367 | 3.33E-05 |
| CXCR4 | AL359076.1 | 0.409958236 | 1.17E-07 |
| DRAM1 | AL359076.1 | 0.311224639 | 8.09E-05 |
| IFNG | AL359076.1 | 0.367256036 | 2.59E-06 |
| IKBKE | AL359076.1 | 0.372466944 | 1.82E-06 |
| ITPR1 | AL359076.1 | 0.302869662 | 0.000127862 |
| KLHL24 | AL359076.1 | 0.404042887 | 1.85E-07 |
| MAP2K7 | AL359076.1 | 0.40491552 | 1.73E-07 |
| MTMR14 | AL359076.1 | 0.32332477 | 4.07E-05 |
| NBR1 | AL359076.1 | 0.329136496 | 2.89E-05 |
| NKX2-3 | AL359076.1 | 0.367590591 | 2.54E-06 |
| PARP1 | AL359076.1 | 0.30702845 | 0.000101988 |
| PRKAB1 | AL359076.1 | 0.339279271 | 1.57E-05 |
| PTK6 | AL359076.1 | -0.387371088 | 6.37E-07 |
| TP53 | AL359076.1 | 0.395602606 | 3.49E-07 |
| TSC2 | AL359076.1 | 0.301478642 | 0.000137802 |
| WDR45 | AL359076.1 | 0.334883538 | 2.05E-05 |
| ARSB | LINC02031 | -0.311695809 | 7.88E-05 |
| ATG16L2 | LINC02031 | 0.309170327 | 9.07E-05 |
| ATG9B | LINC02031 | 0.367639899 | 2.53E-06 |
| BAG3 | LINC02031 | 0.305600588 | 0.000110263 |
| BID | LINC02031 | 0.368589382 | 2.37E-06 |
| FOXO1 | LINC02031 | -0.354412561 | 6.06E-06 |
| PTK6 | LINC02031 | 0.306324023 | 0.000105995 |
| RAB11A | LINC02031 | 0.337791736 | 1.72E-05 |
| ARNT | TRAPPC12-AS1 | 0.417909982 | 6.28E-08 |
| BECN1 | TRAPPC12-AS1 | 0.358683503 | 4.59E-06 |
| BIRC6 | TRAPPC12-AS1 | 0.479695603 | 2.70E-10 |
| CD46 | TRAPPC12-AS1 | 0.372334322 | 1.84E-06 |
| CDKN1B | TRAPPC12-AS1 | 0.335034991 | 2.03E-05 |
| EEF2K | TRAPPC12-AS1 | 0.43113066 | 2.14E-08 |
| EIF2AK3 | TRAPPC12-AS1 | 0.490671023 | 9.09E-11 |
| HDAC1 | TRAPPC12-AS1 | 0.395541386 | 3.51E-07 |
| HDAC6 | TRAPPC12-AS1 | 0.38373743 | 8.27E-07 |
| IKBKB | TRAPPC12-AS1 | 0.361796286 | 3.74E-06 |
| KLHL24 | TRAPPC12-AS1 | 0.322093693 | 4.37E-05 |
| MAPK8 | TRAPPC12-AS1 | 0.398872349 | 2.74E-07 |
| MTOR | TRAPPC12-AS1 | 0.425242593 | 3.48E-08 |
| NBR1 | TRAPPC12-AS1 | 0.42527786 | 3.47E-08 |
| PARP1 | TRAPPC12-AS1 | 0.395364735 | 3.55E-07 |
| PIK3R4 | TRAPPC12-AS1 | 0.375479692 | 1.48E-06 |
| RAB33B | TRAPPC12-AS1 | 0.347949788 | 9.15E-06 |
| RAF1 | TRAPPC12-AS1 | 0.351585499 | 7.26E-06 |
| RB1CC1 | TRAPPC12-AS1 | 0.327324973 | 3.22E-05 |
| SIRT1 | TRAPPC12-AS1 | 0.365894988 | 2.84E-06 |
| TP73 | TRAPPC12-AS1 | 0.331595369 | 2.50E-05 |
| TSC1 | TRAPPC12-AS1 | 0.401554313 | 2.24E-07 |
| TSC2 | TRAPPC12-AS1 | 0.3316298 | 2.49E-05 |
| UVRAG | TRAPPC12-AS1 | 0.301788534 | 0.000135527 |
| ATG16L2 | AL031600.1 | 0.347259719 | 9.56E-06 |
| EEF2K | AL031600.1 | 0.31803439 | 5.51E-05 |
| HDAC6 | AL031600.1 | 0.317672298 | 5.63E-05 |
| IKBKB | AL031600.1 | 0.31211926 | 7.70E-05 |
| MAP2K7 | AL031600.1 | 0.305206672 | 0.000112653 |
| SPNS1 | AL031600.1 | 0.457690917 | 2.13E-09 |
| TSC1 | AL031600.1 | 0.418006301 | 6.24E-08 |
| TSC2 | AL031600.1 | 0.315232564 | 6.46E-05 |
| VEGFA | AL031600.1 | 0.323524446 | 4.02E-05 |
| ARSB | AC040970.1 | 0.318755813 | 5.29E-05 |
| CXCR4 | AC040970.1 | 0.327450839 | 3.20E-05 |
| FOXO1 | AC040970.1 | 0.323307581 | 4.07E-05 |
| GAA | AC040970.1 | 0.471111405 | 6.15E-10 |
| RAB11A | AC040970.1 | -0.301080074 | 0.000140781 |
| RGS19 | AC040970.1 | 0.310039072 | 8.64E-05 |
| ATG10 | AC009005.1 | 0.371808855 | 1.90E-06 |
| ATG4C | AC009005.1 | 0.360762778 | 4.00E-06 |
| ATIC | AC009005.1 | 0.468209007 | 8.08E-10 |
| BAX | AC009005.1 | 0.383807845 | 8.23E-07 |
| BIRC5 | AC009005.1 | 0.363381902 | 3.36E-06 |
| CAPN10 | AC009005.1 | 0.494951287 | 5.89E-11 |
| CDKN1B | AC009005.1 | 0.324932958 | 3.71E-05 |
| DIRAS3 | AC009005.1 | 0.703315569 | 1.92E-24 |
| FKBP1B | AC009005.1 | 0.333343858 | 2.25E-05 |
| GABARAP | AC009005.1 | 0.351679194 | 7.22E-06 |
| HDAC6 | AC009005.1 | 0.400565258 | 2.41E-07 |
| ITGA3 | AC009005.1 | -0.313365571 | 7.18E-05 |
| MAP1LC3A | AC009005.1 | 0.366686539 | 2.70E-06 |
| MAPK8IP1 | AC009005.1 | 0.489802156 | 9.93E-11 |
| MLST8 | AC009005.1 | 0.376326965 | 1.39E-06 |
| MTMR14 | AC009005.1 | 0.320289953 | 4.85E-05 |
| PINK1 | AC009005.1 | -0.306429242 | 0.000105387 |
| PRKCQ | AC009005.1 | 0.300180985 | 0.000147721 |
| ULK3 | AC009005.1 | 0.31081245 | 8.28E-05 |
| WDR45 | AC009005.1 | 0.304622598 | 0.000116287 |
| ATG7 | AC114956.2 | -0.306331652 | 0.000105951 |
| BNIP1 | AC114956.2 | -0.340708 | 1.44E-05 |
| EGFR | AC114956.2 | 0.319047099 | 5.21E-05 |
| FAS | AC114956.2 | -0.32701751 | 3.28E-05 |
| HGS | AC114956.2 | 0.387316628 | 6.40E-07 |
| MTMR14 | AC114956.2 | -0.358195425 | 4.74E-06 |
| TP53 | AC114956.2 | -0.32365396 | 3.99E-05 |
| TP63 | AC114956.2 | 0.312813164 | 7.40E-05 |
| WIPI1 | AC114956.2 | 0.309284887 | 9.01E-05 |
| ATF4 | AL021707.8 | 0.361085491 | 3.92E-06 |
| ATG3 | AL021707.8 | 0.368145968 | 2.44E-06 |
| ATG4B | AL021707.8 | 0.342265307 | 1.31E-05 |
| BAX | AL021707.8 | 0.373648962 | 1.68E-06 |
| BNIP1 | AL021707.8 | 0.360768087 | 4.00E-06 |
| CAPN10 | AL021707.8 | 0.404695404 | 1.76E-07 |
| CDKN2A | AL021707.8 | 0.322651789 | 4.23E-05 |
| CXCR4 | AL021707.8 | 0.345526151 | 1.07E-05 |
| HDAC6 | AL021707.8 | 0.33721288 | 1.78E-05 |
| IKBKB | AL021707.8 | 0.313320876 | 7.20E-05 |
| RAB24 | AL021707.8 | 0.382578787 | 8.98E-07 |
| WDR45 | AL021707.8 | 0.436597245 | 1.35E-08 |
| ATG16L2 | AC135050.6 | 0.613405447 | 2.16E-17 |
| ATG3 | AC135050.6 | 0.303083656 | 0.000126393 |
| ATG4B | AC135050.6 | 0.492847423 | 7.30E-11 |
| ATG4C | AC135050.6 | 0.355811428 | 5.53E-06 |
| ATG4D | AC135050.6 | 0.325184336 | 3.65E-05 |
| BID | AC135050.6 | 0.312890715 | 7.37E-05 |
| CAPN10 | AC135050.6 | 0.622074972 | 5.66E-18 |
| CDKN1B | AC135050.6 | 0.365915975 | 2.84E-06 |
| CFLAR | AC135050.6 | 0.360684716 | 4.02E-06 |
| CLN3 | AC135050.6 | 0.437190526 | 1.29E-08 |
| CXCR4 | AC135050.6 | 0.437524058 | 1.25E-08 |
| DAPK2 | AC135050.6 | 0.385805061 | 7.13E-07 |
| FADD | AC135050.6 | -0.305812151 | 0.000108998 |
| HDAC6 | AC135050.6 | 0.423321093 | 4.07E-08 |
| IKBKB | AC135050.6 | 0.422476446 | 4.36E-08 |
| ITGA3 | AC135050.6 | -0.37077619 | 2.04E-06 |
| ITGA6 | AC135050.6 | -0.306508407 | 0.000104932 |
| LAMP1 | AC135050.6 | -0.300681737 | 0.000143817 |
| MAP1LC3A | AC135050.6 | 0.30486802 | 0.000114747 |
| MAP2K7 | AC135050.6 | 0.398187116 | 2.88E-07 |
| MLST8 | AC135050.6 | 0.41443663 | 8.28E-08 |
| MTMR14 | AC135050.6 | 0.376068869 | 1.42E-06 |
| PEX14 | AC135050.6 | 0.321477844 | 4.53E-05 |
| PRKAB1 | AC135050.6 | 0.390341942 | 5.14E-07 |
| RAB24 | AC135050.6 | 0.422118977 | 4.48E-08 |
| RAF1 | AC135050.6 | 0.376192965 | 1.41E-06 |
| STK11 | AC135050.6 | 0.412731465 | 9.46E-08 |
| TSC2 | AC135050.6 | 0.464488804 | 1.14E-09 |
| ULK3 | AC135050.6 | 0.540316693 | 3.98E-13 |
| WDR45 | AC135050.6 | 0.401808598 | 2.19E-07 |
| BAG3 | AC245041.2 | 0.305231854 | 0.000112499 |
| CASP1 | AC245041.2 | 0.30461004 | 0.000116367 |
| CASP4 | AC245041.2 | 0.308519499 | 9.40E-05 |
| ITGA3 | AC245041.2 | 0.387105779 | 6.50E-07 |
| ITGB4 | AC245041.2 | 0.339803654 | 1.52E-05 |
| NFKB1 | AC245041.2 | 0.376895298 | 1.34E-06 |
| RAC1 | AC245041.2 | 0.370695475 | 2.05E-06 |
| SAR1A | AC245041.2 | 0.305949864 | 0.000108183 |
| VAMP3 | AC245041.2 | 0.371277807 | 1.97E-06 |
| ATG12 | UBA6-AS1 | 0.33260299 | 2.35E-05 |
| ARNT | LINC01703 | 0.378965106 | 1.16E-06 |
| ATF6 | LINC01703 | 0.342769474 | 1.27E-05 |
| CASP4 | LINC01703 | -0.317140318 | 5.80E-05 |
| CDKN1A | LINC01703 | -0.381296838 | 9.84E-07 |
| DAPK1 | LINC01703 | 0.326078834 | 3.46E-05 |
| EEF2 | LINC01703 | 0.341872525 | 1.34E-05 |
| GAA | LINC01703 | 0.354894277 | 5.87E-06 |
| GABARAPL1 | LINC01703 | 0.371210346 | 1.98E-06 |
| GNAI3 | LINC01703 | -0.373488318 | 1.70E-06 |
| ITGA3 | LINC01703 | -0.354621062 | 5.97E-06 |
| ITGB4 | LINC01703 | -0.337356075 | 1.77E-05 |
| KLHL24 | LINC01703 | 0.464804324 | 1.11E-09 |
| MBTPS2 | LINC01703 | 0.418489489 | 6.00E-08 |
| NBR1 | LINC01703 | 0.382290823 | 9.17E-07 |
| PARP1 | LINC01703 | 0.3941328 | 3.89E-07 |
| PTK6 | LINC01703 | -0.355282664 | 5.72E-06 |
| SQSTM1 | LINC01703 | 0.375530308 | 1.47E-06 |
| ST13 | LINC01703 | 0.301437694 | 0.000138106 |
| ULK2 | LINC01703 | 0.380191137 | 1.06E-06 |
| UVRAG | LINC01703 | 0.358761248 | 4.56E-06 |
| ARNT | AC079807.1 | 0.407998179 | 1.37E-07 |
| ATF6 | AC079807.1 | 0.323465328 | 4.04E-05 |
| BECN1 | AC079807.1 | 0.388162192 | 6.02E-07 |
| BIRC6 | AC079807.1 | 0.629793617 | 1.66E-18 |
| BNIP1 | AC079807.1 | -0.304753307 | 0.000115465 |
| CAMKK2 | AC079807.1 | 0.315177499 | 6.48E-05 |
| CASP8 | AC079807.1 | 0.30185727 | 0.000135028 |
| CD46 | AC079807.1 | 0.32050573 | 4.79E-05 |
| EDEM1 | AC079807.1 | 0.321422537 | 4.54E-05 |
| EIF2AK2 | AC079807.1 | 0.323795921 | 3.96E-05 |
| EIF2AK3 | AC079807.1 | 0.545963679 | 2.03E-13 |
| EIF4G1 | AC079807.1 | 0.379774761 | 1.10E-06 |
| ERN1 | AC079807.1 | 0.401659284 | 2.22E-07 |
| FOXO3 | AC079807.1 | 0.455676874 | 2.56E-09 |
| GABARAPL1 | AC079807.1 | 0.365885144 | 2.84E-06 |
| HGS | AC079807.1 | 0.337981862 | 1.70E-05 |
| KIF5B | AC079807.1 | 0.36749622 | 2.55E-06 |
| KLHL24 | AC079807.1 | 0.428250949 | 2.72E-08 |
| LAMP2 | AC079807.1 | 0.309402219 | 8.95E-05 |
| MBTPS2 | AC079807.1 | 0.387061221 | 6.52E-07 |
| NAF1 | AC079807.1 | 0.304789248 | 0.00011524 |
| NBR1 | AC079807.1 | 0.526474806 | 1.98E-12 |
| NCKAP1 | AC079807.1 | 0.469548172 | 7.12E-10 |
| NFE2L2 | AC079807.1 | 0.516791165 | 5.83E-12 |
| PARP1 | AC079807.1 | 0.300753142 | 0.000143269 |
| PEX14 | AC079807.1 | -0.363868205 | 3.26E-06 |
| PIK3R4 | AC079807.1 | 0.392377249 | 4.43E-07 |
| PRKCD | AC079807.1 | 0.309151958 | 9.07E-05 |
| RAB1A | AC079807.1 | 0.308924098 | 9.19E-05 |
| RAB5A | AC079807.1 | 0.386290511 | 6.89E-07 |
| RB1CC1 | AC079807.1 | 0.464306915 | 1.16E-09 |
| RPS6KB1 | AC079807.1 | 0.399632363 | 2.59E-07 |
| SIRT1 | AC079807.1 | 0.314778513 | 6.63E-05 |
| SPNS1 | AC079807.1 | 0.45873582 | 1.94E-09 |
| TP53INP2 | AC079807.1 | 0.32958158 | 2.82E-05 |
| TP63 | AC079807.1 | 0.396443447 | 3.28E-07 |
| TSC1 | AC079807.1 | 0.32611565 | 3.46E-05 |
| USP10 | AC079807.1 | 0.436401206 | 1.38E-08 |
| UVRAG | AC079807.1 | 0.404421286 | 1.80E-07 |
| VAMP7 | AC079807.1 | 0.340319272 | 1.47E-05 |
| WDFY3 | AC079807.1 | 0.4373294 | 1.27E-08 |
| DIRAS3 | LINC02315 | 0.385777015 | 7.15E-07 |
| ATG10 | PART1 | 0.312494825 | 7.54E-05 |
| ATG4D | PART1 | 0.320313394 | 4.84E-05 |
| ATG7 | PART1 | 0.323100316 | 4.12E-05 |
| CAPNS1 | PART1 | 0.460233277 | 1.69E-09 |
| CDKN2A | PART1 | 0.465575412 | 1.03E-09 |
| IKBKE | PART1 | 0.303444299 | 0.000123954 |
| MTMR14 | PART1 | 0.324221285 | 3.86E-05 |
| ATF4 | TMEM161B-AS1 | 0.385399301 | 7.35E-07 |
| ATG10 | TMEM161B-AS1 | 0.45642964 | 2.39E-09 |
| ATG12 | TMEM161B-AS1 | 0.472151406 | 5.57E-10 |
| ATG16L2 | TMEM161B-AS1 | 0.344751949 | 1.12E-05 |
| ATG2B | TMEM161B-AS1 | -0.305658826 | 0.000109913 |
| ATG4B | TMEM161B-AS1 | 0.479390338 | 2.78E-10 |
| ATG4C | TMEM161B-AS1 | 0.469163363 | 7.39E-10 |
| ATG4D | TMEM161B-AS1 | 0.372003052 | 1.88E-06 |
| ATG7 | TMEM161B-AS1 | 0.332036018 | 2.44E-05 |
| BAG3 | TMEM161B-AS1 | -0.406924708 | 1.49E-07 |
| BAX | TMEM161B-AS1 | 0.477198317 | 3.44E-10 |
| BCL2 | TMEM161B-AS1 | 0.402588351 | 2.07E-07 |
| BNIP1 | TMEM161B-AS1 | 0.600162584 | 1.54E-16 |
| CAPN10 | TMEM161B-AS1 | 0.619409166 | 8.58E-18 |
| CDKN1B | TMEM161B-AS1 | 0.307843092 | 9.75E-05 |
| CDKN2A | TMEM161B-AS1 | 0.315134218 | 6.50E-05 |
| CXCR4 | TMEM161B-AS1 | 0.435277555 | 1.51E-08 |
| DAPK2 | TMEM161B-AS1 | 0.321427418 | 4.54E-05 |
| DIRAS3 | TMEM161B-AS1 | 0.319620638 | 5.04E-05 |
| EIF4G1 | TMEM161B-AS1 | -0.400978182 | 2.34E-07 |
| FAS | TMEM161B-AS1 | 0.327561989 | 3.18E-05 |
| FKBP1A | TMEM161B-AS1 | 0.323882425 | 3.94E-05 |
| GABARAP | TMEM161B-AS1 | 0.400557897 | 2.41E-07 |
| HDAC6 | TMEM161B-AS1 | 0.380613278 | 1.03E-06 |
| HIF1A | TMEM161B-AS1 | -0.37430284 | 1.60E-06 |
| IKBKE | TMEM161B-AS1 | 0.313400214 | 7.17E-05 |
| ITGA6 | TMEM161B-AS1 | -0.336423616 | 1.87E-05 |
| ITGB4 | TMEM161B-AS1 | -0.320855881 | 4.69E-05 |
| MAP1LC3A | TMEM161B-AS1 | 0.345982716 | 1.04E-05 |
| MAP2K7 | TMEM161B-AS1 | 0.362517466 | 3.56E-06 |
| MAPK1 | TMEM161B-AS1 | -0.350777902 | 7.65E-06 |
| MAPK8 | TMEM161B-AS1 | 0.35133721 | 7.38E-06 |
| MLST8 | TMEM161B-AS1 | 0.301441786 | 0.000138075 |
| MTMR14 | TMEM161B-AS1 | 0.434748611 | 1.58E-08 |
| NCKAP1 | TMEM161B-AS1 | -0.406502345 | 1.53E-07 |
| NKX2-3 | TMEM161B-AS1 | 0.386455024 | 6.81E-07 |
| NPC1 | TMEM161B-AS1 | -0.300686463 | 0.000143781 |
| PELP1 | TMEM161B-AS1 | 0.317404426 | 5.72E-05 |
| PEX14 | TMEM161B-AS1 | 0.425393983 | 3.44E-08 |
| RAB24 | TMEM161B-AS1 | 0.422742344 | 4.26E-08 |
| STK11 | TMEM161B-AS1 | 0.324510077 | 3.80E-05 |
| TP53 | TMEM161B-AS1 | 0.304467412 | 0.000117271 |
| TP63 | TMEM161B-AS1 | -0.351388781 | 7.35E-06 |
| TP73 | TMEM161B-AS1 | 0.319116114 | 5.18E-05 |
| TSC2 | TMEM161B-AS1 | 0.432558618 | 1.90E-08 |
| ULK3 | TMEM161B-AS1 | 0.372483237 | 1.82E-06 |
| USP10 | TMEM161B-AS1 | -0.392196914 | 4.49E-07 |
| WDFY3 | TMEM161B-AS1 | -0.340539073 | 1.45E-05 |
| WDR45 | TMEM161B-AS1 | 0.505518228 | 1.96E-11 |
| ATG10 | PSMA3-AS1 | 0.313736042 | 7.03E-05 |
| ATG12 | PSMA3-AS1 | 0.303170553 | 0.000125802 |
| ATG16L2 | PSMA3-AS1 | 0.38775976 | 6.20E-07 |
| ATG3 | PSMA3-AS1 | 0.360005156 | 4.21E-06 |
| ATG4B | PSMA3-AS1 | 0.380017534 | 1.08E-06 |
| ATG4C | PSMA3-AS1 | 0.403333136 | 1.95E-07 |
| BAX | PSMA3-AS1 | 0.330306341 | 2.70E-05 |
| CAPN10 | PSMA3-AS1 | 0.468527752 | 7.84E-10 |
| CD46 | PSMA3-AS1 | 0.312413209 | 7.57E-05 |
| CDKN1B | PSMA3-AS1 | 0.421265538 | 4.80E-08 |
| CDKN2A | PSMA3-AS1 | 0.317075493 | 5.82E-05 |
| CX3CL1 | PSMA3-AS1 | 0.360115823 | 4.17E-06 |
| CXCR4 | PSMA3-AS1 | 0.524139935 | 2.58E-12 |
| DAPK2 | PSMA3-AS1 | 0.40324959 | 1.97E-07 |
| EEF2K | PSMA3-AS1 | 0.307128957 | 0.000101428 |
| HDAC6 | PSMA3-AS1 | 0.504466583 | 2.19E-11 |
| IKBKB | PSMA3-AS1 | 0.456864747 | 2.30E-09 |
| MAP1LC3B | PSMA3-AS1 | -0.338481302 | 1.65E-05 |
| MAP2K7 | PSMA3-AS1 | 0.410380537 | 1.14E-07 |
| MTMR14 | PSMA3-AS1 | 0.37771848 | 1.26E-06 |
| NKX2-3 | PSMA3-AS1 | 0.337767266 | 1.72E-05 |
| P4HB | PSMA3-AS1 | -0.326506042 | 3.38E-05 |
| PINK1 | PSMA3-AS1 | -0.338594371 | 1.64E-05 |
| PRKAB1 | PSMA3-AS1 | 0.3172984 | 5.75E-05 |
| RAF1 | PSMA3-AS1 | 0.457509788 | 2.17E-09 |
| STK11 | PSMA3-AS1 | 0.345159767 | 1.09E-05 |
| TP53 | PSMA3-AS1 | 0.364332951 | 3.16E-06 |
| TP73 | PSMA3-AS1 | 0.368231616 | 2.43E-06 |
| TSC1 | PSMA3-AS1 | 0.408913989 | 1.27E-07 |
| TSC2 | PSMA3-AS1 | 0.45494492 | 2.73E-09 |
| ULK3 | PSMA3-AS1 | 0.51413316 | 7.79E-12 |
| WDR45 | PSMA3-AS1 | 0.419576976 | 5.50E-08 |
| ATF4 | AC127024.5 | 0.302768715 | 0.00012856 |
| ATG16L2 | AC127024.5 | 0.37116521 | 1.99E-06 |
| ATG3 | AC127024.5 | 0.316937669 | 5.87E-05 |
| ATG4B | AC127024.5 | 0.399690562 | 2.57E-07 |
| ATG4D | AC127024.5 | 0.332465083 | 2.37E-05 |
| CAPN10 | AC127024.5 | 0.500021285 | 3.49E-11 |
| CASP3 | AC127024.5 | 0.33785914 | 1.71E-05 |
| CDKN1B | AC127024.5 | 0.345572099 | 1.06E-05 |
| CXCR4 | AC127024.5 | 0.409506305 | 1.22E-07 |
| DAPK2 | AC127024.5 | 0.410461231 | 1.13E-07 |
| HDAC6 | AC127024.5 | 0.424455176 | 3.71E-08 |
| IKBKB | AC127024.5 | 0.407777405 | 1.39E-07 |
| ITGA3 | AC127024.5 | -0.316951572 | 5.87E-05 |
| MAP2K7 | AC127024.5 | 0.431472398 | 2.08E-08 |
| PELP1 | AC127024.5 | 0.420232104 | 5.22E-08 |
| PINK1 | AC127024.5 | -0.420417741 | 5.14E-08 |
| PTK6 | AC127024.5 | -0.315146387 | 6.50E-05 |
| RAF1 | AC127024.5 | 0.381710404 | 9.55E-07 |
| RHEB | AC127024.5 | 0.332983762 | 2.30E-05 |
| STK11 | AC127024.5 | 0.361964276 | 3.69E-06 |
| TSC1 | AC127024.5 | 0.324443114 | 3.81E-05 |
| ULK3 | AC127024.5 | 0.442640821 | 8.07E-09 |
| WDR45 | AC127024.5 | 0.401952931 | 2.17E-07 |
| ATG16L2 | AC015726.1 | 0.347562326 | 9.38E-06 |
| ATG4B | AC015726.1 | 0.44587886 | 6.09E-09 |
| CAPN10 | AC015726.1 | 0.459260358 | 1.85E-09 |
| CXCR4 | AC015726.1 | 0.384599238 | 7.78E-07 |
| DAPK2 | AC015726.1 | 0.429805874 | 2.39E-08 |
| GABARAP | AC015726.1 | 0.423616337 | 3.97E-08 |
| HDAC6 | AC015726.1 | 0.389157023 | 5.60E-07 |
| IKBKB | AC015726.1 | 0.31993935 | 4.95E-05 |
| MAP2K7 | AC015726.1 | 0.373185034 | 1.73E-06 |
| PELP1 | AC015726.1 | 0.464010057 | 1.19E-09 |
| TSC2 | AC015726.1 | 0.352084784 | 7.03E-06 |
| ULK3 | AC015726.1 | 0.307556713 | 9.91E-05 |
| WDR45 | AC015726.1 | 0.458609245 | 1.96E-09 |
| BAK1 | AC093388.1 | 0.3072113 | 0.000100972 |
| CASP1 | AC093388.1 | 0.392639611 | 4.35E-07 |
| CASP4 | LNCAROD | 0.344100144 | 1.17E-05 |
| IL24 | LNCAROD | 0.368908025 | 2.32E-06 |
| ITGA3 | LNCAROD | 0.44080209 | 9.46E-09 |
| ITGA6 | LNCAROD | 0.408881975 | 1.28E-07 |
| ITGB4 | LNCAROD | 0.327987824 | 3.10E-05 |
| NRG1 | LNCAROD | 0.343110321 | 1.24E-05 |
| SAR1A | LNCAROD | 0.373353109 | 1.71E-06 |
| ATF4 | AC010969.2 | 0.461538008 | 1.50E-09 |
| ATG2B | AC010969.2 | -0.357459829 | 4.97E-06 |
| ATG4B | AC010969.2 | 0.609063318 | 4.16E-17 |
| BAG1 | AC010969.2 | 0.351803731 | 7.16E-06 |
| BAX | AC010969.2 | 0.523228708 | 2.85E-12 |
| BIRC5 | AC010969.2 | 0.430409946 | 2.27E-08 |
| BNIP1 | AC010969.2 | 0.573294497 | 6.39E-15 |
| CANX | AC010969.2 | -0.315820806 | 6.25E-05 |
| CAPN10 | AC010969.2 | 0.504212263 | 2.25E-11 |
| CDKN2A | AC010969.2 | 0.411095328 | 1.08E-07 |
| CHMP4B | AC010969.2 | 0.361232365 | 3.88E-06 |
| CLN3 | AC010969.2 | 0.329245994 | 2.88E-05 |
| DAPK2 | AC010969.2 | 0.331256097 | 2.55E-05 |
| DLC1 | AC010969.2 | -0.347042179 | 9.69E-06 |
| EIF4G1 | AC010969.2 | -0.365910699 | 2.84E-06 |
| FKBP1A | AC010969.2 | 0.337144953 | 1.79E-05 |
| HIF1A | AC010969.2 | -0.36689666 | 2.66E-06 |
| ITGB1 | AC010969.2 | -0.327289568 | 3.23E-05 |
| KIF5B | AC010969.2 | -0.347028395 | 9.70E-06 |
| LAMP2 | AC010969.2 | -0.305857714 | 0.000108728 |
| MAP1LC3A | AC010969.2 | 0.386422203 | 6.83E-07 |
| MAP2K7 | AC010969.2 | 0.305301208 | 0.000112075 |
| MAPK1 | AC010969.2 | -0.381506874 | 9.69E-07 |
| MLST8 | AC010969.2 | 0.309544644 | 8.88E-05 |
| MTMR14 | AC010969.2 | 0.384441667 | 7.87E-07 |
| NCKAP1 | AC010969.2 | -0.427798788 | 2.82E-08 |
| PELP1 | AC010969.2 | 0.301039456 | 0.000141088 |
| PEX14 | AC010969.2 | 0.439565647 | 1.05E-08 |
| PINK1 | AC010969.2 | -0.320840537 | 4.70E-05 |
| RAB24 | AC010969.2 | 0.416025352 | 7.30E-08 |
| STK11 | AC010969.2 | 0.602231447 | 1.14E-16 |
| TP63 | AC010969.2 | -0.301162479 | 0.00014016 |
| USP10 | AC010969.2 | -0.355024562 | 5.82E-06 |
| WDFY3 | AC010969.2 | -0.38265213 | 8.94E-07 |
| WDR45 | AC010969.2 | 0.466555359 | 9.43E-10 |
| ATG16L2 | TONSL-AS1 | 0.427402728 | 2.91E-08 |
| BCL2L1 | TONSL-AS1 | 0.473286639 | 5.00E-10 |
| BID | TONSL-AS1 | 0.308629573 | 9.34E-05 |
| CAPN10 | TONSL-AS1 | 0.327560209 | 3.18E-05 |
| DIRAS3 | TONSL-AS1 | 0.338645615 | 1.63E-05 |
| EGFR | AP002884.1 | 0.428145814 | 2.74E-08 |
| ATG16L2 | AC114296.1 | 0.363531937 | 3.33E-06 |
| BID | AC114296.1 | 0.401646688 | 2.22E-07 |
| DIRAS3 | AC114296.1 | 0.378931219 | 1.16E-06 |
| ATG16L2 | AC005393.1 | 0.339437635 | 1.56E-05 |
| ATG4B | AC005393.1 | 0.411036759 | 1.08E-07 |
| ATG4D | AC005393.1 | 0.372046731 | 1.87E-06 |
| CAPN10 | AC005393.1 | 0.366817058 | 2.67E-06 |
| DAPK2 | AC005393.1 | 0.477551714 | 3.32E-10 |
| GABARAP | AC005393.1 | 0.33143263 | 2.52E-05 |
| HDAC6 | AC005393.1 | 0.311690112 | 7.88E-05 |
| IKBKB | AC005393.1 | 0.301396488 | 0.000138412 |
| MAP2K7 | AC005393.1 | 0.362226359 | 3.63E-06 |
| PELP1 | AC005393.1 | 0.509179603 | 1.33E-11 |
| PINK1 | AC005393.1 | -0.323403096 | 4.05E-05 |
| WDR45 | AC005393.1 | 0.331784761 | 2.47E-05 |
| ARSB | AC134312.5 | 0.331453449 | 2.52E-05 |
| DLC1 | AC134312.5 | 0.414724637 | 8.09E-08 |
| PEA15 | AC134312.5 | 0.340547612 | 1.45E-05 |
| RAB11A | AC134312.5 | -0.306281477 | 0.000106242 |
| ATG7 | LINC01943 | 0.330560082 | 2.66E-05 |
| BAX | LINC01943 | 0.506626681 | 1.75E-11 |
| CAPN10 | LINC01943 | 0.316747706 | 5.93E-05 |
| CAPNS1 | LINC01943 | 0.328448676 | 3.01E-05 |
| CCL2 | LINC01943 | 0.38912573 | 5.61E-07 |
| CCR2 | LINC01943 | 0.607275499 | 5.43E-17 |
| CTSD | LINC01943 | 0.366002392 | 2.82E-06 |
| CXCR4 | LINC01943 | 0.489629462 | 1.01E-10 |
| DRAM1 | LINC01943 | 0.379064053 | 1.15E-06 |
| GABARAP | LINC01943 | 0.396723968 | 3.21E-07 |
| IFNG | LINC01943 | 0.601946094 | 1.19E-16 |
| ITPR1 | LINC01943 | 0.307179654 | 0.000101147 |
| MTMR14 | LINC01943 | 0.473299116 | 4.99E-10 |
| MYC | LINC01943 | -0.342370522 | 1.30E-05 |
| NCKAP1 | LINC01943 | -0.343570131 | 1.20E-05 |
| RAB24 | LINC01943 | 0.302960567 | 0.000127236 |
| RGS19 | LINC01943 | 0.520866974 | 3.71E-12 |
| SERPINA1 | LINC01943 | 0.412496377 | 9.64E-08 |
| STK11 | LINC01943 | 0.343286448 | 1.23E-05 |
| WDFY3 | LINC01943 | -0.311754881 | 7.86E-05 |
| BNIP3 | AC084117.1 | 0.323612545 | 4.00E-05 |
| PPP1R15A | AC084117.1 | 0.336786771 | 1.83E-05 |
| RAB24 | AC084117.1 | 0.348582563 | 8.79E-06 |
| VEGFA | AC084117.1 | 0.545804403 | 2.06E-13 |
| ARSA | CHKB-DT | 0.367553682 | 2.54E-06 |
| ATG4C | IPO5P1 | 0.368212955 | 2.43E-06 |
| BCL2 | IPO5P1 | 0.540619477 | 3.84E-13 |
| CASP3 | IPO5P1 | 0.368779506 | 2.34E-06 |
| CDKN1B | IPO5P1 | 0.340622528 | 1.45E-05 |
| HDAC6 | IPO5P1 | 0.421009387 | 4.90E-08 |
| KLHL24 | IPO5P1 | 0.347209672 | 9.59E-06 |
| MAPK8 | IPO5P1 | 0.349437109 | 8.33E-06 |
| PIK3R4 | IPO5P1 | 0.300067975 | 0.000148616 |
| RAF1 | IPO5P1 | 0.361489254 | 3.81E-06 |
| WDR45 | IPO5P1 | 0.330874415 | 2.61E-05 |
| ATF4 | AC005726.3 | 0.374693751 | 1.56E-06 |
| ATG16L2 | AC005726.3 | 0.395379325 | 3.55E-07 |
| ATG3 | AC005726.3 | 0.310875557 | 8.25E-05 |
| ATG4B | AC005726.3 | 0.563894813 | 2.18E-14 |
| BAG1 | AC005726.3 | 0.322833792 | 4.19E-05 |
| BAX | AC005726.3 | 0.391857959 | 4.60E-07 |
| BNIP1 | AC005726.3 | 0.345723554 | 1.05E-05 |
| CAPN10 | AC005726.3 | 0.540651479 | 3.83E-13 |
| CXCR4 | AC005726.3 | 0.429362425 | 2.48E-08 |
| DAPK2 | AC005726.3 | 0.483837577 | 1.80E-10 |
| KIF5B | AC005726.3 | -0.312329511 | 7.61E-05 |
| MAP2K7 | AC005726.3 | 0.350631429 | 7.72E-06 |
| NCKAP1 | AC005726.3 | -0.308155125 | 9.59E-05 |
| PELP1 | AC005726.3 | 0.382408785 | 9.09E-07 |
| PINK1 | AC005726.3 | -0.391207893 | 4.83E-07 |
| RAB24 | AC005726.3 | 0.309158887 | 9.07E-05 |
| STK11 | AC005726.3 | 0.420673965 | 5.04E-08 |
| ULK3 | AC005726.3 | 0.318473666 | 5.38E-05 |
| WDFY3 | AC005726.3 | -0.361626127 | 3.78E-06 |
| WDR45 | AC005726.3 | 0.401465537 | 2.25E-07 |
| ARNT | SNHG5 | -0.316803322 | 5.91E-05 |
| ATF4 | SNHG5 | 0.468196386 | 8.09E-10 |
| ATG2B | SNHG5 | -0.319583544 | 5.05E-05 |
| BAX | SNHG5 | 0.359756935 | 4.27E-06 |
| BNIP1 | SNHG5 | 0.333449024 | 2.24E-05 |
| CAPN10 | SNHG5 | 0.413515488 | 8.90E-08 |
| DDIT3 | SNHG5 | 0.374192489 | 1.62E-06 |
| DIRAS3 | SNHG5 | 0.385531693 | 7.28E-07 |
| FOXO1 | SNHG5 | -0.300774047 | 0.000143108 |
| LAMP2 | SNHG5 | -0.338664952 | 1.63E-05 |
| MAPK1 | SNHG5 | -0.346406956 | 1.01E-05 |
| NCKAP1 | SNHG5 | -0.309587214 | 8.86E-05 |
| ARNT | AC091057.1 | 0.350119027 | 7.98E-06 |
| ATG10 | AC091057.1 | 0.419910563 | 5.36E-08 |
| ATG12 | AC091057.1 | 0.395162528 | 3.61E-07 |
| ATG2A | AC091057.1 | 0.363011044 | 3.45E-06 |
| ATG3 | AC091057.1 | 0.387409643 | 6.36E-07 |
| ATG4A | AC091057.1 | 0.311213719 | 8.10E-05 |
| ATG4C | AC091057.1 | 0.465437524 | 1.05E-09 |
| ATG7 | AC091057.1 | 0.361971872 | 3.69E-06 |
| BCL2 | AC091057.1 | 0.408540622 | 1.31E-07 |
| BECN1 | AC091057.1 | 0.310410119 | 8.46E-05 |
| CALCOCO2 | AC091057.1 | 0.339832703 | 1.52E-05 |
| CASP3 | AC091057.1 | 0.375400134 | 1.49E-06 |
| CD46 | AC091057.1 | 0.429941066 | 2.36E-08 |
| CDKN1B | AC091057.1 | 0.466783077 | 9.23E-10 |
| CDKN2A | AC091057.1 | 0.366232825 | 2.78E-06 |
| CFLAR | AC091057.1 | 0.336328483 | 1.88E-05 |
| CHMP4B | AC091057.1 | 0.302816977 | 0.000128226 |
| CXCR4 | AC091057.1 | 0.31042849 | 8.46E-05 |
| EEF2K | AC091057.1 | 0.442238632 | 8.36E-09 |
| FAS | AC091057.1 | 0.443340751 | 7.60E-09 |
| HDAC1 | AC091057.1 | 0.369427692 | 2.24E-06 |
| HDAC6 | AC091057.1 | 0.5910588 | 5.67E-16 |
| IFNG | AC091057.1 | 0.344420982 | 1.14E-05 |
| IKBKB | AC091057.1 | 0.372439608 | 1.82E-06 |
| IKBKE | AC091057.1 | 0.319899125 | 4.96E-05 |
| KLHL24 | AC091057.1 | 0.305801321 | 0.000109063 |
| MAP2K7 | AC091057.1 | 0.486354622 | 1.40E-10 |
| MAPK8 | AC091057.1 | 0.486779978 | 1.34E-10 |
| MAPK9 | AC091057.1 | 0.399690786 | 2.57E-07 |
| MTMR14 | AC091057.1 | 0.442178738 | 8.40E-09 |
| MTOR | AC091057.1 | 0.373893958 | 1.65E-06 |
| NFKB1 | AC091057.1 | 0.346349747 | 1.01E-05 |
| NKX2-3 | AC091057.1 | 0.491881104 | 8.05E-11 |
| P4HB | AC091057.1 | -0.304938352 | 0.00011431 |
| PARP1 | AC091057.1 | 0.450067487 | 4.22E-09 |
| PIK3C3 | AC091057.1 | 0.315231727 | 6.46E-05 |
| PIK3R4 | AC091057.1 | 0.465594167 | 1.03E-09 |
| PRKAB1 | AC091057.1 | 0.364796803 | 3.06E-06 |
| PTK6 | AC091057.1 | -0.30708808 | 0.000101656 |
| RAB33B | AC091057.1 | 0.464581136 | 1.13E-09 |
| RAF1 | AC091057.1 | 0.604259662 | 8.48E-17 |
| SH3GLB1 | AC091057.1 | 0.305406621 | 0.000111434 |
| SIRT1 | AC091057.1 | 0.452658849 | 3.35E-09 |
| STK11 | AC091057.1 | 0.461850954 | 1.46E-09 |
| TP53 | AC091057.1 | 0.437216377 | 1.28E-08 |
| TP73 | AC091057.1 | 0.546646271 | 1.87E-13 |
| TSC1 | AC091057.1 | 0.482398371 | 2.07E-10 |
| TSC2 | AC091057.1 | 0.457293255 | 2.21E-09 |
| ULK2 | AC091057.1 | 0.311105482 | 8.14E-05 |
| ULK3 | AC091057.1 | 0.417477263 | 6.51E-08 |
| ARSB | AC023157.3 | -0.358686884 | 4.59E-06 |
| ATG16L2 | AC023157.3 | 0.306571263 | 0.000104572 |
| ATG9B | AC023157.3 | 0.363941321 | 3.24E-06 |
| FKBP1B | AC023157.3 | 0.32699303 | 3.28E-05 |
| FOXO1 | AC023157.3 | -0.420542927 | 5.09E-08 |
| MYC | AC023157.3 | 0.375990297 | 1.43E-06 |
| PPP1R15A | AC023157.3 | 0.387668672 | 6.24E-07 |
| PTK6 | AC023157.3 | 0.368712929 | 2.35E-06 |
| FOXO1 | AL391422.4 | 0.358645178 | 4.60E-06 |
| GAA | AL391422.4 | 0.39501327 | 3.65E-07 |
| ATIC | AL512598.1 | 0.356504837 | 5.29E-06 |
| DIRAS3 | AL512598.1 | 0.584031108 | 1.50E-15 |
| FKBP1B | AL512598.1 | 0.362495123 | 3.57E-06 |
| GABARAPL2 | AL512598.1 | 0.342381504 | 1.30E-05 |
| MAPK8IP1 | AL512598.1 | 0.383627564 | 8.34E-07 |
| PRKCQ | AL512598.1 | 0.376584757 | 1.37E-06 |
| FOXO3 | AC007249.1 | 0.317545294 | 5.67E-05 |
| GABARAPL1 | AC007249.1 | 0.458315915 | 2.01E-09 |
| NFE2L2 | AC007249.1 | 0.382629738 | 8.95E-07 |
| PELP1 | AC007249.1 | 0.329684331 | 2.80E-05 |
| PIK3C3 | AC007249.1 | 0.350998522 | 7.54E-06 |
| PINK1 | AC007249.1 | -0.329033224 | 2.91E-05 |
| TP63 | AC007249.1 | 0.390959506 | 4.91E-07 |
| UVRAG | AC007249.1 | 0.377114974 | 1.32E-06 |
| VAMP3 | AC007249.1 | -0.368074074 | 2.45E-06 |
| ATG4D | AL365356.5 | -0.303413118 | 0.000124163 |
| BAK1 | AL365356.5 | 0.358354809 | 4.69E-06 |
| BCL2 | AL365356.5 | -0.315476617 | 6.38E-05 |
| CASP4 | AL365356.5 | 0.382955432 | 8.75E-07 |
| CDKN1B | AL365356.5 | -0.327766554 | 3.14E-05 |
| EEF2 | AL365356.5 | -0.303584722 | 0.000123016 |
| EEF2K | AL365356.5 | -0.337730677 | 1.73E-05 |
| EIF2AK3 | AL365356.5 | -0.388192611 | 6.01E-07 |
| HDAC6 | AL365356.5 | -0.332972041 | 2.30E-05 |
| ITGA3 | AL365356.5 | 0.500717584 | 3.25E-11 |
| ITGA6 | AL365356.5 | 0.346623582 | 9.95E-06 |
| ITGB4 | AL365356.5 | 0.330039025 | 2.74E-05 |
| KLHL24 | AL365356.5 | -0.327904547 | 3.11E-05 |
| NBR1 | AL365356.5 | -0.32701972 | 3.28E-05 |
| NFE2L2 | AL365356.5 | -0.313832175 | 6.99E-05 |
| PARP1 | AL365356.5 | -0.40824229 | 1.34E-07 |
| PIK3R4 | AL365356.5 | -0.308871931 | 9.22E-05 |
| PTK6 | AL365356.5 | 0.351932623 | 7.10E-06 |
| SIRT1 | AL365356.5 | -0.302217284 | 0.000132438 |
| ARSB | AL591845.1 | -0.305567063 | 0.000110464 |
| ATG16L2 | AL591845.1 | 0.372504014 | 1.81E-06 |
| BIRC6 | AL591845.1 | -0.360351294 | 4.11E-06 |
| FOXO1 | AL591845.1 | -0.316968847 | 5.86E-05 |
| GABARAPL2 | AL591845.1 | 0.344455788 | 1.14E-05 |
| MBTPS2 | AL591845.1 | -0.333597914 | 2.22E-05 |
| RAB24 | AL591845.1 | 0.358693111 | 4.58E-06 |
| VEGFA | AL591845.1 | 0.380186865 | 1.06E-06 |
| ATF6 | SAMD12-AS1 | 0.301981267 | 0.00013413 |
| ATG4D | SAMD12-AS1 | 0.300898547 | 0.000142157 |
| GABARAPL1 | SAMD12-AS1 | 0.549655719 | 1.29E-13 |
| GAPDH | SAMD12-AS1 | 0.318362403 | 5.41E-05 |
| GNAI3 | SAMD12-AS1 | -0.305420418 | 0.00011135 |
| KLHL24 | SAMD12-AS1 | 0.334772844 | 2.07E-05 |
| NAMPT | SAMD12-AS1 | 0.347245368 | 9.57E-06 |
| NCKAP1 | SAMD12-AS1 | 0.347521533 | 9.40E-06 |
| NFE2L2 | SAMD12-AS1 | 0.455427772 | 2.61E-09 |
| RB1CC1 | SAMD12-AS1 | 0.326957865 | 3.29E-05 |
| RHEB | SAMD12-AS1 | 0.327715495 | 3.15E-05 |
| SQSTM1 | SAMD12-AS1 | 0.329590289 | 2.82E-05 |
| TP63 | SAMD12-AS1 | 0.412806465 | 9.41E-08 |
| UVRAG | SAMD12-AS1 | 0.389314169 | 5.54E-07 |
| VAMP3 | SAMD12-AS1 | -0.324155464 | 3.88E-05 |
| WIPI1 | SAMD12-AS1 | 0.365901026 | 2.84E-06 |
| ATG5 | AC019205.1 | 0.410389686 | 1.14E-07 |
| CASP8 | AC019205.1 | 0.344020359 | 1.17E-05 |
| TSC1 | AC019205.1 | 0.325262646 | 3.63E-05 |
| ATF4 | SCAMP1-AS1 | 0.329843868 | 2.77E-05 |
| ATG10 | SCAMP1-AS1 | 0.343682554 | 1.20E-05 |
| ATG12 | SCAMP1-AS1 | 0.321718214 | 4.46E-05 |
| BAX | SCAMP1-AS1 | 0.320858898 | 4.69E-05 |
| CAPN10 | SCAMP1-AS1 | 0.424757715 | 3.62E-08 |
| CDKN1B | SCAMP1-AS1 | 0.31600739 | 6.19E-05 |
| GABARAP | SCAMP1-AS1 | 0.310737488 | 8.31E-05 |
| ITGA6 | SCAMP1-AS1 | -0.306161896 | 0.000106938 |
| MAP2K7 | SCAMP1-AS1 | 0.375824778 | 1.44E-06 |
| ULK3 | SCAMP1-AS1 | 0.397675401 | 2.99E-07 |
| WDR45 | SCAMP1-AS1 | 0.338378039 | 1.66E-05 |
| ATF4 | AC080013.6 | 0.339280102 | 1.57E-05 |
| ATG4B | AC080013.6 | 0.406608882 | 1.52E-07 |
| ATG4D | AC080013.6 | 0.301137542 | 0.000140348 |
| BAX | AC080013.6 | 0.385795853 | 7.14E-07 |
| BNIP1 | AC080013.6 | 0.337601376 | 1.74E-05 |
| CAPN10 | AC080013.6 | 0.338006313 | 1.70E-05 |
| DAPK2 | AC080013.6 | 0.374975833 | 1.53E-06 |
| GABARAP | AC080013.6 | 0.318235987 | 5.45E-05 |
| ITGA6 | AC080013.6 | -0.3088433 | 9.23E-05 |
| PELP1 | AC080013.6 | 0.365784566 | 2.86E-06 |
| ATF4 | AL049840.2 | -0.387237373 | 6.44E-07 |
| ATG2B | AL049840.2 | 0.61330906 | 2.19E-17 |
| ATG4B | AL049840.2 | -0.312692962 | 7.45E-05 |
| BAX | AL049840.2 | -0.415272384 | 7.75E-08 |
| BIRC6 | AL049840.2 | 0.314462033 | 6.75E-05 |
| BNIP1 | AL049840.2 | -0.424276421 | 3.76E-08 |
| CAPN2 | AL049840.2 | 0.313768189 | 7.02E-05 |
| CLN3 | AL049840.2 | -0.330453993 | 2.68E-05 |
| DDIT3 | AL049840.2 | -0.368472736 | 2.39E-06 |
| GABARAPL2 | AL049840.2 | -0.363316657 | 3.38E-06 |
| GOPC | AL049840.2 | 0.337205241 | 1.78E-05 |
| HSPA8 | AL049840.2 | 0.343538053 | 1.21E-05 |
| ITGA6 | AL049840.2 | 0.341057236 | 1.41E-05 |
| ITGB4 | AL049840.2 | 0.386230828 | 6.92E-07 |
| MAPK1 | AL049840.2 | 0.328097892 | 3.08E-05 |
| MTOR | AL049840.2 | 0.333188837 | 2.27E-05 |
| NFKB1 | AL049840.2 | 0.354422576 | 6.05E-06 |
| NRG1 | AL049840.2 | 0.342785917 | 1.26E-05 |
| PINK1 | AL049840.2 | 0.338637856 | 1.63E-05 |
| TM9SF1 | AL049840.2 | 0.304147923 | 0.000119321 |
| WDFY3 | AL049840.2 | 0.406430199 | 1.54E-07 |
| ZFYVE1 | AL049840.2 | 0.315546402 | 6.35E-05 |
| ATIC | PRRT3-AS1 | 0.496287468 | 5.14E-11 |
| BIRC5 | PRRT3-AS1 | 0.439264459 | 1.08E-08 |
| FOXO1 | PRRT3-AS1 | -0.305632249 | 0.000110073 |
| GABARAPL2 | PRRT3-AS1 | 0.374657614 | 1.56E-06 |
| MAPK8IP1 | PRRT3-AS1 | 0.682639102 | 1.34E-22 |
| MLST8 | PRRT3-AS1 | 0.308122922 | 9.60E-05 |
| PRKAR1A | PRRT3-AS1 | 0.387445613 | 6.34E-07 |
| ARSB | AL136084.3 | 0.405294218 | 1.68E-07 |
| ATG2B | AL136084.3 | 0.399801043 | 2.55E-07 |
| ATG4B | AL136084.3 | -0.342478808 | 1.29E-05 |
| BIRC6 | AL136084.3 | 0.376563542 | 1.37E-06 |
| BNIP1 | AL136084.3 | -0.332651463 | 2.35E-05 |
| CANX | AL136084.3 | 0.344231873 | 1.16E-05 |
| CAPN10 | AL136084.3 | -0.328150632 | 3.07E-05 |
| CCL2 | AL136084.3 | 0.362218299 | 3.63E-06 |
| CCR2 | AL136084.3 | 0.411686764 | 1.03E-07 |
| DAPK1 | AL136084.3 | 0.377738204 | 1.26E-06 |
| DLC1 | AL136084.3 | 0.629935116 | 1.62E-18 |
| ITPR1 | AL136084.3 | 0.32207383 | 4.37E-05 |
| KIF5B | AL136084.3 | 0.334148742 | 2.15E-05 |
| MAPK1 | AL136084.3 | 0.301292472 | 0.000139186 |
| MBTPS2 | AL136084.3 | 0.424492129 | 3.70E-08 |
| NRG1 | AL136084.3 | 0.347631903 | 9.34E-06 |
| PEX14 | AL136084.3 | -0.362912256 | 3.47E-06 |
| RAB24 | AL136084.3 | -0.362601795 | 3.54E-06 |
| WDFY3 | AL136084.3 | 0.37172868 | 1.91E-06 |
| ATF4 | AC073611.1 | 0.327261937 | 3.23E-05 |
| BIRC6 | AC073611.1 | -0.316154224 | 6.14E-05 |
| EEF2K | AC073611.1 | -0.303506645 | 0.000123537 |
| ATF4 | SPINT1-AS1 | 0.394997676 | 3.65E-07 |
| ATG2B | SPINT1-AS1 | -0.335834989 | 1.94E-05 |
| ATG3 | SPINT1-AS1 | 0.362094098 | 3.66E-06 |
| ATG4B | SPINT1-AS1 | 0.458059323 | 2.06E-09 |
| BAG1 | SPINT1-AS1 | 0.368199969 | 2.43E-06 |
| BAX | SPINT1-AS1 | 0.412811993 | 9.40E-08 |
| BID | SPINT1-AS1 | 0.416753901 | 6.89E-08 |
| BNIP1 | SPINT1-AS1 | 0.416687179 | 6.93E-08 |
| CANX | SPINT1-AS1 | -0.335440717 | 1.98E-05 |
| CAPN10 | SPINT1-AS1 | 0.419251496 | 5.65E-08 |
| CLN3 | SPINT1-AS1 | 0.417129169 | 6.69E-08 |
| DAPK2 | SPINT1-AS1 | 0.304958944 | 0.000114182 |
| DDIT3 | SPINT1-AS1 | 0.353608669 | 6.38E-06 |
| DLC1 | SPINT1-AS1 | -0.322376732 | 4.30E-05 |
| GABARAPL2 | SPINT1-AS1 | 0.385322494 | 7.39E-07 |
| GOPC | SPINT1-AS1 | -0.375829988 | 1.44E-06 |
| ITGA3 | SPINT1-AS1 | -0.427206329 | 2.96E-08 |
| ITGA6 | SPINT1-AS1 | -0.355648173 | 5.59E-06 |
| ITGB1 | SPINT1-AS1 | -0.333222567 | 2.27E-05 |
| ITGB4 | SPINT1-AS1 | -0.384623427 | 7.76E-07 |
| MAP1LC3A | SPINT1-AS1 | 0.353093536 | 6.59E-06 |
| MAPK1 | SPINT1-AS1 | -0.355136852 | 5.78E-06 |
| MTOR | SPINT1-AS1 | -0.3007624 | 0.000143198 |
| NRG1 | SPINT1-AS1 | -0.300832418 | 0.000142662 |
| PELP1 | SPINT1-AS1 | 0.315735436 | 6.28E-05 |
| PINK1 | SPINT1-AS1 | -0.324906306 | 3.71E-05 |
| RAB11A | SPINT1-AS1 | 0.322971687 | 4.15E-05 |
| RAB24 | SPINT1-AS1 | 0.311501618 | 7.97E-05 |
| TM9SF1 | SPINT1-AS1 | -0.354898478 | 5.87E-06 |
| ULK3 | SPINT1-AS1 | 0.389373542 | 5.51E-07 |
| USP10 | SPINT1-AS1 | -0.308338687 | 9.49E-05 |
| WDR45 | SPINT1-AS1 | 0.439702921 | 1.04E-08 |
| ZFYVE1 | SPINT1-AS1 | -0.306063044 | 0.000107517 |
| ARNT | LINC00592 | -0.303869497 | 0.000121135 |
| ARSB | LINC00592 | -0.342292469 | 1.30E-05 |
| ATG4C | LINC00592 | -0.35082457 | 7.62E-06 |
| CANX | LINC00592 | -0.32774298 | 3.14E-05 |
| DRAM1 | LINC00592 | -0.32925053 | 2.87E-05 |
| EDEM1 | LINC00592 | -0.312686378 | 7.46E-05 |
| MAPK9 | LINC00592 | -0.303963877 | 0.000120517 |
| MTOR | LINC00592 | -0.366528646 | 2.72E-06 |
| NBR1 | LINC00592 | -0.305715287 | 0.000109576 |
| PARP1 | LINC00592 | -0.339331623 | 1.57E-05 |
| TP53 | LINC00592 | -0.349213341 | 8.45E-06 |
| ATF4 | SNHG12 | 0.407296804 | 1.44E-07 |
| ATG16L2 | SNHG12 | 0.575313977 | 4.89E-15 |
| ATG4B | SNHG12 | 0.531738275 | 1.08E-12 |
| ATG4D | SNHG12 | 0.352799729 | 6.72E-06 |
| BAX | SNHG12 | 0.468350182 | 7.97E-10 |
| BIRC6 | SNHG12 | -0.349792136 | 8.14E-06 |
| BNIP1 | SNHG12 | 0.454136388 | 2.94E-09 |
| CAPN10 | SNHG12 | 0.68851027 | 4.17E-23 |
| CFLAR | SNHG12 | 0.315766722 | 6.27E-05 |
| CHMP4B | SNHG12 | 0.303947902 | 0.000120621 |
| CLN3 | SNHG12 | 0.352281433 | 6.95E-06 |
| CXCR4 | SNHG12 | 0.392360008 | 4.44E-07 |
| EIF4G1 | SNHG12 | -0.402686321 | 2.05E-07 |
| HIF1A | SNHG12 | -0.34797492 | 9.14E-06 |
| IKBKB | SNHG12 | 0.371851839 | 1.90E-06 |
| ITGA6 | SNHG12 | -0.30746956 | 9.96E-05 |
| ITGB1 | SNHG12 | -0.306660058 | 0.000104066 |
| KIF5B | SNHG12 | -0.37851058 | 1.20E-06 |
| LAMP2 | SNHG12 | -0.343191599 | 1.23E-05 |
| MAP1LC3A | SNHG12 | 0.308426325 | 9.45E-05 |
| MAP2K7 | SNHG12 | 0.35711327 | 5.08E-06 |
| MAPK1 | SNHG12 | -0.398758236 | 2.76E-07 |
| MBTPS2 | SNHG12 | -0.324538636 | 3.79E-05 |
| MLST8 | SNHG12 | 0.311920029 | 7.78E-05 |
| MTMR14 | SNHG12 | 0.323505794 | 4.03E-05 |
| NCKAP1 | SNHG12 | -0.390838033 | 4.96E-07 |
| NKX2-3 | SNHG12 | 0.301823355 | 0.000135274 |
| PEX14 | SNHG12 | 0.439627495 | 1.05E-08 |
| RAB24 | SNHG12 | 0.587989899 | 8.70E-16 |
| STK11 | SNHG12 | 0.502514985 | 2.69E-11 |
| TP63 | SNHG12 | -0.331939691 | 2.45E-05 |
| ULK3 | SNHG12 | 0.440516859 | 9.69E-09 |
| USP10 | SNHG12 | -0.332307129 | 2.40E-05 |
| WDFY3 | SNHG12 | -0.419352139 | 5.60E-08 |
| WDR45 | SNHG12 | 0.519609406 | 4.27E-12 |
| ZFYVE1 | SNHG12 | -0.30410697 | 0.000119586 |
| ARNT | AC073046.1 | 0.459652507 | 1.78E-09 |
| ATF4 | AC073046.1 | -0.331504532 | 2.51E-05 |
| ATF6 | AC073046.1 | 0.403359529 | 1.95E-07 |
| ATG12 | AC073046.1 | -0.306109872 | 0.000107242 |
| ATG2B | AC073046.1 | 0.361351761 | 3.85E-06 |
| ATG4B | AC073046.1 | -0.308934733 | 9.18E-05 |
| BAX | AC073046.1 | -0.373530637 | 1.69E-06 |
| BECN1 | AC073046.1 | 0.309894055 | 8.71E-05 |
| BIRC6 | AC073046.1 | 0.769031651 | 1.53E-31 |
| BNIP1 | AC073046.1 | -0.467381482 | 8.73E-10 |
| CAMKK2 | AC073046.1 | 0.373856034 | 1.65E-06 |
| DAPK1 | AC073046.1 | 0.362521977 | 3.56E-06 |
| EEF2 | AC073046.1 | 0.305048813 | 0.000113625 |
| EIF2AK3 | AC073046.1 | 0.509856206 | 1.24E-11 |
| EIF4G1 | AC073046.1 | 0.477002162 | 3.50E-10 |
| FOXO3 | AC073046.1 | 0.458788926 | 1.93E-09 |
| GABARAPL2 | AC073046.1 | -0.394971196 | 3.66E-07 |
| KIF5B | AC073046.1 | 0.492242797 | 7.76E-11 |
| KLHL24 | AC073046.1 | 0.349052663 | 8.53E-06 |
| MAPK1 | AC073046.1 | 0.446362248 | 5.84E-09 |
| MBTPS2 | AC073046.1 | 0.396654273 | 3.23E-07 |
| MTOR | AC073046.1 | 0.355694256 | 5.57E-06 |
| NAF1 | AC073046.1 | 0.31475015 | 6.64E-05 |
| NBR1 | AC073046.1 | 0.447991204 | 5.06E-09 |
| NCKAP1 | AC073046.1 | 0.529958948 | 1.33E-12 |
| NFE2L2 | AC073046.1 | 0.38273743 | 8.88E-07 |
| NPC1 | AC073046.1 | 0.36474453 | 3.07E-06 |
| PARP1 | AC073046.1 | 0.352324375 | 6.93E-06 |
| PEX14 | AC073046.1 | -0.453485287 | 3.11E-09 |
| PIK3R4 | AC073046.1 | 0.396169822 | 3.35E-07 |
| RAB24 | AC073046.1 | -0.400666222 | 2.39E-07 |
| RB1CC1 | AC073046.1 | 0.409233809 | 1.24E-07 |
| RPTOR | AC073046.1 | 0.369971238 | 2.16E-06 |
| SIRT1 | AC073046.1 | 0.317374256 | 5.73E-05 |
| SPNS1 | AC073046.1 | 0.464676797 | 1.12E-09 |
| TP63 | AC073046.1 | 0.414409029 | 8.29E-08 |
| USP10 | AC073046.1 | 0.362173797 | 3.64E-06 |
| UVRAG | AC073046.1 | 0.337576052 | 1.74E-05 |
| WDFY3 | AC073046.1 | 0.461962476 | 1.44E-09 |
| ATF4 | LINC01089 | 0.312327519 | 7.61E-05 |
| ATG16L2 | LINC01089 | 0.391852234 | 4.60E-07 |
| ATG3 | LINC01089 | 0.329326589 | 2.86E-05 |
| ATG4B | LINC01089 | 0.47020264 | 6.70E-10 |
| BAG3 | LINC01089 | -0.300420395 | 0.000145843 |
| BAX | LINC01089 | 0.50687911 | 1.70E-11 |
| BIRC5 | LINC01089 | 0.365302031 | 2.96E-06 |
| CAPN10 | LINC01089 | 0.576108749 | 4.40E-15 |
| CDKN1B | LINC01089 | 0.308040634 | 9.65E-05 |
| CXCR4 | LINC01089 | 0.55699677 | 5.21E-14 |
| DAPK2 | LINC01089 | 0.38351822 | 8.40E-07 |
| FKBP1A | LINC01089 | 0.340611795 | 1.45E-05 |
| HDAC6 | LINC01089 | 0.344606523 | 1.13E-05 |
| IKBKB | LINC01089 | 0.382954507 | 8.75E-07 |
| ITGA3 | LINC01089 | -0.343351846 | 1.22E-05 |
| MAP2K7 | LINC01089 | 0.386144609 | 6.96E-07 |
| MLST8 | LINC01089 | 0.309637096 | 8.84E-05 |
| MTMR14 | LINC01089 | 0.348448595 | 8.87E-06 |
| PELP1 | LINC01089 | 0.361685998 | 3.76E-06 |
| PINK1 | LINC01089 | -0.377537984 | 1.28E-06 |
| STK11 | LINC01089 | 0.348387627 | 8.90E-06 |
| TSC2 | LINC01089 | 0.315246158 | 6.46E-05 |
| ULK3 | LINC01089 | 0.409719815 | 1.20E-07 |
| WDR45 | LINC01089 | 0.445275449 | 6.42E-09 |
| ATF4 | AP006621.3 | 0.385815096 | 7.13E-07 |
| ATG16L2 | AP006621.3 | 0.330981168 | 2.59E-05 |
| ATG4B | AP006621.3 | 0.43454508 | 1.61E-08 |
| ATIC | AP006621.3 | 0.385837722 | 7.12E-07 |
| BIRC5 | AP006621.3 | 0.402354047 | 2.11E-07 |
| BNIP1 | AP006621.3 | 0.354319763 | 6.09E-06 |
| CANX | AP006621.3 | -0.303323067 | 0.000124769 |
| CAPN10 | AP006621.3 | 0.413681 | 8.78E-08 |
| CAPN2 | AP006621.3 | -0.363562392 | 3.32E-06 |
| CLN3 | AP006621.3 | 0.330643229 | 2.65E-05 |
| GABARAPL2 | AP006621.3 | 0.397282856 | 3.08E-07 |
| LAMP2 | AP006621.3 | -0.352033257 | 7.06E-06 |
| MAPK1 | AP006621.3 | -0.363789997 | 3.27E-06 |
| MAPK8IP1 | AP006621.3 | 0.323238992 | 4.09E-05 |
| MLST8 | AP006621.3 | 0.388217851 | 6.00E-07 |
| NFKB1 | AP006621.3 | -0.337382339 | 1.76E-05 |
| PELP1 | AP006621.3 | 0.304479773 | 0.000117193 |
| RB1 | AP006621.3 | -0.301172485 | 0.000140085 |
| STK11 | AP006621.3 | 0.337867921 | 1.71E-05 |
| WDR45 | AP006621.3 | 0.366765473 | 2.68E-06 |
| CAPN10 | LENG8-AS1 | 0.347990274 | 9.13E-06 |
| CDKN1B | LENG8-AS1 | 0.322917816 | 4.17E-05 |
| CXCR4 | LENG8-AS1 | 0.422630901 | 4.30E-08 |
| HDAC6 | LENG8-AS1 | 0.408399401 | 1.33E-07 |
| IKBKB | LENG8-AS1 | 0.384752181 | 7.69E-07 |
| MAP2K7 | LENG8-AS1 | 0.326983458 | 3.29E-05 |
| RAF1 | LENG8-AS1 | 0.339536333 | 1.55E-05 |
| TSC1 | LENG8-AS1 | 0.372228233 | 1.85E-06 |
| UVRAG | LENG8-AS1 | 0.335162119 | 2.02E-05 |
| ARSB | AC010789.1 | -0.310871577 | 8.25E-05 |
| ATG16L2 | AC010789.1 | 0.334252996 | 2.13E-05 |
| ATG9B | AC010789.1 | 0.310327023 | 8.50E-05 |
| BID | AC010789.1 | 0.308712916 | 9.30E-05 |
| FKBP1B | AC010789.1 | 0.511763381 | 1.01E-11 |
| FOXO1 | AC010789.1 | -0.375610355 | 1.46E-06 |
| MYC | AC010789.1 | 0.344784836 | 1.12E-05 |
| ARSB | AL035587.1 | 0.382210292 | 9.22E-07 |
| ATG4C | AL035587.1 | 0.339252386 | 1.57E-05 |
| BCL2 | AL035587.1 | 0.497877421 | 4.36E-11 |
| BIRC6 | AL035587.1 | 0.356981466 | 5.13E-06 |
| BNIP3 | AL035587.1 | -0.321571136 | 4.50E-05 |
| CAMKK2 | AL035587.1 | 0.308873945 | 9.22E-05 |
| CCR2 | AL035587.1 | 0.352621184 | 6.80E-06 |
| CDKN1B | AL035587.1 | 0.437247839 | 1.28E-08 |
| CX3CL1 | AL035587.1 | 0.331529269 | 2.51E-05 |
| CXCR4 | AL035587.1 | 0.539001628 | 4.65E-13 |
| DRAM1 | AL035587.1 | 0.315611797 | 6.33E-05 |
| EEF2K | AL035587.1 | 0.443531198 | 7.47E-09 |
| EIF2AK3 | AL035587.1 | 0.319867926 | 4.97E-05 |
| FAS | AL035587.1 | 0.35875431 | 4.57E-06 |
| HDAC6 | AL035587.1 | 0.405813596 | 1.62E-07 |
| IKBKB | AL035587.1 | 0.392309555 | 4.45E-07 |
| IKBKE | AL035587.1 | 0.385747178 | 7.16E-07 |
| KLHL24 | AL035587.1 | 0.407917154 | 1.38E-07 |
| MAP2K7 | AL035587.1 | 0.434963667 | 1.55E-08 |
| MAPK8 | AL035587.1 | 0.371185622 | 1.99E-06 |
| MAPK9 | AL035587.1 | 0.3163175 | 6.08E-05 |
| NBR1 | AL035587.1 | 0.368245895 | 2.43E-06 |
| NKX2-3 | AL035587.1 | 0.380475385 | 1.04E-06 |
| PARP1 | AL035587.1 | 0.390388036 | 5.12E-07 |
| PIK3C3 | AL035587.1 | 0.335876549 | 1.93E-05 |
| PIK3R4 | AL035587.1 | 0.394566038 | 3.77E-07 |
| PINK1 | AL035587.1 | -0.32846911 | 3.01E-05 |
| PRKAB1 | AL035587.1 | 0.439413804 | 1.06E-08 |
| PTK6 | AL035587.1 | -0.380519841 | 1.04E-06 |
| RAF1 | AL035587.1 | 0.3575101 | 4.95E-06 |
| SIRT1 | AL035587.1 | 0.303030815 | 0.000126755 |
| STK11 | AL035587.1 | 0.307509417 | 9.93E-05 |
| TP53 | AL035587.1 | 0.531351279 | 1.13E-12 |
| TP73 | AL035587.1 | 0.388896085 | 5.71E-07 |
| TSC1 | AL035587.1 | 0.426725915 | 3.08E-08 |
| TSC2 | AL035587.1 | 0.464891265 | 1.10E-09 |
| ULK2 | AL035587.1 | 0.372688757 | 1.79E-06 |
| UVRAG | AL035587.1 | 0.337697232 | 1.73E-05 |
| VAMP7 | AL035587.1 | 0.300204039 | 0.000147539 |
| ATG3 | MBNL1-AS1 | 0.34110195 | 1.40E-05 |
| EEF2K | MBNL1-AS1 | 0.305705299 | 0.000109635 |
| PIK3R4 | MBNL1-AS1 | 0.456774516 | 2.32E-09 |
| GABARAP | BX640514.2 | -0.335207672 | 2.01E-05 |
| PTK6 | BX640514.2 | 0.367630541 | 2.53E-06 |
| ATF4 | AC010719.1 | 0.343354892 | 1.22E-05 |
| ATG16L2 | AC010719.1 | 0.305430656 | 0.000111288 |
| ATG4B | AC010719.1 | 0.315452721 | 6.38E-05 |
| ATG4C | AC010719.1 | 0.305865081 | 0.000108684 |
| ATIC | AC010719.1 | 0.451938964 | 3.57E-09 |
| BAG3 | AC010719.1 | -0.384897276 | 7.61E-07 |
| BAX | AC010719.1 | 0.405558552 | 1.65E-07 |
| BIRC5 | AC010719.1 | 0.497428521 | 4.57E-11 |
| BNIP1 | AC010719.1 | 0.390845886 | 4.95E-07 |
| CAPN10 | AC010719.1 | 0.449303269 | 4.51E-09 |
| CAPN2 | AC010719.1 | -0.3175774 | 5.66E-05 |
| CXCR4 | AC010719.1 | 0.378411053 | 1.21E-06 |
| EIF4G1 | AC010719.1 | -0.338202005 | 1.68E-05 |
| FKBP1A | AC010719.1 | 0.461513157 | 1.50E-09 |
| HDAC6 | AC010719.1 | 0.31347704 | 7.13E-05 |
| MAPK1 | AC010719.1 | -0.308980535 | 9.16E-05 |
| MAPK8IP1 | AC010719.1 | 0.573298378 | 6.39E-15 |
| MLST8 | AC010719.1 | 0.320521644 | 4.78E-05 |
| MTMR14 | AC010719.1 | 0.353084811 | 6.60E-06 |
| NCKAP1 | AC010719.1 | -0.321018118 | 4.65E-05 |
| RAB24 | AC010719.1 | 0.348859523 | 8.64E-06 |
| ULK3 | AC010719.1 | 0.316815536 | 5.91E-05 |
| WDFY3 | AC010719.1 | -0.30942765 | 8.94E-05 |
| WDR45 | AC010719.1 | 0.508459632 | 1.44E-11 |
| CXCR4 | AC004585.1 | 0.382579059 | 8.98E-07 |
| RGS19 | AC004585.1 | 0.415076066 | 7.87E-08 |
| ATF4 | ARHGAP27P1-BPTFP1-KPNA2P3 | 0.302640282 | 0.000129454 |
| ATG16L2 | ARHGAP27P1-BPTFP1-KPNA2P3 | 0.543738047 | 2.65E-13 |
| ATG4B | ARHGAP27P1-BPTFP1-KPNA2P3 | 0.35330687 | 6.50E-06 |
| ATG9B | ARHGAP27P1-BPTFP1-KPNA2P3 | 0.538411864 | 4.99E-13 |
| CAPN10 | ARHGAP27P1-BPTFP1-KPNA2P3 | 0.434945938 | 1.56E-08 |
| DAPK2 | ARHGAP27P1-BPTFP1-KPNA2P3 | 0.314109231 | 6.89E-05 |
| IKBKB | ARHGAP27P1-BPTFP1-KPNA2P3 | 0.317316641 | 5.74E-05 |
| MAPK8IP1 | ARHGAP27P1-BPTFP1-KPNA2P3 | 0.301155673 | 0.000140211 |
| RAB24 | ARHGAP27P1-BPTFP1-KPNA2P3 | 0.356352229 | 5.34E-06 |
| ULK3 | ARHGAP27P1-BPTFP1-KPNA2P3 | 0.43043295 | 2.27E-08 |
| WDR45 | ARHGAP27P1-BPTFP1-KPNA2P3 | 0.338400346 | 1.66E-05 |
| WIPI1 | ARHGAP27P1-BPTFP1-KPNA2P3 | 0.381819428 | 9.48E-07 |
| AMBRA1 | AC090559.1 | 0.417288449 | 6.60E-08 |
| ARNT | AC090559.1 | 0.3967433 | 3.21E-07 |
| ARSB | AC090559.1 | 0.689650437 | 3.31E-23 |
| ATF6 | AC090559.1 | 0.326638167 | 3.35E-05 |
| ATG2B | AC090559.1 | 0.387311752 | 6.40E-07 |
| ATG4C | AC090559.1 | 0.336681661 | 1.84E-05 |
| BCL2 | AC090559.1 | 0.376930192 | 1.34E-06 |
| BIRC6 | AC090559.1 | 0.452754188 | 3.32E-09 |
| BNIP3 | AC090559.1 | -0.30000034 | 0.000149154 |
| CANX | AC090559.1 | 0.324847571 | 3.72E-05 |
| CCL2 | AC090559.1 | 0.372990851 | 1.76E-06 |
| CCR2 | AC090559.1 | 0.767864444 | 2.14E-31 |
| CXCR4 | AC090559.1 | 0.36675447 | 2.68E-06 |
| DAPK1 | AC090559.1 | 0.436904777 | 1.32E-08 |
| DLC1 | AC090559.1 | 0.476952238 | 3.52E-10 |
| DRAM1 | AC090559.1 | 0.475792675 | 3.93E-10 |
| EDEM1 | AC090559.1 | 0.405697137 | 1.63E-07 |
| EEF2K | AC090559.1 | 0.336778122 | 1.83E-05 |
| ERN1 | AC090559.1 | 0.335621478 | 1.96E-05 |
| FAS | AC090559.1 | 0.371248098 | 1.98E-06 |
| FKBP1B | AC090559.1 | -0.310240296 | 8.54E-05 |
| FOXO1 | AC090559.1 | 0.351636456 | 7.24E-06 |
| IFNG | AC090559.1 | 0.366634529 | 2.70E-06 |
| IKBKE | AC090559.1 | 0.300631532 | 0.000144204 |
| ITPR1 | AC090559.1 | 0.652279983 | 3.79E-20 |
| MTOR | AC090559.1 | 0.4191545 | 5.69E-08 |
| NBR1 | AC090559.1 | 0.375071056 | 1.52E-06 |
| PARP1 | AC090559.1 | 0.322701557 | 4.22E-05 |
| PEA15 | AC090559.1 | 0.380301899 | 1.06E-06 |
| PRKCD | AC090559.1 | 0.362512389 | 3.56E-06 |
| PTK6 | AC090559.1 | -0.333927425 | 2.17E-05 |
| RGS19 | AC090559.1 | 0.434897026 | 1.56E-08 |
| SERPINA1 | AC090559.1 | 0.438877406 | 1.11E-08 |
| SH3GLB1 | AC090559.1 | 0.337526231 | 1.75E-05 |
| SIRT1 | AC090559.1 | 0.366371011 | 2.75E-06 |
| ULK2 | AC090559.1 | 0.413385469 | 8.99E-08 |
| VAMP7 | AC090559.1 | 0.318089778 | 5.50E-05 |
| AMBRA1 | AC125807.2 | 0.369075218 | 2.29E-06 |
| ARNT | AC125807.2 | 0.322260467 | 4.33E-05 |
| ARSB | AC125807.2 | 0.320344769 | 4.83E-05 |
| BIRC6 | AC125807.2 | 0.39575163 | 3.45E-07 |
| CAMKK2 | AC125807.2 | 0.46052636 | 1.65E-09 |
| CANX | AC125807.2 | 0.303131354 | 0.000126068 |
| CCL2 | AC125807.2 | 0.304019448 | 0.000120155 |
| CDKN1B | AC125807.2 | 0.450713296 | 3.98E-09 |
| DRAM1 | AC125807.2 | 0.336187086 | 1.90E-05 |
| EEF2K | AC125807.2 | 0.392974421 | 4.24E-07 |
| GABARAPL2 | AC125807.2 | -0.305888364 | 0.000108546 |
| MAPK1 | AC125807.2 | 0.320409273 | 4.81E-05 |
| MAPK8 | AC125807.2 | 0.404504929 | 1.79E-07 |
| MTOR | AC125807.2 | 0.4678847 | 8.33E-10 |
| NAF1 | AC125807.2 | 0.321519315 | 4.52E-05 |
| NFKB1 | AC125807.2 | 0.413755482 | 8.73E-08 |
| NKX2-3 | AC125807.2 | 0.319738761 | 5.00E-05 |
| PARP1 | AC125807.2 | 0.390745638 | 4.99E-07 |
| PIK3R4 | AC125807.2 | 0.343065808 | 1.24E-05 |
| PTEN | AC125807.2 | 0.311239886 | 8.08E-05 |
| RAB24 | AC125807.2 | -0.302237646 | 0.000132293 |
| RB1 | AC125807.2 | 0.373878118 | 1.65E-06 |
| SPNS1 | AC125807.2 | 0.334097322 | 2.15E-05 |
| TP73 | AC125807.2 | 0.310455354 | 8.44E-05 |
| AMBRA1 | AC015922.2 | 0.351454456 | 7.32E-06 |
| ARNT | AC015922.2 | 0.406024352 | 1.59E-07 |
| ARSB | AC015922.2 | 0.423744044 | 3.93E-08 |
| ATF6 | AC015922.2 | 0.434212536 | 1.65E-08 |
| BIRC6 | AC015922.2 | 0.317515137 | 5.68E-05 |
| CANX | AC015922.2 | 0.370126685 | 2.14E-06 |
| DAPK1 | AC015922.2 | 0.460307843 | 1.68E-09 |
| DLC1 | AC015922.2 | 0.313295066 | 7.21E-05 |
| EDEM1 | AC015922.2 | 0.317358179 | 5.73E-05 |
| EIF2AK2 | AC015922.2 | 0.311312028 | 8.05E-05 |
| FOXO1 | AC015922.2 | 0.43594112 | 1.43E-08 |
| FOXO3 | AC015922.2 | 0.300295534 | 0.00014682 |
| ITPR1 | AC015922.2 | 0.383317104 | 8.52E-07 |
| KIF5B | AC015922.2 | 0.397963321 | 2.93E-07 |
| KLHL24 | AC015922.2 | 0.349227686 | 8.44E-06 |
| MBTPS2 | AC015922.2 | 0.323367744 | 4.06E-05 |
| NBR1 | AC015922.2 | 0.450110177 | 4.20E-09 |
| PARP1 | AC015922.2 | 0.34180627 | 1.34E-05 |
| PEA15 | AC015922.2 | 0.349201294 | 8.45E-06 |
| PIK3C3 | AC015922.2 | 0.324669775 | 3.76E-05 |
| PRKAR1A | AC015922.2 | 0.351531126 | 7.29E-06 |
| SIRT1 | AC015922.2 | 0.399914417 | 2.53E-07 |
| SQSTM1 | AC015922.2 | 0.408246661 | 1.34E-07 |
| ST13 | AC015922.2 | 0.349503402 | 8.29E-06 |
| ULK2 | AC015922.2 | 0.496182598 | 5.19E-11 |
| USP10 | AC015922.2 | 0.348888553 | 8.62E-06 |
| UVRAG | AC015922.2 | 0.369336808 | 2.25E-06 |
| VAMP7 | AC015922.2 | 0.30420472 | 0.000118954 |
| BIRC6 | AL121603.2 | 0.302538461 | 0.000130166 |
| CDKN1B | AL121603.2 | 0.361845399 | 3.72E-06 |
| EIF2AK3 | AL121603.2 | 0.375544414 | 1.47E-06 |
| PRKAB1 | AL121603.2 | 0.448802058 | 4.72E-09 |
| ARNT | AL391121.1 | 0.370156389 | 2.13E-06 |
| ARSB | AL391121.1 | 0.34107803 | 1.41E-05 |
| ATG2A | AL391121.1 | 0.429748174 | 2.40E-08 |
| ATG7 | AL391121.1 | 0.36298367 | 3.45E-06 |
| BCL2 | AL391121.1 | 0.490951289 | 8.84E-11 |
| BECN1 | AL391121.1 | 0.350010195 | 8.03E-06 |
| BIRC6 | AL391121.1 | 0.44240375 | 8.24E-09 |
| CANX | AL391121.1 | 0.332905264 | 2.31E-05 |
| CCR2 | AL391121.1 | 0.308895077 | 9.20E-05 |
| CD46 | AL391121.1 | 0.315383548 | 6.41E-05 |
| CDKN1B | AL391121.1 | 0.486624257 | 1.36E-10 |
| CFLAR | AL391121.1 | 0.331792462 | 2.47E-05 |
| DRAM1 | AL391121.1 | 0.395149677 | 3.61E-07 |
| EEF2K | AL391121.1 | 0.552694363 | 8.90E-14 |
| EIF2AK3 | AL391121.1 | 0.382459773 | 9.06E-07 |
| FAS | AL391121.1 | 0.48593366 | 1.46E-10 |
| FOXO3 | AL391121.1 | 0.320967926 | 4.66E-05 |
| HDAC6 | AL391121.1 | 0.383606397 | 8.35E-07 |
| IKBKB | AL391121.1 | 0.365302089 | 2.96E-06 |
| IKBKE | AL391121.1 | 0.459689835 | 1.78E-09 |
| ITPR1 | AL391121.1 | 0.346909685 | 9.77E-06 |
| MAPK8 | AL391121.1 | 0.608347575 | 4.62E-17 |
| MAPK9 | AL391121.1 | 0.398846278 | 2.74E-07 |
| MTOR | AL391121.1 | 0.502891 | 2.59E-11 |
| NBR1 | AL391121.1 | 0.544155554 | 2.52E-13 |
| NFKB1 | AL391121.1 | 0.426234564 | 3.21E-08 |
| NKX2-3 | AL391121.1 | 0.55115387 | 1.08E-13 |
| PARP1 | AL391121.1 | 0.501330899 | 3.05E-11 |
| PIK3R4 | AL391121.1 | 0.495695759 | 5.46E-11 |
| PRKAB1 | AL391121.1 | 0.418275528 | 6.10E-08 |
| PTEN | AL391121.1 | 0.325985836 | 3.48E-05 |
| RAB33B | AL391121.1 | 0.382360185 | 9.12E-07 |
| RAF1 | AL391121.1 | 0.432416012 | 1.92E-08 |
| RB1CC1 | AL391121.1 | 0.338809629 | 1.62E-05 |
| SH3GLB1 | AL391121.1 | 0.330113393 | 2.73E-05 |
| SIRT1 | AL391121.1 | 0.470506025 | 6.51E-10 |
| SPNS1 | AL391121.1 | 0.323566207 | 4.01E-05 |
| TP53 | AL391121.1 | 0.499940231 | 3.52E-11 |
| TP73 | AL391121.1 | 0.554038789 | 7.54E-14 |
| TSC2 | AL391121.1 | 0.558076282 | 4.55E-14 |
| ULK2 | AL391121.1 | 0.31809497 | 5.50E-05 |
| UVRAG | AL391121.1 | 0.322983239 | 4.15E-05 |
| WDFY3 | AL391121.1 | 0.339968736 | 1.51E-05 |
| ATG10 | AC099518.6 | 0.340526566 | 1.45E-05 |
| ATG3 | AC099518.6 | 0.325465497 | 3.59E-05 |
| ATG4A | AC099518.6 | 0.349859919 | 8.11E-06 |
| ATG4D | AC099518.6 | 0.355067935 | 5.80E-06 |
| BAX | AC099518.6 | 0.319334204 | 5.12E-05 |
| BNIP1 | AC099518.6 | 0.379111161 | 1.15E-06 |
| CAPNS1 | AC099518.6 | 0.316166344 | 6.13E-05 |
| CDKN2A | AC099518.6 | 0.327891378 | 3.11E-05 |
| CFLAR | AC099518.6 | 0.304171097 | 0.000119171 |
| CLN3 | AC099518.6 | 0.457541905 | 2.16E-09 |
| GABARAP | AC099518.6 | 0.328388226 | 3.02E-05 |
| HDAC6 | AC099518.6 | 0.483578749 | 1.84E-10 |
| HSPA8 | AC099518.6 | -0.304027301 | 0.000120104 |
| IKBKB | AC099518.6 | 0.360063082 | 4.19E-06 |
| IKBKE | AC099518.6 | 0.394495281 | 3.79E-07 |
| ITGA6 | AC099518.6 | -0.328456402 | 3.01E-05 |
| MAPK9 | AC099518.6 | 0.348316539 | 8.94E-06 |
| MTMR14 | AC099518.6 | 0.322189434 | 4.35E-05 |
| NKX2-3 | AC099518.6 | 0.365411229 | 2.94E-06 |
| PEX14 | AC099518.6 | 0.40996555 | 1.17E-07 |
| WDR45 | AC099518.6 | 0.460096231 | 1.71E-09 |
| BID | AC016642.1 | 0.345035565 | 1.10E-05 |
| DAPK2 | AC016642.1 | 0.366064079 | 2.81E-06 |
| DIRAS3 | AC016642.1 | 0.430523624 | 2.25E-08 |
| FKBP1B | AC016642.1 | 0.324097634 | 3.89E-05 |
| PELP1 | AC016642.1 | 0.406370625 | 1.55E-07 |
| ARSB | AC011477.3 | 0.338287923 | 1.67E-05 |
| BCL2 | AC011477.3 | 0.408563938 | 1.31E-07 |
| BIRC6 | AC011477.3 | 0.317495099 | 5.69E-05 |
| CCR2 | AC011477.3 | 0.370652289 | 2.06E-06 |
| CDKN1B | AC011477.3 | 0.360717963 | 4.01E-06 |
| CXCR4 | AC011477.3 | 0.375850681 | 1.44E-06 |
| DAPK1 | AC011477.3 | 0.37604214 | 1.42E-06 |
| EEF2K | AC011477.3 | 0.314338305 | 6.80E-05 |
| EIF2AK3 | AC011477.3 | 0.309621502 | 8.84E-05 |
| HDAC6 | AC011477.3 | 0.355453354 | 5.66E-06 |
| IKBKB | AC011477.3 | 0.331235023 | 2.55E-05 |
| IKBKE | AC011477.3 | 0.308327738 | 9.50E-05 |
| ITGA3 | AC011477.3 | -0.319662752 | 5.03E-05 |
| ITPR1 | AC011477.3 | 0.306496268 | 0.000105002 |
| KLHL24 | AC011477.3 | 0.340449255 | 1.46E-05 |
| MAP2K7 | AC011477.3 | 0.424426473 | 3.72E-08 |
| MTMR14 | AC011477.3 | 0.351108102 | 7.49E-06 |
| PARP1 | AC011477.3 | 0.308807157 | 9.25E-05 |
| PIK3C3 | AC011477.3 | 0.313507208 | 7.12E-05 |
| PRKAB1 | AC011477.3 | 0.331366785 | 2.53E-05 |
| RAF1 | AC011477.3 | 0.391336268 | 4.78E-07 |
| TP53 | AC011477.3 | 0.309454017 | 8.92E-05 |
| TSC1 | AC011477.3 | 0.332210886 | 2.41E-05 |
| TSC2 | AC011477.3 | 0.389517298 | 5.46E-07 |
| ULK2 | AC011477.3 | 0.356821142 | 5.18E-06 |
| UVRAG | AC011477.3 | 0.464379007 | 1.15E-09 |
| BAK1 | AL391244.2 | -0.370779101 | 2.04E-06 |
| BID | AL391244.2 | 0.312349966 | 7.60E-05 |
| ITGA3 | AL391244.2 | -0.404388041 | 1.80E-07 |
| PARP1 | AL391244.2 | 0.33949021 | 1.55E-05 |
| PINK1 | AL391244.2 | -0.301349986 | 0.000138757 |
| PRKAB1 | AL391244.2 | 0.383738513 | 8.27E-07 |
| RAF1 | AL391244.2 | 0.325014889 | 3.69E-05 |
| ULK3 | AL391244.2 | 0.356091795 | 5.43E-06 |
| ATF4 | PLBD1-AS1 | -0.309452356 | 8.93E-05 |
| ATG2B | PLBD1-AS1 | 0.317338046 | 5.74E-05 |
| ATG9B | PLBD1-AS1 | 0.33456298 | 2.09E-05 |
| BIRC6 | PLBD1-AS1 | 0.438688649 | 1.13E-08 |
| BNIP1 | PLBD1-AS1 | -0.313361857 | 7.18E-05 |
| EIF2AK3 | PLBD1-AS1 | 0.349229153 | 8.44E-06 |
| EIF4G1 | PLBD1-AS1 | 0.312464523 | 7.55E-05 |
| MAPK1 | PLBD1-AS1 | 0.327465337 | 3.19E-05 |
| MTOR | PLBD1-AS1 | 0.34634195 | 1.01E-05 |
| NAF1 | PLBD1-AS1 | 0.302130883 | 0.000133055 |
| NCKAP1 | PLBD1-AS1 | 0.370772147 | 2.04E-06 |
| RAB11A | PLBD1-AS1 | 0.371133967 | 1.99E-06 |
| TP53INP2 | PLBD1-AS1 | 0.467785488 | 8.41E-10 |
| WDFY3 | PLBD1-AS1 | 0.390089266 | 5.24E-07 |
| ARNT | USP46-AS1 | 0.347494834 | 9.42E-06 |
| ATG2B | USP46-AS1 | 0.321225757 | 4.59E-05 |
| BECN1 | USP46-AS1 | 0.368521675 | 2.38E-06 |
| BIRC6 | USP46-AS1 | 0.416086724 | 7.26E-08 |
| BNIP1 | USP46-AS1 | -0.318949382 | 5.23E-05 |
| CD46 | USP46-AS1 | 0.353334385 | 6.49E-06 |
| EIF2AK3 | USP46-AS1 | 0.442377505 | 8.26E-09 |
| ERN1 | USP46-AS1 | 0.306080982 | 0.000107411 |
| GABARAPL1 | USP46-AS1 | 0.307016584 | 0.000102055 |
| MBTPS2 | USP46-AS1 | 0.323074519 | 4.13E-05 |
| NAF1 | USP46-AS1 | 0.346969343 | 9.74E-06 |
| NBR1 | USP46-AS1 | 0.351404587 | 7.35E-06 |
| NCKAP1 | USP46-AS1 | 0.359558647 | 4.33E-06 |
| NFE2L2 | USP46-AS1 | 0.403836326 | 1.88E-07 |
| RAB33B | USP46-AS1 | 0.335345897 | 2.00E-05 |
| RAB5A | USP46-AS1 | 0.331110163 | 2.57E-05 |
| TP53INP2 | USP46-AS1 | 0.363448235 | 3.35E-06 |
| WDFY3 | USP46-AS1 | 0.421255693 | 4.81E-08 |
| AMBRA1 | AP001453.2 | -0.324154639 | 3.88E-05 |
| ARNT | AP001453.2 | -0.415014766 | 7.91E-08 |
| ARSB | AP001453.2 | -0.522638645 | 3.05E-12 |
| ATF6 | AP001453.2 | -0.330902985 | 2.61E-05 |
| BCL2 | AP001453.2 | -0.375289579 | 1.50E-06 |
| BIRC6 | AP001453.2 | -0.432742302 | 1.87E-08 |
| CAMKK2 | AP001453.2 | -0.351698261 | 7.21E-06 |
| CANX | AP001453.2 | -0.387989507 | 6.10E-07 |
| CASP4 | AP001453.2 | 0.338486442 | 1.65E-05 |
| CD46 | AP001453.2 | -0.428816722 | 2.59E-08 |
| CDKN1B | AP001453.2 | -0.412063237 | 9.97E-08 |
| CXCR4 | AP001453.2 | -0.355070191 | 5.80E-06 |
| DRAM1 | AP001453.2 | -0.396835239 | 3.19E-07 |
| EEF2 | AP001453.2 | -0.325593184 | 3.56E-05 |
| EEF2K | AP001453.2 | -0.380020524 | 1.08E-06 |
| EIF2AK2 | AP001453.2 | -0.310544777 | 8.40E-05 |
| EIF2AK3 | AP001453.2 | -0.395598387 | 3.49E-07 |
| FAS | AP001453.2 | -0.354526199 | 6.01E-06 |
| FOXO1 | AP001453.2 | -0.380515989 | 1.04E-06 |
| GAA | AP001453.2 | -0.318629291 | 5.33E-05 |
| GABARAP | AP001453.2 | -0.321257848 | 4.59E-05 |
| HDAC6 | AP001453.2 | -0.442423342 | 8.22E-09 |
| IKBKB | AP001453.2 | -0.325718117 | 3.54E-05 |
| ITPR1 | AP001453.2 | -0.372253865 | 1.85E-06 |
| KLHL24 | AP001453.2 | -0.412050379 | 9.98E-08 |
| MAP2K7 | AP001453.2 | -0.32339787 | 4.05E-05 |
| MAPK8 | AP001453.2 | -0.371398565 | 1.96E-06 |
| MAPK9 | AP001453.2 | -0.49025297 | 9.49E-11 |
| MBTPS2 | AP001453.2 | -0.321384538 | 4.55E-05 |
| MTMR14 | AP001453.2 | -0.306461334 | 0.000105203 |
| MTOR | AP001453.2 | -0.346301707 | 1.02E-05 |
| NBR1 | AP001453.2 | -0.474658926 | 4.39E-10 |
| PARP1 | AP001453.2 | -0.504384738 | 2.21E-11 |
| PIK3R4 | AP001453.2 | -0.371878648 | 1.89E-06 |
| PRKAB1 | AP001453.2 | -0.312535421 | 7.52E-05 |
| PRKAR1A | AP001453.2 | -0.304586058 | 0.000116518 |
| PTK6 | AP001453.2 | 0.556868272 | 5.30E-14 |
| RAB33B | AP001453.2 | -0.368182225 | 2.44E-06 |
| RAF1 | AP001453.2 | -0.306152134 | 0.000106995 |
| RB1CC1 | AP001453.2 | -0.384281706 | 7.96E-07 |
| SIRT1 | AP001453.2 | -0.402840283 | 2.03E-07 |
| SQSTM1 | AP001453.2 | -0.399604555 | 2.59E-07 |
| TBK1 | AP001453.2 | -0.311885847 | 7.80E-05 |
| TP53 | AP001453.2 | -0.415302045 | 7.73E-08 |
| TP73 | AP001453.2 | -0.302827857 | 0.000128151 |
| TSC2 | AP001453.2 | -0.327347104 | 3.22E-05 |
| ULK2 | AP001453.2 | -0.361532291 | 3.80E-06 |
| UVRAG | AP001453.2 | -0.37187361 | 1.90E-06 |
| VAMP7 | AP001453.2 | -0.345658784 | 1.06E-05 |
| ATF4 | AC020978.4 | -0.330662874 | 2.64E-05 |
| ATG9B | AC020978.4 | 0.430654876 | 2.23E-08 |
| CAPN1 | AC020978.4 | 0.343898296 | 1.18E-05 |
| CAPN2 | AC020978.4 | 0.305532217 | 0.000110674 |
| EIF2AK3 | AC020978.4 | 0.352282292 | 6.95E-06 |
| PTK6 | AC020978.4 | 0.304807835 | 0.000115123 |
| RAB11A | AC020978.4 | 0.408434555 | 1.32E-07 |
| TP53INP2 | AC020978.4 | 0.580750958 | 2.35E-15 |
| WDFY3 | AC020978.4 | 0.309906177 | 8.70E-05 |
| ITGA3 | ZNF503-AS2 | -0.308815839 | 9.24E-05 |
| ULK3 | ZNF503-AS2 | 0.315784349 | 6.27E-05 |
| ATG3 | AC092368.3 | 0.326458361 | 3.39E-05 |
| BECN1 | AC092368.3 | 0.419438366 | 5.56E-08 |
| EDEM1 | AC092368.3 | 0.380130327 | 1.07E-06 |
| FOXO1 | AC092368.3 | 0.318429659 | 5.39E-05 |
| NBR1 | AC092368.3 | 0.347293091 | 9.54E-06 |
| NCKAP1 | AC092368.3 | 0.372024186 | 1.88E-06 |
| PIK3R4 | AC092368.3 | 0.500656679 | 3.27E-11 |
| RAB5A | AC092368.3 | 0.363066639 | 3.43E-06 |
| RAB7A | AC092368.3 | 0.499647485 | 3.63E-11 |
| TM9SF1 | AC092368.3 | 0.348476593 | 8.85E-06 |
| SPHK1 | AC011374.1 | 0.312684839 | 7.46E-05 |
| AMBRA1 | AP003392.1 | 0.313475438 | 7.14E-05 |
| BIRC6 | AP003392.1 | 0.424702671 | 3.64E-08 |
| CASP8 | AP003392.1 | 0.313340096 | 7.19E-05 |
| EDEM1 | AP003392.1 | 0.332115149 | 2.42E-05 |
| EEF2K | AP003392.1 | 0.316562912 | 6.00E-05 |
| EGFR | AP003392.1 | 0.421195685 | 4.83E-08 |
| EIF2AK3 | AP003392.1 | 0.330153695 | 2.72E-05 |
| ERN1 | AP003392.1 | 0.347884045 | 9.19E-06 |
| GOPC | AP003392.1 | 0.331523204 | 2.51E-05 |
| MTOR | AP003392.1 | 0.303784196 | 0.000121696 |
| SPNS1 | AP003392.1 | 0.45212495 | 3.51E-09 |
| TSC1 | AP003392.1 | 0.347741768 | 9.27E-06 |
| UVRAG | AP003392.1 | 0.31980697 | 4.98E-05 |
| ATG16L2 | TMEM147-AS1 | 0.412081795 | 9.96E-08 |
| CAPN10 | TMEM147-AS1 | 0.354453167 | 6.04E-06 |
| CASP8 | TMEM147-AS1 | 0.381597519 | 9.63E-07 |
| CDKN1A | TMEM147-AS1 | -0.301981726 | 0.000134127 |
| CFLAR | TMEM147-AS1 | 0.386935721 | 6.58E-07 |
| HDAC6 | TMEM147-AS1 | 0.302868872 | 0.000127867 |
| IKBKB | TMEM147-AS1 | 0.371529266 | 1.94E-06 |
| TSC1 | TMEM147-AS1 | 0.323963383 | 3.92E-05 |
| ULK3 | TMEM147-AS1 | 0.344193977 | 1.16E-05 |
| ATG10 | AC008771.1 | 0.571810276 | 7.78E-15 |
| ATG16L2 | AC008771.1 | 0.314679819 | 6.67E-05 |
| ATG4C | AC008771.1 | 0.356433855 | 5.31E-06 |
| ATG4D | AC008771.1 | 0.328361784 | 3.03E-05 |
| CAPN10 | AC008771.1 | 0.360542204 | 4.06E-06 |
| CLN3 | AC008771.1 | 0.379185639 | 1.14E-06 |
| GABARAP | AC008771.1 | 0.321182929 | 4.60E-05 |
| HDAC1 | AC008771.1 | 0.30915882 | 9.07E-05 |
| HDAC6 | AC008771.1 | 0.39979 | 2.56E-07 |
| IKBKE | AC008771.1 | 0.414117689 | 8.49E-08 |
| MAPK8 | AC008771.1 | 0.324198608 | 3.87E-05 |
| MTMR14 | AC008771.1 | 0.436327722 | 1.38E-08 |
| RAF1 | AC008771.1 | 0.319508974 | 5.07E-05 |
| ULK3 | AC008771.1 | 0.409332166 | 1.23E-07 |
| WDR45 | AC008771.1 | 0.373175652 | 1.73E-06 |
| ATF6 | AP003119.3 | 0.366270644 | 2.77E-06 |
| GABARAPL1 | AP003119.3 | 0.353995392 | 6.22E-06 |
| PELP1 | AP003119.3 | 0.344158864 | 1.16E-05 |
| PIK3C3 | AP003119.3 | 0.446898384 | 5.57E-09 |
| RHEB | AP003119.3 | 0.30118885 | 0.000139962 |
| SQSTM1 | AP003119.3 | 0.334624852 | 2.08E-05 |
| TP63 | AP003119.3 | 0.311174714 | 8.11E-05 |
| UVRAG | AP003119.3 | 0.448527374 | 4.83E-09 |
| VAMP3 | AP003119.3 | -0.335426837 | 1.99E-05 |
| ATG2B | AL122035.1 | 0.377991183 | 1.24E-06 |
| EIF2S1 | AL122035.1 | 0.360095909 | 4.18E-06 |
| GOPC | AL122035.1 | 0.303780041 | 0.000121723 |
| NPC1 | AL122035.1 | 0.310842036 | 8.26E-05 |
| PEX3 | AL122035.1 | 0.329701696 | 2.80E-05 |
| PTEN | AL122035.1 | 0.313811684 | 7.00E-05 |
| TP73 | AL122035.1 | 0.333648006 | 2.21E-05 |
| ZFYVE1 | AL122035.1 | 0.335816209 | 1.94E-05 |
| ATG9B | AC092171.3 | 0.304202953 | 0.000118966 |
| BAK1 | AC092171.3 | -0.302442078 | 0.000130844 |
| BID | AC092171.3 | 0.342065669 | 1.32E-05 |
| ITGA3 | AC092171.3 | -0.313710166 | 7.04E-05 |
| TP53INP2 | AC092171.3 | 0.328734826 | 2.96E-05 |
| ATG3 | LINC01389 | 0.389529964 | 5.45E-07 |
| BAG1 | LINC01389 | 0.321527215 | 4.51E-05 |
| CDKN2A | LINC01389 | 0.331476198 | 2.52E-05 |
| CHMP4B | LINC01389 | 0.327615753 | 3.17E-05 |
| RAF1 | LINC01389 | 0.301541728 | 0.000137336 |
| STK11 | LINC01389 | 0.395761155 | 3.45E-07 |
| ITGB4 | AL023754.1 | 0.306775255 | 0.000103412 |
| RAB1A | AL023754.1 | 0.353406372 | 6.46E-06 |
| ATG16L2 | AATBC | 0.306811626 | 0.000103206 |
| ATG4B | AATBC | 0.3245073 | 3.80E-05 |
| ATG4D | AATBC | 0.451309684 | 3.78E-09 |
| ATG9B | AATBC | 0.340286436 | 1.48E-05 |
| BID | AATBC | 0.338916796 | 1.61E-05 |
| CFLAR | AATBC | 0.417806236 | 6.34E-08 |
| HDAC6 | AATBC | 0.323505809 | 4.03E-05 |
| IKBKE | AATBC | 0.441246044 | 9.10E-09 |
| PRKAB1 | AATBC | 0.44140312 | 8.98E-09 |
| TP73 | AATBC | 0.373752292 | 1.67E-06 |
| TSC2 | AATBC | 0.350668504 | 7.70E-06 |
| WDR45 | AATBC | 0.37862743 | 1.19E-06 |
| ARNT | AC106782.2 | 0.30966549 | 8.82E-05 |
| CLN3 | AC106782.2 | 0.461817121 | 1.46E-09 |
| EEF2K | AC106782.2 | 0.301039291 | 0.000141089 |
| IKBKE | AC106782.2 | 0.387017377 | 6.54E-07 |
| MLST8 | AC106782.2 | 0.358882754 | 4.53E-06 |
| PARP1 | AC106782.2 | 0.320858487 | 4.69E-05 |
| PRKAB1 | AC106782.2 | 0.304494897 | 0.000117096 |
| TSC2 | AC106782.2 | 0.304247475 | 0.000118679 |
| ATG4A | AL049597.2 | 0.354957194 | 5.85E-06 |
| BNIP3 | AL049597.2 | -0.316605729 | 5.98E-05 |
| GABARAPL2 | AL049597.2 | 0.336154066 | 1.90E-05 |
| HDAC1 | AL049597.2 | 0.357526494 | 4.95E-06 |
| RAB11A | AL049597.2 | 0.368558689 | 2.38E-06 |
| SH3GLB1 | AL049597.2 | 0.439155248 | 1.09E-08 |
| ULK3 | AL049597.2 | 0.370099834 | 2.14E-06 |
| ARSB | AL138789.1 | -0.36607783 | 2.81E-06 |
| CASP1 | AL138789.1 | 0.363065699 | 3.43E-06 |
| CASP4 | AL138789.1 | 0.353717673 | 6.33E-06 |
| FKBP1B | AL138789.1 | 0.319636068 | 5.03E-05 |
| FOXO1 | AL138789.1 | -0.32725007 | 3.23E-05 |
| ITGA6 | AL138789.1 | 0.308954507 | 9.17E-05 |
| LAMP2 | AL138789.1 | -0.31555109 | 6.35E-05 |
| ULK2 | AL138789.1 | -0.307167984 | 0.000101212 |
| ARNT | AC026979.2 | -0.337478144 | 1.75E-05 |
| ATF4 | AC026979.2 | 0.36790979 | 2.48E-06 |
| ATG4B | AC026979.2 | 0.343095895 | 1.24E-05 |
| BAG1 | AC026979.2 | 0.331762072 | 2.48E-05 |
| BAX | AC026979.2 | 0.547827563 | 1.62E-13 |
| BECN1 | AC026979.2 | -0.330150609 | 2.72E-05 |
| BIRC5 | AC026979.2 | 0.506837055 | 1.71E-11 |
| BIRC6 | AC026979.2 | -0.308753066 | 9.28E-05 |
| BNIP1 | AC026979.2 | 0.4141204 | 8.49E-08 |
| CAPN10 | AC026979.2 | 0.323007148 | 4.14E-05 |
| CAPN2 | AC026979.2 | -0.390689369 | 5.01E-07 |
| EIF4EBP1 | AC026979.2 | 0.402129625 | 2.14E-07 |
| FKBP1A | AC026979.2 | 0.446039949 | 6.01E-09 |
| HGS | AC026979.2 | -0.327979031 | 3.10E-05 |
| MAPK1 | AC026979.2 | -0.364969853 | 3.02E-06 |
| MTOR | AC026979.2 | -0.304931363 | 0.000114353 |
| NFKB1 | AC026979.2 | -0.342332429 | 1.30E-05 |
| WDR45 | AC026979.2 | 0.432601585 | 1.89E-08 |
| BID | MAFA-AS1 | 0.358156822 | 4.75E-06 |
| GABARAPL2 | MAFA-AS1 | 0.341767332 | 1.35E-05 |
| AMBRA1 | AC022034.1 | 0.329063863 | 2.91E-05 |
| BECN1 | AC022034.1 | 0.31039536 | 8.47E-05 |
| IKBKB | AC022034.1 | 0.447606471 | 5.24E-09 |
| MTOR | AC022034.1 | 0.334375069 | 2.12E-05 |
| NBR1 | AC022034.1 | 0.349065406 | 8.53E-06 |
| RB1CC1 | AC022034.1 | 0.307660594 | 9.85E-05 |
| TM9SF1 | AC022034.1 | 0.330985949 | 2.59E-05 |
| FKBP1B | LINC02582 | 0.340477256 | 1.46E-05 |
| ITGA6 | LINC02582 | 0.355321181 | 5.71E-06 |
| ATG16L2 | AC060766.4 | 0.351286488 | 7.40E-06 |
| ATG4B | AC060766.4 | 0.389025883 | 5.66E-07 |
| BAX | AC060766.4 | 0.309636316 | 8.84E-05 |
| CAPN10 | AC060766.4 | 0.455191681 | 2.67E-09 |
| CX3CL1 | AC060766.4 | 0.311024503 | 8.18E-05 |
| CXCR4 | AC060766.4 | 0.521963859 | 3.29E-12 |
| DAPK2 | AC060766.4 | 0.408488941 | 1.32E-07 |
| TSC2 | AC060766.4 | 0.364587749 | 3.10E-06 |
| USP10 | AC060766.4 | -0.310316754 | 8.51E-05 |
| BAK1 | AL022323.1 | -0.32851006 | 3.00E-05 |
| BIRC6 | AL022323.1 | 0.530290108 | 1.28E-12 |
| EIF2AK3 | AL022323.1 | 0.497677639 | 4.45E-11 |
| EIF4G1 | AL022323.1 | 0.310482499 | 8.43E-05 |
| ERN1 | AL022323.1 | 0.33276463 | 2.33E-05 |
| FOXO3 | AL022323.1 | 0.332221852 | 2.41E-05 |
| GABARAPL1 | AL022323.1 | 0.345193273 | 1.09E-05 |
| ITGA3 | AL022323.1 | -0.31250415 | 7.53E-05 |
| KLHL24 | AL022323.1 | 0.377553601 | 1.28E-06 |
| MAPK1 | AL022323.1 | 0.353441402 | 6.45E-06 |
| MBTPS2 | AL022323.1 | 0.357349974 | 5.00E-06 |
| NBR1 | AL022323.1 | 0.422161304 | 4.47E-08 |
| NCKAP1 | AL022323.1 | 0.405463675 | 1.66E-07 |
| NFE2L2 | AL022323.1 | 0.557732971 | 4.75E-14 |
| PEX14 | AL022323.1 | -0.307168787 | 0.000101207 |
| PRKAB1 | AL022323.1 | 0.371219717 | 1.98E-06 |
| RB1CC1 | AL022323.1 | 0.383243599 | 8.57E-07 |
| RHEB | AL022323.1 | 0.326364423 | 3.41E-05 |
| TP63 | AL022323.1 | 0.336470798 | 1.86E-05 |
| WDFY3 | AL022323.1 | 0.378059093 | 1.24E-06 |
| WIPI1 | AL022323.1 | 0.30610701 | 0.000107259 |
| BAX | AL359182.1 | -0.321643902 | 4.48E-05 |
| EIF4G1 | AL359182.1 | 0.314374748 | 6.78E-05 |
| TP63 | AL359182.1 | 0.359941771 | 4.22E-06 |
| ATF4 | EPB41L4A-AS1 | 0.427431429 | 2.91E-08 |
| ATG10 | EPB41L4A-AS1 | 0.426786262 | 3.07E-08 |
| ATG12 | EPB41L4A-AS1 | 0.327868129 | 3.12E-05 |
| ATG16L2 | EPB41L4A-AS1 | 0.301685194 | 0.000136282 |
| ATG4B | EPB41L4A-AS1 | 0.431826418 | 2.02E-08 |
| ATG4C | EPB41L4A-AS1 | 0.302447543 | 0.000130805 |
| ATG4D | EPB41L4A-AS1 | 0.341186535 | 1.40E-05 |
| ATIC | EPB41L4A-AS1 | 0.44002299 | 1.01E-08 |
| BAG1 | EPB41L4A-AS1 | 0.342909217 | 1.26E-05 |
| BAG3 | EPB41L4A-AS1 | -0.40005053 | 2.51E-07 |
| BAX | EPB41L4A-AS1 | 0.478638699 | 2.99E-10 |
| BIRC5 | EPB41L4A-AS1 | 0.349292202 | 8.41E-06 |
| BNIP1 | EPB41L4A-AS1 | 0.542388642 | 3.11E-13 |
| CAPN10 | EPB41L4A-AS1 | 0.576343029 | 4.26E-15 |
| CAPN2 | EPB41L4A-AS1 | -0.350571688 | 7.75E-06 |
| CDKN1B | EPB41L4A-AS1 | 0.326796602 | 3.32E-05 |
| CDKN2A | EPB41L4A-AS1 | 0.370460425 | 2.09E-06 |
| CLN3 | EPB41L4A-AS1 | 0.300766476 | 0.000143166 |
| CXCR4 | EPB41L4A-AS1 | 0.422035381 | 4.51E-08 |
| DIRAS3 | EPB41L4A-AS1 | 0.357406487 | 4.99E-06 |
| EEF2 | EPB41L4A-AS1 | 0.343096625 | 1.24E-05 |
| EIF4G1 | EPB41L4A-AS1 | -0.372439291 | 1.82E-06 |
| FKBP1A | EPB41L4A-AS1 | 0.394394017 | 3.82E-07 |
| GABARAP | EPB41L4A-AS1 | 0.408609663 | 1.30E-07 |
| HIF1A | EPB41L4A-AS1 | -0.388115762 | 6.04E-07 |
| ITGA6 | EPB41L4A-AS1 | -0.395464087 | 3.53E-07 |
| ITGB1 | EPB41L4A-AS1 | -0.319268368 | 5.14E-05 |
| ITGB4 | EPB41L4A-AS1 | -0.301472966 | 0.000137844 |
| MAP1LC3A | EPB41L4A-AS1 | 0.3948825 | 3.68E-07 |
| MAP2K7 | EPB41L4A-AS1 | 0.307615652 | 9.88E-05 |
| MAPK1 | EPB41L4A-AS1 | -0.431404776 | 2.09E-08 |
| MAPK8IP1 | EPB41L4A-AS1 | 0.355101111 | 5.79E-06 |
| MLST8 | EPB41L4A-AS1 | 0.31779922 | 5.59E-05 |
| MTMR14 | EPB41L4A-AS1 | 0.441127178 | 9.20E-09 |
| NCKAP1 | EPB41L4A-AS1 | -0.360881485 | 3.97E-06 |
| PEX14 | EPB41L4A-AS1 | 0.304210252 | 0.000118919 |
| PRKAB1 | EPB41L4A-AS1 | 0.327585424 | 3.17E-05 |
| RAB1A | EPB41L4A-AS1 | -0.337995427 | 1.70E-05 |
| RAB24 | EPB41L4A-AS1 | 0.4109992 | 1.08E-07 |
| STK11 | EPB41L4A-AS1 | 0.371693732 | 1.92E-06 |
| TP63 | EPB41L4A-AS1 | -0.358183437 | 4.74E-06 |
| TSC2 | EPB41L4A-AS1 | 0.316209482 | 6.12E-05 |
| ULK3 | EPB41L4A-AS1 | 0.330913417 | 2.60E-05 |
| USP10 | EPB41L4A-AS1 | -0.313214799 | 7.24E-05 |
| WDFY3 | EPB41L4A-AS1 | -0.351988435 | 7.08E-06 |
| WDR45 | EPB41L4A-AS1 | 0.494809131 | 5.98E-11 |
| ARSB | AC004656.1 | 0.344394992 | 1.14E-05 |
| ATG10 | AC004656.1 | 0.328639309 | 2.98E-05 |
| ATG4C | AC004656.1 | 0.411812257 | 1.02E-07 |
| ATIC | AC004656.1 | 0.347942717 | 9.16E-06 |
| BCL2 | AC004656.1 | 0.365085797 | 3.00E-06 |
| BIRC5 | AC004656.1 | 0.436137499 | 1.41E-08 |
| BNIP3L | AC004656.1 | 0.314147058 | 6.87E-05 |
| CASP4 | AC004656.1 | -0.319372404 | 5.11E-05 |
| CDKN1B | AC004656.1 | 0.397655839 | 3.00E-07 |
| CX3CL1 | AC004656.1 | 0.300759549 | 0.000143219 |
| CXCR4 | AC004656.1 | 0.347189344 | 9.60E-06 |
| DRAM1 | AC004656.1 | 0.379582573 | 1.11E-06 |
| FAS | AC004656.1 | 0.334521661 | 2.10E-05 |
| GABARAP | AC004656.1 | 0.362554316 | 3.55E-06 |
| HDAC6 | AC004656.1 | 0.639600221 | 3.32E-19 |
| MAPK8IP1 | AC004656.1 | 0.558849672 | 4.13E-14 |
| MAPK9 | AC004656.1 | 0.399331668 | 2.64E-07 |
| MBTPS2 | AC004656.1 | 0.323112383 | 4.12E-05 |
| MTMR14 | AC004656.1 | 0.335098462 | 2.03E-05 |
| MYC | AC004656.1 | -0.30597438 | 0.000108038 |
| NKX2-3 | AC004656.1 | 0.416327206 | 7.13E-08 |
| PARP1 | AC004656.1 | 0.408835119 | 1.28E-07 |
| PRKAR1A | AC004656.1 | 0.507198662 | 1.64E-11 |
| PTK6 | AC004656.1 | -0.363832617 | 3.26E-06 |
| TP53 | AC004656.1 | 0.436995205 | 1.31E-08 |
| TP73 | AC004656.1 | 0.389839812 | 5.33E-07 |
| TSC2 | AC004656.1 | 0.396159383 | 3.35E-07 |
| ULK2 | AC004656.1 | 0.44185236 | 8.64E-09 |
| VAMP7 | AC004656.1 | 0.361966094 | 3.69E-06 |
| WDR45 | AC004656.1 | 0.375656166 | 1.46E-06 |
| DNAJB9 | LINC01436 | 0.432342674 | 1.94E-08 |
| ATG10 | AL391244.1 | 0.364940758 | 3.03E-06 |
| ATG16L2 | AL391244.1 | 0.410629106 | 1.12E-07 |
| ATG3 | AL391244.1 | 0.365354525 | 2.95E-06 |
| ATG4A | AL391244.1 | 0.337367175 | 1.76E-05 |
| ATG4B | AL391244.1 | 0.388916025 | 5.70E-07 |
| BAX | AL391244.1 | 0.348241821 | 8.98E-06 |
| BNIP1 | AL391244.1 | 0.390404623 | 5.12E-07 |
| CAPN10 | AL391244.1 | 0.461592584 | 1.49E-09 |
| CFLAR | AL391244.1 | 0.48440564 | 1.70E-10 |
| CLN3 | AL391244.1 | 0.410733062 | 1.11E-07 |
| FOXO1 | AL391244.1 | -0.314361438 | 6.79E-05 |
| MAP1LC3A | AL391244.1 | 0.316137662 | 6.14E-05 |
| MTMR14 | AL391244.1 | 0.359691633 | 4.29E-06 |
| PEX14 | AL391244.1 | 0.570587098 | 9.13E-15 |
| RAB24 | AL391244.1 | 0.360490927 | 4.07E-06 |
| ULK3 | AL391244.1 | 0.335585579 | 1.97E-05 |
| VAMP3 | AL391244.1 | 0.369660154 | 2.20E-06 |
| WDR45 | AL391244.1 | 0.414831131 | 8.02E-08 |
| RAB1A | LINC01160 | 0.325253345 | 3.64E-05 |
| ATG10 | AC073508.3 | 0.32564082 | 3.55E-05 |
| CAPNS1 | AC073508.3 | 0.374451943 | 1.59E-06 |
| CLN3 | AC073508.3 | 0.377167862 | 1.31E-06 |
| GABARAP | AC073508.3 | 0.313702134 | 7.05E-05 |
| HDAC6 | AC073508.3 | 0.318385295 | 5.41E-05 |
| IKBKE | AC073508.3 | 0.387188704 | 6.46E-07 |
| ITGA3 | AC073508.3 | -0.314297564 | 6.81E-05 |
| ITGA6 | AC073508.3 | -0.304275639 | 0.000118498 |
| WDR45 | AC073508.3 | 0.398101028 | 2.90E-07 |
| ARSB | LINC01614 | 0.432949839 | 1.84E-08 |
| DLC1 | LINC01614 | 0.436303771 | 1.39E-08 |
| FOXO1 | LINC01614 | 0.35280193 | 6.72E-06 |
| GABARAPL1 | LINC01614 | 0.316389076 | 6.06E-05 |
| PEA15 | LINC01614 | 0.371157287 | 1.99E-06 |
| ARNT | AF131215.5 | 0.328159191 | 3.07E-05 |
| ARSB | AF131215.5 | 0.398414943 | 2.83E-07 |
| ATG2A | AF131215.5 | 0.339001977 | 1.60E-05 |
| ATG4C | AF131215.5 | 0.39780286 | 2.97E-07 |
| ATG7 | AF131215.5 | 0.400881753 | 2.35E-07 |
| BCL2 | AF131215.5 | 0.676493807 | 4.45E-22 |
| BNIP3 | AF131215.5 | -0.322238108 | 4.33E-05 |
| BNIP3L | AF131215.5 | 0.331209289 | 2.56E-05 |
| CAMKK2 | AF131215.5 | 0.313941854 | 6.95E-05 |
| CCR2 | AF131215.5 | 0.406313681 | 1.56E-07 |
| CD46 | AF131215.5 | 0.350330315 | 7.87E-06 |
| CDKN1B | AF131215.5 | 0.453277401 | 3.17E-09 |
| CFLAR | AF131215.5 | 0.344942761 | 1.11E-05 |
| CX3CL1 | AF131215.5 | 0.361670335 | 3.77E-06 |
| CXCR4 | AF131215.5 | 0.517082203 | 5.64E-12 |
| DAPK1 | AF131215.5 | 0.319668046 | 5.02E-05 |
| DRAM1 | AF131215.5 | 0.409566672 | 1.21E-07 |
| EEF2 | AF131215.5 | 0.343086783 | 1.24E-05 |
| EEF2K | AF131215.5 | 0.527478214 | 1.77E-12 |
| EIF2AK3 | AF131215.5 | 0.321810348 | 4.44E-05 |
| FAS | AF131215.5 | 0.452547871 | 3.38E-09 |
| HDAC6 | AF131215.5 | 0.448384983 | 4.89E-09 |
| HSPA5 | AF131215.5 | -0.351706261 | 7.21E-06 |
| IFNG | AF131215.5 | 0.316906576 | 5.88E-05 |
| IKBKB | AF131215.5 | 0.374008174 | 1.64E-06 |
| IKBKE | AF131215.5 | 0.441493205 | 8.91E-09 |
| ITPR1 | AF131215.5 | 0.421802232 | 4.60E-08 |
| KLHL24 | AF131215.5 | 0.403092374 | 1.99E-07 |
| MAP2K7 | AF131215.5 | 0.499688633 | 3.61E-11 |
| MAPK8 | AF131215.5 | 0.439195337 | 1.09E-08 |
| MTMR14 | AF131215.5 | 0.4129521 | 9.30E-08 |
| MTOR | AF131215.5 | 0.312004012 | 7.75E-05 |
| NBR1 | AF131215.5 | 0.36195458 | 3.70E-06 |
| NKX2-3 | AF131215.5 | 0.544691274 | 2.36E-13 |
| PARP1 | AF131215.5 | 0.466071041 | 9.87E-10 |
| PIK3R4 | AF131215.5 | 0.443568524 | 7.45E-09 |
| PRKAB1 | AF131215.5 | 0.436622499 | 1.35E-08 |
| RAB33B | AF131215.5 | 0.300072068 | 0.000148583 |
| RAF1 | AF131215.5 | 0.549507029 | 1.32E-13 |
| SIRT1 | AF131215.5 | 0.399121168 | 2.69E-07 |
| TP53 | AF131215.5 | 0.547368527 | 1.71E-13 |
| TP73 | AF131215.5 | 0.486290799 | 1.41E-10 |
| TSC1 | AF131215.5 | 0.408416051 | 1.32E-07 |
| TSC2 | AF131215.5 | 0.629319834 | 1.79E-18 |
| UVRAG | AF131215.5 | 0.354967635 | 5.84E-06 |
| ATG4A | AP000251.1 | 0.340165889 | 1.49E-05 |
| ATG4C | AP000251.1 | 0.339278014 | 1.57E-05 |
| ATIC | AP000251.1 | 0.585229435 | 1.28E-15 |
| BAX | AP000251.1 | 0.340541499 | 1.45E-05 |
| BIRC5 | AP000251.1 | 0.617078043 | 1.23E-17 |
| CDKN1B | AP000251.1 | 0.309950393 | 8.68E-05 |
| CDKN2A | AP000251.1 | 0.337497938 | 1.75E-05 |
| FKBP1A | AP000251.1 | 0.332306401 | 2.40E-05 |
| HDAC6 | AP000251.1 | 0.425612072 | 3.38E-08 |
| MAPK8IP1 | AP000251.1 | 0.769267521 | 1.43E-31 |
| MLST8 | AP000251.1 | 0.342312238 | 1.30E-05 |
| MTMR14 | AP000251.1 | 0.374285108 | 1.61E-06 |
| PRKAR1A | AP000251.1 | 0.51506951 | 7.03E-12 |
| VAMP7 | AP000251.1 | 0.389664833 | 5.40E-07 |
| WDR45 | AP000251.1 | 0.354572541 | 5.99E-06 |
| ATG2B | AL118506.1 | 0.350123892 | 7.97E-06 |
| BAX | AL118506.1 | -0.38536896 | 7.36E-07 |
| BNIP1 | AL118506.1 | -0.392844919 | 4.28E-07 |
| EIF4G1 | AL118506.1 | 0.374956298 | 1.53E-06 |
| GABARAP | AL118506.1 | -0.357515391 | 4.95E-06 |
| HIF1A | AL118506.1 | 0.360485262 | 4.07E-06 |
| HSPA8 | AL118506.1 | 0.353827212 | 6.29E-06 |
| KIF5B | AL118506.1 | 0.329275431 | 2.87E-05 |
| MYC | AL118506.1 | 0.33836598 | 1.66E-05 |
| NCKAP1 | AL118506.1 | 0.353946548 | 6.24E-06 |
| PTK6 | AL118506.1 | 0.320343641 | 4.83E-05 |
| RAB1A | AL118506.1 | 0.346011918 | 1.03E-05 |
| RPTOR | AL118506.1 | 0.407529868 | 1.42E-07 |
| TP53INP2 | AL118506.1 | 0.362217514 | 3.63E-06 |
| USP10 | AL118506.1 | 0.411588716 | 1.03E-07 |
| WDFY3 | AL118506.1 | 0.362453097 | 3.58E-06 |
| ZFYVE1 | AL118506.1 | 0.303157273 | 0.000125892 |
| ATF4 | AC025165.5 | 0.363552425 | 3.32E-06 |
| ATG4B | AC025165.5 | 0.403786434 | 1.89E-07 |
| ATG4D | AC025165.5 | 0.407813294 | 1.39E-07 |
| BAX | AC025165.5 | 0.34785051 | 9.21E-06 |
| BNIP1 | AC025165.5 | 0.350960514 | 7.56E-06 |
| CAPN10 | AC025165.5 | 0.442931538 | 7.87E-09 |
| CDKN1B | AC025165.5 | 0.350395519 | 7.84E-06 |
| CHMP4B | AC025165.5 | 0.307546918 | 9.91E-05 |
| CXCR4 | AC025165.5 | 0.343326714 | 1.22E-05 |
| DAPK2 | AC025165.5 | 0.360195587 | 4.15E-06 |
| DDIT3 | AC025165.5 | 0.347152984 | 9.62E-06 |
| IKBKB | AC025165.5 | 0.328681104 | 2.97E-05 |
| MAP2K7 | AC025165.5 | 0.414886426 | 7.99E-08 |
| PELP1 | AC025165.5 | 0.355334322 | 5.71E-06 |
| STK11 | AC025165.5 | 0.328473816 | 3.01E-05 |
| USP10 | AC025165.5 | -0.30738792 | 1.00E-04 |
| CD46 | HOXA-AS2 | 0.391817234 | 4.61E-07 |
| IKBKB | HOXA-AS2 | 0.352762121 | 6.74E-06 |
| PRKAB1 | HOXA-AS2 | 0.300215598 | 0.000147448 |
| SH3GLB1 | HOXA-AS2 | 0.312091028 | 7.71E-05 |
| PEX14 | AL589765.4 | 0.339034496 | 1.59E-05 |
| TP73 | AL589765.4 | 0.31133338 | 8.04E-05 |
| AMBRA1 | DGUOK-AS1 | -0.350047277 | 8.01E-06 |
| ARSB | DGUOK-AS1 | -0.413667861 | 8.79E-08 |
| ATG16L2 | DGUOK-AS1 | 0.327983041 | 3.10E-05 |
| ATG2A | DGUOK-AS1 | -0.345652513 | 1.06E-05 |
| ATG9B | DGUOK-AS1 | 0.333254158 | 2.26E-05 |
| CANX | DGUOK-AS1 | -0.375264871 | 1.50E-06 |
| CAPN10 | DGUOK-AS1 | 0.333615775 | 2.21E-05 |
| DLC1 | DGUOK-AS1 | -0.31449883 | 6.74E-05 |
| FAS | DGUOK-AS1 | -0.414954585 | 7.95E-08 |
| FOXO1 | DGUOK-AS1 | -0.329059346 | 2.91E-05 |
| MAPK9 | DGUOK-AS1 | -0.343975183 | 1.17E-05 |
| PTEN | DGUOK-AS1 | -0.310481066 | 8.43E-05 |
| GABARAPL1 | LINC00519 | 0.301549286 | 0.000137281 |
| NFE2L2 | LINC00519 | 0.42280719 | 4.24E-08 |
| TP63 | LINC00519 | 0.429285354 | 2.50E-08 |
| ATF4 | RPARP-AS1 | 0.343214664 | 1.23E-05 |
| ATG16L2 | RPARP-AS1 | 0.44821459 | 4.97E-09 |
| ATG4B | RPARP-AS1 | 0.434008179 | 1.68E-08 |
| ATG4C | RPARP-AS1 | 0.315505711 | 6.37E-05 |
| ATIC | RPARP-AS1 | 0.418763914 | 5.87E-08 |
| BAX | RPARP-AS1 | 0.391847459 | 4.60E-07 |
| BIRC5 | RPARP-AS1 | 0.42055313 | 5.09E-08 |
| BNIP1 | RPARP-AS1 | 0.356043142 | 5.45E-06 |
| CAPN10 | RPARP-AS1 | 0.550854873 | 1.12E-13 |
| CDKN1B | RPARP-AS1 | 0.323810458 | 3.96E-05 |
| CLN3 | RPARP-AS1 | 0.390153988 | 5.21E-07 |
| CXCR4 | RPARP-AS1 | 0.396952823 | 3.16E-07 |
| FOXO1 | RPARP-AS1 | -0.303830557 | 0.00012139 |
| GABARAPL2 | RPARP-AS1 | 0.31006258 | 8.63E-05 |
| HDAC6 | RPARP-AS1 | 0.364761368 | 3.07E-06 |
| HIF1A | RPARP-AS1 | -0.307824139 | 9.76E-05 |
| ITGA3 | RPARP-AS1 | -0.396931568 | 3.16E-07 |
| ITGA6 | RPARP-AS1 | -0.333358875 | 2.25E-05 |
| ITGB1 | RPARP-AS1 | -0.330129501 | 2.73E-05 |
| ITGB4 | RPARP-AS1 | -0.372701468 | 1.79E-06 |
| MAP1LC3A | RPARP-AS1 | 0.302202757 | 0.000132541 |
| MAPK8IP1 | RPARP-AS1 | 0.384937691 | 7.59E-07 |
| MLST8 | RPARP-AS1 | 0.424133534 | 3.81E-08 |
| MTMR14 | RPARP-AS1 | 0.34144967 | 1.37E-05 |
| PELP1 | RPARP-AS1 | 0.327160718 | 3.25E-05 |
| PINK1 | RPARP-AS1 | -0.389795859 | 5.35E-07 |
| STK11 | RPARP-AS1 | 0.334249123 | 2.13E-05 |
| ULK3 | RPARP-AS1 | 0.436152607 | 1.41E-08 |
| WDR45 | RPARP-AS1 | 0.504173045 | 2.26E-11 |
| ZFYVE1 | RPARP-AS1 | -0.329363621 | 2.86E-05 |
| BNIP1 | AC103702.2 | 0.307096902 | 0.000101607 |
| ATG16L2 | AC027020.2 | 0.325514989 | 3.58E-05 |
| ATG3 | AC027020.2 | 0.30791674 | 9.71E-05 |
| ATG4B | AC027020.2 | 0.337407486 | 1.76E-05 |
| CAPN10 | AC027020.2 | 0.377880016 | 1.25E-06 |
| CXCR4 | AC027020.2 | 0.360733751 | 4.01E-06 |
| DAPK2 | AC027020.2 | 0.389248332 | 5.57E-07 |
| IKBKB | AC027020.2 | 0.399171017 | 2.68E-07 |
| MAP2K7 | AC027020.2 | 0.318694449 | 5.31E-05 |
| PINK1 | AC027020.2 | -0.317478082 | 5.69E-05 |
| RAF1 | AC027020.2 | 0.334845404 | 2.06E-05 |
| STK11 | AC027020.2 | 0.346419214 | 1.01E-05 |
| TSC1 | AC027020.2 | 0.366747955 | 2.68E-06 |
| ULK3 | AC027020.2 | 0.469278895 | 7.31E-10 |
| ATG16L2 | AP001318.2 | 0.325513214 | 3.58E-05 |
| CAPN10 | AP001318.2 | 0.302034058 | 0.00013375 |
| GNAI3 | AP001318.2 | -0.311622006 | 7.91E-05 |
| UVRAG | AP001318.2 | 0.348773284 | 8.69E-06 |
| ATG7 | AL357033.4 | 0.306457778 | 0.000105223 |
| ATG9B | AL357033.4 | 0.329490928 | 2.83E-05 |
| CAPNS1 | AL357033.4 | 0.330567223 | 2.66E-05 |
| CFLAR | AL357033.4 | 0.397726103 | 2.98E-07 |
| HSP90AB1 | AL357033.4 | -0.326277607 | 3.42E-05 |
| IKBKE | AL357033.4 | 0.316722744 | 5.94E-05 |
| MAPK3 | AL357033.4 | 0.312246252 | 7.64E-05 |
| RAB24 | AL357033.4 | 0.494041215 | 6.46E-11 |
| ST13 | AL357033.4 | -0.371363915 | 1.96E-06 |
| ATG16L2 | PCAT19 | 0.316867349 | 5.89E-05 |
| BID | PCAT19 | 0.320890402 | 4.68E-05 |
| DAPK2 | PCAT19 | 0.421499429 | 4.71E-08 |
| PELP1 | PCAT19 | 0.45641425 | 2.39E-09 |
| ATG10 | AL031673.1 | 0.36479594 | 3.06E-06 |
| ATG12 | AL031673.1 | 0.317597179 | 5.65E-05 |
| ATG3 | AL031673.1 | 0.36367266 | 3.30E-06 |
| ATG4A | AL031673.1 | 0.384181691 | 8.01E-07 |
| ATG4B | AL031673.1 | 0.375924949 | 1.43E-06 |
| ATG4D | AL031673.1 | 0.30338107 | 0.000124379 |
| ATIC | AL031673.1 | 0.367757548 | 2.51E-06 |
| BAG1 | AL031673.1 | 0.310623656 | 8.37E-05 |
| BAX | AL031673.1 | 0.362569135 | 3.55E-06 |
| BIRC5 | AL031673.1 | 0.478111238 | 3.15E-10 |
| BNIP1 | AL031673.1 | 0.361897355 | 3.71E-06 |
| CAPN10 | AL031673.1 | 0.384520483 | 7.82E-07 |
| CDKN1B | AL031673.1 | 0.346188083 | 1.02E-05 |
| CDKN2A | AL031673.1 | 0.397994052 | 2.92E-07 |
| CXCR4 | AL031673.1 | 0.308773614 | 9.27E-05 |
| HDAC6 | AL031673.1 | 0.454585813 | 2.82E-09 |
| HIF1A | AL031673.1 | -0.395101042 | 3.62E-07 |
| HSPA5 | AL031673.1 | -0.347881721 | 9.19E-06 |
| IKBKE | AL031673.1 | 0.314137956 | 6.87E-05 |
| ITGB4 | AL031673.1 | -0.307171865 | 0.00010119 |
| MAP1LC3A | AL031673.1 | 0.302467119 | 0.000130668 |
| MAP2K7 | AL031673.1 | 0.341127431 | 1.40E-05 |
| MAPK8 | AL031673.1 | 0.335021225 | 2.03E-05 |
| MAPK8IP1 | AL031673.1 | 0.338522073 | 1.64E-05 |
| MLST8 | AL031673.1 | 0.342297815 | 1.30E-05 |
| MTMR14 | AL031673.1 | 0.39345996 | 4.09E-07 |
| NKX2-3 | AL031673.1 | 0.348663763 | 8.75E-06 |
| P4HB | AL031673.1 | -0.314241718 | 6.83E-05 |
| PARP1 | AL031673.1 | 0.339570193 | 1.54E-05 |
| PINK1 | AL031673.1 | -0.365451516 | 2.93E-06 |
| PRKAB1 | AL031673.1 | 0.355146693 | 5.78E-06 |
| RAF1 | AL031673.1 | 0.37863959 | 1.19E-06 |
| STK11 | AL031673.1 | 0.405138837 | 1.70E-07 |
| TP53 | AL031673.1 | 0.333342709 | 2.25E-05 |
| TP73 | AL031673.1 | 0.313554939 | 7.10E-05 |
| TSC2 | AL031673.1 | 0.312093163 | 7.71E-05 |
| ULK3 | AL031673.1 | 0.322601481 | 4.24E-05 |
| WDR45 | AL031673.1 | 0.525153157 | 2.30E-12 |
| ATG12 | AL355488.1 | 0.430961036 | 2.17E-08 |
| ATG16L2 | AL355488.1 | 0.333381404 | 2.25E-05 |
| ATG3 | AL355488.1 | 0.425811381 | 3.32E-08 |
| ATG4B | AL355488.1 | 0.391192692 | 4.83E-07 |
| ATG4C | AL355488.1 | 0.488072526 | 1.18E-10 |
| BAG3 | AL355488.1 | -0.307421659 | 9.98E-05 |
| BAX | AL355488.1 | 0.368670199 | 2.36E-06 |
| BCL2 | AL355488.1 | 0.37705673 | 1.32E-06 |
| BIRC5 | AL355488.1 | 0.334173853 | 2.14E-05 |
| BNIP1 | AL355488.1 | 0.356322099 | 5.35E-06 |
| CAPN10 | AL355488.1 | 0.524120605 | 2.58E-12 |
| CDKN1B | AL355488.1 | 0.421948846 | 4.55E-08 |
| CDKN2A | AL355488.1 | 0.36160003 | 3.79E-06 |
| CXCR4 | AL355488.1 | 0.567559093 | 1.36E-14 |
| DAPK2 | AL355488.1 | 0.393148963 | 4.19E-07 |
| FAS | AL355488.1 | 0.338978056 | 1.60E-05 |
| HDAC6 | AL355488.1 | 0.475629281 | 4.00E-10 |
| IKBKB | AL355488.1 | 0.360120287 | 4.17E-06 |
| MAP2K7 | AL355488.1 | 0.38610403 | 6.98E-07 |
| MAPK8 | AL355488.1 | 0.362327117 | 3.61E-06 |
| MAPK9 | AL355488.1 | 0.32614835 | 3.45E-05 |
| MTMR14 | AL355488.1 | 0.40760449 | 1.41E-07 |
| NCKAP1 | AL355488.1 | -0.301564474 | 0.000137169 |
| NKX2-3 | AL355488.1 | 0.390715703 | 5.00E-07 |
| PINK1 | AL355488.1 | -0.366634639 | 2.70E-06 |
| PRKAB1 | AL355488.1 | 0.301079646 | 0.000140784 |
| PTK6 | AL355488.1 | -0.300462321 | 0.000145516 |
| RAF1 | AL355488.1 | 0.447380029 | 5.34E-09 |
| STK11 | AL355488.1 | 0.413012647 | 9.26E-08 |
| TP53 | AL355488.1 | 0.420087157 | 5.28E-08 |
| TP73 | AL355488.1 | 0.331155988 | 2.57E-05 |
| TSC1 | AL355488.1 | 0.332662724 | 2.35E-05 |
| TSC2 | AL355488.1 | 0.433489239 | 1.76E-08 |
| ULK3 | AL355488.1 | 0.457000552 | 2.27E-09 |
| WDFY3 | AL355488.1 | -0.309955447 | 8.68E-05 |
| WDR45 | AL355488.1 | 0.442741976 | 8.00E-09 |
| ARNT | SNHG19 | -0.340005326 | 1.50E-05 |
| ATF4 | SNHG19 | 0.37366434 | 1.68E-06 |
| ATG4B | SNHG19 | 0.353484766 | 6.43E-06 |
| ATIC | SNHG19 | 0.363838554 | 3.26E-06 |
| BAG1 | SNHG19 | 0.326213931 | 3.44E-05 |
| BAX | SNHG19 | 0.446375217 | 5.84E-09 |
| BIRC5 | SNHG19 | 0.528197581 | 1.63E-12 |
| BIRC6 | SNHG19 | -0.303303671 | 0.0001249 |
| BNIP1 | SNHG19 | 0.348629864 | 8.77E-06 |
| CAPN10 | SNHG19 | 0.355567679 | 5.62E-06 |
| CAPN2 | SNHG19 | -0.358945714 | 4.51E-06 |
| CLN3 | SNHG19 | 0.320549834 | 4.78E-05 |
| EIF4EBP1 | SNHG19 | 0.317143341 | 5.80E-05 |
| FKBP1A | SNHG19 | 0.343315884 | 1.22E-05 |
| GABARAPL2 | SNHG19 | 0.332627313 | 2.35E-05 |
| GOPC | SNHG19 | -0.305636409 | 0.000110048 |
| LAMP2 | SNHG19 | -0.315840226 | 6.25E-05 |
| MAPK1 | SNHG19 | -0.385290447 | 7.40E-07 |
| MAPK8IP1 | SNHG19 | 0.379877465 | 1.09E-06 |
| MLST8 | SNHG19 | 0.439662694 | 1.04E-08 |
| NFKB1 | SNHG19 | -0.340283266 | 1.48E-05 |
| SH3GLB1 | SNHG19 | -0.327589282 | 3.17E-05 |
| WDR45 | SNHG19 | 0.357378352 | 5.00E-06 |
| ATG7 | USP30-AS1 | 0.319684869 | 5.02E-05 |
| BAX | USP30-AS1 | 0.355080437 | 5.80E-06 |
| BNIP1 | USP30-AS1 | 0.317025834 | 5.84E-05 |
| CALCOCO2 | USP30-AS1 | 0.389224079 | 5.57E-07 |
| CAPNS1 | USP30-AS1 | 0.354141639 | 6.16E-06 |
| CASP1 | USP30-AS1 | 0.326881614 | 3.31E-05 |
| CCR2 | USP30-AS1 | 0.364101049 | 3.21E-06 |
| CDKN2A | USP30-AS1 | 0.395305537 | 3.57E-07 |
| CTSD | USP30-AS1 | 0.424754938 | 3.62E-08 |
| CX3CL1 | USP30-AS1 | 0.334183433 | 2.14E-05 |
| FAS | USP30-AS1 | 0.308475683 | 9.42E-05 |
| IFNG | USP30-AS1 | 0.629701823 | 1.68E-18 |
| MTMR14 | USP30-AS1 | 0.391116324 | 4.86E-07 |
| NCKAP1 | USP30-AS1 | -0.361482818 | 3.81E-06 |
| PEX14 | USP30-AS1 | 0.331470446 | 2.52E-05 |
| RAB24 | USP30-AS1 | 0.390387982 | 5.12E-07 |
| RGS19 | USP30-AS1 | 0.320125126 | 4.89E-05 |
| TNFSF10 | USP30-AS1 | 0.301630591 | 0.000136683 |
| WDR45 | USP30-AS1 | 0.31937943 | 5.11E-05 |
| ATG16L2 | AL355802.2 | 0.373048335 | 1.75E-06 |
| ATG4B | AL355802.2 | 0.393870834 | 3.97E-07 |
| BID | AL355802.2 | 0.424379419 | 3.73E-08 |
| CAPN10 | AL355802.2 | 0.323862621 | 3.94E-05 |
| DAPK2 | AL355802.2 | 0.498063514 | 4.28E-11 |
| PELP1 | AL355802.2 | 0.464292878 | 1.16E-09 |
| ATG12 | AP003469.4 | 0.354062452 | 6.19E-06 |
| EIF4G1 | AP003469.4 | -0.3042022 | 0.000118971 |
| CCR2 | AC012236.1 | 0.40625359 | 1.56E-07 |
| CXCR4 | AC012236.1 | 0.392038093 | 4.54E-07 |
| ERN1 | AC012236.1 | 0.373360754 | 1.71E-06 |
| ITPR1 | AC012236.1 | 0.305366743 | 0.000111676 |
| PRKCD | AC012236.1 | 0.313618012 | 7.08E-05 |
| RGS19 | AC012236.1 | 0.365311182 | 2.96E-06 |
| ATG16L2 | DCST1-AS1 | 0.330300122 | 2.70E-05 |
| ATG3 | DCST1-AS1 | 0.342658369 | 1.27E-05 |
| CAPN10 | DCST1-AS1 | 0.333960482 | 2.17E-05 |
| CDKN2A | DCST1-AS1 | 0.371097383 | 2.00E-06 |
| DAPK2 | DCST1-AS1 | 0.375464691 | 1.48E-06 |
| STK11 | DCST1-AS1 | 0.320609957 | 4.76E-05 |
| ULK3 | DCST1-AS1 | 0.383270007 | 8.55E-07 |
| ARNT | MIR193BHG | -0.418551795 | 5.97E-08 |
| ARSB | MIR193BHG | -0.439473392 | 1.06E-08 |
| ATF6 | MIR193BHG | -0.351841471 | 7.14E-06 |
| BCL2 | MIR193BHG | -0.353601689 | 6.38E-06 |
| BIRC6 | MIR193BHG | -0.429642851 | 2.42E-08 |
| BNIP3 | MIR193BHG | 0.394588492 | 3.76E-07 |
| CASP3 | MIR193BHG | -0.351751015 | 7.19E-06 |
| CD46 | MIR193BHG | -0.443614424 | 7.42E-09 |
| CDKN1B | MIR193BHG | -0.323067756 | 4.13E-05 |
| CX3CL1 | MIR193BHG | -0.304343975 | 0.000118059 |
| DAPK1 | MIR193BHG | -0.322496721 | 4.27E-05 |
| DRAM1 | MIR193BHG | -0.326879514 | 3.31E-05 |
| EEF2 | MIR193BHG | -0.341903345 | 1.34E-05 |
| EEF2K | MIR193BHG | -0.363851734 | 3.26E-06 |
| EIF2AK3 | MIR193BHG | -0.429517938 | 2.45E-08 |
| FAS | MIR193BHG | -0.314507468 | 6.73E-05 |
| FOXO1 | MIR193BHG | -0.326732543 | 3.33E-05 |
| FOXO3 | MIR193BHG | -0.325047416 | 3.68E-05 |
| HDAC6 | MIR193BHG | -0.430933444 | 2.18E-08 |
| IKBKE | MIR193BHG | -0.43433825 | 1.64E-08 |
| ITPR1 | MIR193BHG | -0.342929183 | 1.25E-05 |
| KLHL24 | MIR193BHG | -0.445633217 | 6.23E-09 |
| LAMP2 | MIR193BHG | -0.306022065 | 0.000107757 |
| MAPK1 | MIR193BHG | -0.324049995 | 3.90E-05 |
| MAPK8 | MIR193BHG | -0.326955842 | 3.29E-05 |
| MBTPS2 | MIR193BHG | -0.377089948 | 1.32E-06 |
| MTMR14 | MIR193BHG | -0.319679472 | 5.02E-05 |
| NBR1 | MIR193BHG | -0.451441013 | 3.73E-09 |
| NFE2L2 | MIR193BHG | -0.319232658 | 5.15E-05 |
| PARP1 | MIR193BHG | -0.537645412 | 5.46E-13 |
| PIK3R4 | MIR193BHG | -0.408775958 | 1.29E-07 |
| PRKAB1 | MIR193BHG | -0.352572564 | 6.82E-06 |
| PRKCD | MIR193BHG | -0.383896685 | 8.18E-07 |
| PTK6 | MIR193BHG | 0.398903622 | 2.73E-07 |
| RAB33B | MIR193BHG | -0.303284254 | 0.000125031 |
| RAF1 | MIR193BHG | -0.349239359 | 8.43E-06 |
| RB1CC1 | MIR193BHG | -0.341961608 | 1.33E-05 |
| RHEB | MIR193BHG | -0.370431823 | 2.09E-06 |
| RPS6KB1 | MIR193BHG | -0.328588326 | 2.99E-05 |
| SIRT1 | MIR193BHG | -0.36996431 | 2.16E-06 |
| TP53 | MIR193BHG | -0.476534929 | 3.66E-10 |
| ULK2 | MIR193BHG | -0.302114059 | 0.000133175 |
| UVRAG | MIR193BHG | -0.347944691 | 9.15E-06 |
| VAMP7 | MIR193BHG | -0.349792056 | 8.14E-06 |
| FKBP1B | AFAP1-AS1 | 0.315123658 | 6.50E-05 |
| FOXO1 | AFAP1-AS1 | -0.312272128 | 7.63E-05 |
| MYC | AFAP1-AS1 | 0.402073481 | 2.15E-07 |
| NAF1 | AFAP1-AS1 | 0.375506163 | 1.48E-06 |
| ATG3 | LINC02585 | 0.3355799 | 1.97E-05 |
| ATG4B | LINC02585 | 0.344994764 | 1.10E-05 |
| ATIC | LINC02585 | 0.366835241 | 2.67E-06 |
| BAG1 | LINC02585 | 0.310322193 | 8.51E-05 |
| BAX | LINC02585 | 0.4175068 | 6.49E-08 |
| BIRC5 | LINC02585 | 0.388653047 | 5.81E-07 |
| CAPN10 | LINC02585 | 0.472272731 | 5.51E-10 |
| CLN3 | LINC02585 | 0.323877097 | 3.94E-05 |
| FKBP1A | LINC02585 | 0.30472467 | 0.000115645 |
| HDAC6 | LINC02585 | 0.310006648 | 8.66E-05 |
| HIF1A | LINC02585 | -0.309605179 | 8.85E-05 |
| ITGA3 | LINC02585 | -0.347779507 | 9.25E-06 |
| MAPK8IP1 | LINC02585 | 0.427698794 | 2.84E-08 |
| MLST8 | LINC02585 | 0.402357531 | 2.11E-07 |
| MTMR14 | LINC02585 | 0.357430408 | 4.98E-06 |
| PINK1 | LINC02585 | -0.372173778 | 1.86E-06 |
| STK11 | LINC02585 | 0.301918987 | 0.00013458 |
| ULK3 | LINC02585 | 0.358005625 | 4.79E-06 |
| WDR45 | LINC02585 | 0.389716764 | 5.38E-07 |
| ATG3 | AC079414.3 | 0.316735548 | 5.94E-05 |
| ATG4B | AC079414.3 | 0.47692996 | 3.53E-10 |
| ATIC | AC079414.3 | 0.338335422 | 1.66E-05 |
| BAX | AC079414.3 | 0.491481875 | 8.38E-11 |
| BIRC5 | AC079414.3 | 0.426546471 | 3.13E-08 |
| BNIP1 | AC079414.3 | 0.350419921 | 7.82E-06 |
| CAPN10 | AC079414.3 | 0.479074858 | 2.86E-10 |
| CDKN1B | AC079414.3 | 0.405361026 | 1.67E-07 |
| CDKN2A | AC079414.3 | 0.363076869 | 3.43E-06 |
| CLN3 | AC079414.3 | 0.319879383 | 4.96E-05 |
| CXCR4 | AC079414.3 | 0.424994152 | 3.55E-08 |
| DAPK2 | AC079414.3 | 0.348893861 | 8.62E-06 |
| GABARAPL2 | AC079414.3 | 0.318444993 | 5.39E-05 |
| HDAC6 | AC079414.3 | 0.47540734 | 4.08E-10 |
| HIF1A | AC079414.3 | -0.341396079 | 1.38E-05 |
| ITGA3 | AC079414.3 | -0.35599778 | 5.46E-06 |
| ITGA6 | AC079414.3 | -0.337468759 | 1.75E-05 |
| MAP2K7 | AC079414.3 | 0.386486386 | 6.79E-07 |
| MAPK8IP1 | AC079414.3 | 0.393902937 | 3.96E-07 |
| MLST8 | AC079414.3 | 0.353770197 | 6.31E-06 |
| MTMR14 | AC079414.3 | 0.326521829 | 3.38E-05 |
| NCKAP1 | AC079414.3 | -0.335159774 | 2.02E-05 |
| PEX14 | AC079414.3 | 0.329028713 | 2.91E-05 |
| PINK1 | AC079414.3 | -0.342406244 | 1.29E-05 |
| PRKAB1 | AC079414.3 | 0.333314701 | 2.26E-05 |
| STK11 | AC079414.3 | 0.428484552 | 2.67E-08 |
| TP53 | AC079414.3 | 0.339155771 | 1.58E-05 |
| TSC2 | AC079414.3 | 0.300739856 | 0.000143371 |
| ULK3 | AC079414.3 | 0.315909347 | 6.22E-05 |
| WDFY3 | AC079414.3 | -0.304988294 | 0.000114 |
| WDR45 | AC079414.3 | 0.403113956 | 1.99E-07 |
| HDAC1 | AL133338.1 | 0.344684603 | 1.12E-05 |
| HDAC6 | AL133338.1 | 0.39852322 | 2.81E-07 |
| MAPK8 | AL133338.1 | 0.318152799 | 5.48E-05 |
| RAB33B | AL133338.1 | 0.301997582 | 0.000134013 |
| RB1CC1 | AL133338.1 | 0.338563694 | 1.64E-05 |
| ATIC | AC004540.2 | 0.621734399 | 5.97E-18 |
| BIRC5 | AC004540.2 | 0.452591632 | 3.37E-09 |
| DIRAS3 | AC004540.2 | 0.385138868 | 7.48E-07 |
| GABARAPL2 | AC004540.2 | 0.338437974 | 1.65E-05 |
| MAPK8IP1 | AC004540.2 | 0.931923056 | 2.77E-69 |
| PRKAR1A | AC004540.2 | 0.636472892 | 5.57E-19 |
| PRKCQ | AC004540.2 | 0.305897395 | 0.000108493 |
| VAMP7 | AC004540.2 | 0.424888341 | 3.58E-08 |
| ARNT | AC108673.3 | -0.346004528 | 1.03E-05 |
| ATF4 | AC108673.3 | 0.431297457 | 2.11E-08 |
| ATF6 | AC108673.3 | -0.321368683 | 4.56E-05 |
| ATG16L2 | AC108673.3 | 0.417562937 | 6.46E-08 |
| ATG2B | AC108673.3 | -0.393809891 | 3.99E-07 |
| ATG3 | AC108673.3 | 0.415409392 | 7.67E-08 |
| ATG4B | AC108673.3 | 0.637353792 | 4.82E-19 |
| BAG1 | AC108673.3 | 0.352280506 | 6.95E-06 |
| BAG3 | AC108673.3 | -0.356504503 | 5.29E-06 |
| BAX | AC108673.3 | 0.553014156 | 8.56E-14 |
| BIRC5 | AC108673.3 | 0.420243863 | 5.21E-08 |
| BIRC6 | AC108673.3 | -0.441023713 | 9.28E-09 |
| BNIP1 | AC108673.3 | 0.55533675 | 6.42E-14 |
| CANX | AC108673.3 | -0.373397587 | 1.71E-06 |
| CAPN10 | AC108673.3 | 0.577638619 | 3.58E-15 |
| CAPN2 | AC108673.3 | -0.326482239 | 3.38E-05 |
| CDKN2A | AC108673.3 | 0.392884713 | 4.27E-07 |
| CLN3 | AC108673.3 | 0.332126648 | 2.42E-05 |
| CXCR4 | AC108673.3 | 0.351290643 | 7.40E-06 |
| DAPK2 | AC108673.3 | 0.423025994 | 4.17E-08 |
| DLC1 | AC108673.3 | -0.316121053 | 6.15E-05 |
| EIF4G1 | AC108673.3 | -0.37377693 | 1.66E-06 |
| FKBP1A | AC108673.3 | 0.350243327 | 7.91E-06 |
| GOPC | AC108673.3 | -0.336285531 | 1.88E-05 |
| ITGA6 | AC108673.3 | -0.344813071 | 1.11E-05 |
| ITGB1 | AC108673.3 | -0.311631762 | 7.91E-05 |
| KIF5B | AC108673.3 | -0.403717208 | 1.90E-07 |
| LAMP2 | AC108673.3 | -0.395654696 | 3.48E-07 |
| MAP1LC3A | AC108673.3 | 0.328542518 | 3.00E-05 |
| MAP2K7 | AC108673.3 | 0.303740098 | 0.000121986 |
| MAPK1 | AC108673.3 | -0.418242654 | 6.12E-08 |
| MBTPS2 | AC108673.3 | -0.313858303 | 6.98E-05 |
| MLST8 | AC108673.3 | 0.346637772 | 9.94E-06 |
| MTMR14 | AC108673.3 | 0.342995821 | 1.25E-05 |
| NCKAP1 | AC108673.3 | -0.400178493 | 2.48E-07 |
| NPC1 | AC108673.3 | -0.327341461 | 3.22E-05 |
| PEX14 | AC108673.3 | 0.396850148 | 3.18E-07 |
| PINK1 | AC108673.3 | -0.335848025 | 1.94E-05 |
| RAB24 | AC108673.3 | 0.447084182 | 5.48E-09 |
| RB1 | AC108673.3 | -0.323366239 | 4.06E-05 |
| STK11 | AC108673.3 | 0.499507853 | 3.68E-11 |
| TP63 | AC108673.3 | -0.338429635 | 1.65E-05 |
| ULK3 | AC108673.3 | 0.33587839 | 1.93E-05 |
| USP10 | AC108673.3 | -0.376838922 | 1.34E-06 |
| WDFY3 | AC108673.3 | -0.431562894 | 2.07E-08 |
| WDR45 | AC108673.3 | 0.521467656 | 3.47E-12 |
| ATF6 | LOXL1-AS1 | 0.327075922 | 3.27E-05 |
| BAK1 | LOXL1-AS1 | -0.337468419 | 1.75E-05 |
| CASP4 | LOXL1-AS1 | -0.378756892 | 1.18E-06 |
| EIF2AK3 | LOXL1-AS1 | 0.319258144 | 5.14E-05 |
| FOXO1 | LOXL1-AS1 | 0.343069532 | 1.24E-05 |
| GABARAPL1 | LOXL1-AS1 | 0.371395192 | 1.96E-06 |
| KLHL24 | LOXL1-AS1 | 0.369732909 | 2.19E-06 |
| MBTPS2 | LOXL1-AS1 | 0.342437229 | 1.29E-05 |
| NFE2L2 | LOXL1-AS1 | 0.308156543 | 9.59E-05 |
| PIK3C3 | LOXL1-AS1 | 0.313471647 | 7.14E-05 |
| PTK6 | LOXL1-AS1 | -0.316811352 | 5.91E-05 |
| RHEB | LOXL1-AS1 | 0.357727725 | 4.88E-06 |
| SESN2 | LOXL1-AS1 | -0.333679201 | 2.21E-05 |
| ST13 | LOXL1-AS1 | 0.401036086 | 2.33E-07 |
| TP63 | LOXL1-AS1 | 0.386388442 | 6.84E-07 |
| UVRAG | LOXL1-AS1 | 0.371049082 | 2.01E-06 |
| WIPI1 | LOXL1-AS1 | 0.389653179 | 5.40E-07 |
| ATG16L2 | TRPM2-AS | 0.36500685 | 3.02E-06 |
| BID | TRPM2-AS | 0.465054264 | 1.08E-09 |
| CLN3 | TRPM2-AS | 0.305789696 | 0.000109132 |
| GNAI3 | TRPM2-AS | -0.327761482 | 3.14E-05 |
| MAP1LC3A | TRPM2-AS | 0.352713098 | 6.76E-06 |
| MLST8 | TRPM2-AS | 0.303132207 | 0.000126063 |
| PELP1 | TRPM2-AS | 0.342381798 | 1.30E-05 |
| ATG12 | PRKCZ-AS1 | 0.330518684 | 2.67E-05 |
| ATG16L2 | PRKCZ-AS1 | 0.379174577 | 1.14E-06 |
| ATG4C | PRKCZ-AS1 | 0.311479009 | 7.98E-05 |
| CAPN10 | PRKCZ-AS1 | 0.341808934 | 1.34E-05 |
| CFLAR | PRKCZ-AS1 | 0.30327682 | 0.000125081 |
| HDAC1 | PRKCZ-AS1 | 0.375058389 | 1.52E-06 |
| HDAC6 | PRKCZ-AS1 | 0.347716671 | 9.29E-06 |
| IKBKB | PRKCZ-AS1 | 0.300942221 | 0.000141825 |
| MAPK8 | PRKCZ-AS1 | 0.34076386 | 1.43E-05 |
| MTOR | PRKCZ-AS1 | 0.346710593 | 9.90E-06 |
| PEX14 | PRKCZ-AS1 | 0.366082962 | 2.81E-06 |
| RAF1 | PRKCZ-AS1 | 0.340817484 | 1.43E-05 |
| STK11 | PRKCZ-AS1 | 0.314670389 | 6.67E-05 |
| TP73 | PRKCZ-AS1 | 0.366701559 | 2.69E-06 |
| TSC1 | PRKCZ-AS1 | 0.343892771 | 1.18E-05 |
| TSC2 | PRKCZ-AS1 | 0.350474758 | 7.80E-06 |
| ULK3 | PRKCZ-AS1 | 0.361117682 | 3.91E-06 |
| VAMP3 | PRKCZ-AS1 | 0.3402881 | 1.48E-05 |
| ITGB4 | AC023906.5 | 0.327064058 | 3.27E-05 |
| BNIP3 | AL078590.2 | 0.361299454 | 3.86E-06 |
| EIF2S1 | AL078590.2 | 0.493988709 | 6.50E-11 |
| FADD | AL078590.2 | 0.343705581 | 1.19E-05 |
| HIF1A | AL078590.2 | 0.535310585 | 7.17E-13 |
| NPC1 | AL078590.2 | 0.369839223 | 2.18E-06 |
| PEX3 | AL078590.2 | 0.647910149 | 8.09E-20 |
| ZFYVE1 | AL078590.2 | 0.381216774 | 9.89E-07 |
| ATF6 | LINC02542 | 0.307913754 | 9.72E-05 |
| EEF2K | LINC02542 | 0.326869071 | 3.31E-05 |
| ATG16L2 | AL121820.2 | 0.436090904 | 1.41E-08 |
| ATG9B | AL121820.2 | 0.31471046 | 6.66E-05 |
| BID | AL121820.2 | 0.43944752 | 1.06E-08 |
| FOXO1 | AL121820.2 | -0.318546497 | 5.36E-05 |
| PRKAB1 | AL121820.2 | 0.333404852 | 2.24E-05 |
| APOL1 | AL596244.1 | 0.372465385 | 1.82E-06 |
| ATG5 | AL596244.1 | 0.307052269 | 0.000101855 |
| BAK1 | AL596244.1 | 0.314484572 | 6.74E-05 |
| CASP1 | AL596244.1 | 0.352319307 | 6.93E-06 |
| CASP4 | AL596244.1 | 0.464025974 | 1.19E-09 |
| IL24 | AL596244.1 | 0.338074784 | 1.69E-05 |
| ITGA3 | AL596244.1 | 0.652278127 | 3.79E-20 |
| ITGA6 | AL596244.1 | 0.4956789 | 5.47E-11 |
| ITGB4 | AL596244.1 | 0.398360402 | 2.84E-07 |
| PINK1 | AL596244.1 | 0.398300503 | 2.86E-07 |
| SAR1A | AL596244.1 | 0.323558987 | 4.01E-05 |
| ERN1 | AC068580.1 | 0.325964049 | 3.49E-05 |
| HGS | AC068580.1 | 0.343387162 | 1.22E-05 |
| NPC1 | AC068580.1 | 0.32808434 | 3.08E-05 |
| SPNS1 | AC068580.1 | 0.475423062 | 4.08E-10 |
| VEGFA | AC068580.1 | 0.306797635 | 0.000103285 |
| ATG16L2 | AC145207.5 | 0.33906709 | 1.59E-05 |
| ATG4B | AC145207.5 | 0.421301315 | 4.79E-08 |
| ATG4C | AC145207.5 | 0.401234937 | 2.29E-07 |
| BAX | AC145207.5 | 0.308058581 | 9.64E-05 |
| BIRC5 | AC145207.5 | 0.327648151 | 3.16E-05 |
| CAPN10 | AC145207.5 | 0.518088522 | 5.05E-12 |
| CDKN1B | AC145207.5 | 0.332974122 | 2.30E-05 |
| CXCR4 | AC145207.5 | 0.450636762 | 4.01E-09 |
| DAPK2 | AC145207.5 | 0.338844375 | 1.61E-05 |
| HDAC6 | AC145207.5 | 0.356321437 | 5.35E-06 |
| IKBKB | AC145207.5 | 0.325490711 | 3.59E-05 |
| MAP2K7 | AC145207.5 | 0.379792207 | 1.09E-06 |
| MAPK8 | AC145207.5 | 0.309870531 | 8.72E-05 |
| MTMR14 | AC145207.5 | 0.334815381 | 2.06E-05 |
| RAF1 | AC145207.5 | 0.35628064 | 5.37E-06 |
| STK11 | AC145207.5 | 0.422396325 | 4.38E-08 |
| TP53 | AC145207.5 | 0.343451866 | 1.21E-05 |
| TSC2 | AC145207.5 | 0.419273656 | 5.64E-08 |
| ULK3 | AC145207.5 | 0.371739825 | 1.91E-06 |
| DIRAS3 | LINC00239 | 0.353753748 | 6.32E-06 |
| RAB24 | LINC00239 | 0.325646625 | 3.55E-05 |
| ATG10 | AL391834.2 | 0.305260285 | 0.000112325 |
| ATG4C | AL391834.2 | 0.39836128 | 2.84E-07 |
| BAX | AL391834.2 | 0.350455534 | 7.81E-06 |
| CDKN1B | AL391834.2 | 0.37167305 | 1.92E-06 |
| CXCR4 | AL391834.2 | 0.374338156 | 1.60E-06 |
| DRAM1 | AL391834.2 | 0.396877148 | 3.18E-07 |
| EIF4G1 | AL391834.2 | -0.367443627 | 2.56E-06 |
| GABARAP | AL391834.2 | 0.411126835 | 1.07E-07 |
| HIF1A | AL391834.2 | -0.319178429 | 5.17E-05 |
| MAPK9 | AL391834.2 | 0.321337364 | 4.56E-05 |
| MTMR14 | AL391834.2 | 0.339745369 | 1.53E-05 |
| NKX2-3 | AL391834.2 | 0.380453486 | 1.04E-06 |
| PTK6 | AL391834.2 | -0.319875917 | 4.96E-05 |
| TP53 | AL391834.2 | 0.322750905 | 4.21E-05 |
| TP73 | AL391834.2 | 0.302790359 | 0.00012841 |
| TSC2 | AL391834.2 | 0.309653194 | 8.83E-05 |
| TUSC1 | AL391834.2 | 0.486533201 | 1.38E-10 |
| ULK2 | AL391834.2 | 0.355560913 | 5.62E-06 |
| WDR45 | AL391834.2 | 0.322464644 | 4.28E-05 |
| BIRC5 | AC112491.1 | 0.307109111 | 0.000101539 |
| BNIP1 | AC112491.1 | 0.378070774 | 1.23E-06 |
| EIF4EBP1 | AC112491.1 | 0.305199391 | 0.000112698 |
| FKBP1A | AC112491.1 | 0.324447132 | 3.81E-05 |
| HGS | AC112491.1 | -0.302015624 | 0.000133883 |
| ARNT | SNHG7 | -0.302271449 | 0.000132052 |
| ATF4 | SNHG7 | 0.466573032 | 9.42E-10 |
| ATG4B | SNHG7 | 0.31307359 | 7.30E-05 |
| ATIC | SNHG7 | 0.303981209 | 0.000120404 |
| BAG1 | SNHG7 | 0.370101205 | 2.14E-06 |
| BIRC6 | SNHG7 | -0.311484018 | 7.97E-05 |
| BNIP1 | SNHG7 | 0.32509813 | 3.67E-05 |
| CAPN1 | SNHG7 | -0.301853371 | 0.000135056 |
| CAPN10 | SNHG7 | 0.380305257 | 1.05E-06 |
| CAPN2 | SNHG7 | -0.349554369 | 8.27E-06 |
| LAMP2 | SNHG7 | -0.432438779 | 1.92E-08 |
| MAPK1 | SNHG7 | -0.43331681 | 1.78E-08 |
| MTOR | SNHG7 | -0.325953668 | 3.49E-05 |
| SH3GLB1 | SNHG7 | -0.301652398 | 0.000136523 |
| WDFY3 | SNHG7 | -0.336053396 | 1.91E-05 |
| GABARAP | TPRG1-AS1 | -0.317577852 | 5.66E-05 |
| HSPA8 | TPRG1-AS1 | 0.30283202 | 0.000128122 |
| ATG16L2 | THAP9-AS1 | 0.355388802 | 5.69E-06 |
| ATG3 | THAP9-AS1 | 0.39632216 | 3.31E-07 |
| ATG4B | THAP9-AS1 | 0.337331156 | 1.77E-05 |
| ATG4D | THAP9-AS1 | 0.378420061 | 1.20E-06 |
| CAPN10 | THAP9-AS1 | 0.404152753 | 1.84E-07 |
| CD46 | THAP9-AS1 | 0.303752556 | 0.000121904 |
| CDKN2A | THAP9-AS1 | 0.43300383 | 1.83E-08 |
| DAPK2 | THAP9-AS1 | 0.378283353 | 1.22E-06 |
| HDAC6 | THAP9-AS1 | 0.328914174 | 2.93E-05 |
| MAP2K7 | THAP9-AS1 | 0.358944296 | 4.51E-06 |
| MAPK8 | THAP9-AS1 | 0.339633013 | 1.54E-05 |
| PEX14 | THAP9-AS1 | 0.337035268 | 1.80E-05 |
| PINK1 | THAP9-AS1 | -0.347845217 | 9.21E-06 |
| RAB33B | THAP9-AS1 | 0.377284246 | 1.30E-06 |
| RAF1 | THAP9-AS1 | 0.350320687 | 7.87E-06 |
| STK11 | THAP9-AS1 | 0.401990114 | 2.16E-07 |
| ULK3 | THAP9-AS1 | 0.324315461 | 3.84E-05 |
| ATF6 | LINC01564 | 0.367865347 | 2.49E-06 |
| GABARAPL1 | LINC01564 | 0.498098428 | 4.26E-11 |
| NAMPT | LINC01564 | 0.388741799 | 5.77E-07 |
| NCKAP1 | LINC01564 | 0.345943851 | 1.04E-05 |
| NFE2L2 | LINC01564 | 0.444442377 | 6.91E-09 |
| PIK3C3 | LINC01564 | 0.32662046 | 3.36E-05 |
| RHEB | LINC01564 | 0.380332716 | 1.05E-06 |
| SQSTM1 | LINC01564 | 0.415078773 | 7.87E-08 |
| ST13 | LINC01564 | 0.307766054 | 9.79E-05 |
| TP63 | LINC01564 | 0.370889405 | 2.03E-06 |
| TP73 | LINC01564 | -0.304222752 | 0.000118838 |
| ATG9B | ACBD3-AS1 | 0.525385663 | 2.24E-12 |
| CFLAR | ACBD3-AS1 | 0.323767718 | 3.97E-05 |
| RAB11A | ACBD3-AS1 | 0.324809951 | 3.73E-05 |
| TP53INP2 | ACBD3-AS1 | 0.402239936 | 2.12E-07 |
| ATG9B | AL512363.1 | 0.653658892 | 2.97E-20 |
| HSPA5 | AL512363.1 | 0.302572834 | 0.000129925 |
| RAB1A | AL512363.1 | 0.307175519 | 0.00010117 |
| TP53INP2 | AL512363.1 | 0.501669269 | 2.94E-11 |
| BAK1 | LINC00165 | 0.41225494 | 9.82E-08 |
| CASP1 | LINC00165 | 0.632764253 | 1.02E-18 |
| CASP4 | LINC00165 | 0.475555121 | 4.02E-10 |
| HSPA8 | LINC00165 | 0.334798744 | 2.06E-05 |
| BAK1 | SERTAD4-AS1 | -0.306365699 | 0.000105754 |
| BID | SERTAD4-AS1 | 0.42818618 | 2.73E-08 |
| CTSB | MSC-AS1 | 0.41286859 | 9.36E-08 |
| NAMPT | MSC-AS1 | 0.422219423 | 4.45E-08 |
| CAPN10 | SCGB1B2P | 0.320159899 | 4.88E-05 |
| DIRAS3 | SCGB1B2P | 0.968518625 | 2.66E-94 |
| FKBP1B | SCGB1B2P | 0.42499605 | 3.55E-08 |
| MAP1LC3A | SCGB1B2P | 0.320804654 | 4.71E-05 |
| PRKCQ | SCGB1B2P | 0.35142914 | 7.34E-06 |
| ATG9B | ELFN1-AS1 | 0.345130485 | 1.09E-05 |
| FKBP1B | ELFN1-AS1 | 0.413774113 | 8.72E-08 |
| PRKCQ | ELFN1-AS1 | 0.328863394 | 2.94E-05 |
| ATF4 | Z83851.2 | 0.366322279 | 2.76E-06 |
| ATIC | Z83851.2 | 0.323952784 | 3.92E-05 |
| BIRC5 | Z83851.2 | 0.307580525 | 9.89E-05 |
| PTEN | Z83851.2 | -0.319176012 | 5.17E-05 |
| ARNT | LINC02560 | -0.385745258 | 7.17E-07 |
| ARSB | LINC02560 | -0.455332148 | 2.64E-09 |
| ATF6 | LINC02560 | -0.373942741 | 1.64E-06 |
| ATG16L2 | LINC02560 | 0.315488216 | 6.37E-05 |
| ATG9B | LINC02560 | 0.529116601 | 1.47E-12 |
| BIRC6 | LINC02560 | -0.30902649 | 9.14E-05 |
| CANX | LINC02560 | -0.388500996 | 5.87E-07 |
| CD46 | LINC02560 | -0.308224667 | 9.55E-05 |
| EEF2 | LINC02560 | -0.305284363 | 0.000112178 |
| FOXO1 | LINC02560 | -0.39425301 | 3.86E-07 |
| GABARAPL2 | LINC02560 | 0.323864928 | 3.94E-05 |
| GOPC | LINC02560 | -0.382802851 | 8.84E-07 |
| LAMP2 | LINC02560 | -0.384024575 | 8.10E-07 |
| MAPK1 | LINC02560 | -0.307437528 | 9.97E-05 |
| MAPK3 | LINC02560 | 0.441049799 | 9.26E-09 |
| MAPK9 | LINC02560 | -0.353435037 | 6.45E-06 |
| MTOR | LINC02560 | -0.303524712 | 0.000123416 |
| NBR1 | LINC02560 | -0.328223466 | 3.05E-05 |
| PARP1 | LINC02560 | -0.408635105 | 1.30E-07 |
| PTK6 | LINC02560 | 0.46094951 | 1.58E-09 |
| RAB11A | LINC02560 | 0.406936916 | 1.48E-07 |
| RAB24 | LINC02560 | 0.465839067 | 1.01E-09 |
| TM9SF1 | LINC02560 | -0.354292923 | 6.10E-06 |
| ATF4 | AC048341.2 | 0.327858482 | 3.12E-05 |
| ATG12 | AC048341.2 | 0.350405875 | 7.83E-06 |
| ATG16L2 | AC048341.2 | 0.46839596 | 7.94E-10 |
| ATG4B | AC048341.2 | 0.437344149 | 1.27E-08 |
| BAG3 | AC048341.2 | -0.334438127 | 2.11E-05 |
| BAX | AC048341.2 | 0.367976667 | 2.47E-06 |
| CAPN10 | AC048341.2 | 0.536133776 | 6.51E-13 |
| CXCR4 | AC048341.2 | 0.600780274 | 1.41E-16 |
| DAPK2 | AC048341.2 | 0.389358974 | 5.52E-07 |
| EIF4G1 | AC048341.2 | -0.362281747 | 3.62E-06 |
| FKBP1A | AC048341.2 | 0.301840907 | 0.000135146 |
| IKBKB | AC048341.2 | 0.385859558 | 7.11E-07 |
| MTMR14 | AC048341.2 | 0.330646124 | 2.65E-05 |
| RAB24 | AC048341.2 | 0.363302071 | 3.38E-06 |
| TSC2 | AC048341.2 | 0.42482166 | 3.60E-08 |
| ULK3 | AC048341.2 | 0.388874257 | 5.72E-07 |
| WDFY3 | AC048341.2 | -0.300744159 | 0.000143338 |
| WDR45 | AC048341.2 | 0.440062954 | 1.01E-08 |
| AMBRA1 | PRR34-AS1 | -0.363794322 | 3.27E-06 |
| ARNT | PRR34-AS1 | -0.422029234 | 4.52E-08 |
| ARSB | PRR34-AS1 | -0.302330778 | 0.000131631 |
| ATF4 | PRR34-AS1 | 0.496751316 | 4.90E-11 |
| ATF6 | PRR34-AS1 | -0.319069194 | 5.20E-05 |
| ATG2A | PRR34-AS1 | -0.315923673 | 6.22E-05 |
| ATG2B | PRR34-AS1 | -0.34940879 | 8.34E-06 |
| ATG4B | PRR34-AS1 | 0.307275897 | 0.000100615 |
| BAG1 | PRR34-AS1 | 0.302500423 | 0.000130433 |
| BAX | PRR34-AS1 | 0.423748621 | 3.93E-08 |
| BECN1 | PRR34-AS1 | -0.322940112 | 4.16E-05 |
| BIRC5 | PRR34-AS1 | 0.371564452 | 1.94E-06 |
| BIRC6 | PRR34-AS1 | -0.52513678 | 2.30E-12 |
| BNIP1 | PRR34-AS1 | 0.393187211 | 4.17E-07 |
| CANX | PRR34-AS1 | -0.433244536 | 1.79E-08 |
| CAPN2 | PRR34-AS1 | -0.336593322 | 1.85E-05 |
| EEF2K | PRR34-AS1 | -0.413071713 | 9.21E-08 |
| EIF2AK2 | PRR34-AS1 | -0.360557338 | 4.06E-06 |
| EIF2AK3 | PRR34-AS1 | -0.372623624 | 1.80E-06 |
| EIF4EBP1 | PRR34-AS1 | 0.308547389 | 9.38E-05 |
| EIF4G1 | PRR34-AS1 | -0.320555861 | 4.77E-05 |
| FKBP1A | PRR34-AS1 | 0.315163107 | 6.49E-05 |
| FOXO3 | PRR34-AS1 | -0.319044581 | 5.21E-05 |
| GOPC | PRR34-AS1 | -0.368981942 | 2.31E-06 |
| HGS | PRR34-AS1 | -0.392722371 | 4.32E-07 |
| KIF5B | PRR34-AS1 | -0.347481152 | 9.43E-06 |
| LAMP2 | PRR34-AS1 | -0.303084449 | 0.000126388 |
| MAPK1 | PRR34-AS1 | -0.401141809 | 2.31E-07 |
| MLST8 | PRR34-AS1 | 0.321140416 | 4.62E-05 |
| MTOR | PRR34-AS1 | -0.448720079 | 4.75E-09 |
| NAF1 | PRR34-AS1 | -0.360852572 | 3.98E-06 |
| NBR1 | PRR34-AS1 | -0.435339546 | 1.51E-08 |
| NCKAP1 | PRR34-AS1 | -0.389126451 | 5.61E-07 |
| NFKB1 | PRR34-AS1 | -0.341127914 | 1.40E-05 |
| PIK3R4 | PRR34-AS1 | -0.351681358 | 7.22E-06 |
| RAB24 | PRR34-AS1 | 0.306340508 | 0.0001059 |
| RB1 | PRR34-AS1 | -0.315212195 | 6.47E-05 |
| RB1CC1 | PRR34-AS1 | -0.304053378 | 0.000119934 |
| RELA | PRR34-AS1 | -0.301885979 | 0.000134819 |
| RPS6KB1 | PRR34-AS1 | -0.344758027 | 1.12E-05 |
| RPTOR | PRR34-AS1 | -0.334416184 | 2.11E-05 |
| SH3GLB1 | PRR34-AS1 | -0.305895163 | 0.000108506 |
| SIRT1 | PRR34-AS1 | -0.360801302 | 3.99E-06 |
| SPNS1 | PRR34-AS1 | -0.396910011 | 3.17E-07 |
| USP10 | PRR34-AS1 | -0.373745431 | 1.67E-06 |
| WDFY3 | PRR34-AS1 | -0.400124027 | 2.49E-07 |
| WDR45 | PRR34-AS1 | 0.399381515 | 2.63E-07 |
| ITGA3 | AC002470.1 | -0.334921885 | 2.05E-05 |
| NFKB1 | AC002470.1 | -0.318589135 | 5.34E-05 |
| ST13 | AC002470.1 | 0.427324789 | 2.93E-08 |
| WIPI1 | AC002470.1 | 0.314432354 | 6.76E-05 |
| ATG16L2 | DLGAP1-AS1 | 0.323013516 | 4.14E-05 |
| BID | DLGAP1-AS1 | 0.340605893 | 1.45E-05 |
| CAPN10 | DLGAP1-AS1 | 0.36269268 | 3.52E-06 |
| FOXO1 | DLGAP1-AS1 | -0.312541508 | 7.52E-05 |
| ULK3 | DLGAP1-AS1 | 0.382310949 | 9.15E-07 |
| AMBRA1 | AL445524.1 | -0.355302591 | 5.72E-06 |
| BIRC6 | AL445524.1 | -0.363342675 | 3.37E-06 |
| DDIT3 | AL445524.1 | 0.425908595 | 3.29E-08 |
| EIF2AK2 | AL445524.1 | -0.325594039 | 3.56E-05 |
| GOPC | AL445524.1 | -0.310200328 | 8.56E-05 |
| NBR1 | AL445524.1 | -0.310675187 | 8.34E-05 |
| USP10 | AL445524.1 | -0.314057834 | 6.91E-05 |
| MYC | AC006329.1 | 0.338949485 | 1.60E-05 |
| ATG7 | AC126768.2 | -0.33773308 | 1.73E-05 |
| MTMR14 | AC126768.2 | -0.32324351 | 4.09E-05 |
| SESN2 | AC126768.2 | -0.32673364 | 3.33E-05 |
| RHEB | AC113383.1 | 0.308947192 | 9.18E-05 |
| TP63 | AC113383.1 | 0.316555401 | 6.00E-05 |
| ATG3 | AL645933.2 | 0.340491632 | 1.46E-05 |
| CAPN10 | AL645933.2 | 0.31642768 | 6.04E-05 |
| CDKN2A | AL645933.2 | 0.303812873 | 0.000121507 |
| MAP2K7 | AL645933.2 | 0.411482706 | 1.04E-07 |
| STK11 | AL645933.2 | 0.393842991 | 3.98E-07 |
| ULK3 | AL645933.2 | 0.365494813 | 2.92E-06 |
| ATG4D | AC007114.1 | 0.33668685 | 1.84E-05 |
| BAK1 | AC007114.1 | -0.301487836 | 0.000137734 |
| BNIP1 | AC007114.1 | 0.303573708 | 0.00012309 |
| CDKN1B | AC007114.1 | 0.342102571 | 1.32E-05 |
| CDKN2A | AC007114.1 | 0.312602682 | 7.49E-05 |
| FKBP1A | AC007114.1 | 0.335953297 | 1.92E-05 |
| GABARAP | AC007114.1 | 0.358653934 | 4.60E-06 |
| HDAC6 | AC007114.1 | 0.344173756 | 1.16E-05 |
| IKBKE | AC007114.1 | 0.315600744 | 6.33E-05 |
| MTMR14 | AC007114.1 | 0.342787446 | 1.26E-05 |
| PTK6 | AC007114.1 | -0.371549929 | 1.94E-06 |
| WDR45 | AC007114.1 | 0.38029174 | 1.06E-06 |
| ATG16L2 | SLC9A3-AS1 | 0.374766985 | 1.55E-06 |
| CAPN10 | SLC9A3-AS1 | 0.322890118 | 4.17E-05 |
| DIRAS3 | SLC9A3-AS1 | 0.357412734 | 4.98E-06 |
| FKBP1B | SLC9A3-AS1 | 0.346204175 | 1.02E-05 |
| FOXO1 | SLC9A3-AS1 | -0.321492613 | 4.52E-05 |
| GABARAPL2 | SLC9A3-AS1 | 0.307390751 | 1.00E-04 |
| ATIC | FOXD2-AS1 | 0.362009334 | 3.68E-06 |
| BIRC5 | FOXD2-AS1 | 0.395825583 | 3.43E-07 |
| MAPK8IP1 | FOXD2-AS1 | 0.556473348 | 5.57E-14 |
| PRKAR1A | FOXD2-AS1 | 0.396028069 | 3.38E-07 |
| VAMP7 | FOXD2-AS1 | 0.352585995 | 6.81E-06 |
| NFE2L2 | AC004990.1 | 0.521202377 | 3.58E-12 |
| TP63 | AC004990.1 | 0.358655416 | 4.60E-06 |
| WIPI1 | AC004990.1 | 0.390024252 | 5.26E-07 |
| AMBRA1 | AC139795.2 | 0.321257178 | 4.59E-05 |
| ARNT | AC139795.2 | 0.363787264 | 3.27E-06 |
| ARSB | AC139795.2 | 0.396173925 | 3.35E-07 |
| ATG12 | AC139795.2 | 0.351877873 | 7.13E-06 |
| ATG2A | AC139795.2 | 0.304769799 | 0.000115361 |
| ATG4C | AC139795.2 | 0.36224118 | 3.63E-06 |
| ATG4D | AC139795.2 | 0.358697513 | 4.58E-06 |
| BCL2 | AC139795.2 | 0.500290219 | 3.40E-11 |
| CAMKK2 | AC139795.2 | 0.305290021 | 0.000112144 |
| CD46 | AC139795.2 | 0.444439049 | 6.91E-09 |
| CDKN1A | AC139795.2 | -0.339099639 | 1.59E-05 |
| CDKN1B | AC139795.2 | 0.432587031 | 1.90E-08 |
| CX3CL1 | AC139795.2 | 0.363327827 | 3.37E-06 |
| CXCR4 | AC139795.2 | 0.490040509 | 9.69E-11 |
| DRAM1 | AC139795.2 | 0.380182633 | 1.06E-06 |
| EEF2 | AC139795.2 | 0.347772603 | 9.25E-06 |
| EEF2K | AC139795.2 | 0.524884955 | 2.37E-12 |
| EIF2AK3 | AC139795.2 | 0.35182724 | 7.15E-06 |
| FAS | AC139795.2 | 0.458740556 | 1.94E-09 |
| HDAC6 | AC139795.2 | 0.500381628 | 3.36E-11 |
| IKBKB | AC139795.2 | 0.376571547 | 1.37E-06 |
| IKBKE | AC139795.2 | 0.350199056 | 7.93E-06 |
| KLHL24 | AC139795.2 | 0.425050815 | 3.53E-08 |
| MAP2K7 | AC139795.2 | 0.457604772 | 2.15E-09 |
| MAPK8 | AC139795.2 | 0.462810459 | 1.33E-09 |
| MAPK9 | AC139795.2 | 0.425311727 | 3.46E-08 |
| MTOR | AC139795.2 | 0.371655886 | 1.92E-06 |
| NBR1 | AC139795.2 | 0.378011819 | 1.24E-06 |
| NKX2-3 | AC139795.2 | 0.56495182 | 1.90E-14 |
| PARP1 | AC139795.2 | 0.433155111 | 1.81E-08 |
| PIK3C3 | AC139795.2 | 0.40077535 | 2.37E-07 |
| PIK3R4 | AC139795.2 | 0.425477045 | 3.41E-08 |
| PRKAB1 | AC139795.2 | 0.403017252 | 2.00E-07 |
| PTK6 | AC139795.2 | -0.415866083 | 7.39E-08 |
| RAF1 | AC139795.2 | 0.373793776 | 1.66E-06 |
| SIRT1 | AC139795.2 | 0.363381592 | 3.36E-06 |
| SQSTM1 | AC139795.2 | 0.364897944 | 3.04E-06 |
| STK11 | AC139795.2 | 0.314792795 | 6.63E-05 |
| TP53 | AC139795.2 | 0.504983064 | 2.08E-11 |
| TP73 | AC139795.2 | 0.528146694 | 1.64E-12 |
| TSC1 | AC139795.2 | 0.41611164 | 7.25E-08 |
| TSC2 | AC139795.2 | 0.570764439 | 8.92E-15 |
| ULK1 | AC139795.2 | 0.35726734 | 5.03E-06 |
| ULK2 | AC139795.2 | 0.300858318 | 0.000142464 |
| UVRAG | AC139795.2 | 0.431555745 | 2.07E-08 |
| ATG2A | AC010343.3 | -0.306363601 | 0.000105766 |
| CAPNS1 | AC010343.3 | -0.30474058 | 0.000115545 |
| MTMR14 | AC010343.3 | -0.329637013 | 2.81E-05 |
| BAX | CEBPA-DT | 0.37155806 | 1.94E-06 |
| ITGA3 | CEBPA-DT | -0.31530863 | 6.44E-05 |
| ATF4 | KMT2E-AS1 | 0.3580073 | 4.79E-06 |
| ATG16L2 | KMT2E-AS1 | 0.40446209 | 1.79E-07 |
| ATG3 | KMT2E-AS1 | 0.320348609 | 4.83E-05 |
| ATG4B | KMT2E-AS1 | 0.444700049 | 6.75E-09 |
| ATG9B | KMT2E-AS1 | 0.393996403 | 3.93E-07 |
| BAX | KMT2E-AS1 | 0.448553164 | 4.82E-09 |
| BIRC5 | KMT2E-AS1 | 0.374646769 | 1.57E-06 |
| BNIP1 | KMT2E-AS1 | 0.335348446 | 2.00E-05 |
| CANX | KMT2E-AS1 | -0.407424443 | 1.43E-07 |
| CAPN10 | KMT2E-AS1 | 0.451481604 | 3.72E-09 |
| CLN3 | KMT2E-AS1 | 0.343190199 | 1.23E-05 |
| DDIT3 | KMT2E-AS1 | 0.340864647 | 1.42E-05 |
| DLC1 | KMT2E-AS1 | -0.337902235 | 1.71E-05 |
| FKBP1A | KMT2E-AS1 | 0.307552793 | 9.91E-05 |
| FOXO1 | KMT2E-AS1 | -0.309839029 | 8.74E-05 |
| GABARAPL2 | KMT2E-AS1 | 0.402028442 | 2.16E-07 |
| GOPC | KMT2E-AS1 | -0.308898927 | 9.20E-05 |
| ITGA3 | KMT2E-AS1 | -0.375779893 | 1.45E-06 |
| ITGA6 | KMT2E-AS1 | -0.333873976 | 2.18E-05 |
| ITGB1 | KMT2E-AS1 | -0.375671831 | 1.46E-06 |
| KIF5B | KMT2E-AS1 | -0.321381196 | 4.55E-05 |
| LAMP2 | KMT2E-AS1 | -0.355194904 | 5.76E-06 |
| MAPK1 | KMT2E-AS1 | -0.336613569 | 1.85E-05 |
| MLST8 | KMT2E-AS1 | 0.322579383 | 4.25E-05 |
| MTOR | KMT2E-AS1 | -0.327910278 | 3.11E-05 |
| PINK1 | KMT2E-AS1 | -0.352151907 | 7.00E-06 |
| RAB24 | KMT2E-AS1 | 0.360075689 | 4.19E-06 |
| TM9SF1 | KMT2E-AS1 | -0.342870875 | 1.26E-05 |
| WDFY3 | KMT2E-AS1 | -0.352452301 | 6.87E-06 |
| WDR45 | KMT2E-AS1 | 0.368601201 | 2.37E-06 |
| ZFYVE1 | KMT2E-AS1 | -0.313802139 | 7.01E-05 |
| DIRAS3 | AC090152.1 | 0.302860134 | 0.000127928 |
| ATG4D | AC083880.1 | 0.32977273 | 2.79E-05 |
| ITGA3 | AC083880.1 | -0.358602025 | 4.61E-06 |
| ITGA6 | AC083880.1 | -0.323685397 | 3.98E-05 |
| RB1 | AC083880.1 | -0.312795574 | 7.41E-05 |
| RHEB | AC083880.1 | 0.411815936 | 1.02E-07 |
| SH3GLB1 | AC083880.1 | -0.300084926 | 0.000148481 |
| VAMP3 | AC083880.1 | -0.321687805 | 4.47E-05 |
| WIPI1 | AC083880.1 | 0.381248315 | 9.87E-07 |
| CAPN1 | LINC02577 | 0.361404306 | 3.83E-06 |
| ITGA3 | LINC02577 | 0.426897369 | 3.04E-08 |
| NRG1 | LINC02577 | 0.403956543 | 1.86E-07 |
| RB1 | LINC02577 | 0.31770685 | 5.62E-05 |
| ULK3 | LINC02577 | -0.318037517 | 5.51E-05 |
| ARSB | AC087741.2 | -0.321684989 | 4.47E-05 |
| BIRC6 | AC087741.2 | -0.357781054 | 4.87E-06 |
| CAMKK2 | AC087741.2 | -0.333940899 | 2.17E-05 |
| CANX | AC087741.2 | -0.343251486 | 1.23E-05 |
| EIF4EBP1 | AC087741.2 | 0.354664464 | 5.96E-06 |
| GABARAPL2 | AC087741.2 | 0.362013327 | 3.68E-06 |
| KIF5B | AC087741.2 | -0.320908747 | 4.68E-05 |
| MAPK1 | AC087741.2 | -0.353312369 | 6.50E-06 |
| MTOR | AC087741.2 | -0.325103001 | 3.67E-05 |
| NFKB1 | AC087741.2 | -0.313757821 | 7.02E-05 |
| PIK3R4 | AC087741.2 | -0.313762574 | 7.02E-05 |
| ARSB | AL512274.1 | -0.338404769 | 1.66E-05 |
| ATG9B | AL512274.1 | 0.358285916 | 4.71E-06 |
| CAPN1 | AL512274.1 | 0.310285568 | 8.52E-05 |
| CHMP2B | AL512274.1 | 0.348662936 | 8.75E-06 |
| GOPC | AL512274.1 | -0.321865865 | 4.43E-05 |
| PTK6 | AL512274.1 | 0.384307816 | 7.94E-07 |
| RAB11A | AL512274.1 | 0.501210384 | 3.09E-11 |
| CASP4 | LINC01322 | 0.416529088 | 7.01E-08 |
| CD46 | LINC01322 | -0.327568696 | 3.17E-05 |
| CDKN1B | LINC01322 | -0.342230674 | 1.31E-05 |
| CX3CL1 | LINC01322 | -0.312999163 | 7.33E-05 |
| GABARAP | LINC01322 | -0.312645359 | 7.47E-05 |
| HSPA8 | LINC01322 | 0.34782073 | 9.23E-06 |
| ITGA3 | LINC01322 | 0.450321951 | 4.12E-09 |
| ITGA6 | LINC01322 | 0.344452775 | 1.14E-05 |
| ITGB4 | LINC01322 | 0.361523493 | 3.80E-06 |
| KLHL24 | LINC01322 | -0.316833905 | 5.90E-05 |
| NRG1 | LINC01322 | 0.449623612 | 4.39E-09 |
| PARP1 | LINC01322 | -0.303817967 | 0.000121473 |
| ATG4A | LINC00467 | 0.390655575 | 5.02E-07 |
| BNIP1 | LINC00467 | 0.307058265 | 0.000101822 |
| ARNT | AL450992.2 | 0.373394003 | 1.71E-06 |
| ATG4C | AL450992.2 | 0.328579521 | 2.99E-05 |
| CAMKK2 | AL450992.2 | 0.346556702 | 9.99E-06 |
| CDKN1B | AL450992.2 | 0.420805765 | 4.98E-08 |
| CXCR4 | AL450992.2 | 0.346814063 | 9.83E-06 |
| EDEM1 | AL450992.2 | 0.389804066 | 5.34E-07 |
| FAS | AL450992.2 | 0.359509168 | 4.34E-06 |
| ITPR1 | AL450992.2 | 0.316252959 | 6.10E-05 |
| MAPK8 | AL450992.2 | 0.318362287 | 5.41E-05 |
| NAF1 | AL450992.2 | 0.330384621 | 2.69E-05 |
| NKX2-3 | AL450992.2 | 0.309092939 | 9.10E-05 |
| PARP1 | AL450992.2 | 0.338254408 | 1.67E-05 |
| RAB33B | AL450992.2 | 0.388357785 | 5.94E-07 |
| RAF1 | AL450992.2 | 0.35765147 | 4.91E-06 |
| SIRT1 | AL450992.2 | 0.344382924 | 1.14E-05 |
| TP53 | AL450992.2 | 0.388788884 | 5.75E-07 |
| TP73 | AL450992.2 | 0.312196586 | 7.66E-05 |
| TSC1 | AL450992.2 | 0.325281583 | 3.63E-05 |
| TSC2 | AL450992.2 | 0.347030434 | 9.70E-06 |
| ARNT | NORAD | 0.317855659 | 5.57E-05 |
| ATG2B | NORAD | 0.311104133 | 8.15E-05 |
| BCL2L1 | NORAD | 0.403878073 | 1.88E-07 |
| BECN1 | NORAD | 0.386059983 | 7.01E-07 |
| BIRC6 | NORAD | 0.310468199 | 8.44E-05 |
| BNIP3 | NORAD | -0.301849228 | 0.000135086 |
| CAMKK2 | NORAD | 0.320061501 | 4.91E-05 |
| CD46 | NORAD | 0.326355121 | 3.41E-05 |
| CHMP4B | NORAD | 0.417057148 | 6.73E-08 |
| EIF2AK3 | NORAD | 0.381792031 | 9.50E-07 |
| HDAC6 | NORAD | 0.335584376 | 1.97E-05 |
| MAPK1 | NORAD | 0.356023501 | 5.46E-06 |
| NCKAP1 | NORAD | 0.353603754 | 6.38E-06 |
| NFE2L2 | NORAD | 0.331343266 | 2.54E-05 |
| NFKB1 | NORAD | 0.319048676 | 5.20E-05 |
| PIK3R4 | NORAD | 0.322621623 | 4.24E-05 |
| PRKAB1 | NORAD | 0.350498555 | 7.78E-06 |
| RAB33B | NORAD | 0.42305943 | 4.15E-08 |
| RAF1 | NORAD | 0.364669011 | 3.09E-06 |
| RB1CC1 | NORAD | 0.343986463 | 1.17E-05 |
| SIRT1 | NORAD | 0.390718886 | 5.00E-07 |
| TP53INP2 | NORAD | 0.348148712 | 9.04E-06 |
| WDFY3 | NORAD | 0.307117814 | 0.00010149 |
| ATG12 | AP006621.2 | 0.304571321 | 0.000116612 |
| ATG16L2 | AP006621.2 | 0.381052513 | 1.00E-06 |
| ATG4C | AP006621.2 | 0.344637315 | 1.13E-05 |
| CAPN10 | AP006621.2 | 0.401395794 | 2.26E-07 |
| CXCR4 | AP006621.2 | 0.343258224 | 1.23E-05 |
| HDAC6 | AP006621.2 | 0.471405593 | 5.98E-10 |
| IKBKB | AP006621.2 | 0.361578891 | 3.79E-06 |
| MAP2K7 | AP006621.2 | 0.302599425 | 0.000129739 |
| STK11 | AP006621.2 | 0.328679955 | 2.97E-05 |
| TSC2 | AP006621.2 | 0.341797628 | 1.34E-05 |
| ULK3 | AP006621.2 | 0.412053743 | 9.98E-08 |
| BID | AC128709.1 | 0.300948513 | 0.000141777 |
| NFE2L2 | AC128709.1 | 0.352310475 | 6.93E-06 |
| TP53INP2 | AC128709.1 | 0.330063407 | 2.74E-05 |
| EIF2AK3 | AL139158.2 | -0.350141898 | 7.96E-06 |
| GABARAPL1 | AL139158.2 | -0.316565752 | 5.99E-05 |
| PINK1 | AL139158.2 | 0.382472718 | 9.05E-07 |
| SIRT2 | AL139158.2 | 0.333472746 | 2.23E-05 |
| VAMP3 | AL139158.2 | 0.461454677 | 1.51E-09 |
| ATG10 | LINC00847 | 0.316345656 | 6.07E-05 |
| ATG3 | LINC00847 | 0.364086043 | 3.21E-06 |
| ATG4C | LINC00847 | 0.330318337 | 2.70E-05 |
| ATG4D | LINC00847 | 0.450743648 | 3.97E-09 |
| BCL2 | LINC00847 | 0.318843544 | 5.27E-05 |
| BNIP1 | LINC00847 | 0.363981937 | 3.23E-06 |
| CALCOCO2 | LINC00847 | 0.338822314 | 1.61E-05 |
| CAPN10 | LINC00847 | 0.375200571 | 1.51E-06 |
| CD46 | LINC00847 | 0.317906725 | 5.56E-05 |
| CDKN1B | LINC00847 | 0.391755961 | 4.64E-07 |
| CLN3 | LINC00847 | 0.351471558 | 7.32E-06 |
| CXCR4 | LINC00847 | 0.39099864 | 4.90E-07 |
| EEF2 | LINC00847 | 0.351724626 | 7.20E-06 |
| GABARAP | LINC00847 | 0.428469643 | 2.67E-08 |
| GABARAPL1 | LINC00847 | 0.302519268 | 0.000130301 |
| HDAC6 | LINC00847 | 0.478007747 | 3.18E-10 |
| IFNG | LINC00847 | 0.324781792 | 3.74E-05 |
| IKBKB | LINC00847 | 0.355758946 | 5.55E-06 |
| IKBKE | LINC00847 | 0.31079548 | 8.29E-05 |
| ITGA3 | LINC00847 | -0.314421922 | 6.77E-05 |
| ITGA6 | LINC00847 | -0.374005517 | 1.64E-06 |
| ITGB4 | LINC00847 | -0.340514894 | 1.46E-05 |
| ITPR1 | LINC00847 | 0.314357577 | 6.79E-05 |
| KLHL24 | LINC00847 | 0.378199217 | 1.22E-06 |
| MAP2K7 | LINC00847 | 0.366417465 | 2.74E-06 |
| MAPK9 | LINC00847 | 0.571299442 | 8.32E-15 |
| MTMR14 | LINC00847 | 0.314689873 | 6.66E-05 |
| NBR1 | LINC00847 | 0.362631246 | 3.53E-06 |
| NKX2-3 | LINC00847 | 0.346713374 | 9.89E-06 |
| NRG1 | LINC00847 | -0.322622483 | 4.24E-05 |
| PARP1 | LINC00847 | 0.347852706 | 9.21E-06 |
| PTK6 | LINC00847 | -0.406005881 | 1.59E-07 |
| RB1CC1 | LINC00847 | 0.334109626 | 2.15E-05 |
| SQSTM1 | LINC00847 | 0.307722357 | 9.82E-05 |
| TP53 | LINC00847 | 0.465690729 | 1.02E-09 |
| TSC2 | LINC00847 | 0.328416256 | 3.02E-05 |
| ULK1 | LINC00847 | 0.377133877 | 1.32E-06 |
| ULK2 | LINC00847 | 0.347077849 | 9.67E-06 |
| UVRAG | LINC00847 | 0.359549827 | 4.33E-06 |
| WDR45 | LINC00847 | 0.449529988 | 4.42E-09 |
| ATF4 | AC046143.2 | 0.368815603 | 2.33E-06 |
| ATG2B | AC046143.2 | -0.339479262 | 1.55E-05 |
| ATG3 | AC046143.2 | 0.35528179 | 5.72E-06 |
| ATG4B | AC046143.2 | 0.38258602 | 8.98E-07 |
| BAX | AC046143.2 | 0.398445362 | 2.83E-07 |
| BNIP1 | AC046143.2 | 0.386080226 | 7.00E-07 |
| CAPN10 | AC046143.2 | 0.305721443 | 0.000109539 |
| DAPK2 | AC046143.2 | 0.387815725 | 6.17E-07 |
| DDIT3 | AC046143.2 | 0.343587948 | 1.20E-05 |
| NCKAP1 | AC046143.2 | -0.304639074 | 0.000116183 |
| NFKB1 | AC046143.2 | -0.31436855 | 6.79E-05 |
| PELP1 | AC046143.2 | 0.49809861 | 4.26E-11 |
| PINK1 | AC046143.2 | -0.383898928 | 8.18E-07 |
| WDR45 | AC046143.2 | 0.314909649 | 6.58E-05 |
| ARNT | AC093627.4 | 0.360163764 | 4.16E-06 |
| ATG10 | AC093627.4 | 0.339403183 | 1.56E-05 |
| ATG3 | AC093627.4 | 0.310671776 | 8.34E-05 |
| CDKN2A | AC093627.4 | 0.343418735 | 1.22E-05 |
| DAPK1 | AC093627.4 | 0.343701796 | 1.19E-05 |
| IKBKE | AC093627.4 | 0.451594411 | 3.68E-09 |
| MTMR14 | AC093627.4 | 0.31533538 | 6.43E-05 |
| PARP1 | AC093627.4 | 0.348681695 | 8.74E-06 |
| RAF1 | AC093627.4 | 0.383754752 | 8.26E-07 |
| TP53 | AC093627.4 | 0.328410965 | 3.02E-05 |
| TP73 | AC093627.4 | 0.358401316 | 4.67E-06 |
| TSC2 | AC093627.4 | 0.351048279 | 7.52E-06 |
| ULK3 | AC093627.4 | 0.392423698 | 4.41E-07 |
| CAPN1 | AP002761.4 | 0.513340702 | 8.49E-12 |
| CAPN2 | AP002761.4 | 0.466319203 | 9.64E-10 |
| CAPNS1 | AP002761.4 | 0.333821986 | 2.19E-05 |
| PTK6 | AP002761.4 | 0.323215881 | 4.09E-05 |
| RAC1 | AP002761.4 | 0.361824513 | 3.73E-06 |
| ST13 | AP002761.4 | -0.338355883 | 1.66E-05 |
| ATG4C | AL390778.2 | 0.307342436 | 0.000100248 |
| ATG4D | AL390778.2 | 0.341860084 | 1.34E-05 |
| ATG7 | AL390778.2 | 0.418395408 | 6.05E-08 |
| BCL2 | AL390778.2 | 0.632236292 | 1.12E-18 |
| CAPNS1 | AL390778.2 | 0.322711757 | 4.22E-05 |
| CDKN1B | AL390778.2 | 0.422448429 | 4.37E-08 |
| CFLAR | AL390778.2 | 0.333752039 | 2.20E-05 |
| DRAM1 | AL390778.2 | 0.331609182 | 2.50E-05 |
| EEF2K | AL390778.2 | 0.342193589 | 1.31E-05 |
| FAS | AL390778.2 | 0.57899649 | 2.98E-15 |
| HDAC6 | AL390778.2 | 0.311241801 | 8.08E-05 |
| IKBKE | AL390778.2 | 0.489741425 | 9.99E-11 |
| MAP1LC3A | AL390778.2 | 0.31208734 | 7.71E-05 |
| MAPK8 | AL390778.2 | 0.546074154 | 2.00E-13 |
| NKX2-3 | AL390778.2 | 0.761195103 | 1.41E-30 |
| PARP1 | AL390778.2 | 0.358734368 | 4.57E-06 |
| PRKAB1 | AL390778.2 | 0.522561631 | 3.07E-12 |
| TP53 | AL390778.2 | 0.387512629 | 6.31E-07 |
| TP73 | AL390778.2 | 0.561293832 | 3.03E-14 |
| TSC2 | AL390778.2 | 0.615507972 | 1.57E-17 |
| WDR45 | AL390778.2 | 0.344681784 | 1.12E-05 |
| ATG16L2 | AC020916.1 | 0.42915937 | 2.52E-08 |
| ATG4B | AC020916.1 | 0.482276317 | 2.10E-10 |
| CAPN10 | AC020916.1 | 0.442463639 | 8.20E-09 |
| CXCR4 | AC020916.1 | 0.38621272 | 6.93E-07 |
| DAPK2 | AC020916.1 | 0.326300529 | 3.42E-05 |
| FOS | AC020916.1 | 0.467896754 | 8.32E-10 |
| PPP1R15A | AC020916.1 | 0.356676745 | 5.23E-06 |
| RAB24 | AC020916.1 | 0.342840382 | 1.26E-05 |
| STK11 | AC020916.1 | 0.315866302 | 6.24E-05 |
| TSC2 | AC020916.1 | 0.353299613 | 6.51E-06 |
| WDFY3 | AC020916.1 | -0.318565286 | 5.35E-05 |
| CALCOCO2 | PSMB8-AS1 | 0.457702759 | 2.13E-09 |
| CCR2 | PSMB8-AS1 | 0.362167348 | 3.65E-06 |
| CXCR4 | PSMB8-AS1 | 0.312990359 | 7.33E-05 |
| IFNG | PSMB8-AS1 | 0.456811352 | 2.31E-09 |
| RGS19 | PSMB8-AS1 | 0.419797941 | 5.40E-08 |
| ATG16L2 | AC022706.1 | 0.31625465 | 6.10E-05 |
| CAPN10 | AC022706.1 | 0.39449793 | 3.79E-07 |
| DAPK2 | AC022706.1 | 0.389625542 | 5.41E-07 |
| DIRAS3 | AC022706.1 | 0.49471633 | 6.03E-11 |
| GABARAP | AC022706.1 | 0.455676784 | 2.56E-09 |
| PELP1 | AC022706.1 | 0.458300772 | 2.02E-09 |
| ULK3 | AC022706.1 | 0.30600516 | 0.000107857 |
| ATG16L2 | AC010883.1 | 0.302851335 | 0.000127988 |
| ATG4B | AC010883.1 | 0.415048585 | 7.89E-08 |
| BAX | AC010883.1 | 0.367232603 | 2.60E-06 |
| BID | AC010883.1 | 0.351578595 | 7.27E-06 |
| BIRC5 | AC010883.1 | 0.326309019 | 3.42E-05 |
| BNIP1 | AC010883.1 | 0.3006004 | 0.000144445 |
| CAPN10 | AC010883.1 | 0.478161999 | 3.13E-10 |
| CXCR4 | AC010883.1 | 0.509409091 | 1.30E-11 |
| DAPK2 | AC010883.1 | 0.329058358 | 2.91E-05 |
| FKBP1A | AC010883.1 | 0.381733222 | 9.54E-07 |
| HDAC6 | AC010883.1 | 0.34776125 | 9.26E-06 |
| HIF1A | AC010883.1 | -0.341948504 | 1.33E-05 |
| IKBKB | AC010883.1 | 0.304481673 | 0.000117181 |
| ITGA3 | AC010883.1 | -0.325589925 | 3.57E-05 |
| MTMR14 | AC010883.1 | 0.329664485 | 2.80E-05 |
| RAB24 | AC010883.1 | 0.331622179 | 2.50E-05 |
| STK11 | AC010883.1 | 0.387805214 | 6.18E-07 |
| ULK3 | AC010883.1 | 0.334406553 | 2.11E-05 |
| WDFY3 | AC010883.1 | -0.311683304 | 7.89E-05 |
| WDR45 | AC010883.1 | 0.422593931 | 4.31E-08 |
| ZFYVE1 | AC010883.1 | -0.353893801 | 6.26E-06 |
| BNIP3 | FLJ46906 | 0.529239118 | 1.45E-12 |
| MTOR | FLJ46906 | -0.32158238 | 4.50E-05 |
| NFKB1 | FLJ46906 | -0.381058277 | 1.00E-06 |
| PEX3 | FLJ46906 | 0.421583279 | 4.68E-08 |
| ATG16L2 | AC004918.1 | 0.378168525 | 1.23E-06 |
| ATG4B | AC004918.1 | 0.38217252 | 9.24E-07 |
| BAX | AC004918.1 | 0.374731122 | 1.56E-06 |
| CAPN10 | AC004918.1 | 0.549501923 | 1.32E-13 |
| CXCR4 | AC004918.1 | 0.400325486 | 2.45E-07 |
| DAPK2 | AC004918.1 | 0.301169654 | 0.000140106 |
| HIF1A | AC004918.1 | -0.303499896 | 0.000123582 |
| IKBKB | AC004918.1 | 0.303933819 | 0.000120714 |
| MTMR14 | AC004918.1 | 0.350520973 | 7.77E-06 |
| RAB24 | AC004918.1 | 0.382959331 | 8.74E-07 |
| STK11 | AC004918.1 | 0.354849931 | 5.89E-06 |
| TSC2 | AC004918.1 | 0.33398579 | 2.17E-05 |
| ULK3 | AC004918.1 | 0.470533317 | 6.49E-10 |
| WDR45 | AC004918.1 | 0.313049678 | 7.31E-05 |
| ATG10 | AL355338.1 | 0.307721139 | 9.82E-05 |
| ATG4B | AL355338.1 | 0.32348676 | 4.03E-05 |
| ATG4D | AL355338.1 | 0.460942433 | 1.58E-09 |
| BAX | AL355338.1 | 0.313407022 | 7.16E-05 |
| BID | AL355338.1 | 0.442698996 | 8.03E-09 |
| CAPN10 | AL355338.1 | 0.348422372 | 8.88E-06 |
| CAPNS1 | AL355338.1 | 0.356460538 | 5.30E-06 |
| CDKN2A | AL355338.1 | 0.4043293 | 1.81E-07 |
| CLN3 | AL355338.1 | 0.307278398 | 0.000100601 |
| IKBKE | AL355338.1 | 0.415679076 | 7.50E-08 |
| ITGA3 | AL355338.1 | -0.400284548 | 2.46E-07 |
| MTMR14 | AL355338.1 | 0.391803956 | 4.62E-07 |
| NRG1 | AL355338.1 | -0.308115198 | 9.61E-05 |
| PINK1 | AL355338.1 | -0.499243503 | 3.79E-11 |
| PRKAB1 | AL355338.1 | 0.391272323 | 4.80E-07 |
| ZFYVE1 | AL355338.1 | -0.309279861 | 9.01E-05 |
| ARNT | AC005034.5 | 0.42930272 | 2.49E-08 |
| ARSB | AC005034.5 | 0.367884005 | 2.49E-06 |
| ATF6 | AC005034.5 | 0.31421796 | 6.84E-05 |
| ATG4C | AC005034.5 | 0.314437497 | 6.76E-05 |
| BCL2 | AC005034.5 | 0.342884605 | 1.26E-05 |
| BIRC6 | AC005034.5 | 0.444850036 | 6.66E-09 |
| CAMKK2 | AC005034.5 | 0.324790861 | 3.74E-05 |
| CANX | AC005034.5 | 0.321048463 | 4.64E-05 |
| CCR2 | AC005034.5 | 0.388054178 | 6.07E-07 |
| CD46 | AC005034.5 | 0.412820786 | 9.40E-08 |
| CDKN1B | AC005034.5 | 0.372655056 | 1.80E-06 |
| DRAM1 | AC005034.5 | 0.383628637 | 8.34E-07 |
| EEF2K | AC005034.5 | 0.389393952 | 5.51E-07 |
| EIF2AK3 | AC005034.5 | 0.335235704 | 2.01E-05 |
| FAS | AC005034.5 | 0.427601836 | 2.87E-08 |
| HDAC6 | AC005034.5 | 0.310638025 | 8.36E-05 |
| ITPR1 | AC005034.5 | 0.449558649 | 4.41E-09 |
| LAMP2 | AC005034.5 | 0.311477816 | 7.98E-05 |
| MAPK1 | AC005034.5 | 0.326261772 | 3.43E-05 |
| MAPK8 | AC005034.5 | 0.444235078 | 7.03E-09 |
| MAPK9 | AC005034.5 | 0.329610409 | 2.81E-05 |
| MTOR | AC005034.5 | 0.420752841 | 5.01E-08 |
| NBR1 | AC005034.5 | 0.450091879 | 4.21E-09 |
| NKX2-3 | AC005034.5 | 0.303310236 | 0.000124856 |
| PARP1 | AC005034.5 | 0.421471198 | 4.72E-08 |
| PTEN | AC005034.5 | 0.344185608 | 1.16E-05 |
| RAB33B | AC005034.5 | 0.336231395 | 1.89E-05 |
| RB1 | AC005034.5 | 0.334508382 | 2.10E-05 |
| RB1CC1 | AC005034.5 | 0.340202704 | 1.48E-05 |
| SH3GLB1 | AC005034.5 | 0.358036192 | 4.79E-06 |
| SIRT1 | AC005034.5 | 0.422343274 | 4.40E-08 |
| TP53 | AC005034.5 | 0.363261045 | 3.39E-06 |
| TP73 | AC005034.5 | 0.366988027 | 2.64E-06 |
| ULK2 | AC005034.5 | 0.329377942 | 2.85E-05 |
| UVRAG | AC005034.5 | 0.341459339 | 1.37E-05 |
| ATF4 | AC139530.1 | 0.3273454 | 3.22E-05 |
| ATG16L2 | AC139530.1 | 0.312802328 | 7.41E-05 |
| ATG3 | AC139530.1 | 0.32851854 | 3.00E-05 |
| ATG4B | AC139530.1 | 0.379051682 | 1.15E-06 |
| ATG9B | AC139530.1 | 0.360709905 | 4.01E-06 |
| ATIC | AC139530.1 | 0.394983436 | 3.66E-07 |
| BAG1 | AC139530.1 | 0.310936949 | 8.22E-05 |
| BAX | AC139530.1 | 0.485820939 | 1.48E-10 |
| BIRC5 | AC139530.1 | 0.553560385 | 8.00E-14 |
| BNIP1 | AC139530.1 | 0.318822827 | 5.27E-05 |
| CAPN10 | AC139530.1 | 0.473210455 | 5.04E-10 |
| CLN3 | AC139530.1 | 0.3734708 | 1.70E-06 |
| DLC1 | AC139530.1 | -0.308416447 | 9.45E-05 |
| GABARAPL2 | AC139530.1 | 0.310162486 | 8.58E-05 |
| HDAC6 | AC139530.1 | 0.350603149 | 7.73E-06 |
| ITGA3 | AC139530.1 | -0.363580005 | 3.32E-06 |
| ITGA6 | AC139530.1 | -0.369104971 | 2.29E-06 |
| ITGB1 | AC139530.1 | -0.335390864 | 1.99E-05 |
| ITGB4 | AC139530.1 | -0.301712713 | 0.000136081 |
| MAPK8IP1 | AC139530.1 | 0.46094877 | 1.58E-09 |
| MLST8 | AC139530.1 | 0.352013186 | 7.07E-06 |
| PINK1 | AC139530.1 | -0.305875649 | 0.000108622 |
| RAB24 | AC139530.1 | 0.340318072 | 1.47E-05 |
| ULK3 | AC139530.1 | 0.3149778 | 6.56E-05 |
| WDR45 | AC139530.1 | 0.441602006 | 8.83E-09 |
| ARNT | AC006206.2 | 0.373481847 | 1.70E-06 |
| ARSB | AC006206.2 | 0.325222995 | 3.64E-05 |
| BIRC6 | AC006206.2 | 0.300808463 | 0.000142845 |
| CD46 | AC006206.2 | 0.319193028 | 5.16E-05 |
| CDKN1B | AC006206.2 | 0.341660574 | 1.36E-05 |
| EEF2K | AC006206.2 | 0.314588662 | 6.70E-05 |
| GAA | AC006206.2 | 0.316679106 | 5.96E-05 |
| GABARAPL1 | AC006206.2 | 0.33312983 | 2.28E-05 |
| KLHL24 | AC006206.2 | 0.378042404 | 1.24E-06 |
| LAMP2 | AC006206.2 | 0.341950264 | 1.33E-05 |
| MBTPS2 | AC006206.2 | 0.322180028 | 4.35E-05 |
| NBR1 | AC006206.2 | 0.340026843 | 1.50E-05 |
| TP73 | AC006206.2 | 0.31374512 | 7.03E-05 |
| ULK1 | AC006206.2 | 0.337484044 | 1.75E-05 |
| KLHL24 | LINC00987 | 0.341700795 | 1.35E-05 |
| ATF6 | AL359962.1 | 0.392660833 | 4.34E-07 |
| GABARAPL1 | AL359962.1 | 0.411868778 | 1.01E-07 |
| KLHL24 | AL359962.1 | 0.367212643 | 2.60E-06 |
| MBTPS2 | AL359962.1 | 0.36300589 | 3.45E-06 |
| NCKAP1 | AL359962.1 | 0.306967909 | 0.000102327 |
| PEA15 | AL359962.1 | 0.302042333 | 0.00013369 |
| SQSTM1 | AL359962.1 | 0.3476092 | 9.35E-06 |
| TP63 | AL359962.1 | 0.308421001 | 9.45E-05 |
| ULK1 | AL359962.1 | 0.305554801 | 0.000110538 |
| UVRAG | AL359962.1 | 0.401386985 | 2.27E-07 |
| ATG2A | AP001330.5 | -0.314849903 | 6.61E-05 |
| CANX | AP001330.5 | -0.325927466 | 3.50E-05 |
| FAS | AP001330.5 | -0.311745721 | 7.86E-05 |
| MAPK8 | AP001330.5 | -0.368069697 | 2.46E-06 |
| MAPK9 | AP001330.5 | -0.325543697 | 3.58E-05 |
| MTOR | AP001330.5 | -0.382704423 | 8.90E-07 |
| NFKB1 | AP001330.5 | -0.340669835 | 1.44E-05 |
| PARP1 | AP001330.5 | -0.330342642 | 2.69E-05 |
| TP73 | AP001330.5 | -0.357744684 | 4.88E-06 |
| ARNT | SNHG25 | -0.39681828 | 3.19E-07 |
| ATF4 | SNHG25 | 0.480593585 | 2.47E-10 |
| ATG16L2 | SNHG25 | 0.332248134 | 2.40E-05 |
| ATG2B | SNHG25 | -0.328601046 | 2.99E-05 |
| ATG4B | SNHG25 | 0.433870983 | 1.70E-08 |
| ATIC | SNHG25 | 0.406123668 | 1.58E-07 |
| BAX | SNHG25 | 0.468285953 | 8.02E-10 |
| BIRC5 | SNHG25 | 0.589769474 | 6.79E-16 |
| BIRC6 | SNHG25 | -0.408539461 | 1.31E-07 |
| BNIP1 | SNHG25 | 0.441949832 | 8.57E-09 |
| CAPN10 | SNHG25 | 0.448764868 | 4.73E-09 |
| CAPN2 | SNHG25 | -0.399879499 | 2.54E-07 |
| CHMP4B | SNHG25 | 0.307758475 | 9.80E-05 |
| CLN3 | SNHG25 | 0.309726208 | 8.79E-05 |
| EIF4EBP1 | SNHG25 | 0.3204074 | 4.81E-05 |
| EIF4G1 | SNHG25 | -0.334109306 | 2.15E-05 |
| FKBP1A | SNHG25 | 0.387802897 | 6.18E-07 |
| FOXO1 | SNHG25 | -0.319444416 | 5.09E-05 |
| FOXO3 | SNHG25 | -0.300214107 | 0.00014746 |
| GABARAPL2 | SNHG25 | 0.40713503 | 1.46E-07 |
| GOPC | SNHG25 | -0.329234427 | 2.88E-05 |
| ITGA6 | SNHG25 | -0.318794674 | 5.28E-05 |
| ITGB1 | SNHG25 | -0.308320896 | 9.50E-05 |
| KIF5B | SNHG25 | -0.343840609 | 1.18E-05 |
| LAMP2 | SNHG25 | -0.350936285 | 7.57E-06 |
| MAPK1 | SNHG25 | -0.453480065 | 3.11E-09 |
| MAPK8IP1 | SNHG25 | 0.490380159 | 9.36E-11 |
| MLST8 | SNHG25 | 0.407860733 | 1.38E-07 |
| MTOR | SNHG25 | -0.323537874 | 4.02E-05 |
| NCKAP1 | SNHG25 | -0.378083582 | 1.23E-06 |
| RAB24 | SNHG25 | 0.357734832 | 4.88E-06 |
| TP63 | SNHG25 | -0.303503942 | 0.000123555 |
| WDFY3 | SNHG25 | -0.389782531 | 5.35E-07 |
| WDR45 | SNHG25 | 0.438513216 | 1.15E-08 |
| ZFYVE1 | SNHG25 | -0.324141141 | 3.88E-05 |
| ARNT | AL162413.1 | -0.313655904 | 7.06E-05 |
| ARSB | AL162413.1 | -0.323245409 | 4.09E-05 |
| BNIP1 | AL162413.1 | 0.306031229 | 0.000107703 |
| CASP1 | AL162413.1 | 0.492873205 | 7.28E-11 |
| CASP4 | AL162413.1 | 0.360479814 | 4.08E-06 |
| EIF2AK3 | AL162413.1 | -0.306637269 | 0.000104196 |
| GOPC | AL162413.1 | -0.304167437 | 0.000119195 |
| NCKAP1 | AL162413.1 | -0.322643957 | 4.23E-05 |
| PRKCD | AL162413.1 | -0.305622822 | 0.000110129 |
| SIRT1 | AL162413.1 | -0.314881218 | 6.59E-05 |
| TM9SF1 | AL162413.1 | -0.356244674 | 5.38E-06 |
| BCL2 | LINC01560 | 0.318585189 | 5.34E-05 |
| CD46 | LINC01560 | 0.315524327 | 6.36E-05 |
| HDAC6 | LINC01560 | 0.627539008 | 2.38E-18 |
| IKBKB | LINC01560 | 0.432216524 | 1.96E-08 |
| ITGA3 | LINC01560 | -0.353898833 | 6.26E-06 |
| KLHL24 | LINC01560 | 0.421977557 | 4.53E-08 |
| MAPK8IP1 | LINC01560 | 0.376718303 | 1.36E-06 |
| MAPK9 | LINC01560 | 0.324818491 | 3.73E-05 |
| MBTPS2 | LINC01560 | 0.337971552 | 1.70E-05 |
| NBR1 | LINC01560 | 0.311760515 | 7.85E-05 |
| PARP1 | LINC01560 | 0.372447176 | 1.82E-06 |
| PRKAR1A | LINC01560 | 0.418282147 | 6.10E-08 |
| RB1CC1 | LINC01560 | 0.37228217 | 1.84E-06 |
| SIRT1 | LINC01560 | 0.31745093 | 5.70E-05 |
| ST13 | LINC01560 | 0.323825309 | 3.95E-05 |
| ULK2 | LINC01560 | 0.341842043 | 1.34E-05 |
| UVRAG | LINC01560 | 0.332279914 | 2.40E-05 |
| VAMP7 | LINC01560 | 0.406623392 | 1.52E-07 |
| ATG10 | LINC01315 | 0.382380071 | 9.11E-07 |
| ATG12 | LINC01315 | 0.34170727 | 1.35E-05 |
| ATG4B | LINC01315 | 0.327860727 | 3.12E-05 |
| ATG4C | LINC01315 | 0.421847259 | 4.58E-08 |
| ATG4D | LINC01315 | 0.332674125 | 2.34E-05 |
| ATG7 | LINC01315 | 0.368142628 | 2.44E-06 |
| BAX | LINC01315 | 0.330798199 | 2.62E-05 |
| BCL2 | LINC01315 | 0.50791159 | 1.52E-11 |
| BNIP1 | LINC01315 | 0.451376322 | 3.76E-09 |
| CAPN10 | LINC01315 | 0.363688272 | 3.29E-06 |
| CAPNS1 | LINC01315 | 0.323425512 | 4.04E-05 |
| CASP3 | LINC01315 | 0.302644376 | 0.000129425 |
| CDKN1B | LINC01315 | 0.373639906 | 1.68E-06 |
| CDKN2A | LINC01315 | 0.429941803 | 2.36E-08 |
| CFLAR | LINC01315 | 0.33656075 | 1.85E-05 |
| CXCR4 | LINC01315 | 0.605198389 | 7.38E-17 |
| DAPK2 | LINC01315 | 0.306947613 | 0.000102441 |
| DRAM1 | LINC01315 | 0.6143848 | 1.86E-17 |
| EIF4G1 | LINC01315 | -0.313567391 | 7.10E-05 |
| FAS | LINC01315 | 0.564212005 | 2.09E-14 |
| FKBP1A | LINC01315 | 0.364250059 | 3.17E-06 |
| GABARAP | LINC01315 | 0.339864313 | 1.51E-05 |
| HDAC6 | LINC01315 | 0.317216069 | 5.78E-05 |
| HIF1A | LINC01315 | -0.331598448 | 2.50E-05 |
| IFNG | LINC01315 | 0.337998705 | 1.70E-05 |
| IKBKE | LINC01315 | 0.498056878 | 4.28E-11 |
| ITGA6 | LINC01315 | -0.325953082 | 3.49E-05 |
| MAP1LC3A | LINC01315 | 0.386892836 | 6.60E-07 |
| MAPK8 | LINC01315 | 0.445764258 | 6.15E-09 |
| MAPK9 | LINC01315 | 0.331276412 | 2.55E-05 |
| MTMR14 | LINC01315 | 0.444226381 | 7.04E-09 |
| MYC | LINC01315 | -0.375387605 | 1.49E-06 |
| NKX2-3 | LINC01315 | 0.500431389 | 3.35E-11 |
| PARP1 | LINC01315 | 0.315878696 | 6.23E-05 |
| PEX14 | LINC01315 | 0.322177406 | 4.35E-05 |
| PRKAB1 | LINC01315 | 0.389939499 | 5.29E-07 |
| PTK6 | LINC01315 | -0.316857756 | 5.90E-05 |
| RAB1A | LINC01315 | -0.34557414 | 1.06E-05 |
| RAB24 | LINC01315 | 0.312893059 | 7.37E-05 |
| SIRT1 | LINC01315 | 0.317699657 | 5.62E-05 |
| STK11 | LINC01315 | 0.358329826 | 4.69E-06 |
| TP53 | LINC01315 | 0.492094893 | 7.88E-11 |
| TP73 | LINC01315 | 0.595610937 | 2.97E-16 |
| TSC2 | LINC01315 | 0.593075287 | 4.26E-16 |
| USP10 | LINC01315 | -0.304893629 | 0.000114588 |
| WDR45 | LINC01315 | 0.346803061 | 9.84E-06 |
| ATG12 | AL365330.1 | 0.41556889 | 7.57E-08 |
| ATG16L2 | AL365330.1 | 0.421196858 | 4.83E-08 |
| ATG4B | AL365330.1 | 0.330917544 | 2.60E-05 |
| ATG4C | AL365330.1 | 0.352212666 | 6.98E-06 |
| CAPN10 | AL365330.1 | 0.411381973 | 1.05E-07 |
| CASP8 | AL365330.1 | 0.31529583 | 6.44E-05 |
| CFLAR | AL365330.1 | 0.389417723 | 5.50E-07 |
| CXCR4 | AL365330.1 | 0.487044553 | 1.31E-10 |
| EEF2K | AL365330.1 | 0.325150945 | 3.66E-05 |
| FAS | AL365330.1 | 0.304926778 | 0.000114381 |
| HDAC1 | AL365330.1 | 0.321571199 | 4.50E-05 |
| HDAC6 | AL365330.1 | 0.420855762 | 4.96E-08 |
| IKBKB | AL365330.1 | 0.434751448 | 1.58E-08 |
| MAP2K7 | AL365330.1 | 0.352365831 | 6.91E-06 |
| MAPK8 | AL365330.1 | 0.347429555 | 9.46E-06 |
| MAPK9 | AL365330.1 | 0.329971615 | 2.75E-05 |
| MTOR | AL365330.1 | 0.313132396 | 7.27E-05 |
| NKX2-3 | AL365330.1 | 0.321416408 | 4.54E-05 |
| PEX14 | AL365330.1 | 0.381097786 | 9.98E-07 |
| RAF1 | AL365330.1 | 0.383632093 | 8.33E-07 |
| STK11 | AL365330.1 | 0.347629026 | 9.34E-06 |
| TP73 | AL365330.1 | 0.42321936 | 4.10E-08 |
| TSC1 | AL365330.1 | 0.401865807 | 2.18E-07 |
| TSC2 | AL365330.1 | 0.50142859 | 3.02E-11 |
| ULK3 | AL365330.1 | 0.390469681 | 5.09E-07 |
| UVRAG | AL365330.1 | 0.309410145 | 8.95E-05 |
| WDR45 | AL365330.1 | 0.330408087 | 2.68E-05 |
| ATF4 | Z95115.1 | 0.306735298 | 0.000103638 |
| ATG4B | Z95115.1 | 0.323513456 | 4.02E-05 |
| ATIC | Z95115.1 | 0.419673001 | 5.46E-08 |
| BNIP1 | Z95115.1 | 0.309343922 | 8.98E-05 |
| CAPN10 | Z95115.1 | 0.381959025 | 9.39E-07 |
| HDAC6 | Z95115.1 | 0.324850098 | 3.72E-05 |
| ITGB4 | Z95115.1 | -0.3184371 | 5.39E-05 |
| MAPK8IP1 | Z95115.1 | 0.374181493 | 1.62E-06 |
| PRKAB1 | Z95115.1 | 0.333589674 | 2.22E-05 |
| WDR45 | Z95115.1 | 0.356140623 | 5.41E-06 |
| ARSB | NUP50-DT | -0.328676799 | 2.97E-05 |
| ATF4 | NUP50-DT | 0.442911845 | 7.88E-09 |
| ATG16L1 | NUP50-DT | 0.302823017 | 0.000128184 |
| ATG4B | NUP50-DT | 0.316777985 | 5.92E-05 |
| ATG9B | NUP50-DT | 0.312384238 | 7.58E-05 |
| CANX | NUP50-DT | -0.384352789 | 7.92E-07 |
| CAPN10 | NUP50-DT | 0.300337765 | 0.000146489 |
| DDIT3 | NUP50-DT | 0.341815008 | 1.34E-05 |
| ITPR1 | NUP50-DT | -0.323487562 | 4.03E-05 |
| MTOR | NUP50-DT | -0.436730092 | 1.34E-08 |
| PTEN | NUP50-DT | -0.362338341 | 3.60E-06 |
| ATG4D | PITPNA-AS1 | 0.393974436 | 3.94E-07 |
| ATIC | PITPNA-AS1 | 0.328700671 | 2.97E-05 |
| BAX | PITPNA-AS1 | 0.345415797 | 1.07E-05 |
| BIRC5 | PITPNA-AS1 | 0.328575421 | 2.99E-05 |
| CAPN10 | PITPNA-AS1 | 0.365985641 | 2.83E-06 |
| CLN3 | PITPNA-AS1 | 0.320430198 | 4.81E-05 |
| FKBP1A | PITPNA-AS1 | 0.32087648 | 4.69E-05 |
| GABARAP | PITPNA-AS1 | 0.533614863 | 8.73E-13 |
| MAPK8IP1 | PITPNA-AS1 | 0.323516266 | 4.02E-05 |
| PELP1 | PITPNA-AS1 | 0.581353015 | 2.17E-15 |
| PTK6 | PITPNA-AS1 | -0.304102542 | 0.000119615 |
| WDR45 | PITPNA-AS1 | 0.499512987 | 3.68E-11 |
| APOL1 | U62317.1 | 0.311038571 | 8.17E-05 |
| BIRC6 | U62317.1 | -0.393913164 | 3.96E-07 |
| CASP1 | U62317.1 | 0.330324512 | 2.70E-05 |
| CTSD | U62317.1 | 0.304878642 | 0.000114681 |
| DDIT3 | U62317.1 | 0.33249837 | 2.37E-05 |
| EEF2 | U62317.1 | -0.355779399 | 5.54E-06 |
| EIF2AK3 | U62317.1 | -0.333505342 | 2.23E-05 |
| FOXO3 | U62317.1 | -0.326449477 | 3.39E-05 |
| MBTPS2 | U62317.1 | -0.308962992 | 9.17E-05 |
| SESN2 | U62317.1 | 0.317647192 | 5.64E-05 |
| SPNS1 | U62317.1 | -0.303090013 | 0.00012635 |
| ATG9B | AC011476.3 | 0.317689658 | 5.62E-05 |
| BECN1 | AC011476.3 | 0.316018064 | 6.18E-05 |
| EIF2AK3 | AC011476.3 | 0.308167305 | 9.58E-05 |
| SPNS1 | AC011476.3 | 0.313947251 | 6.95E-05 |
| HIF1A | AC132807.2 | 0.32703496 | 3.28E-05 |
| ATG4D | LINC01503 | 0.306290863 | 0.000106187 |
| BNIP1 | LINC01503 | 0.332130055 | 2.42E-05 |
| CANX | LINC01503 | -0.300622143 | 0.000144277 |
| CAPNS1 | LINC01503 | 0.345577443 | 1.06E-05 |
| CDKN2A | LINC01503 | 0.32461467 | 3.77E-05 |
| CHMP4B | LINC01503 | 0.396486137 | 3.27E-07 |
| CLN3 | LINC01503 | 0.331890459 | 2.46E-05 |
| CTSD | LINC01503 | 0.351172118 | 7.46E-06 |
| IKBKE | LINC01503 | 0.328230506 | 3.05E-05 |
| MAP1LC3A | LINC01503 | 0.412908861 | 9.33E-08 |
| RAC1 | LINC01503 | 0.386348984 | 6.86E-07 |
| WDR45 | LINC01503 | 0.446922449 | 5.56E-09 |
| ATG12 | PCAT6 | -0.310133592 | 8.60E-05 |
| ATG4C | PCAT6 | -0.382801992 | 8.84E-07 |
| ATG7 | PCAT6 | -0.386704503 | 6.69E-07 |
| CALCOCO2 | PCAT6 | -0.31474886 | 6.64E-05 |
| DRAM1 | PCAT6 | -0.318694903 | 5.31E-05 |
| FAS | PCAT6 | -0.407859448 | 1.38E-07 |
| MAPK8 | PCAT6 | -0.314485268 | 6.74E-05 |
| MAPK9 | PCAT6 | -0.309442506 | 8.93E-05 |
| MTMR14 | PCAT6 | -0.353631576 | 6.37E-06 |
| NFKB1 | PCAT6 | -0.329884618 | 2.77E-05 |
| RAF1 | PCAT6 | -0.305931731 | 0.00010829 |
| TP73 | PCAT6 | -0.305186799 | 0.000112775 |
| ATG3 | AC083799.1 | 0.591491103 | 5.33E-16 |
| NFE2L2 | AC083799.1 | 0.410387472 | 1.14E-07 |
| PIK3R4 | AC083799.1 | 0.40297611 | 2.01E-07 |
| RAB7A | AC083799.1 | 0.497845957 | 4.37E-11 |
| RHEB | AC000123.1 | 0.367283371 | 2.59E-06 |
| ATG10 | AC098484.1 | 0.374142456 | 1.62E-06 |
| ATG2A | AC098484.1 | 0.300373831 | 0.000146206 |
| ATG4C | AC098484.1 | 0.323663379 | 3.99E-05 |
| EEF2K | AC098484.1 | 0.350319175 | 7.87E-06 |
| HDAC6 | AC098484.1 | 0.346854215 | 9.81E-06 |
| MAPK8 | AC098484.1 | 0.334870722 | 2.05E-05 |
| MTOR | AC098484.1 | 0.468295859 | 8.01E-10 |
| PEX14 | AC098484.1 | 0.309594369 | 8.86E-05 |
| PRKAB1 | AC098484.1 | 0.300808222 | 0.000142847 |
| TP73 | AC098484.1 | 0.471584368 | 5.88E-10 |
| TSC2 | AC098484.1 | 0.371128959 | 1.99E-06 |
| ITGA3 | AC007342.5 | -0.349163197 | 8.47E-06 |
| TP53INP2 | AC007342.5 | 0.30507166 | 0.000113484 |
| DNAJB1 | AL445673.1 | 0.342450133 | 1.29E-05 |
| KLHL24 | AL445673.1 | 0.303636119 | 0.000122675 |
| ATG4B | AL121772.3 | 0.367980381 | 2.47E-06 |
| CAPN10 | AL121772.3 | 0.35083963 | 7.62E-06 |
| PINK1 | AL121772.3 | -0.319239227 | 5.15E-05 |
| ARSB | AC104083.1 | 0.547379245 | 1.71E-13 |
| DAPK1 | AC104083.1 | 0.369973277 | 2.16E-06 |
| DLC1 | AC104083.1 | 0.516003641 | 6.35E-12 |
| FOXO1 | AC104083.1 | 0.352106845 | 7.02E-06 |
| ITPR1 | AC104083.1 | 0.315995569 | 6.19E-05 |
| MAPK1 | AC104083.1 | 0.301177468 | 0.000140048 |
| NBR1 | AC104083.1 | 0.31529287 | 6.44E-05 |
| BAX | AC034213.1 | 0.332595482 | 2.35E-05 |
| BNIP1 | AC034213.1 | 0.572406243 | 7.19E-15 |
| DDIT3 | AC034213.1 | 0.499949491 | 3.52E-11 |
| FKBP1A | AC034213.1 | 0.308792495 | 9.26E-05 |
| MAPK1 | AC034213.1 | -0.305695602 | 0.000109693 |
| RAB1A | AC034213.1 | -0.312532736 | 7.52E-05 |
| RAC1 | AC034213.1 | 0.324724572 | 3.75E-05 |
| ATG4A | AL133215.2 | 0.307102273 | 0.000101577 |
| ATIC | AL133215.2 | 0.395483692 | 3.52E-07 |
| BAX | AL133215.2 | 0.450113183 | 4.20E-09 |
| BIRC5 | AL133215.2 | 0.599239115 | 1.76E-16 |
| BNIP1 | AL133215.2 | 0.40767652 | 1.40E-07 |
| CAPN10 | AL133215.2 | 0.34109697 | 1.40E-05 |
| CLN3 | AL133215.2 | 0.378650917 | 1.18E-06 |
| FKBP1A | AL133215.2 | 0.399000965 | 2.71E-07 |
| HDAC6 | AL133215.2 | 0.379735468 | 1.10E-06 |
| ITGB4 | AL133215.2 | -0.326095433 | 3.46E-05 |
| MAPK8IP1 | AL133215.2 | 0.517450099 | 5.42E-12 |
| MLST8 | AL133215.2 | 0.351444646 | 7.33E-06 |
| MTMR14 | AL133215.2 | 0.303761385 | 0.000121846 |
| WDR45 | AL133215.2 | 0.526573666 | 1.96E-12 |
| ATF4 | AC107952.2 | 0.353707813 | 6.34E-06 |
| ATG10 | AC107952.2 | 0.325375848 | 3.61E-05 |
| ATG16L2 | AC107952.2 | 0.32256341 | 4.25E-05 |
| ATG4B | AC107952.2 | 0.395915946 | 3.41E-07 |
| ATG4C | AC107952.2 | 0.384786455 | 7.67E-07 |
| ATG4D | AC107952.2 | 0.342328038 | 1.30E-05 |
| BAG3 | AC107952.2 | -0.300940825 | 0.000141836 |
| BAX | AC107952.2 | 0.395565958 | 3.50E-07 |
| BCL2 | AC107952.2 | 0.365260611 | 2.97E-06 |
| BID | AC107952.2 | 0.301397838 | 0.000138402 |
| BNIP1 | AC107952.2 | 0.324691296 | 3.76E-05 |
| CAPN10 | AC107952.2 | 0.528244483 | 1.62E-12 |
| CASP3 | AC107952.2 | 0.309386197 | 8.96E-05 |
| CDKN1B | AC107952.2 | 0.304535017 | 0.000116842 |
| CXCR4 | AC107952.2 | 0.451541034 | 3.70E-09 |
| DIRAS3 | AC107952.2 | 0.467183622 | 8.89E-10 |
| DRAM1 | AC107952.2 | 0.321566659 | 4.50E-05 |
| EIF4G1 | AC107952.2 | -0.338272101 | 1.67E-05 |
| GABARAP | AC107952.2 | 0.373999247 | 1.64E-06 |
| HDAC6 | AC107952.2 | 0.309121043 | 9.09E-05 |
| IKBKB | AC107952.2 | 0.31419513 | 6.85E-05 |
| MAP1LC3A | AC107952.2 | 0.355639713 | 5.59E-06 |
| MAP2K7 | AC107952.2 | 0.383523349 | 8.40E-07 |
| MAPK8 | AC107952.2 | 0.305862229 | 0.000108701 |
| MTMR14 | AC107952.2 | 0.372546564 | 1.81E-06 |
| PELP1 | AC107952.2 | 0.387098318 | 6.50E-07 |
| RAF1 | AC107952.2 | 0.334103001 | 2.15E-05 |
| STK11 | AC107952.2 | 0.416651185 | 6.95E-08 |
| TP53 | AC107952.2 | 0.324399862 | 3.82E-05 |
| TSC2 | AC107952.2 | 0.382756035 | 8.87E-07 |
| USP10 | AC107952.2 | -0.304145628 | 0.000119336 |
| ATG10 | AC005332.3 | 0.340503953 | 1.46E-05 |
| ATG12 | AC005332.3 | 0.326986048 | 3.29E-05 |
| ATG4B | AC005332.3 | 0.334261597 | 2.13E-05 |
| ATG4C | AC005332.3 | 0.38070409 | 1.03E-06 |
| ATG4D | AC005332.3 | 0.307504517 | 9.94E-05 |
| ATIC | AC005332.3 | 0.30972804 | 8.79E-05 |
| BAX | AC005332.3 | 0.324869733 | 3.72E-05 |
| BIRC5 | AC005332.3 | 0.391166054 | 4.84E-07 |
| BNIP1 | AC005332.3 | 0.306560382 | 0.000104635 |
| CAPN10 | AC005332.3 | 0.452291179 | 3.46E-09 |
| CDKN1B | AC005332.3 | 0.364901881 | 3.04E-06 |
| CXCR4 | AC005332.3 | 0.386464368 | 6.80E-07 |
| DAPK2 | AC005332.3 | 0.32608386 | 3.46E-05 |
| FKBP1A | AC005332.3 | 0.311266926 | 8.07E-05 |
| GABARAP | AC005332.3 | 0.38572119 | 7.18E-07 |
| HDAC6 | AC005332.3 | 0.487768413 | 1.22E-10 |
| ITGA3 | AC005332.3 | -0.303104436 | 0.000126252 |
| ITGA6 | AC005332.3 | -0.318006322 | 5.52E-05 |
| MAPK8 | AC005332.3 | 0.339299898 | 1.57E-05 |
| MAPK8IP1 | AC005332.3 | 0.425325261 | 3.46E-08 |
| MAPK9 | AC005332.3 | 0.350453529 | 7.81E-06 |
| MTMR14 | AC005332.3 | 0.400057526 | 2.50E-07 |
| PARP1 | AC005332.3 | 0.336918811 | 1.81E-05 |
| PELP1 | AC005332.3 | 0.342170467 | 1.31E-05 |
| PRKAB1 | AC005332.3 | 0.301323613 | 0.000138954 |
| PRKAR1A | AC005332.3 | 0.376037828 | 1.42E-06 |
| PTK6 | AC005332.3 | -0.350772444 | 7.65E-06 |
| RAF1 | AC005332.3 | 0.330951921 | 2.60E-05 |
| STK11 | AC005332.3 | 0.306006212 | 0.000107851 |
| TP73 | AC005332.3 | 0.328019337 | 3.09E-05 |
| TSC2 | AC005332.3 | 0.328156314 | 3.07E-05 |
| ULK2 | AC005332.3 | 0.366449956 | 2.74E-06 |
| ULK3 | AC005332.3 | 0.346594984 | 9.97E-06 |
| WDR45 | AC005332.3 | 0.430013215 | 2.35E-08 |
| ATG10 | AL390719.2 | 0.336949215 | 1.81E-05 |
| ATG16L2 | AL390719.2 | 0.496923035 | 4.81E-11 |
| ATG3 | AL390719.2 | 0.354367973 | 6.07E-06 |
| ATG4B | AL390719.2 | 0.530298205 | 1.28E-12 |
| BAX | AL390719.2 | 0.451728447 | 3.64E-09 |
| CAPN10 | AL390719.2 | 0.648694263 | 7.07E-20 |
| CAPNS1 | AL390719.2 | 0.332312266 | 2.40E-05 |
| CDKN2A | AL390719.2 | 0.363632943 | 3.31E-06 |
| CLN3 | AL390719.2 | 0.37475964 | 1.55E-06 |
| DLC1 | AL390719.2 | -0.381479489 | 9.71E-07 |
| FOXO1 | AL390719.2 | -0.311823337 | 7.83E-05 |
| HDAC1 | AL390719.2 | 0.392677888 | 4.33E-07 |
| IKBKB | AL390719.2 | 0.353416959 | 6.46E-06 |
| ITGB1 | AL390719.2 | -0.309362702 | 8.97E-05 |
| MAP1LC3A | AL390719.2 | 0.303338289 | 0.000124667 |
| MAP2K7 | AL390719.2 | 0.35822175 | 4.73E-06 |
| MAPK1 | AL390719.2 | -0.318974665 | 5.23E-05 |
| MLST8 | AL390719.2 | 0.328839011 | 2.95E-05 |
| MTMR14 | AL390719.2 | 0.392783331 | 4.30E-07 |
| PEX14 | AL390719.2 | 0.445447741 | 6.33E-09 |
| RAB24 | AL390719.2 | 0.432614652 | 1.89E-08 |
| STK11 | AL390719.2 | 0.472792746 | 5.24E-10 |
| ULK3 | AL390719.2 | 0.451438134 | 3.74E-09 |
| WDR45 | AL390719.2 | 0.410868083 | 1.09E-07 |
| ATG16L2 | AC015871.3 | 0.306161846 | 0.000106938 |
| BAK1 | AC015871.3 | -0.317948144 | 5.54E-05 |
| CAPN10 | AC015871.3 | 0.365789203 | 2.86E-06 |
| CXCR4 | AC015871.3 | 0.414991757 | 7.92E-08 |
| EIF2AK3 | AC015871.3 | 0.336630405 | 1.85E-05 |
| HDAC6 | AC015871.3 | 0.37373264 | 1.67E-06 |
| IKBKB | AC015871.3 | 0.357451935 | 4.97E-06 |
| ITGA3 | AC015871.3 | -0.363008911 | 3.45E-06 |
| KLHL24 | AC015871.3 | 0.32410271 | 3.89E-05 |
| MAP2K7 | AC015871.3 | 0.322867197 | 4.18E-05 |
| NFE2L2 | AC015871.3 | 0.304550155 | 0.000116746 |
| PRKAB1 | AC015871.3 | 0.348932746 | 8.60E-06 |
| RAF1 | AC015871.3 | 0.304194455 | 0.000119021 |
| RB1CC1 | AC015871.3 | 0.354868111 | 5.88E-06 |
| SIRT1 | AC015871.3 | 0.306457777 | 0.000105223 |
| TSC1 | AC015871.3 | 0.323915849 | 3.93E-05 |
| ULK3 | AC015871.3 | 0.417481817 | 6.50E-08 |
| UVRAG | AC015871.3 | 0.43973756 | 1.04E-08 |
| ARNT | AC026471.1 | 0.316359413 | 6.07E-05 |
| BIRC6 | AC026471.1 | 0.39870388 | 2.77E-07 |
| EEF2K | AC026471.1 | 0.320719971 | 4.73E-05 |
| EIF2AK3 | AC026471.1 | 0.315571321 | 6.34E-05 |
| EIF4G1 | AC026471.1 | 0.334980863 | 2.04E-05 |
| SAR1A | AC026471.1 | -0.41310508 | 9.19E-08 |
| ATG10 | LINC01547 | 0.399815189 | 2.55E-07 |
| ATG4C | LINC01547 | 0.437168952 | 1.29E-08 |
| ATG7 | LINC01547 | 0.306583765 | 0.000104501 |
| CDKN2A | LINC01547 | 0.395485204 | 3.52E-07 |
| CXCR4 | LINC01547 | 0.37918574 | 1.14E-06 |
| HDAC6 | LINC01547 | 0.321133385 | 4.62E-05 |
| IKBKE | LINC01547 | 0.419829902 | 5.39E-08 |
| MAP1LC3B | LINC01547 | -0.330720271 | 2.63E-05 |
| MTMR14 | LINC01547 | 0.408062192 | 1.36E-07 |
| PARP1 | LINC01547 | 0.377065646 | 1.32E-06 |
| RAF1 | LINC01547 | 0.38780518 | 6.18E-07 |
| TP53 | LINC01547 | 0.392357143 | 4.44E-07 |
| TP73 | LINC01547 | 0.473288879 | 5.00E-10 |
| TSC2 | LINC01547 | 0.418586292 | 5.95E-08 |
| ULK3 | LINC01547 | 0.312263257 | 7.64E-05 |
| ARSB | AL133371.2 | 0.415782394 | 7.44E-08 |
| ATG4C | AL133371.2 | 0.338469357 | 1.65E-05 |
| ATG7 | AL133371.2 | 0.317253886 | 5.77E-05 |
| CCL2 | AL133371.2 | 0.320580132 | 4.77E-05 |
| CCR2 | AL133371.2 | 0.67037582 | 1.42E-21 |
| CTSB | AL133371.2 | 0.333026735 | 2.29E-05 |
| CTSD | AL133371.2 | 0.377964526 | 1.24E-06 |
| CXCR4 | AL133371.2 | 0.404973865 | 1.73E-07 |
| DAPK1 | AL133371.2 | 0.325073086 | 3.67E-05 |
| DRAM1 | AL133371.2 | 0.381739413 | 9.53E-07 |
| GABARAP | AL133371.2 | 0.348390943 | 8.90E-06 |
| IFNG | AL133371.2 | 0.375909825 | 1.43E-06 |
| ITPR1 | AL133371.2 | 0.433266598 | 1.79E-08 |
| MTMR14 | AL133371.2 | 0.31144896 | 7.99E-05 |
| RGS19 | AL133371.2 | 0.479713372 | 2.69E-10 |
| SERPINA1 | AL133371.2 | 0.46701608 | 9.03E-10 |
| ATF4 | AC002116.2 | 0.313645984 | 7.07E-05 |
| ATG10 | AC002116.2 | 0.322590628 | 4.25E-05 |
| ATG3 | AC002116.2 | 0.326870614 | 3.31E-05 |
| ATG4B | AC002116.2 | 0.363338025 | 3.37E-06 |
| ATIC | AC002116.2 | 0.399572257 | 2.60E-07 |
| BAG3 | AC002116.2 | -0.319383849 | 5.11E-05 |
| BAX | AC002116.2 | 0.45406833 | 2.95E-09 |
| BIRC5 | AC002116.2 | 0.570289169 | 9.50E-15 |
| BNIP1 | AC002116.2 | 0.417505745 | 6.49E-08 |
| CAPN10 | AC002116.2 | 0.401639631 | 2.22E-07 |
| CDKN1B | AC002116.2 | 0.348573713 | 8.80E-06 |
| CDKN2A | AC002116.2 | 0.370721664 | 2.05E-06 |
| CXCR4 | AC002116.2 | 0.453183434 | 3.20E-09 |
| FKBP1A | AC002116.2 | 0.325055107 | 3.68E-05 |
| HDAC6 | AC002116.2 | 0.398247999 | 2.87E-07 |
| IKBKB | AC002116.2 | 0.329073971 | 2.90E-05 |
| MAP2K7 | AC002116.2 | 0.317942869 | 5.54E-05 |
| MAPK8IP1 | AC002116.2 | 0.486389669 | 1.40E-10 |
| MLST8 | AC002116.2 | 0.321418491 | 4.54E-05 |
| MTMR14 | AC002116.2 | 0.426361324 | 3.17E-08 |
| PELP1 | AC002116.2 | 0.339277905 | 1.57E-05 |
| PINK1 | AC002116.2 | -0.371462204 | 1.95E-06 |
| RAB1A | AC002116.2 | -0.321381248 | 4.55E-05 |
| RAF1 | AC002116.2 | 0.317134974 | 5.80E-05 |
| STK11 | AC002116.2 | 0.346845829 | 9.81E-06 |
| TP53 | AC002116.2 | 0.347029054 | 9.70E-06 |
| TSC2 | AC002116.2 | 0.300459565 | 0.000145537 |
| WDFY3 | AC002116.2 | -0.343736436 | 1.19E-05 |
| WDR45 | AC002116.2 | 0.452002668 | 3.55E-09 |
| ARSB | AC008556.1 | -0.303316634 | 0.000124813 |
| ATG4C | AC008556.1 | -0.34237354 | 1.30E-05 |
| CANX | AC008556.1 | -0.30679457 | 0.000103303 |
| DRAM1 | AC008556.1 | -0.32825218 | 3.05E-05 |
| FAS | AC008556.1 | -0.302861226 | 0.00012792 |
| GOPC | AC008556.1 | -0.308419679 | 9.45E-05 |
| MAPK8 | AC008556.1 | -0.309147702 | 9.08E-05 |
| MAPK9 | AC008556.1 | -0.310797272 | 8.29E-05 |
| PTK6 | AC008556.1 | 0.308916152 | 9.19E-05 |
| TP53 | AC008556.1 | -0.308591523 | 9.36E-05 |
| CASP8 | LINC01128 | 0.361773653 | 3.74E-06 |
| CFLAR | LINC01128 | 0.354240771 | 6.12E-06 |
| HIF1A | LINC01128 | 0.357454773 | 4.97E-06 |
| MTOR | LINC01128 | 0.304192004 | 0.000119036 |
| PEX14 | LINC01128 | 0.342381392 | 1.30E-05 |
| VAMP3 | LINC01128 | 0.606058021 | 6.50E-17 |
| CD46 | HAGLR | 0.363298664 | 3.38E-06 |
| EIF2AK3 | HAGLR | 0.308875495 | 9.21E-05 |
| HDAC6 | HAGLR | 0.306371155 | 0.000105723 |
| KLHL24 | HAGLR | 0.442389289 | 8.25E-09 |
| PARP1 | HAGLR | 0.32546185 | 3.59E-05 |
| PIK3R4 | HAGLR | 0.305009075 | 0.000113871 |
| VAMP7 | HAGLR | 0.343681939 | 1.20E-05 |
| ATF4 | MYLK-AS1 | 0.315270389 | 6.45E-05 |
| ATG3 | MYLK-AS1 | 0.331205652 | 2.56E-05 |
| ATG4B | MYLK-AS1 | 0.435260053 | 1.52E-08 |
| ATG4D | MYLK-AS1 | 0.320525422 | 4.78E-05 |
| ATIC | MYLK-AS1 | 0.327295835 | 3.23E-05 |
| BAX | MYLK-AS1 | 0.344635451 | 1.13E-05 |
| BID | MYLK-AS1 | 0.365919524 | 2.84E-06 |
| BIRC5 | MYLK-AS1 | 0.365676003 | 2.88E-06 |
| BNIP1 | MYLK-AS1 | 0.405800934 | 1.62E-07 |
| CANX | MYLK-AS1 | -0.305693099 | 0.000109708 |
| CAPN10 | MYLK-AS1 | 0.397061169 | 3.13E-07 |
| FKBP1A | MYLK-AS1 | 0.343067384 | 1.24E-05 |
| LAMP2 | MYLK-AS1 | -0.351134028 | 7.48E-06 |
| MAP1LC3A | MYLK-AS1 | 0.337156246 | 1.79E-05 |
| PEX14 | MYLK-AS1 | 0.32253092 | 4.26E-05 |
| WDFY3 | MYLK-AS1 | -0.309887673 | 8.71E-05 |
| ZFYVE1 | MYLK-AS1 | -0.300387581 | 0.000146099 |
| IL24 | MYOSLID | 0.497503273 | 4.53E-11 |
| ITGA3 | MYOSLID | 0.414511163 | 8.23E-08 |
| ITGB4 | MYOSLID | 0.419221316 | 5.66E-08 |
| SERPINA1 | MYOSLID | 0.439705594 | 1.04E-08 |
| ATG4C | AC002310.1 | 0.317848726 | 5.57E-05 |
| ATIC | AC002310.1 | 0.514562525 | 7.43E-12 |
| BIRC5 | AC002310.1 | 0.321698306 | 4.47E-05 |
| CAPN10 | AC002310.1 | 0.325002231 | 3.69E-05 |
| CDKN1B | AC002310.1 | 0.302406865 | 0.000131092 |
| CLN3 | AC002310.1 | 0.389443638 | 5.49E-07 |
| HDAC6 | AC002310.1 | 0.332011631 | 2.44E-05 |
| ITGA3 | AC002310.1 | -0.315527434 | 6.36E-05 |
| MAPK8IP1 | AC002310.1 | 0.525080754 | 2.32E-12 |
| MLST8 | AC002310.1 | 0.449680553 | 4.36E-09 |
| PRKAR1A | AC002310.1 | 0.373064755 | 1.75E-06 |
| WDR45 | AC002310.1 | 0.306063112 | 0.000107516 |
| ARNT | YTHDF3-AS1 | -0.301201816 | 0.000139865 |
| BAX | YTHDF3-AS1 | 0.520306485 | 3.95E-12 |
| BIRC5 | YTHDF3-AS1 | 0.480994894 | 2.38E-10 |
| BNIP1 | YTHDF3-AS1 | 0.372474999 | 1.82E-06 |
| CAPN2 | YTHDF3-AS1 | -0.327764708 | 3.14E-05 |
| CLN3 | YTHDF3-AS1 | 0.315918951 | 6.22E-05 |
| EIF4EBP1 | YTHDF3-AS1 | 0.396948468 | 3.16E-07 |
| FKBP1A | YTHDF3-AS1 | 0.435991494 | 1.42E-08 |
| MAPK1 | YTHDF3-AS1 | -0.363823924 | 3.27E-06 |
| WDR45 | YTHDF3-AS1 | 0.368965759 | 2.31E-06 |
| FAS | AP005233.2 | 0.307817119 | 9.77E-05 |
| ATIC | MAFG-DT | 0.391870198 | 4.60E-07 |
| BAK1 | MAFG-DT | -0.369071207 | 2.29E-06 |
| CAPN2 | MAFG-DT | -0.342483939 | 1.29E-05 |
| CLN3 | MAFG-DT | 0.303681621 | 0.000122373 |
| DIRAS3 | MAFG-DT | 0.308561341 | 9.38E-05 |
| GNAI3 | MAFG-DT | -0.381846279 | 9.46E-07 |
| ITGA3 | MAFG-DT | -0.372178201 | 1.86E-06 |
| MAPK8IP1 | MAFG-DT | 0.389141959 | 5.61E-07 |
| MLST8 | MAFG-DT | 0.379692072 | 1.10E-06 |
| RPTOR | MAFG-DT | 0.322673722 | 4.23E-05 |
| VAMP3 | MAFG-DT | -0.317835794 | 5.58E-05 |
| WIPI1 | MAFG-DT | 0.337005522 | 1.80E-05 |
| ZFYVE1 | MAFG-DT | -0.307573279 | 9.90E-05 |
| BCL2L1 | AC025181.2 | 0.324282302 | 3.85E-05 |
| CAPN10 | AC025181.2 | 0.31418044 | 6.86E-05 |
| DIRAS3 | AC025181.2 | 0.372285728 | 1.84E-06 |
| GABARAPL2 | AC025181.2 | 0.384750396 | 7.69E-07 |
| HIF1A | AC025181.2 | -0.301902801 | 0.000134697 |
| EDEM1 | AC079949.1 | 0.333205768 | 2.27E-05 |
| HSP90AB1 | AC079949.1 | 0.309490878 | 8.91E-05 |
| HSPA8 | AC079949.1 | 0.309459679 | 8.92E-05 |
| ITGB1 | AC079949.1 | 0.484272867 | 1.72E-10 |
| ITGB4 | AC079949.1 | 0.392571848 | 4.37E-07 |
| MYC | AC079949.1 | 0.348043729 | 9.10E-06 |
| NAF1 | AC079949.1 | 0.387514848 | 6.31E-07 |
| USP10 | AC079949.1 | 0.3013869 | 0.000138483 |
| TP53 | AC055736.1 | -0.329637485 | 2.81E-05 |
| ATF4 | AL117379.1 | 0.321937942 | 4.41E-05 |
| ATG16L2 | AL117379.1 | 0.439245352 | 1.08E-08 |
| ATG3 | AL117379.1 | 0.418832395 | 5.84E-08 |
| ATG4B | AL117379.1 | 0.507239192 | 1.64E-11 |
| BAG3 | AL117379.1 | -0.327927867 | 3.11E-05 |
| BAX | AL117379.1 | 0.539511491 | 4.38E-13 |
| BIRC5 | AL117379.1 | 0.407807743 | 1.39E-07 |
| BIRC6 | AL117379.1 | -0.33714664 | 1.79E-05 |
| BNIP1 | AL117379.1 | 0.414337351 | 8.34E-08 |
| CANX | AL117379.1 | -0.300450404 | 0.000145609 |
| CAPN10 | AL117379.1 | 0.531284598 | 1.14E-12 |
| CDKN2A | AL117379.1 | 0.320667373 | 4.74E-05 |
| CXCR4 | AL117379.1 | 0.428125201 | 2.75E-08 |
| DLC1 | AL117379.1 | -0.32735863 | 3.21E-05 |
| EIF4G1 | AL117379.1 | -0.313122593 | 7.28E-05 |
| FKBP1A | AL117379.1 | 0.305118476 | 0.000113195 |
| HDAC6 | AL117379.1 | 0.301394714 | 0.000138425 |
| ITGA6 | AL117379.1 | -0.303176656 | 0.00012576 |
| ITGB1 | AL117379.1 | -0.308273104 | 9.53E-05 |
| KIF5B | AL117379.1 | -0.322310848 | 4.31E-05 |
| MAPK1 | AL117379.1 | -0.398784467 | 2.76E-07 |
| MTMR14 | AL117379.1 | 0.351372264 | 7.36E-06 |
| NCKAP1 | AL117379.1 | -0.343697316 | 1.19E-05 |
| PEX14 | AL117379.1 | 0.377217633 | 1.31E-06 |
| RAB24 | AL117379.1 | 0.485314022 | 1.55E-10 |
| STK11 | AL117379.1 | 0.44136287 | 9.01E-09 |
| ULK3 | AL117379.1 | 0.331197411 | 2.56E-05 |
| WDFY3 | AL117379.1 | -0.328806921 | 2.95E-05 |
| WDR45 | AL117379.1 | 0.491573085 | 8.30E-11 |
| ATG16L2 | AC011477.2 | 0.32444059 | 3.81E-05 |
| CXCR4 | AC011477.2 | 0.349051325 | 8.54E-06 |
| UVRAG | AC011477.2 | 0.325253791 | 3.64E-05 |
| GAA | AC092687.3 | 0.33388607 | 2.18E-05 |
| GABARAP | AL590705.3 | 0.308185738 | 9.57E-05 |
| KLHL24 | AL590705.3 | 0.38914397 | 5.61E-07 |
| PELP1 | AL590705.3 | 0.343962089 | 1.18E-05 |
| PINK1 | AL590705.3 | -0.327608391 | 3.17E-05 |
| RB1CC1 | AL590705.3 | 0.324084236 | 3.89E-05 |
| VAMP3 | AL590705.3 | -0.302830469 | 0.000128133 |
| ATF4 | AC016394.1 | 0.300809265 | 0.000142839 |
| ATIC | AC016394.1 | 0.404431837 | 1.80E-07 |
| BIRC5 | AC016394.1 | 0.307944324 | 9.70E-05 |
| BNIP1 | AC016394.1 | 0.300593762 | 0.000144496 |
| CAPN2 | AC016394.1 | -0.333546981 | 2.22E-05 |
| MAPK8IP1 | AC016394.1 | 0.371681512 | 1.92E-06 |
| ATF4 | APTR | 0.352166854 | 7.00E-06 |
| ATG4B | APTR | 0.31991468 | 4.95E-05 |
| ATG4D | APTR | 0.404522615 | 1.79E-07 |
| BCL2L1 | APTR | 0.341327885 | 1.38E-05 |
| BID | APTR | 0.424550992 | 3.68E-08 |
| CAPN10 | APTR | 0.361752843 | 3.75E-06 |
| CASP3 | APTR | 0.324604128 | 3.78E-05 |
| EIF2AK3 | APTR | 0.351612901 | 7.25E-06 |
| HDAC6 | APTR | 0.340019372 | 1.50E-05 |
| ITGA3 | APTR | -0.321212877 | 4.60E-05 |
| MAP1LC3A | APTR | 0.302792935 | 0.000128392 |
| NFE2L2 | APTR | 0.324427698 | 3.82E-05 |
| PINK1 | APTR | -0.435106333 | 1.53E-08 |
| PRKAB1 | APTR | 0.448269865 | 4.94E-09 |
| RAB33B | APTR | 0.389757215 | 5.36E-07 |
| RHEB | APTR | 0.400342681 | 2.45E-07 |
| ULK3 | APTR | 0.308713499 | 9.30E-05 |
| WIPI1 | APTR | 0.414442464 | 8.27E-08 |
| BID | AL138781.1 | 0.372432096 | 1.82E-06 |
| CANX | AL138781.1 | -0.320841808 | 4.70E-05 |
| CAPN10 | AL138781.1 | 0.308839454 | 9.23E-05 |
| FKBP1B | AL138781.1 | 0.313906012 | 6.97E-05 |
| FOXO1 | AL138781.1 | -0.304075931 | 0.000119788 |
| ATG3 | AC009065.5 | 0.309778895 | 8.77E-05 |
| ATG7 | LINC01980 | -0.302009425 | 0.000133927 |
| ATG12 | VIM-AS1 | 0.36507398 | 3.00E-06 |
| ATG4C | VIM-AS1 | 0.501882487 | 2.88E-11 |
| BCL2 | VIM-AS1 | 0.412071042 | 9.97E-08 |
| CASP3 | VIM-AS1 | 0.329177059 | 2.89E-05 |
| CCL2 | VIM-AS1 | 0.301503341 | 0.00013762 |
| CDKN1B | VIM-AS1 | 0.305502903 | 0.000110851 |
| CX3CL1 | VIM-AS1 | 0.343455555 | 1.21E-05 |
| CXCR4 | VIM-AS1 | 0.37926564 | 1.13E-06 |
| DRAM1 | VIM-AS1 | 0.328619812 | 2.98E-05 |
| FAS | VIM-AS1 | 0.33367038 | 2.21E-05 |
| HDAC6 | VIM-AS1 | 0.302276828 | 0.000132014 |
| ITPR1 | VIM-AS1 | 0.356182573 | 5.40E-06 |
| ATG16L2 | ERVH48-1 | 0.319743804 | 5.00E-05 |
| MYC | ERVH48-1 | 0.331430554 | 2.52E-05 |
| ATIC | RNASEH1-AS1 | 0.387967375 | 6.11E-07 |
| CAPN10 | RNASEH1-AS1 | 0.300223685 | 0.000147385 |
| CLN3 | RNASEH1-AS1 | 0.317769371 | 5.60E-05 |
| DIRAS3 | RNASEH1-AS1 | 0.372081624 | 1.87E-06 |
| MAPK8IP1 | RNASEH1-AS1 | 0.370446542 | 2.09E-06 |
| MLST8 | RNASEH1-AS1 | 0.370029344 | 2.15E-06 |
| PARP1 | RNASEH1-AS1 | 0.305956467 | 0.000108144 |
| PRKCQ | RNASEH1-AS1 | 0.33717018 | 1.79E-05 |
| RHEB | RNASEH1-AS1 | 0.319353858 | 5.11E-05 |
| ATIC | AC104986.2 | 0.315913328 | 6.22E-05 |
| BAX | AC104986.2 | 0.317194551 | 5.78E-05 |
| BID | AC104986.2 | 0.30011393 | 0.000148252 |
| BIRC5 | AC104986.2 | 0.346543665 | 1.00E-05 |
| DIRAS3 | AC104986.2 | 0.411436957 | 1.05E-07 |
| FKBP1B | AC104986.2 | 0.302613175 | 0.000129643 |
| GABARAPL2 | AC104986.2 | 0.507805586 | 1.54E-11 |
| MAPK8IP1 | AC104986.2 | 0.366741383 | 2.69E-06 |
| ATG12 | AC008735.2 | 0.316891342 | 5.89E-05 |
| ATG16L2 | AC008735.2 | 0.453823361 | 3.02E-09 |
| ATG4B | AC008735.2 | 0.302658343 | 0.000129328 |
| CAPN10 | AC008735.2 | 0.403412403 | 1.94E-07 |
| CASP8 | AC008735.2 | 0.311904129 | 7.79E-05 |
| CFLAR | AC008735.2 | 0.329936803 | 2.76E-05 |
| CXCR4 | AC008735.2 | 0.475756122 | 3.95E-10 |
| HDAC6 | AC008735.2 | 0.370033524 | 2.15E-06 |
| IKBKB | AC008735.2 | 0.443468205 | 7.51E-09 |
| TSC1 | AC008735.2 | 0.306290619 | 0.000106189 |
| TSC2 | AC008735.2 | 0.375826445 | 1.44E-06 |
| ULK3 | AC008735.2 | 0.340404098 | 1.47E-05 |
| UVRAG | AC008735.2 | 0.319074287 | 5.20E-05 |
| BAX | OGFRP1 | -0.385684127 | 7.20E-07 |
| BIRC6 | OGFRP1 | 0.43412168 | 1.67E-08 |
| BNIP1 | OGFRP1 | -0.424785937 | 3.61E-08 |
| EIF2AK3 | OGFRP1 | 0.341160747 | 1.40E-05 |
| EIF4G1 | OGFRP1 | 0.5541304 | 7.45E-14 |
| GABARAP | OGFRP1 | -0.338274416 | 1.67E-05 |
| MAPK1 | OGFRP1 | 0.556159594 | 5.79E-14 |
| NCKAP1 | OGFRP1 | 0.377961198 | 1.24E-06 |
| NFE2L2 | OGFRP1 | 0.33308096 | 2.29E-05 |
| NPC1 | OGFRP1 | 0.311539815 | 7.95E-05 |
| PEX14 | OGFRP1 | -0.355215308 | 5.75E-06 |
| RAB24 | OGFRP1 | -0.341251505 | 1.39E-05 |
| RPTOR | OGFRP1 | 0.370669459 | 2.06E-06 |
| TP63 | OGFRP1 | 0.36208444 | 3.67E-06 |
| USP10 | OGFRP1 | 0.329642641 | 2.81E-05 |
| WDFY3 | OGFRP1 | 0.335828147 | 1.94E-05 |
| ATG3 | AC016888.1 | 0.415022672 | 7.90E-08 |
| ATG4A | AC016888.1 | 0.340530053 | 1.45E-05 |
| ATG4D | AC016888.1 | 0.371790019 | 1.91E-06 |
| BECN1 | AC016888.1 | 0.42326128 | 4.09E-08 |
| CDKN2A | AC016888.1 | 0.405537828 | 1.65E-07 |
| DLC1 | AC016888.1 | -0.305212109 | 0.00011262 |
| EIF2AK3 | AC016888.1 | 0.398464141 | 2.82E-07 |
| NFE2L2 | AC016888.1 | 0.393879899 | 3.97E-07 |
| PTEN | AC016888.1 | -0.33521984 | 2.01E-05 |
| RAF1 | AC016888.1 | 0.330519848 | 2.67E-05 |
| ULK3 | AC016888.1 | 0.33044348 | 2.68E-05 |
| CAPN10 | AC087623.1 | 0.399797784 | 2.55E-07 |
| CXCR4 | AC087623.1 | 0.413902715 | 8.63E-08 |
| DIRAS3 | AC087623.1 | 0.62100882 | 6.69E-18 |
| UVRAG | AC087623.1 | 0.313462155 | 7.14E-05 |
| ATG3 | AL354754.1 | 0.344405418 | 1.14E-05 |
| CAPN2 | AL354754.1 | 0.359144021 | 4.45E-06 |
| EEF2K | AL354754.1 | 0.368100501 | 2.45E-06 |
| EIF2AK3 | AL354754.1 | 0.357928744 | 4.82E-06 |
| FOS | AL354754.1 | 0.353589681 | 6.39E-06 |
| HDAC1 | AL354754.1 | 0.324223355 | 3.86E-05 |
| SH3GLB1 | AL354754.1 | 0.310070645 | 8.63E-05 |
| ARSB | AC018647.2 | 0.307089417 | 0.000101648 |
| ATF4 | AC018647.2 | -0.3152704 | 6.45E-05 |
| ATG2B | AC018647.2 | 0.380436162 | 1.05E-06 |
| ATG4B | AC018647.2 | -0.353410482 | 6.46E-06 |
| GOPC | AC018647.2 | 0.332497168 | 2.37E-05 |
| MBTPS2 | AC018647.2 | 0.312902429 | 7.37E-05 |
| STK11 | AC018647.2 | -0.310028319 | 8.65E-05 |
| WIPI2 | AC018647.2 | 0.333483863 | 2.23E-05 |
